# Enabling sulfur-centered nucleophiles in conjunctive coupling: triethylborane-mediated trifluoromethylation *via* vinylboronate complexes

**DOI:** 10.1039/d6sc04418b

**Published:** 2026-07-07

**Authors:** Sufal Paul, Nayanthara T Anilkumar, Eluvathingal D. Jemmis, K. Geetharani

**Affiliations:** a Department of Inorganic and Physical Chemistry, Indian Institute of Science Bengaluru jemmis@iisc.ac.in geetharani@iisc.ac.in; b Department of Inorganic and Physical Chemistry, Indian Institute of Science Bengaluru

## Abstract

A three-component conjunctive coupling reaction of vinylboronic esters has been developed *via* a 1,2-boronate rearrangement involving sulfur-centered nucleophiles to construct highly functionalized tertiary alkyl boronic esters. Togni-II has been used as the electrophilic source of the CF_3_ group in this reaction. A broad array of substrates containing various functional groups has been well accommodated with good to excellent isolated yields, retaining the valuable boronic ester moiety in the products, which can be subjected to further functionalization reactions. The mechanistic studies suggested the formation of a thiolate-coordinated boron ‘ate’ complex and the crucial role of the boron Lewis acid BEt_3_ in activating the Togni-II reagent. Density functional theory studies suggested that the interaction between the sp^3^-boronate complex and the Togni-II–BEt_3_ adduct triggers the 1,2-shift of the thiol moiety, which leads to the formation of the coupling product.

## Abstract 2

A BEt_3_-mediated, metal-free three-component conjunctive coupling of vinylboronic esters with S-nucleophiles, *via* 1,2-boronate rearrangement, delivers CF_3_-substituted tertiary boronates in high yield under mild conditions.

## Graphical abstract

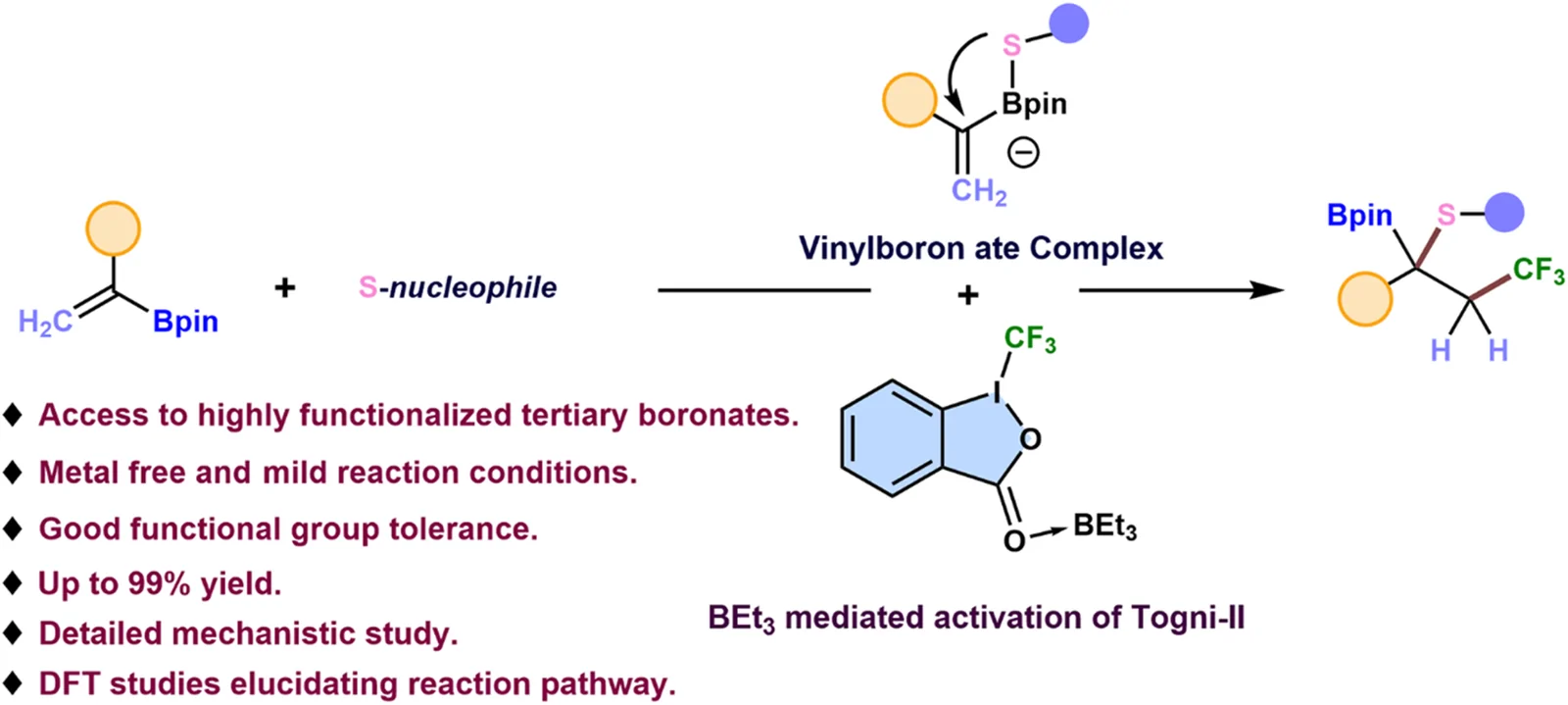

## Introduction

**Fig. 1:**
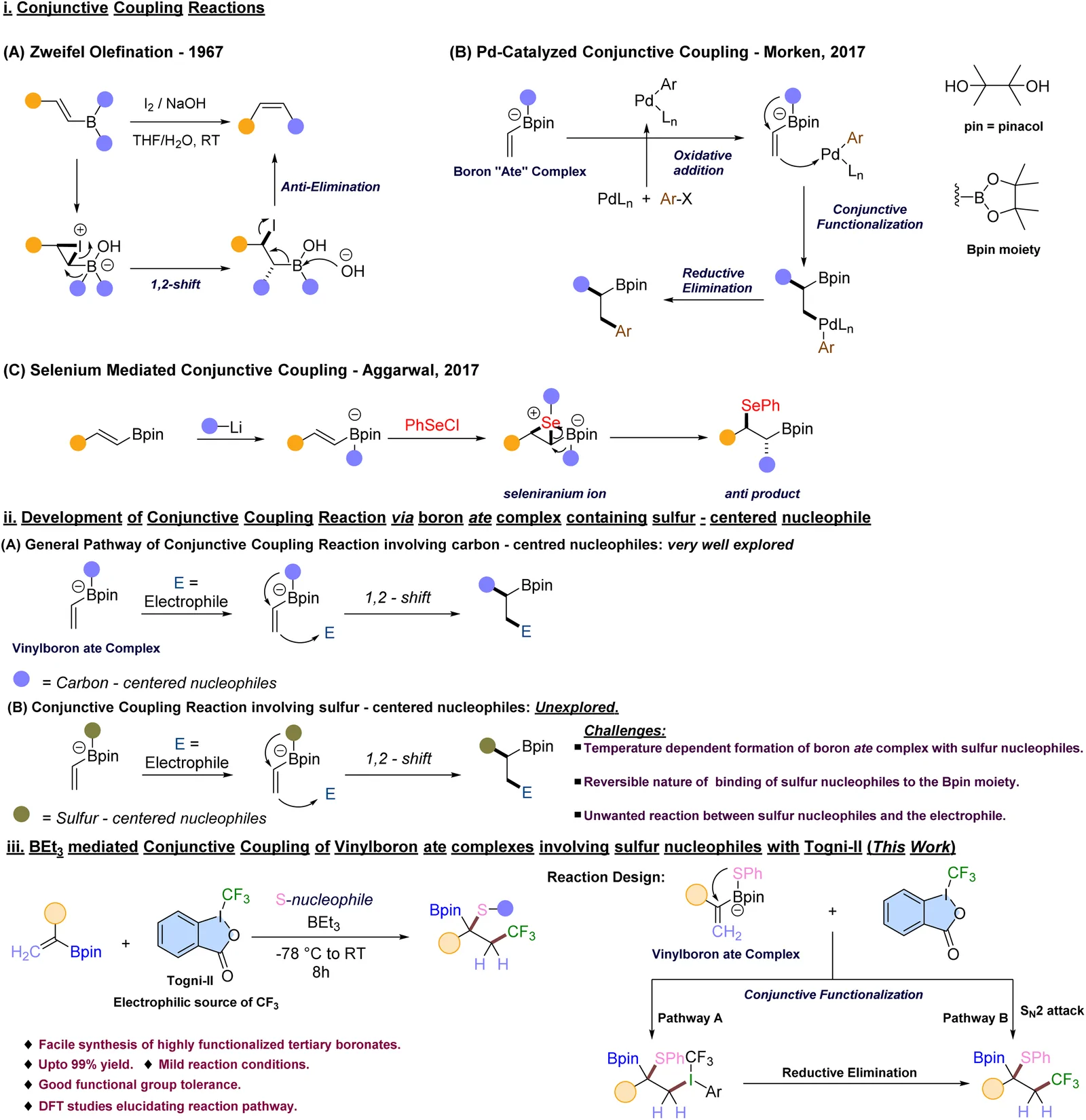
Conjunctive coupling reactions: (i, A–C): electrophile-induced conjunctive coupling; (ii, A): general pathway of conjunctive coupling; (ii, B): formation of the boron ate complex with sulfur-centered nucleophiles and conjunctive coupling with electrophile – key challenges; (iii): current work – hypervalent iodine-induced conjunctive coupling, reductive elimination pathway A and S_N_2 attack pathway B.

Organoboronic esters are important synthons in organic synthesis, as they can be subjected to various coupling reactions and can be easily converted to virtually any other functional group.^1–4^ Vinyl boronic esters are particularly important as they are used in Suzuki–Miyaura coupling reactions^5^ and, more recently, in three-component conjunctive coupling reactions, where they act as the conjunctive agent.^6^ The very first example of this kind of coupling reaction is the Zweifel olefination reaction (Fig. 1 i(A)). Herein, the vinyl boronic esters form the boron ‘ate’ complex with NaOH, and the π-system of the vinyl moiety interacts with iodine, inducing the 1,2-boronate shift.^7^ In recent years, the Morken group developed Pd-^8–15^ and Ni-^16–18^catalysed conjunctive coupling of vinyl boronates with aryl/alkyl halides or triflates (Fig. 1 i(B)). In 2017, the Aggarwal group,^19^ and in 2018, the Denmark group^20^ reported conjunctive coupling reactions involving seleniranium and thiiranium intermediates, respectively, to form β-seleno/thioboronic esters (Fig. 1 i(C)). A similar strategy was used by the Shen group, which employed electrophilic fluoroalkylthiolating reagents (*e.g.*, PhSO_2_SCF_2_H) to induce 1,2-migration of alkenylboronate complexes.^21^ Although the conjunctive coupling reactions are common with carbon-centred nucleophiles (such as organolithium or organomagnesium species forming a boronate complex with alkenyl boronic esters, followed by 1,2-migration) *via* both ionic^6^ and radical pathways,^22–24^ to the best of our knowledge, this strategy is unexplored with heteroatom-centered nucleophiles involving a 1,2-boronate shift. This approach is particularly intriguing, as it directly forms a carbon–heteroatom bond within an organoboronic ester. In our previous studies, we had demonstrated the 1,2-boronate rearrangement involving chalcogen nucleophiles.^25,26^ Thus, we envisaged the applicability of sulfur nucleophiles in the realm of conjunctive coupling reactions involving a 1,2-boronate shift. We selected the hypervalent iodine reagent, Togni-II (1-(trifluoromethyl)-1λ^3^-benzo[*d*][1,2]iodaoxol-3(1*H*)-one) (Fig. 1 iii) as the electrophilic coupling partner for the trifluoromethylation of vinyl boronates, due to the importance of the trifluoromethyl group in pharmaceutical chemistry.^27,28^ We envision that the attack of the thiolate nucleophile on the vinyl boronic ester would form the boronate complex. Then, the interaction between the π-bond of the boronate complex and the electrophilic Togni-II reagent would trigger the 1,2-shift to form the highly functionalized conjunctive coupling product. The presence of the trifluoromethyl group, the thioether moiety, along with the boronic ester functionality, would be an ideal synthetic handle. We must overcome several challenges in this attempt: (i) the coordination of sulfur nucleophiles like thiolate to the Bpin moiety is temperature-dependent, and the boronate complex formation is favoured at low temperatures,^25^ (ii) the reversible nature of binding can cause unwanted side reactions between the sulfur nucleophile and the trifluoromethylation reagent Togni-II. In this work, we successfully navigated these challenges to achieve the first example of a three-component conjunctive coupling of vinylboronates involving the 1,2-shift of sulfur nucleophiles. We demonstrate its versatility across a variety of substrates, and the mechanistic details of the reaction pathway are further elucidated by DFT studies.

## Results and discussion

We first describe the general precautions in the experiment and then optimise the reaction conditions. The scope of substrates and the usefulness of the reaction in synthetic chemistry are demonstrated next using several complex molecules. Reactions designed to give insights into the mechanistic pathways are analysed next. The results of DFT studies of selected steps are discussed at the end.

### Optimisation of reaction conditions

We anticipate that the generation of ˙CF_3_ and PhS˙ radicals upon the reaction of sulfur nucleophile and Togni-II reagent^29,30^ can be a significant challenge associated with the successful development of this conjunctive coupling reaction. These radicals can form several unwanted byproducts, such as PhSCF_3_,^4^ the hydrotrifluoromethylation product 3a and the thiol–ene product 3b (Table S1). To mitigate the undesired radical pathways, we adopted a sequential addition approach. The formation of the vinyl boronate complex by reacting the sulfur nucleophile with the vinyl boronic ester 1a at −78 °C is confirmed first. This is followed by the addition of the Togni-II reagent. We began the investigation of the three-component coupling reaction involving vinyl boronate 1a as a substrate, and the disulfide 2a and lithium triethylborohydride combination to *in situ* generate the thiolate nucleophile^31^ (1.1 eq.). To our delight, when THF was used as the solvent for this reaction along with DMF to dissolve the Togni-II reagent, we obtained 37% yield of our desired product 3aa (entry 2, Table 1), while the radical byproducts 3a and 3b did not form at all, and 4 formed only in trace amounts. The yield remained similar when THF was used as the sole solvent (entry 3, Table 1), but increased to 60% when MTBE (methyl *tert*-butyl ether) was used (entry 4, Table 1). Next, changing the solvent to CPME (cyclopentyl methyl ether) further increased the yield to 81% (entry 5, Table 1). The yield increased further to 94% when 1.5 eq. of PhSLi was used (entry 1, Table 1). When PhSH was used directly as the nucleophile instead of PhSLi, a drastic drop in the yield to 28% was observed (entry 7, Table 1). We speculated that while using the combination of diphenyl disulfide and LiHBEt_3_ to *in situ* generate PhSLi, the Lewis acid BEt_3_ was also generated, which might activate the Togni-II reagent. Thus, we decided to use thiol as a source of sulfur nucleophile in the presence of BEt_3_ (1.5 eq.) as an additive. Interestingly, the yield improved to 98%, indicating the crucial role of BEt_3_ in this reaction. The role of BEt_3_ was also confirmed when the reaction was carried out with isolated PhSLi, avoiding its use, offering only 25% yield (entry 9, Table 1). Finally, when the reaction was carried out at 0 °C, the yield decreased to 21%, and the amount of 4 increased to 12%, emphasising the role of the temperature in this reaction.

**Table 1:** Optimisation of reaction conditions^a^

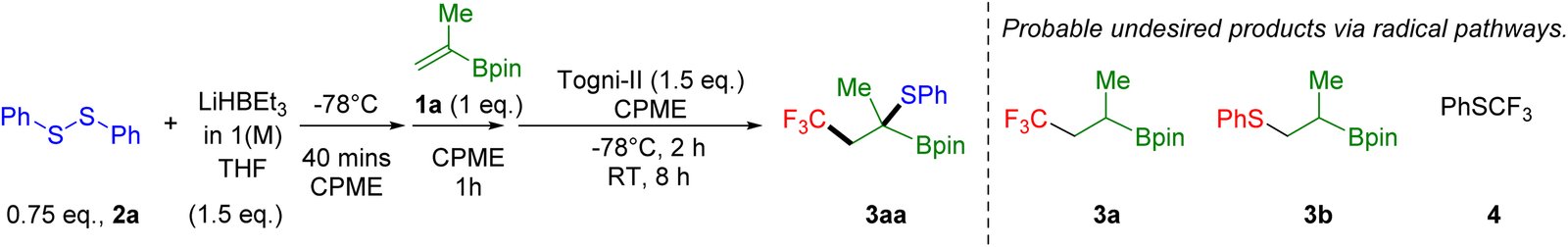			
Entry	Deviation from standard conditions	Yield (3aa) b(%)	Yield (4) b(%)
1	None	94%	n.d.
2	THF/DMF instead of CPME^c^ (1.1 eq. PhSLi^d^)	37%	<5%
3	THF instead of CPME as solvent (1.1 eq. PhSLi^d^)	40%	<5%
4	MTBE instead of CPME as solvent (1.1 eq. PhSLi^d^)	60%	<5%
5	CPME as solvent (1.1 eq. PhSLi^d^)	81%	<5%
6	0 °C instead of −78 °C	21%	12%
7	1.5 eq. PhSH as the nucleophile^e^	28%	<5%
8	Using 1.5 eq. PhSH and 1.5 eq. BEt_3_ additive^f^	98%	n.d.
9	Using 1.5 eq. isolated PhSLi^g^ without BEt_3_ additive	25%	<5%
10	Using 1.5 eq. isolated PhSLi^g^ with 1.5 eq. BEt_3_^f^	90%	n.d.

a Standard conditions: 2a (0.15 mmol, 0.75 eq.) in 0.2 ml CPME; 0.3 ml LiHBEt_3_ (1.5 eq. of 1 M solution in THF) added at −78 °C; stirred for 40 mins; 1a (0.2 mmol, 1 eq.) in 0.5 ml CPME added to the reaction at −78 °C; stirred for 1 h; the reaction mixture transferred to a 0.2 ml CPME solution of Togni-II (0.3 mmol, 1.5 eq.) at −78 °C; stirred for 2 h at −78 °C and stirred at RT for another 8 h.

b ^19^F NMR yields are reported using 3-trifluoromethylphenol as the internal standard.

c DMF was used to dissolve Togni-II, and the resulting solution was added to the reaction mixture.

d 1.1 eq. PhSLi was generated *in situ via* reacting 0.55 eq. (PhS)_2_ and 1.1 eq. (0.22 ml) LiHBEt_3_ (1 M solution in THF).

e Reaction was carried out in the absence of LiHBEt_3_.

f 0.3 ml (1.5 eq.) of 1 M BEt_3_ solution in THF was added as an additive.

g PhSLi was generated by the reaction of PhSH and ^*n*^BuLi.

### Scope of the reaction and usefulness in synthetic chemistry

**Fig. 2:**
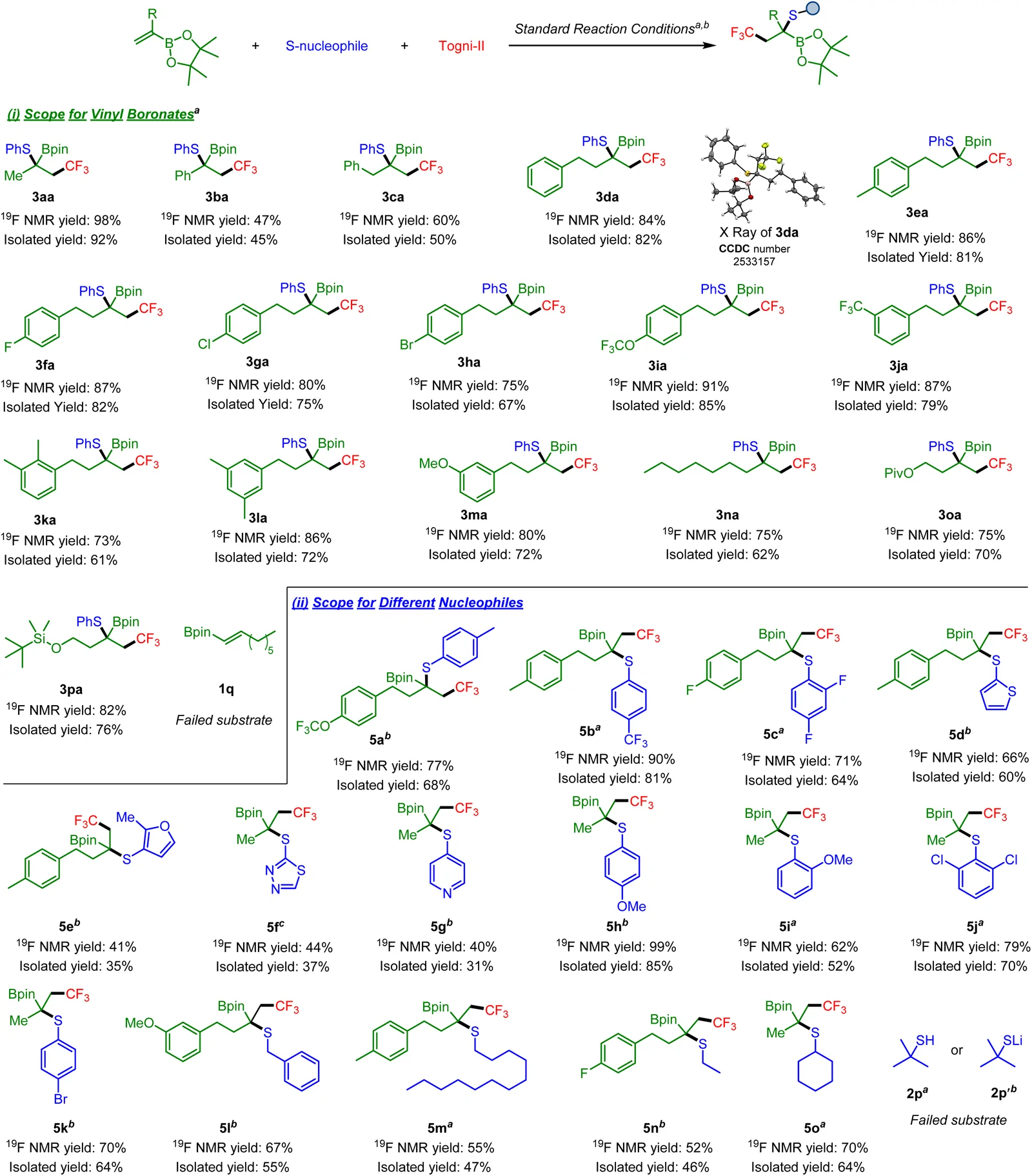
Substrate scope for vinyl boronates: (i) 3aa–3pa – for sulfur-nucleophiles: (ii) 5a–5o. ^*a*^Standard condition A: thiol (0.3 mmol) in 0.2 ml CPME; 0.3 ml BEt_3_ (1 M solution in THF) added at −78 °C; stirred for 40 mins; vinyl boronate (0.2 mmol) in 0.5 ml CPME added to the reaction at −78 °C; stirred for 1 h; reaction mixture transferred to a 0.2 ml CPME solution of Togni-II (0.3 mmol) at −78 °C; stirred for 2 h at −78 °C and stirred at RT for another 8 h. ^*b*^Standard condition B: disulfide (0.15 mmol) in 0.2 ml CPME; 0.3 ml LiHBEt_3_ (1 M solution in THF) added at −78 °C; stirred for 40 min; vinyl boronate (0.2 mmol) in 0.5 ml CPME added to the reaction at −78 °C; stirred for 1 h; reaction mixture transferred to a 0.2 ml CPME solution of Togni-II (0.3 mmol) at −78 °C; stirred for 2 h at −78 °C and stirred at RT for another 8 h. ^*c*^1,3,4-Thiadiazole-2-thiol in 0.2 ml DMF; 0.3 ml BEt_3_ (1 M solution in THF) added at −60 °C; stirred for 40 min; 1a (0.2 mmol) in 0.5 ml DMF added to the reaction at −60 °C; stirred for 1 h; reaction mixture transferred to a 0.2 ml DMF solution of Togni-II (0.3 mmol) at −60 °C; stirred for 2 h at −60 °C and stirred at RT for another 8 h.

With the optimised reaction conditions in hand, we explored the substrate scope of this reaction. A broad array of vinyl boronates was successfully used in this transformation. When the methyl group of vinyl boronate 1a was replaced with a phenyl group, the yield decreased to 47% (3ba, Fig. 2). When it was replaced with a benzyl group, in which the phenyl group is one carbon away from the double bond, the yield increased to 60% (3ca, Fig. 2). The yield increased to 84% when the phenyl group is two carbons away from the double bond (3da, Fig. 2). This trend can be explained by the observation that as the phenyl group moves farther away from the reaction centre, steric hindrance decreases, making the migration of the SPh group more facile. Next, the substituents on the phenyl group were changed, and the functional groups were well tolerated, giving good to excellent yields (3ea–3ma). The halide-containing vinyl boronates were well accommodated to provide the products 3fa–3ha in high yields. The ether linkage of the OCH_3_ and OCF_3_ groups was also compatible with this reaction to give excellent yields of 3ia and 3ma. The electron-withdrawing trifluoromethyl group was also well supported in forming the product with an 87% yield (3ja). As for the products 3ka and 3la, the yield was relatively lower for 3ka as the phenyl group was substituted at 2- and 3-positions. The completely aliphatic vinyl boronate also gave a good yield under these reaction conditions (3na). Delightfully, the ester group remained intact in the product 3oa due to the mild reaction conditions of this transformation, and the protected alcohol group was tolerated as well (3pa). Although this reaction worked very well with vinyl boronates having α-substitution, the internally substituted vinyl boronate 1q failed to undergo this reaction (any side products arising from hydrotrifluoromethylation or hydrothiolation were not observed; 1q remained unreacted). Next, we explored the compatibility of different sulfur nucleophiles in this reaction. The electronic effect of the thiol seemed to have little effect on the reaction, as thiophenols substituted with both the electron-donating CH_3_ and the electron-withdrawing CF_3_ gave good to excellent yields (5a, 5b). In contrast, the steric effect of the ortho-substituted thiophenol is clearly demonstrated in products 5h and 5i, where the yield decreased from 99% to 62% when the methoxy group was on the ortho-position instead of the para-position of the phenyl ring. The decrease in the yield indicates the steric hindrance during the 1,2-rearrangement. Both 2,4-difluoro- and 2,6-dichloro-substituted thiophenols, as well as 4-bromo-substituted thiophenol, were also well tolerated in the products 5c, 5j and 5k. Heterocyclic thiols were also successfully incorporated in this coupling reaction, directly installing a heteroaryl thioether—a highly versatile linkage widely utilized in the design and optimization of pharmaceuticals (5d–5g).^32^ The product 5d contained a thiophene group, while 5e contained a furan group with a methyl-substituent at the 2-position of the furan ring, which reduced the yield significantly. Remarkably, the transformation successfully incorporated 1,3,4-thiadiazole-2-thiol (5f), a privileged structural motif in medicinal chemistry and a critical building block for potent anticancer, antimicrobial, and anticonvulsant therapeutics.^33–35^ Furthermore, employing bis(4-pyridyl) disulfide under our standard conditions delivered the product 5g in a moderate yield. The aliphatic thiols were also well accommodated in products 5l–5o, which were formed in good to moderate yields. While the secondary thiol, cyclohexanethiol, was well accommodated in this transformation (5o), tertiary thiol or thiolate (2p or 2p′) failed to undergo the reaction, highlighting the steric limits of the 1,2-boronate shift.

**Fig. 3:**
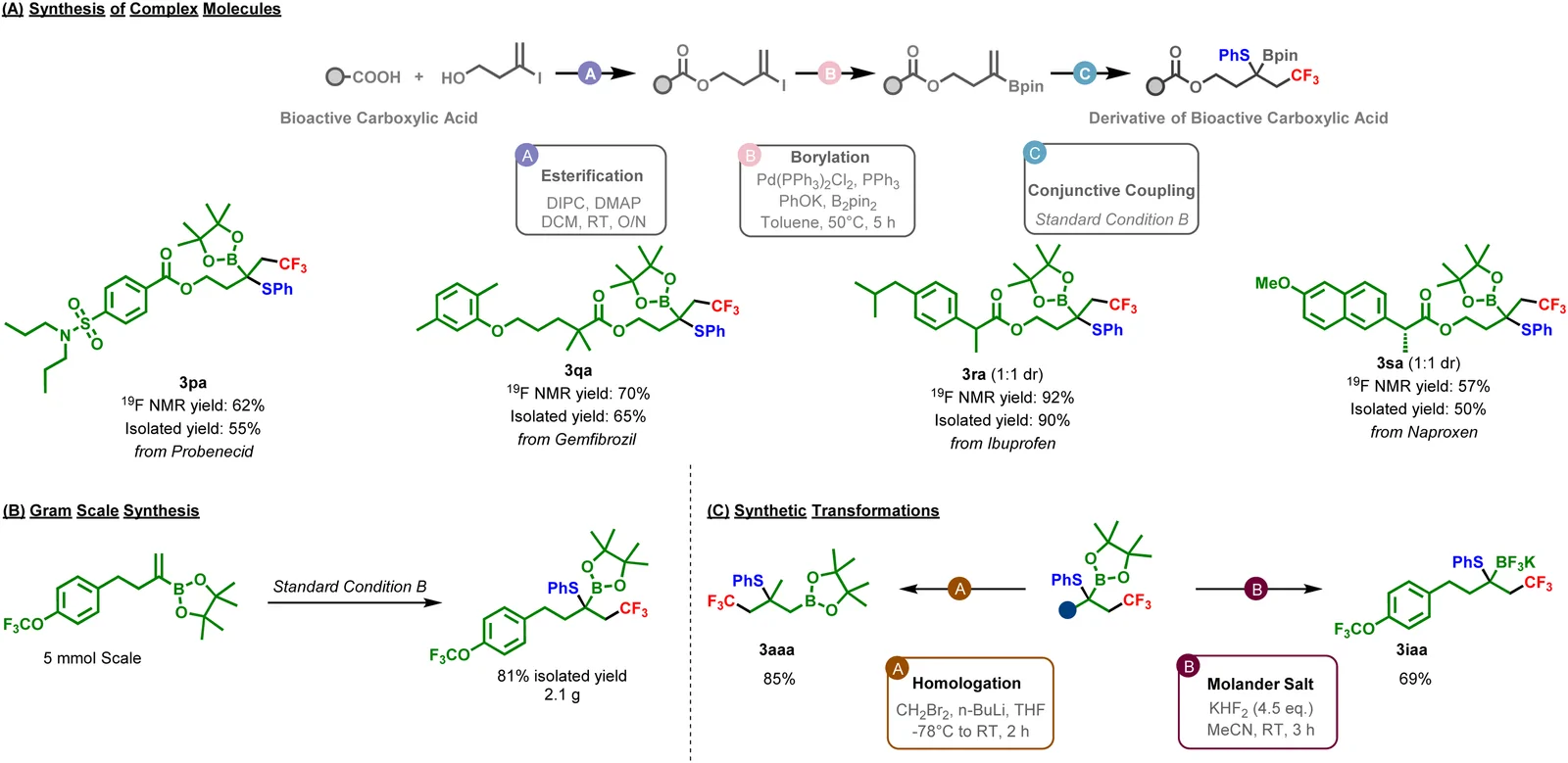
Synthetic utility: (A) late-stage modification of drug molecules; (B) gram-scale synthesis (standard condition B); (C) synthetic transformations of the coupling products 3aa and 3ia. DIPC (*N*,*N*′-diisopropylcarbodiimide); DMAP (4-(dimethylamino)pyridine).

To showcase the synthetic applications of our methodology, we envisioned incorporating drug molecules into this conjunctive coupling reaction. For example, we performed an esterification reaction of carboxylic acid-containing drug molecules with vinyl iodo alcohol, followed by Miyaura borylation to form the corresponding vinyl boronates, which were subjected to our reaction conditions for the synthesis of highly functionalized drug derivatives (Fig. 3A). To our delight, probenecid and gemfibrozil were successfully incorporated into the products 3pa and 3qa, respectively. Notably, the sulfonamide moiety of probenecid and the phenoxide moiety of gemfibrozil were well tolerated along with the ester functional groups, depicting the excellent functional-group tolerance of this transformation. Similarly, the NSAIDs ibuprofen and naproxen were well accommodated in the products 3ra and 3sa, respectively, as 1 : 1 diastereomeric mixtures with good to excellent yields. Next, we performed the scale-up reaction with substrate 1i on a 5 mmol scale, forming 3ia in 81% isolated yield (2.1 g) (Fig. 3B). To demonstrate the synthetic transformations of the conjunctive coupling products, we performed the homologation reaction and successfully introduced one methylene group between the C–B bond of 3aa to form 3aaa, which still contained the Bpin moiety as a functional handle (Fig. 3C). Next, we converted the Bpin moiety of 3ia to the air-stable BF_3_K salt 3iaa. BF_3_K salts are versatile synthetic units that can act as coupling partners in Suzuki–Miyaura coupling reactions^36,37^ as well as various radical reactions.^38,39^

### Mechanistic studies: elimination of the radical pathway

To probe any involvement of radicals in this three-component coupling reaction, several radical trapping experiments were conducted.

**Scheme 1:**
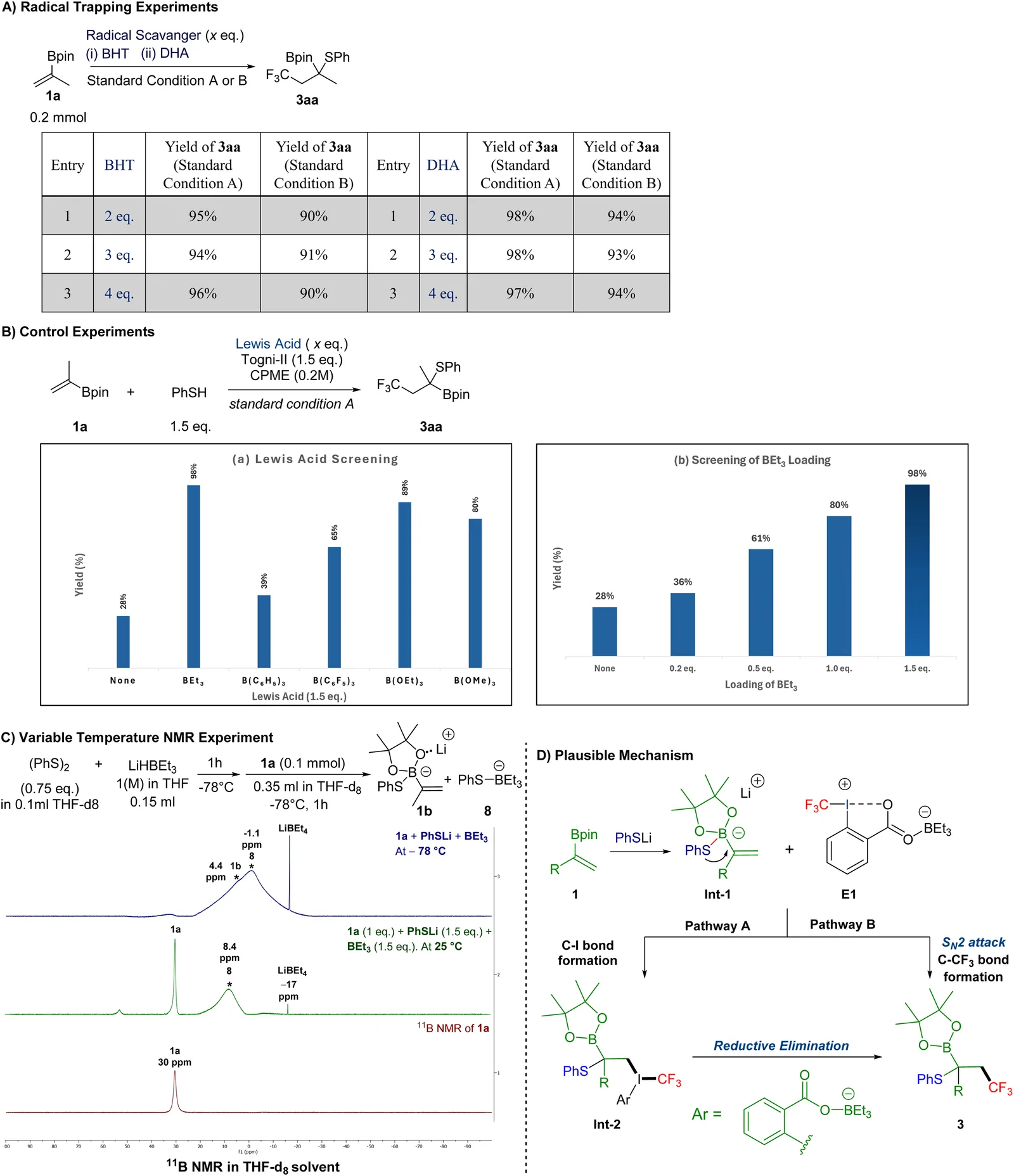
(A) Radical trapping experiments (radical scavengers BHT (butylated hydroxytoluene) and DHA (9,10-dihydroanthracene); (B) control experiments: (a) control experiments involving various Lewis acids; (b) screening of BEt_3_ loading; (C) low temperature ^11^B NMR experiments of the standard reaction; (D) plausible mechanism: pathway A and pathway B.

Since thiols are readily oxidised by TEMPO ((2,2,6,6-tetramethylpiperidin-1-yl)oxyl),^40,41^ it is incompatible as a radical scavenger under our reaction conditions. Thus, we used BHT (butylated hydroxytoluene) as a radical scavenger, and the yield of 3aa remained nearly the same as the optimised yield under both reaction conditions A and B (Scheme 1A) when two equivalents of BHT were added. Upon increasing the loading of BHT up to 4 equivalents, the yield of the product 3aa remained unaffected (Scheme 1A). These results indicate involvement of an ionic mechanism rather than a radical pathway, which was further confirmed by using 9,10-dihydroanthracene (DHA), a known hydrogen donor to trap thiyl radicals (PhS˙),^42^ as the yield of 3aa remained unaltered (Scheme 1A). During the optimisation study, it was observed that without the presence of BEt_3_, the yield of 3aa decreased dramatically, indicating the pivotal importance of BEt_3_ in this transformation (Table 1, entries 7–10). We recognised that BEt_3_ can act as a Lewis acid and activate the Togni-II reagent. Indeed, the Yang group reported similar Lewis acid-promoted activation of Togni-II.^43^ Likewise, we screened other boron-based Lewis acids such as B(OEt)_3_, B(OMe)_3_, BPh_3_, and B(C_6_F_5_)_3_. Using B(OEt)_3_ and B(OMe)_3_ resulted in similar yields of 3aa. While only a 39% yield of 3aa was observed using BPh_3_, switching to the more Lewis acidic B(C_6_F_5_)_3_ increased the yield to 65% (Scheme 1B, a). These results indicate that BEt_3_ might act as a Lewis acid and coordinate to an oxygen atom of the Togni-II reagent, thereby activating it. Next, we screened the loading of BEt_3_, and as the BEt_3_ equivalence was reduced, the yield of 3aa also gradually decreased (Scheme 1B, b). To probe the possibility of the coordination of sulfur-nucleophile to the Bpin moiety of 1a, variable-temperature ^11^B NMR experiments were carried out (Scheme 1C). After reacting the *in situ* prepared PhSLi with 1a for 1 h at −78 °C, the NMR spectrum was recorded at RT, which shows a peak at 30 ppm corresponding to 1a along with a peak at 8.4 ppm indicating the coordination of thiolate nucleophile to BEt_3_. (The peak at −17 ppm corresponds to the LiBEt_4_ impurity present in the commercially available Super-Hydride solution.^44^) Upon lowering the temperature to −78 °C, the ^11^B NMR shows the absence of 1a and formation of a broad peak with a shoulder at 4.4 ppm (1b) and a peak at −1.1 ppm (8), indicating complete coordination of PhSLi to both 1a and BEt_3_ (while LiBEt_4_ is chemically inert, at lower temperatures, the peak at −17 ppm intensifies due to the suppressed ethyl ligand exchange at −78 °C). The gradual upfield shift of the resonance at 8.4 ppm to −1.1 ppm implies a much stronger coordination of PhS in the [PhS-BEt_3_]^−^ adduct 8 at a lower temperature (Scheme 1C and Fig. S1). Crucially, the formation of adduct 8 switches the counteranion of the lithium cation from a highly localised, tightly bound thiolate (in PhSLi) to a large ate complex (the [PhS-BEt_3_]^−^ adduct, 8), where charge is distributed across the system. As demonstrated by Uchiyama and co-workers,^45^ pairing lithium with large, weakly coordinating anions (such as borates or carboranes rather than traditional weakly coordinating anions like ClO^4−^, OTf^−^, *etc.*) generates a highly active, “naked” Li + cation capable of mediating several organic reactions. Similarly, in our work, the efficient coordination of thiolate to BEt_3_ effectively liberates the lithium cation, profoundly enhancing its Lewis acidic character. Driven by its innate oxophilicity, this reactive Li^+^ can effectively coordinate to the pinacol oxygen of the adjacent Bpin moiety of 1a. This coordination increases the electrophilicity of the boron center of the vinyl boronate by lowering the energy of the LUMO (Fig. S6), which in turn facilitates the thiolate binding to the Bpin moiety to form 1b. When the variable temperature NMR experiment was carried out by reacting isolated PhSLi with 1a in the absence of BEt_3_, even at −78 °C, the boronate complex (1b) was not formed (Fig. S3), indicating the pivotal importance of triethylborane.

### Detailed mechanism of conjunctive coupling

A plausible reaction mechanism has been proposed based on all these pieces of experimental evidence. At −78 °C, the sulfur nucleophile coordinates to 1 to form Int-1 (Scheme 1D). The Togni-II reagent was activated by the coordination of BEt_3_ to an oxygen atom to form E1. Next, the conjunctive coupling can happen *via* two different pathways. (1) Reductive elimination pathway (Pathway A): here, the π-system of vinyl boronate interacts with E1, triggering the 1,2-boronate shift to form the C–S bond and C–I bond in a concerted way (Int-2). Consequently, the reductive elimination from Int-2 forms the product 3. (2) S_N_2 pathway (Pathway B): Here, instead of C–I bond formation, the terminal alkene (which becomes nucleophilic due to the 1,2-migration) can directly attack the electrophilic CF_3_ group in an S_N_2 manner to form the product 3. To gain further insight into the mechanism and confirm the reaction pathway, DFT calculations were performed using the Gaussian 16 program suite.^46^ All calculations were carried out at the B3LYP-D3 (ref. 47–49)/def2-SVP^50,51^ level of theory, employing the CPCM^52–55^ solvation model with THF as the solvent at 298 K.

**Fig. 4:**
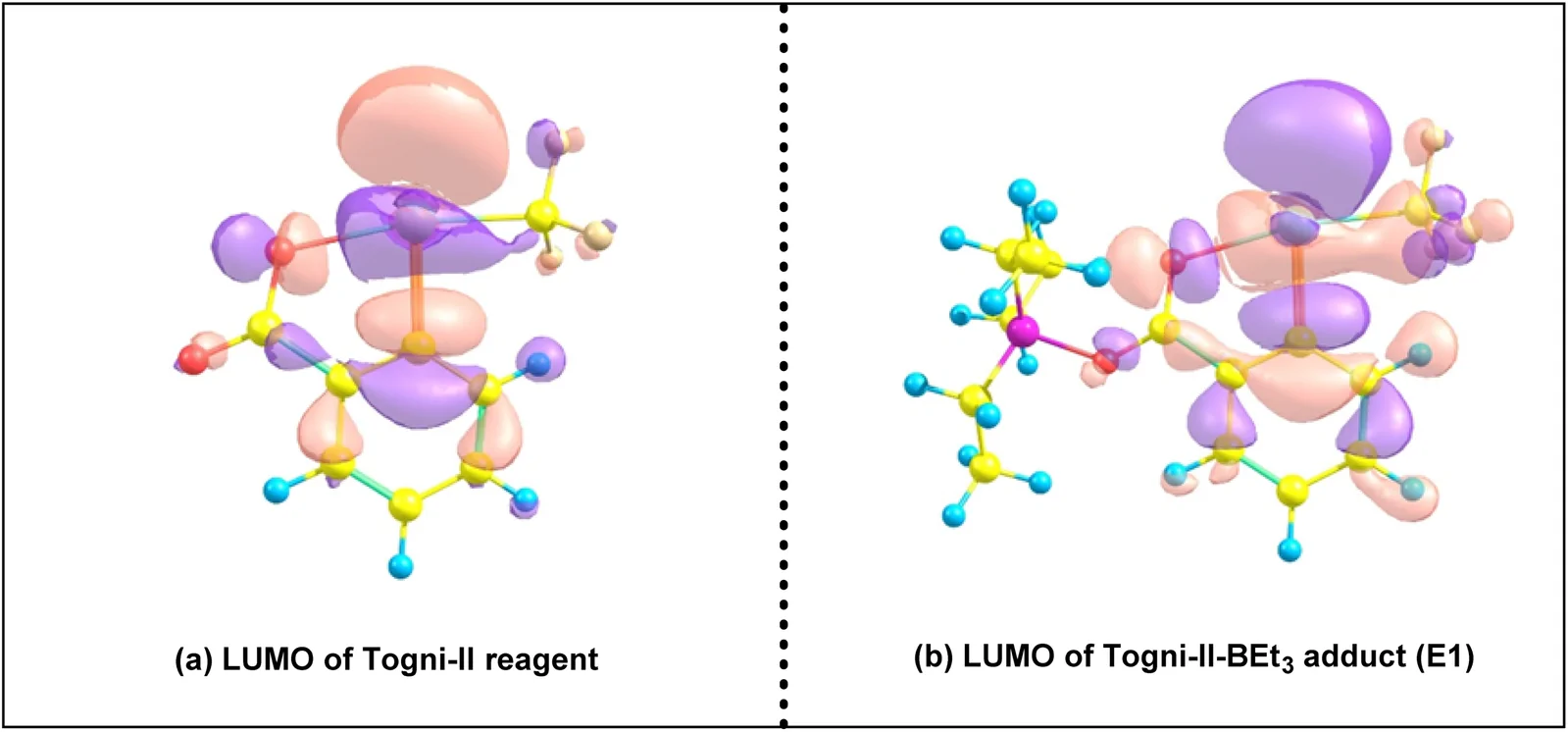
Comparing the LUMO of Togni-II reagent and the Togni-II–BEt_3_ adduct (E1).

To determine the role of BEt_3_ in the electronic activation of the Togni-II reagent, the lowest unoccupied molecular orbitals (LUMOs) of both the free and the Lewis acid-coordinated species were analysed (Fig. 4). For the free Togni-II reagent, the LUMO is primarily localised on the hypervalent iodine center with significant contributions from the antibonding σ*(I–O) orbital. This orbital distribution indicates that the iodine atom serves as the principal electrophilic site, consistent with its well-established role as an electron-accepting center in electrophilic trifluoromethylation reactions. Upon coordination of a Lewis acidic BEt_3_ to the oxygen atom, the electronic structure undergoes a marked change. The oxygen atom donates electron density to the boron center, thereby stabilising the σ*(I–O) orbital. Consequently, the I–CF_3_ bond has a higher orbital coefficient in the LUMO, indicating an enhanced electrophilic character. This interaction effectively increases the CF_3_-transfer ability by weakening the I–CF_3_ bond and facilitating nucleophilic attack or reductive elimination in subsequent coupling steps. Thus, the LUMO analysis clearly demonstrates that the BEt_3_ coordination electronically activates the Togni-II reagent, transforming it into a more potent electrophilic CF_3_ donor.

**Fig. 5:**
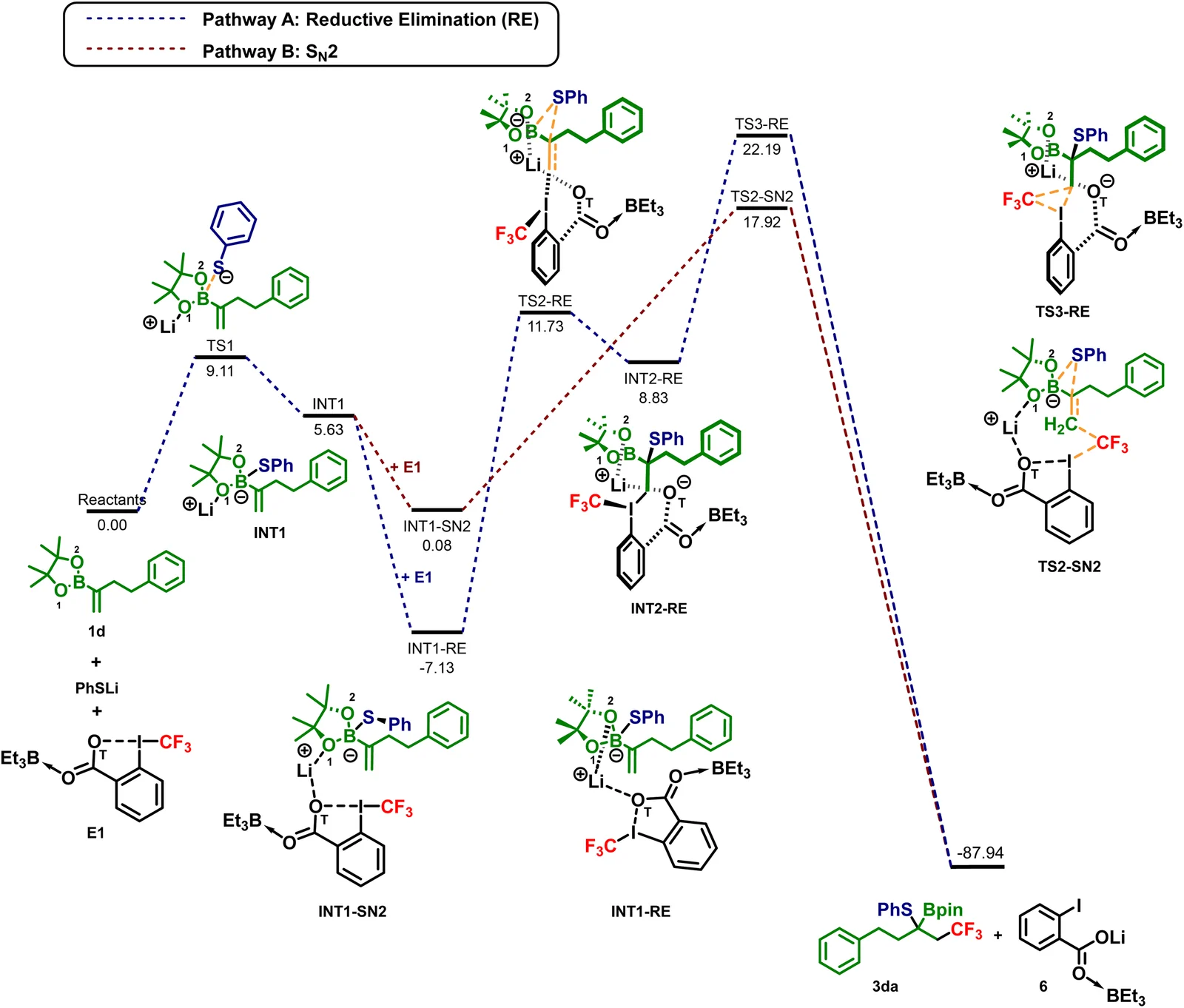
DFT study (B3LYP-D3/def2-SVP, CPCM solvation model for THF at 298 K) of the reaction profile: Reductive elimination (Pathway A in black) and S_N_2 (Pathway B, in red). All free energies (kcal mol^−1^) are relative to the reactants (1d, PhSLi, and Togni–BEt_3_ adduct E1) kept as zero.

Next, the difference in the energetics of the two reaction pathways was studied by DFT computations (B3LYP-D3/def2-SVP, continuum model of solvation CPCM). The vinyl–Bpin species corresponding to product 3da, 4,4,5,5-tetramethyl-2-(4-phenylbut-1-en-2-yl)-1,3,2-dioxaborolane (1d), was chosen as the model substrate, as the crystal structure of 3da was already determined (Fig. S2). The calculated structural parameters of 1d at this level are comparable to those obtained by X-ray analysis. The reaction of PhSLi with 1d leads to the formation of the boron ate complex INT1 (5.63 kcal mol^−1^, above the starting materials) with a free energy barrier of 9.11 kcal mol^−1^*via*TS1 (Fig. 5). In the reductive elimination pathway (Pathway A), as the Togni–BEt_3_ adduct E1 approaches INT1, the orientation of the attack reorganizes such that the Li atom initially coordinated to O1 in INT1 shifts to coordinate with O2 while maintaining a simultaneous coordination with O_T_, affording a stabilized intermediate INT1-RE located at −7.13 kcal mol^−1^ relative to the reactants. This reorganisation minimises steric repulsion and orients the Togni–BEt_3_ adduct nearly perpendicular to the terminal alkene, thereby enabling a favourable approach of the iodine centre. Moreover, the coordination of O_T_ to the Li-atom increases the electrophilicity of E1 even more. From this intermediate, the system must overcome a free-energy barrier of 18.86 kcal mol^−1^ to reach TS2-RE, which leads to INT2-RE, 8.83 kcal mol^−1^ above the reactants. Subsequent reductive elimination occurs through a transition state with a barrier of 13.35 kcal mol^−1^, resulting in highly exergonic products 3da and 6, which lie at −87.94 kcal mol^−1^.

In the S_N_2 pathway (Pathway B), upon interaction with the Togni–BEt_3_ adduct E1, the Li atom coordinates simultaneously with the free oxygen O_T_ of the Togni moiety and with O1 to form INT1-SN2 at 0.08 kcal mol^−1^. As a consequence, a concerted 1,2-migration generates a more nucleophilic terminal alkene, which attacks the electrophilic CF_3_ group through the transition state TS2-SN2 with an activation barrier of 17.85 kcal mol^−1^, leading directly to the conjunctive coupled product.

A comparison of the activation barriers for the rate-determining steps indicates that the S_N_2 pathway is favoured over the reductive elimination pathway, albeit by a small magnitude of 1 kcal mol^−1^. Although the difference between the barriers is relatively small, the S_N_2 pathway proceeds by crossing a single barrier that directly leads to product formation. In contrast, the reductive elimination pathway requires first crossing a barrier of 18.86 kcal mol^−1^, which involves reorganisation of the Togni–BEt_3_ (E1) adduct to form INT2–RE, followed by a subsequent reductive elimination step. Although the overall activation barrier is lower for the S_N_2 pathway, the difference between the relative barriers is not sufficiently large to establish a clear mechanistic preference. Accordingly, both pathways are considered energetically accessible and plausible under the reaction conditions.

## Conclusion

We have developed a practical method of constructing trifluoromethyl-substituted tertiary gem-thioalkyl boronic esters. The reaction proceeds under very mild conditions, with excellent yields, while tolerating a broad range of functional groups and accommodating a wide variety of readily available thiols and disulfides. The synthetic application of this transformation has been showcased by incorporating complex drug molecules into this reaction and by the synthetic transformations of the Bpin moiety present in the product molecules. Both the experimental studies and the DFT analysis revealed the pivotal role of BEt_3_ in the formation of the vinyl boronate complex as well as in the activation of Togni-II reagent. Coordination of the S-nucleophile to the vinyl boronic ester prior to the 1,2-shift has been observed in variable-temperature NMR studies. Although DFT studies (B3LYP-D3/def2-SVP) yield comparable barriers for the S_N_2 and reductive elimination pathways, the overall barrier remains lower for the S_N_2 pathway.

## Author contributions

The manuscript was written through the contributions of all authors. All authors have given approval to the final version of the manuscript.

## Conflicts of interest

There are no conflicts of interest to declare.

## Supplementary Material

SC-017-D6SC04418B-s001

SC-017-D6SC04418B-s002

## Data Availability

The data supporting this article have been included as part of the supplementary information (SI). The authors have cited additional references.^56–65^ Supplementary information: all general methods, experimental procedures, X-ray crystallographic data, NMR spectra, and computational details. See DOI: https://doi.org/10.1039/d6sc04418b. CCDC 2533157 contains the supplementary crystallographic data for this paper.^66^
