# Supplementary material for: Enabling sulfur-centered nucleophiles in conjunctive coupling: triethylborane-mediated trifluoromethylation via vinylboronate complexes
Source: Chem Sci. 2026 Jul 7;17(34):16458–68. doi: 10.1039/d6sc04418b (PMC13377990; doi:10.1039/d6sc04418b)
Supplement: Supplementary file 1 [file SC-017-D6SC04418B-s001.pdf]

Supplementary Information (SI) for Chemical Science.
This journal is © The Royal Society of Chemistry 2026

Enabling Sulfur-Centered Nucleophiles in Conjunctive Coupling: Triethylborane-Mediated Trifluoromethylation via Vinylboronate Complexes

Sufal Paul, Nayanthara T Anilkumar, E. D. Jemmis*, K. Geetharani*

Table of Contents

I	General Information	2
II	General experimental procedure for optimization of reaction conditions	3
III	Preparation of Starting materials	8
IV	General procedure for Three-component Conjunctive Coupling	19
V	Detailed description of products	22
VI	Procedures for Synthetic Transformations	47
VII	Mechanistic Studies	49
VIII	X-Ray Crystallographic Data	55
IX	DFT Calculations	56
X	NMR Spectra	97
XI	References	196

I. General information

Unless otherwise noted, all the reactions are performed in a nitrogen-filled MBraun glove box or using the standard Schlenk technique. All chemicals are purchased either from Sigma Aldrich, TCI chemicals, Alfa Aesar and Avra-chemicals and used without further purification unless mentioned. B_2pin_2 was obtained from AllyChem Co. Ltd., China, and was used after further purification by sublimation process. Reagent-grade solvents are purchased from SD Fine Chemicals (India), distilled, and deoxygenated by freeze pump thaw cycle (three to four times) before use. Togni-II reagent (1-(trifluoromethyl)-1λ^3-benzo[d][1,2]iodaoxol-3(1H)-one) was purchased from Chemscene. $CDCl_3$, Acetone-d_6 and THF-d_8 were purchased from either Cambridge Isotope Laboratories or Sigma Aldrich and deoxygenated by freeze pump thaw cycle and stored over molecular sieves before use. All NMR spectra (^1H (400 MHz / 500 MHz), $^{13}C\{^1H\}$ (100 MHz/ 125MHz), ^{11}B (128 MHz/ 160MHz), were recorded by a Bruker Avance 400 MHz NMR/ 500 MHz spectrometer at an ambient temperature. ^1H NMR chemical shifts are reported relative to TMS and were referenced *via* residual proton resonances of the corresponding deuterated solvent ($CDCl_3$: 7.26 ppm, C_6D_6: 7.16 ppm), whereas ^{13}C NMR spectra are reported relative to TMS using the carbon signals of the deuterated solvent ($CDCl_3$: 77.16 ppm, C_6D_6: 128.0 ppm). ^{11}B NMR signals and ^{19}F NMR signals are quoted relative to $BF_3{\cdot}Et_2O$ and $CFCl_3$ respectively. ^1H NMR yield was calculated as nitromethane or mesitylene (CH_3NO_2 or C_9H_{12}) as an internal standard. GC-MS data were acquired using the GCMS-QP2010 SE SHIMADZU system. For Electrospray ionization (ESI) mass spectral analysis Waters Xevo G3-Q-TOF (LC-HRMS) model mass spectrometers were used. X-ray crystallographic data were collected using Bruker Smart Apex-II ultra-system. Crystal data were refined using Apex 4 software. Commercially available, pre-coated TLC-sheets ALUGRAM® Xtra Sil G/UV_{254} were purchased from MACHEREY-NAGEL GmbH & Co. KG.

II. General experimental procedure for optimization of reaction conditions

Diphenyl disulfide (**2a**) (0.55eq., 0.11 mmol; 24 mg) was dissolved in solvent, then 0.22 mL $LiHBEt_3$ (1(M) in THF) was added at -78 °C; stirred for 40 mins. **1a** (0.2 mmol, 33.6 mg) was dissolved in solvent and added to the reaction at -78 °C and stirred for 1 hr. The resulting reaction mixture was transferred to a solution of Togni-II (1.5 eq., 0.3 mmol, 95 mg) and stirred for 2 hrs at -78 °C. The reaction mixture was allowed to warm up to RT over 2 hrs and stirred at RT for another 8 hrs. After the completion of the reaction, the reaction was diluted with 2 mL ethyl acetate and quenched with 2 mL brine. The layers were separated and the aqueous layer was washed with ethyl acetate (2 mL × 3). The combined organic layer was dried under sodium sulfate, filtered and concentrated under reduced pressure to obtain the crude product. ^{19}F NMR yields are reported using 3-trifluoromethylphenol as the internal standard.

Optimization of reaction condition

Table S1: Screening for Addition Sequence

Entry	Deviation from mentioned Conditions	Yield[d] (**3aa**)	Yield[d] (**3a**, **3b**, **4**)
1	None[a]	37%	nil (**3a**, **3b**); <5% (**4**)
2	Reaction components were added in a simultaneous manner at RT.[b]	nil	23% (**3a**), trace (**3b**), 15% (**4**)
3	Reaction components were added in a simultaneous manner at -78 °C. RT, 8 h.[c]	<5%	16% (**3a**), trace (**3b**), 25% (**4**)

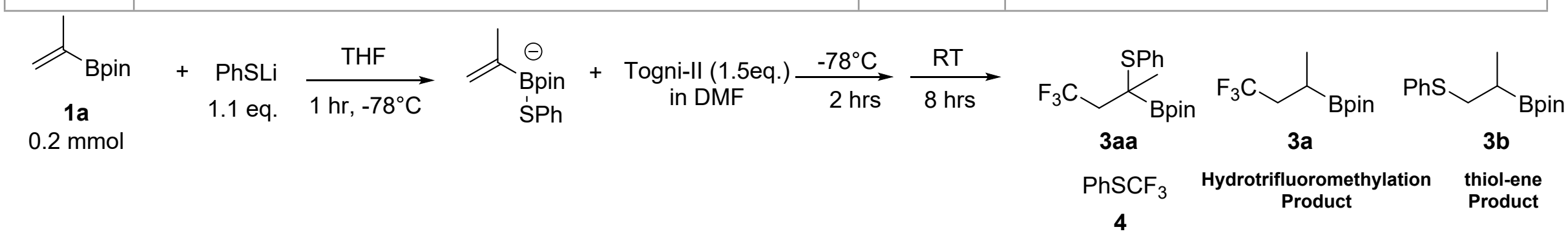

[a] **2a** (0.55eq., 0.11 mmol; 24 mg) was dissolved in 0.2 mL THF, then 0.22 mL $LiHBEt_3$ (1(M) in THF) was added at -78 °C; stirred for 40 mins. **1a** (0.2 mmol, 33.6 mg) in 0.5 mL THF added to the reaction at -78 °C and stirred for 1hr. The resulting reaction mixture was transferred to a 0.2 mL solution of Togni-II (1.5 eq., 0.3 mmol, 95 mg) at -78 °C and stirred for 2hrs at -78°C.and stirred at RT for another 8 hrs. (a) ^{19}F NMR yields are reported using 3-trifluoromethylphenol

as the internal standard. [b] **2a** (0.55eq., 0.11 mmol; 24 mg) in 0.2 ml CPME; 0.3 ml $LiHBEt_3$ (1M solution in THF) added at -78 ºC; stirred for 40 mins. **1a** (0.2 mmol) in 0.5 ml CPME along with Togni-II (0.3 mmol) dissolved in 0.5 ml DMF were added to the reaction at RT. Stirred for 8 h. [c] **2a** (0.55eq., 0.11 mmol; 24 mg) in 0.2 ml CPME; 0.3 ml $LiHBEt_3$ (1M solution in THF) added at -78 ºC; stirred for 40 mins. **1a** (0.2 mmol) in 0.5 ml CPME along with Togni-II (0.3 mmol) dissolved in 0.5 ml DMF were added to the reaction at -78 °C; warmed to RT and stirred for 8 h. [d] ^{19}F NMR, ^{1}H NMR and GC-MS.

Table S2: Solvent Screening 1

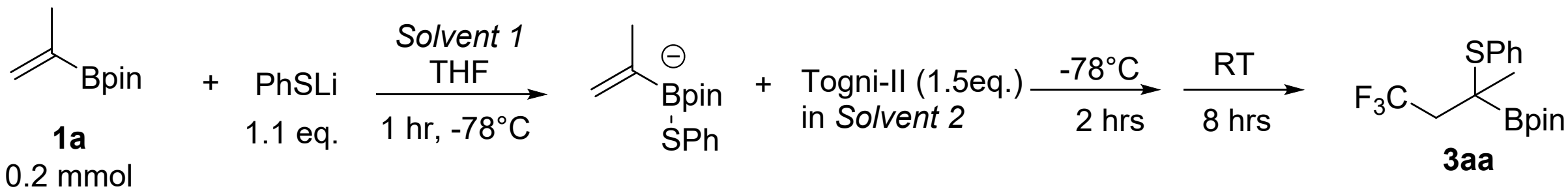

Entry	Solvent 2	Yield[(a)]
1	DMF	37%
2	DMAc	27%
3	MeCN	32%

Diphenyl disulfide (**2a**) (0.55eq., 0.11 mmol; 24 mg) was dissolved in 0.2 mL THF, then 0.22 mL $LiHBEt_3$ (1(M) in THF) was added at -78 °C; stirred for 40 mins. **1a** (0.2 mmol, 33.6 mg) in 0.5 mL THF added to the reaction at -78 °C and stirred for 1 hr. The resulting reaction mixture was transferred to a 0.2 mL solution of Togni-II (1.5 eq., 0.3 mmol, 95 mg) at -78 °C and stirred for 2 hrs at -78°C.and stirred at RT for another 8 hrs. [(a)] ^{19}F NMR yields are reported using 3-trifluoromethylphenol as the internal standard.

Table S3: Solvent Screening 2

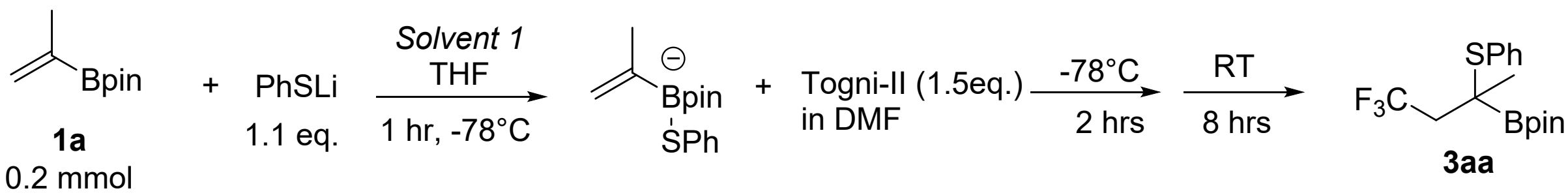

Entry	Solvent 1	Yield[(a)]
1	Toluene	25%
2	THF	37%
3	MTBE	48%
4	CPME	40%

5	DME	30%

Diphenyl disulfide (**2a**) (0.55eq., 0.11 mmol; 24 mg) was dissolved in 0.2 mL THF, then 0.22 mL $LiHBEt_3$ (1(M) in THF) was added at -78 °C; stirred for 40 mins. **1a** (0.2 mmol, 33.6 mg) in 0.5 mL THF added to the reaction at -78 °C and stirred for 1hr. The resulting reaction mixture was transferred to a 0.2 mL DMF solution of Togni-II (1.5 eq., 0.3 mmol, 95 mg) at -78 °C and stirred for 2 hrs at -78°C. and stirred at RT for another 8 hrs. [(a)] ^{19}F NMR yields are reported using 3-trifluoromethylphenol as the internal standard.

Table S4: Solvent Screening 3

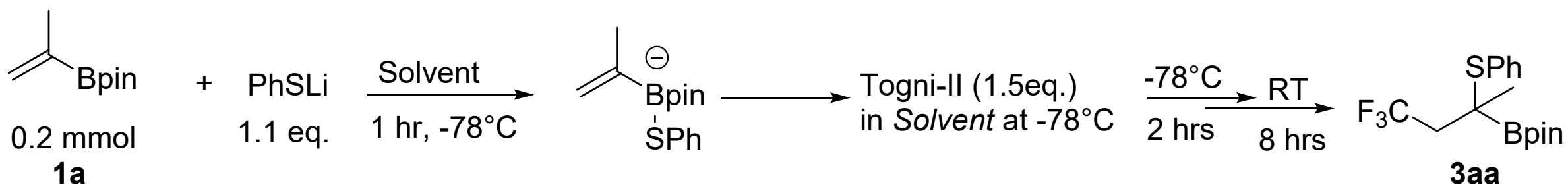

Entry	Solvent	Yield[(a)]
1	MTBE	60%
2	CPME	65%
3	2-Me THF	51%
4	THF	40%

Diphenyl disulfide (**2a**) (0.55 eq., 0.11 mmol; 24 mg) was dissolved in 0.2 mL solvent, then 0.22 mL $LiHBEt_3$ (1(M) in THF) was added at -78 °C; stirred for 40 mins. **1a** (0.2 mmol, 33.6 mg) in 0.5 mL solvent added to the reaction at -78 °C and stirred for 1hr. The resulting reaction mixture was transferred to a 0.2 mL solution of Togni-II (1.5 eq., 0.3 mmol, 95 mg) at -78 °C and stirred for 2 hrs at -78 °C. and stirred at RT for another 8 hrs. [(a)] ^{19}F NMR yields are reported using 3-trifluoromethylphenol as the internal standard.

Table S5: Concentration Screening

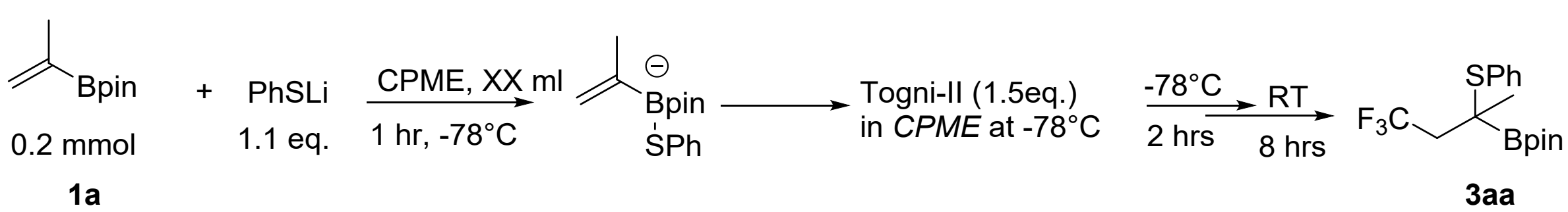

Entry	Total volume XX ml	Conc.	Yield[(a)]
1	2 ml	0.1 (M)	65%
2	1.3 ml	0.15 (M)	75%

3	1 ml	0.2 (M)	78%

Diphenyl disulfide (**2a**) (0.55eq., 0.11 mmol; 24 mg) was dissolved in CPME solvent, then 0.22 mL $LiHBEt_3$ (1(M) in THF) was added at -78 °C; stirred for 40 mins. **1a** (0.2 mmol, 33.6 mg) in CPME solvent added to the reaction at -78 °C and stirred for 1 hr. The resulting reaction mixture was transferred to a CPME solution of Togni-II (1.5 eq., 0.3 mmol, 95 mg) at -78 °C and stirred for 2hrs at -78 °C. and stirred at RT for another 8 hrs. [(a)] ^{19}F NMR yields are reported using 3-trifluoromethylphenol as the internal standard.

Table S6: Equivalence Screening

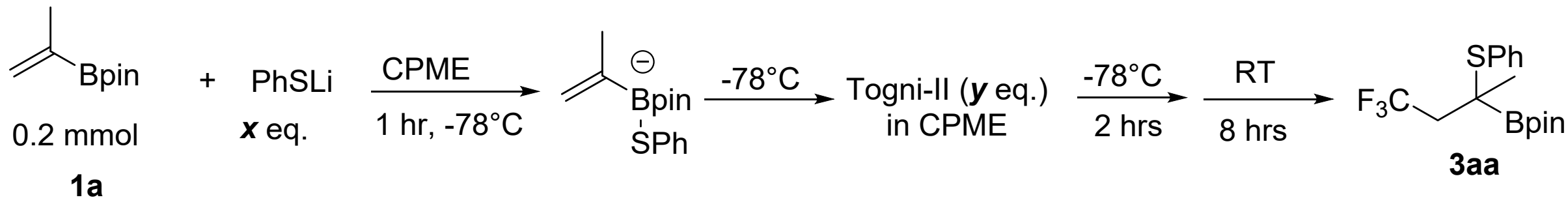

Entry	PhSLi (x eq.)	Togni-II (y eq.)	Total Volume ml	Yield[(a)]
1	1.5 eq.	1.5 eq.	1.2 ml	94%
2	1.5 eq.	2 eq.	1.2 ml	81%

Diphenyl disulfide (**2a**) (0.75 eq., 0.15 mmol; 32.7 mg) was dissolved in CPME solvent, then 0.3 mL $LiHBEt_3$ (1(M) in THF) was added at -78 °C; stirred for 40 mins. **1a** (0.2 mmol, 33.6 mg) in CPME solvent added to the reaction at -78 °C and stirred for 1 hr. The resulting reaction mixture was transferred to a CPME solution of Togni-II (y eq.) at -78 °C and stirred for 2 hrs at -78°C. and stirred at RT for another 8 hrs. [(a)] ^{19}F NMR yields are reported using 3-trifluoromethylphenol as the internal standard.

Table S7: Temperature Screening

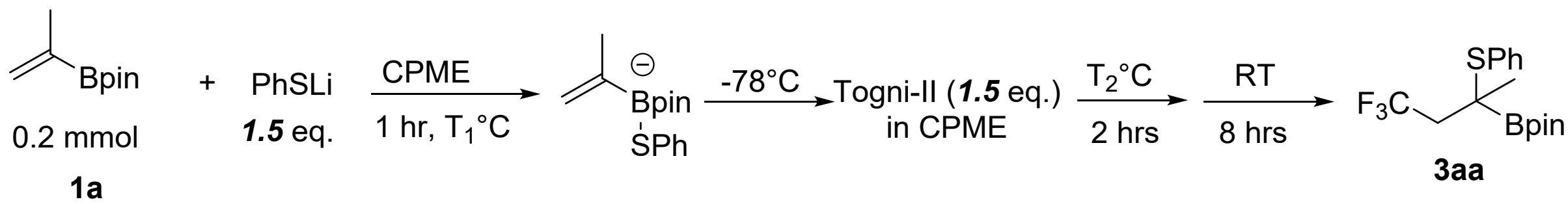

Entry	T1 (°C)	T2 (°C)	Yield[(a)]
1	-78	RT[(b)]	75%
2	0	0	25%
3	-78	-78 to RT[(c)]	94%

4	-30	-30 to RT	40%
5	RT	RT	10%

Diphenyl disulfide (**2a**) (0.75 eq., 0.15 mmol; 32.7 mg) was dissolved in CPME solvent, then 0.3 mL $LiHBEt_3$ (1(M) in THF) was added at -78 °C; stirred for 40 mins. **1a** (0.2 mmol, 33.6 mg) in CPME solvent added to the reaction at T1 °C and stirred for 1 hr. The resulting reaction mixture was transferred to a CPME solution of Togni-II (1.5 eq., 0.3 mmol, 95 mg) at T2 °C and stirred for 2hrs at -78 °C. Stirred at RT for 8 hrs. [(a)] ^{19}F NMR yields are reported using 3-trifluoromethylphenol as the internal standard. [(b)]after adding Togni-ii reagent at -78 °C, the reaction was immediately warmed to RT. (c) after adding Togni-II reagent at -78 °C, the reaction was stirred at -78 °C for 2 hrs, then warmed to RT over another 2 hrs, followed by stirring at RT for 8 hrs.

Table S8: Lewis Acid Screening

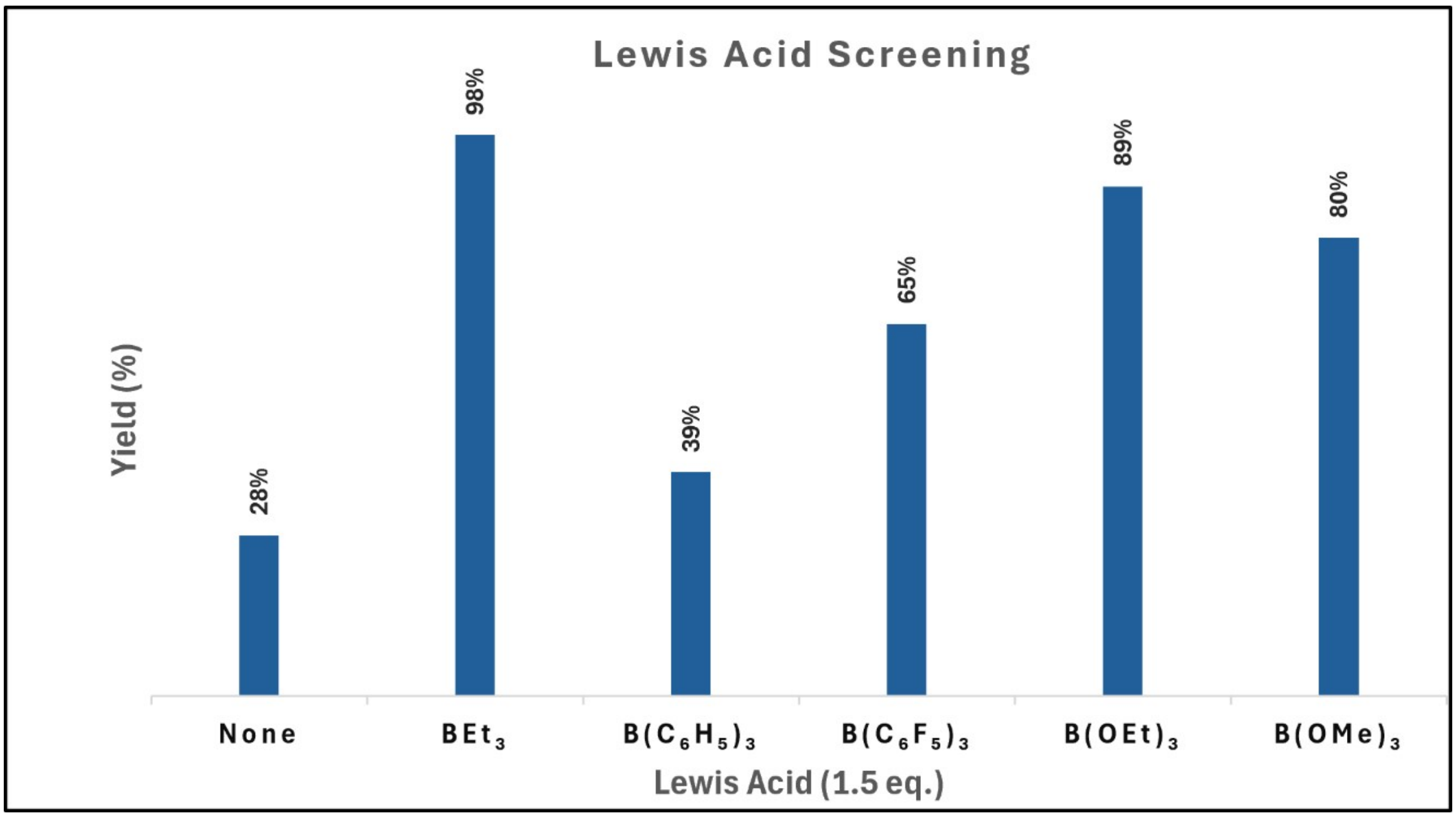

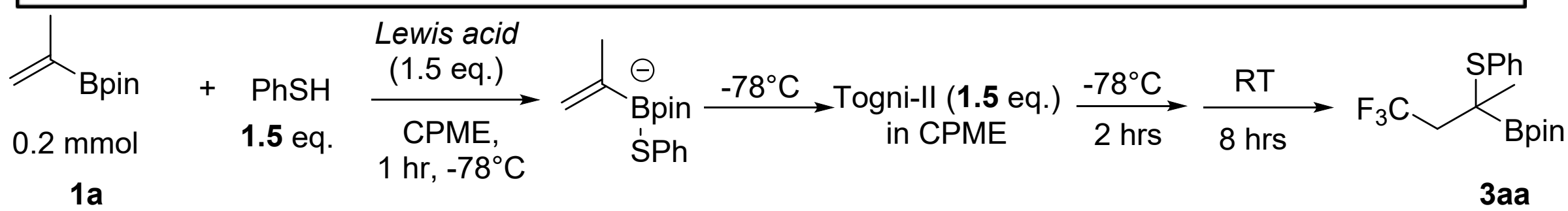

PhSH (0.3 mmol, 33 mg) in 0.2 ml CPME; 0.3 ml *Lewis Acid* (1M solution in THF) added at -78 ºC; stirred for 40 mins; **1a** (0.2 mmol, 33.6 mg) in 0.5 ml CPME added to the reaction at -78 ºC; Stirred for 1 h; Reaction mixture transferred to a 0.2 ml CPME solution of Togni-II (0.3 mmol, 95 mg) at -78 ºC; Stirred for 2 h at -78 ºC and stirred at RT for another 8 h. * While BPh_3 was completely soluble in THF or CPME at room temperature, the mixture formed a suspension at lower temperatures (–78 °C).

Table S9: Control Experiments

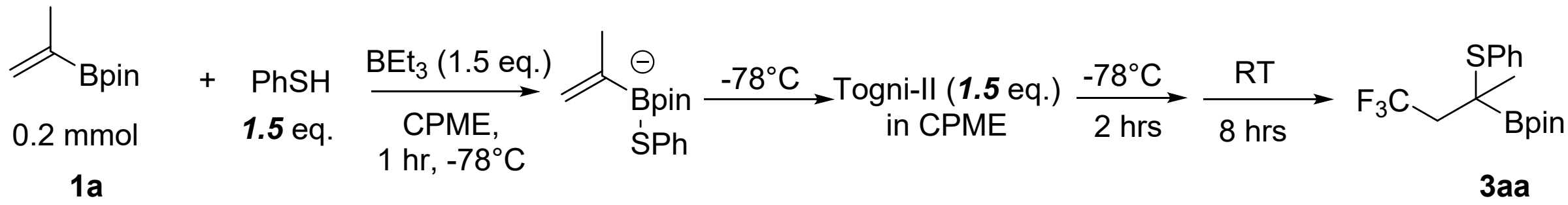

Entry	Reaction Conditions	Yield[(a)]
1	PhSH instead of PhSLi along with 1.5 eq. BEt_3 (1M in THF)	98%
2	Isolated PhSLi instead of [diphenyl disulfide + $LiHBEt_3$]	25%
3	PhSH without BEt_3	28%
4	PhSH instead of PhSLi along with 0.2 eq. BEt_3 (1M in THF)	36%
5	Togni-I instead of Togni-II as the hypervalent iodine(III) reagent[b]	77%
6	Togni-I instead of Togni-II as the hypervalent iodine(III) reagent[c]	73%

Thiophenol (1.5 eq., 0.3 mmol; 33 mg) was dissolved in CPME solvent, then 0.3 mL BEt_3 (1(M) in THF) was added at -78 °C; stirred for 40 mins. **1a** (0.2 mmol, 33.6 mg) in CPME solvent added to the reaction at -78 °C and stirred for 1hr. The resulting reaction mixture was transferred to a CPME solution of Togni-II (1.5 eq., 0.3 mmol, 95 mg) at -78 °C and stirred for 2 hrs at -78 °C and stirred at RT for another 8 hrs. [a] ^{19}F NMR yields are reported using 3-trifluoromethylphenol as the internal standard. [b] Thiophenol (1.5 eq., 0.3 mmol; 33 mg) was dissolved in CPME solvent, then 0.3 mL BEt_3 (1(M) in THF) was added at -78 °C; stirred for 40 mins. **1a** (0.2 mmol, 33.6 mg) in CPME solvent added to the reaction at -78 °C and stirred for 1hr. The resulting reaction mixture was transferred to a CPME solution of Togni-I [3,3-Dimethyl-1-(trifluoromethyl)-1,2-benziodoxole] (1.5 eq., 0.3 mmol, 99.03 mg) at -78 °C and stirred for 2 hrs at -78 °C and stirred at RT for another 8 hrs. [c] Diphenyl disulfide (**2a**) (0.15 mmol, 0.75 eq.) in 0.2 ml CPME; 0.3 ml $LiHBEt_3$ (1.5 eq., 1M solution in THF) added at -78 ºC; stirred for 40 mins; **1a** (0.2 mmol, 1 eq.) in 0.5 ml CPME added to the reaction at -78 ºC; Stirred for 1 h; Reaction mixture transferred to a 0.2 ml CPME solution of Togni-I (99.03 mg, 0.3 mmol, 1.5 eq.) at -78 ºC; Stirred for 2 h at -78 ºC and stirred at RT for another 8 h.

III. Preparation of Starting materials

Compound **1a** and **1b** are commercially available and purchased from BLDpharm.

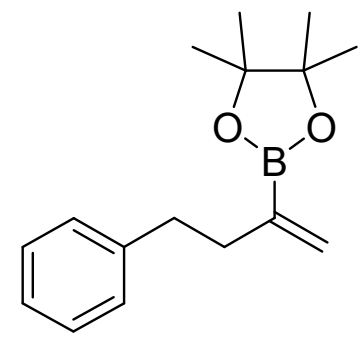

1d

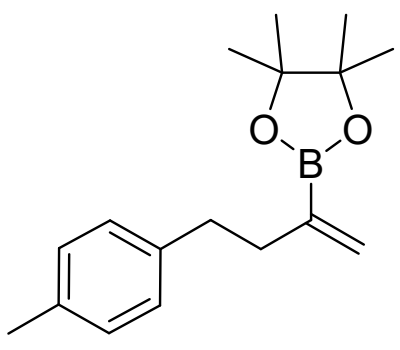

1e

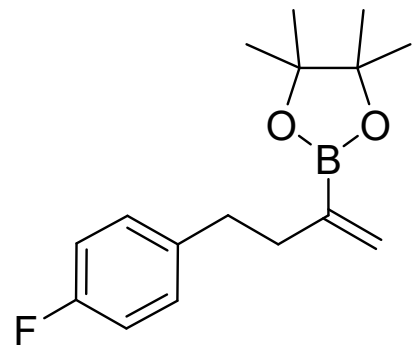

1f

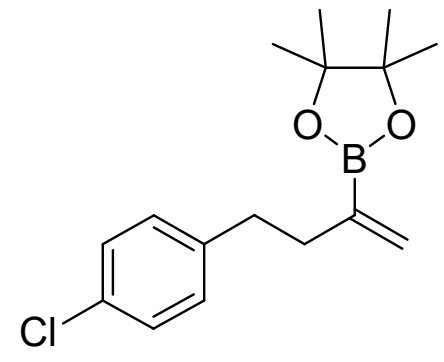

1g

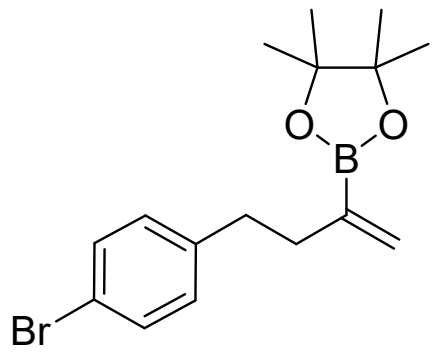

1h

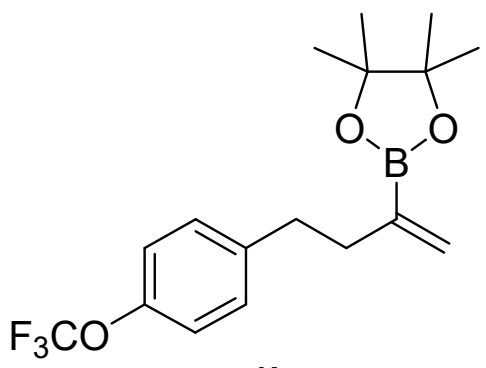

1i

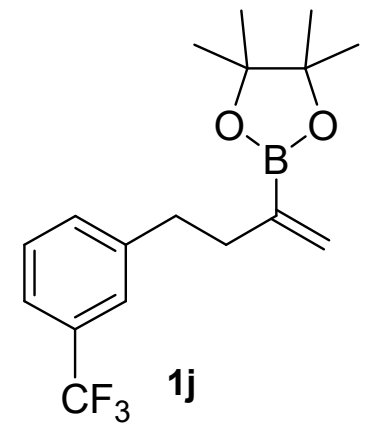

1j

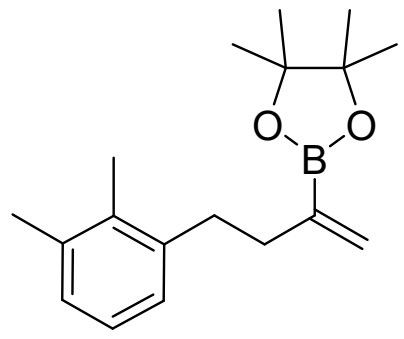

1k

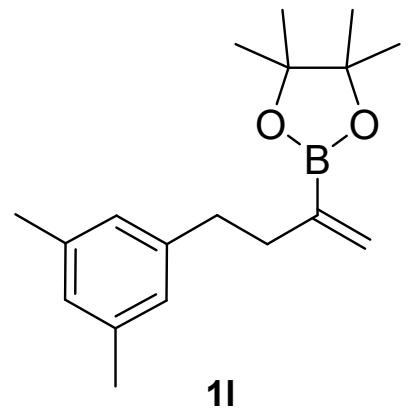

1l

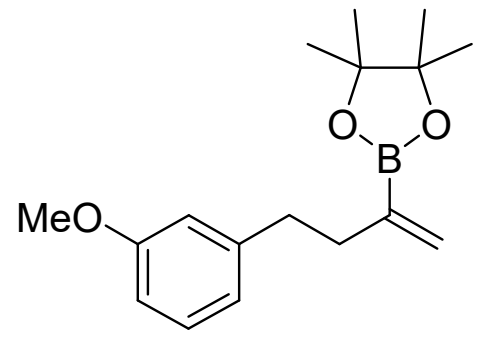

1m

Compounds **1d** – **1m** are prepared following the general procedure 1A.

<u>General Procedure for Starting Material Preparation 1A</u>

<u>Step 1 – Preparation of Allenes[(1)]</u>

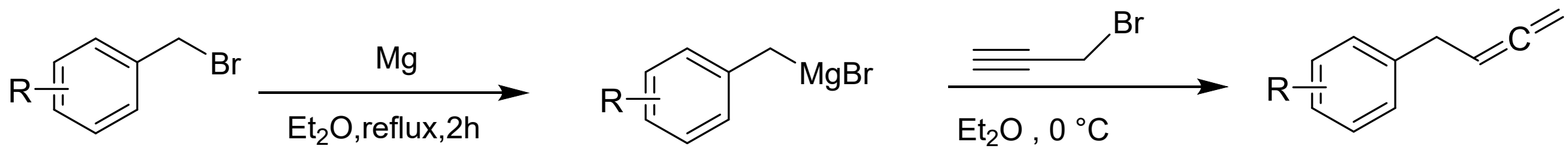

The allenes are prepared following literature procedure. To a 100-mL round-bottomed flask containing dry magnesium turnings (2.9 g, 0.12 mol, 1.2 equiv), diethyl ether (40 mL) and 1,2-Dibromoethane (0.5 mL) were added under N_2. Then benzyl bromide (0.1 mol) was added to the reaction mixture slowly. After the addition, the mixture is heated under reflux for 2 h. Another 250-mL round-bottomed flask containing propargyl bromide (17.8 mL of an 80 wt. % solution in toluene, 0.12 mol, 1.2 equiv) and diethyl ether (20 mL) was cooled to 0 °C. The prepared Grignard reagent was added to the propargyl bromide slowly. After the addition, the resulting mixture is stirred for an additional 2 h at 0 °C. Then the mixture is quenched with aqueous ammonium chloride solution. The aqueous phase is extracted with diethyl ether. The combined organic phases were dried over anhydrous $MgSO_4$ and concentrated by rotary evaporation. Purification is performed by silica gel flash coloumn chromatography to afford corresponding allene.

<u>Step 2 – Hydroboration of allenes[(2)]</u>

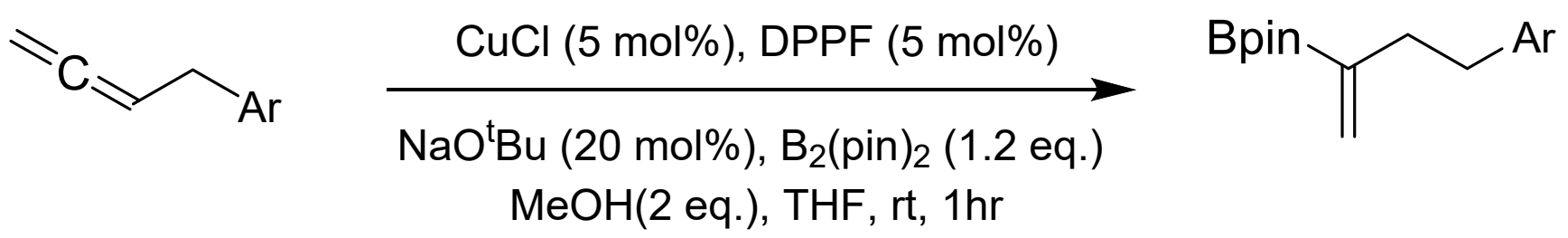

The hydroboration of allenes is carried out following literature procedure.(2) To an oven-dried 50 ml round bottom Schlenck flask equipped with a magnetic stirring bar, bis(pinacolato)diboron (1.2 eq.), dppf (5 mol%), CuCl (5 mol%), NaO^{t}Bu (20 mol%), allene (1 eq.), MeOH (2 eq.) and THF solvent were added followed by stirring for 1 hour at room temperature. The crude reaction mixture was passed through a short silica gel plug and dried followed by purification using flash column chromatography to afford the corresponding vinyl boronate.

4,4,5,5-tetramethyl-2-(4-phenylbut-1-en-2-yl)-1,3,2-dioxaborolane (1d)

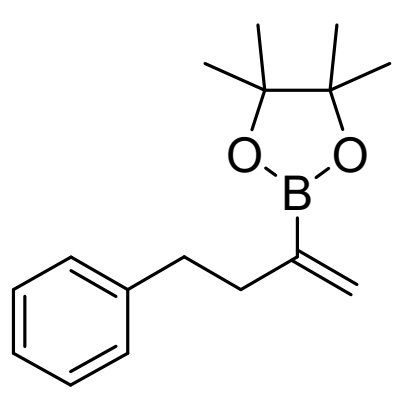

1d

1d was prepared following General Procedure for Starting Material Preparation 1A in 20 mmol scale. The desired product **1d** (2.79g; Overall yield: 54%) was isolated by flash column chromatography using hexane : diethyl ether (98:2) as a colourless oil.

^{1}H NMR (500 MHz, Chloroform-*d*) δ 7.22 – 7.16 (m, 2H), 7.14 – 7.06 (m, 3H), 5.72 (d, J = 3.4 Hz, 1H), 5.53 (br, 1H), 2.71 – 2.62 (m, 2H), 2.44 – 2.34 (m, 2H), 1.20 (s, 12H).

^{13}C NMR (125 MHz, $CDCl_3$) δ 142.4, 129.4, 128.6, 128.2, 125.6, 83.4, 37.3, 35.7, 24.8. The carbon attached to the boron was not found due to quadrupolar relaxation.

^{11}B NMR (160 MHz, $CDCl_3$) δ 30.2

4,4,5,5-tetramethyl-2-(4-(p-tolyl)but-1-en-2-yl)-1,3,2-dioxaborolane (1e)

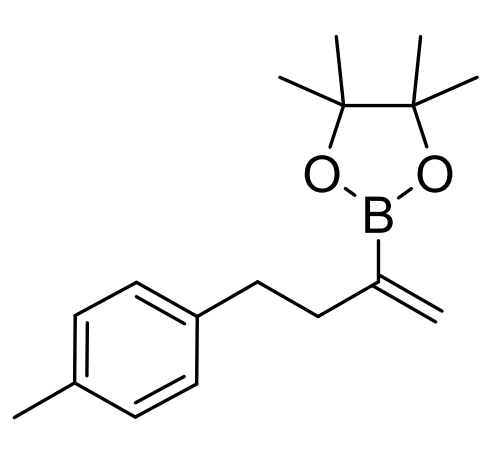

1e

1e was prepared following General Procedure for Starting Material Preparation 1A in 20 mmol scale. The desired product **1e** (3.23 g; Overall yield: 60%) was isolated by flash column chromatography using hexane : diethyl ether (98:2) as a colourless oil.

^{1}H NMR (500 MHz, Chloroform-*d*) δ 7.09 (s, 4H), 5.80 (d, *J* = 3.1 Hz, 1H), 5.62 (br, 1H), 2.79 – 2.55 (m, 2H), 2.55 – 2.40 (m, 2H), 2.32 (s, 3H), 1.28 (s, 12H).

^{13}C NMR (125 MHz, Chloroform-*d*) δ 139.4, 134.9, 129.4, 128.9, 128.4, 83.4, 37.5, 35.3, 24.8, 21.0. The carbon attached to the boron was not found due to quadrupolar relaxation.

^{11}B NMR (160 MHz, Chloroform-*d*) δ 30.1.

2-(4-(4-fluorophenyl)but-1-en-2-yl)-4,4,5,5-tetramethyl-1,3,2-dioxaborolane (1f)

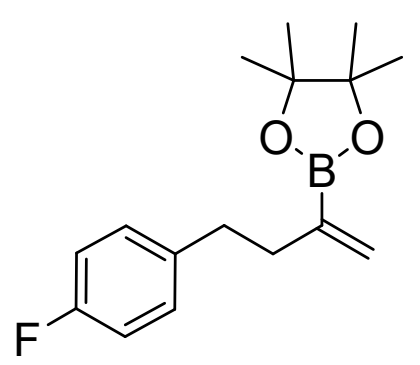

1f

1f was prepared following General Procedure for Starting Material Preparation 1A in 20 mmol scale. The desired product **1f** (2.82 g; Overall yield: 51%) was isolated by flash column chromatography using hexane : diethyl ether (98:2) as a colourless oil.

^{1}H NMR (500 MHz, Chloroform-*d*) δ 7.13 (dd, *J* = 8.3, 5.6 Hz, 2H), 6.94 (t, *J* = 8.7 Hz, 2H), 5.79 (d, *J* = 3.0 Hz, 1H), 5.58 (br, 1H), 2.77 – 2.66 (m, 2H), 2.48 – 2.36 (m, 2H), 1.27 (s, 12H).

^{13}C NMR (125 MHz, Chloroform-*d*) δ 161.2 (d, *J* = 243 Hz), 137.9 (d, *J* = 2.8Hz)

129.9 (d, *J* = 75 Hz), 114.8 (d, *J* = 114.8Hz), 83.4, 37.3, 34.9, 24.8. The carbon attached to the boron was not found due to quadrupolar relaxation.

^{11}B NMR (160 MHz, Chloroform-*d*) δ 30.1.

^{19}F NMR (470 MHz, Chloroform-*d*) δ -118.25 (m).

2-(4-(4-chlorophenyl)but-1-en-2-yl)-4,4,5,5-tetramethyl-1,3,2-dioxaborolane (1g)

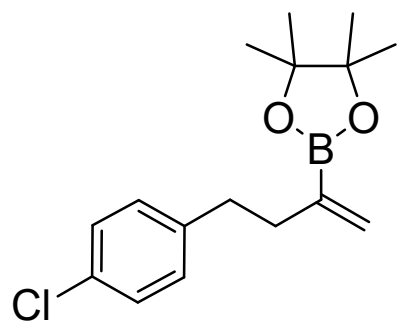

1g

1g was prepared following General Procedure for Starting Material Preparation 1A in 20 mmol scale. The desired product **1g** (3.5 g; Overall yield: 59%) was isolated by flash column chromatography using hexane : diethyl ether (98:2) as a colourless oil.

^{1}H NMR (500 MHz, Chloroform-*d*) δ 7.22 (d, *J* = 8.2 Hz, 2H), 7.11 (d, *J* = 8.2 Hz, 2H), 5.79 (d, *J* = 3.3 Hz, 1H), 5.58 (br, 1H), 2.76 – 2.61 (m, 2H), 2.49 – 2.38 (m, 2H), 1.26 (s, 12H).

^{13}C NMR (125 MHz, Chloroform-*d*) δ 140.8, 131.3, 129.9, 129.9, 128.2, 83.4, 37.1, 35.1, 24.8. The carbon attached to the boron was not found due to quadrupolar relaxation.

^{11}B NMR (160 MHz, Chloroform-*d*) δ 29.9.

2-(4-(4-bromophenyl)but-1-en-2-yl)-4,4,5,5-tetramethyl-1,3,2-dioxaborolane (1h)

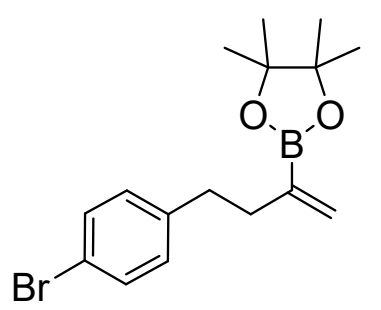

1h

1h was prepared following General Procedure for Starting Material Preparation 1A in 20 mmol scale. The desired product **1h** (3.51 g; Overall yield: 52%) was isolated by flash column chromatography using hexane : diethyl ether (98:2) as a white solid.

^{1}H NMR (500 MHz, Chloroform-*d*) δ 7.37 (d, *J* = 8.0 Hz, 2H), 7.05 (d, *J* = 8.0 Hz, 2H), 5.79 (d, *J* = 3.4 Hz, 1H), 5.58 (br, 1H), 2.76 – 2.60 (m, 2H), 2.48 – 2.29 (m, 2H), 1.26 (s, 12H).

^{13}C NMR (125 MHz, Chloroform-*d*) δ 141.3, 131.1, 130.4, 129.9, 119.3, 83.4, 37.1, 35.1, 24.7 The carbon attached to the boron was not found due to quadrupolar relaxation.

^{11}B NMR (160 MHz, Chloroform-*d*) δ 30.0.

4,4,5,5-tetramethyl-2-(4-(4-(trifluoromethoxy)phenyl)but-1-en-2-yl)-1,3,2-dioxaborolane 1i)

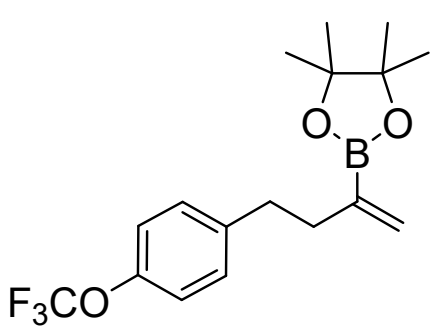

1i

1i was prepared following General Procedure for Starting Material Preparation 1A in 20 mmol scale. The desired product **1i** (3.4 g; Overall yield: 50%) was isolated by flash column chromatography using hexane : diethyl ether (98:2) as a colourless oil.

¹H NMR (500 MHz, Chloroform-*d*) δ 7.19 (d, *J* = 8.4 Hz, 2H), 7.10 (d, *J* = 8.3 Hz, 2H), 5.81 (d, *J* = 3.3 Hz, 1H), 5.60 (br, 1H), 2.76 – 2.73 (m, 2H), 2.46 – 2.43 (m, 2H), 1.26 (s, 12H).

¹³C NMR (125 MHz, Chloroform-*d*) δ 147.3, 141.1, 129.8, 120.8, 120.5 (q, *J* = 256.4 Hz), 83.5, 37.0, 35.1, 24.8. The carbon attached to the boron was not found due to quadrupolar relaxation.

¹¹B NMR (160 MHz, Chloroform-*d*) δ 30.1.

¹⁹F NMR (470 MHz, Chloroform-*d*) δ -57.9.

4,4,5,5-tetramethyl-2-(4-(3-(trifluoromethyl)phenyl)but-1-en-2-yl)-1,3,2-dioxaborolane (1j)

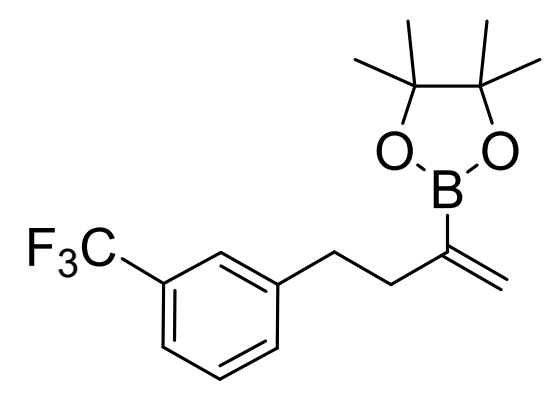

1j

1j was prepared following General Procedure for Starting Material Preparation 1A in 20 mmol scale. The desired product **1j** (3.46 g; Overall yield: 53%) was isolated by flash column chromatography using hexane : diethyl ether (98:2) as a colourless oil.

¹H NMR (500 MHz, Chloroform-*d*) δ 7.44 – 7.36 (m, 4H), 5.81 (d, *J* = 3.2 Hz, 1H), 5.60 (br, 1H), 2.88 – 2.71 (m, 2H), 2.56 – 2.34 (m, 2H), 1.27 (s, 12H).

¹³C NMR (125 MHz, Chloroform-*d*) δ 143.2, 132.0, 130.0, 128.5, δ 125.3 (q, *J* = 3.8 Hz), 124.4 (q, *J* = 272.2 Hz), 122.5 (q, *J* = 3.9 Hz), 122.4, 83.5, 36.9, 35.5, 24.8. The carbon attached to the boron was not found due to quadrupolar relaxation.

¹¹B NMR (160 MHz, Chloroform-*d*) δ 30.1.

¹⁹F NMR (470 MHz, Chloroform-*d*) δ -62.51.

2-(4-(2,3-dimethylphenyl)but-1-en-2-yl)-4,4,5,5-tetramethyl-1,3,2-dioxaborolane (1k)

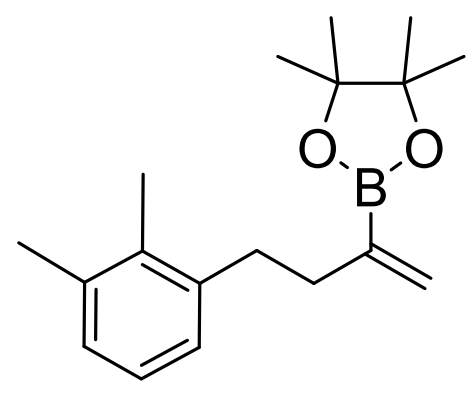

1k

1k was prepared following General Procedure for Starting Material Preparation 1A in 20 mmol scale. The desired product **1k** (3.32 g; Overall yield: 58%) was isolated by flash column chromatography using hexane : diethyl ether (98:2) as a colourless oil.

^{1}H NMR (500 MHz, Chloroform-*d*) δ 7.03 – 0.99 (m, 3H), 5.81 (d, *J* = 3.4 Hz, 1H), 5.66 (br, 1H), 2.82 – 2.66 (m, 2H), 2.47 – 2.34 (m, 2H), 2.29 (s, 3H), 2.24 (s, 3H), 1.29 (s, 12H).

^{13}C NMR (125 MHz, Chloroform-*d*) δ 140.6, 136.7, 134.6, 129.2, 127.6, 127.1, 125.2, 83.4, 36.8, 34.2, 24.8, 20.8, 14.9. The carbon attached to the boron was not found due to quadrupolar relaxation.

^{11}B NMR (160 MHz, Chloroform-*d*) δ 30.3.

2-(4-(3,5-dimethylphenyl)but-1-en-2-yl)-4,4,5,5-tetramethyl-1,3,2-dioxaborolane (1l)

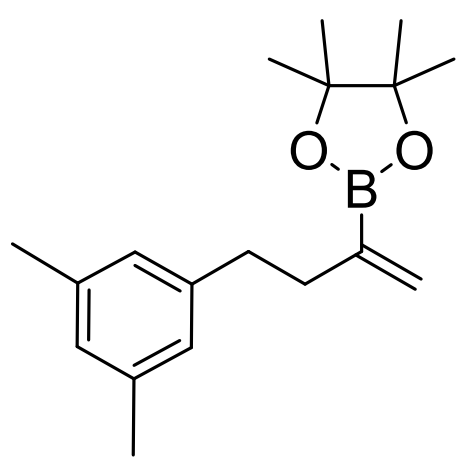

1l

1l was prepared following General Procedure for Starting Material Preparation 1A in 20 mmol scale. The desired product **1l** (3.21 g; Overall yield: 56%) was isolated by flash column chromatography using hexane : diethyl ether (98:2) as a colourless oil.

^{1}H NMR (500 MHz, Chloroform-*d*) δ 6.82 (d, *J* = 7.6 Hz, 3H), 5.80 (d, *J* = 3.5 Hz, 1H), 5.64 (br, 1H), 2.74 – 2.52 (m, 2H), 2.52 – 2.35 (m, 2H), 2.29 (s, 6H), 1.28 (s, 12H).

^{13}C NMR (125 MHz, Chloroform-*d*) δ 142.4, 137.6, 129.2, 127.3, 126.4, 83.4, 37.3, 35.6, 24.8, 21.3. The carbon attached to the boron was not found due to quadrupolar relaxation.

^{11}B NMR (160 MHz, Chloroform-*d*) δ 30.2.

2-(4-(3-methoxyphenyl)but-1-en-2-yl)-4,4,5,5-tetramethyl-1,3,2-dioxaborolane (1m)

1m was prepared following General Procedure for Starting Material Preparation 1A in 20 mmol scale. The desired product **1m** (3.29 g; Overall yield: 57%) was isolated by flash column chromatography using hexane : diethyl ether (95:5) as a colourless oil.

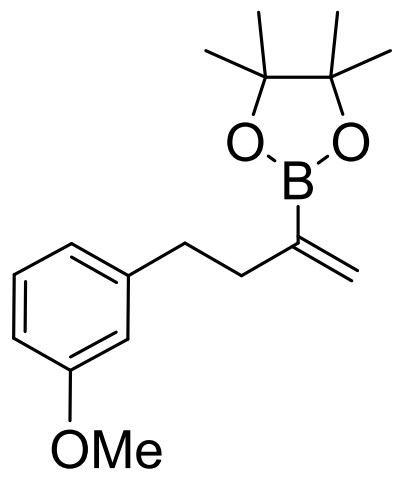

1m

^{1}H NMR (500 MHz, Chloroform-*d*): δ 7.18 (t, *J* = 7.8 Hz, 1H), 6.87 – 6.55 (m, 3H), 5.79 (d, *J* = 3.3 Hz, 1H), 5.61 (br, 1H), 3.80 (s, 3H), 2.81 – 2.57 (m, 2H), 2.52 – 2.31 (m, 2H), 1.27 (s, 12H).

^{13}C NMR (125 MHz, Chloroform-*d*): δ 159.5, 144.1, 129.4, 129.1, 121.0, 114.3, 110.9, 83.4, 55.1, 37.2, 35.8, 24.8. The carbon attached to the boron was not found due to quadrupolar relaxation.

^{11}B NMR (160 MHz, Chloroform-*d*): δ 30.2.

<u>General Procedure for Starting Material Preparation 1B</u>

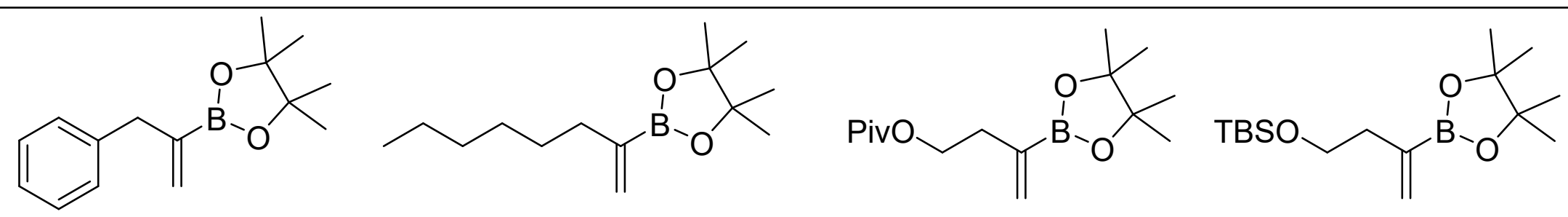

1c **1n** **1o** **1p**

Compounds **1c**, **1n**, **1o** and **1p** were prepared following General Procedure for Starting Material Preparation 1B.

<u>Step 1: Bromination of alkynes[3]</u>

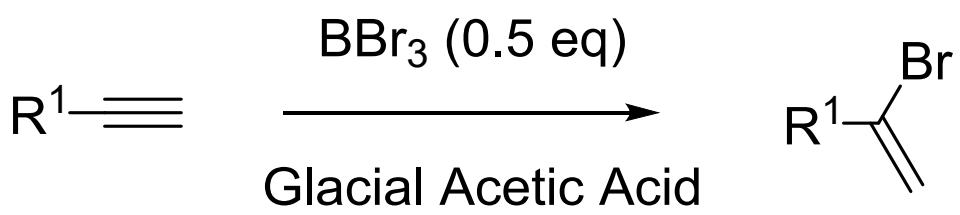

Alkyne (10 mmol) was added drop wise to a 1 M solution of boron tribromide (5.0 mmol) at –78 °C. The resulting solution was allowed to warm to room temperature over 3 h. Glacial acetic acid (10 mL) was added to the mixture and stirred for 1 h. This mixture was quenched

with water (10 mL), extracted with pentane (3×20 mL). Combined organic layers were washed with sat. $NaHCO_3$ solution (4×15 mL) until the pH of the water phase stays basic, then washed with water (15 mL) and brine (15 mL). After drying over $MgSO_4$, the solvent was removed under reduced pressure. The crude product was diluted with pentane (1 mL) and purified over a plug of silica (3 cm × 4 cm), followed by elution with *n*-pentane (300 mL). The solvent was removed under reduced pressure (>300 mbar, 40 °C) to obtain the intermediate vinyl bromide.

Step 2: Miyaura Borylation of vinyl bromides[4]

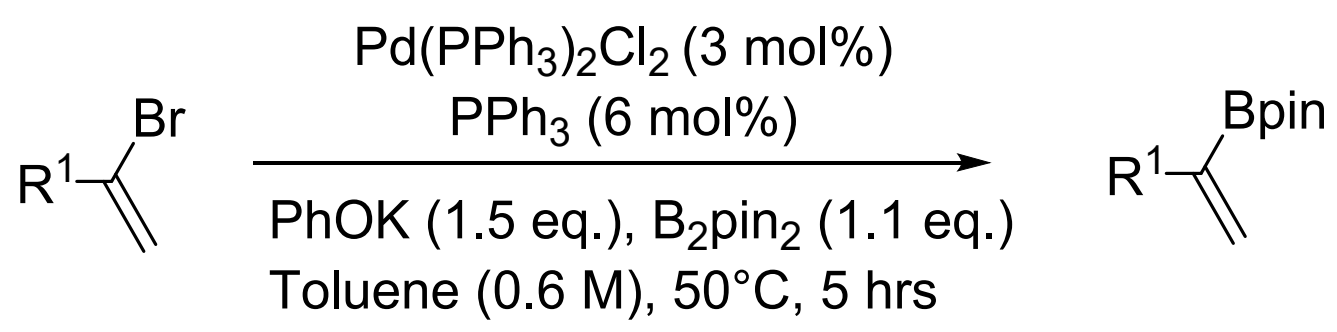

A mixture of $PdCl_2(PPh_3)_2$ (3 mol%), Ph_3P (6 mol-%), bis(pinacolato)dibron (1.1 eq.), and potassium phenoxide (1.5 eq.) was treated with vinyl bromide (1.0 eq.) and toluene (0.6 M). The red brown reaction mixture was stirred for 5 hours at 50 °C. After cooling down to rt the reaction mixture was extracted with water (10 mL). The water phase was washed with DCM (10 mL) and combined organic layers were washed with brine (15 mL). Drying over $MgSO_4$ and concentration in vacuo gave a red crude oil. Purification by flash chromatography (SiO_2) gave the desired vinylboronate.

The NMR data of the compounds **1c**, **1n, 1o** and **1p** are in accordance with the NMR data repported in the literature.[3,4]

General Procedure for Starting Material Preparation 1C

Preparation of Vinyl Iodide: 3-iodobut-3-en-1-ol

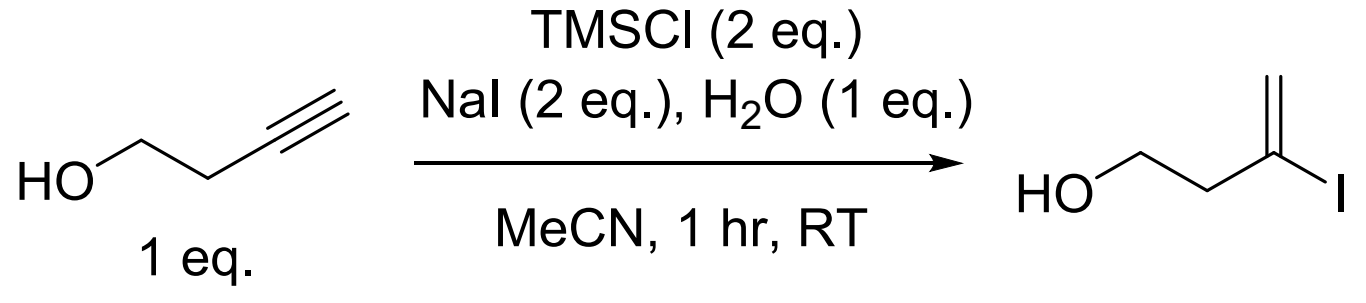

According to the Takayuki Shioiri group,[5] NaI (6.0 g, 40 mmol) was dissolved in CH_3CN (30 mL) at rt and then to the mixture was added TMSCl (5.08 mL, 40 mmol) followed by H_2O (360 μL, 20mmol). After 10 min, a solution of 3-butyn-1-ol (1.4 g, 20 mmol) in CH_3CN (5.0 mL) was added and the resulting mixture was allowed to react for 1 h at room temperature. The reaction was quenched with H_2O (60 mL) and the mixture was extracted with ether. Drying over Na_2SO4, filtration, and evaporating ether gave crude the iodo

alcohol (3.2 g, 81%) as a reddish-brown oil. The NMR data of 3-iodobut-3-en-1-ol are in accordance with the data reported in the literature.

Step 1: Esterification of Carboxylic Acid with Vinyl iodo alcohol

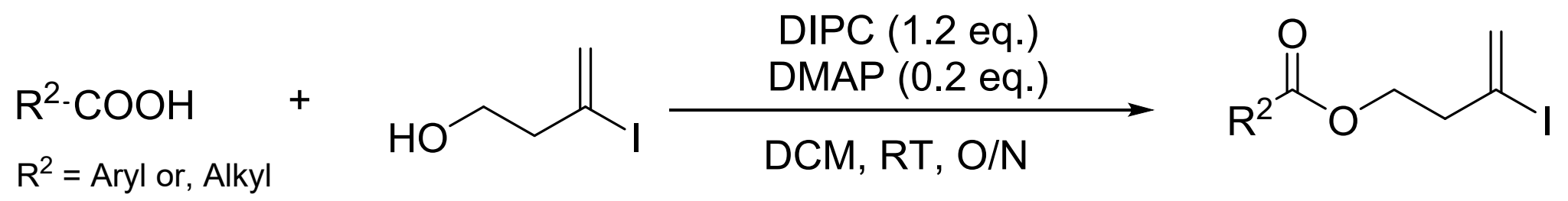

To a mixture of the aromatic or aliphatic acid (1.0 equiv.), 3-iodobut-3-en-1-ol (1.5 equiv.) and DMAP (0.2 equiv.) in DCM (0.2 M) was added *N*,*N*'- Diisopropylcarbodiimide (DIPC, 1.2 equiv.) dropwise. The reaction was stirred at room temperature overnight. The reaction was quenched with H_2O and extracted with CH_2Cl_2. The organic layers were washed with brine, dried over anhydrous Na_2SO_4, filtered, concentrated in vacuo. Flash chromatography afforded the desired vinyl iodides.

Step 2: Miyaura Borylation of Vinyl Iodides

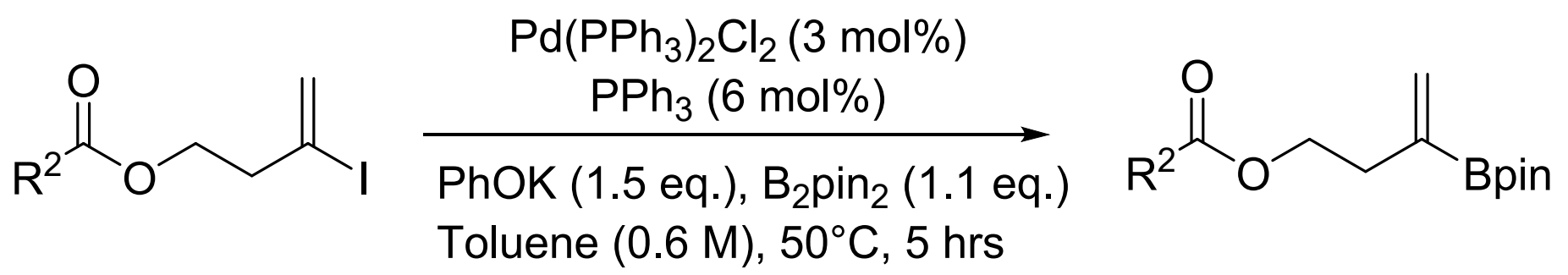

A mixture of $PdCl_2(PPh_3)_2$ (3 mol%), Ph_3P (6 mol%), bis(pinacolato)dibron (1.1 eq.), and potassium phenoxide (1.5 eq.) was treated with vinyl bromide (1.0 eq.) and toluene (0.6 M). The red brown reaction mixture was stirred for 5 hours at 50 °C. After cooling down to rt the reaction mixture was extracted with water (10 mL). The water phase was washed with DCM (10 mL) and combined organic layers were washed with brine (15 mL). Drying over $MgSO_4$ and concentration in vacuo gave a red crude oil. Purification by flash chromatography (SiO_2) gave the desired vinylboronate.

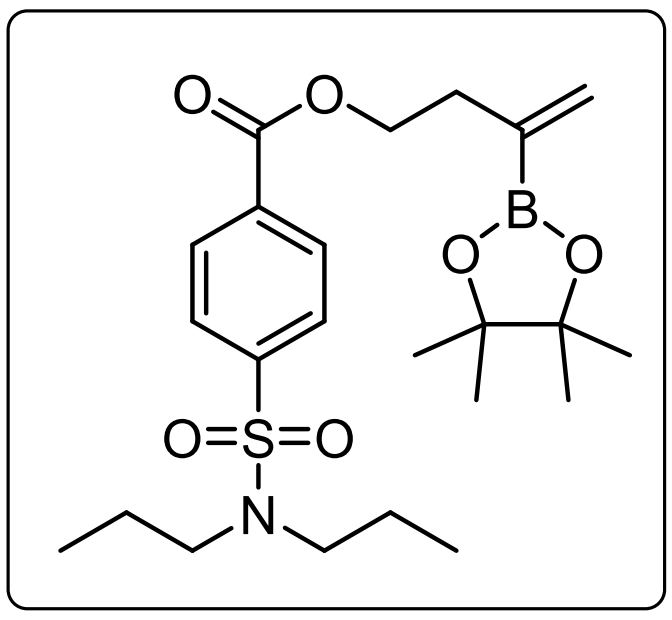

3-(4,4,5,5-tetramethyl-1,3,2-dioxaborolan-2-yl)but-3-en-1-yl 4-(N,N-dipropylsulfamoyl)benzoate (1p)

1p was prepared following General Procedure for Starting Material Preparation 1C in 5 mmol scale from probenecid. The desired product **1p** (954 mg; Overall yield: 41%) was isolated by flash column chromatography using hexane : ethyl acetate (90:10) as a white solid.

^{1}H NMR (400 MHz, Chloroform-*d*) δ 8.16 – 8.09 (m, 2H), 7.86 – 7.81 (m, 2H), 5.91 (d, *J* = 3.2 Hz, 1H), 5.75 (d, *J* = 3.6 Hz, 1H), 4.44 (t, *J* = 6.8 Hz, 2H), 3.14 – 3.04 (m, 4H), 2.62 (t, *J* = 6.8 Hz, 2H), 1.53 (hd, *J* = 7.6, 2.5 Hz, 4H), 1.25 (s, 12H), 0.86 (t, *J* = 7.4 Hz, 6H).

^{13}C NMR (100 MHz, Chloroform-*d*) δ 165.2, 144.0, 133.9, 132.3, 130.2, 126.9, 83.8, 64.9, 49.9, 34.6, 25.0, 24.8, 21.9, 11.2 The carbon attached to the boron was not found due to quadrupolar relaxation.

^{11}B NMR (128 MHz, Chloroform-*d*) δ 30.1

HRMS (ESI) calcd for $C_{23}H_{37}BNO_6S$ $[M+H]^+$: 466.2435; found: 466.2422.

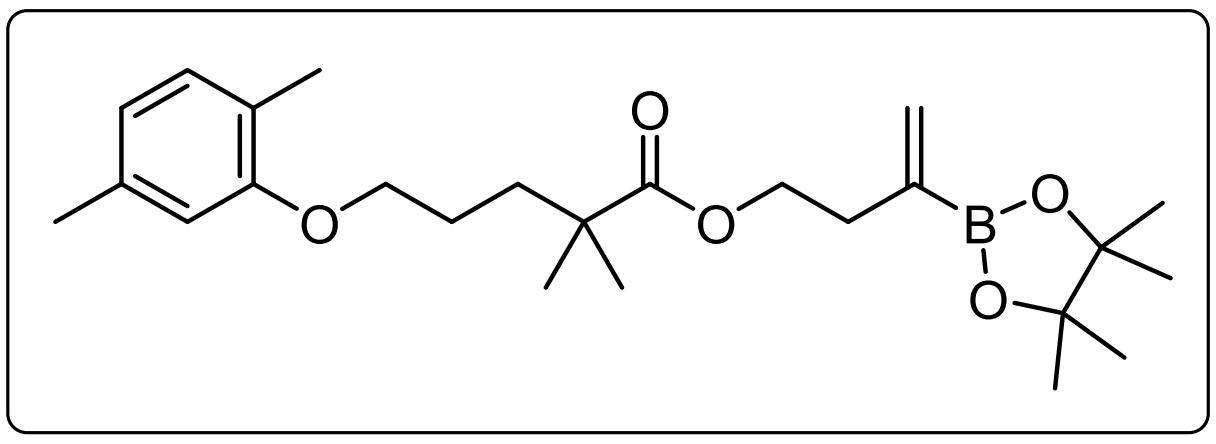

3-(4,4,5,5-tetramethyl-1,3,2-dioxaborolan-2-yl)but-3-en-1-yl 5-(2,5-dimethylphenoxy)-2,2-dimethylpentanoate (1q)

1q was prepared following General Procedure for Starting Material Preparation 1C in 5 mmol scale from gemfibrozil. The desired product **1q** (1.07 g; Overall yield: 50%) was isolated by flash column chromatography using hexane : ethyl acetate (95:5) as a colourless oil.

^{1}H NMR (400 MHz, Chloroform-*d*) δ 7.00 (d, *J* = 7.5 Hz, 1H), 6.65 (d, *J* = 7.4 Hz, 1H), 6.61 (d, *J* = 1.6 Hz, 1H), 5.88 (d, *J* = 3.3 Hz, 1H), 5.69 (d, *J* = 3.3 Hz, 1H), 4.18 (t, *J* = 6.8 Hz, 2H), 3.91 (t, *J* = 5.6 Hz, 2H), 2.48 (t, *J* = 6.8 Hz, 2H), 2.31 (s, 3H), 2.18 (s, 3H), 1.75 – 1.63 (m, 4H), 1.27 (s, 12H), 1.20 (s, 6H).

^{13}C NMR (100 MHz, Chloroform-*d*) δ 176.7, 155.9, 135.4, 130.9, 129.2, 122.6, 119.6, 110.9, 82.5, 66.9, 62.5, 41.0, 36.1, 33.7, 30.6, 25.9, 24.2, 24.2, 23.7, 20.4, 14.8 The carbon attached to the boron was not found due to quadrupolar relaxation.

^{11}B NMR (128 MHz, Chloroform-*d*) δ 29.9

HRMS (ESI) calcd for $C_{25}H_{39}BNaO_5$ $[M+Na]^+$: 453.2788; found: 453.2777.

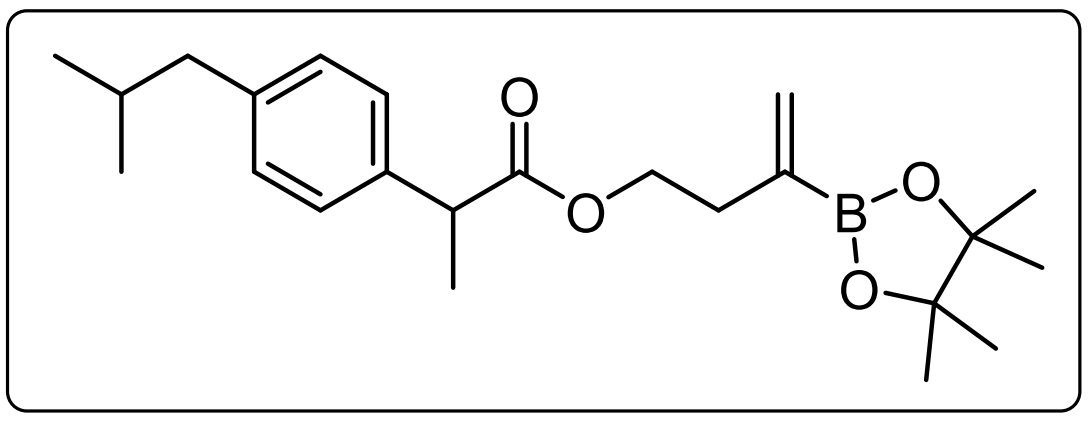

3-(4,4,5,5-tetramethyl-1,3,2-dioxaborolan-2-yl)but-3-en-1-yl 2-(4-isobutylphenyl)propanoate (1r)

1r was prepared following General Procedure for Starting Material Preparation 1C in 5 mmol scale from ibuprofen. The desired product **1r** (830.5 mg; Overall yield: 43%) was isolated by flash column chromatography using hexane : ethyl acetate (95:5) as a colourless oil.

^{1}H NMR (400 MHz, Chloroform-*d*) δ 7.19 (d, *J* = 7.8 Hz, 2H), 7.08 (d, *J* = 7.8 Hz, 2H), 5.81 (d, *J* = 3.3 Hz, 1H), 5.56 (d, *J* = 3.3 Hz, 1H), 4.22 – 4.11 (m, 2H), 3.66 (q, *J* = 7.1 Hz, 1H), 2.43 (dd, *J* = 10.7, 7.0 Hz, 4H), 1.85 (dh, *J* = 13.5, 6.7 Hz, 1H), 1.47 (d, *J* = 7.1 Hz, 3H), 1.25 (s, 12H), 0.89 (d, *J* = 6.6 Hz, 6H).

^{13}C NMR (100 MHz, Chloroform-*d*) δ 174.7, 140.4, 137.9, 132.0, 129.2, 127.2, 83.5, 63.9, 45.2, 45.1, 34.6, 30.2, 24.7, 22.4, 18.6. The carbon attached to the boron was not found due to quadrupolar relaxation.

^{11}B NMR (128 MHz, Chloroform-*d*) δ 29.7

HRMS (ESI) calcd for $C_{25}H_{35}BKO_4$ $[M+K]^+$: 425.2265; found: 425.2260.

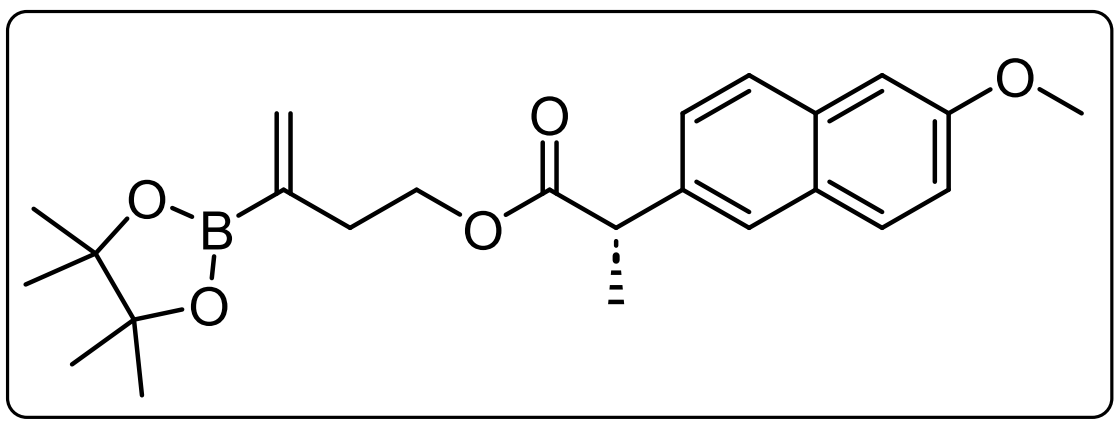

3-(4,4,5,5-tetramethyl-1,3,2-dioxaborolan-2-yl)but-3-en-1-yl (S)-2-(6-methoxynaphthalen-2-yl)propanoate (1s)

1s was prepared following General Procedure for Starting Material Preparation 1C in 5 mmol scale from naproxen. The desired product **1s** (1.05 g; Overall yield: 51%) was isolated by flash column chromatography using hexane : ethyl acetate (80:20) as a colourless oil.

^{1}H NMR (400 MHz, Chloroform-*d*) δ 7.60 (dd, *J* = 8.7, 5.0 Hz, 2H), 7.56 (d, *J* = 1.8 Hz, 1H), 7.31 (dd, *J* = 8.4, 1.9 Hz, 1H), 7.04 – 7.01 (m, 2H), 5.72 (d, *J* = 3.4 Hz, 1H), 5.48 (d, *J* = 3.3 Hz, 1H), 4.17 – 4.04 (m, 2H), 3.79 (s, 3H), 3.73 (q, *J* = 7.1 Hz, 1H), 2.34 (t, *J* = 6.9 Hz, 2H), 1.47 (d, *J* = 7.1 Hz, 3H), 1.14 (s, 12H).

^{13}C NMR (100 MHz, Chloroform-*d*) δ174.6, 157.6, 135.8, 133.7, 132.1, 129.3, 128.9, 127.1, 126.4, 125.9, 118.9, 105.6, 83.5, 64.0, 55.3, 45.5, 34.7, 24.8, 18.6. The carbon attached to the boron was not found due to quadrupolar relaxation.

^{11}B NMR (128 MHz, Chloroform-*d*) δ 30.2

HRMS (ESI) calcd for $C_{24}H_{32}BO_5$ $[M+K]^+$: 411.2343; found: 411.2342.

IV. General procedure for Three-component Conjunctive Coupling

General Procedure for Three-Component Conjunctive Coupling 1 (GP1)

In a reaction tube equipped with teflon screw cap containing teflon coated magnetic stir bar, the corresponding disulfide (0.75 eq., 0.15 mmol) was dissolved in 0.2 mL CPME under nitrogen atmosphere, then 0.3 mL $LiHBEt_3$ (1(M) in THF) (1.5 eq.) was added at -78 °C; stirred for 40 mins. Vinyl boronate (0.2 mmol) in 0.5 mL CPME was added to the reaction at -78 °C and stirred for 1 hr. The resulting reaction mixture was transferred to another reaction tube containing a suspension of Togni-II (1.5 eq., 0.3 mmol, 95 mg) in 0.2 mL CPME at -78 °C and stirred for 2 hrs at -78 °C. The reaction mixture was allowed to warm up to RT over 2 hrs and stirred at RT for another 8 hrs. After the completion of the reaction, the reaction was diluted with 2 mL ethyl acetate and quenched with 2 mL brine. The layers were separated and the aqueous layer was washed with ethyl acetate (2 mL × 3). The combined organic layer was dried over Na_2SO_4, filtered and concentrated under reduced pressure to obtain the crude product. ^{19}F NMR yields are reported using 3-trifluoromethylphenol as the internal standard. The pure product was obtained by silica gel flash coloumn chromatography.

General Procedure for Three-Component Conjunctive Coupling 2 (GP2)

In a reaction tube equipped with teflon screw cap containing teflon coated magnetic stir bar, the corresponding thiol (1.5 eq., 0.3 mmol) was dissolved in 0.2 mL CPME, then 0.3 mL BEt_3 (1(M) in THF) was added at -78 °C; stirred for 40 mins. Vinyl boronate (0.2 mmol) in 0.5 mL CPME was added to the reaction at -78 °C and stirred for 1 hr. The resulting reaction mixture was transferred to another reaction tube containing a suspension of Togni-II (1.5 eq., 0.3 mmol, 95 mg) in 0.2 mL CPME at -78°C and stirred for 2 hrs at -78 °C. The reaction mixture was allowed to warm up to RT over 2 hrs and stirred at RT for another 8 hrs. After the completion of the reaction, the reaction was diluted with 2 mL ethyl acetate and quenched with 2 mL brine. The layers were separated and the aqueous layer was washed with ethyl acetate (2 mL × 3). The combined organic layer was dried over Na_2SO_4, filtered and concentrated under reduced pressure to obtain the crude product. ^{19}F NMR yields are reported using 3-trifluoromethylphenol as the internal standard. The pure product was obtained by silica gel flash coloumn chromatography.

General Procedure for Scale-Up Reaction of Three-Component Conjunctive Coupling

The thiophenol (1.5 eq., 7.5 mmol, 825 mg) was dissolved in 2.5 mL CPME, then 7.5 mL BEt_3 (1(M) in THF) was added at -78 °C; stirred for 40 mins. Vinyl boronate (5 mmol) in 3 mL CPME was added to the reaction at -78 °C and stirred for 1 h. The resulting reaction mixture was transferred to a suspension of Togni-II (1.5 eq., 7.5 mmol, 2.4 g) in 0.2 mL CPME at -78 °C and stirred for 2 hrs at -78 °C. The reaction mixture was allowed to warm up to RT over 2 hrs and stirred at RT for another 12 hrs. After the completion of the reaction, the reaction was diluted with 10 mL ethyl acetate and quenched with 10 mL brine. The layers were separated and the aqueous layer was washed with ethyl acetate (15 mL × 3). The combined organic layer was dried over Na_2SO_4, filtered and concentrated under reduced pressure to obtain the crude product. The pure product was obtained after silica gel flash coloumn chromatography.

Failed Substrates

Vinyl Boronate:

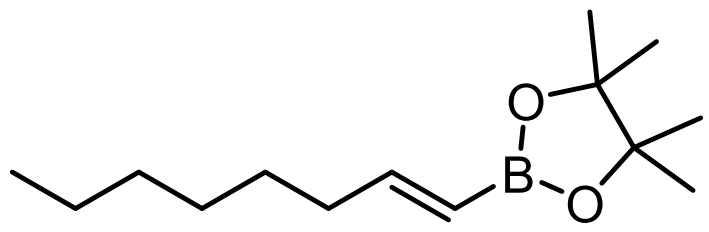

1q

(*E*)-4,4,5,5-tetramethyl-2-(oct-1-en-1-yl)-1,3,2-dioxaborolane

Thiol/Dsulfide:

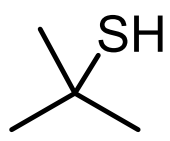

2p

2-methylpropane-2-thiol

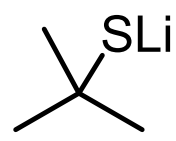

2p′

from

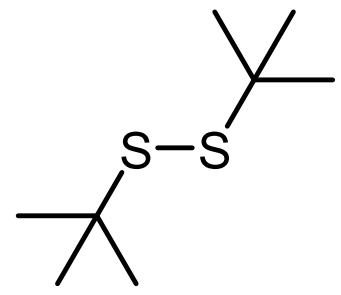

1,2-di-*tert*-butyldisulfane

Hypervalent Iodine Reagents:

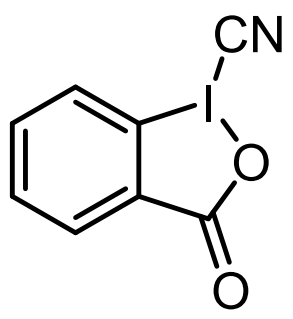

HI-1

3-oxo-1λ^3-benzo[*d*][1,2]iodaoxole-1(3*H*)-carbonitrile

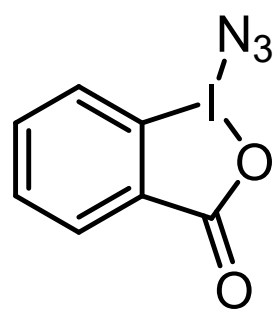

HI-2

1-azido-1λ^3-benzo[*d*][1,2]iodaoxol-3(1*H*)-one

Figure S1. Failed Substrates.

V. Detailed description of products

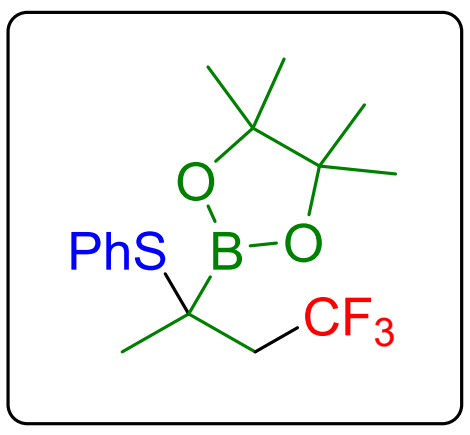

4,4,5,5-tetramethyl-2-(4,4,4-trifluoro-2-(phenylthio)butan-2-yl)-1,3,2-dioxaborolane (3aa)

The product **3aa** was synthesized following general procedure for Three-Component Conjunctive Coupling 2 (GP2) using thiophenol. **3aa** was isolated by flash column chromatography (200-400 mesh silica gel) using MTBE : hexane (0:100 →1:99) as colourless oil (64 mg, 92%).

^{1}H NMR (400 MHz, Chloroform-*d*) δ 7.51 – 7.44 (m, 2H), 7.32 – 7.17 (m, 3H), 2.61 (dq, *J* = 14.7, 11.6 Hz, 1H), 2.16 (dq, *J* = 14.7, 10.7 Hz, 1H), 1.28 (d, *J* = 1.8 Hz, 3H), 1.18 (s, 6H), 1.16 (s, 6H).

^{13}C NMR (126 MHz, Chloroform-*d*) δ 137.5, 130.4, 129.3, 128.8,126.3 (q, *J* = 279.4 Hz) ,84.5, 42.9 (q, *J* = 26.9 Hz), 24.9, 24.7, 22.2. The carbon attached to the boron was not found due to quadrupolar relaxation.

^{11}B NMR (128 MHz, Chloroform-*d*) δ 31.8.

^{19}F NMR (376 MHz, Chloroform-*d*) δ -60.59 (t, *J* = 11.1 Hz).

HRMS (ESI) calcd for $C_{16}H_{22}BF_3NaO_2S$ $[M+Na]^+$: 369.1283; found: 369.1286.

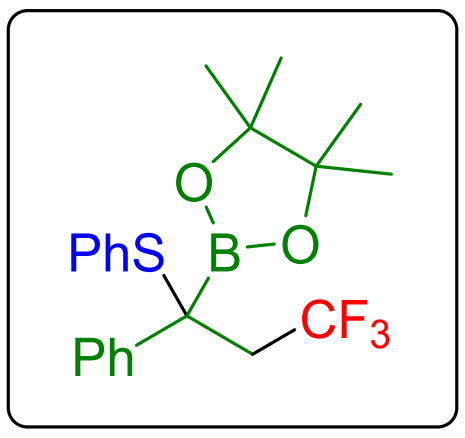

4,4,5,5-tetramethyl-2-(3,3,3-trifluoro-1-phenyl-1-(phenylthio)propyl)-1,3,2-dioxaborolane (3ba).

The product **3ba** was synthesized following general procedure for Three-Component Conjunctive Coupling 2 (GP2) using thiophenol. **3ba** is highly unstable in silica gel and while silica gel flash coloumn chromatography it decomposed rapidly. **3ba** was isolated in the following method. The crude reaction mixture completely dried under high vacuum to evaporate the volatiles. In the obtained mixture cold n-pentane was added. The resulting suspension was filtered carefully followed by cold n-pentane wash (5ml × 3). The filtrate was dried under reduced pressure to obtain **3ba** as a colourless oil (36.7 mg, 45%).

^{1}H NMR (400 MHz, Chloroform-*d*) δ 7.37 – 7.35 (m, 2H), 7.19 – 7.15 (m, 2H), 7.10 (ddd, J = 7.7, 5.8, 1.6 Hz, 2H), 7.01 – 6.95 (m, 4H), 2.81 (dddd, J = 25.0, 14.8, 10.3, 4.5 Hz, 2H), 1.19 (s, 6H), 1.12 (s, 6H).

^{13}C NMR (126 MHz, Chloroform-*d*) δ 140.6, 135.3, 131.3, 128.3, 128.2, 128.2, 128.1, 126.8, 126.3 (q, *J* = 279.3 Hz), 84.8, 39.9 (q, *J* = 27.1 Hz), 24.6, 24.5

^{11}B NMR (128 MHz, Chloroform-*d*) δ 31.7

^{19}F NMR (376 MHz, Chloroform-*d*) δ -58.27 (t, *J* = 10.3 Hz).

HRMS (ESI) calcd for $C_{21}H_{24}BF_3NaO_2S$ $[M+Na]^+$: 431.1440; found: 431.1458.

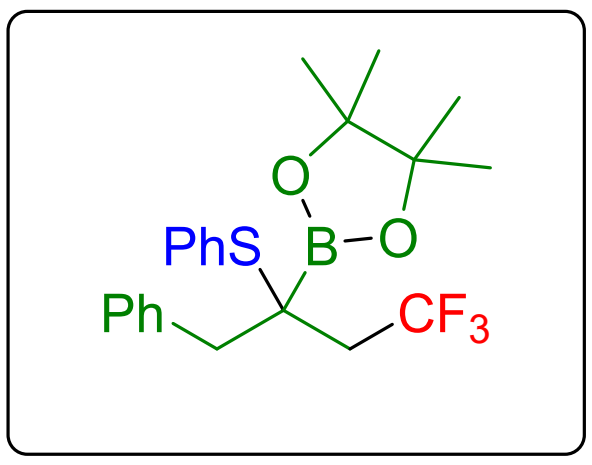

4,4,5,5-tetramethyl-2-(4,4,4-trifluoro-1-phenyl-2-(phenylthio)butan-2-yl)-1,3,2-dioxaborolane (3ca)

The product **3ca** was synthesized following general procedure for Three-Component Conjunctive Coupling 2 (GP2) using thiophenol. **3ca** was isolated by flash column chromatography (200-400 mesh silica gel) using MTBE : hexane (0:100→1:99) as colourless oil (42.2 mg, 50%).

^{1}H NMR (400 MHz, Chloroform-*d*) δ 7.45 (d, *J* = 6.7 Hz, 2H), 7.34 (d, *J* = 7.1 Hz, 2H), 7.26-7.16 (m, 6H), 3.02 (q, *J* = 15 Hz, 2H), 2.47-2.28 (m, 2H), 1.07 (s, 6H), 1.06 (s, 6H).

^{13}C NMR (126 MHz, Chloroform-*d*) δ 137.1, 136.5, 131.5, 128.7, 128.6, 127.9, 126.7, 126.6 (q, *J* = 279.7 Hz), 84.5, 40.2, 38.6 (q, *J* = 26.9 Hz), 25.1, 24.8. The carbon attached to the boron was not found due to quadrupolar relaxation.

^{11}B NMR (128 MHz, Chloroform-*d*) δ 31.6.

^{19}F NMR (376 MHz, Chloroform-*d*) δ -57.45 (t, *J* = 10.9 Hz).

HRMS (ESI) calcd for $C_{22}H_{26}BF_3NaO_2S$ $[M+Na]^+$: 445.1596; found: 445.1596.

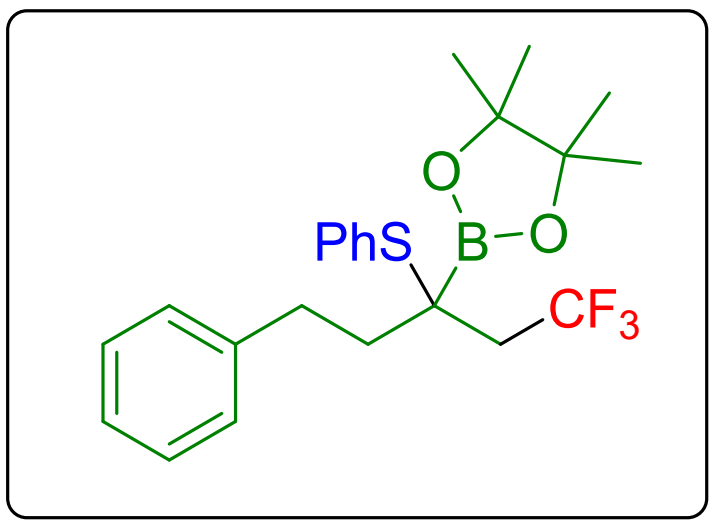

4,4,5,5-tetramethyl-2-(1,1,1-trifluoro-5-phenyl-3-(phenylthio)pentan-3-yl)-1,3,2-dioxaborolane (3da)

The product **3da** was synthesized following General Procedure for Three-Component Conjunctive Coupling 2 (GP2) using thiophenol. **3da** was isolated by flash column

chromatography (200-400 mesh silica gel) using MTBE : hexane (0:100 → 1:99) as white solid (71.5 mg, 82%).

^{1}H NMR (400 MHz, Chloroform-*d*) δ 7.58 – 7.51 (m, 2H), 7.33 – 7.15 (m, 5H), 7.14 – 7.05 (m, 3H), 2.93 – 2.79 (m, 2H), 2.58 – 2.30 (m, 2H), 1.95 (ddd, *J* = 14.7, 10.4, 6.7 Hz, 1H), 1.80 (ddd, *J* = 14.7, 10.5, 6.6 Hz, 1H), 1.25 (s, 6H), 1.23 (s, 6H).

^{13}C NMR (101 MHz, Chloroform-*d*) δ 142.2, 136.9, 130.7, 129.1, 128.8, 128.5, 128.4, 126.3 (q, *J* = 279.1 Hz), 84.5, 39.7 (q, *J* = 26.78 Hz), 36.2, 30.8, 24.9, 24.8. The carbon attached to the boron was not found due to quadrupolar relaxation.

^{11}B NMR (128 MHz, Chloroform-*d*) δ 31.8.

^{19}F NMR (376 MHz, Chloroform-*d*) δ -59.78 (t, *J* = 10.83).

HRMS (ESI) calcd for $C_{23}H_{29}BF_3O_2S$ $[M+H]^+$: 437.1933; found: 437.1936.

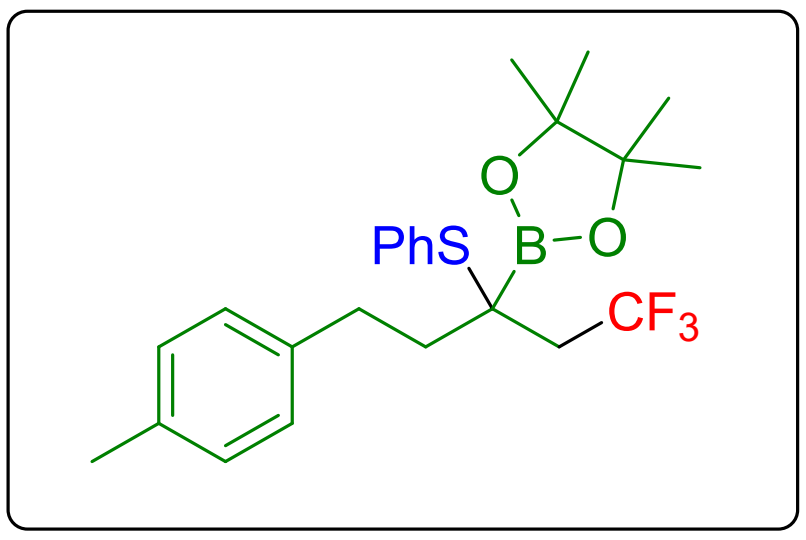

4,4,5,5-tetramethyl-2-(1,1,1-trifluoro-3-(phenylthio)-5-(p-tolyl)pentan-3-yl)-1,3,2-dioxaborolane (3ea)

The product **3ea** was synthesized following General Procedure for Three-Component Conjunctive Coupling 2 (GP2) using thiophenol. **3ea** was isolated by flash column chromatography (200-400 mesh silica gel) using MTBE : hexane (0:100 to 1:99) as colourless solid (73 mg, 81%).

^{1}H NMR (400 MHz, Chloroform-*d*) δ 7.64 (dd, *J* = 7.8, 1.8 Hz, 2H), 7.40 – 7.32 (m, 3H), 7.08 (q, *J* = 8.1 Hz, 4H), 2.94 – 2.89 (m, 2H), 2.67 – 2.42 (m, 2H), 2.33 (s, 3H), 2.07 – 1.99 (m, 1H), 1.91 – 1.80 (m, 1H), 1.27 (s, 6H), 1.25 (s, 6H).

^{13}C NMR (125 MHz, Chloroform-*d*) δ 138.0, 135.9, 134.2, 129.7, 128.0, 127.9, 127.7, 127.3, 125.3 (q, *J* = 279.3 Hz), 83.4, 38.6 (q, *J* = 26.9 Hz), 35.9, 35.3, 29.3, 23.9, 23.7, 19.9

^{11}B NMR (160 MHz, Chloroform-*d*) δ 31.9

^{19}F NMR (376 MHz, Chloroform-*d*) δ -59.70 (t, *J* = 11.4 Hz).

HRMS (ESI) calcd for $C_{24}H_{30}BF_3NaO_2S$ $[M+Na]^+$: 473.1909; found: 473.1920.

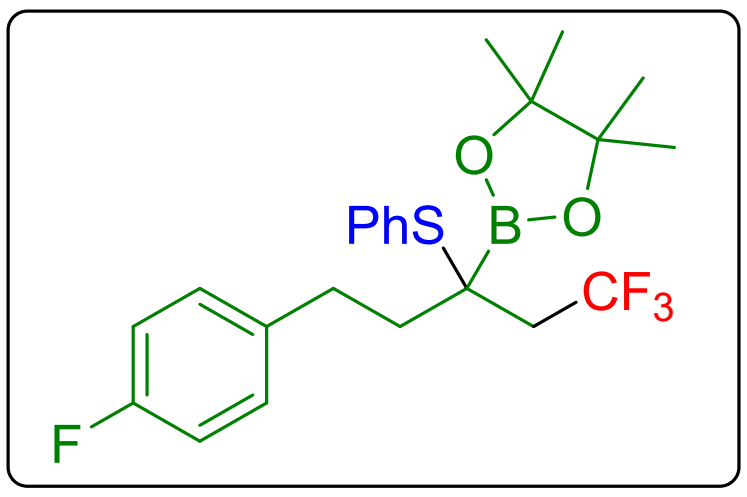

4,4,5,5-tetramethyl-2-(1,1,1-trifluoro-5-(4-fluorophenyl)-3-(phenylthio)pentan-3-yl)-1,3,2-dioxaborolane (3fa)

The product **3fa** was synthesized following General Procedure for Three-Component Conjunctive Coupling 2 (GP2) using thiophenol. **3fa** was isolated by flash column chromatography (200-400 mesh silica gel) using MTBE : hexane (0:100 → 1:99) as colourless solid (74.5 mg, 82%).

^{1}H NMR (400 MHz, Chloroform-*d*) δ 7.63 (d, *J* = 7.2 Hz, 2H), 7.36 (p, *J* = 6.4 Hz, 3H), 7.10 (dd, *J* = 8.2, 5.6 Hz, 2H), 6.95 (t, *J* = 8.7 Hz, 2H), 3.00 – 2.83 (m, 2H), 2.68 – 2.40 (m, 2H), 2.02 (td, *J* = 13.7, 12.8, 4.9 Hz, 1H), 1.87 (ddd, *J* = 16.6, 12.7, 5.0 Hz, 1H), 1.26 (s, 6H), 1.24 (s, 6H).

^{13}C NMR (100 MHz, Chloroform-*d*) δ 161.4 (d, *J* = 243.4 Hz), 137.9 (d, *J* = 2.7 Hz), 136.9, 130.8, 129.9 (d, *J* = 8.2 Hz), 129.2, 128.9, 126.4 (q, *J* = 279.4), 115.2 (d, *J* = 20.9 Hz), 84.6, 39.7 (q, *J* = 26.9 Hz), 36.9 (br), 36.2, 29.9, 25.1, 24.9.

^{11}B NMR (128 MHz, Chloroform-*d*) δ 31.9

^{19}F NMR (376 MHz, Chloroform-*d*) δ -59.85 (t, *J* = 10.9 Hz, 3F), -117.70 (ttd, *J* = 8.0, 5.4, 2.0 Hz, 1F).

HRMS (ESI) calcd for $C_{23}H_{28}BF_4O_2S$ $[M+H]^+$: 455.1839; found: 455.1818.

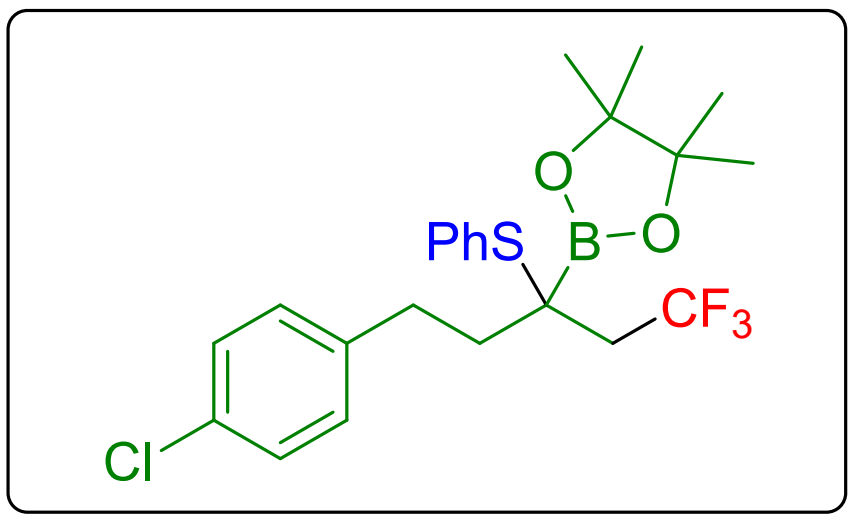

2-(5-(4-chlorophenyl)-1,1,1-trifluoro-3-(phenylthio)pentan-3-yl)-4,4,5,5-tetramethyl-1,3,2-dioxaborolane (3ga)

The product **3ga** was synthesized following General Procedure for Three-Component Conjunctive Coupling 2 (GP2) using thiophenol. **3ga** was isolated by flash column chromatography (200-400 mesh silica gel) using MTBE : hexane (0:100 → 1:99) as white solid (70.5 mg, 75%).

^{1}H NMR (400 MHz, Chloroform-*d*) δ 7.53 – 7.51 (m, 2H), 7.31 – 7.23 (m, 3H), 7.16 – 7.13 (m, 2H), 6.99-6.97 (m, 2H), 2.89-2.74 (m, 2H), 2.55 – 2.30 (m, 2H), 1.91 (ddd, J = 14.7, 12.1, 5.0 Hz, 1H), 1.76 (ddd, J = 14.7, 12.1, 5.2 Hz, 1H), 1.17 (s, 6H), 1.15 (s, 6H).

^{13}C NMR (125 MHz, Chloroform-*d*) δ 140.8, 136.9, 131.7, 130.8, 129.9, 129.2, 128.9, 128.6, 126.4 (q, *J* = 279.2 Hz), 84.6, 39.7 (q, *J* = 27.2 Hz), 36.0, 30.1, 25.1, 24.9. The carbon attached to the boron was not found due to quadrupolar relaxation.

^{11}B NMR (128 MHz, Chloroform-*d*) δ 31.6

^{19}F NMR (376 MHz, Chloroform-*d*) δ -59.89 (t, *J* = 10.9 Hz).

HRMS (ESI) calcd for $C_{23}H_{28}BClF_3O_2S$ $[M+H]^+$: 471.1544; found: 471.1533.

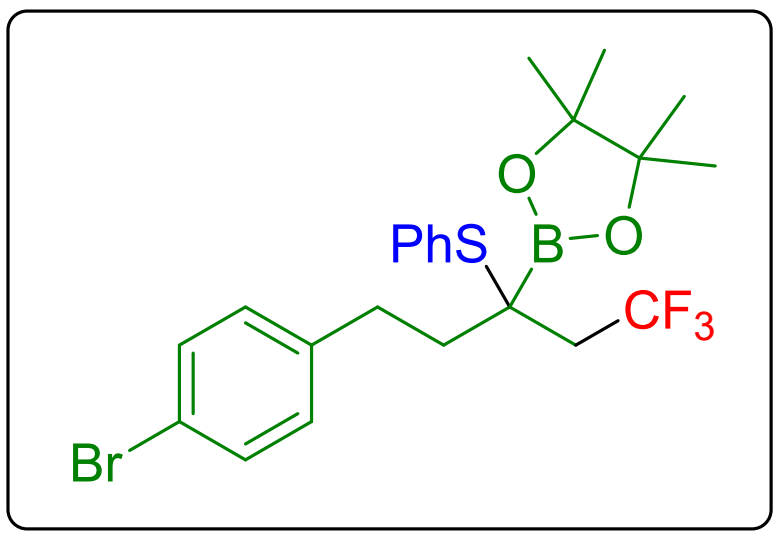

2-(5-(4-bromophenyl)-1,1,1-trifluoro-3-(phenylthio)pentan-3-yl)-4,4,5,5-tetramethyl-1,3,2-dioxaborolane (3ha)

The product **3ha** was synthesized following General Procedure for Three-Component Conjunctive Coupling 2 (GP2) using thiophenol. **3ha** was isolated by flash column

chromatography (200-400 mesh silica gel) using MTBE : hexane (0:100 → 1:99) as white solid (69 mg, 67%).

^{1}H NMR (400 MHz, Chloroform-*d*) δ 7.61 – 7.58 (m, 2H), 7.39 – 7.3 (m, 5H), 7.01 – 6.99 (m, 2H), 2.95-2.8 (m, 2H), 2.63 – 2.38 (m, 2H), 1.99 (ddd, J = 16.9, 12.3, 5.0 Hz, 1H), 1.84 (ddd, J = 14.6, 12.1, 5.1 Hz, 1H), 1.24 (s, 6H), 1.22 (s, 6H).

^{13}C NMR (100 MHz, Chloroform-*d*) δ 141.2, 136.8, 131.4, 130.6, 130.3, 129.1, 128.8, 126.2 (q, *J* = 279.5 Hz), 119.6, 84.5, 39.6 (q, *J* = 26.9 Hz), 35.8, 30.1, 24.9, 24.8. The carbon attached to the boron was not found due to quadrupolar relaxation.

^{11}B NMR (128 MHz, Chloroform-*d*) δ 31.9

^{19}F NMR (376 MHz, Chloroform-*d*) δ -59.89 (t, *J* = 11.4 Hz).

HRMS (ESI) calcd for $C_{23}H_{28}BBrF_3O_2S$ $[M+H]^+$: 515.1039; found: 515.1023.

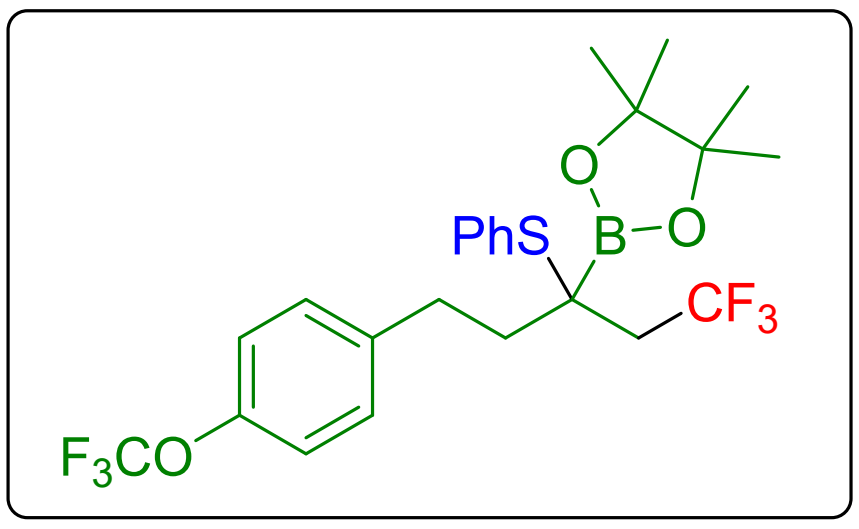

4,4,5,5-tetramethyl-2-(1,1,1-trifluoro-3-(phenylthio)-5-(4-(trifluoromethoxy)phenyl)pentan-3-yl)-1,3,2-dioxaborolane (3ia)

The product **3ia** was synthesized following General Procedure for Three-Component Conjunctive Coupling 2 (GP2) using thiophenol. **3ia** was isolated by flash column chromatography (200-400 mesh silica gel) using MTBE : hexane (0:100 → 1:99) as colourless solid (88.4 mg, 85%).

^{1}H NMR (400 MHz, Chloroform-*d*) δ7.63 – 7.61 (m, 2H), 7.36 (dt, *J* = 8.4, 6.5 Hz, 3H), 7.13 (q, *J* = 8.5 Hz, 4H), 2.94 (dtd, *J* = 32.7, 13.1, 4.8 Hz, 2H), 2.53 (ddq, *J* = 52.9, 15.0, 10.9 Hz, 2H), 2.06 – 1.84 (m, 2H). 1.89 (td, *J* = 14.9, 13.9, 5.0 Hz, 1H), 1.25 (s, 6H), 1.23 (s, 6H).

^{13}C NMR (100 MHz, Chloroform-*d*) δ 147.4 (d, *J* = 2.3 Hz), 140.9, 136.8, 130.6, 129.7, 129.1, 128.8, 126.3 (q, *J* = 279.2 Hz), 120.9, 120.5 (q, *J* = 256.5 Hz), 84.5, 39.6 (q, *J* = 27.1 Hz), 35.8, 29.9, 24.9, 24.7. The carbon attached to the boron was not found due to quadrupolar relaxation.

^{11}B NMR (128 MHz, Chloroform-*d*) δ 32.1

^{19}F NMR (376 MHz, Chloroform-*d*) δ -57.84 (s, 3F), -59.95 (t, *J* = 10.9 Hz, 3F).

HRMS (ESI) calcd for $C_{24}H_{28}BF_6O_3S$ $[M+H]^+$: 521.1756; found: 521.1756.

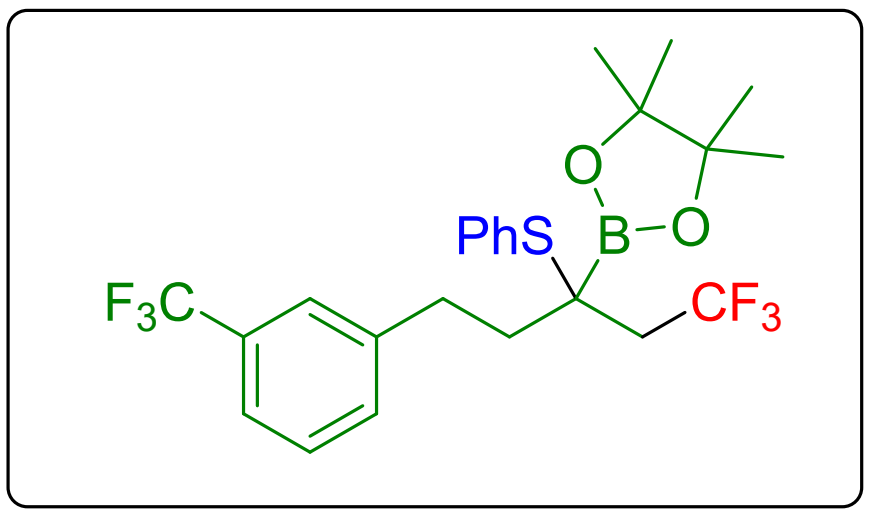

4,4,5,5-tetramethyl-2-(1,1,1-trifluoro-3-(phenylthio)-5-(3-(trifluoromethyl)phenyl)pentan-3-yl)-1,3,2-dioxaborolane (3ja)

The product **3ja** was synthesized following General Procedure for Three-Component Conjunctive Coupling 2 (GP2) using thiophenol. **3ja** was isolated by flash column chromatography (200-400 mesh silica gel) using MTBE : hexane (0:100 → 1:99) as colourless solid (79.6 mg, 79%).

^{1}H NMR (400 MHz, Chloroform-*d*) δ7.66 – 7.59 (m, 2H), 7.44 (d, *J* = 7.8 Hz, 1H), 7.40 – 7.30 (m, 6H), 2.99 (dtd, *J* = 33.7, 13.0, 4.8 Hz, 2H), 2.55 (ddq, *J* = 55.6, 15.1, 10.9, 10.5 Hz, 2H), 2.03 (ddd, *J* = 16.8, 12.5, 4.7 Hz, 1H), 1.90 (ddd, *J* = 14.7, 12.2, 5.0 Hz, 1H), 1.27 (s, 6H), 1.25 (s, 6H).

^{13}C NMR (125 MHz, Chloroform-*d*) δ 143.2, 136.9, 132.1, 130.8 (q, *J* = 32.4 Hz), 129.3, 129.0, 128.9, 126.4 (q, *J* = 279.4 Hz), 125.3 (q, *J* = 3.6 Hz), 124.4 (q, *J* = 272.5 Hz), 122.9 (q, *J* = 3.6 Hz), 84.7, 39.8 (q, *J* = 26.9 Hz), 36.9 (br), 35.8, 30.6, 25.1, 24.9.

^{11}B NMR (160 MHz, Chloroform-*d*) δ 31.7.

^{19}F NMR (376 MHz, Chloroform-*d*) δ -59.96 (t, *J* = 10.9, 3F), -62.52 (s, 3F).

HRMS (ESI) calcd for $C_{24}H_{28}BF_6O_2S$ $[M+H]^+$: 505.1807; found: 505.1785.

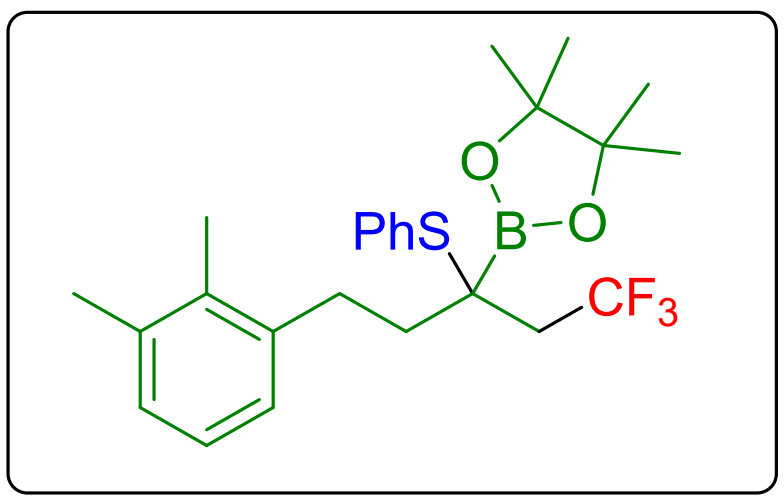

2-(5-(2,3-dimethylphenyl)-1,1,1-trifluoro-3-(phenylthio)pentan-3-yl)-4,4,5,5-tetramethyl-1,3,2-dioxaborolane (3ka)

The product **3ka** was synthesized following General Procedure for Three-Component Conjunctive Coupling 2 (GP2) using thiophenol. **3ka** was isolated by flash column chromatography (200-400 mesh silica gel) using MTBE : hexane (0:100 → 1:99) as colourless solid (57 mg, 61%).

^{1}H NMR (400 MHz, Chloroform-*d*) δ 7.65 – 7.62 (m, 2H), 7.38 – 7.30 (m, 3H), 7.04 – 6.95 (m, 3H), 2.96 (dtd, *J* = 40.8, 13.0, 4.7 Hz, 2H), 2.63 – 2.43 (m, 2H), 2.27 (s, 3H), 2.21 (s, 3H), 1.98 (ddd, *J* = 16.6, 12.6, 4.3 Hz, 1H), 1.84 (ddd, *J* = 14.2, 12.4, 4.9 Hz, 1H)., 1.25 (s, 6H), 1.24 (s, 6H).

^{13}C NMR (125 MHz, Chloroform-*d*) δ 140.4, 137.0, 136.9, 134.8 131.8, 131.1, 129.1, 128.9 127.9, 127.1, 126.4 (q, *J* = 279.4 Hz), 125.6, 84.6, 39.9 (q, *J* = 27.0 Hz), 35.3, 28.9, 25.1, 24.9, 20.9, 15.1 The carbon attached to the boron was not found due to quadrupolar relaxation.

^{11}B NMR (128 MHz, Chloroform-*d*) δ 32.3

^{19}F NMR (376 MHz, Chloroform-*d*) δ -59.63 (t, *J* = 11.08 Hz).

HRMS (ESI) calcd for $C_{25}H_{33}BF_3O_2S$ $[M+H]^+$: 465.2246; found: 465.2279.

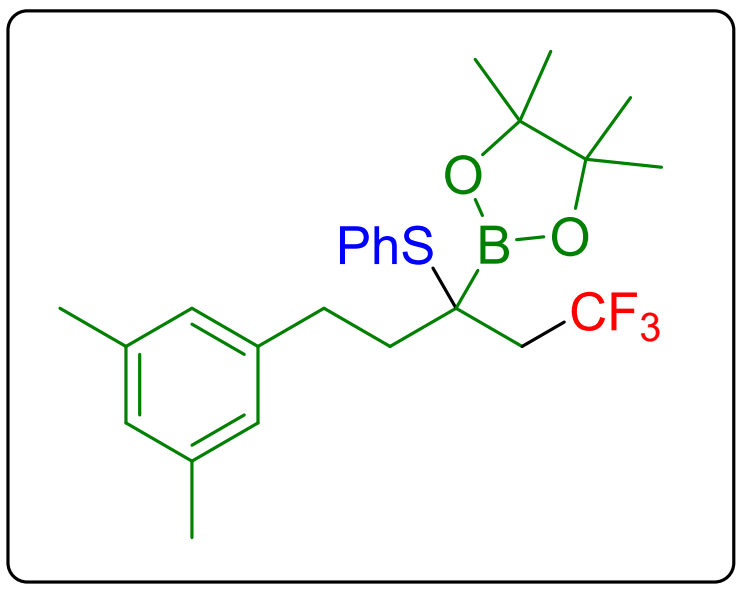

2-(5-(3,5-dimethylphenyl)-1,1,1-trifluoro-3-(phenylthio)pentan-3-yl)-4,4,5,5-tetramethyl-1,3,2-dioxaborolane (3la)

The product **3la** was synthesized following General Procedure for Three-Component Conjunctive Coupling 2 (GP2) using thiophenol. **3la** was isolated by flash column chromatography (200-400 mesh silica gel) using MTBE : hexane (0:100 → 1:99) as colourless solid (67 mg, 72%).

^{1}H NMR (400 MHz, Chloroform-*d*) δ 7.66 – 7.63 (m, 2H), 7.40 – 7.32 (m, 3H), 6.83 (s, 1H), 6.76 (s, 2H), 2.90 – 2.80 (m, 2H), 2.60 – 2.44 (m, 2H), 2.29 (s, 6H), 2.05 – 1.97 (m, 1H), 1.91 – 1.80 (m, 1H)., 1.26 (s, 6H), 1.25 (s, 6H).

^{13}C NMR (125 MHz, Chloroform-*d*) δ 142.1, 137.9, 136.9, 130.9, 129.1, 128.8, 127.5, 127.4, 126.4 (q, *J* = 279.4 Hz), 84.5, 39.7 (q, *J* = 26.9 Hz), 36.3, 30.6, 25.1, 24.9, 21.3. The carbon attached to the boron was not found due to quadrupolar relaxation.

^{11}B NMR (128 MHz, Chloroform-*d*) δ 32.1

^{19}F NMR (470 MHz, Chloroform-*d*) δ -59.63 (t, *J* = 11.27 Hz).

HRMS (ESI) calcd for $C_{25}H_{33}BF_3O_2S$ $[M+H]^+$: 465.2246; found: 465.2255.

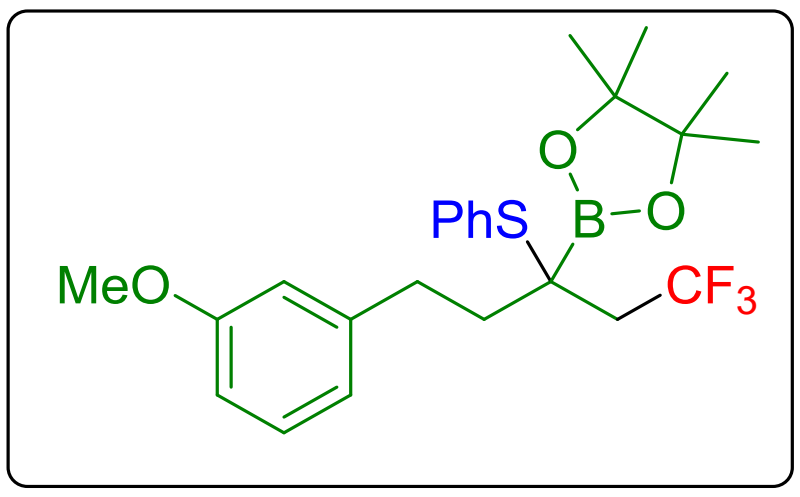

4,4,5,5-tetramethyl-2-(1,1,1-trifluoro-5-(3-methoxyphenyl)-3-(phenylthio)pentan-3-yl)-1,3,2-dioxaborolane (3ma)

The product **3ma** was synthesized following General Procedure for Three-Component Conjunctive Coupling 2 (GP2) using thiophenol. **3ma** was isolated by flash column chromatography (200-400 mesh silica gel) using MTBE : hexane (0:100 → 5:95) as colourless solid (67 mg, 72%).

^{1}H NMR (400 MHz, Chloroform-*d*) δ 7.65 – 7.59 (m, 2H), 7.39 – 7.30 (m, 3H), 7.18 (t, *J* = 7.8 Hz, 1H), 6.76 – 6.70 (m, 2H), 6.68 (t, *J* = 2.0 Hz, 1H), 3.78 (s, 3H), 2.94 – 2.87 (m, 2H), 2.59 – 2.42 (m, 2H), 2.07 – 1.95 (m, 1H), 1.91 – 1.81 (m, 1H), 1.24 (s, 6H), 1.22 (s, 6H).

^{13}C NMR (126 MHz, Chloroform-*d*) δ 159.6, 143.8, 136.9, 130.7, 129.3, 129.1, 128.9, 128.4, 126.3 (q, *J* = 279.1 Hz), 120.9, 114.2, 111.1, 84.5, 55.2, 39.7 (q, *J* = 26.9 Hz), 36.0, 30.8, 24.9, 24.8. The carbon attached to the boron was not found due to quadrupolar relaxation.

^{11}B NMR (128 MHz, Chloroform-*d*) δ 31.8.

^{19}F NMR (376 MHz, Chloroform-*d*) δ -59.78 (t, *J* = 11.0 Hz).

HRMS (ESI) calcd for $C_{24}H_{31}BF_3O_3S$ $[M+H]^+$: 467.2039; found: 467.2034

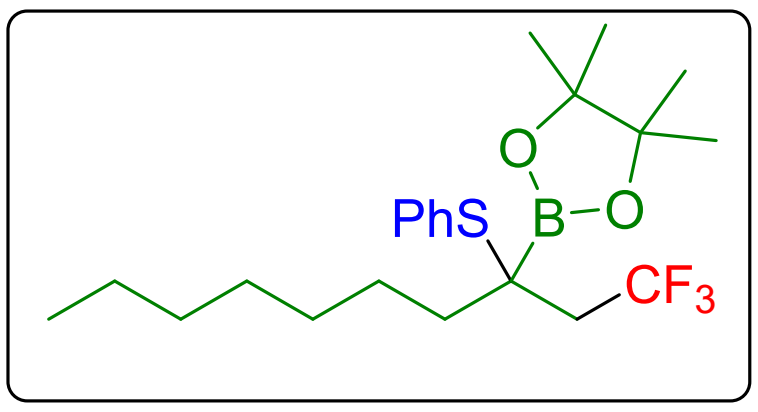

4,4,5,5-tetramethyl-2-(1,1,1-trifluoro-3-(phenylthio)decan-3-yl)-1,3,2-dioxaborolane (3na)

The product **3na** was synthesized following General Procedure for Three-Component Conjunctive Coupling 2 (GP2) using thiophenol. **3na** was isolated by flash column chromatography (200-400 mesh silica gel) using MTBE : hexane (0:100 → 1:99) as colourless oil (53.4 mg, 62%).

^{1}H NMR (500 MHz, Chloroform-*d*) δ 7.55 – 7.53 (m, 2H), 7.35 – 7.29 (m, 3H), 2.49 – 2.33 (m, 2H), 1.68 – 1.53 (m, 4H), 1.28 – 1.25 (m, 6H), 1.23 (s, 6H), 1.21 (s, 6H), 0.88 (t, *J* = 6.3 Hz, 3H).

^{13}C NMR (125 MHz, Chloroform-*d*) δ 135.9, 129.8, 127.8, 127.6, 125.3 (q, *J* = 279.1 Hz), 83.3, 38.5 (q, *J* = 26.64 Hz), 36.2 (br), 33.1, 30.7, 28.4, 23.9, 23.7, 23.3, 21.6, 13.0
^{11}B NMR (160 MHz, Chloroform-*d*) δ 32.1.

^{19}F NMR (470 MHz, Chloroform-*d*) δ -59.63 (t, *J* = 11.03 Hz).

HRMS (ESI) calcd for $C_{21}H_{33}BF_3O_2S$ $[M+H]^+$: 417.2246; found: 417.2261

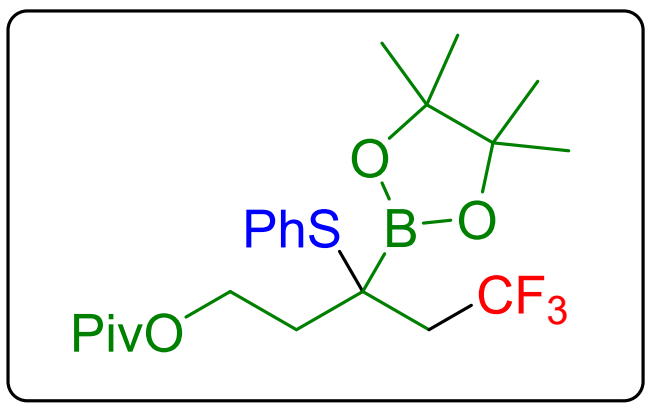

5,5,5-trifluoro-3-(phenylthio)-3-(4,4,5,5-tetramethyl-1,3,2-dioxaborolan-2-yl)pentyl pivalate (3oa)

The product **3oa** was synthesized following General Procedure for Three-Component Conjunctive Coupling 2 (GP2) using thiophenol. **3oa** was isolated by flash column chromatography (200-400 mesh silica gel) using MTBE : hexane (0:100 → 1:99) as colourless oil (64.5 mg, 70%).

^{1}H NMR (400 MHz, Chloroform-*d*) δ 7.58 – 7.55(m, 2H), 7.34 (tt, *J* = 8.8, 6.0 Hz, 3H), 4.46 (ddt, *J* = 10.9, 8.0, 4.0 Hz, 2H), 2.58 (dq, *J* = 15.0, 11.4 Hz, 1H), 2.32 (dq, *J* = 15.0, 10.5 Hz,

1H), 2.13 (ddd, J = 15.0, 9.0, 6.3 Hz, 1H), 1.89 (ddd, J = 14.8, 8.8, 6.2 Hz, 1H), 1.23 (s, 6H), 1.20 (s, 6H), 1.19 (s, 9H).

^{13}C NMR (100 MHz, Chloroform-*d*) δ 177.5, 136.3, 128.7, 128.3, 127.8, 125.0 (q, J = 279.2 Hz), 83.6, 60.4, 39.4 (q, J = 27.2 Hz), 37.6, 31.1, 26.2, 23.9, 23.6. The carbon attached to the boron was not found due to quadrupolar relaxation.

^{11}B NMR (128 MHz, Chloroform-*d*) δ 31.7.

^{19}F NMR (376 MHz, Chloroform-*d*) δ -59.91 (t, J = 11 Hz).

HRMS (ESI) calcd for $C_{22}H_{32}BF_3NaO_4S$ $[M+Na]^+$: 483.1964; found: 483.1972

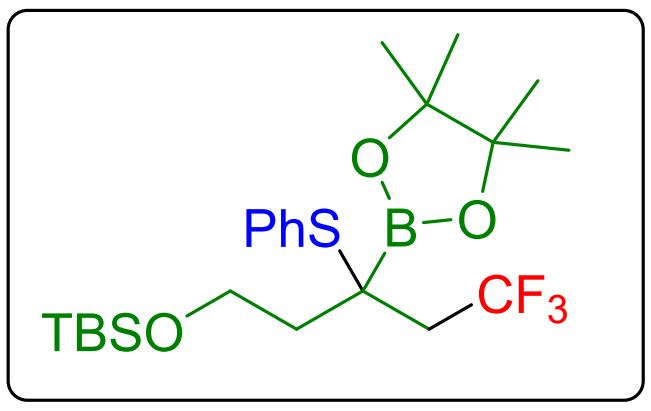

***tert*-butyldimethyl((5,5,5-trifluoro-3-(phenylthio)-3-(4,4,5,5-tetramethyl-1,3,2-dioxaborolan-2-yl)pentyl)oxy)silane (3pa)**

The product **3oa** was synthesized following General Procedure for Three-Component Conjunctive Coupling 2 (GP2) using thiophenol. **3oa** was isolated by flash column chromatography (200-400 mesh silica gel) using MTBE : hexane (0:100 → 1:99) as colourless oil (74.5 mg, 76%).

^{1}H NMR (400 MHz, Chloroform-*d*) δ 7.51 – 7.46 (m, 2H), 7.32 – 7.20 (m, 3H), 3.93 (dtd, J = 32.4, 9.8, 5.4 Hz, 2H), 2.38 (ddq, J = 70.9, 15.1, 11.0 Hz, 2H), 1.87 (dddd, J = 78.9, 14.7, 9.7, 5.4 Hz, 2H), 1.16 (s, 6H), 1.14 (s, 6H), 0.83 (s, 9H), 0.00 (s, 6H).

^{13}C NMR (100 MHz, Chloroform-*d*) δ 137.2, 130.4, 129.1, 128.8, 125.0 (q, J = 279.1 Hz), 84.5, 60.2, 40.7 (q, J = 26.9 Hz), 36.9, 26.1, 24.9, 24.8, 18.4, -5.1. The carbon attached to the boron was not found due to quadrupolar relaxation.

^{11}B NMR (128 MHz, Chloroform-*d*) δ 31.6.

^{19}F NMR (376 MHz, Chloroform-*d*) δ -59.4 (t, J = 11 Hz).

HRMS (ESI) calcd for $C_{23}H_{38}BF_3NaO_3SSi$ $[M+Na]^+$: 513.2254; found: 513.2270

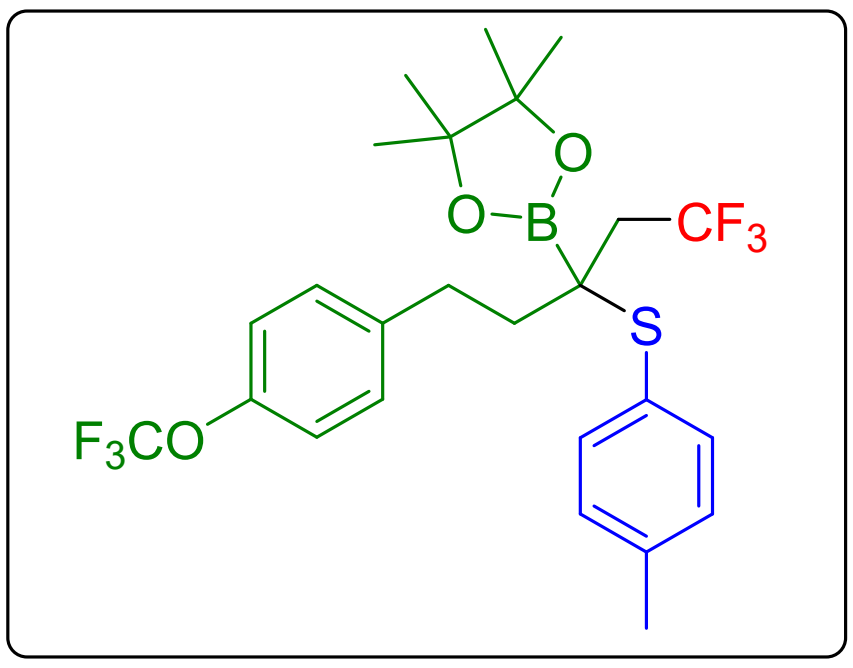

4,4,5,5-tetramethyl-2-(1,1,1-trifluoro-3-((p-tolylthio)methyl)-5-(4-(trifluoromethoxy)phenyl)pentan-3-yl)-1,3,2-dioxaborolane (5a)

The product **5a** was synthesized following General Procedure for Three-Component Conjunctive Coupling 1 (GP1) using p-Tolyl disulfide (0.75 eq., 0.15 mmol, 36.9 mg). **5a** was isolated by flash column chromatography (200-400 mesh silica gel) using MTBE : hexane (0:100 → 1:99) as white solid (72.7 mg, 68%).

^{1}H NMR (400 MHz, Chloroform-*d*) δ7.49 – 7.47 (m, 2H), 7.18 – 7.09 (m, 6H), 3.01 – 2.86 (m, 2H), 2.57 – 2.40 (m, 2H), 2.36 (s, 3H), 1.98 (ddd, *J* = 16.8, 12.3, 4.9 Hz, 1H), 1.83 (ddd, *J* = 14.5, 12.1, 5.1 Hz, 1H), 1.26 (s, 6H), 1.24 (s, 6H).

^{13}C NMR (125 MHz, Chloroform-*d*) δ 147.5, 141.2, 139.6, 137.2, 129.9, 129.7, 126.9, 126.4 (q, *J* = 279 Hz), 121.0, 120.7 (q, *J* = 256.2 Hz), 84.6, 39.8 (q, *J* = 26.9 Hz), 35.9, 30.1, 25.1, 24.9, 21.4. The carbon attached to the boron was not found due to quadrupolar relaxation.

^{11}B NMR (128 MHz, Chloroform-*d*) δ 31.9

^{19}F NMR (376 MHz, Chloroform-*d*) δ -57.9 (s, 3F), -59.93 (t, *J* = 10.9 Hz, 3F).

HRMS (ESI) calcd for $C_{25}H_{30}BF_6O_3S$ $[M+H]^+$: 535.1913; found: 535.1933.

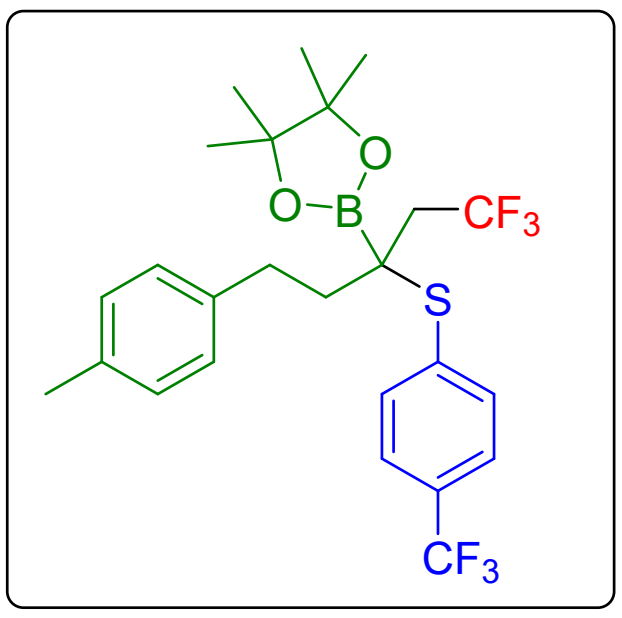

4,4,5,5-tetramethyl-2-(1,1,1-trifluoro-5-(p-tolyl)-3-((4-(trifluoromethyl)phenyl)thio)pentan-3-yl)-1,3,2-dioxaborolane (5b)

The product **5b** was synthesized following General Procedure for Three-Component Conjunctive Coupling 2 (GP2) using 4-(trifluoromethyl)benzenethiol (1.5 eq., 0.3 mmol, 53.4 mg). **5b** was isolated by flash column chromatography (200-400 mesh silica gel) using MTBE : hexane (0:100 → 1:99) as white solid (84 mg, 81%).

^{1}H NMR (400 MHz, Chloroform-*d*) δ 7.74 (d, *J* = 8.1 Hz, 2H), 7.59 (d, *J* = 8.1 Hz, 2H), 7.09 (d, *J* = 7.8 Hz, 2H), 7.01 (d, *J* = 7.9 Hz, 2H), 2.90 – 2.80 (m, 2H), 2.65 – 2.46 (m, 2H), 2.32 (s, 2H), 2.12 – 2.02 (m, 1H), 1.96 – 1.88 (m, 1H), 1.25 (s, 6H), 1.24 (s, 6H).

^{13}C NMR (100 MHz, Chloroform-*d*) δ 138.8, 136.5, 136.2, 135.6, 130.8 (q, *J* = 32.7 Hz), 129.3, 128.4, 126.2 (q, *J* = 279 Hz), 125.6 (q, *J* = 3.6 Hz), 124.1 (q, *J* = 272.2 Hz), 84.8, 39.5 (q, *J* = 27.1 Hz), 37.5 (br), 36.5, 30.4, 25.0, 24.9, 21.1.

^{11}B NMR (128 MHz, Chloroform-*d*) δ 31.9

^{19}F NMR (376 MHz, Chloroform-*d*) δ -59.78 (t, *J* = 10.7 Hz, 3F), -62.77 (s, 3F).

HRMS (ESI) calcd for $C_{25}H_{29}BF_6NaO_2S$ $[M+Na]^+$: 541.1783; found: 541.1791.

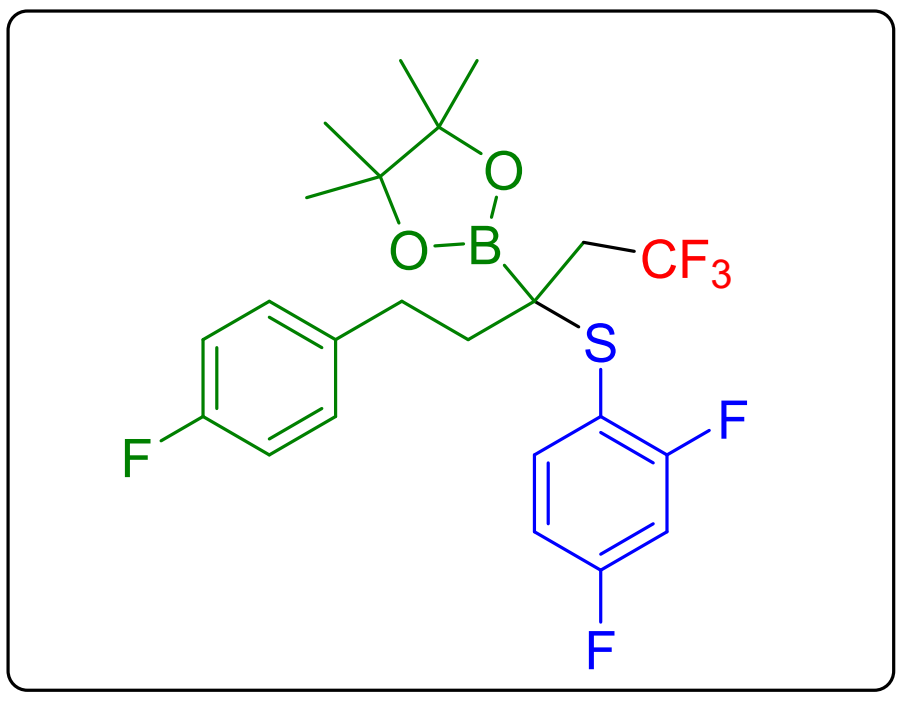

2-(3-((2,4-difluorophenyl)thio)-1,1,1-trifluoro-5-(4-fluorophenyl)pentan-3-yl)-4,4,5,5-tetramethyl-1,3,2-dioxaborolane (5c)

The product **5c** was synthesized following General Procedure for Three-Component Conjunctive Coupling 2 (GP2) using 2,4-difluorobenzenethiol (1.5 eq., 0.3 mmol, 43.8 mg). **5c** was isolated by flash column chromatography (200-400 mesh silica gel) using MTBE : hexane (0:100 → 1:99) as white solid (62.7 mg, 64%).

^{1}H NMR (400 MHz, Chloroform-*d*) δ 7.60 (td, *J* = 9.1, 8.6, 6.5 Hz, 1H), 7.16 – 7.11 (m, 2H), 6.99 – 6.92 (m, 2H), 6.88 (ddt, *J* = 10.9, 8.1, 3.0 Hz, 2H), 2.91 (dtd, *J* = 48.9, 13.0, 4.7 Hz, 2H), 2.53 (qd, *J* = 10.9, 4.7 Hz, 2H), 2.02 (td, *J* = 13.9, 12.9, 4.6 Hz, 1H), 1.88 (td, *J* = 15.0, 14.0, 4.8 Hz, 1H), 1.23 (s, 6H), 1.21 (s, 6H).

^{13}C NMR (100 MHz, Chloroform-*d*) δ 164.8 (dd, *J* = 249.6, 12.7 Hz), 164.2 (dd, *J* = 253.6 Hz. 11.6 Hz), 161.3 (d, *J* = 243.3 Hz), 141.0 (d, *J* = 9.6 Hz), 137.7 (d, *J* = 3.2 Hz), 129.7 (d, *J* = 7.9 Hz), 126.1 (q, *J* = 279.2), 115.1 (d, *J* = 21.1 Hz), 113.4 (dd, *J* = 19.3, 4.1 Hz), 111.8 (dd, *J* = 21.2, 3.9 Hz), 104.6 (dd, *J* = 28.3, 25.6 Hz), 84.5, 39.5 (q, *J* = 27.4 Hz), 36.6, 29.7, 25.1, 24.6.

^{11}B NMR (128 MHz, Chloroform-*d*) δ 31.3

^{19}F NMR (376 MHz, Chloroform-*d*) δ -60.09 (t, *J* = 10.9 Hz, 3F), -97.28 (q, *J* = 9.2 Hz, 1F), -105.99 (quint, *J* = 8.3 Hz, 1F), -117.75 (sep, *J* = 4.8 Hz, 1F).

HRMS (ESI) calcd for $C_{25}H_{29}BF_6NaO_2S$ $[M+Na]^+$: 513.1470; found: 513.1464.

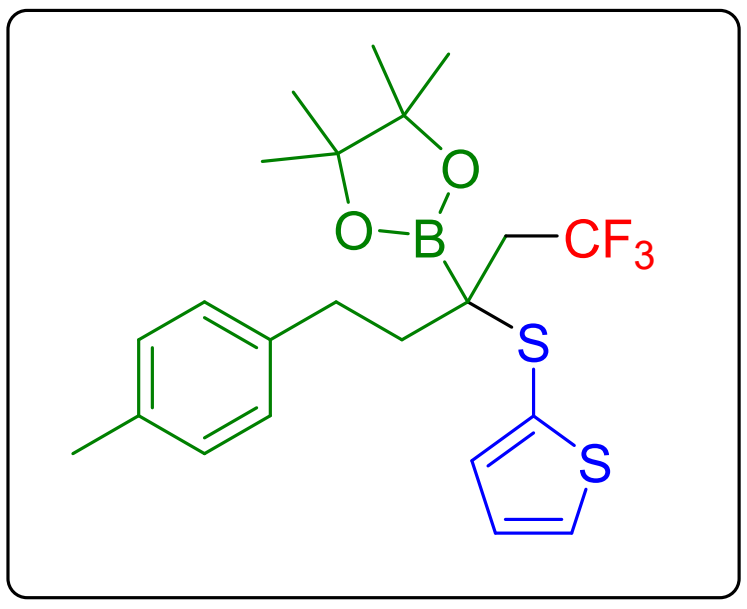

4,4,5,5-tetramethyl-2-(1,1,1-trifluoro-3-(thiophen-2-ylthio)-5-(p-tolyl)pentan-3-yl)-1,3,2-dioxaborolane (5d)

The product **5d** was synthesized following General Procedure for Three-Component Conjunctive Coupling 1 (GP1) using 1,2-di(thiophen-2-yl)disulfane (0.75 eq., 0.15 mmol, 34.5 mg). **5d** was isolated by flash column chromatography (200-400 mesh silica gel) using MTBE : hexane (0:100 → 1:99) as colourless solid (55 mg, 60%).

^{1}H NMR (400 MHz, Chloroform-*d*) δ 7.38 (dd, *J* = 5.4, 1.1 Hz, 1H), 7.19 (dd, *J* = 3.6 Hz, 1.1 Hz, 1H), 7.04 – 7.0 (m, 4H), 6.97 (dd, *J* = 5.4 Hz, 3.6 Hz, 1H), 2.94 (td, *J* = 13.0 Hz, 4.6 Hz, 1H), 2.72 (td, *J* = 13.1, 4.2 Hz, 1H), 2.51 - 2.30 (m, 2H), 2.24 (s, 3H), 1.88 (td, *J* = 13.5 Hz, 4.2 Hz, 1H), 1.7 (td, *J* = 13.6 Hz, 4.7 Hz, 1H), 1.22 (s, 6H), 1.20 (s, 6H).

^{13}C NMR (125 MHz, Chloroform-*d*) δ 139.0, 138.1, 135.5, 131.6, 129.2, 128.6, 128.5, 127.7, 126.3 (q, *J* = 278.3 Hz), 84.8, 40.2 (q, *J* = 26.9 Hz), 37.0, 31.1, 25.1, 24.9, 21.1

^{11}B NMR (128 MHz, Chloroform-*d*) δ 31.9

^{19}F NMR (376 MHz, Chloroform-*d*) δ -59.58 (t, *J* = 10.7 Hz).

HRMS (ESI) calcd for $C_{22}H_{29}BF_3O_2S_2$ $[M+H]^+$: 457.1654; found: 457.1687.

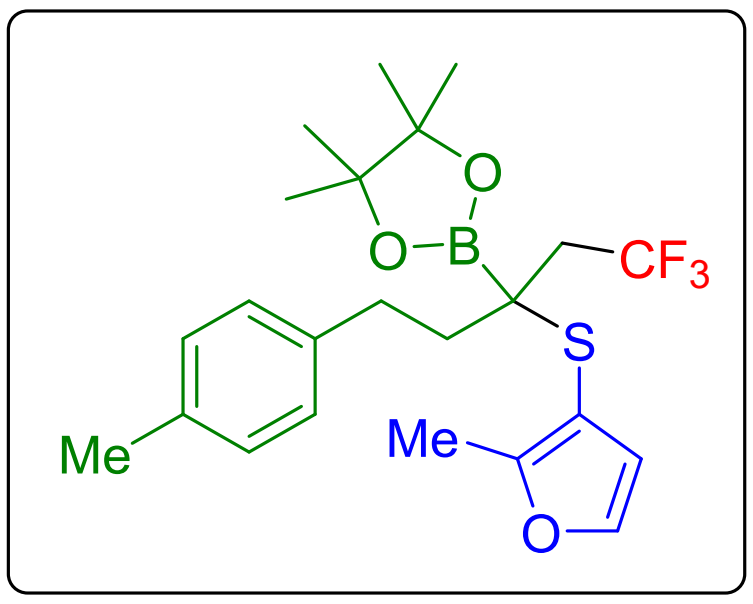

4,4,5,5-tetramethyl-2-(1,1,1-trifluoro-3-((2-methylfuran-3-yl)thio)-5-(p-tolyl)pentan-3-yl)-1,3,2-dioxaborolane (5e)

The product **5e** was synthesized following General Procedure for Three-Component Conjunctive Coupling 1 (GP1) using 1,2-bis(2-methylfuran-3-yl)disulfane (0.75 eq., 0.15 mmol, 33.9 mg). **5e** was isolated by flash column chromatography (200-400 mesh silica gel) using MTBE : hexane (0:100 → 1:99) as white solid (31.8 mg, 35%).

^{1}H NMR (400 MHz, Chloroform-*d*) δ 7.29 (d, J = 1.9 Hz, 1H), 7.10 – 7.05 (m, 4H), 6.43 (d, J = 1.9 Hz, 1H), 2.89 – 2.82 (m, 2H), 2.54 – 2.42 (m, 2H), 2.39 (s, 3H), 2.31 (s, 3H), 2.02 – 1.93 (m, 1H), 1.89 – 1.81 (m, 1H), 1.26 (s, 6H), 1.24 (s, 6H).

^{13}C NMR (100 MHz, Chloroform-*d*) δ 156.7, 139.5, 138.0, 134.3, 128.1, 127.3, 125.3 (q, J = 279.0 Hz), 115.5, 105.5, 83.3, 38.5 (q, J = 27.2 Hz), 35.4, 29.3, 23.8, 23.6, 19.9, 11.3.

^{11}B NMR (128 MHz, Chloroform-*d*) δ 32.2

^{19}F NMR (376 MHz, Chloroform-*d*) δ -59.9 (t, J = 10.9 Hz).

HRMS (ESI) calcd for $C_{23}H_{30}BF_{3}NaO_{3}S$ $[M+Na]^{+}$: 477.1858; found: 477.1853.

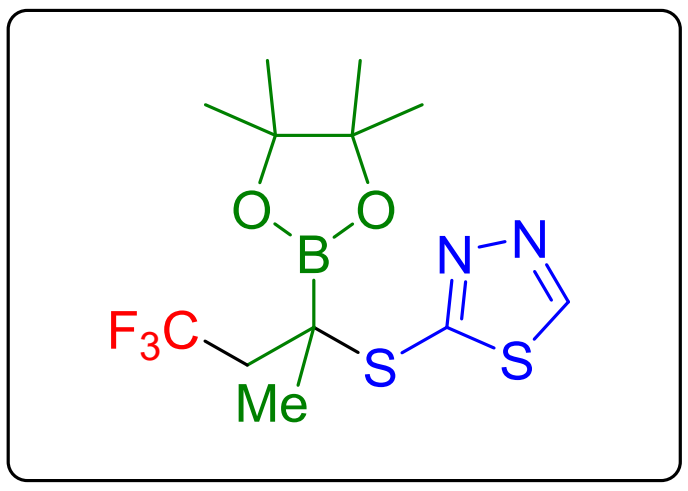

2-((4,4,4-trifluoro-2-(4,4,5,5-tetramethyl-1,3,2-dioxaborolan-2-yl)butan-2-yl)thio)-1,3,4-thiadiazole (5f)

The product **5f** was synthesized following the procedure mentioned below: In the solution of 1,3,4-thiadiazole-2-thiol (1.5 eq., 0.3 mmol, 35.45 mg) in 0.2 ml DMF was added 0.3 ml BEt_3 (1M solution in THF) at -60 ºC and stirred for 40 mins. **1a** (1 eq., 0.2 mmol, 33.6 mg) in 0.5 ml DMF was added to the reaction at -60 ºC; Stirred for 1h; Reaction mixture transferred to a

0.2 ml DMF solution of Togni-II (1.5eq., 0.3 mmol, 95 mg) at -60 ºC; Stirred for 2 h at -60 ºC and stirred at RT for another 8 h. **5f** was isolated by flash column chromatography (200-400 mesh silica gel) using ethyl acetate : hexane (20:80 → 50:50) as yellow oil (26.2 mg, 37%).

^{1}H NMR (400 MHz, Chloroform-*d*) δ 8.91 (s, 1H), 2.81 (qd, *J* = 11.4, 9.0 Hz, 2H), 1.62 (s, 3H), 1.28 (s, 6H), 1.27 (s, 6H).

^{13}C NMR (100 MHz, Chloroform-*d*) δ 151.9, 141.3, 127.4 (q, *J* = 277.3 Hz), 82.4, 41.4 (q, *J* = 26.6 Hz), 25.9, 25.6, 22.7 The carbon attached to the boron was not found due to quadrupolar relaxation.

^{11}B NMR (128 MHz, Chloroform-*d*) δ 17.1

^{19}F NMR (376 MHz, Chloroform-*d*) δ -59.9 (t, *J* = 11.3 Hz).

HRMS (ESI) calcd for $C_{12}H_{18}BF_3N_2NaO_2S_2$ $[M+Na]^+$: 377.0753; found: 377.0749.

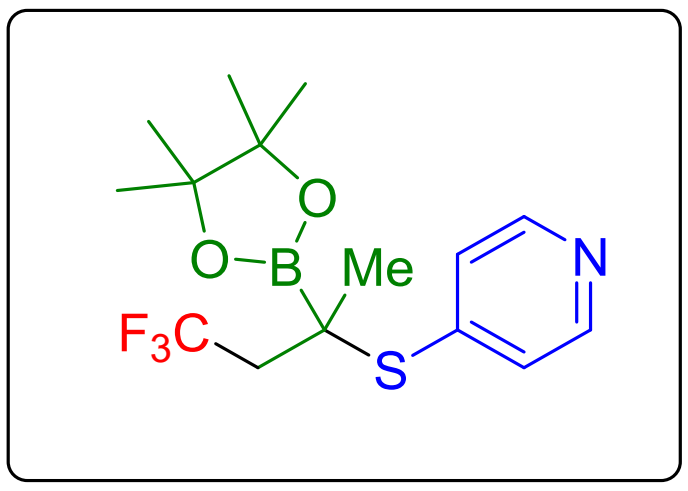

4-((4,4,4-trifluoro-2-(4,4,5,5-tetramethyl-1,3,2-dioxaborolan-2-yl)butan-2-yl)thio)pyridine (5g)

The product **5g** was synthesized following General Procedure for Three-Component Conjunctive Coupling 1 (GP1) using 1,2-di(pyridin-4-yl)disulfane (0.75 eq., 0.15 mmol, 33.05 mg). **5g** was isolated by flash column chromatography (200-400 mesh silica gel) using ethyl acetate : hexane (5:95 → 20:80) as colorless oil (21.5 mg, 31%).

^{1}H NMR (400 MHz, Chloroform-*d*) δ 8.49 (d, *J* = 5.8 Hz, 2H), 7.42 (d, *J* = 5.9 Hz, 2H), 2.78 (dq, *J* = 14.7, 11.3 Hz, 1H), 2.34 (dq, *J* = 14.7, 10.6 Hz, 1H), 1.48 (s, 3H), 1.23 (s, 12H).

^{13}C NMR (100 MHz, Chloroform-*d*) δ 149.2, 144.5, 128.7 (q, *J* = 281.4 Hz), 127.0, 84.9, 41.9 (q, *J* = 27.6 Hz), 24.7, 24.6, 21.9 The carbon attached to the boron was not found due to quadrupolar relaxation.

^{11}B NMR (128 MHz, Chloroform-*d*) δ 31.9

^{19}F NMR (376 MHz, Chloroform-*d*) δ -60.19 (t, *J* = 10.9 Hz).

HRMS (ESI) calcd for $C_{15}H_{22}BF_3NO_2S$ $[M+H]^+$: 348.1416; found: 348.1433.

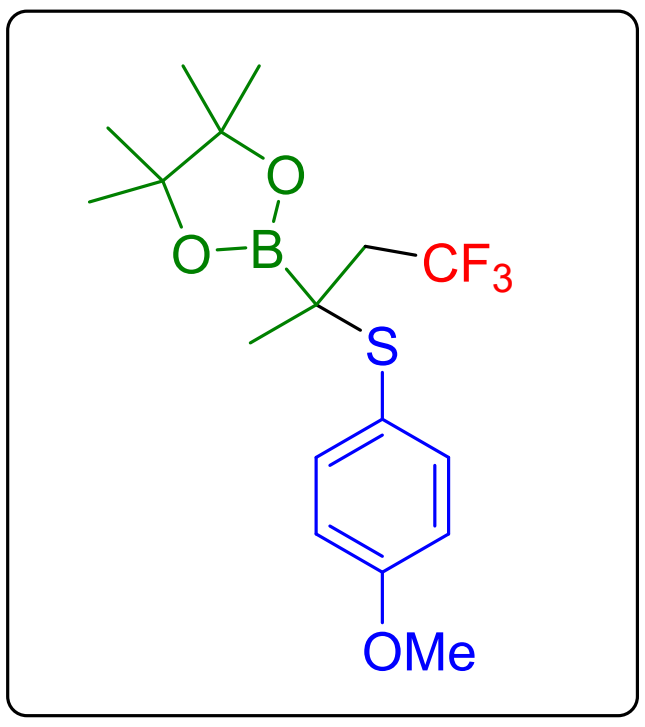

4,4,5,5-tetramethyl-2-(4,4,4-trifluoro-2-((4-methoxyphenyl)thio)butan-2-yl)-1,3,2-dioxaborolane (5h)

The product **5h** was synthesized following General Procedure for Three-Component Conjunctive Coupling 1 (GP1) using 1,2-bis(4-methoxyphenyl)disulfane (0.75 eq., 0.15 mmol, 41.7 mg). **5h** was isolated by flash column chromatography (200-400 mesh silica gel) using MTBE : hexane (0:100 → 5:95) as colourless oil (63.9 mg, 85%).

^{1}H NMR (400 MHz, Chloroform-*d*) δ 7.47 – 7.43 (m, 2H), 6.87 – 6.83 (m, 2H), 3.81 (s, 3H), 2.69 – 2.57 (m 1H), 2.24 – 2.13 (m, 1H), 1.28 (d, *J* = 1.8 Hz, 3H), 1.27 (s, 6H), 1.25 (s, 6H).

^{13}C NMR (125 MHz, Chloroform-*d*) δ 160.7, 139.2, 126.3 (q, *J* = 278.2 Hz), 120.7, 114.2, 84.3, 55.3, 42.5 (q, *J* = 26.9 Hz), 24.8, 24.6, 21.9. The carbon attached to the boron was not found due to quadrupolar relaxation.

^{11}B NMR (128 MHz, Chloroform-*d*) δ 32.1

^{19}F NMR (376 MHz, Chloroform-*d*) δ -60.6 (t, *J* = 11.8 Hz).

HRMS (ESI) calcd for $C_{17}H_{24}BF_3NaO_3S$ $[M+Na]^+$: 399.1389; found: 399.1385.

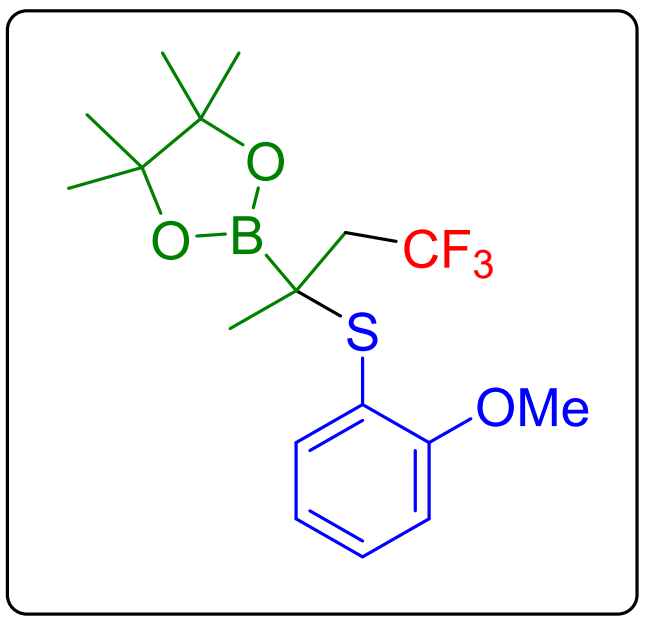

4,4,5,5-tetramethyl-2-(4,4,4-trifluoro-2-((2-methoxyphenyl)thio)butan-2-yl)-1,3,2-dioxaborolane (5i)

The product **5i** was synthesized following General Procedure for Three-Component Conjunctive Coupling 2 (GP2) using 2-methoxybenzenethiol (1.5 eq., 0.3 mmol, 42.06 mg). **5i** was isolated by flash column chromatography (200-400 mesh silica gel) using MTBE : hexane (0:100 → 5:95) as white solid (39.1 mg, 52%).

^{1}H NMR (400 MHz, Chloroform-*d*) δ 7.53 (dt, *J* = 7.7, 2.2 Hz, 1H), 7.40 – 7.32 (m, 1H), 6.91 (dt, *J* = 7.2, 3.4 Hz, 2H), 3.86 (s, 3H), 2.78 (m, 1H), 2.46 – 2.30 (m, 1H), 1.33 (s, 3H), 1.24 (s, 12H).

^{13}C NMR (100 MHz, Chloroform-*d*) δ 161.3, 140.1, 131.2, 126.3 (q, *J* = 279.5 Hz), 120.6, 118.5, 111.1, 84.3, 55.6, 42.7 (q, *J* = 27.1 Hz), 24.8, 24.6, 21.7. The carbon attached to the boron was not found due to quadrupolar relaxation.

^{11}B NMR (128 MHz, Chloroform-*d*) δ 31.9

^{19}F NMR (376 MHz, Chloroform-*d*) δ -60.77 (t, *J* = 11.4 Hz).

HRMS (ESI) calcd for $C_{17}H_{24}BF_3NaO_3S$ $[M+Na]^+$: 399.1389; found: 399.1392.

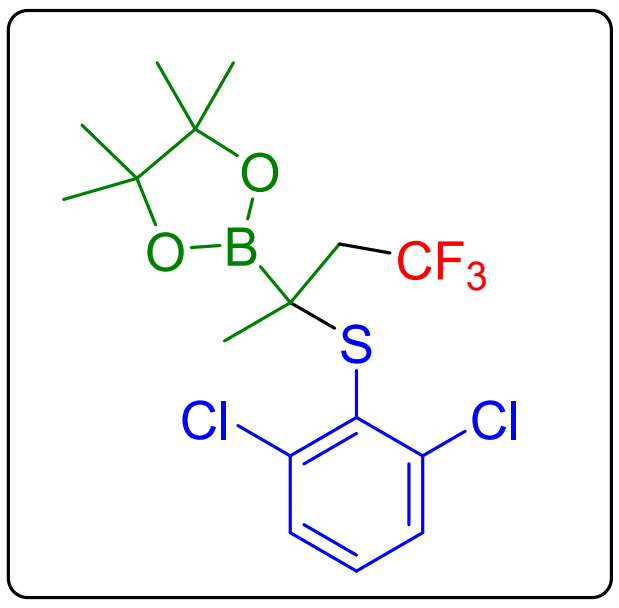

2-(2-((2,6-dichlorophenyl)thio)-4,4,4-trifluorobutan-2-yl)-4,4,5,5-tetramethyl-1,3,2-dioxaborolane (5j)

The product **5j** was synthesized following General Procedure for Three-Component Conjunctive Coupling 2 (GP2) using 2,6-dichlorobenzenethiol (1.5 eq., 0.3 mmol, 53.7 mg). **5j** was isolated by flash column chromatography (200-400 mesh silica gel) using MTBE : hexane (0:100 → 5:95) as white solid (58.1 mg, 70%).

^{1}H NMR (400 MHz, Chloroform-*d*) δ 7.35 (d, *J* = 8.0 Hz, 1H), 7.20 – 7.10 (m, 2H), 2.92 (dq, *J* = 14.8, 11.8 Hz, 1H), 2.66 (dq, *J* = 14.8, 10.7 Hz, 1H), 1.20 (s, 15H).

^{13}C NMR (125 MHz, Chloroform-*d*) δ 144.4, 131.1, 130.3, 128.8, 126.0 (q, *J* = 279.4 Hz), 84.7, 55.6, 44.3 (q, *J* = 26.6 Hz), 35.3 (br), 24.8, 20.8. The carbon attached to the boron was not found due to quadrupolar relaxation.

^{11}B NMR (160 MHz, Chloroform-*d*) δ 31.4

^{19}F NMR (376 MHz, Chloroform-*d*) δ -61.07 (t, *J* = 11.0 Hz).

HRMS (ESI) calcd for $C_{16}H_{20}BCl_2F_3NaO_2S$ $[M+Na]^+$: 437.0504; found: 437.0489.

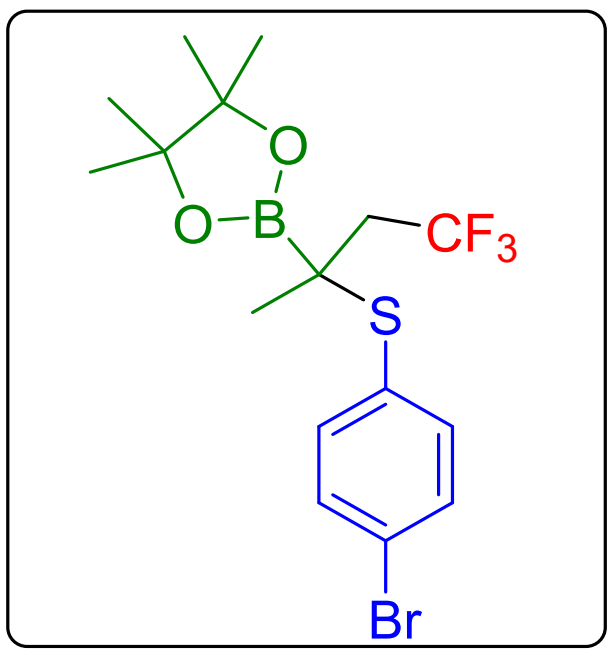

2-(2-((4-bromophenyl)thio)-4,4,4-trifluorobutan-2-yl)-4,4,5,5-tetramethyl-1,3,2-dioxaborolane (5k)

The product **5k** was synthesized following General Procedure for Three-Component Conjunctive Coupling 1 (GP1) using 1,2-bis(4-bromophenyl)disulfane (0.75 eq., 0.15 mmol, 56.4 mg). **5k** was isolated by flash column chromatography (200-400 mesh silica gel) using MTBE : hexane (0:100 → 1:99) as colourless oil (54.4 mg, 64%).

^{1}H NMR (400 MHz, Chloroform-*d*) δ 7.46 (d, *J* = 8.3 Hz, 2H), 7.40 (d, *J* = 8.4 Hz, 2H), 2.64 (dq, *J* = 14.6, 11.5 Hz, 1H), 2.21 (dq, *J* = 14.6, 10.6 Hz, 1H), 1.34 (s, 3H), 1.26 (s, 6H), 1.24 (s, 6H).

^{13}C NMR (100 MHz, Chloroform-*d*) δ 138.7, 131.9, 129.5, 126.1 (q, *J* = 279.3 Hz), 124.0, 84.5, 42.5 (q, *J* = 27.1 Hz), 24.8, 24.5, 22.1

^{11}B NMR (128 MHz, Chloroform-*d*) δ 32.0

^{19}F NMR (376 MHz, Chloroform-*d*) δ -60.56 (t, *J* = 11.1 Hz).

HRMS (ESI) calcd for $C_{16}H_{22}BBrF_3O_2S$ $[M+H]^+$: 425.0569; found: 425.0579.

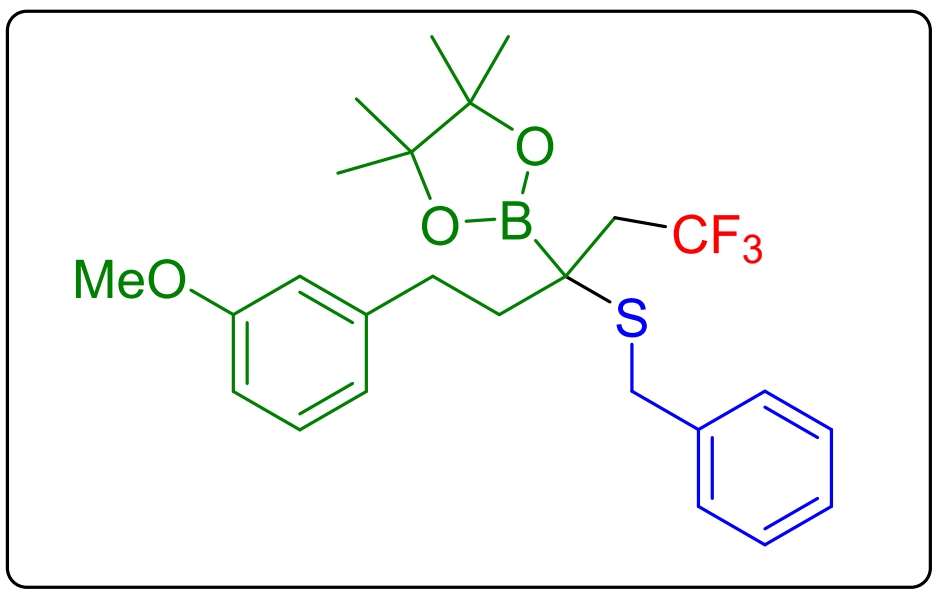

2-(3-(benzylthio)-1,1,1-trifluoro-5-(3-methoxyphenyl)pentan-3-yl)-4,4,5,5-tetramethyl-1,3,2-dioxaborolane (5l)

The product **5l** was synthesized following General Procedure for Three-Component Conjunctive Coupling 1 (GP1) using 1,2-dibenzyldisulfane (0.75 eq., 0.15 mmol, 36.9 mg). **5l** was isolated by flash column chromatography (200-400 mesh silica gel) using MTBE : hexane (0:100 → 5:95) as white solid (52.8 mg, 55%).

^{1}H NMR (400 MHz, Chloroform-*d*) δ 7.36 – 7.29 (m, 4H), 7.26 – 7.24 (m, 1H), 7.18 (t, J = 7.6 Hz, 1H), 6.75 – 6.71 (m, 3H), 3.87 (d, J = 11.5 Hz, 1H), 3.79 (m, 4H), 2.83 (ddd, J = 13.1, 10.7, 6.3 Hz, 1H), 2.70 (ddd, J = 13.1, 9.7, 5.8 Hz, 2H), 2.62 – 2.42 (m, 1H), 2.05 – 2.01 (m, 2H), 1.30 (s, 12H).

^{13}C NMR (125 MHz, Chloroform-*d*) δ 159.7, 143.9, 137.2, 129.3, 129.2, 128.6, 127.2, 126.3 (q, J = 279.1 Hz), 120.9, 114.4, 111.1, 84.4, 55.2, 38.4 (q, J = 26.9 Hz), 34.9, 33.2, 30.3, 24.8, 24.7. The carbon attached to the boron was not found due to quadrupolar relaxation.

^{11}B NMR (128 MHz, Chloroform-*d*) δ 31.4

^{19}F NMR (376 MHz, Chloroform-*d*) δ -60.46 (t, J = 10.9 Hz).

HRMS (ESI) calcd for $C_{25}H_{32}BF_3NaO_3S$ $[M+Na]^+$: 503.2015; found: 503.2009

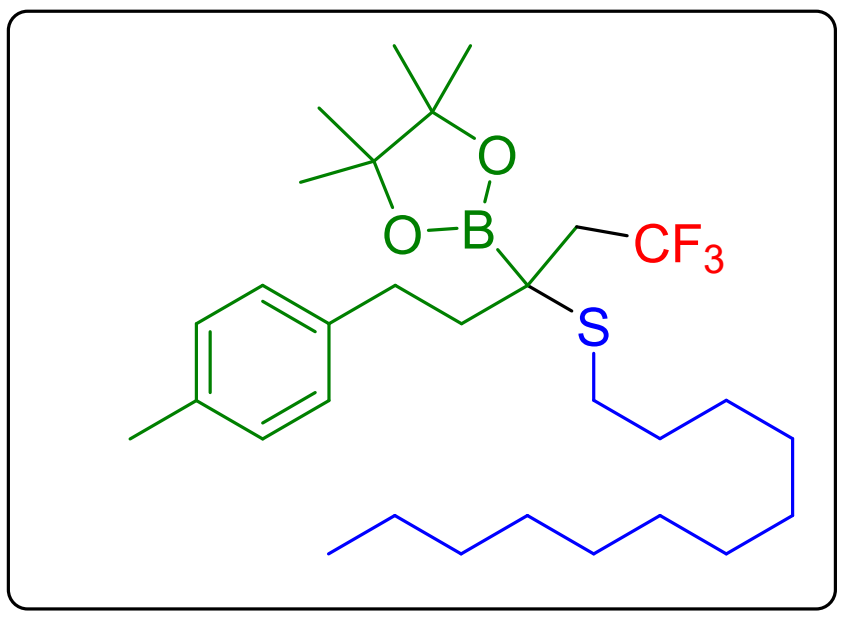

2-(3-(dodecylthio)-1,1,1-trifluoro-5-(3-methoxyphenyl)pentan-3-yl)-4,4,5,5-tetramethyl-1,3,2-dioxaborolane (5m)

The product **5m** was synthesized following General Procedure for Three-Component Conjunctive Coupling 2 (GP2) using dodecane-1-thiol (1.5 eq., 0.3 mmol, 60.7 mg). **5m** was isolated by flash column chromatography (200-400 mesh silica gel) using MTBE : hexane (0:100 → 1:99) as white solid (51 mg, 47%).

^{1}H NMR (400 MHz, Chloroform-*d*) δ 7.08 (s, 4H), 2.92 – 2.76 (m, 1H), 2.73 – 2.41 (m, 5H), 2.31 (s, 3H), 1.96 (dd, *J* = 10.0, 7.3 Hz, 2H), 1.56 (m, 3H), 1.39 (p, *J* = 6.6 Hz, 2H), 1.34 – 1.16 (m, 27H), 0.88 (t, *J* = 6.8 Hz, 3H).

^{13}C NMR (125 MHz, Chloroform-*d*) δ 139.4, 135.3, 129.2, 128.5, 126.4 (q, *J* = 279.1 Hz), 84.2, 38.3 (q, *J* = 26.6 Hz), 35.1, 32.1, 29.8, 29.8, 29.8, 29.7, 29.6, 29.5, 29.4, 29.3, 29.3, 28.2, 24.8, 24.7, 22.8, 21.1, 14.3. The carbon attached to the boron was not found due to quadrupolar relaxation.

^{11}B NMR (128 MHz, Chloroform-*d*) δ 32.2

^{19}F NMR (376 MHz, Chloroform-*d*) δ -60.48 (t, *J* = 11.00 Hz).

HRMS (ESI) calcd for $C_{30}H_{50}BF_3NaO_2S$ $[M+Na]^+$: 543.3655; found: 543.3652

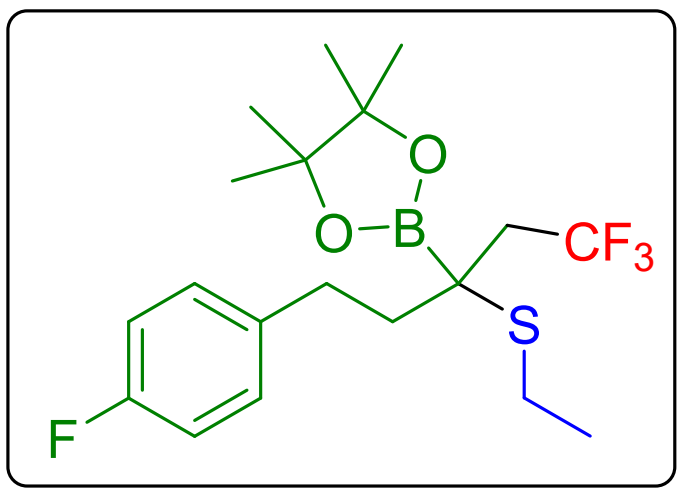

2-(3-(ethylthio)-1,1,1-trifluoro-5-(4-fluorophenyl)pentan-3-yl)-4,4,5,5-tetramethyl-1,3,2-dioxaborolane (5n)

The product **5n** was synthesized following General Procedure for Three-Component Conjunctive Coupling 1 (GP1) using diethyl disulfide (0.75 eq., 0.15 mmol, 18.3 mg). **5n** was isolated by flash column chromatography (200-400 mesh silica gel) using MTBE : hexane (0:100 → 1:99) as colourless oil (37.4 mg, 46%).

^{1}H NMR (400 MHz, Chloroform-*d*) δ 7.14 – 7.11 (m, 2H), 6.96 – 6.93 (m, 2H), 2.86 – 2.41 (m, 8H), 1.95 (dd, *J* = 9.7, 7.6 Hz, 2H). 1.26 – 1.22 (m, 15 H).

^{13}C NMR (100 MHz, Chloroform-*d*) δ 161.3 (d, *J* = 242.5 Hz), 137.9 (d, *J* = 3.6 Hz), 129.8 (d, *J* = 7.3 Hz), 126.3 (q, *J* = 279.1), 115.1 (d, *J* = 20.9 Hz), 84.2, 38.1 (q, *J* = 26.9 Hz), 34.9,

29.2, 24.8, 24.6, 24.6, 22.2, 14.1 The carbon attached to the boron was not found due to quadrupolar relaxation.

^{11}B NMR (128 MHz, Chloroform-*d*) δ 31.3

^{19}F NMR (376 MHz, Chloroform-*d*) δ -60.56 (t, *J* = 10.4 Hz), -117.89 (sep, *J* = 5.2 Hz).

HRMS (ESI) calcd for $C_{19}H_{28}BF_4O_2S$ $[M+H]^+$: 407.1839; found: 407.1845.

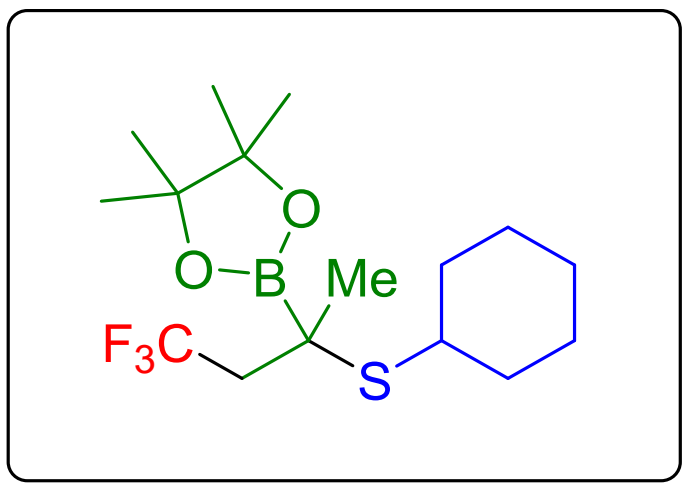

2-(2-(cyclohexylthio)-4,4,4-trifluorobutan-2-yl)-4,4,5,5-tetramethyl-1,3,2-dioxaborolane (5o)

The product **5o** was synthesized following General Procedure for Three-Component Conjunctive Coupling 2 (GP2) using cyclohexanethiol (1.5 eq., 0.3 mmol, 34.9 mg). **5o** was isolated by flash column chromatography (200-400 mesh silica gel) using ethyl acetate : hexane (0:100 → 1:99) as colourless oil (42.9 mg, 61%).

^{1}H NMR (400 MHz, Chloroform-*d*) δ 2.88 – 2.82 (m, 1H), 2.73 (dq, *J* = 14.6, 11.7 Hz, 1H), 2.25 (dq, *J* = 14.6, 10.6 Hz, 1H), 1.97 – 1.88 (m, 2H). 1.74 – 1.67 (m, 2H), 1.57 – 1.52 (m, 1H), 1.42 – 1.31 (m, 7H), 1.26 (s, 6H), 1.25 (s, 6H), 1.22 – 1.15 (m, 1H).

^{13}C NMR (100 MHz, Chloroform-*d*) δ 126.1 (q, *J* = 279.4), 84.1, 42.9 (q, *J* = 26.6 Hz), 41.7, 36.5, 35.9, 26.4, 26.3, 25.4, 24.8, 24.6, 21.6 The carbon attached to the boron was not found due to quadrupolar relaxation.

^{11}B NMR (128 MHz, Chloroform-*d*) δ 31.6

^{19}F NMR (376 MHz, Chloroform-*d*) δ -60.56 (t, *J* = 11.2 Hz).

HRMS (ESI) calcd for $C_{16}H_{29}BF_3O_2S$ $[M+H]^+$: 353.1933; found: 353.1949.

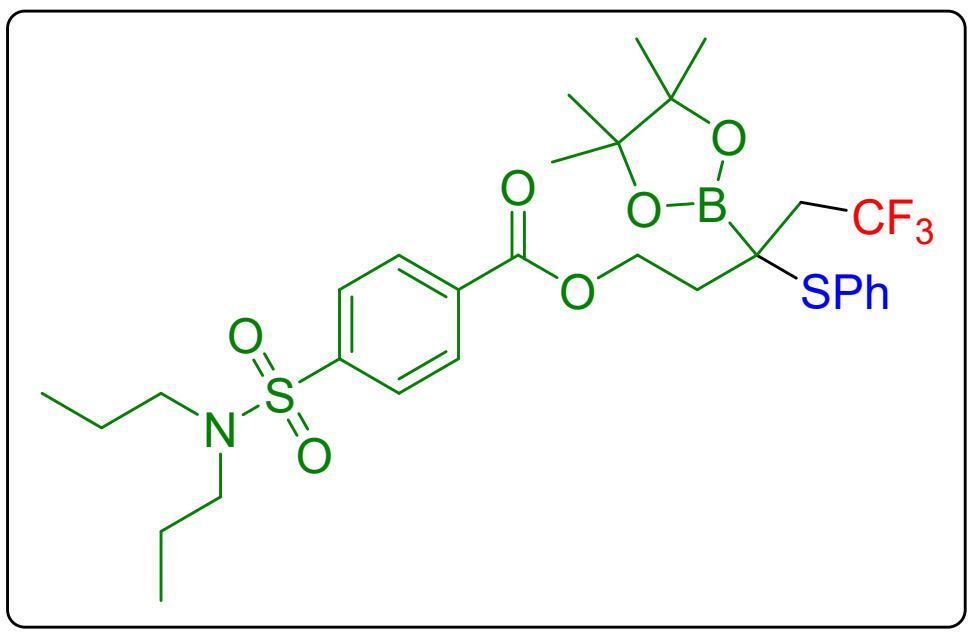

5,5,5-trifluoro-3-(phenylthio)-3-(4,4,5,5-tetramethyl-1,3,2-dioxaborolan-2-yl)pentyl 4-(N,N-dipropylsulfamoyl)benzoate (3pa)

The product **3pa** was synthesized following General Procedure for Three-Component Conjunctive Coupling 2 (GP2) using thiophenol (1.5 eq., 0.3 mmol, 33 mg). **3pa** was isolated by flash column chromatography (200-400 mesh silica gel) using MTBE : hexane (0:100 → 5:95) as white solid (70.8 mg, 55%).

^{1}H NMR (400 MHz, Chloroform-*d*) δ 8.16 (d, *J* = 8.5 Hz, 2H), 7.86 (d, *J* = 8.5 Hz, 2H), 7.63 – 7.58 (m, 2H), 7.35 (dddd, *J* = 14.4, 8.7, 5.8, 2.3 Hz, 3H), 4.79 (ddt, *J* = 8.2, 6.4, 3.0 Hz, 2H), 3.12 – 3.07 (m, 4H), 2.65 (dq, *J* = 15.0, 11.5 Hz, 1H), 2.42 – 2.27 (m, 2H), 2.14 – 2.03 (m, 1H), 1.60 – 1.48 (m, 4H), 1.24 (s, 6H), 1.21 (s, 6H), 0.86 (t, *J* = 7.4 Hz, 6H).

^{13}C NMR (100 MHz, Chloroform-*d*) δ 165.2, 144.2, 137.3, 133.7, 130.3, 129.7, 129.5, 128.9, 126.9, 126.1 (q, *J* = 279.3 Hz), 84.7, 62.7, 49.9, 40.6 (q, *J* = 27.4 Hz), 32.2, 24.9, 24.7, 21.9, 11.2. The carbon attached to the boron was not found due to quadrupolar relaxation.

^{11}B NMR (128 MHz, Chloroform-*d*) δ 32.3

^{19}F NMR (376 MHz, Chloroform-*d*) δ -59.96 (t, *J* = 10.9 Hz).

HRMS (ESI) calcd for $C_{30}H_{41}BF_3NNaO_6S_2$ $[M+Na]^+$: 666.2318; found: 666.2338.

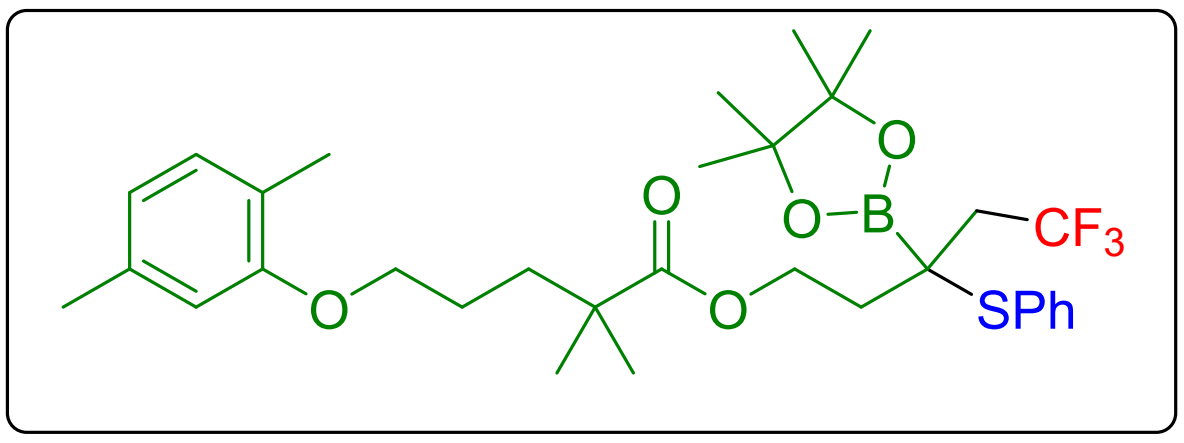

5,5,5-trifluoro-3-(phenylthio)-3-(4,4,5,5-tetramethyl-1,3,2-dioxaborolan-2-yl)pentyl 5-(2,5-dimethylphenoxy)-2,2-dimethylpentanoate (3qa)

The product **3qa** was synthesized following General Procedure for Three-Component Conjunctive Coupling 2 (GP2) using thiophenol (1.5 eq., 0.3 mmol, 33 mg). **3qa** was isolated

by flash column chromatography (200-400 mesh silica gel) using MTBE : hexane (0:100 → 5:95) as white solid (79.1 mg, 65%).

^{1}H NMR (400 MHz, Chloroform-*d*) δ 7.63 – 7.52 (m, 2H), 7.39 – 7.30 (m, 3H), 7.01 (d, *J* = 7.5 Hz, 1H), 6.66 (d, *J* = 7.5 Hz, 1H), 6.61 (s, 1H), 4.50 (pd, *J* = 10.7, 5.6 Hz, 2H), 3.94 – 3.89 (m, 2H), 2.60 (dq, *J* = 15.0, 11.4 Hz, 1H), 2.39 – 2.27 (m, 4H), 2.20 – 2.13 (m, 4H), 1.90 (ddd, *J* = 14.8, 9.5, 5.6 Hz, 1H), 1.80 – 1.69 (m, 1H), 1.25 (s, 6H), 1.23 (s, 6H), 1.22 (s, 6H).

^{13}C NMR (125 MHz, Chloroform-*d*) δ 177.8, 157.0, 137.3, 136.5, 130.3, 129.7, 129.4, 128.8, 126.1 (q, *J* = 279.1 Hz), 123.6, 120.7, 111.9, 84.6, 67.9, 61.5, 42.0, 40.6 (q, *J* = 27.2 Hz), 37.1, 34.8 (br), 32.3, 25.2, 25.2, 24.9, 24.7, 21.4, 15.8.

^{11}B NMR (160 MHz, Chloroform-*d*) δ 31.8

^{19}F NMR (376 MHz, Chloroform-*d*) δ -59.91 (t, *J* = 10.4 Hz).

HRMS (ESI) calcd for $C_{32}H_{44}BF_3NaO_5S$ $[M+Na]^+$: 631.2852; found: 631.2859.

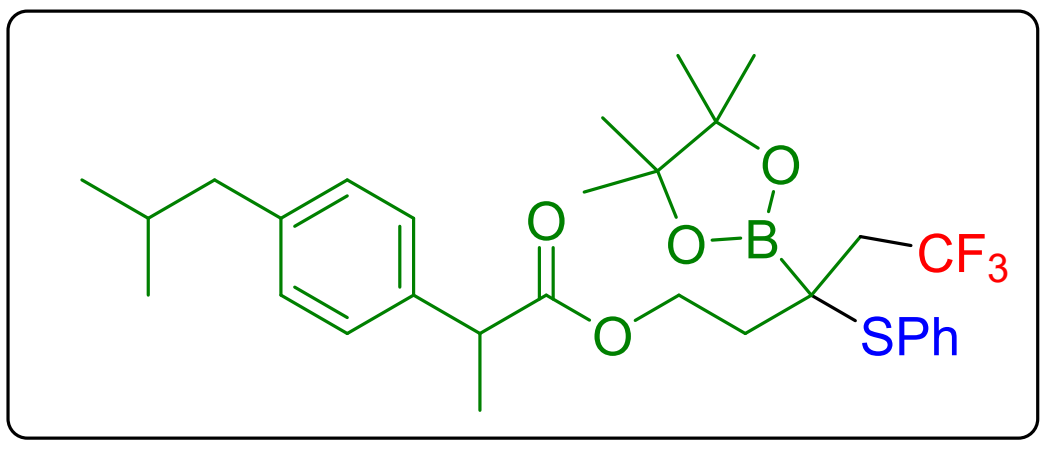

5,5,5-trifluoro-3-(phenylthio)-3-(4,4,5,5-tetramethyl-1,3,2-dioxaborolan-2-yl)pentyl 2-(4-isobutylphenyl)propanoate (3ra)

The product **3ra** was synthesized following General Procedure for Three-Component Conjunctive Coupling 2 (GP2) using thiophenol (1.5 eq., 0.3 mmol, 33 mg) as 1:1 diastereomeric mixture. **3ra** was isolated by flash column chromatography (200-400 mesh silica gel) using MTBE : hexane (0:100 → 5:95) as white solid (101.6 mg, 90%) (1:1 dr).

^{1}H NMR (500 MHz, Chloroform-*d*) δ 7.47 – 7.42 (m, 2H), 7.27 (tt, *J* = 5.9, 2.9 Hz, 1H), 7.23 – 7.18 (m, 2H), 7.12 (dd, *J* = 8.1, 1.9 Hz, 2H), 7.03 – 7.00 (m, 2H), 4.49 – 4.32 (m, 2H), 3.60 (qd, *J* = 7.2, 2.6 Hz, 1H), 2.49 – 2.39 (m, 1H), 2.37 (dd, *J* = 7.1, 1.8 Hz, 1H), 2.25 – 2.12 (m, 1H), 2.02 (qd, *J* = 9.9, 8.9, 3.1 Hz, 1H), 1.82 – 1.73 (m, 2H), 1.41 (dd, *J* = 7.2, 2.3 Hz, 3H), 1.15 – 1.14 (6H), 1.12 – 1.11 (6H), 0.82 (d, *J* = 6.6 Hz, 6H).

^{13}C NMR (125 MHz, Chloroform-*d*) δ 174.7, 140.5, 137.9, 137.8, 137.3, 129.7, 129.6, 129.4, 129.33, 129.31, 129.28, 128.8, 127.2, 127.2, 126.1 (q, *J* = 279.1 Hz), 84.6, 61.8, 61.7, 45.2, 45.1, 40.5 (q, *J* = 26.9 Hz), 40.4 (q, *J* = 27.2 Hz), 34.9 (br), 30.2, 24.9, 24.7, 22.4, 18.7.

^{11}B NMR (160 MHz, Chloroform-*d*) δ 31.8

^{19}F NMR (470 MHz, Chloroform-*d*) δ -59.82 (m).

^{19}F{^{1}H} NMR (470 MHz, Chloroform-*d*) δ -59.81 (s, 3F), -59.83 (s, 3F).

HRMS (ESI) calcd for $C_{30}H_{40}BF_3NaO_4S$ $[M+Na]^+$: 587.2590; found: 587.2617.

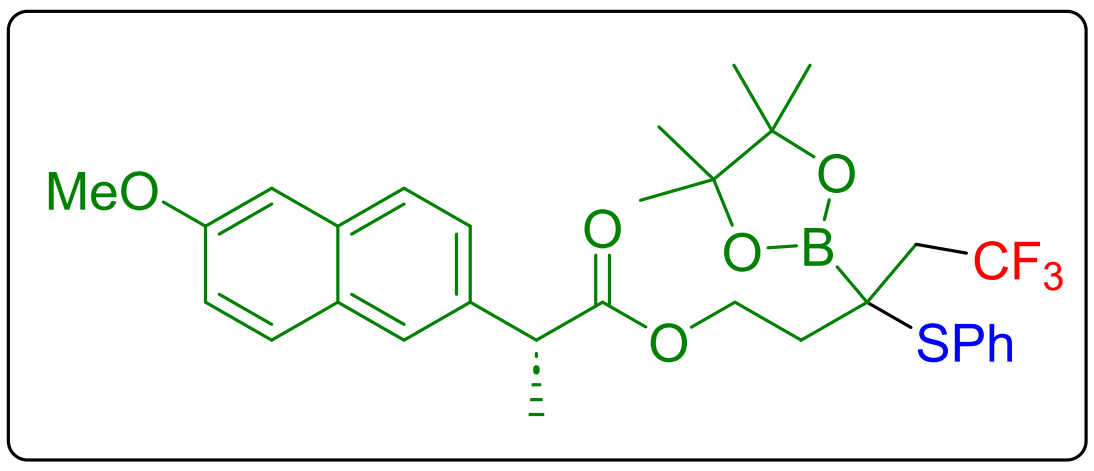

5,5,5-trifluoro-3-(phenylthio)-3-(4,4,5,5-tetramethyl-1,3,2-dioxaborolan-2-yl)pentyl (2R)-2-(6-methoxynaphthalen-2-yl)propanoate (3sa)

The product **3ra** was synthesized following General Procedure for Three-Component Conjunctive Coupling 2 (GP2) using thiophenol (1.5 eq., 0.3 mmol, 33 mg) as 1:1 diastereomeric mixture. **3ra** was isolated by flash column chromatography (200-400 mesh silica gel) using MTBE : hexane (0:100 → 50:50) as white solid (58.8 mg, 50%) (1:1 dr).

^{1}H NMR (500 MHz, Chloroform-*d*) δ 7.71 – 7.66 (m, 3H), 7.52 – 7.40 (m, 3H), 7.34 – 7.10 (m, 5H), 4.59 – 4.42 (m, 2H), 3.92 (s, 3H), 3.85 (qd, *J* = 7.2, 2.7 Hz, 1H), 2.52 (ddq, *J* = 22.9, 15.1, 11.3 Hz, 1H), 2.32 – 2.19 (m, 1H), 2.13 – 2.08 (m, 1H), 1.91 – 1.82 (m, 1H), 1.58 (dd, *J* = 7.2, 2.4 Hz, 3H), 1.22 – 1.18 (m, 12H).

^{13}C NMR (125 MHz, Chloroform-*d*) δ 174.7, 157.7, 137.4, 137.3, 135.9, 135.8, 133.8, 129.8, 129.7, 129.4, 129.3, 129.3, 129.1, 129.0, 128.9, 128.8, 127.3, 127.2, 126.4, 126.3, 126.2 (q, *J* = 279.1 Hz), 126.1, 119.1, 119.0, 105.7, 84.7, 62.1, 62.0, 55.4, 45.6, 45.6, 40.6 (q, *J* = 27.2 Hz), 40.5 (q, *J* = 27.2 Hz), 34.9 (br), 32.3, 32.2, 25.0, 24.9, 24.7, 18.8, 18.7.

^{11}B NMR (160 MHz, Chloroform-*d*) δ 31.9

^{19}F NMR (376 MHz, Chloroform-*d*) δ -59.76 – -59.85 (m).

^{19}F{^{1}H} NMR (376 MHz, Chloroform-*d*) δ -59.79 (s, 3F), -59.83 (s, 3F).

HRMS (ESI) calcd for $C_{31}H_{37}BF_3O_5S$ $[M+H]^+$: 589.2407; found: 589.2424.

VI. Procedures for Synthetic Transformations

Homologation Reaction

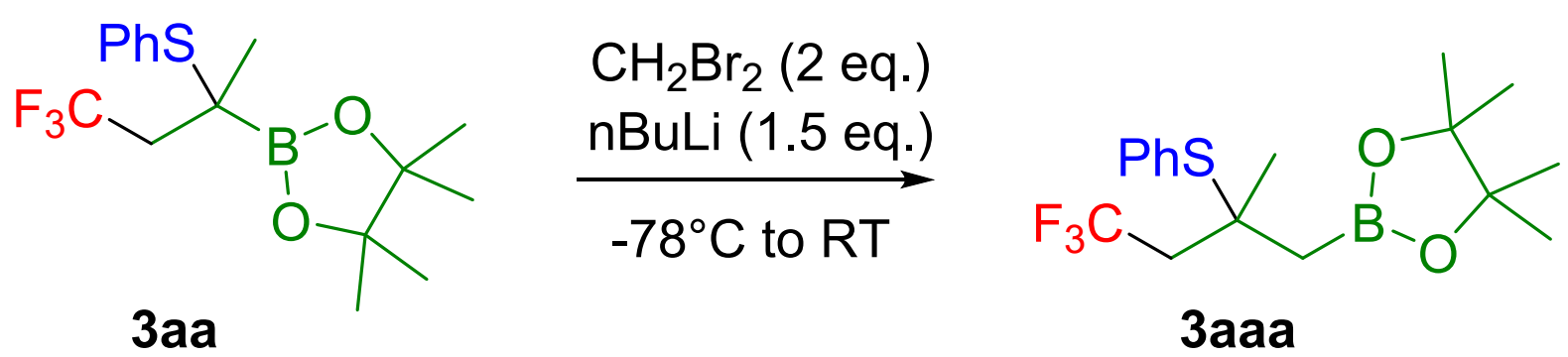

3aa 3aaa

4,4,5,5-tetramethyl-2-(4,4,4-trifluoro-2-methyl-2-(phenylthio)butyl)-1,3,2-dioxaborolane (3aaa)

In an oven dried 25mL Schlenk tube, 3aa (34.6 mg, 0.1 mmol, 1.0 equiv) was dissolved in tetrahydrofuran (3mL), and dibromomethane (34.8 mg, 0.2 mmol, 2.0 equiv) was added. The mixture was cooled to -78 °C, n-butyllithium (0.15 mmol) was added dropwise. The reaction mixture was stirred at -78 °C for 30 mins, and then warmed to room temperature and stirred for another 2 hours. The mixture was quenched with saturated aqueous sodium thiosulfate solution. The aqueous phase was extracted with diethyl ether, dried over anhydrous sodium sulfate. Solvent was removed under reduced pressure, purified by column chromatography on silica gel with hexane/diethyl ether as eluent to give the corresponding product 3c (30.6 mg, 85% yield, colorless oil).

^{1}H NMR (400 MHz, Chloroform-*d*) δ 7.59 – 7.56 (m, 2H), 7.37 (dqd, *J* = 10.2, 7.2, 1.6 Hz, 3H), 2.59 (dqd, *J* = 15.3, 11.4, 1.3 Hz, 1H), 2.41 (dqd, *J* = 15.4, 11.3, 1.3 Hz, 1H), 1.46 (s, 3H), 1.36 (s, 1H), 1.34 (s, 1H), 1.28 (s, 12H).

^{13}C NMR (125 MHz, Chloroform-*d*) δ 137.89, 131.28, 129.26, 128.78,126.32 (q, *J* = 279.4 Hz), 83.39, 46.84, 44.06 (q, *J* = 26.3 Hz), 28.56, 24.89, 24.76. The carbon attached to the boron was not found due to quadrupolar relaxation.

^{11}B NMR (128 MHz, Chloroform-*d*) δ 32.5

^{19}F NMR (376 MHz, Chloroform-*d*) δ -59.52 (t, *J* = 11.3 Hz).

HRMS (ESI) calcd for $C_{17}H_{24}BF_3NaO_2S$ $[M+Na]^+$: 383.1440; found: 383.1454.

Molander Salt Preparation

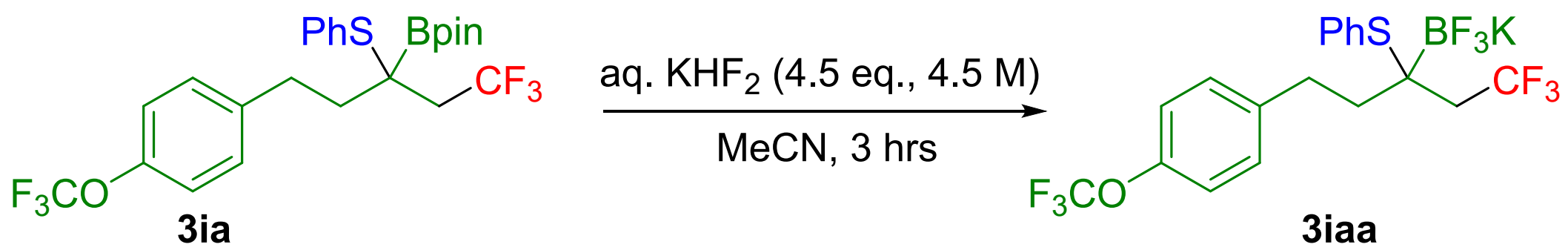

3ia **3iaa**

trifluoro(1,1,1-trifluoro-3-(phenylthio)-5-(4-(trifluoromethoxy)phenyl)pentan-3-yl)-l4-borane, potassium salt (3iaa)

In an oven dried 50 mL round-bottom flask, **3ia** (156.1 mg, 0.347 mmol, 1.0 equiv) was dissolved in 5mL of CH_3CN and saturated aqueous potassium hydrogenfluoride solution (4.5M, 4.5 eq., 0.35 mL) was added. After 3hr stirring at room temperature, the solvent was evaporated under reduced pressure. 5 mL water and 5 mL MeOH were added in the crude mixture and then dried. This process was repeated for three times and then kept under vacuum for 12 hr. The crude product was dissolved in acetone. The solid was filtered off, and the filtrate was concentrated under reduced pressure to obtain half-white solid as a product. The resulting solid was washed twice with Et_2O to afford pure product **3iaa** (119.8 mg, 69%).

^{1}H NMR (400 MHz, Acetone-*d$_6$*) δ7.40 (dd, *J* = 6.4, 3.2 Hz, 2H), 7.14 – 7.12 (m, 5H), 7.00 (d, *J* = 8.2 Hz, 2H), 3.05 (td, *J* = 13.2, 4.3 Hz, 1H), 2.77 (td, *J* = 13.5, 4.6 Hz, 1H), 2.55 – 2.42 (m, 1H), 1.85 (ddt, *J* = 17.9, 13.6, 6.2 Hz, 2H).

^{13}C NMR (125 MHz, Acetone-*d$_6$*) δ 146.79, 144.08, 137.39, 133.55, 129.88, 128.17, 127.8, 127.7 (q, *J* = 279.4 Hz), 120.75, 120.63 (q, *J* = 254.6 Hz), 44.45 (br), 41.74 (q, *J* = 25.7 Hz), 38.41, 32.17.

^{11}B NMR (128 MHz, Acetone-*d$_6$*) δ 3.42.

^{19}F NMR (376 MHz, Acetone-*d$_6$*) δ -58.58 (s, 3F), -58.90 (tq, *J* = 11.7, 5.6 Hz, 3F), -143.94 (br, 3F).

HRMS (ESI) calcd for $C_{18}H_{15}BF_9K_2OS$ $[M+K]^+$: 539.0067; found: 539.0081.

VII. Mechanistic Studies

<u>Radical Trapping Reaction with BHT</u>

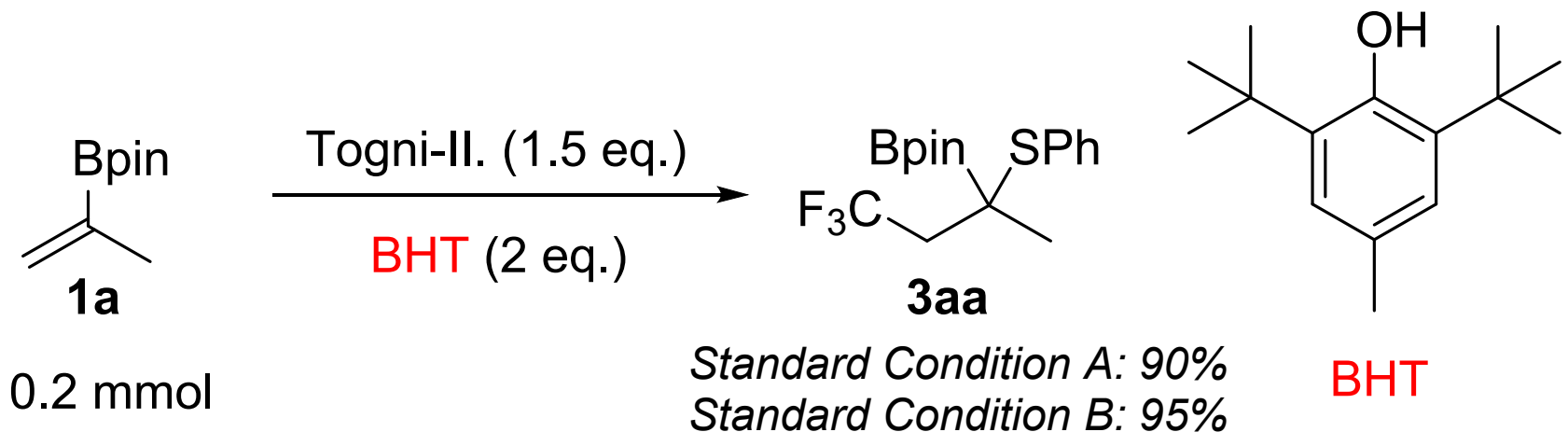

Entry	BHT (eq.)	Yield of **3aa** (Standard Condition B) [(a)]	Yield of **3aa** (standard Condition A) [(a)]
1	2 eq.	90 %	95%
2	3 eq.	91%	94%
3	4 eq.	90%	96%

[(a)] ^{19}F NMR yields are reported using 3-trifluoromethylphenol as the internal standard.

Standard Condition B: In a reaction tube equipped with teflon screw cap containing teflon coated magnetic stirr bar, the corresponding disulfide (0.75 eq., 0.15 mmol) was dissolved in 0.2 mL CPME under nitrogen atmosphere, then 0.3 mL $LiHBEt_3$ (1(M) in THF) (1.5 eq.) was added at -78 °C; stirred for 40 mins. Vinyl boronate (0.2 mmol) in 0.5 mL CPME was added to the reaction at -78 °C and stirred for 1 hr. The resulting reaction mixture was transferred to another reaction tube containing a solution of Togni-II (1.5 eq., 0.3 mmol, 95 mg) and BHT (2eq., 0.4 mmol, 88.2 mg) in 0.2 mL CPME at -78 °C and stirred for 2 hrs at -78 °C. The reaction mixture was allowed to warm up to RT over 2 hrs and stirred at RT for another 8 hrs. After the completion of the reaction, the reaction was diluted with 2 mL ethyl acetate and quenched with 2 mL brine. The layers were separated and the aqueous layer was washed with ethyl acetate (2 mL × 3). The combined organic layer was dried over Na_2SO_4, filtered and concentrated under reduced pressure to obtain the crude reaction mixture. ^{19}F NMR yields are reported using 3-trifluoromethylphenol as the internal standard.

Standard Condition A:

In a reaction tube equipped with teflon screw cap containing teflon coated magnetic stir bar, thiophenol (1.5 eq., 0.3 mmol) was dissolved in 0.2 mL CPME, then 0.3 mL BEt_3 (1(M) in THF) was added at -78 °C; stirred for 40 mins. Vinyl boronate (0.2 mmol) in 0.5 mL CPME was added to the reaction at -78 °C and stirred for 1 hr. The resulting reaction mixture was transferred to another reaction tube containing a solution of Togni-II (1.5 eq., 0.3 mmol, 95 mg) and BHT (2eq., 0.4 mmol, 88.2 mg) in 0.2 mL CPME at -78°C and stirred for 2 hrs at -78°C. The reaction mixture was allowed to warm up to RT over 2 hrs and stirred at RT for another 8 hrs. After the completion of the reaction, the reaction was diluted with 2 mL ethyl

acetate and quenched with 2 mL brine. The layers were separated and the aqueous layer was washed with ethyl acetate (2 mL × 3). The combined organic layer was dried over Na_2SO_4, filtered and concentrated under reduced pressure to obtain the crude reaction mixture. ^{19}F NMR yields are reported using 3-trifluoromethylphenol as the internal standard.

Radical Trapping Reaction with DHA

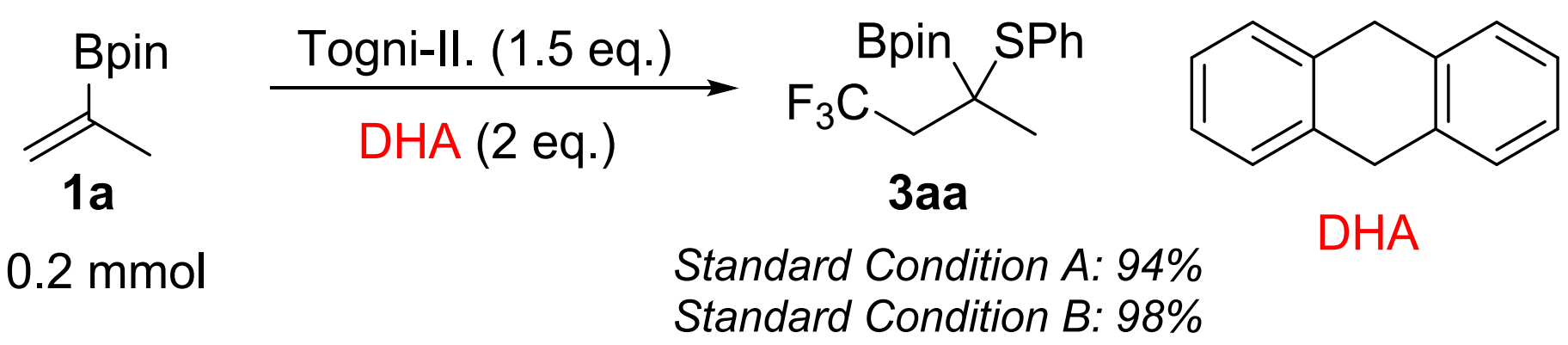

Entry	DHA (eq.)	Yield of **3aa** (Standard Condition B)[(a)]	Yield of **3aa** (satndard Condition A)[(a)]
1	2 eq.	94%	98%
2	3 eq.	93%	98%
3	4 eq.	94%	97%

[(a)] ^{19}F NMR yields are reported using 3-trifluoromethylphenol as the internal standard.

Standard Condition B: In a reaction tube equipped with teflon screw cap containing teflon coated magnetic stir bar, the corresponding disulfide (0.75 eq., 0.15 mmol) was dissolved in 0.2 mL CPME under nitrogen atmosphere, then 0.3 mL $LiHBEt_3$ (1(M) in THF) (1.5 eq.) was added at -78 °C; stirred for 40 mins. Vinyl boronate (0.2 mmol) in 0.5 mL CPME was added to the reaction at -78 °C and stirred for 1 hr. The resulting reaction mixture was transferred to another reaction tube containing a solution of Togni-II (1.5 eq., 0.3 mmol, 95 mg) and DHA (2eq., 0.4 mmol, 72 mg) in 0.2 mL CPME at -78 °C and stirred for 2 hrs at -78 °C. The reaction mixture was allowed to warm up to RT over 2 hrs and stirred at RT for another 8 hrs. After the completion of the reaction, the reaction was diluted with 2 mL ethyl acetate and quenched with 2 mL brine. The layers were separated and the aqueous layer was washed with ethyl acetate (2 mL × 3). The combined organic layer was dried over Na_2SO_4, filtered and concentrated under reduced pressure to obtain the crude reaction mixture. ^{19}F NMR yields are reported using 3-trifluoromethylphenol as the internal standard.

Standard Condition A:

In a reaction tube equipped with teflon screw cap containing teflon coated magnetic stir bar, thiophenol (1.5 eq., 0.3 mmol) was dissolved in 0.2 mL CPME, then 0.3 mL BEt_3 (1(M) in

THF) was added at -78 °C; stirred for 40 mins. Vinyl boronate (0.2 mmol) in 0.5 mL CPME was added to the reaction at -78 °C and stirred for 1 hr. The resulting reaction mixture was transferred to another reaction tube containing a solution of Togni-II (1.5 eq., 0.3 mmol, 95 mg) and DHA (2eq., 0.4 mmol, 72 mg) in 0.2 mL CPME at -78 °C and stirred for 2 hrs at -78 °C. The reaction mixture was allowed to warm up to RT over 2 hrs and stirred at RT for another 8 hrs. After the completion of the reaction, the reaction was diluted with 2 mL ethyl acetate and quenched with 2 mL brine. The layers were seperated and the aquous layer was washed with ethyl acetate (2 mL × 3). The combined organic layer was dried over Na_2SO_4, filtered and concentrated under reduced pressure to obtain the crude reaction mixture. ^{19}F NMR yields are reported using 3-trifluoromethylphenol as the internal standard.

<u>Variable Tepmerature NMR Experiment-i</u>

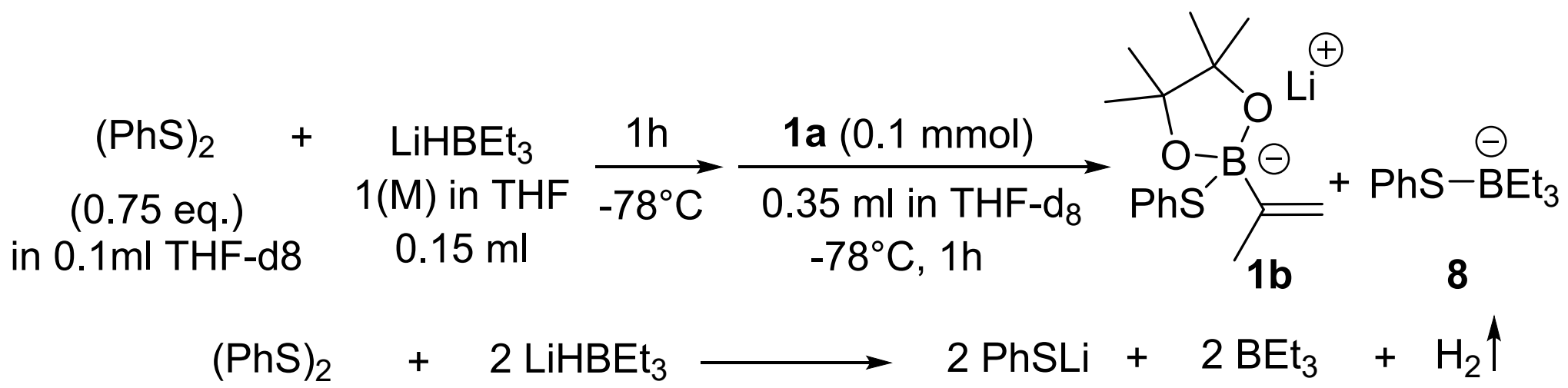

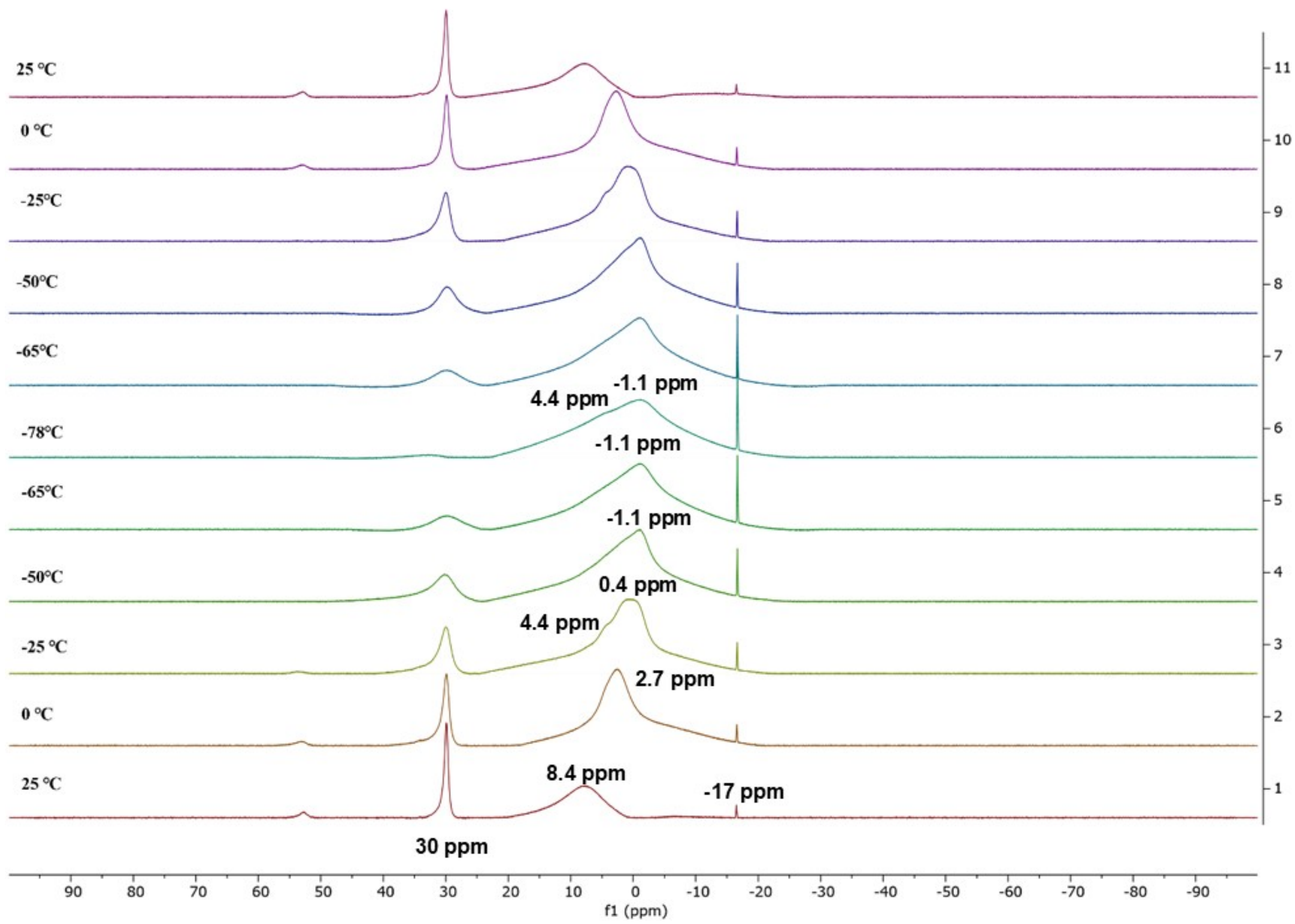

In a schlenk tube with screw cap equuiped with a teflon coated stirr bar, 16.4 mg, 0.075 mmol of $(PhS)_2$ was added followed by 0.1 mL THF-d8 inside a N_2-filled glove box, . The tube was taken out of the glove box and cooled down to -78 °C. 0.15 ml of commercially availbable 1(M) superhydride solution in THF [Super-Hydride® solution 1.0 M lithium triethylborohydride in THF, Sigma-Aldrich] was added dropwise under nitrogen and stirred for 1 hour. To this reaction mixture a 0.35 mL THF-d_8 solution of **1a** was added and stirred for another 1 hour at -78 °C. Then, the tube was warmed up to RT and taken inside glove box. The solution was transferred to NMR tube. After reacting the in-situ prepared PhSLi with **1a** for 1h at -78 °C, the NMR was recorded at RT, which shows a peak at 30 ppm corresponding to **1a**

Figure S2. Variable Temperature ^{11}B NMR of the reaction mixture containing in situ generated PhSLi, BEt_3 and **1a**. From 25 °C to -78 °C and from -78 °C to 25 °C. (From below to up).

along with a peak at 8.4 ppm indicating the coordination of thiolate nucleophile to BEt_3 (The ^{11}B chemical shift of BEt_3 is reported to be δ 86.6,[6] which is not present in the ^{11}B NMR at room temperature. The peak at -17 ppm corresponds to $LiBEt_4$ impurity present in the commercially available superhydride solution). The observed line broadening of these peaks at lower temperatures likely result from the dynamic exchange of the thiolate ligand between the

different ‘ate’ complexes **1b** and **8**. Thus, the coordination of sulfur nucleophile to both the boron center of BEt_3 and the Bpin-group of 1a is temperature dependent and reversible.

Variable Tepmerature NMR Experiment-ii

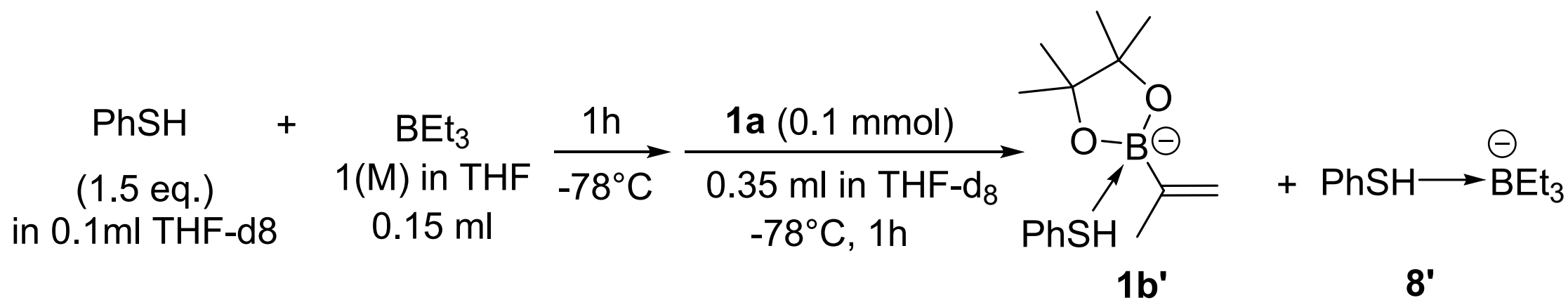

In a schlenk tube with screw cap equuiped with a teflon coated stirr bar, 16.5 mg, 0.15 mmol of PhSH was added followed by 0.1 mL THF-d8 inside a N_2-filled glove box, . The tube was taken out of the glove box and cooled down to -78 °C. 0.15 ml of commercially availbable 1(M) BEt_3 solution in THF was added dropwise under nitrogen and stirred for 1 hour. To this reaction mixture a 0.35 mL THF-d_8 solution of **1a** was added and stirred for another 1 hour at

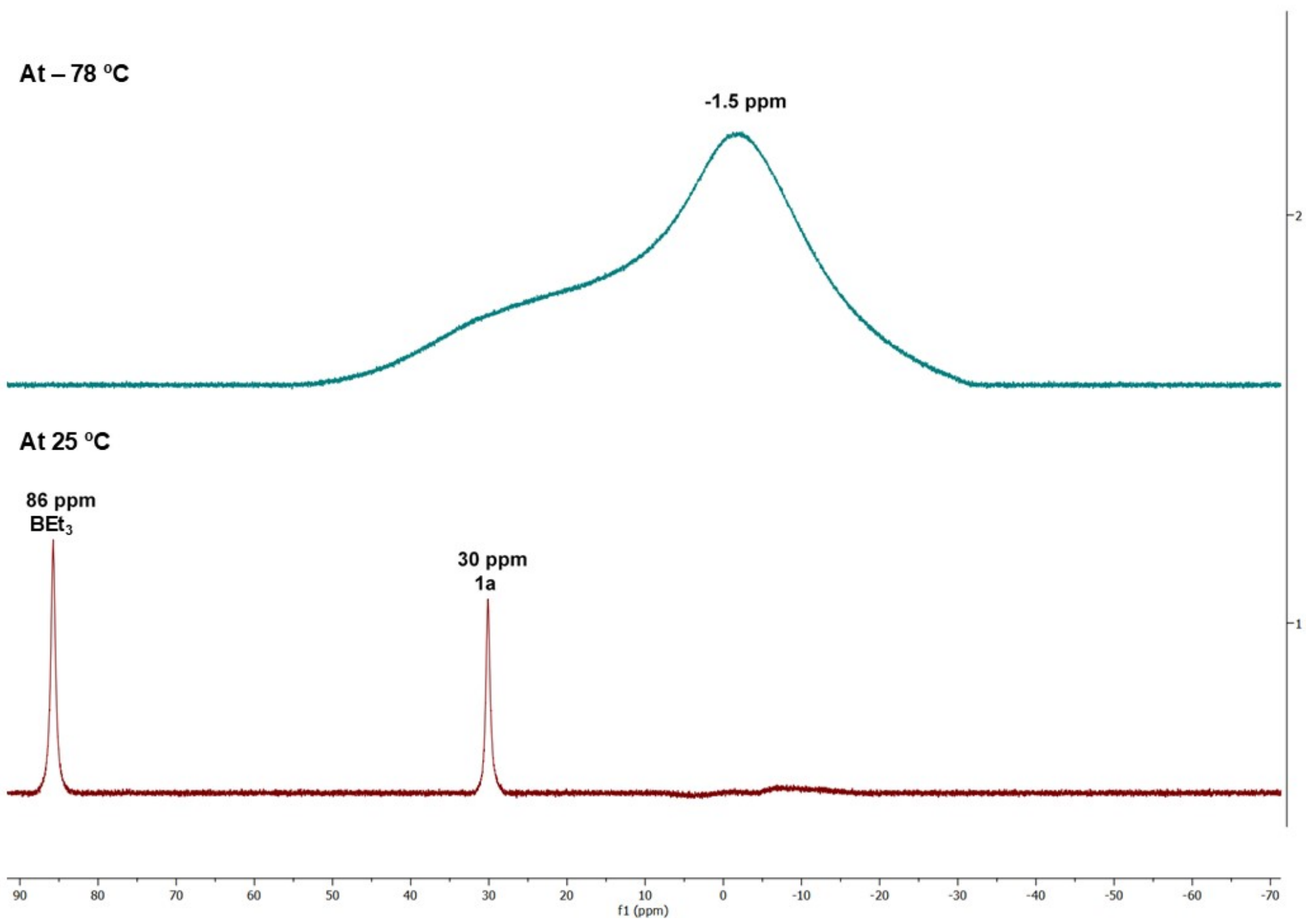

-78 °C. Then, the tube was warmed up to RT and taken inside glove box. The solution was transferred to NMR tube.

Figure S3. Variable Temperature ^{11}B NMR of the reaction mixture containing PhSH, BEt_3 and **1a**

Upon lowering the temperature to –78 °C, the sharp peaks for the free boron species largely disappeared and coalesced into a single, massive broad resonance. This dramatic line broadening is characteristic of an intermediate exchange regime on the NMR timescale. Unlike the PhSLi system, which forms stable, discrete ate-complexes due to the strong nucleophilicity of the thiolate and the electrophilic activation by Li^+, the neutral PhSH is a weaker Lewis base. Consequently, its coordination to the unactivated Bpin group is highly reversible. The broad signal at –78 °C confirms that dynamic coordination is indeed occurring, but the PhSH ligand rapidly exchanges between the free and bound states. This confirms that boronate complexation is accessible in the PhSH pathway, albeit existing as a highly fluxional, dynamic pre-equilibrium rather than a deeply trapped intermediate. While the resonance for free BEt_3 (86 ppm) completely vanishes, a residual signal for free **1a** near 30 ppm remains discernible. This observation aligns perfectly with the relative Lewis acidities of the two boron centers. The neutral PhSH preferentially and quantitatively coordinates to the stronger Lewis acid, BEt_3. Because this secondary coordination event lacks the potent electrophilic activation provided by a lithium cation, it is much weaker and highly reversible. Consequently, while the BEt_3 is fully engaged in the complex, the Bpin moiety is not quantitatively consumed, resulting in the incomplete disappearance of the 30 ppm resonance alongside the massive intermediate exchange signal.

<u>**Variable Tepmerature NMR Experiment-iii**</u>

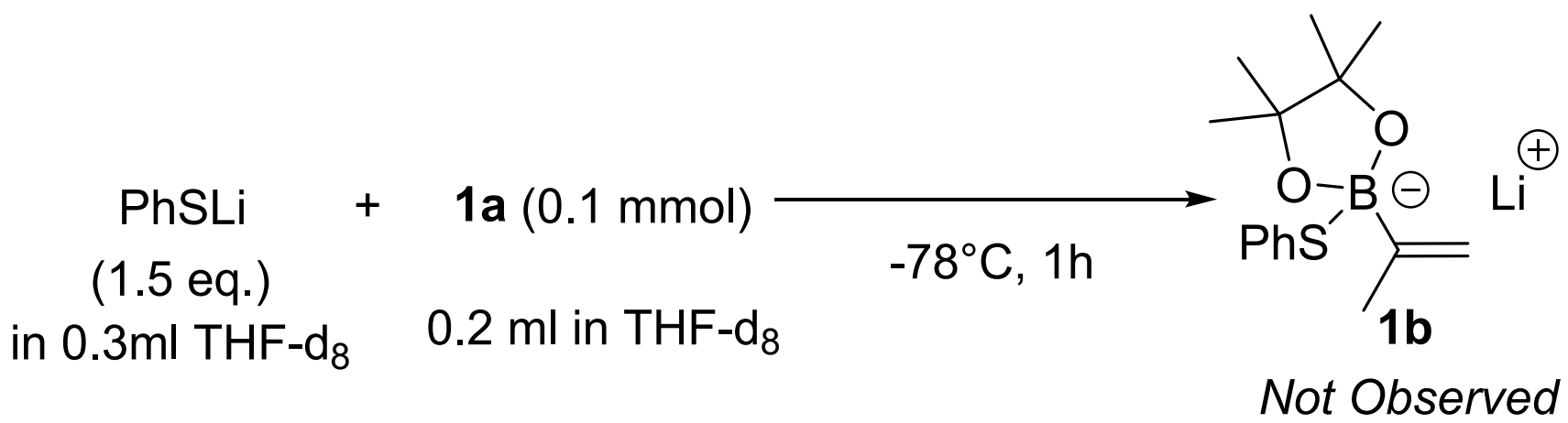

<u>Isolation of PhSLi:</u> A 10 ml hexane solution 5 mmol (1 eq.) of PhSH was reacted with 4.5 mmol of ^nBuLi (1.6 M in hexane) at 0 ºC for 2 hours. The reaction mixture was warmed up to RT and filtered under N_2 using cannula, dried under vacuum.

In a schlenk tube with screw cap equuiped with a teflon coated stirr bar, 17.4 mg, 0.15 mmol of PhSLi was added followed by 0.3 mL THF-d_8 inside a N_2-filled glove box, . The tube was taken out of the glove box and cooled down to -78 °C. 0.3 ml solution of **1a** (0.1 mmol) in THF was added dropwise under nitrogen and stirred for 1 hour. Then, the tube was warmed up to RT and taken inside glove box. The solution was transferred to NMR tube.

To confirm the necessity of BEt_3 and the $[PhS–BEt_3]^–$ ate-complex in activating the system, this control experiment was conducted utilizing only **1a** and PhSLi. Variable-temperature ^{11}B NMR revealed that even at –78 °C, no signals emerged near 0 ppm and the 30 ppm peak corresponding to **1a** remained intact. This demonstrates that without BEt_3 to sequester the thiolate and liberate a highly oxophilic Li^+ cation, the Bpin moiety remains unactivated and cannot be attacked by the thiolate nucleophile.

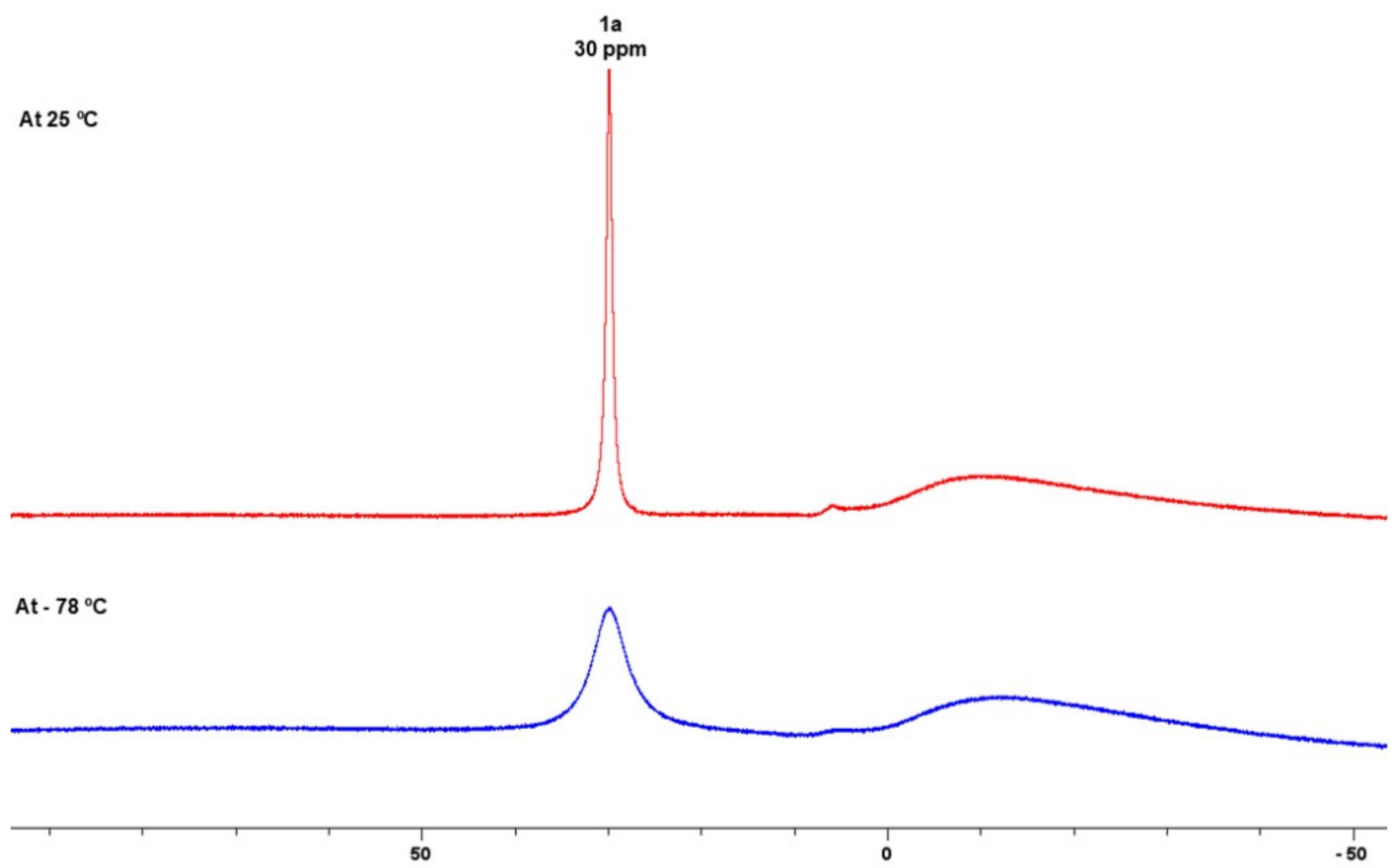

Figure S4. Variable Temperature ^{11}B NMR of the reaction mixture of isolated PhSLi and **1a**.

VIII. X-Ray Crystallography Data

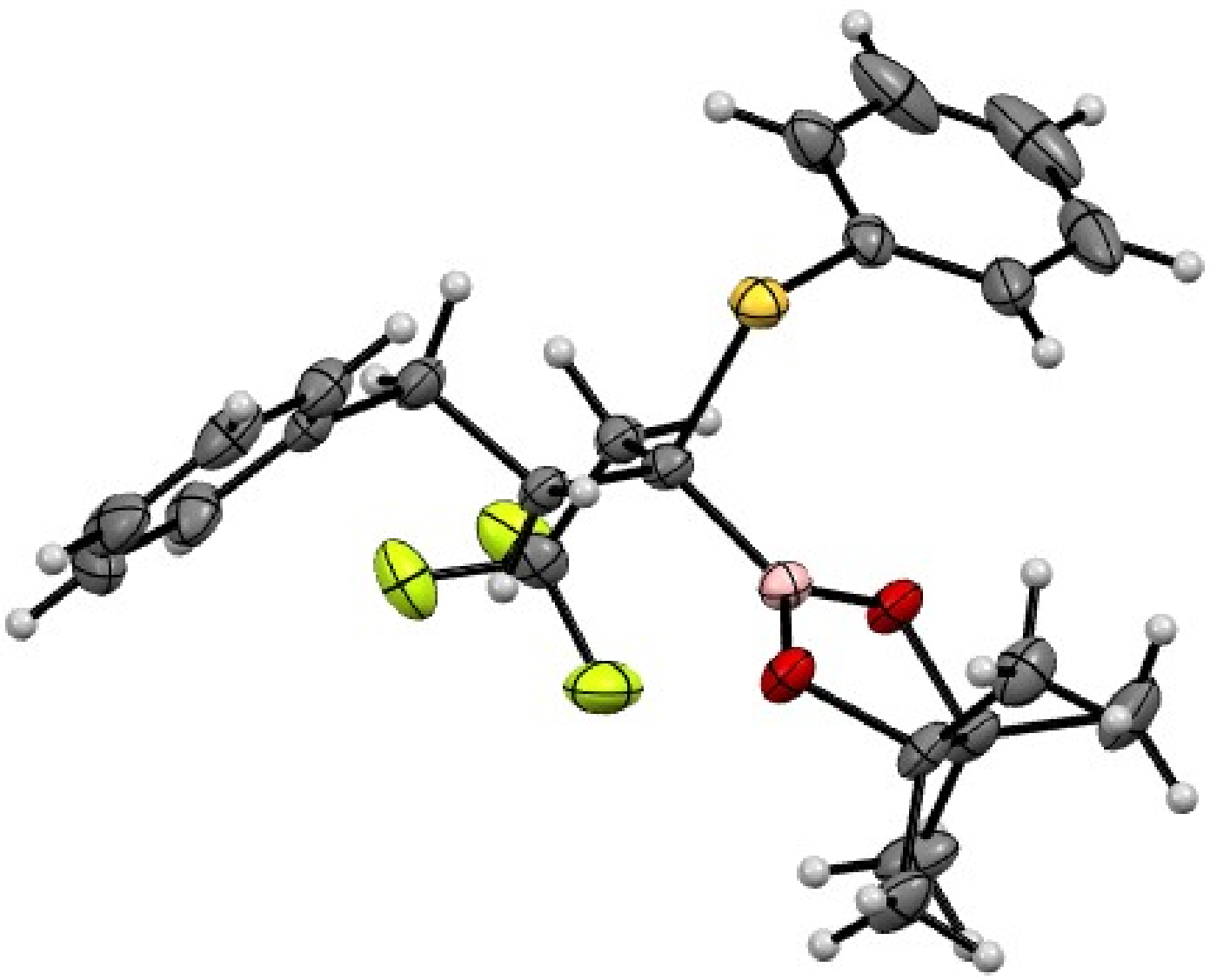

Figure S5. Molecular structure of **3da** with thermal ellipsoids at 50% probability level.

Table S10. Crystallographic information of compound **3da**.

Emperical formula	C23H28BF3O2S
Formula weight	436.32
Temperature/K	120K
Crystal System	monoclinic
Space group	P 21/c
a/Å	13.3845(5)
b/Å	14.5893(6)
c/Å	13.2217(5)
α/º	90
β/º	119.102(2)
γ/º	90
Volume/Å^3	2255.87(16)
Z	4
ρcalcg/cm^3	1.28
μ/mm-1	0.184
F (000)	920.0
Radiation	MoK\α (0.71073)
Reflections collected	5192
Goodness-of-fit on F^2	1.008
R_factor_gt	0.0662

CCDC Deposition Number 2533157

IX. DFT Calculations

All calculations were performed using the Gaussian 16 program at the B3LYP-D3 [7-9] level of theory with the def2-SVP[10-11]basis set in the CPCM[12-15]solvent model, using THF. Harmonic vibrational frequency analyses at 298 K and 1 atm confirmed the nature of each stationary point: intermediates exhibited no imaginary frequencies, whereas transition states had one imaginary frequency corresponding to the expected reaction coordinate. Visualization and analysis of molecular geometries, orbitals, and vibrational modes were carried out using visualization tools like GaussView6 [16], ChemCraft[17], Avogadro[18-19], and CYLview.

Analyzing the effect of Li^+ coordination to the pinacol oxygen of **1d**.

The formation of PhS–BEt_3 increases the availability of Li^+ for coordination. The liberated Li^+ can then interact with the pinacolato oxygen atoms of the vinyl Bpin (in this case, **1d**), altering the electronic distribution within the B–O bond.

To rationalize these observations, we first established the LUMO energy of free **1d** as −0.613 eV using DFT (B3LYP-D3 level of theory with the def2-SVP basis set in the CPCM solvent model, implicit solvation: THF). Coordination of the experimentally relevant species, the Li^+ cation within the **1d-Li-[PhS-BEt$_3$]** complex, where Li^+ bridges the pinacolato oxygen (O1) and the sulfur atom of $[PhS\text{-}BEt_3]^-$, lowers the LUMO energy to −0.796 eV (a stabilization of 0.183 eV). This stabilization of the boron-centered acceptor orbital indicates enhanced electrophilicity at boron, facilitating nucleophilic attack by PhS^- and supporting the experimentally observed role of BEt_3 in promoting boronate complex formation.

For reference, coordination of a fully dissociated ('naked') Li^+ to **1d** lowers the LUMO energy further to −1.245 eV, representing the *theoretical upper limit* of activation.

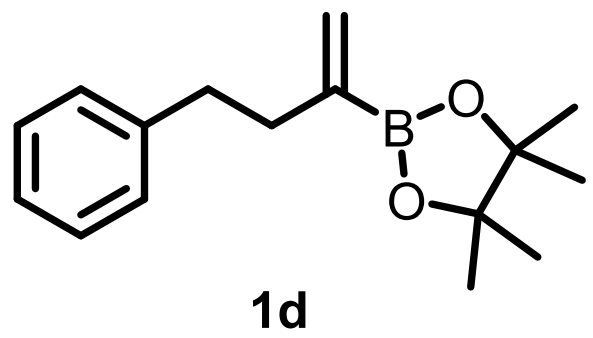

1d

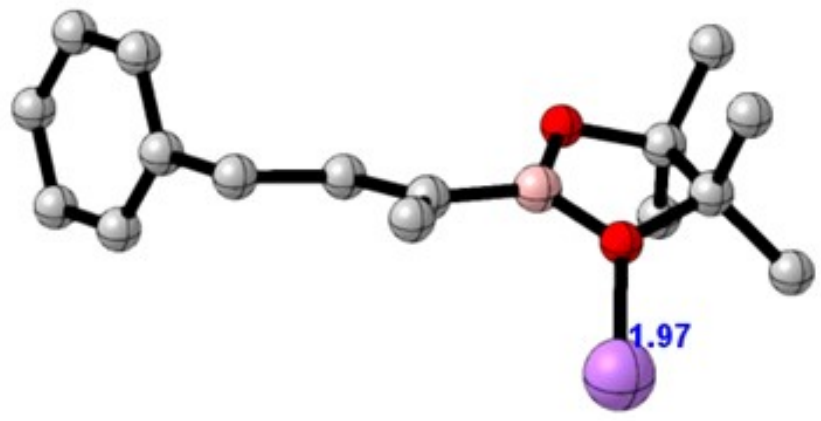

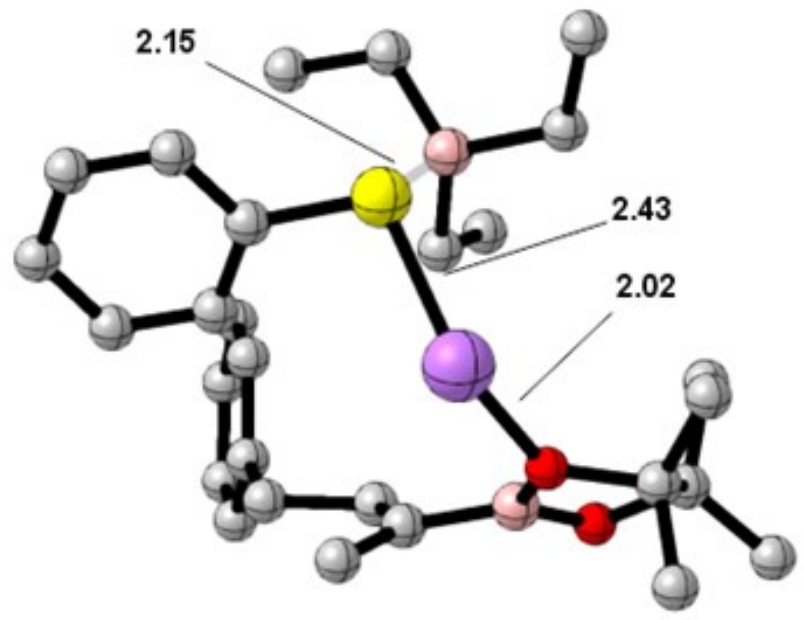

1d-Li: the pinacol oxygen of 1d is coordinated with a 'naked' Li$^+$ cation

1d-Li-[PhS-BEt$_3$]: the pinacol oxygen of 1d is coordinated with a Li$^+$ cation, which is bound to the S-atom of the [PhS-BEt$_3$]$^-$ adduct 8

Structure	LUMO Energy	Stabilization
1d	−0.613 eV	-
1d-Li-[PhS-BEt$_3$]	−0.796 eV	0.183 eV
1d-Li	−1.245 eV	0.632 eV

Figure S6. Analyzing the effect of Li$^+$ coordination to the pinacol oxygen of **1d**.

Cartesian coordinates of optimized structures

(Hydrogen atoms have been omitted for clarity)

1) 1d - $BC_{16}H_{23}O_2$

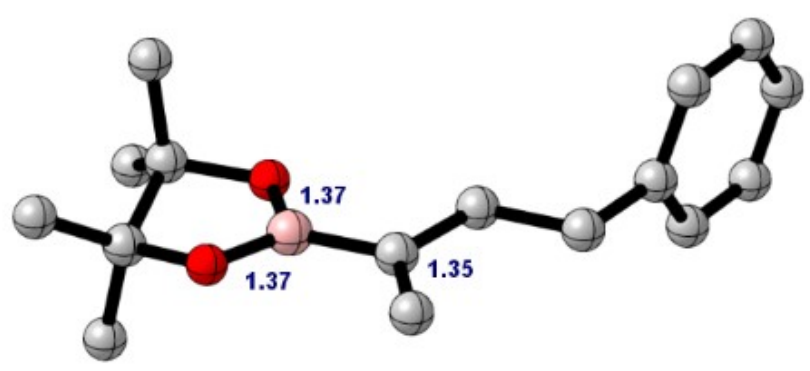

EE+Thermal Free Energy Correction =-798.119546 Hartree

E(RB3LYP) = -798.432214 Hartree

Imaginary Frequency = 0

5	-1.511932000	0.610820000	-0.064871000
8	-2.754126000	1.178268000	-0.186193000
8	-1.562700000	-0.746569000	0.116689000

6	-3.738518000	0.164815000	0.155607000
6	-2.936603000	-1.172763000	-0.095452000
6	-3.026235000	-1.666113000	-1.542534000
1	-2.285093000	-2.465136000	-1.692117000
1	-4.023596000	-2.069008000	-1.770732000
1	-2.802787000	-0.856294000	-2.252654000
6	-3.257884000	-2.305603000	0.871717000
1	-4.321441000	-2.580734000	0.803064000
1	-2.657370000	-3.191958000	0.617520000
1	-3.031543000	-2.028922000	1.909491000
6	-4.966567000	0.351785000	-0.727011000
1	-5.689145000	-0.461476000	-0.559402000
1	-5.459125000	1.304254000	-0.479880000
1	-4.700934000	0.373627000	-1.791776000
6	-4.103162000	0.379819000	1.627585000
1	-4.464448000	1.410700000	1.757280000
1	-4.895192000	-0.310298000	1.952505000
1	-3.227219000	0.241109000	2.278436000
6	1.112926000	0.605449000	-0.058764000
1	1.089394000	-0.015281000	0.855861000
1	1.107358000	-0.131426000	-0.883162000
6	2.429417000	1.394671000	-0.098474000
1	2.448996000	2.112141000	0.738784000
1	2.469740000	1.989351000	-1.026260000
6	-0.168899000	1.409036000	-0.124163000
6	-0.214763000	2.750007000	-0.228398000
1	-1.176039000	3.271456000	-0.269054000
1	0.678705000	3.380658000	-0.277456000
6	3.637664000	0.489774000	-0.021801000
6	4.183031000	0.119975000	1.219003000
6	4.211325000	-0.046411000	-1.186798000

6	5.270543000	-0.756325000	1.295247000
1	3.749918000	0.528170000	2.137204000
6	5.298868000	-0.923184000	-1.116282000
1	3.800591000	0.230907000	-2.162352000
6	5.832829000	-1.281820000	0.126488000
1	5.682402000	-1.027719000	2.271051000
1	5.732908000	-1.325699000	-2.035555000
1	6.684357000	-1.964789000	0.183771000

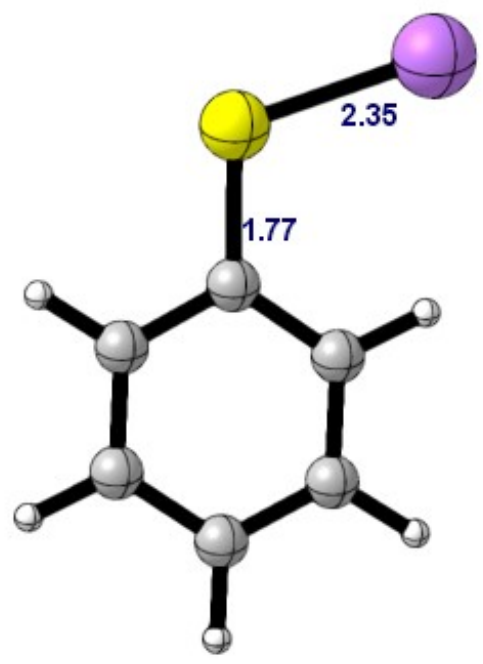

2) PhSLi -LiC_6H_5S

EE+Thermal Free Energy Correction =-637.127192 Hartree

E(RB3LYP) = -637.186736 Hartree

Imaginary Frequency = 0

6	1.567128000	1.321281000	0.000007000
6	2.427841000	0.216949000	0.000012000
6	1.869507000	-1.067833000	-0.000024000
6	0.483905000	-1.244416000	0.000010000
6	-0.406861000	-0.141900000	0.000141000
6	0.180399000	1.147230000	0.000037000
1	1.978885000	2.335323000	-0.000026000
1	3.512042000	0.354797000	-0.000020000
1	2.521601000	-1.946703000	-0.000075000

1	0.066158000	-2.254918000	-0.000037000
1	-0.463296000	2.031448000	0.000001000
16	-2.154654000	-0.425098000	-0.000020000
3	-3.290818000	1.631249000	-0.000205000

3) Togni-BEt_3 (6)- $BC_{14}H_{19}O_2IF_3$

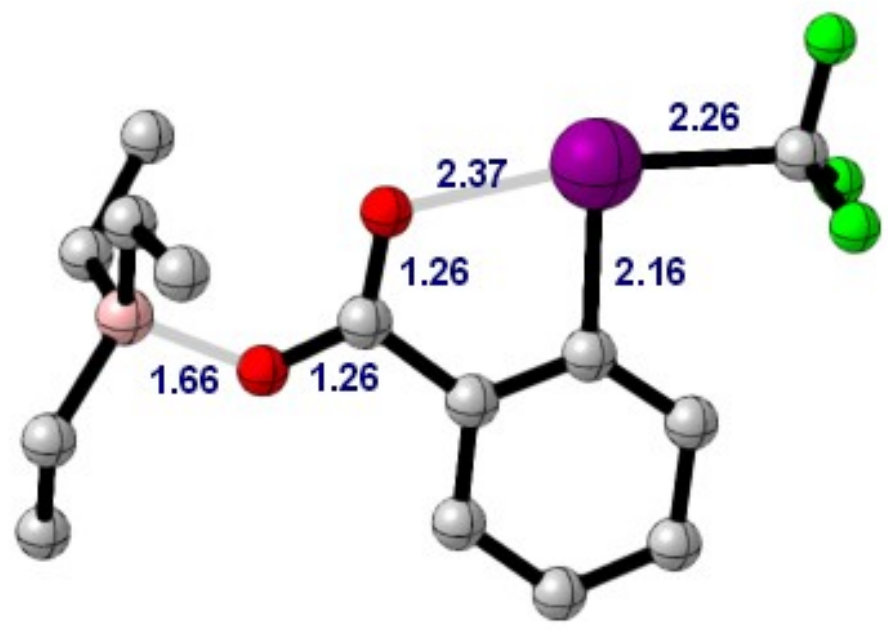

EE+Thermal Free Energy Correction =-1316.547104 Hartree

E(RB3LYP) = -1316.799082 Hartree

Imaginary Frequency = 0

53	-1.667849000	-0.918016000	-0.020885000
6	-3.884471000	-0.484893000	0.017715000
9	-4.227834000	0.209413000	1.097714000
9	-4.271343000	0.186268000	-1.062338000
9	-4.488188000	-1.666569000	0.042673000
6	-1.096235000	1.168127000	-0.032183000
6	-2.021581000	2.205250000	-0.030464000
6	-1.522227000	3.514740000	-0.033696000
6	-0.144787000	3.757066000	-0.037325000
6	0.754186000	2.690665000	-0.038640000
6	0.285294000	1.368394000	-0.037312000
6	1.226839000	0.197394000	-0.041053000
8	2.454878000	0.456311000	-0.030378000
8	0.704246000	-0.952962000	-0.053582000
1	-3.096356000	2.040582000	-0.027476000
1	-2.229494000	4.347009000	-0.033053000

1	0.228274000	4.783065000	-0.038918000
1	1.833313000	2.851895000	-0.040551000
5	3.782919000	-0.536755000	0.044588000
6	4.908604000	0.482219000	0.644590000
6	5.264259000	1.699695000	-0.218550000
1	5.827238000	-0.108000000	0.836316000
1	4.585863000	0.840779000	1.641191000
1	5.986040000	2.381042000	0.267732000
1	5.704373000	1.401280000	-1.185091000
1	4.363434000	2.294098000	-0.452177000
6	4.080762000	-0.976280000	-1.499009000
6	3.110105000	-1.962268000	-2.163307000
1	5.101364000	-1.410152000	-1.517984000
1	4.149001000	-0.068918000	-2.129341000
1	3.384343000	-2.193874000	-3.208847000
1	3.070516000	-2.921376000	-1.619809000
1	2.079801000	-1.570223000	-2.173562000
6	3.448698000	-1.777383000	1.047747000
6	2.995865000	-1.411489000	2.467694000
1	4.374895000	-2.384074000	1.112272000
1	2.690826000	-2.442351000	0.599822000
1	2.795039000	-2.297008000	3.098013000
1	3.749253000	-0.803913000	2.996744000
1	2.065093000	-0.817459000	2.450700000

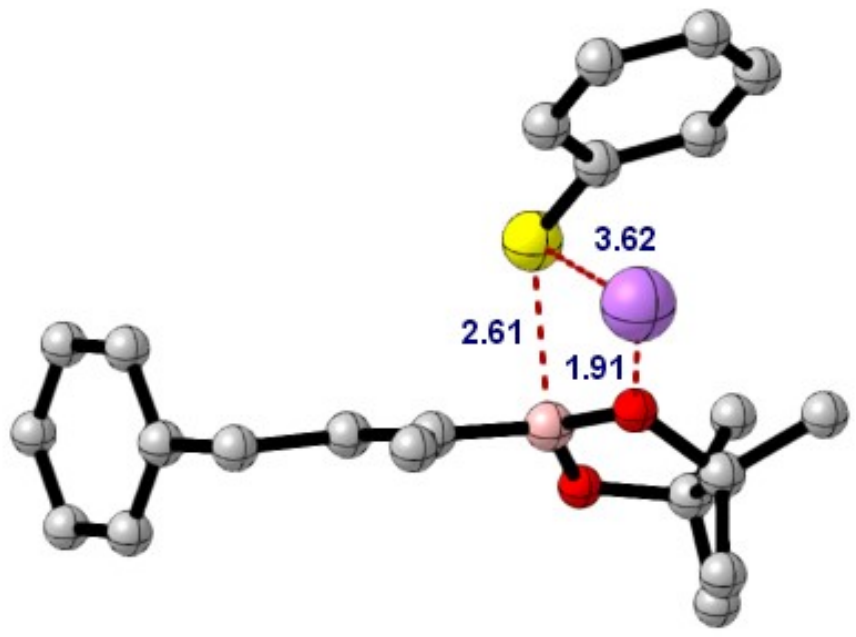

4) TS1-$LiBC_{22}H_{28}SO_2$

EE+Thermal Free Energy Correction =-1435.232227 Hartree

E(RB3LYP) = -1435.630394 Hartree

Imaginary Frequency = 1

5	-0.515633000	0.855071000	0.337837000
8	-0.352650000	1.947876000	-0.497246000
8	-1.851476000	0.821633000	0.833439000
6	-1.634915000	2.533711000	-0.794094000
6	-2.525923000	2.046192000	0.419368000
6	-3.969752000	1.725670000	0.048241000
1	-4.537165000	1.426555000	0.944173000
1	-4.462135000	2.613712000	-0.375669000
1	-4.024009000	0.908808000	-0.682616000
6	-2.480111000	2.991478000	1.623397000
1	-3.006333000	3.935197000	1.419767000
1	-2.967992000	2.503826000	2.481249000
1	-1.442240000	3.218437000	1.906620000
6	-2.109497000	2.006033000	-2.152615000
1	-3.060850000	2.468527000	-2.454227000
1	-1.348933000	2.256766000	-2.907647000
1	-2.207828000	0.913331000	-2.137965000
6	-1.460236000	4.049885000	-0.866693000
1	-0.833863000	4.303293000	-1.735138000
1	-2.432962000	4.550572000	-0.989684000
1	-0.968283000	4.446748000	0.030768000

6	2.037240000	0.221080000	0.515172000
1	1.919305000	-0.489016000	-0.321051000
1	2.264387000	1.190904000	0.034634000
6	3.237426000	-0.211948000	1.370622000
1	3.012192000	-1.182858000	1.843210000
1	3.392900000	0.510751000	2.188731000
6	0.698623000	0.342012000	1.207347000
6	0.525710000	0.122276000	2.525115000
1	-0.449412000	0.274809000	2.999855000
1	1.333627000	-0.190403000	3.194753000
6	4.499607000	-0.332515000	0.548371000
6	4.775130000	-1.507897000	-0.171395000
6	5.397318000	0.741646000	0.434422000
6	5.913718000	-1.609770000	-0.976510000
1	4.085634000	-2.354371000	-0.096865000
6	6.538309000	0.645259000	-0.369734000
1	5.199104000	1.665574000	0.986114000
6	6.800915000	-0.531871000	-1.078841000
1	6.110627000	-2.534965000	-1.524986000
1	7.225778000	1.492525000	-0.441411000
1	7.692666000	-0.610071000	-1.706204000
16	-0.604320000	-1.186078000	-1.280544000
6	-1.989879000	-2.072153000	-0.659239000
6	-3.281353000	-1.941338000	-1.237337000
6	-1.876546000	-2.959426000	0.445819000
6	-4.381474000	-2.644273000	-0.740163000
1	-3.405227000	-1.274429000	-2.093890000
6	-2.983119000	-3.664406000	0.940204000
1	-0.892168000	-3.101032000	0.900675000
6	-4.246044000	-3.511189000	0.354144000
1	-5.359195000	-2.514519000	-1.213776000

1	-2.851389000	-4.342184000	1.788887000
1	-5.108358000	-4.061058000	0.738812000
3	-2.636151000	-0.703448000	1.673083000

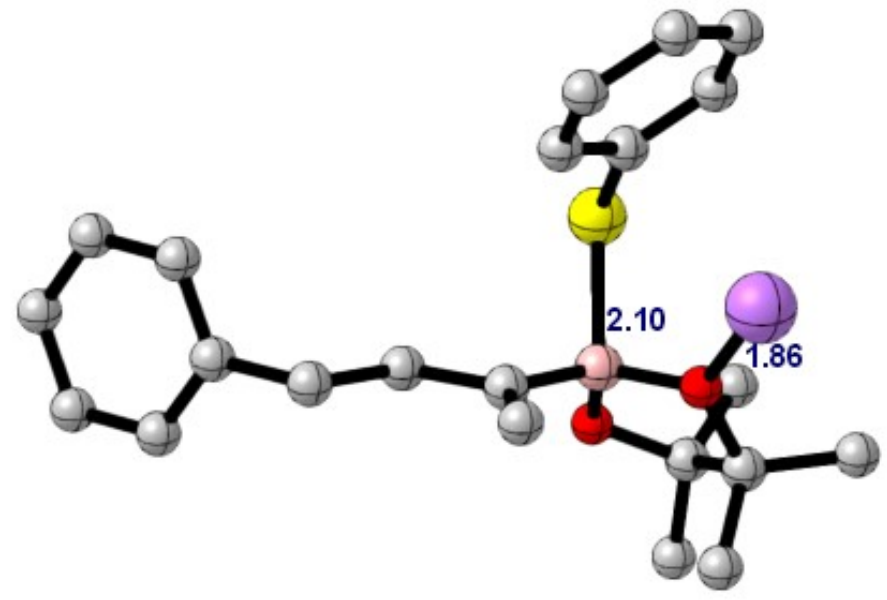

5)INT1-$LiBC_{22}H_{28}SO_2$

EE+Thermal Free Energy Correction =-1435.237771 Hartree

E(RB3LYP) = -1435.634460 Hartree

Imaginary Frequency = 0

5	-0.963586000	0.634012000	-0.008920000
8	-0.886853000	1.919553000	-0.644389000
8	-2.317240000	0.560349000	0.592796000
6	-2.179956000	2.519451000	-0.698353000
6	-2.912121000	1.874385000	0.539812000
6	-4.425127000	1.742606000	0.378672000
1	-4.873939000	1.323702000	1.294301000
1	-4.881896000	2.729688000	0.207960000
1	-4.686629000	1.089521000	-0.464828000
6	-2.587717000	2.588829000	1.858166000
1	-3.063337000	3.579178000	1.915328000
1	-2.960722000	1.979167000	2.695748000
1	-1.502320000	2.703097000	1.982200000
6	-2.852838000	2.166264000	-2.034519000
1	-3.810664000	2.693279000	-2.162851000
1	-2.180307000	2.468361000	-2.851994000
1	-3.017144000	1.084186000	-2.121130000
6	-2.014171000	4.036446000	-0.601287000

1	-1.518070000	4.410966000	-1.509965000
1	-2.991089000	4.537802000	-0.514224000
1	-1.393744000	4.317244000	0.260012000
6	1.645363000	0.421328000	0.383006000
1	1.673283000	-0.249508000	-0.494154000
1	1.764755000	1.433950000	-0.046143000
6	2.846961000	0.112782000	1.289610000
1	2.728991000	-0.899686000	1.711935000
1	2.858428000	0.811065000	2.142768000
6	0.263300000	0.333433000	0.990066000
6	0.061198000	0.063508000	2.292188000
1	-0.950880000	0.035206000	2.708512000
1	0.871758000	-0.126964000	3.004913000
6	4.156569000	0.198954000	0.541737000
6	4.631099000	-0.896456000	-0.200266000
6	4.902498000	1.388995000	0.518052000
6	5.815154000	-0.808701000	-0.938875000
1	4.062249000	-1.831327000	-0.195425000
6	6.087997000	1.482660000	-0.219147000
1	4.548694000	2.253289000	1.088292000
6	6.549161000	0.383164000	-0.951275000
1	6.167780000	-1.674640000	-1.505867000
1	6.654664000	2.417843000	-0.220362000
1	7.476082000	0.453498000	-1.526435000
16	-0.933459000	-0.793648000	-1.554406000
6	-1.295988000	-2.329052000	-0.728349000
6	-2.465655000	-3.051339000	-1.056793000
6	-0.435019000	-2.879634000	0.248049000
6	-2.768558000	-4.264241000	-0.426728000
1	-3.139240000	-2.645832000	-1.816313000
6	-0.746866000	-4.085735000	0.881985000

1	0.480208000	-2.348561000	0.510738000
6	-1.913980000	-4.786093000	0.550907000
1	-3.679985000	-4.802095000	-0.702827000
1	-0.065066000	-4.486728000	1.637501000
1	-2.151057000	-5.731362000	1.045615000
3	-3.067541000	-1.034857000	1.184865000

6) INT1-SN2 - $LiB_2C_{36}H_{47}SO_4IF_3$

EE+Thermal Free Energy Correction = -2751.793712 Hartree

E(RB3LYP) = -2752.474160 Hartree

Imaginary Frequency = 0

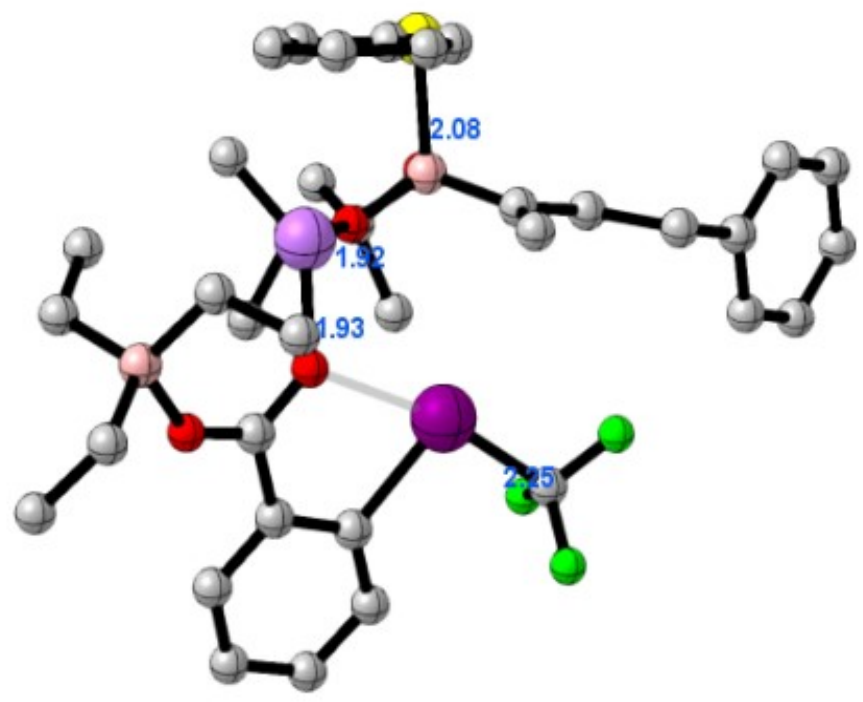

16	-1.330825000	3.875691000	0.725644000
8	0.012360000	1.268852000	1.169041000
8	-2.088497000	1.591453000	2.126295000
6	-5.777177000	0.145919000	-1.098884000
6	-6.824558000	1.079781000	-1.045976000
1	-6.626691000	2.121411000	-1.316212000
6	-8.112971000	0.695972000	-0.657942000
1	-8.915066000	1.438464000	-0.628029000
6	-8.375895000	-0.633833000	-0.311802000
1	-9.382293000	-0.936135000	-0.010719000
6	-7.340499000	-1.574846000	-0.357149000

1	-7.536203000	-2.617225000	-0.091023000
6	-6.054840000	-1.186520000	-0.745926000
1	-5.250269000	-1.926594000	-0.780169000
6	-4.372746000	0.564073000	-1.466812000
1	-4.400164000	1.446254000	-2.127647000
1	-3.893940000	-0.244769000	-2.038963000
6	-3.529236000	0.888420000	-0.224816000
1	-3.564927000	0.035654000	0.477837000
1	-4.010739000	1.707260000	0.337735000
6	-2.074247000	1.251689000	-0.428042000
6	0.152092000	1.034853000	2.596181000
6	-1.343226000	0.806094000	3.044642000
6	-1.485514000	1.121067000	-1.640658000
1	-2.022880000	0.793764000	-2.537416000
1	-0.454662000	1.433923000	-1.819364000
6	0.011283000	4.159229000	-0.411393000
6	-0.194742000	4.196827000	-1.804715000
1	-1.206365000	4.064120000	-2.192685000
6	0.877189000	4.387551000	-2.683254000
1	0.694233000	4.403961000	-3.761386000
6	2.177232000	4.555255000	-2.192545000
1	3.014379000	4.695403000	-2.880580000
6	2.394687000	4.542959000	-0.809598000
1	3.404013000	4.677888000	-0.412144000
6	1.324680000	4.349558000	0.071885000
1	1.498907000	4.341875000	1.149888000
6	0.787477000	2.275845000	3.238115000
1	0.950734000	2.129395000	4.315807000
1	0.160035000	3.161888000	3.078616000
1	1.768896000	2.463997000	2.775827000
6	1.053641000	-0.176542000	2.837821000

1	1.054367000	-0.433934000	3.907885000
1	2.092811000	0.043673000	2.553718000
1	0.726527000	-1.059458000	2.276896000
6	-1.644466000	1.282539000	4.466496000
1	-1.014886000	0.761013000	5.204446000
1	-2.698117000	1.072746000	4.706697000
1	-1.486338000	2.364252000	4.565851000
6	-1.797064000	-0.656984000	2.899431000
1	-1.329723000	-1.319994000	3.642668000
1	-1.576985000	-1.048233000	1.897946000
1	-2.887870000	-0.696181000	3.037004000
5	-1.346658000	1.807054000	0.914474000
53	-0.054351000	-1.529662000	-0.698707000
6	-1.790127000	-2.956089000	-0.835267000
9	-1.493900000	-4.022631000	-1.562670000
9	-2.159184000	-3.345807000	0.379610000
9	-2.789273000	-2.293694000	-1.404822000
6	1.353396000	-3.012856000	0.039368000
6	0.970224000	-4.309424000	0.368684000
6	1.949838000	-5.185504000	0.854100000
6	3.274921000	-4.764360000	0.997262000
6	3.632087000	-3.459205000	0.660643000
6	2.671590000	-2.555143000	0.180089000
6	3.057378000	-1.139609000	-0.143541000
8	4.274546000	-0.859691000	-0.123224000
8	2.104808000	-0.335913000	-0.404785000
1	-0.054440000	-4.660538000	0.268473000
1	1.661682000	-6.204893000	1.120296000
1	4.031008000	-5.455193000	1.375277000
1	4.659334000	-3.107750000	0.766240000
5	5.254430000	0.417089000	-0.617117000

6	6.450427000	-0.394181000	-1.373639000
6	7.315402000	-1.326885000	-0.517057000
1	7.102053000	0.358897000	-1.860531000
1	6.018849000	-0.979188000	-2.208237000
1	8.062817000	-1.885287000	-1.108833000
1	7.868501000	-0.773665000	0.260383000
1	6.694326000	-2.073561000	0.007220000
6	5.718206000	1.113625000	0.779761000
6	4.636066000	1.801010000	1.620278000
1	6.501761000	1.854008000	0.521933000
1	6.229123000	0.366680000	1.415213000
1	5.027840000	2.251993000	2.549224000
1	4.126285000	2.606121000	1.064022000
1	3.858066000	1.080250000	1.925562000
6	4.443679000	1.405583000	-1.623329000
6	3.881809000	0.813386000	-2.922356000
1	5.169604000	2.201740000	-1.885547000
1	3.638462000	1.957943000	-1.109504000
1	3.369656000	1.570000000	-3.543764000
1	4.676020000	0.366966000	-3.543097000
1	3.146528000	0.017753000	-2.717158000
3	1.600062000	1.459888000	0.098919000

7) TS2-SN2 - $LiB_2C_{36}H_{47}SO_4IF_3$

EE+Thermal Free Energy Correction = -2751.765272 Hartree

E(RB3LYP) = -2752.439937 Hartree

Imaginary Frequency = 1

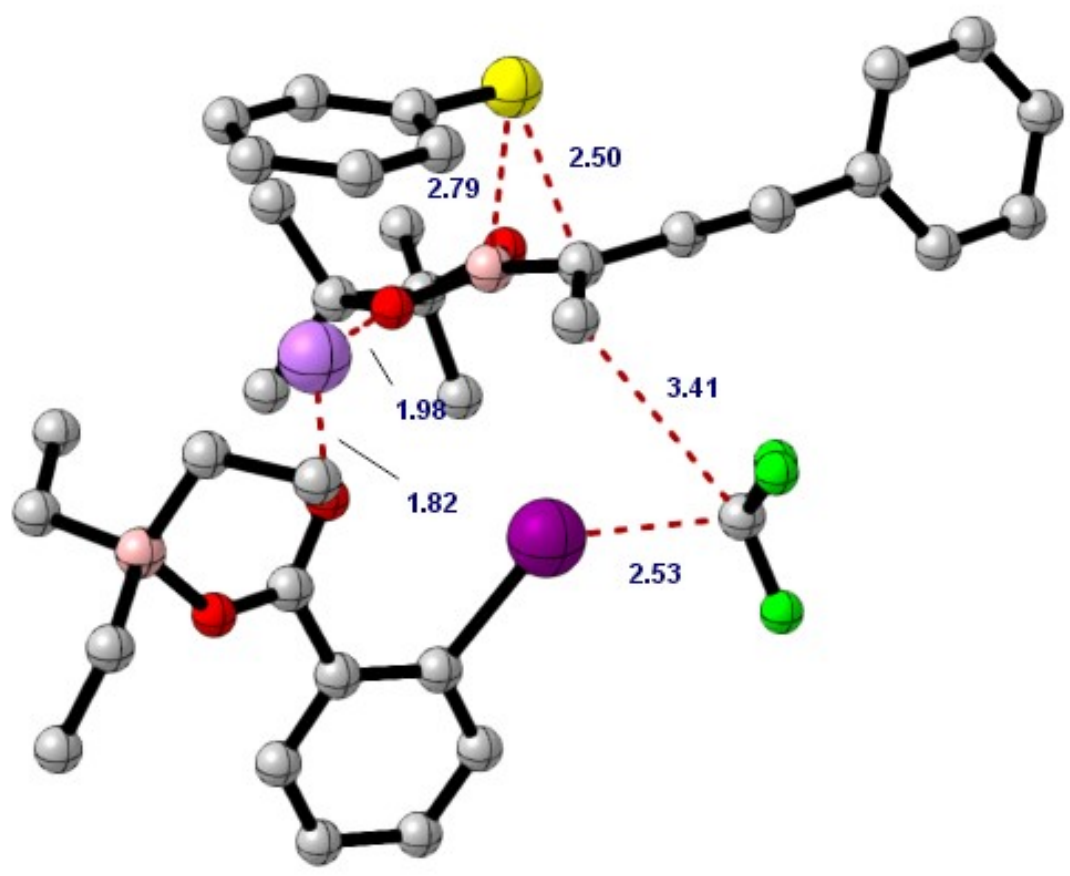

16	-2.383678000	-3.242985000	0.109340000
8	-0.007955000	-1.262840000	-1.484683000
8	-2.027184000	-0.895874000	-2.494965000
6	-5.781628000	0.036236000	1.022923000
6	-6.745074000	-0.965619000	0.823181000
1	-6.444001000	-2.016143000	0.877739000
6	-8.079064000	-0.636965000	0.559282000
1	-8.815551000	-1.431418000	0.411297000
6	-8.470960000	0.704342000	0.487463000
1	-9.513264000	0.963028000	0.283614000
6	-7.519154000	1.712472000	0.680187000
1	-7.816341000	2.763318000	0.626473000
6	-6.186907000	1.379620000	0.944315000
1	-5.444605000	2.169706000	1.088801000
6	-4.331531000	-0.315777000	1.258592000
1	-4.248044000	-1.303491000	1.739846000
1	-3.890422000	0.421425000	1.946009000
6	-3.534198000	-0.347370000	-0.053261000
1	-3.554518000	0.652302000	-0.520693000
1	-4.039669000	-1.007806000	-0.774719000
6	-2.079201000	-0.761670000	0.056125000
6	0.223484000	-1.443355000	-2.933723000
6	-1.040098000	-0.731626000	-3.544388000
6	-1.330619000	-0.449513000	1.185306000
1	-1.786428000	-0.064675000	2.099750000

1	-0.308909000	-0.809481000	1.301197000
6	-0.873395000	-3.782152000	0.823876000
6	-0.557096000	-3.481488000	2.168957000
1	-1.279854000	-2.929943000	2.773601000
6	0.665866000	-3.871940000	2.716690000
1	0.900248000	-3.616296000	3.752856000
6	1.591780000	-4.586863000	1.946121000
1	2.551861000	-4.880660000	2.375527000
6	1.279743000	-4.918351000	0.621156000
1	1.993186000	-5.483065000	0.015227000
6	0.064832000	-4.517167000	0.062153000
1	-0.170575000	-4.764362000	-0.973284000
6	0.254764000	-2.947420000	-3.198531000
1	0.458513000	-3.154242000	-4.258503000
1	-0.698054000	-3.418709000	-2.919418000
1	1.055895000	-3.406459000	-2.601591000
6	1.549056000	-0.796242000	-3.315263000
1	1.635604000	-0.747055000	-4.410859000
1	2.394487000	-1.394827000	-2.946172000
1	1.639935000	0.217410000	-2.905856000
6	-1.568984000	-1.365774000	-4.824380000
1	-0.800228000	-1.345814000	-5.611579000
1	-2.440564000	-0.797943000	-5.181960000
1	-1.883253000	-2.404599000	-4.661590000
6	-0.846225000	0.776724000	-3.732416000
1	-0.167126000	0.996551000	-4.568172000
1	-0.446421000	1.244451000	-2.820896000
1	-1.823367000	1.232700000	-3.948340000
5	-1.372972000	-1.056860000	-1.308169000
53	0.161924000	1.985502000	0.348053000
6	-1.990886000	2.894003000	1.325619000

9	-1.790630000	4.192569000	1.524988000
9	-3.008162000	2.745053000	0.473518000
9	-2.318234000	2.330624000	2.490924000
6	2.057279000	2.909062000	-0.453986000
6	1.981031000	4.225849000	-0.910221000
6	3.143300000	4.877971000	-1.340692000
6	4.375253000	4.216805000	-1.293910000
6	4.444597000	2.905708000	-0.819819000
6	3.285774000	2.228838000	-0.400040000
6	3.385365000	0.796969000	0.058631000
8	4.521075000	0.444931000	0.494069000
8	2.362082000	0.078294000	-0.025701000
1	1.023417000	4.752999000	-0.932326000
1	3.082344000	5.905595000	-1.708311000
1	5.283774000	4.724267000	-1.626225000
1	5.398514000	2.376659000	-0.775270000
5	5.109968000	-0.910598000	1.171164000
6	6.262669000	-0.322736000	2.175739000
6	7.339395000	0.570539000	1.546376000
1	6.750229000	-1.189037000	2.666585000
1	5.783511000	0.240137000	3.000751000
1	8.071517000	0.952154000	2.281478000
1	7.913342000	0.036381000	0.769838000
1	6.882678000	1.447173000	1.056277000
6	5.748528000	-1.773621000	-0.063223000
6	4.779535000	-2.288239000	-1.134532000
1	6.288347000	-2.634963000	0.379077000
1	6.530627000	-1.172388000	-0.564608000
1	5.284077000	-2.826010000	-1.957818000
1	4.033072000	-2.983827000	-0.712538000
1	4.220214000	-1.454583000	-1.592566000

6	3.956187000	-1.716372000	2.005041000
6	3.123323000	-0.918135000	3.017013000
1	4.492920000	-2.520004000	2.549293000
1	3.269218000	-2.269531000	1.339713000
1	2.410116000	-1.550215000	3.575952000
1	3.761390000	-0.413767000	3.761876000
1	2.529857000	-0.134389000	2.517917000
3	1.537620000	-1.529291000	-0.280477000

8) INT1-RE - $LiB_2C_{36}H_{47}SO_4IF_3$

EE+Thermal Free Energy Correction = -2751.805199 Hartree

E(RB3LYP) = -2752.483118 Hartree

Imaginary Frequency = 0

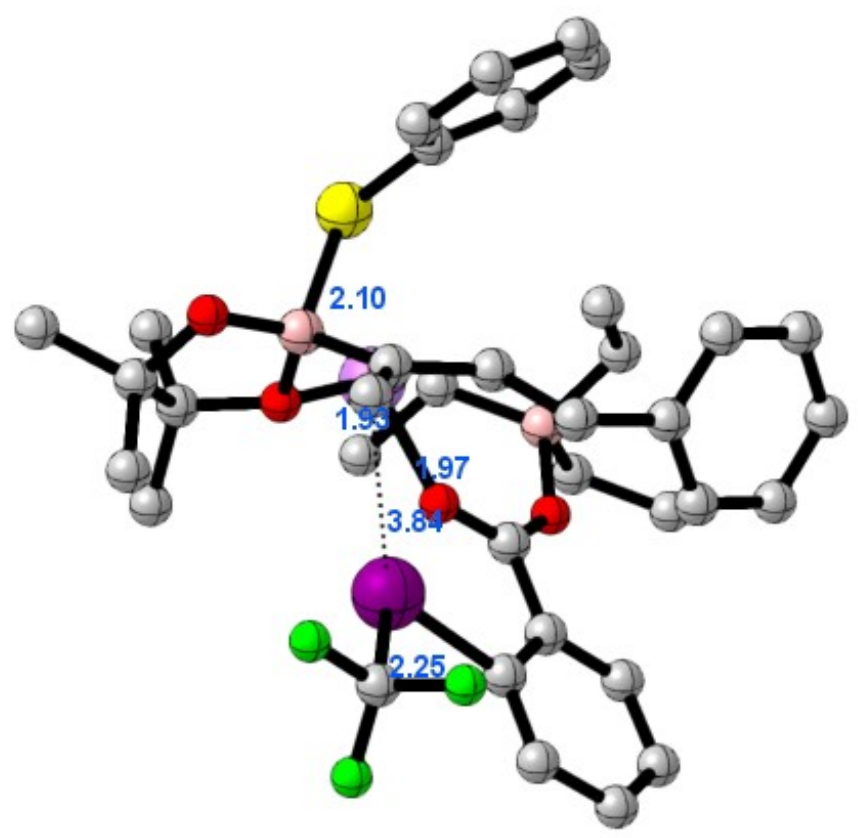

16	2.710413000	2.161886000	1.123765000
8	4.118745000	-0.339577000	0.513468000
8	1.958596000	-0.526682000	1.358176000
6	-1.117511000	1.483310000	-2.771355000
6	-1.121442000	2.882372000	-2.906576000
1	-0.176396000	3.407849000	-3.074185000
6	-2.309777000	3.609779000	-2.803792000
1	-2.290053000	4.698138000	-2.905411000
6	-3.520844000	2.951265000	-2.558039000
1	-4.449183000	3.520955000	-2.467502000

6	-3.532425000	1.559036000	-2.428609000
1	-4.470073000	1.031127000	-2.236106000
6	-2.340260000	0.834711000	-2.539804000
1	-2.359633000	-0.252794000	-2.436743000
6	0.186993000	0.720232000	-2.755995000
1	0.842709000	1.064326000	-3.573057000
1	0.001467000	-0.352859000	-2.929907000
6	0.895876000	0.929550000	-1.409177000
1	0.170023000	0.710546000	-0.608264000
1	1.085873000	2.005761000	-1.289971000
6	2.183579000	0.185539000	-1.139127000
6	4.109512000	-1.464118000	1.390960000
6	2.837192000	-1.213865000	2.285018000
6	2.797639000	-0.542105000	-2.090315000
1	2.410238000	-0.651332000	-3.109523000
1	3.737502000	-1.055864000	-1.871411000
6	2.805932000	3.295238000	-0.244396000
6	1.933678000	4.399144000	-0.314220000
1	1.196295000	4.550307000	0.476875000
6	1.980524000	5.276770000	-1.401003000
1	1.288018000	6.122210000	-1.439739000
6	2.894798000	5.069584000	-2.441030000
1	2.924730000	5.751327000	-3.294668000
6	3.772268000	3.980779000	-2.373541000
1	4.494609000	3.809759000	-3.176712000
6	3.737396000	3.106076000	-1.283638000
1	4.423043000	2.257904000	-1.240700000
6	5.424228000	-1.497417000	2.169723000
1	5.416014000	-2.294883000	2.929243000
1	5.619785000	-0.537457000	2.664952000
1	6.256045000	-1.694000000	1.476139000

6	3.988485000	-2.740732000	0.544668000
1	4.049162000	-3.653677000	1.155567000
1	4.810800000	-2.757376000	-0.185941000
1	3.049394000	-2.756032000	-0.024425000
6	3.124613000	-0.294252000	3.480496000
1	3.740672000	-0.804896000	4.234540000
1	2.175774000	-0.013164000	3.963901000
1	3.639732000	0.624124000	3.169403000
6	2.142339000	-2.476170000	2.790447000
1	2.810701000	-3.035424000	3.462794000
1	1.844162000	-3.148603000	1.976945000
1	1.237814000	-2.205710000	3.357715000
5	2.806811000	0.205138000	0.357744000
53	-0.113263000	-2.202693000	-0.220588000
6	0.339893000	-3.672957000	-1.861189000
9	-0.153687000	-4.878840000	-1.618067000
9	-0.145567000	-3.227476000	-3.012901000
9	1.661857000	-3.738416000	-1.930792000
6	-2.250150000	-2.250583000	-0.505773000
6	-2.856649000	-3.165089000	-1.361531000
6	-4.243149000	-3.078670000	-1.539923000
6	-4.988768000	-2.105993000	-0.868012000
6	-4.354501000	-1.214538000	-0.004265000
6	-2.965740000	-1.270439000	0.192858000
6	-2.292749000	-0.295181000	1.115218000
8	-3.034239000	0.534562000	1.684171000
8	-1.031090000	-0.395854000	1.241586000
1	-2.299338000	-3.936433000	-1.887394000
1	-4.733219000	-3.786051000	-2.212298000
1	-6.068516000	-2.044691000	-1.015815000
1	-4.916310000	-0.450467000	0.533561000

5	-2.862731000	1.798235000	2.743639000
6	-4.246965000	1.695263000	3.600912000
6	-5.556001000	1.923672000	2.835077000
1	-4.180777000	2.427429000	4.430159000
1	-4.295726000	0.706536000	4.096503000
1	-6.453464000	1.771724000	3.461110000
1	-5.617273000	2.945778000	2.425745000
1	-5.639978000	1.235054000	1.976567000
6	-2.787324000	3.103583000	1.771472000
6	-1.576313000	3.219667000	0.839393000
1	-2.833467000	3.997408000	2.425773000
1	-3.704274000	3.151045000	1.155252000
1	-1.609048000	4.118215000	0.199882000
1	-0.626339000	3.261370000	1.396964000
1	-1.510104000	2.360829000	0.150619000
6	-1.550645000	1.573732000	3.684077000
6	-1.456772000	0.261975000	4.475970000
1	-1.551371000	2.419359000	4.398657000
1	-0.614839000	1.743776000	3.117223000
1	-0.545065000	0.199829000	5.096512000
1	-2.318592000	0.144897000	5.152073000
1	-1.452914000	-0.616590000	3.808897000
3	0.508694000	0.631467000	1.905668000

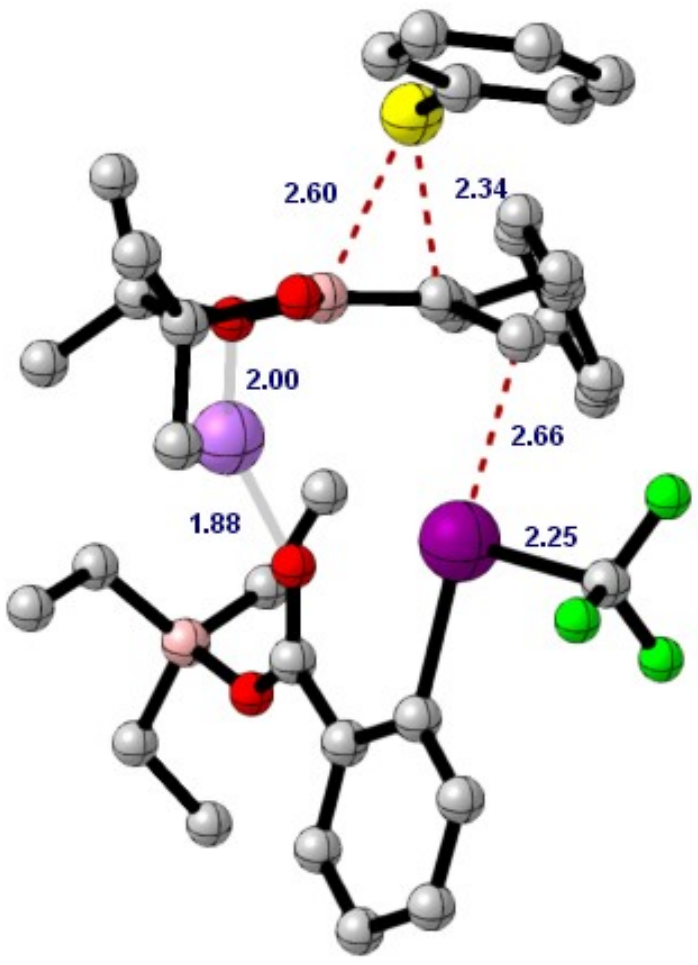

9) TS2-RE - $LiB_2C_{36}H47SO_4IF_3$

EE+Thermal Free Energy Correction = -2751.775140 Hartree

E(RB3LYP) = -2752.454945 Hartree

Imaginary Frequency = 1

16	3.714910000	1.969558000	0.531571000
8	2.539360000	-0.843721000	1.605273000
8	0.964818000	0.718929000	2.181429000
6	0.375558000	3.848893000	-2.197097000
6	0.544954000	5.091880000	-1.564333000
1	1.424536000	5.255574000	-0.934283000
6	-0.393064000	6.115951000	-1.728772000
1	-0.241696000	7.077215000	-1.230306000
6	-1.522435000	5.912307000	-2.530305000
1	-2.256901000	6.711371000	-2.659775000
6	-1.703316000	4.679028000	-3.165682000
1	-2.582559000	4.509942000	-3.793029000
6	-0.762037000	3.658299000	-2.997840000
1	-0.913328000	2.693935000	-3.492161000
6	1.348787000	2.719967000	-1.950661000
1	2.375972000	3.103083000	-1.847713000
1	1.339649000	2.031891000	-2.809905000

6	0.985518000	1.956162000	-0.666599000
1	-0.048946000	1.583136000	-0.792396000
1	0.937126000	2.668429000	0.172808000
6	1.869971000	0.778440000	-0.290815000
6	2.064205000	-1.224691000	2.919227000
6	1.428111000	0.112970000	3.446923000
6	2.184348000	-0.187480000	-1.287620000
1	2.280654000	0.120271000	-2.332990000
1	2.834606000	-1.019091000	-1.001093000
6	4.976793000	0.784328000	0.190898000
6	5.333618000	0.483448000	-1.139772000
1	4.834410000	1.008984000	-1.955919000
6	6.318682000	-0.470591000	-1.414350000
1	6.582116000	-0.691714000	-2.452364000
6	6.968766000	-1.136049000	-0.369254000
1	7.741111000	-1.878704000	-0.585238000
6	6.625224000	-0.840932000	0.956873000
1	7.128019000	-1.355215000	1.780529000
6	5.635637000	0.103682000	1.234973000
1	5.363097000	0.323597000	2.268399000
6	3.240912000	-1.732210000	3.742782000
1	2.925148000	-1.952845000	4.773809000
1	4.058290000	-1.001465000	3.770893000
1	3.630152000	-2.658894000	3.295564000
6	1.030982000	-2.338117000	2.714599000
1	0.688776000	-2.755189000	3.672705000
1	1.495611000	-3.145250000	2.129674000
1	0.155187000	-1.975925000	2.156148000
6	2.447718000	1.080779000	4.049189000
1	2.817836000	0.712755000	5.016468000
1	1.960361000	2.053670000	4.212004000

1	3.296657000	1.239194000	3.370631000
6	0.258281000	-0.072230000	4.403125000
1	0.612277000	-0.561717000	5.322636000
1	-0.547902000	-0.690164000	3.988208000
1	-0.161843000	0.905460000	4.685537000
5	1.861166000	0.272133000	1.188000000
53	-0.171854000	-1.411803000	-1.093864000
6	0.536408000	-2.668146000	-2.820270000
9	0.787470000	-3.908215000	-2.410102000
9	-0.402523000	-2.702235000	-3.756631000
9	1.639935000	-2.149318000	-3.326720000
6	-2.139654000	-2.582303000	-1.047021000
6	-2.323721000	-3.812400000	-1.679157000
6	-3.567458000	-4.453695000	-1.612772000
6	-4.627890000	-3.863024000	-0.920382000
6	-4.438970000	-2.637261000	-0.283854000
6	-3.192524000	-1.984385000	-0.325975000
6	-3.036756000	-0.695387000	0.439917000
8	-4.111722000	-0.123218000	0.772298000
8	-1.872582000	-0.286224000	0.710873000
1	-1.516258000	-4.300192000	-2.223334000
1	-3.699906000	-5.420334000	-2.105674000
1	-5.599191000	-4.360166000	-0.870595000
1	-5.252008000	-2.164366000	0.269182000
5	-4.496092000	1.203844000	1.635757000
6	-6.076476000	0.931113000	1.963180000
6	-7.005152000	0.758663000	0.754133000
1	-6.444616000	1.769783000	2.587038000
1	-6.169974000	0.033435000	2.605096000
1	-8.043751000	0.510052000	1.038356000
1	-7.051241000	1.673684000	0.139618000

1	-6.644798000	-0.048349000	0.093156000
6	-4.289869000	2.495682000	0.656309000
6	-2.873575000	2.874748000	0.205008000
1	-4.740220000	3.363154000	1.179648000
1	-4.911213000	2.359949000	-0.248799000
1	-2.848959000	3.761937000	-0.451303000
1	-2.215747000	3.112417000	1.060829000
1	-2.395836000	2.057914000	-0.360018000
6	-3.622214000	1.243078000	3.022283000
6	-3.521062000	-0.070148000	3.811554000
1	-4.116028000	2.000820000	3.660730000
1	-2.607213000	1.675911000	2.895267000
1	-2.976630000	0.039781000	4.765589000
1	-4.520877000	-0.466786000	4.049550000
1	-3.001243000	-0.853317000	3.233072000
3	-0.993693000	0.941548000	1.825341000

10) INT2-RE - $LiB_2C_{36}H_{47}SO_4IF_3$

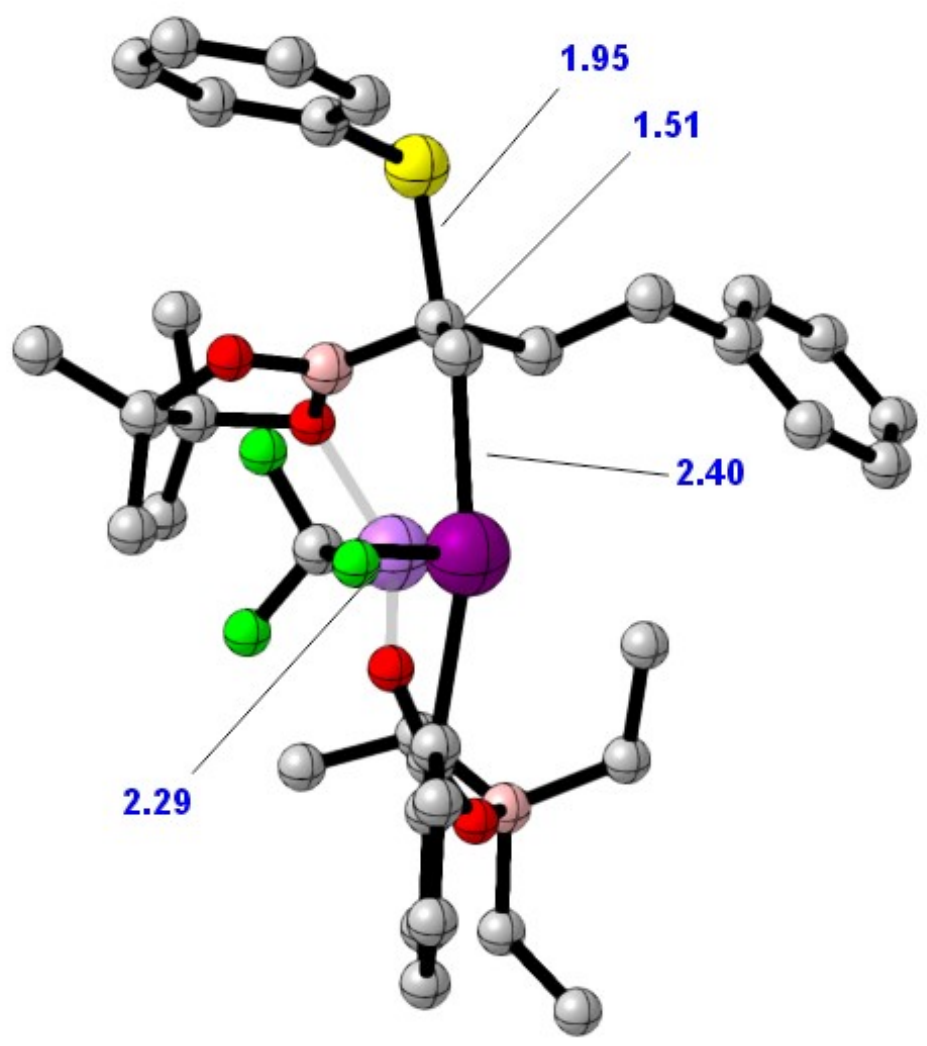

EE+Thermal Free Energy Correction = -2751.779763 Hartree

E(RB3LYP) = -2752.460917 Hartree

Imaginary Frequency = 0

16	3.536666000	-2.208345000	-0.308088000
8	2.579758000	0.754557000	-1.309115000
8	0.846638000	-0.488694000	-2.120126000
6	-0.161053000	-4.345470000	1.436543000
6	-0.248630000	-5.413387000	0.527196000
1	0.563610000	-5.576247000	-0.187819000
6	-1.354183000	-6.269320000	0.528596000
1	-1.402417000	-7.096466000	-0.184714000
6	-2.394746000	-6.071767000	1.444032000
1	-3.259014000	-6.740783000	1.447683000
6	-2.320326000	-5.011926000	2.353554000
1	-3.128906000	-4.847454000	3.070560000
6	-1.213737000	-4.156238000	2.345813000
1	-1.165770000	-3.325446000	3.056064000
6	1.011902000	-3.393204000	1.396587000
1	1.949163000	-3.950558000	1.246374000
1	1.099019000	-2.884193000	2.368338000
6	0.848545000	-2.350734000	0.273820000
1	-0.136933000	-1.876164000	0.416364000
1	0.780791000	-2.873004000	-0.695169000
6	1.916424000	-1.239458000	0.186367000
6	2.126982000	1.451141000	-2.501672000
6	1.340327000	0.322206000	-3.257752000
6	2.064789000	-0.424741000	1.448244000
1	2.143060000	-1.018841000	2.369294000
1	2.877309000	0.303219000	1.396994000
6	4.861007000	-1.044955000	-0.032382000
6	5.327396000	-0.792554000	1.269522000
1	4.863511000	-1.304039000	2.114832000
6	6.379020000	0.105168000	1.477478000

1	6.730349000	0.301034000	2.493742000
6	6.986091000	0.741684000	0.389127000
1	7.811185000	1.439354000	0.553156000
6	6.538875000	0.476760000	-0.910525000
1	7.011732000	0.967278000	-1.765144000
6	5.478572000	-0.408279000	-1.121067000
1	5.120449000	-0.606282000	-2.131967000
6	3.342626000	1.985718000	-3.246781000
1	3.037576000	2.449114000	-4.197169000
1	4.072911000	1.194601000	-3.456743000
1	3.839803000	2.751439000	-2.633456000
6	1.228804000	2.599377000	-2.035862000
1	0.898343000	3.216782000	-2.883301000
1	1.799578000	3.235507000	-1.347051000
1	0.341130000	2.226283000	-1.503424000
6	2.234019000	-0.606583000	-4.078664000
1	2.623814000	-0.096421000	-4.970553000
1	1.643091000	-1.474940000	-4.404884000
1	3.080128000	-0.977001000	-3.481738000
6	0.168415000	0.817547000	-4.091860000
1	0.536758000	1.485101000	-4.884687000
1	-0.565329000	1.374666000	-3.495475000
1	-0.343413000	-0.027664000	-4.577155000
5	1.776634000	-0.320421000	-1.092546000
53	-0.014367000	0.765045000	1.642402000
6	1.257396000	2.671243000	1.632409000
9	2.448453000	2.454039000	1.097037000
9	0.639398000	3.612846000	0.935161000
9	1.421732000	3.096253000	2.884959000
6	-1.760978000	2.327253000	1.644623000
6	-1.815797000	3.285962000	2.663693000

6	-2.916559000	4.139837000	2.791040000
6	-3.991809000	4.022621000	1.903370000
6	-3.961421000	3.046645000	0.908403000
6	-2.848529000	2.192961000	0.759412000
6	-2.913013000	1.149848000	-0.338101000
8	-4.092965000	0.750223000	-0.597802000
8	-1.865648000	0.760730000	-0.899351000
1	-0.988081000	3.383975000	3.371482000
1	-2.935738000	4.891003000	3.585693000
1	-4.856881000	4.684359000	1.993027000
1	-4.803166000	2.931280000	0.223115000
5	-4.741520000	-0.372474000	-1.553339000
6	-6.229229000	0.247282000	-1.855909000
6	-7.123869000	0.504965000	-0.636425000
1	-6.753301000	-0.436902000	-2.553312000
1	-6.121550000	1.197939000	-2.414221000
1	-8.081352000	0.992865000	-0.895766000
1	-7.374077000	-0.430383000	-0.107477000
1	-6.612817000	1.154489000	0.094893000
6	-4.817226000	-1.727609000	-0.636421000
6	-3.499035000	-2.385225000	-0.208932000
1	-5.426176000	-2.472037000	-1.188448000
1	-5.400335000	-1.504301000	0.276987000
1	-3.636092000	-3.249353000	0.463311000
1	-2.924052000	-2.757120000	-1.075847000
1	-2.846210000	-1.672985000	0.327157000
6	-3.902364000	-0.558369000	-2.949248000
6	-3.622197000	0.713774000	-3.760969000
1	-4.502056000	-1.250936000	-3.572474000
1	-2.956183000	-1.120038000	-2.806229000
1	-3.057941000	0.521802000	-4.691333000

1	-4.560263000	1.215286000	-4.048635000
1	-3.039614000	1.441705000	-3.172117000
3	-1.124616000	-0.611570000	-1.859342000

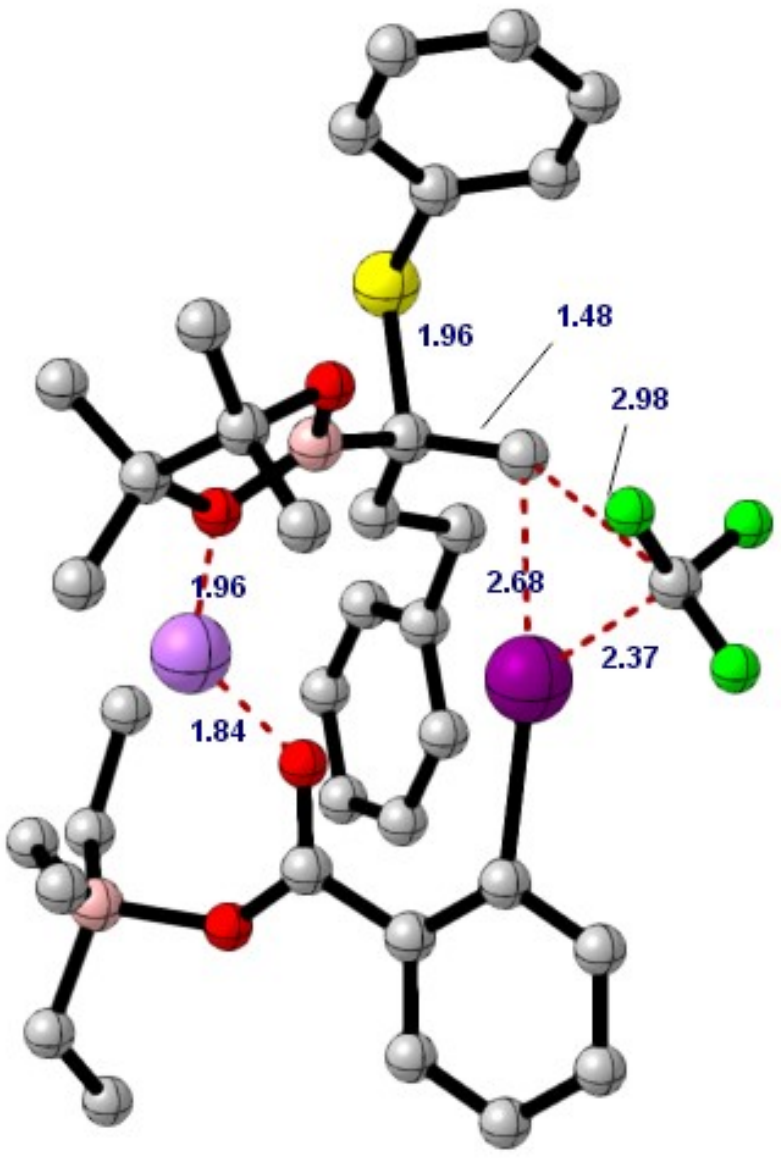

11) TS3-RE - $LiB_2C_{36}H_{47}SO_4IF_3$

EE+Thermal Free Energy Correction = -2751.758483 Hartree

E(RB3LYP) = -2752.439157 Hartree

Imaginary Frequency = 1

16	3.189246000	-1.735282000	2.021037000
8	2.998175000	-0.805617000	-1.204146000
8	0.960053000	-1.822015000	-1.069875000
6	-1.235151000	-0.710334000	3.496885000
6	-1.637216000	-1.815193000	4.260684000
1	-0.878088000	-2.469274000	4.699657000
6	-2.994780000	-2.097264000	4.453991000
1	-3.288889000	-2.965811000	5.049206000
6	-3.972437000	-1.279534000	3.879288000
1	-5.032222000	-1.506802000	4.018617000

6	-3.584405000	-0.169048000	3.120363000
1	-4.338729000	0.473620000	2.659242000
6	-2.229228000	0.112173000	2.936973000
1	-1.937095000	0.974937000	2.332231000
6	0.221005000	-0.459076000	3.169590000
1	0.882346000	-0.908909000	3.925714000
1	0.409333000	0.625359000	3.178488000
6	0.536362000	-1.029571000	1.774861000
1	-0.272284000	-0.685730000	1.113325000
1	0.450408000	-2.128448000	1.809899000
6	1.856406000	-0.630859000	1.097920000
6	2.651605000	-1.151725000	-2.571860000
6	1.545990000	-2.249816000	-2.362164000
6	2.193293000	0.808488000	1.161300000
1	2.128413000	1.314297000	2.130503000
1	3.056271000	1.111542000	0.565310000
6	4.750634000	-1.182992000	1.358701000
6	5.329473000	0.020368000	1.796622000
1	4.815829000	0.621088000	2.549553000
6	6.554297000	0.442512000	1.270456000
1	6.993703000	1.383882000	1.610137000
6	7.219701000	-0.341230000	0.321017000
1	8.179374000	-0.011925000	-0.085241000
6	6.655957000	-1.550355000	-0.103091000
1	7.171942000	-2.167239000	-0.843237000
6	5.424426000	-1.967453000	0.407838000
1	4.973047000	-2.900148000	0.064393000
6	3.905161000	-1.640350000	-3.284285000
1	3.658874000	-2.002861000	-4.293688000
1	4.399651000	-2.445436000	-2.726286000
1	4.618127000	-0.808473000	-3.381214000

6	2.116232000	0.126629000	-3.222377000
1	1.915739000	-0.018339000	-4.293297000
1	2.868157000	0.920727000	-3.108322000
1	1.193005000	0.467198000	-2.731678000
6	2.111232000	-3.649106000	-2.126074000
1	2.530077000	-4.067731000	-3.051858000
1	1.302266000	-4.309883000	-1.781538000
1	2.897026000	-3.637721000	-1.356703000
6	0.465719000	-2.270041000	-3.432110000
1	0.923853000	-2.450087000	-4.415957000
1	-0.090437000	-1.325464000	-3.478322000
1	-0.250214000	-3.084507000	-3.245526000
5	1.951493000	-1.115270000	-0.398499000
53	0.106073000	2.033036000	0.009795000
6	1.973231000	3.485327000	-0.119631000
9	2.894139000	3.016889000	-0.956586000
9	1.490608000	4.624234000	-0.613540000
9	2.526286000	3.736439000	1.058303000
6	-2.137338000	2.624901000	-0.543480000
6	-2.385694000	3.997854000	-0.635134000
6	-3.684906000	4.474631000	-0.849289000
6	-4.750053000	3.571482000	-0.960277000
6	-4.509956000	2.200744000	-0.867338000
6	-3.201890000	1.715787000	-0.661673000
6	-2.963911000	0.234392000	-0.618011000
8	-3.999374000	-0.492596000	-0.724790000
8	-1.785232000	-0.197685000	-0.506277000
1	-1.561791000	4.714751000	-0.538254000
1	-3.867761000	5.550527000	-0.925376000
1	-5.766925000	3.937766000	-1.121337000
1	-5.328368000	1.484625000	-0.960035000

5	-4.268660000	-2.047022000	-1.067100000
6	-5.828120000	-2.009050000	-1.563234000
6	-6.845679000	-1.490895000	-0.538844000
1	-6.116665000	-3.032394000	-1.875643000
1	-5.907681000	-1.393187000	-2.480628000
1	-7.872113000	-1.419932000	-0.942520000
1	-6.894923000	-2.140780000	0.351425000
1	-6.564959000	-0.485693000	-0.180108000
6	-4.093665000	-2.929894000	0.297015000
6	-2.684288000	-3.111022000	0.868626000
1	-4.524315000	-3.929883000	0.090508000
1	-4.727866000	-2.500414000	1.093614000
1	-2.652502000	-3.747863000	1.768445000
1	-2.008520000	-3.598232000	0.138332000
1	-2.255632000	-2.144028000	1.176818000
6	-3.286688000	-2.513336000	-2.300677000
6	-3.109880000	-1.523758000	-3.459885000
1	-3.737830000	-3.444289000	-2.695011000
1	-2.284921000	-2.866280000	-1.975267000
1	-2.539296000	-1.946660000	-4.305121000
1	-4.085920000	-1.198306000	-3.854740000
1	-2.577020000	-0.612558000	-3.139677000
3	-0.969776000	-1.838348000	-0.711877000

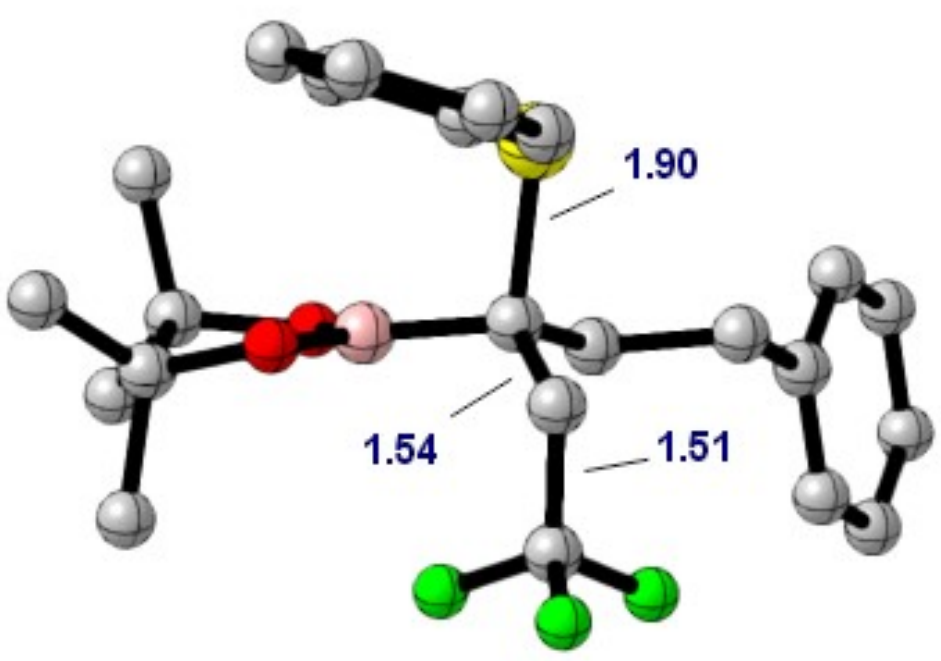

12) Product-3da - $BC_{23}H_{28}SO_2F_3$

EE+Thermal Free Energy Correction = -1765.034778 Hartree

E(RB3LYP) = -1765.444820 Hartree

Imaginary Frequency = 0

16	0.382044000	-1.421242000	-1.533222000
9	-0.085897000	0.785092000	2.585823000
9	0.017723000	-1.120136000	3.617603000
9	-1.808565000	-0.526473000	2.623342000
8	2.039736000	0.877610000	0.381901000
8	0.402022000	2.046762000	-0.700053000
6	-4.076129000	-0.640532000	-0.519684000
6	-4.679023000	-0.628076000	-1.787226000
1	-4.135745000	-1.039470000	-2.643166000
6	-5.963101000	-0.101486000	-1.967067000
1	-6.417269000	-0.104848000	-2.961628000
6	-6.665013000	0.425689000	-0.877946000
1	-7.668900000	0.835515000	-1.016149000
6	-6.073492000	0.422439000	0.391049000
1	-6.615267000	0.830454000	1.248642000
6	-4.790726000	-0.104705000	0.566006000
1	-4.330613000	-0.104325000	1.558583000
6	-2.672037000	-1.163790000	-0.322796000
1	-2.421307000	-1.883420000	-1.116729000
1	-2.623981000	-1.707893000	0.631819000
6	-1.643129000	-0.017852000	-0.316458000
1	-1.944443000	0.725215000	0.439970000
1	-1.687649000	0.516563000	-1.278972000
6	-0.175871000	-0.398425000	-0.032342000
6	2.535485000	2.243258000	0.302408000
6	1.596193000	2.874613000	-0.803197000
6	0.039480000	-1.197631000	1.261591000
1	-0.443882000	-2.185137000	1.225797000

1	1.113176000	-1.365411000	1.418951000
6	-0.460478000	-0.513330000	2.516972000
6	1.976358000	-2.089171000	-1.060847000
6	2.051408000	-3.311842000	-0.372421000
1	1.131879000	-3.837374000	-0.106620000
6	3.295501000	-3.851236000	-0.031993000
1	3.344967000	-4.799964000	0.508160000
6	4.471810000	-3.181999000	-0.387791000
1	5.443836000	-3.605776000	-0.123306000
6	4.401565000	-1.973054000	-1.089090000
1	5.317062000	-1.448723000	-1.374183000
6	3.159747000	-1.427761000	-1.425026000
1	3.102854000	-0.484243000	-1.967529000
6	4.015513000	2.206936000	-0.057554000
1	4.402084000	3.227248000	-0.202077000
1	4.199151000	1.627760000	-0.971184000
1	4.582076000	1.736955000	0.760158000
6	2.337633000	2.862404000	1.688544000
1	2.744421000	3.882770000	1.735370000
1	2.861714000	2.242596000	2.430915000
1	1.274203000	2.889194000	1.963810000
6	2.129467000	2.697745000	-2.226828000
1	3.011492000	3.327829000	-2.409867000
1	1.341946000	2.982937000	-2.939690000
1	2.397178000	1.649835000	-2.424061000
6	1.206792000	4.326970000	-0.558445000
1	2.101901000	4.966978000	-0.535662000
1	0.663800000	4.446084000	0.387870000
1	0.554584000	4.677935000	-1.372105000
5	0.767381000	0.868859000	-0.114085000

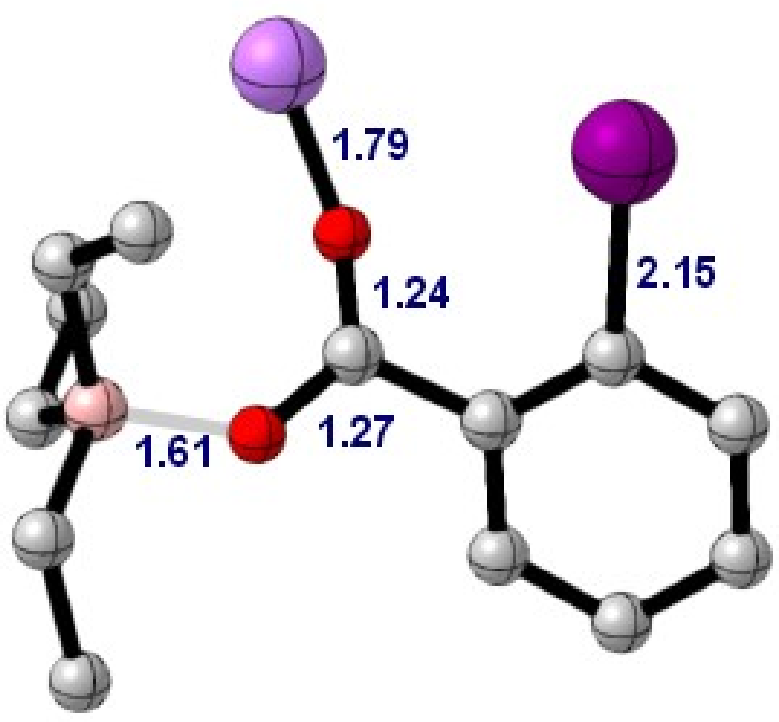

13) Biproduct -5-$LiBC_{13}H_{19}O_2I$

EE+Thermal Free Energy Correction = -986.899203 Hartree

E(RB3LYP) = -987.143977 Hartree

Imaginary Frequency = 0

53	2.666955000	-1.132348000	-0.155041000
6	1.909720000	0.863278000	0.067579000
6	2.860277000	1.886029000	-0.046694000
6	2.463168000	3.223763000	0.030060000
6	1.113306000	3.543438000	0.199316000
6	0.171296000	2.520549000	0.292842000
6	0.546163000	1.164203000	0.244710000
6	-0.550766000	0.136721000	0.412675000
8	-1.653128000	0.464265000	-0.130814000
8	-0.325465000	-0.897033000	1.063302000
1	3.913013000	1.643116000	-0.200391000
1	3.215380000	4.012155000	-0.051628000
1	0.794067000	4.586701000	0.252575000
1	-0.888463000	2.752807000	0.411153000
5	-3.071595000	-0.304811000	-0.197498000
6	-3.811591000	0.439292000	-1.456415000
6	-3.963057000	1.962978000	-1.357934000
1	-4.813590000	-0.018533000	-1.580725000

1	-3.271068000	0.204436000	-2.394647000
1	-4.432411000	2.413159000	-2.252022000
1	-4.578015000	2.258022000	-0.490375000
1	-2.979146000	2.445535000	-1.227724000
6	-3.846294000	-0.017843000	1.216810000
6	-3.257473000	-0.605013000	2.507093000
1	-4.882345000	-0.394820000	1.095194000
1	-3.958738000	1.074803000	1.355634000
1	-3.874356000	-0.386683000	3.398329000
1	-3.160407000	-1.703498000	2.449088000
1	-2.248282000	-0.209199000	2.706328000
6	-2.842218000	-1.896559000	-0.511068000
6	-1.877361000	-2.243820000	-1.653035000
1	-3.841259000	-2.312162000	-0.751834000
1	-2.532694000	-2.446415000	0.398145000
1	-1.806797000	-3.329983000	-1.847013000
1	-2.183592000	-1.767986000	-2.599580000
1	-0.854557000	-1.890000000	-1.438181000
3	-0.423223000	-2.597625000	1.612267000

14) 1d-Li-[PhS-BEt_3] - LiB2C28H43SO2

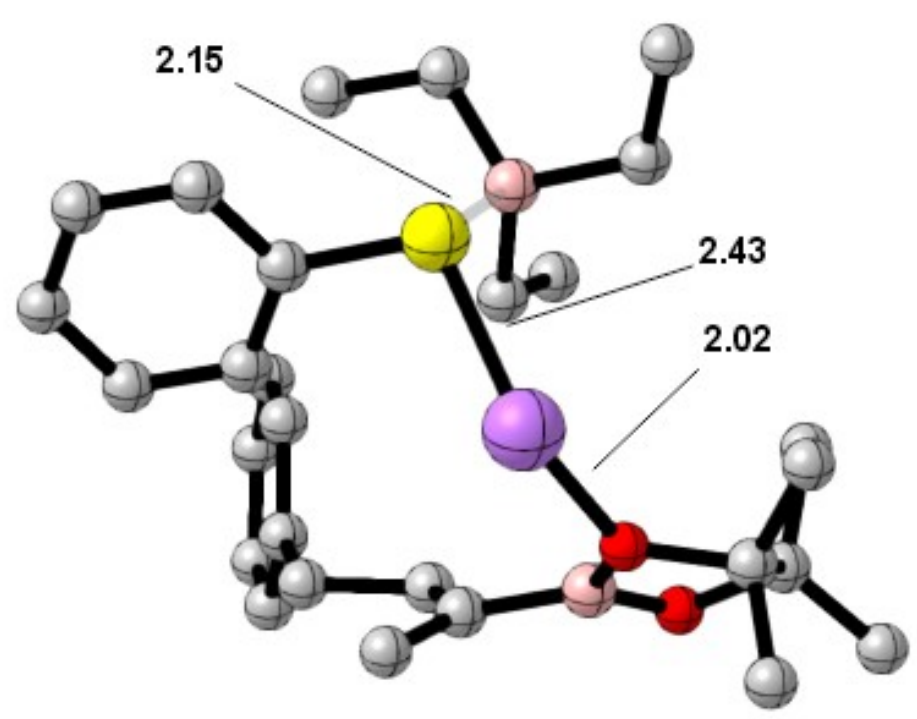

EE+Thermal Free Energy Correction = -1697.458496 Hartree

E(RB3LYP) = -1698.042743 Hartree

Imaginary Frequency = 0

5	-1.912528000	-1.523562000	-1.003001000
8	-2.497908000	-2.429531000	-0.176737000
8	-2.718944000	-0.409023000	-1.218815000
6	-3.734361000	-1.859792000	0.344307000
6	-4.065819000	-0.751604000	-0.725877000
6	-4.737287000	0.492981000	-0.163557000
1	-4.951766000	1.207638000	-0.972944000
1	-5.693085000	0.221581000	0.308661000
1	-4.109711000	0.993850000	0.584474000
6	-4.838202000	-1.283068000	-1.933859000
1	-5.878165000	-1.519327000	-1.667764000
1	-4.847939000	-0.513427000	-2.719622000
1	-4.367096000	-2.187925000	-2.345065000
6	-3.413547000	-1.290303000	1.727854000
1	-4.319303000	-0.913028000	2.223667000
1	-2.985432000	-2.090773000	2.348507000
1	-2.679426000	-0.476001000	1.670846000
6	-4.773980000	-2.968218000	0.448462000
1	-4.466688000	-3.689077000	1.220382000
1	-5.751014000	-2.552509000	0.737576000
1	-4.886468000	-3.511085000	-0.498788000
6	0.551918000	-2.382042000	-0.788333000
1	0.497571000	-1.953557000	0.224573000
1	0.272973000	-3.443852000	-0.656544000
6	2.004488000	-2.285574000	-1.280543000
1	2.221985000	-1.237231000	-1.540115000
1	2.133439000	-2.874810000	-2.202776000
6	-0.498570000	-1.700006000	-1.638934000

6	-0.282905000	-1.249841000	-2.888844000
1	-1.079332000	-0.760691000	-3.459287000
1	0.679186000	-1.348087000	-3.401161000
6	2.986128000	-2.743179000	-0.226413000
6	3.368953000	-1.867941000	0.805714000
6	3.496531000	-4.050365000	-0.216337000
6	4.238077000	-2.286655000	1.816870000
1	2.977723000	-0.846550000	0.815359000
6	4.367854000	-4.473889000	0.794032000
1	3.209279000	-4.744489000	-1.011837000
6	4.741367000	-3.593289000	1.814642000
1	4.525038000	-1.589227000	2.608463000
1	4.757467000	-5.495430000	0.782378000
1	5.422969000	-3.922049000	2.603437000
16	-0.457897000	2.999508000	-0.333316000
6	1.097910000	2.901177000	-1.208667000
6	1.876481000	4.061213000	-1.382706000
6	1.582177000	1.687749000	-1.733289000
6	3.107192000	4.005630000	-2.044057000
1	1.512308000	5.009831000	-0.980789000
6	2.814087000	1.633545000	-2.392000000
1	0.988133000	0.780893000	-1.620388000
6	3.586031000	2.790629000	-2.548506000
1	3.697819000	4.918380000	-2.162740000
1	3.171619000	0.678374000	-2.787636000
1	4.549521000	2.747277000	-3.062694000
3	-1.910779000	1.423714000	-1.478460000
5	-0.219819000	2.095542000	1.597343000
6	-1.709460000	2.353822000	2.219127000
6	-2.099032000	3.795351000	2.571128000
1	-1.774307000	1.738036000	3.138660000
1	-2.478946000	1.926575000	1.546544000

1	-3.117825000	3.875837000	2.992087000
1	-1.406637000	4.226277000	3.313658000
1	-2.057194000	4.449244000	1.683641000
6	0.963231000	2.891737000	2.386748000
6	2.415708000	2.551929000	2.022101000
1	0.814827000	2.679068000	3.466900000
1	0.814568000	3.983879000	2.296016000
1	3.150507000	3.116443000	2.625013000
1	2.632681000	1.480864000	2.174344000
1	2.627013000	2.768245000	0.964115000
6	0.090418000	0.519266000	1.334297000
6	0.268855000	-0.308044000	2.616423000
1	0.997019000	0.409127000	0.714102000
1	-0.722907000	0.068904000	0.736341000
1	0.501989000	-1.369197000	2.414184000
1	1.088491000	0.087287000	3.239206000
1	-0.641101000	-0.291339000	3.239476000

15) BEt_3 - BC6H15

EE+Thermal Free Energy Correction = -262.201974 Hartree

E(RB3LYP) = -262.365489 Hartree

Imaginary Frequency = 0

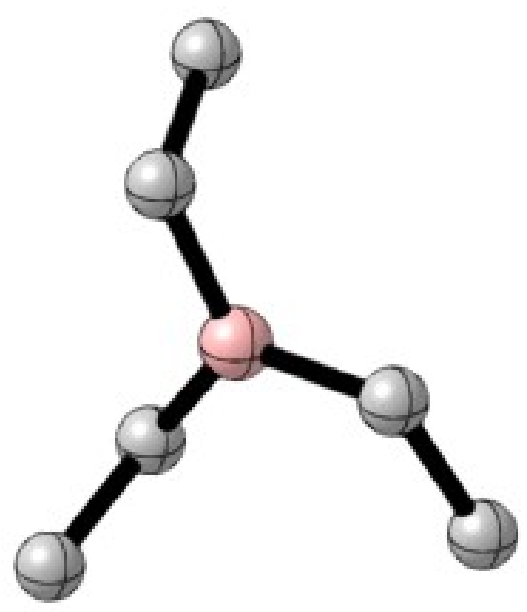

5	-0.170962000	-0.054789000	-0.119489000
6	-1.656786000	0.012617000	-0.661439000

6	-2.623481000	-0.392035000	0.475595000
1	-1.816820000	-0.657586000	-1.524727000
1	-1.907026000	1.038408000	-0.989231000
1	-3.676483000	-0.344427000	0.152283000
1	-2.433439000	-1.422775000	0.819953000
1	-2.518243000	0.270026000	1.351480000
6	0.698246000	-1.333191000	-0.439963000
6	2.024264000	-1.533347000	0.300637000
1	0.876472000	-1.269116000	-1.535762000
1	0.052860000	-2.228404000	-0.344008000
1	2.559981000	-2.436987000	-0.034292000
1	2.701865000	-0.676491000	0.152287000
1	1.863999000	-1.630046000	1.387434000
6	0.413920000	1.177551000	0.680358000
6	1.188206000	2.077540000	-0.313158000
1	1.102639000	0.862119000	1.483258000
1	-0.387848000	1.774708000	1.149423000
1	1.607644000	2.966520000	0.186063000
1	2.027428000	1.537207000	-0.781932000
1	0.535572000	2.435977000	-1.126968000

15) 1d-Li - LiBC16H23O2

EE+Thermal Free Energy Correction = -805.588811 Hartree

E(RB3LYP) = -805.902223 Hartree

Imaginary Frequency = 0

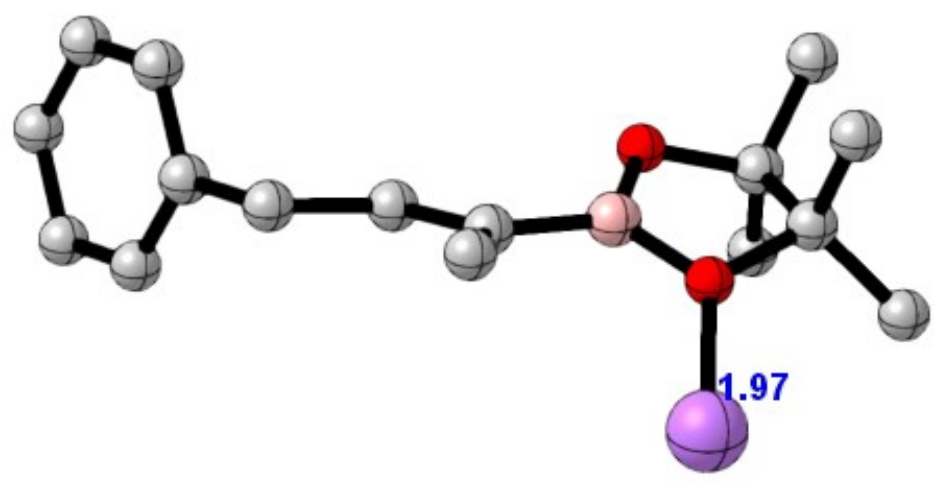

5	-1.444130000	0.544422000	0.074666000
8	-2.664960000	1.055781000	-0.366916000
8	-1.524921000	-0.775029000	0.384403000
6	-3.694347000	0.045171000	-0.035909000
6	-2.834732000	-1.272375000	-0.011627000
6	-2.662490000	-1.908101000	-1.393291000
1	-1.882525000	-2.680582000	-1.331304000
1	-3.592774000	-2.381371000	-1.737936000
1	-2.347172000	-1.163745000	-2.139539000
6	-3.296519000	-2.308394000	1.004410000
1	-4.330018000	-2.620962000	0.791610000
1	-2.650760000	-3.196661000	0.944743000
1	-3.248064000	-1.920504000	2.029906000
6	-4.790490000	0.063211000	-1.090818000
1	-5.483463000	-0.773872000	-0.920031000
1	-5.373068000	0.995167000	-1.024221000
1	-4.386251000	-0.025573000	-2.107793000
6	-4.251768000	0.435578000	1.332726000
1	-4.650421000	1.459496000	1.280863000
1	-5.067033000	-0.237112000	1.633860000
1	-3.471203000	0.408619000	2.106899000
6	1.164680000	0.597561000	0.039990000
1	1.126991000	-0.278694000	0.712222000
1	1.185719000	0.159127000	-0.975380000
6	2.472029000	1.364978000	0.281877000
1	2.461013000	1.786844000	1.300727000
1	2.532644000	2.217630000	-0.414584000
6	-0.127134000	1.371507000	0.206866000
6	-0.184859000	2.678808000	0.524301000
1	-1.142143000	3.187016000	0.679100000

1	0.705984000	3.295761000	0.676363000
6	3.685490000	0.479316000	0.110854000
6	4.164487000	-0.294085000	1.181588000
6	4.328472000	0.366670000	-1.132667000
6	5.255584000	-1.153103000	1.016844000
1	3.676762000	-0.218157000	2.158169000
6	5.420543000	-0.491000000	-1.302576000
1	3.970404000	0.962448000	-1.977734000
6	5.887893000	-1.254803000	-0.227516000
1	5.615692000	-1.742992000	1.863966000
1	5.910186000	-0.560814000	-2.277589000
1	6.742572000	-1.923570000	-0.357606000
3	-2.928137000	2.652913000	-1.486088000

X. NMR Spectra

4,4,5,5-tetramethyl-2-(4-phenylbut-1-en-2-yl)-1,3,2-dioxaborolane (1d)

^{1}H NMR (500 MHz, Chloroform-*d*)

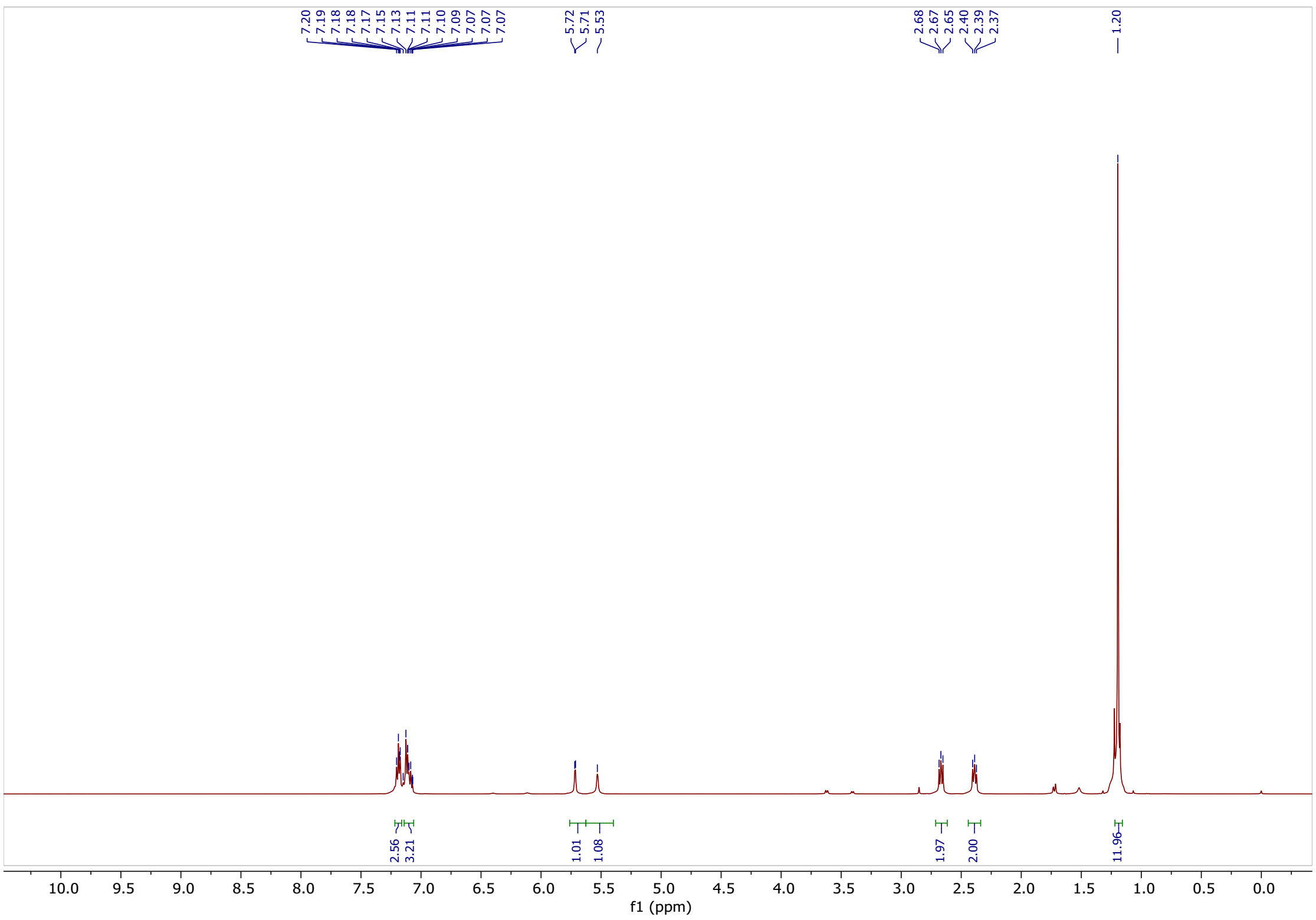

^{13}C NMR (125 MHz, $CDCl_3$)

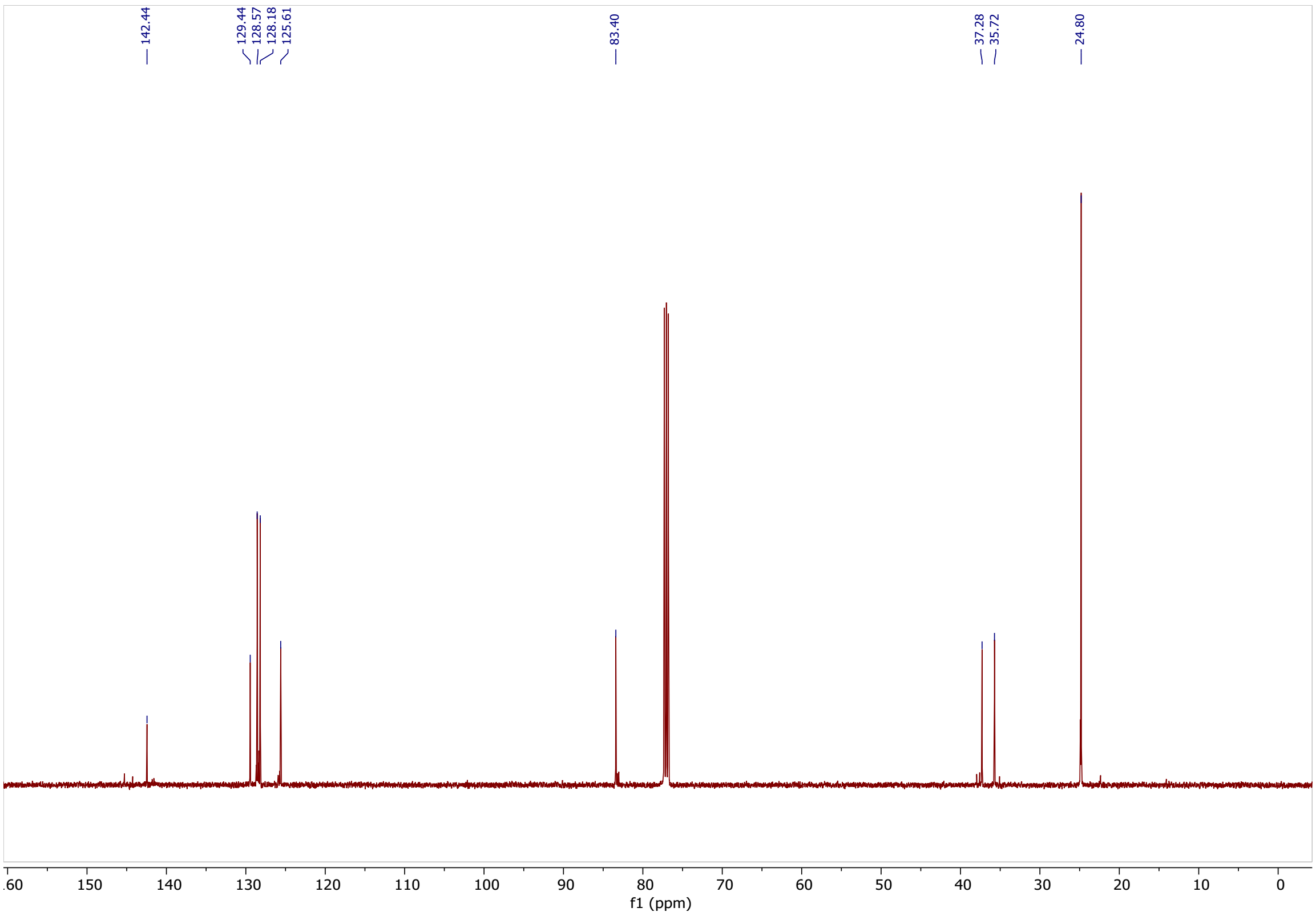

^{11}B NMR (160 MHz, $CDCl_3$)

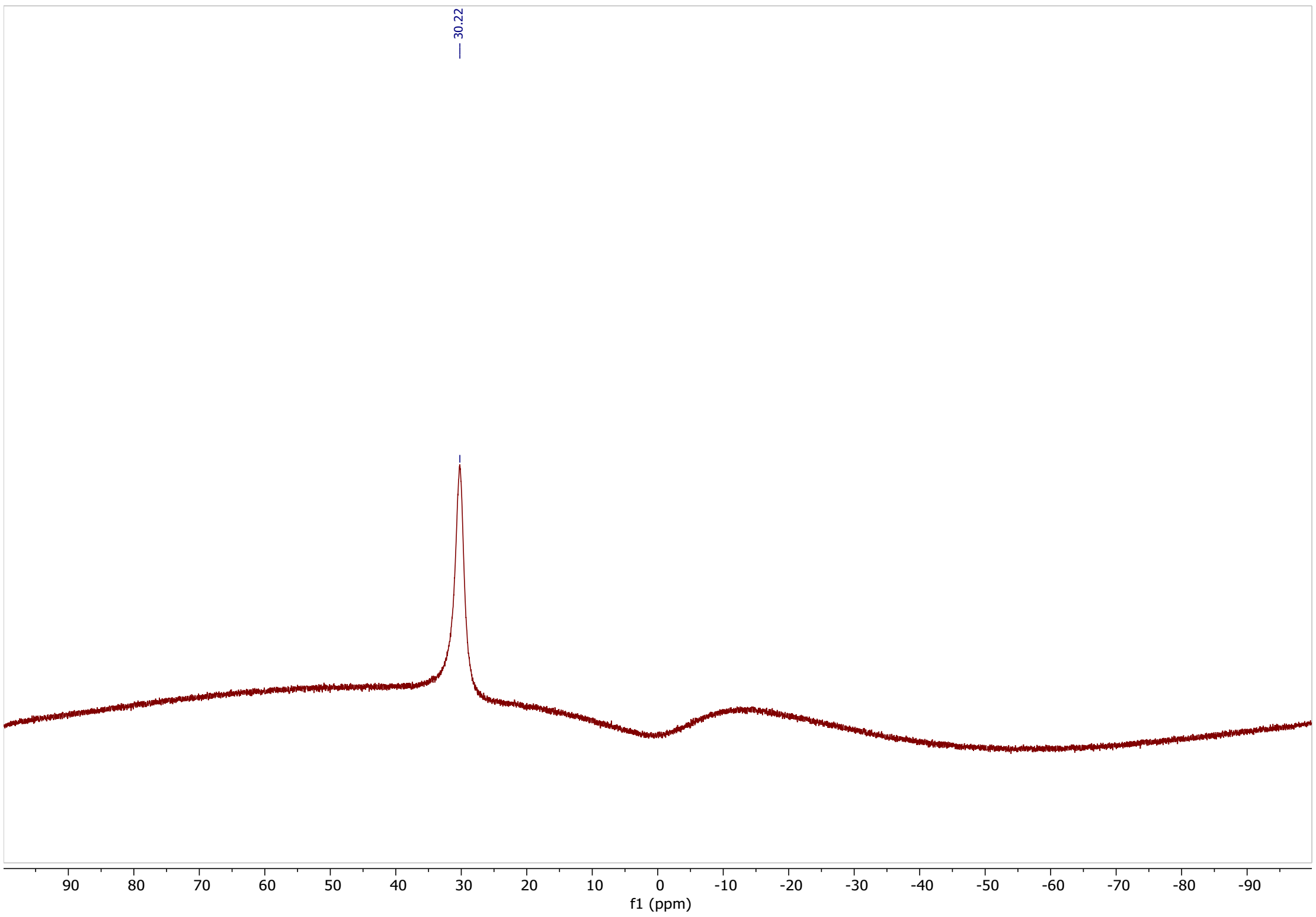

4,4,5,5-tetramethyl-2-(4-(p-tolyl)but-1-en-2-yl)-1,3,2-dioxaborolane (1e)

^{1}H NMR (500 MHz, Chloroform-*d*)

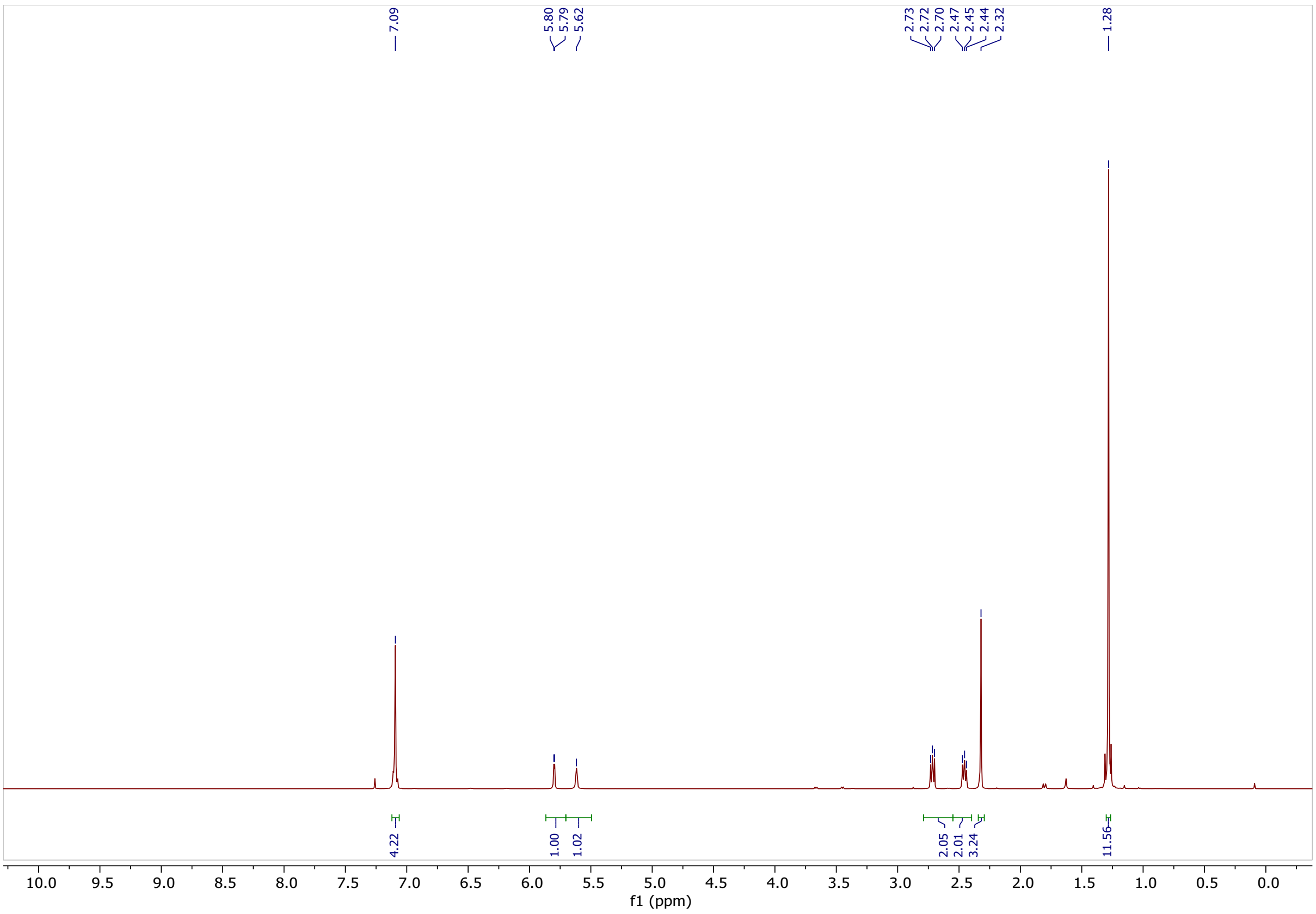

^{13}C NMR (125 MHz, Chloroform-*d*)

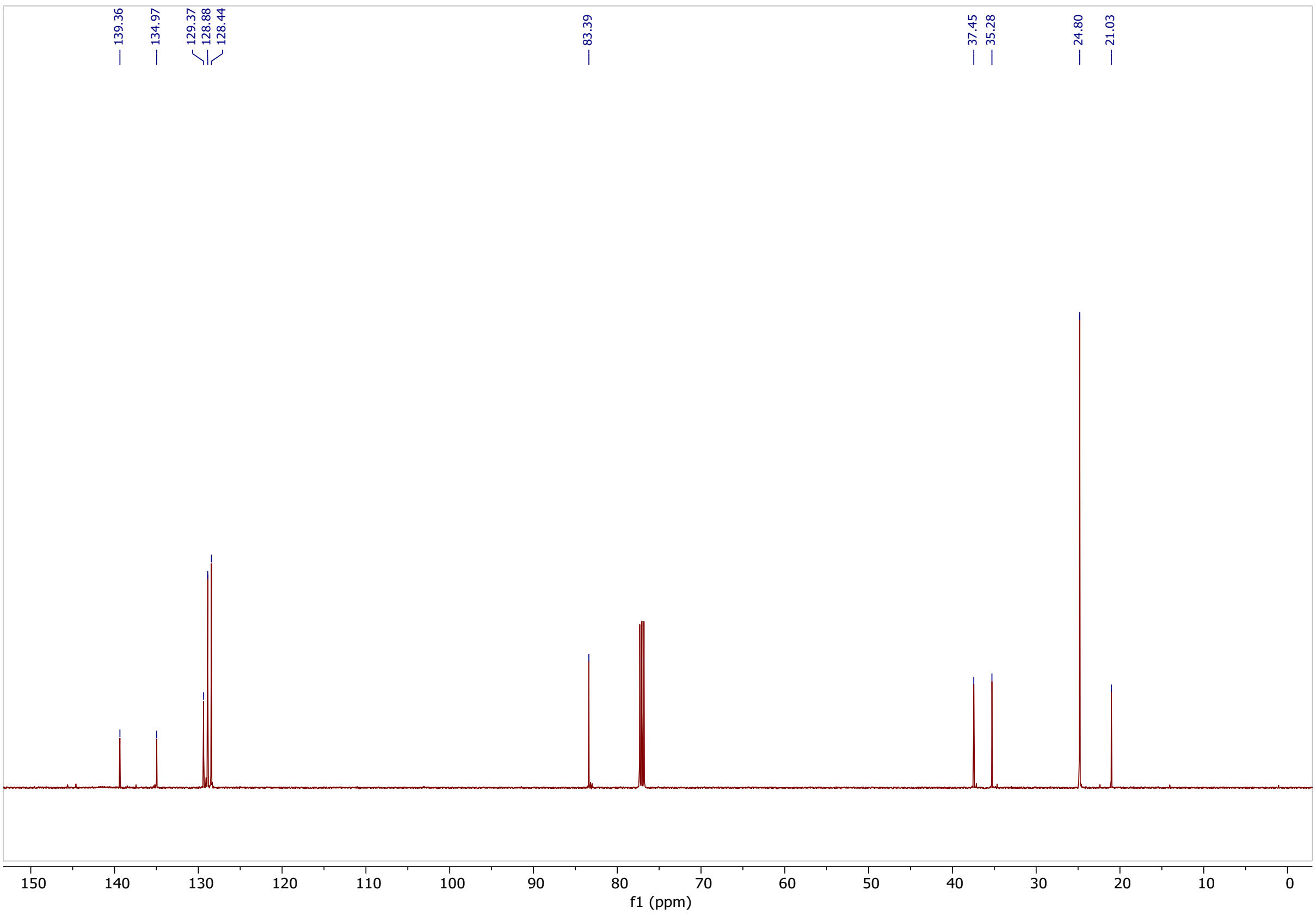

^{11}B NMR (160 MHz, Chloroform-*d*)

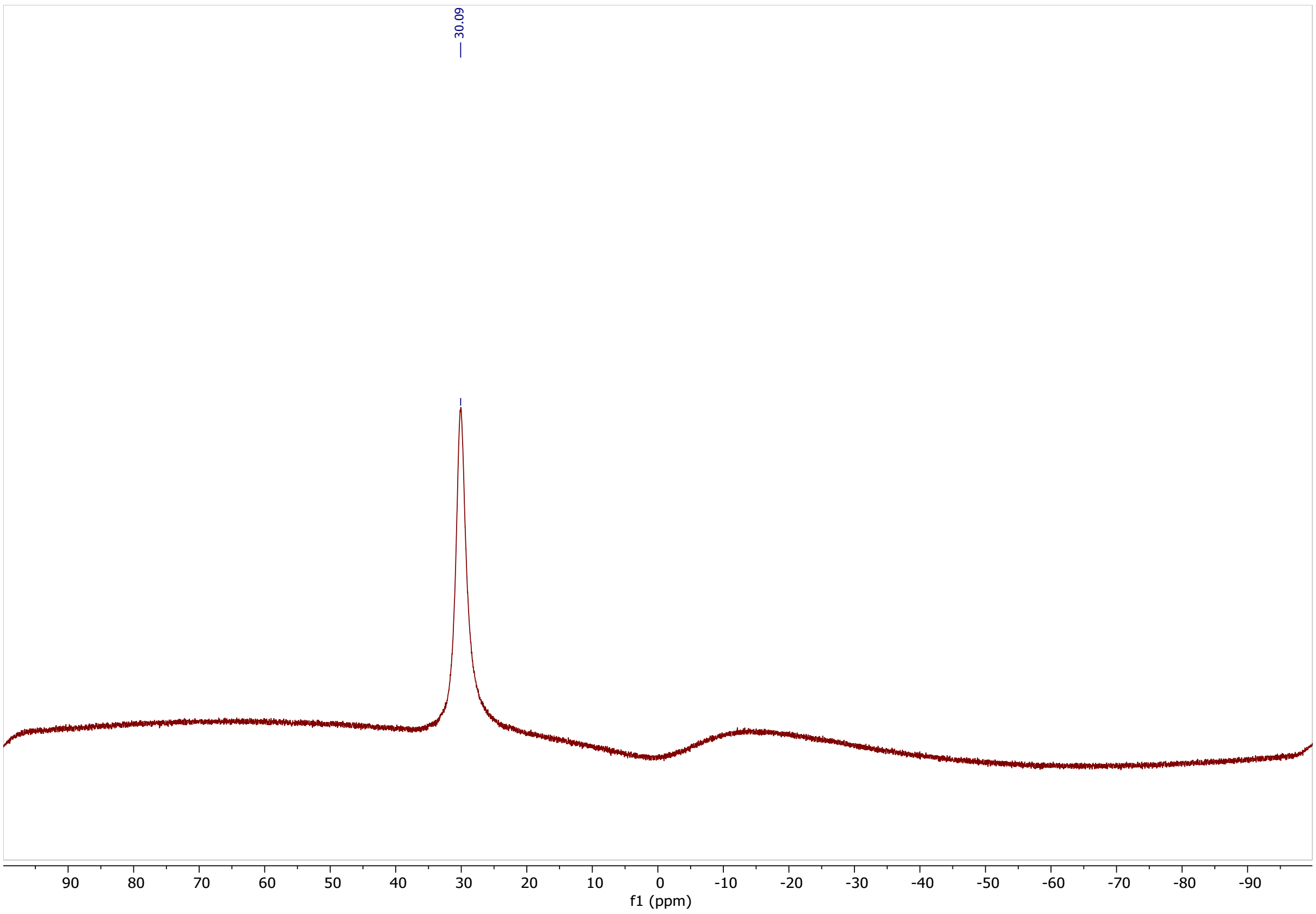

2-(4-(4-fluorophenyl)but-1-en-2-yl)-4,4,5,5-tetramethyl-1,3,2-dioxaborolane (1f)

^{1}H NMR (500 MHz, Chloroform-*d*)

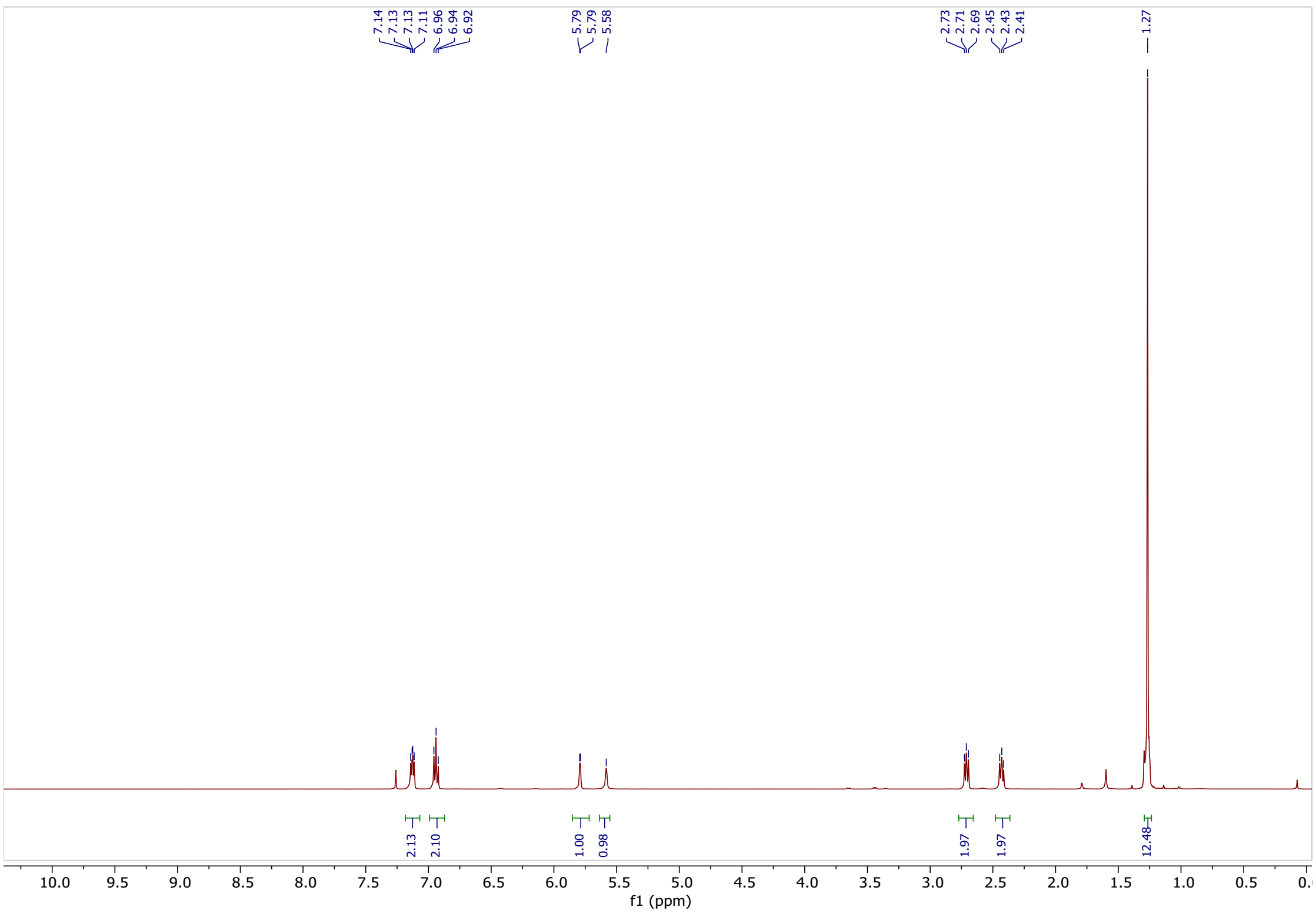

^{13}C NMR (125 MHz, Chloroform-*d*)

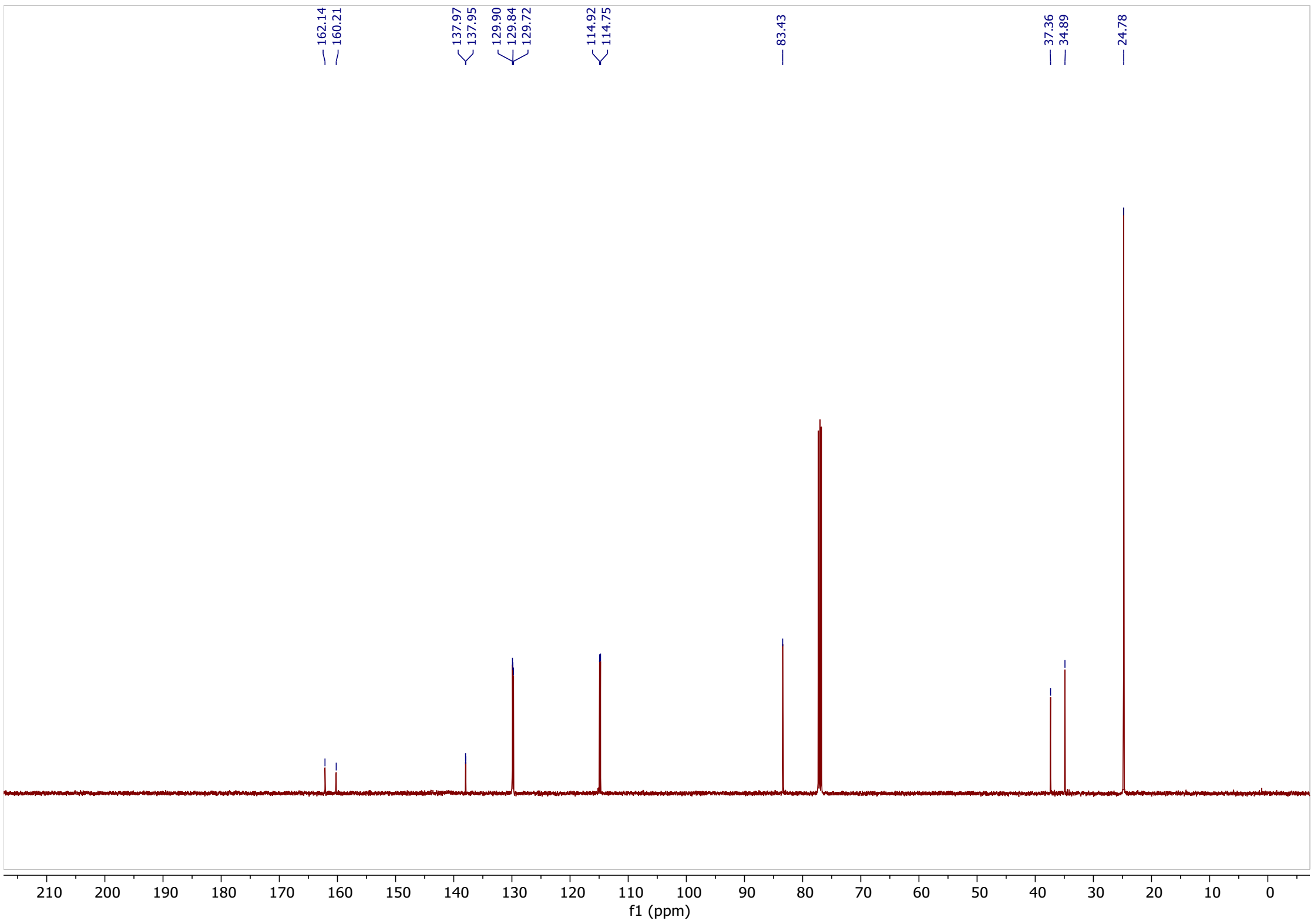

^{11}B NMR (160 MHz, Chloroform-*d*)

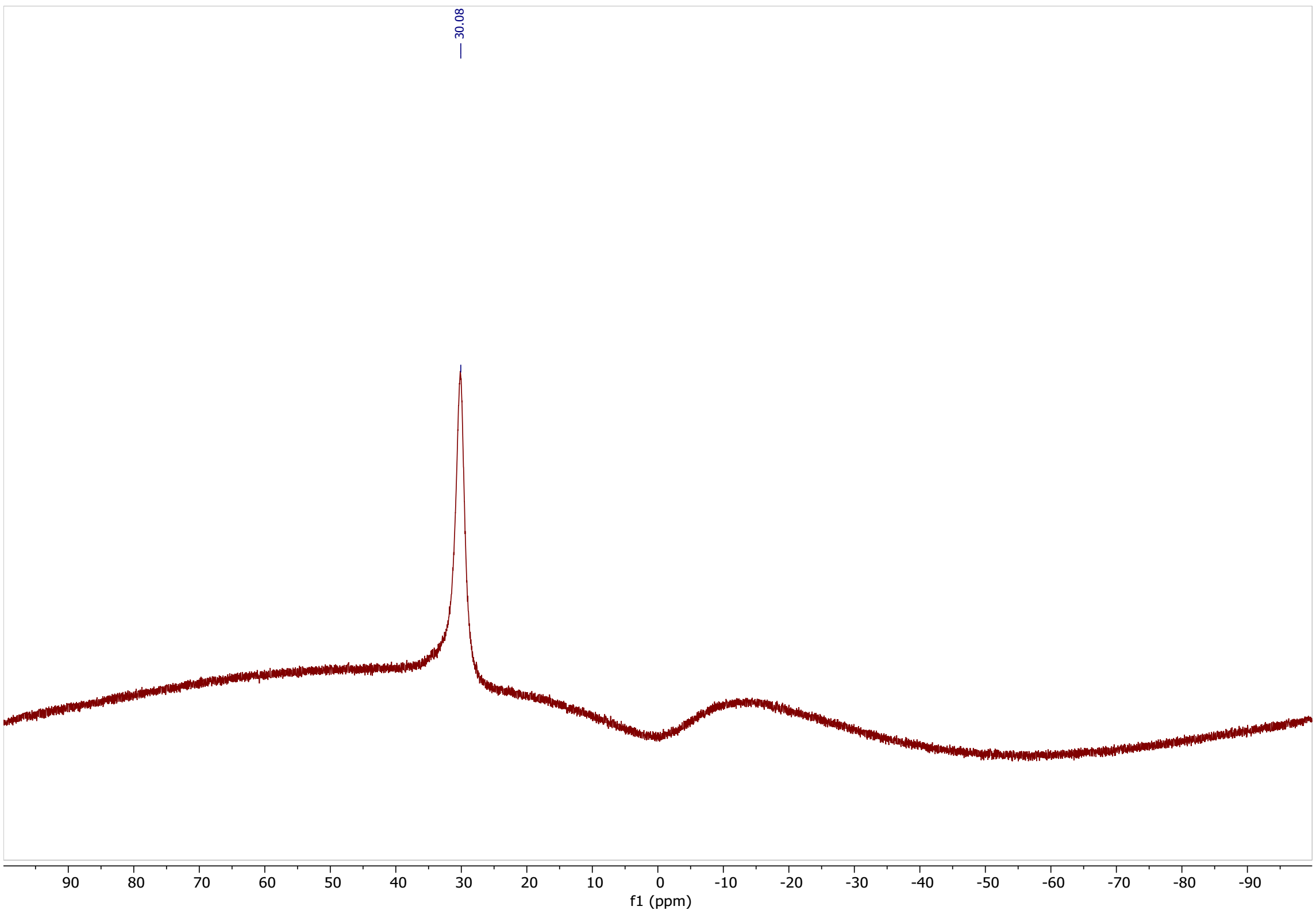

^{19}F NMR (470 MHz, Chloroform-*d*)

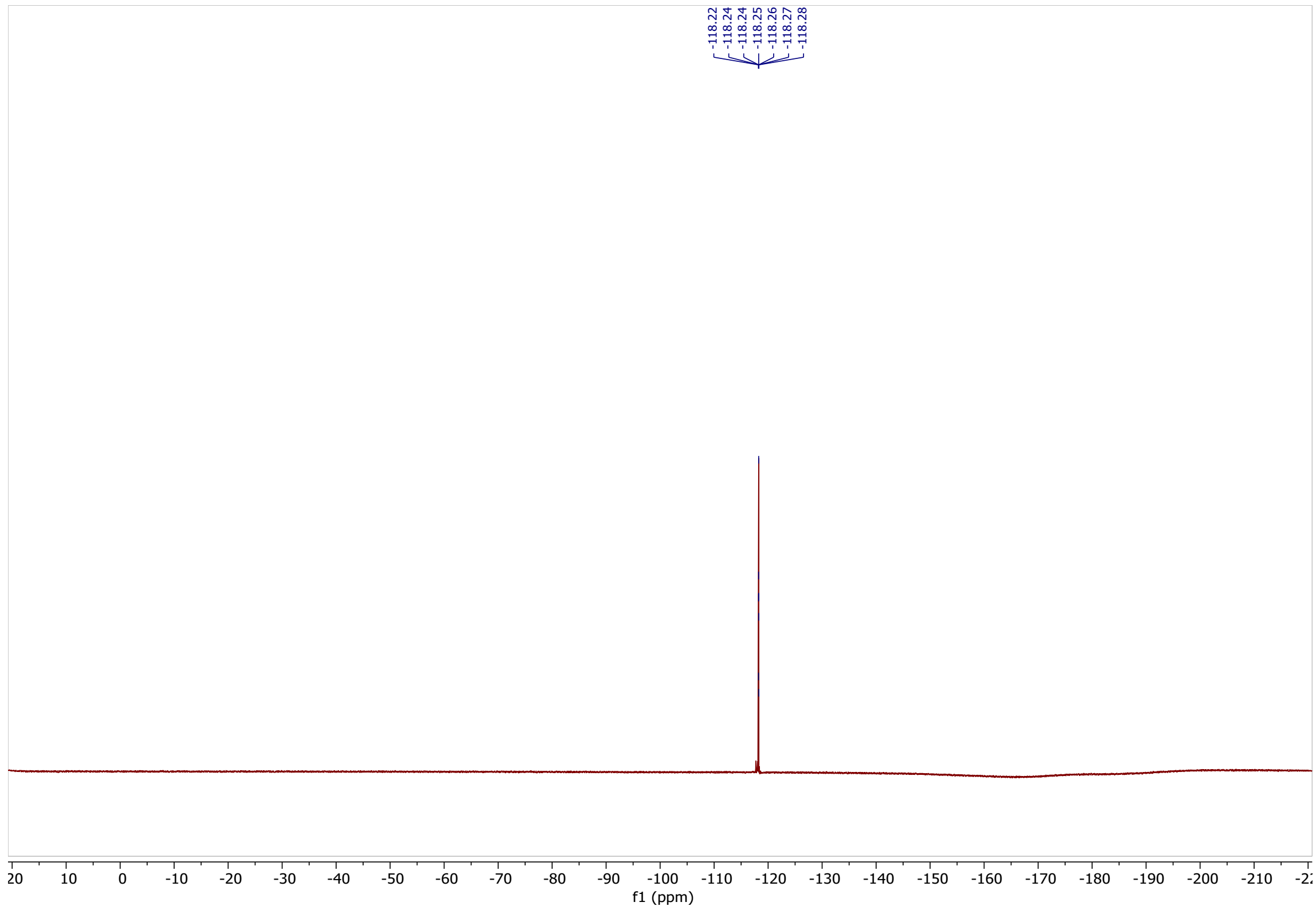

2-(4-(4-chlorophenyl)but-1-en-2-yl)-4,4,5,5-tetramethyl-1,3,2-dioxaborolane (1g)

^{1}H NMR (500 MHz, Chloroform-*d*)

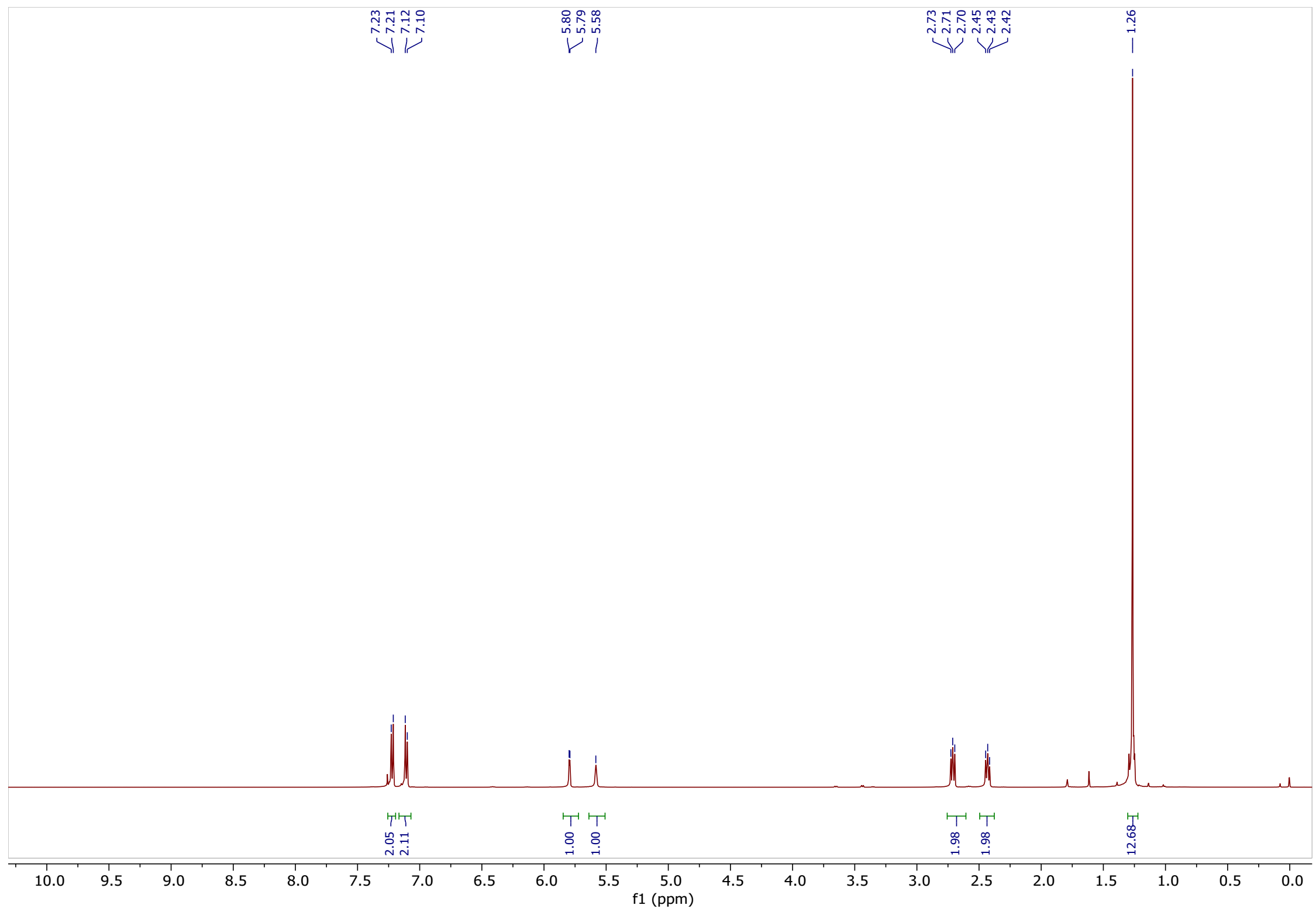

^{13}C NMR (125 MHz, Chloroform-*d*)

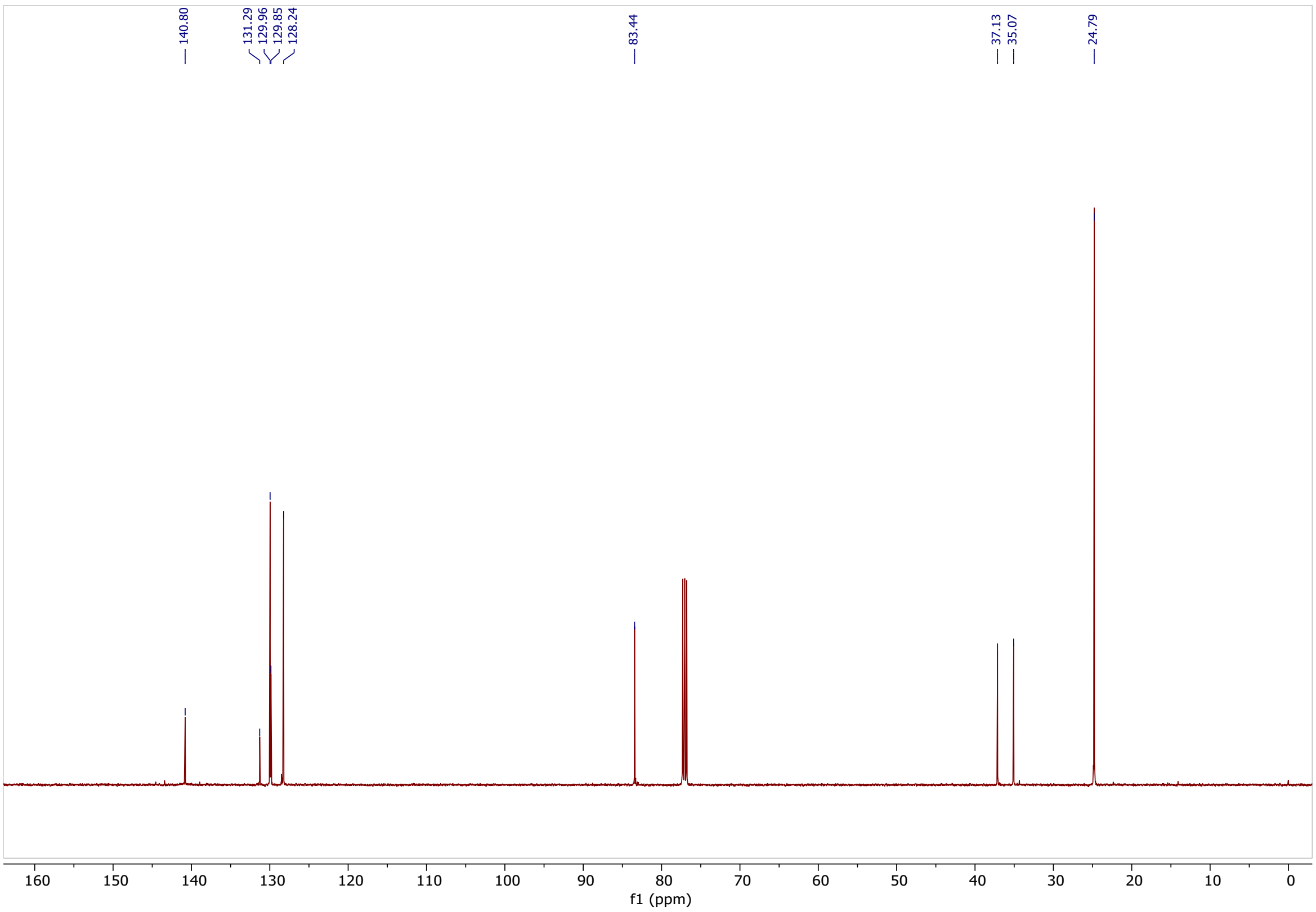

^{11}B NMR (160 MHz, Chloroform-*d*)

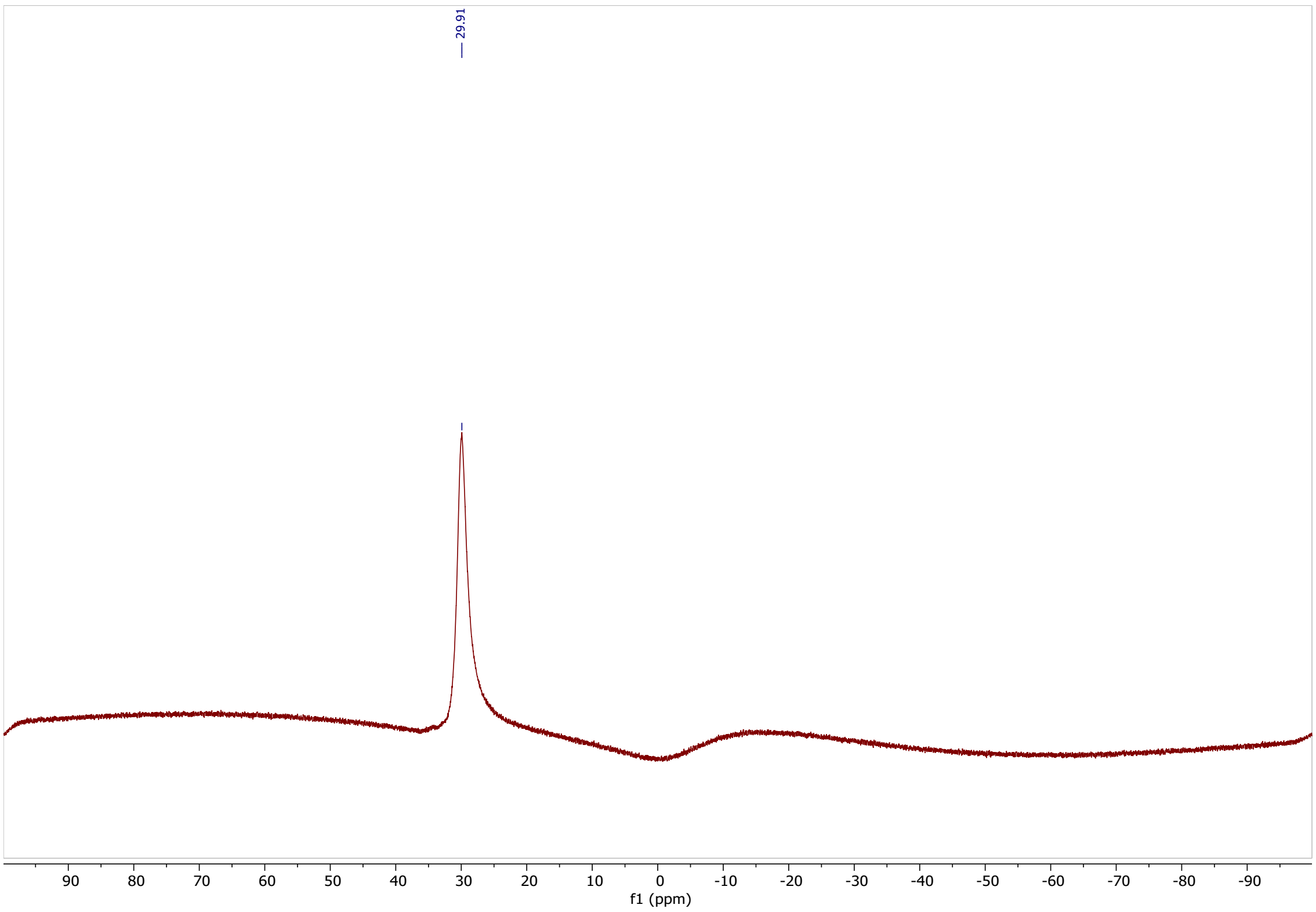

2-(4-(4-bromophenyl)but-1-en-2-yl)-4,4,5,5-tetramethyl-1,3,2-dioxaborolane (1h)

^{1}H NMR (500 MHz, Chloroform-*d*)

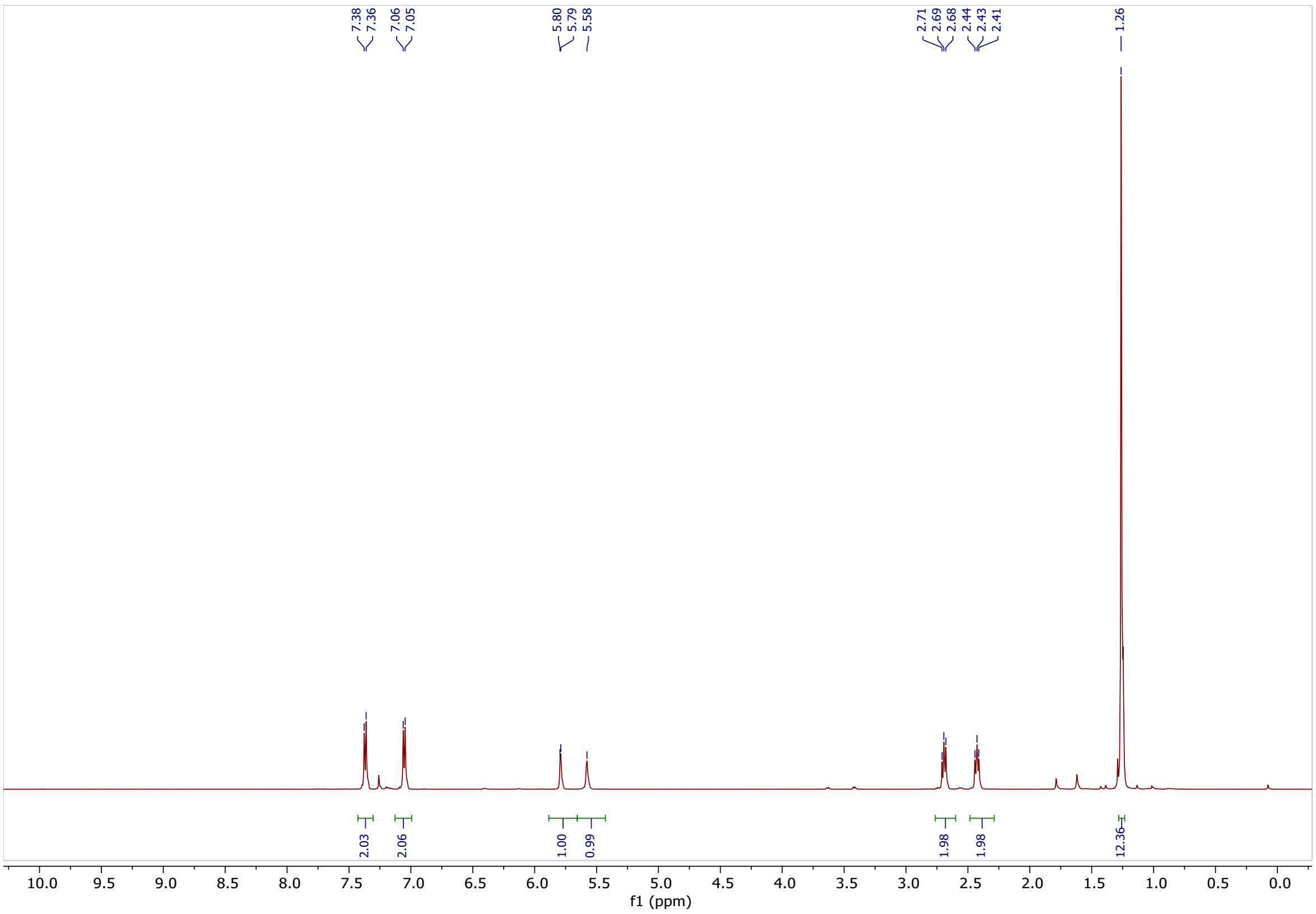

^{13}C NMR (125 MHz, Chloroform-*d*)

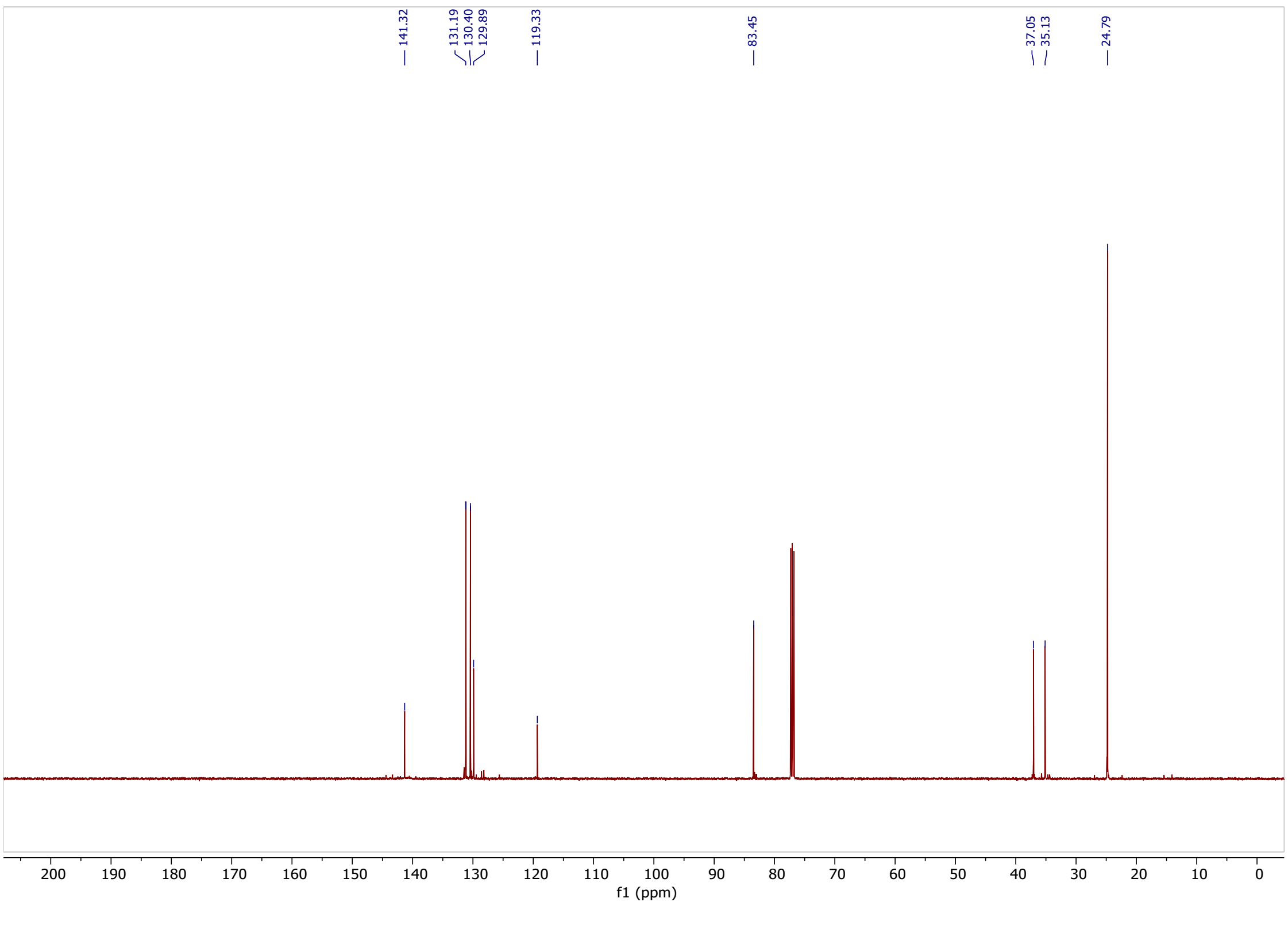

^{11}B NMR (160 MHz, Chloroform-*d*)

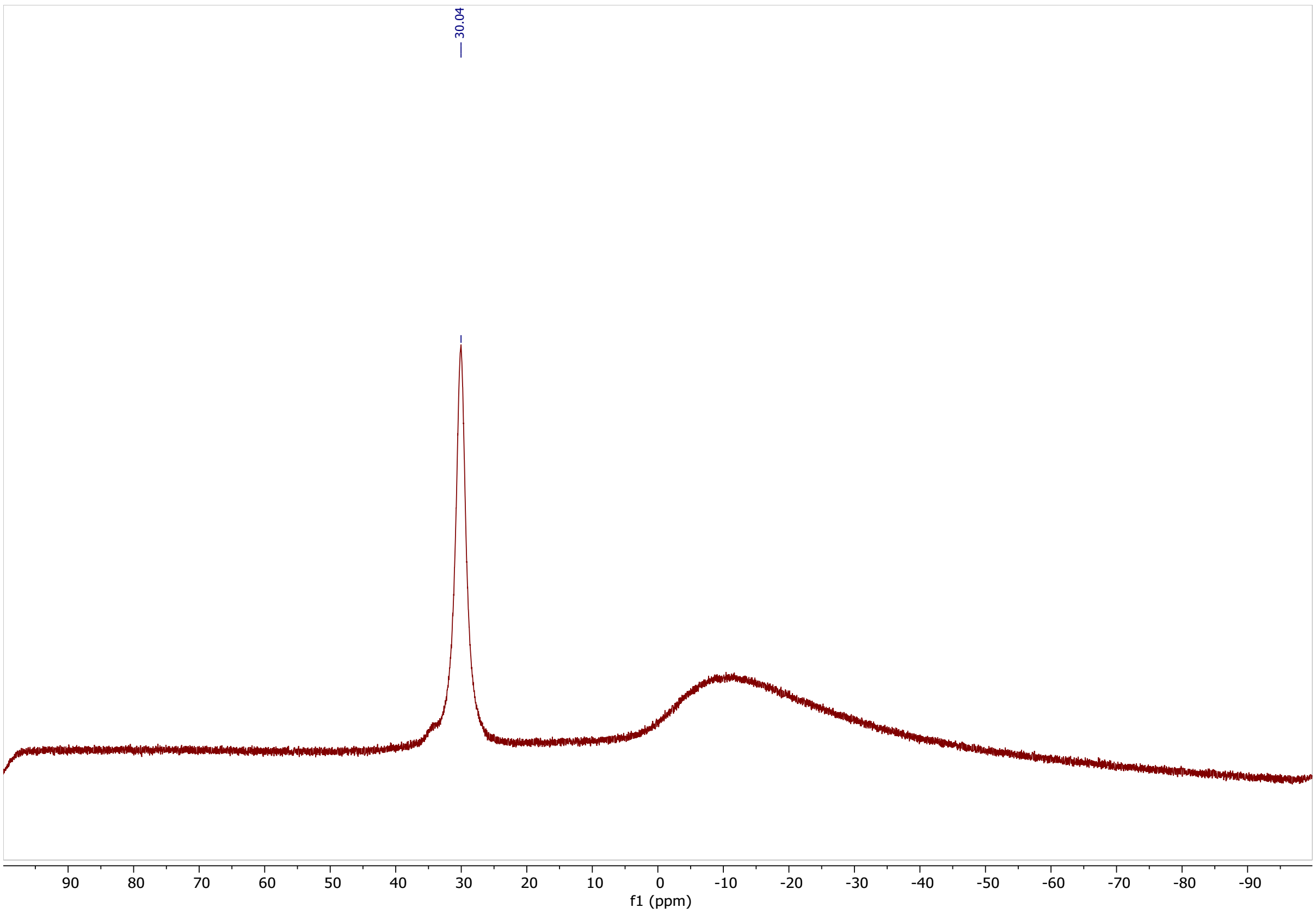

4,4,5,5-tetramethyl-2-(4-(4-(trifluoromethoxy)phenyl)but-1-en-2-yl)-1,3,2-dioxaborolane 1i)

^{1}H NMR (500 MHz, Chloroform-*d*)

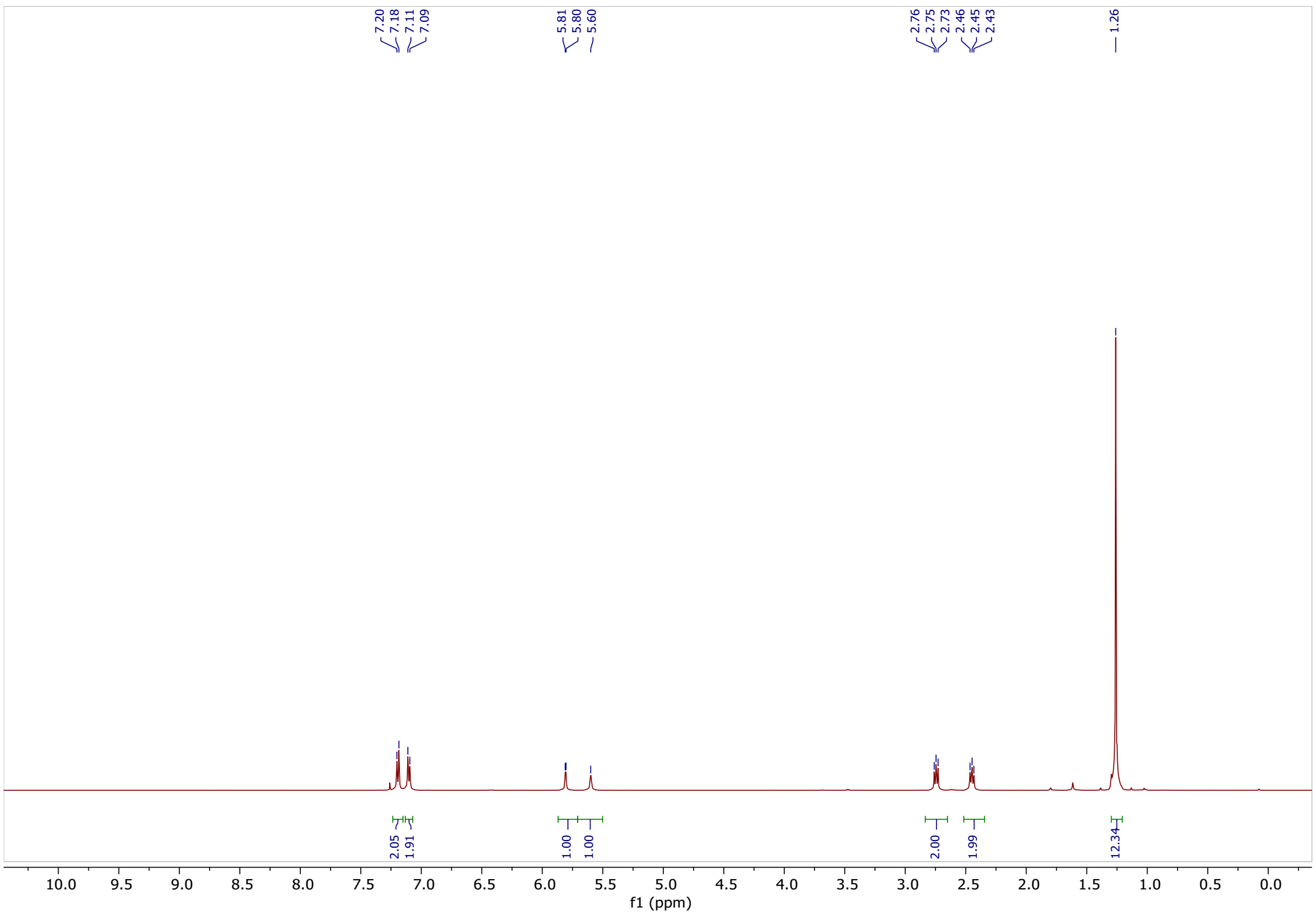

^{13}C NMR (125 MHz, Chloroform-*d*)

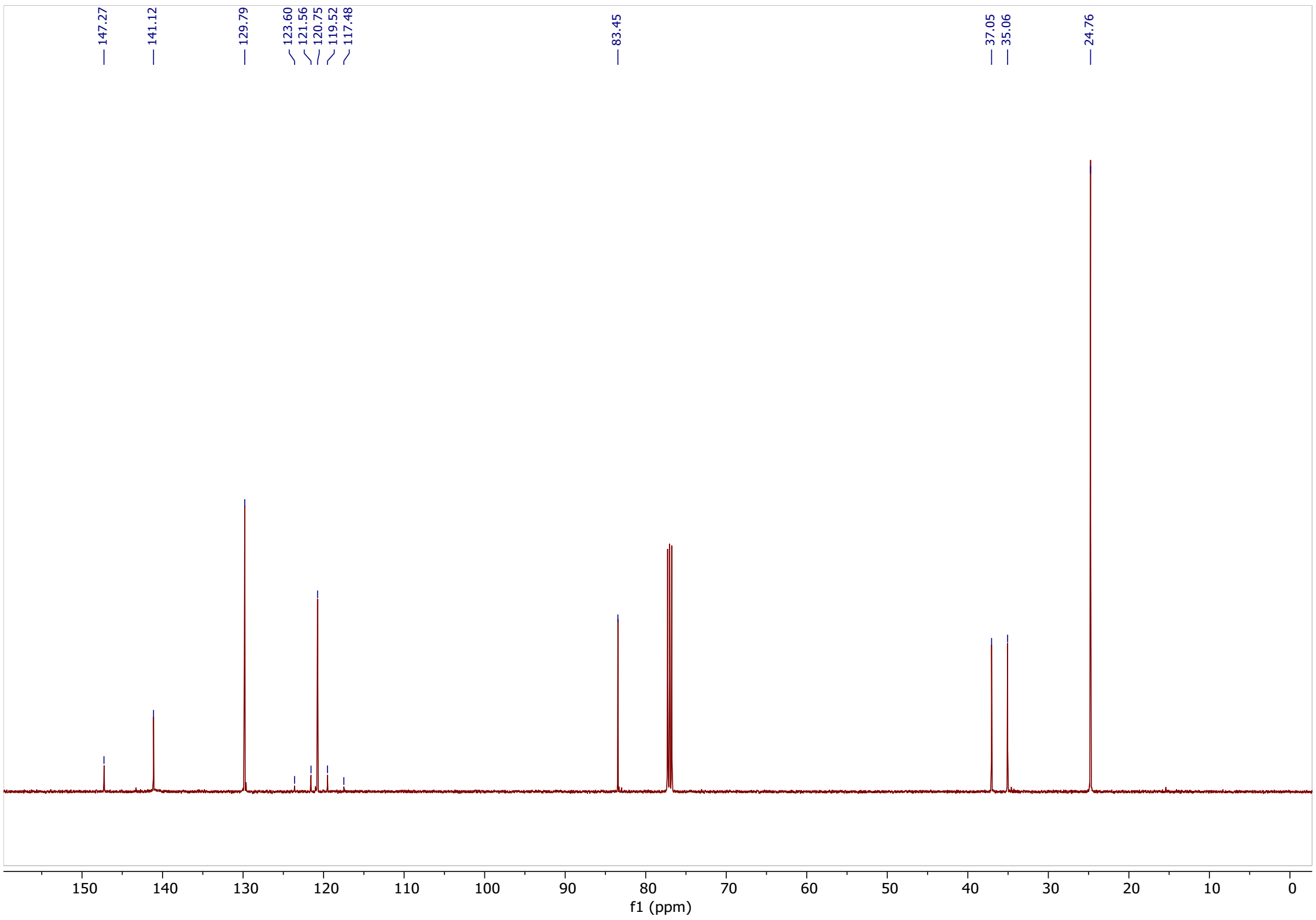

^{11}B NMR (160 MHz, Chloroform-*d*)

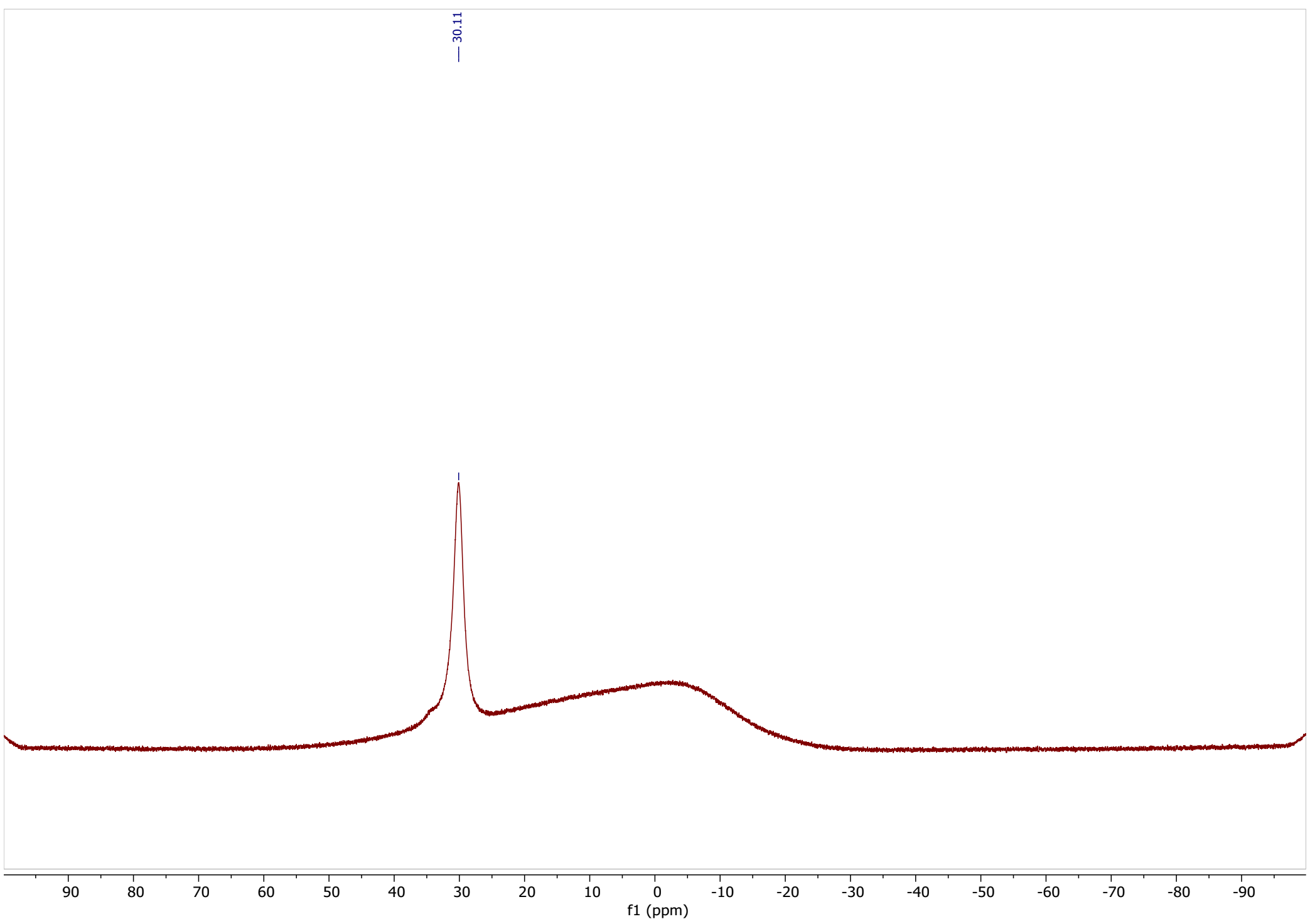

^{19}F NMR (470 MHz, Chloroform-*d*)

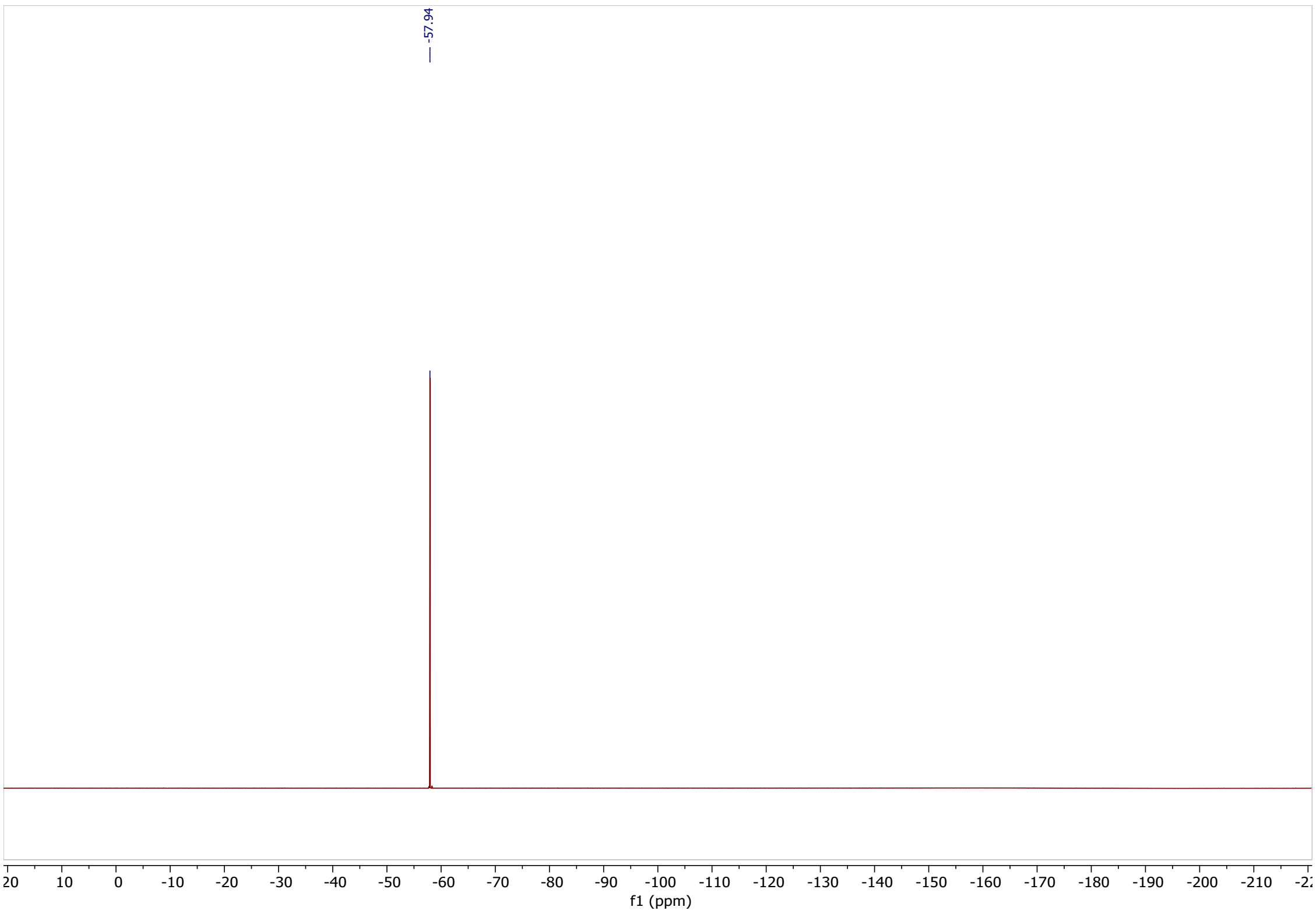

4,4,5,5-tetramethyl-2-(4-(3-(trifluoromethyl)phenyl)but-1-en-2-yl)-1,3,2-dioxaborolane (1j)

^{1}H NMR (500 MHz, Chloroform-*d*)

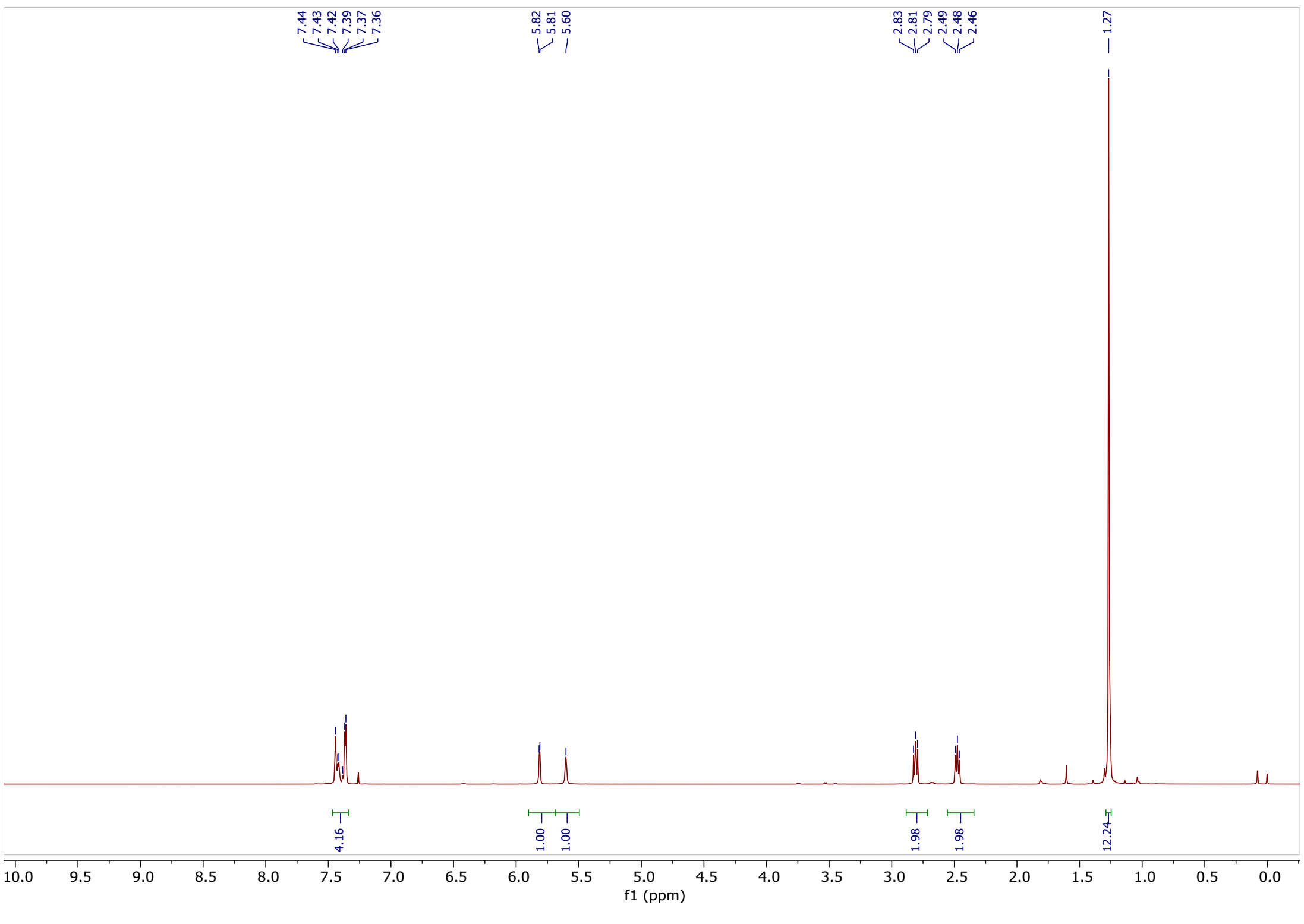

^{13}C NMR (125 MHz, Chloroform-*d*)

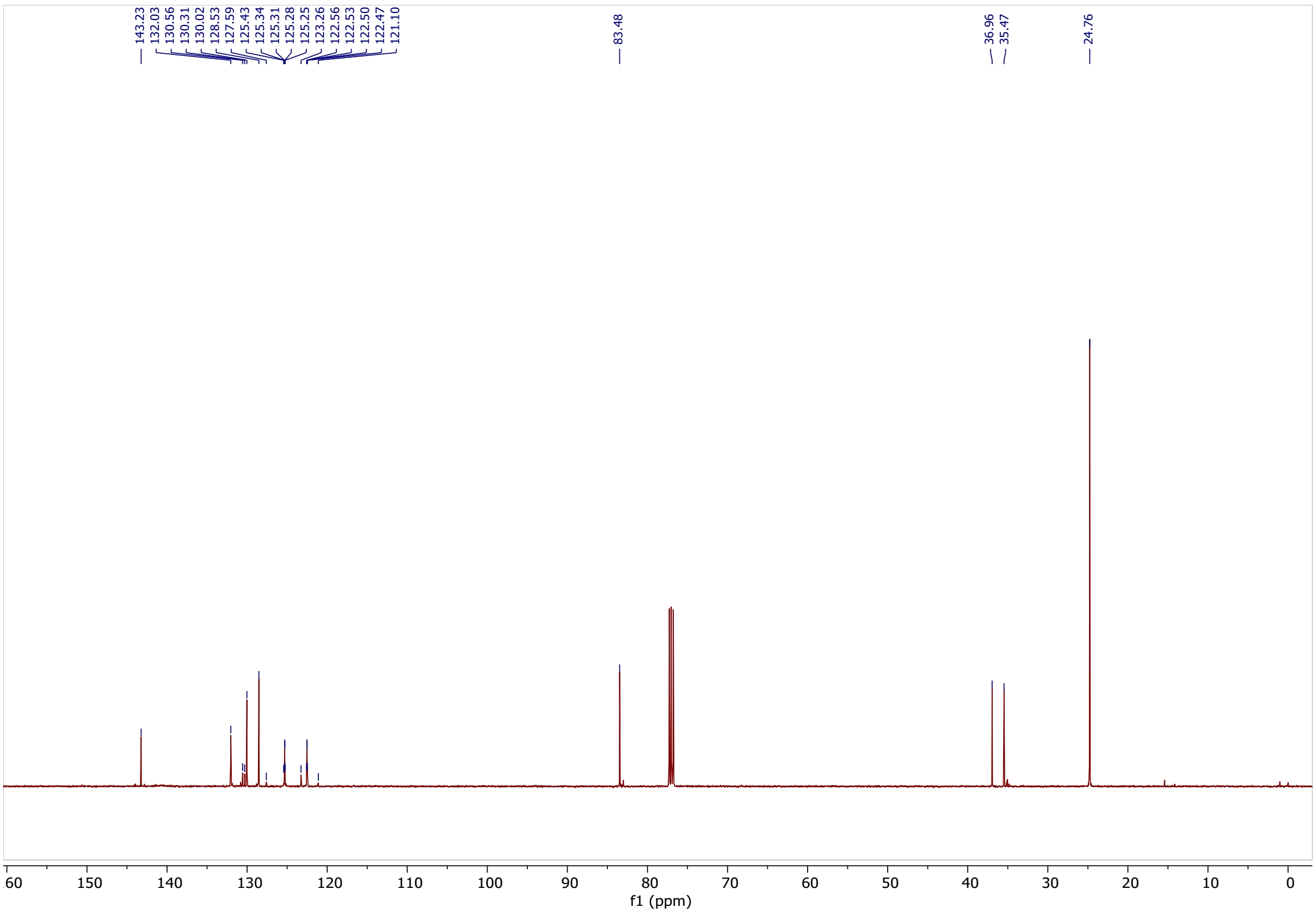

^{11}B NMR (160 MHz, Chloroform-*d*)

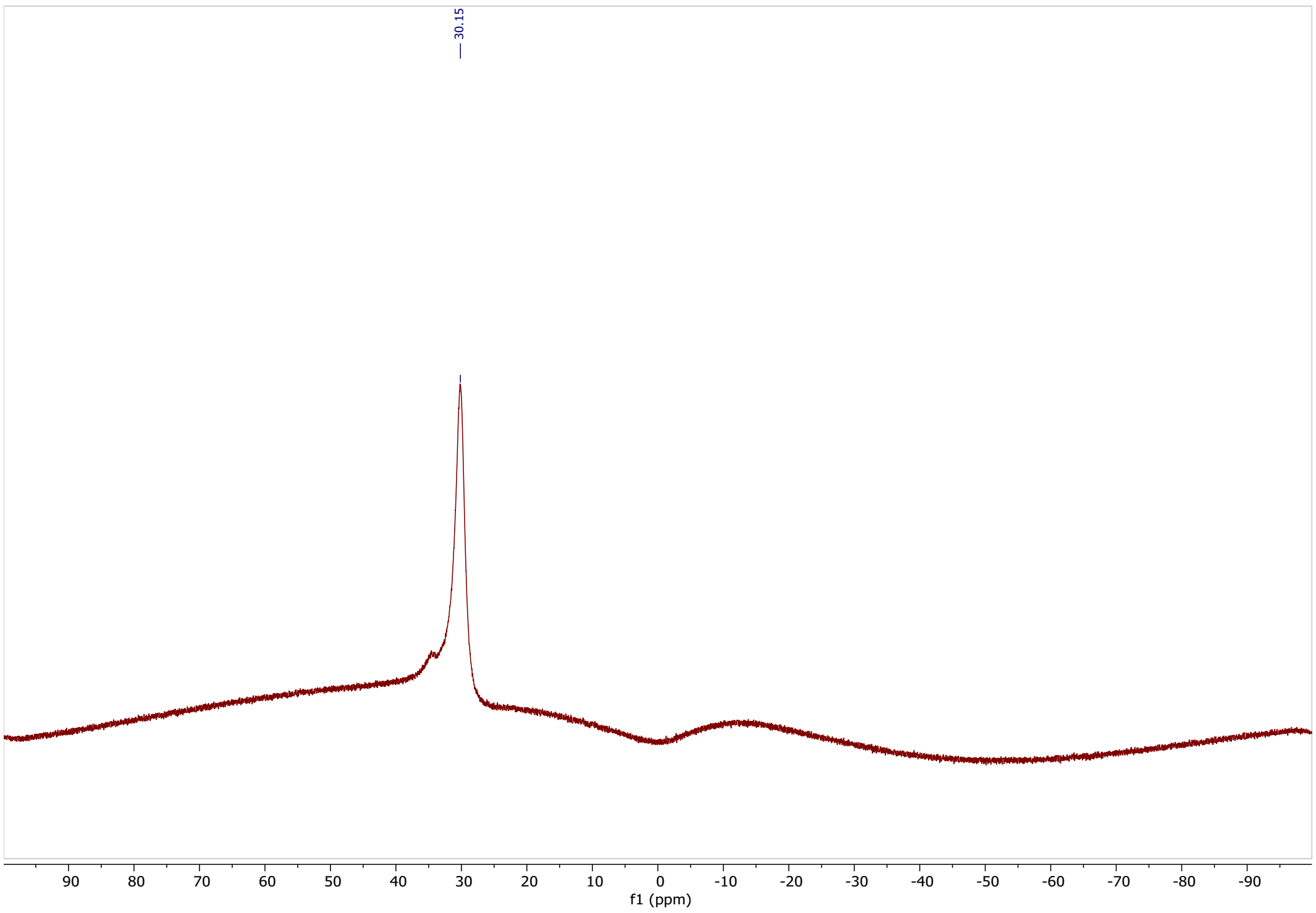

^{19}F NMR (470 MHz, Chloroform-*d*)

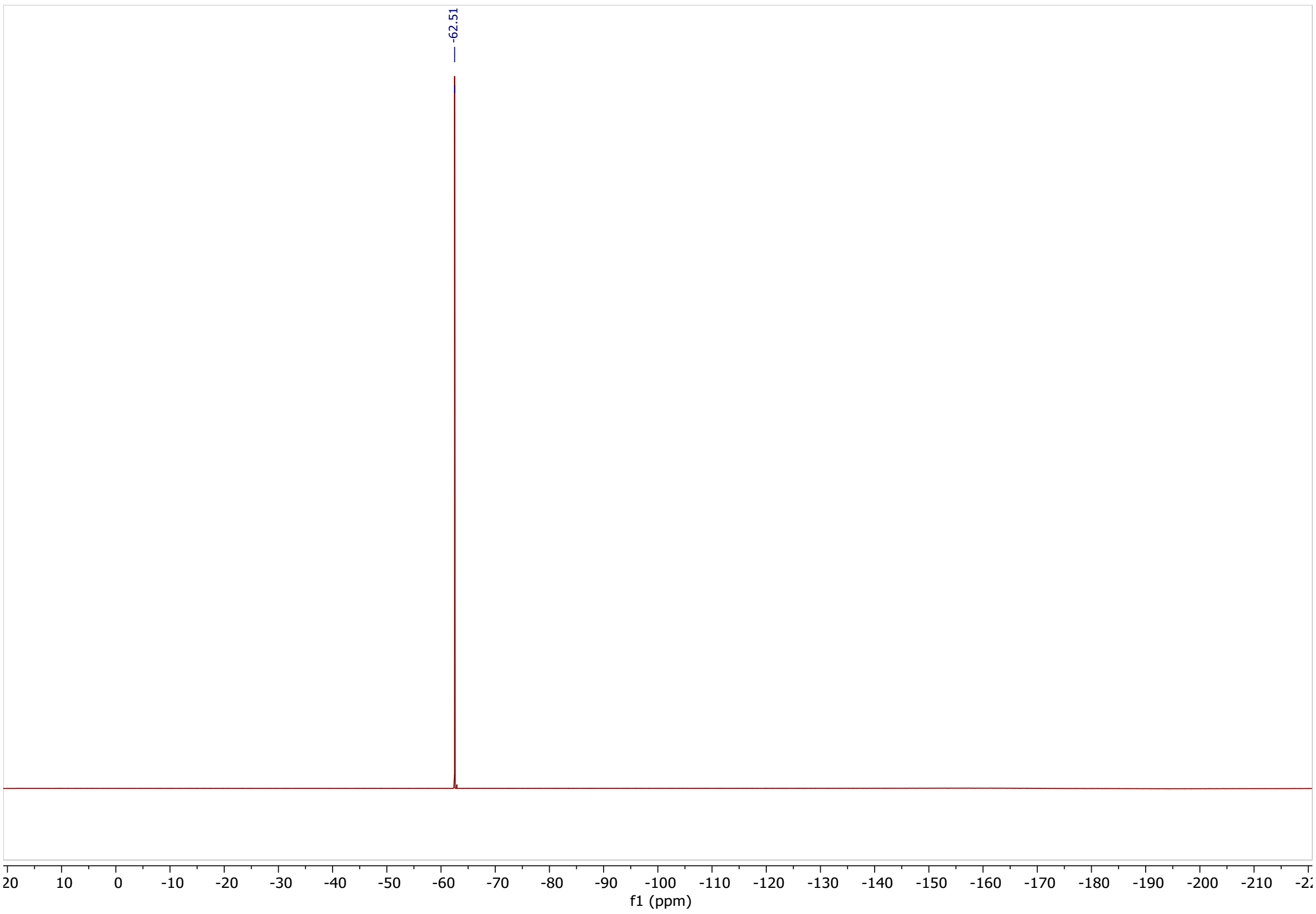

2-(4-(2,3-dimethylphenyl)but-1-en-2-yl)-4,4,5,5-tetramethyl-1,3,2-dioxaborolane (1k)

^{1}H NMR (500 MHz, Chloroform-*d*)

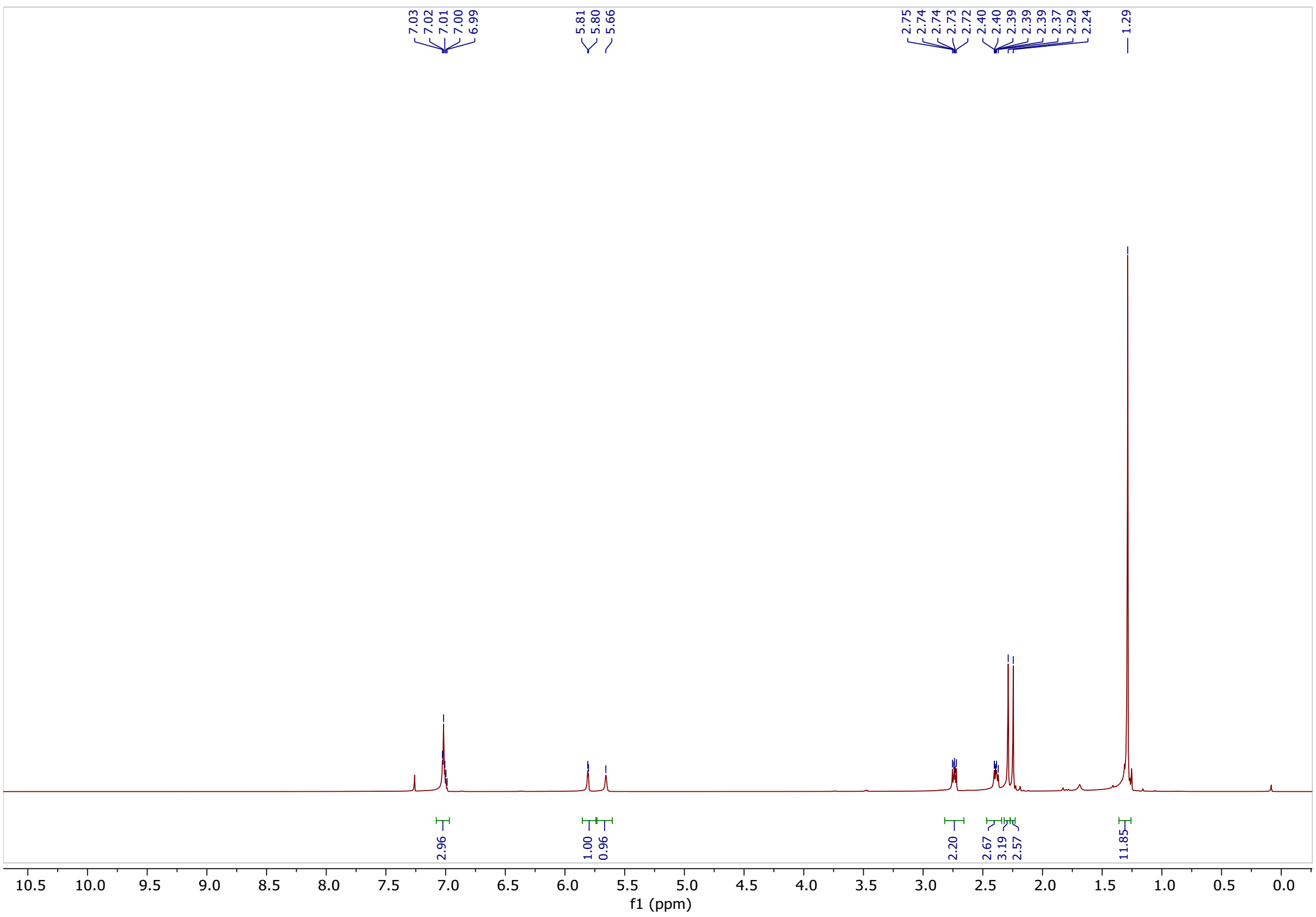

^{13}C NMR (125 MHz, Chloroform-*d*)

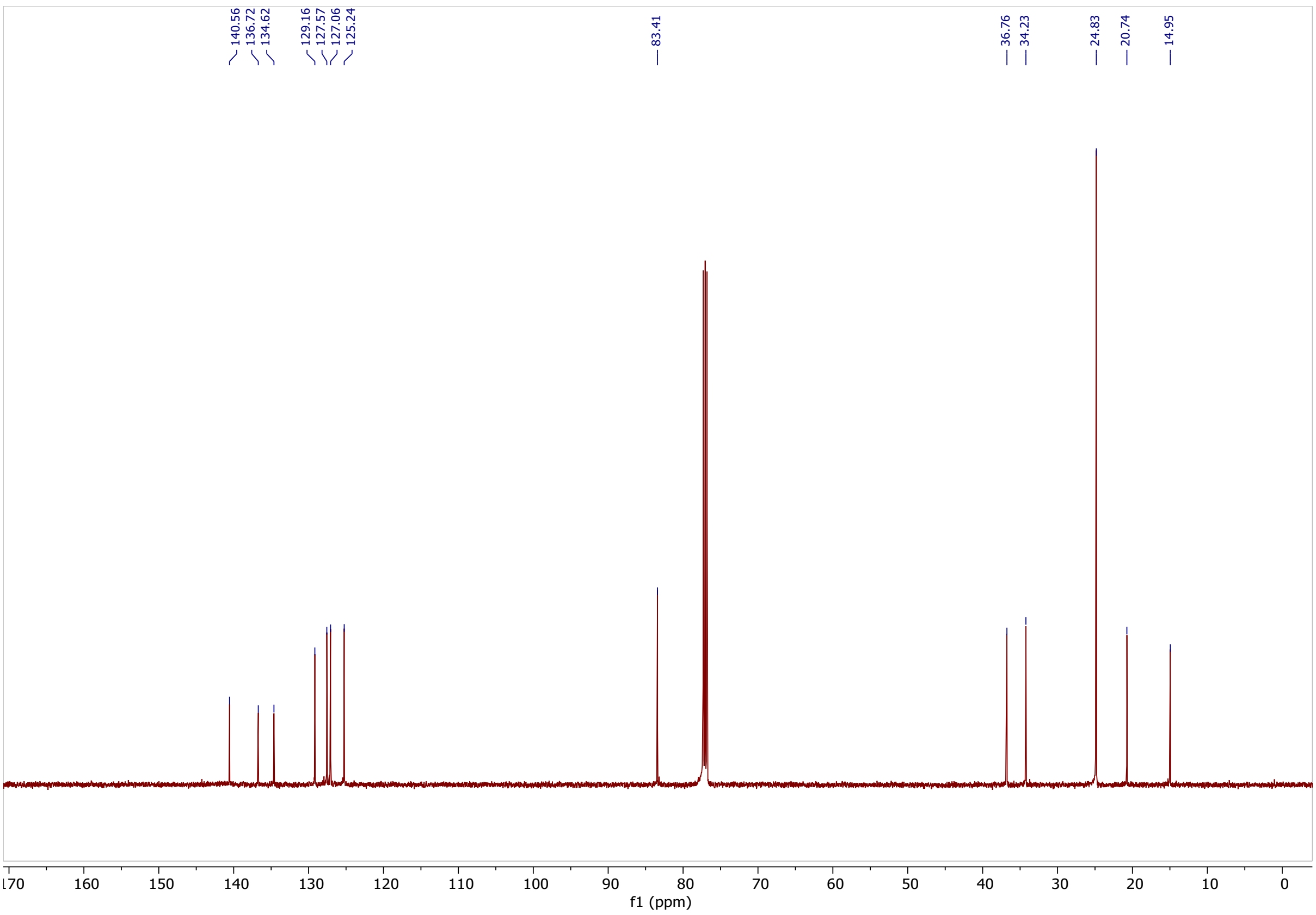

^{11}B NMR (160 MHz, Chloroform-*d*)

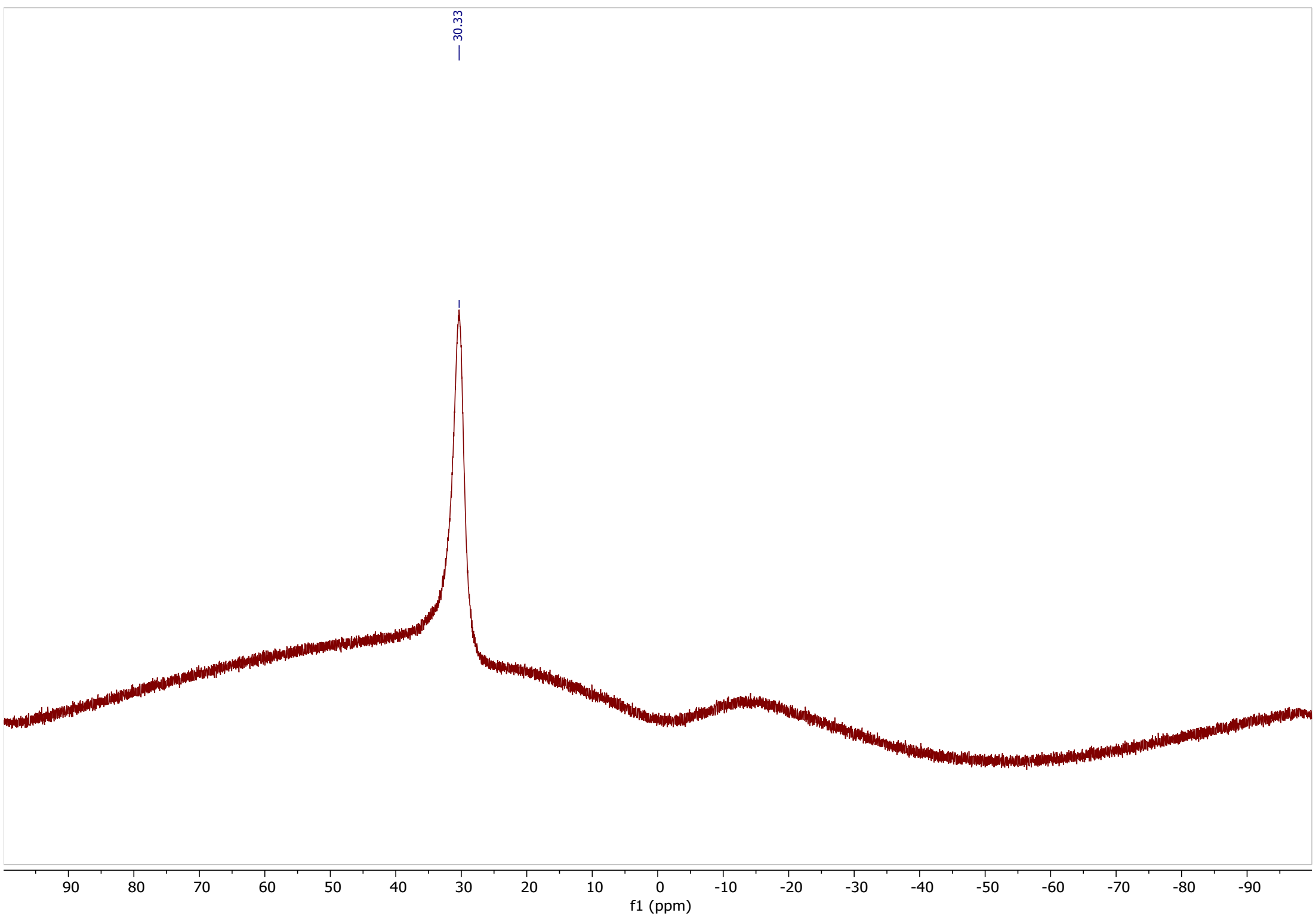

2-(4-(3,5-dimethylphenyl)but-1-en-2-yl)-4,4,5,5-tetramethyl-1,3,2-dioxaborolane (1l)

^{1}H NMR (500 MHz, Chloroform-*d*)

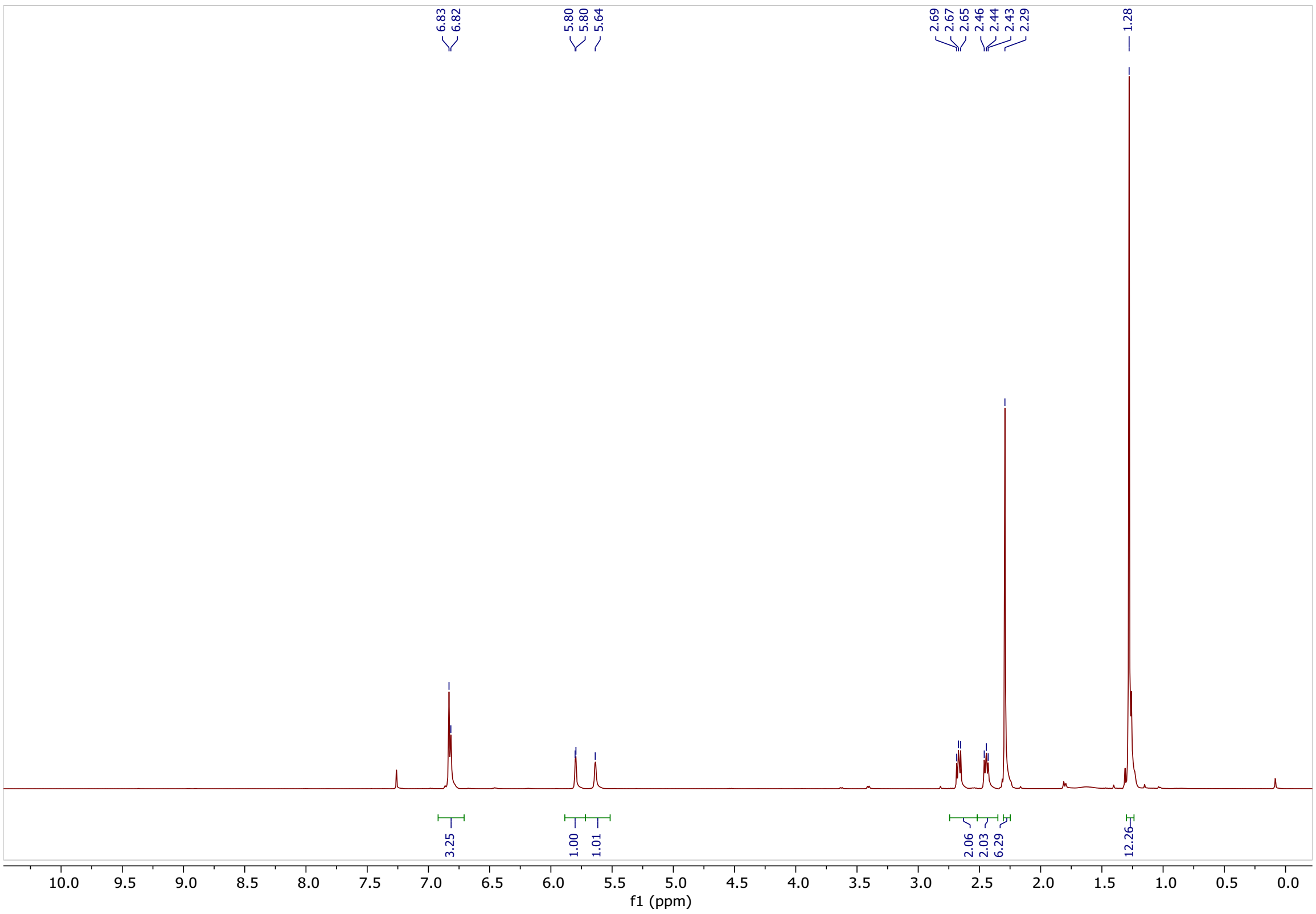

^{13}C NMR (125 MHz, Chloroform-*d*)

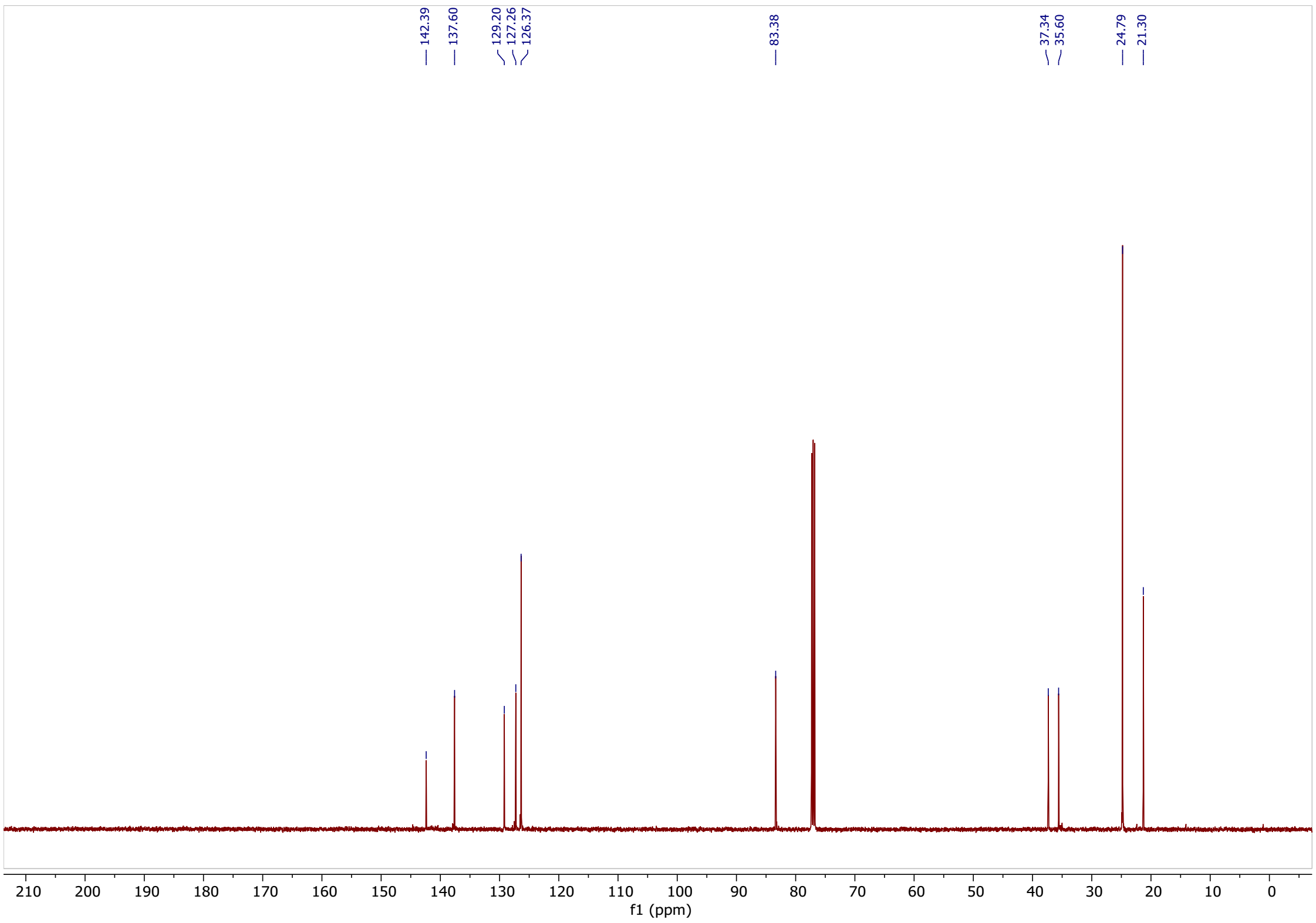

^{11}B NMR (160 MHz, Chloroform-*d*)

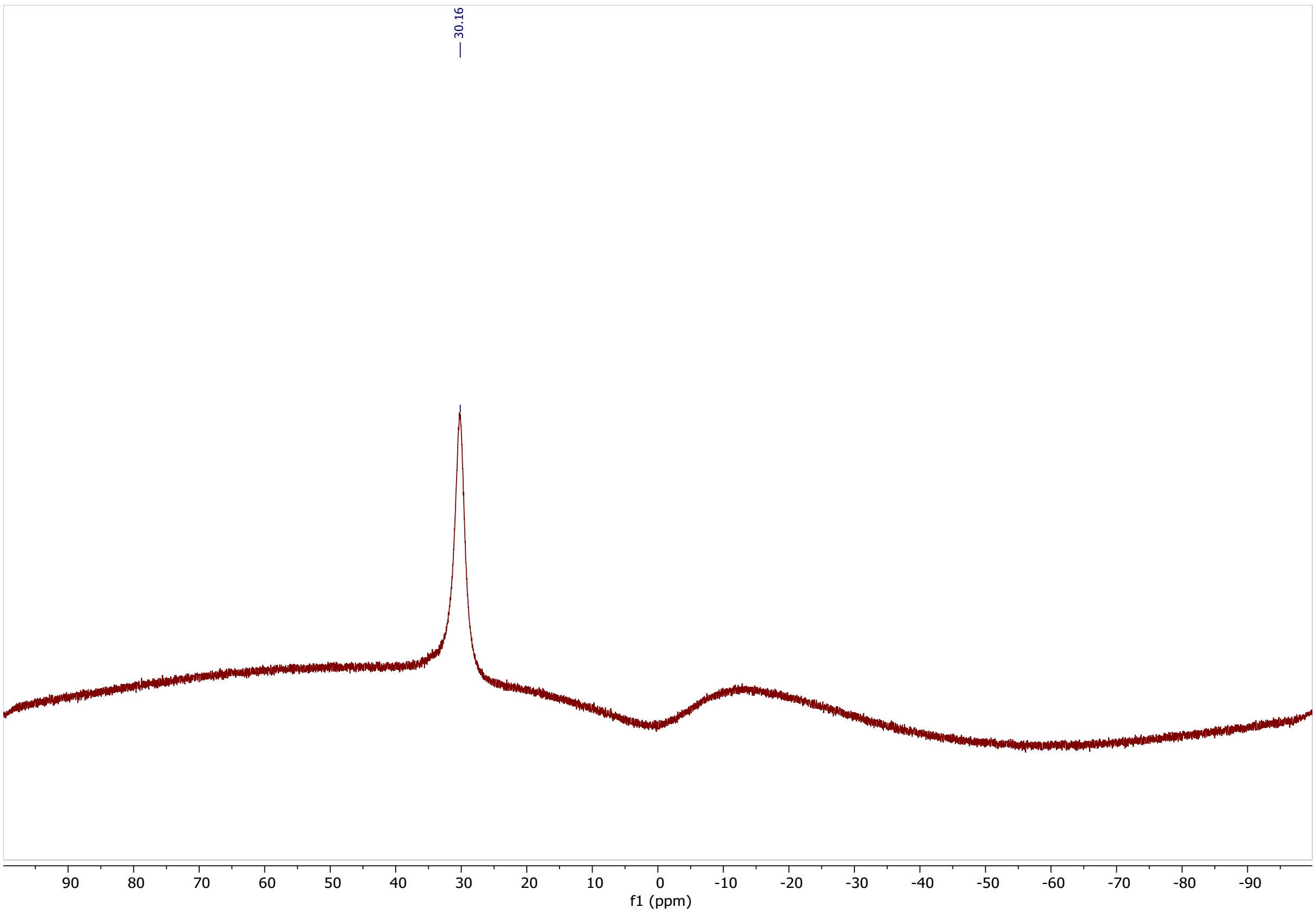

2-(4-(3-methoxyphenyl)but-1-en-2-yl)-4,4,5,5-tetramethyl-1,3,2-dioxaborolane (1m)

^{1}H NMR (500 MHz, Chloroform-*d*)

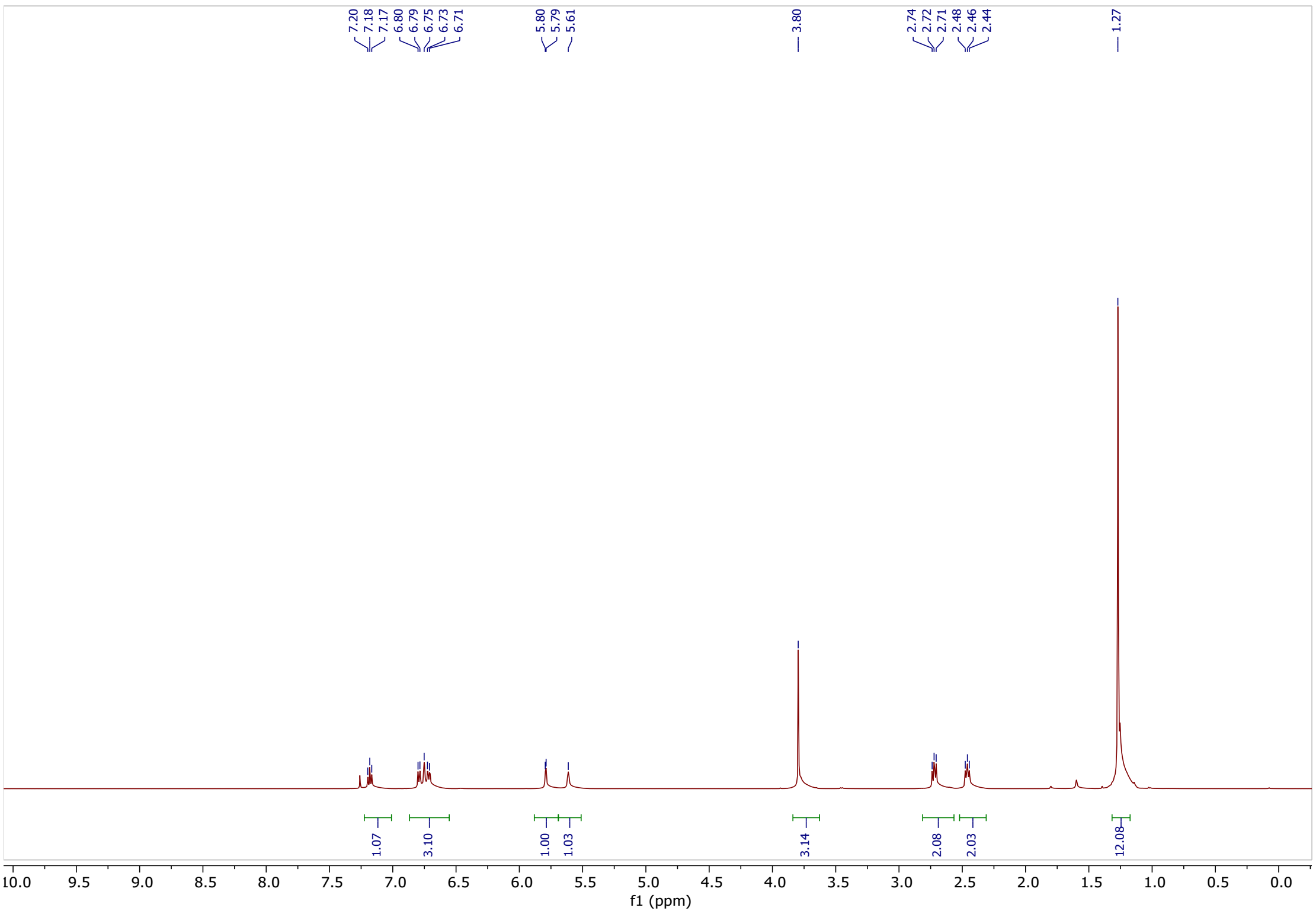

^{13}C NMR (125 MHz, Chloroform-*d*)

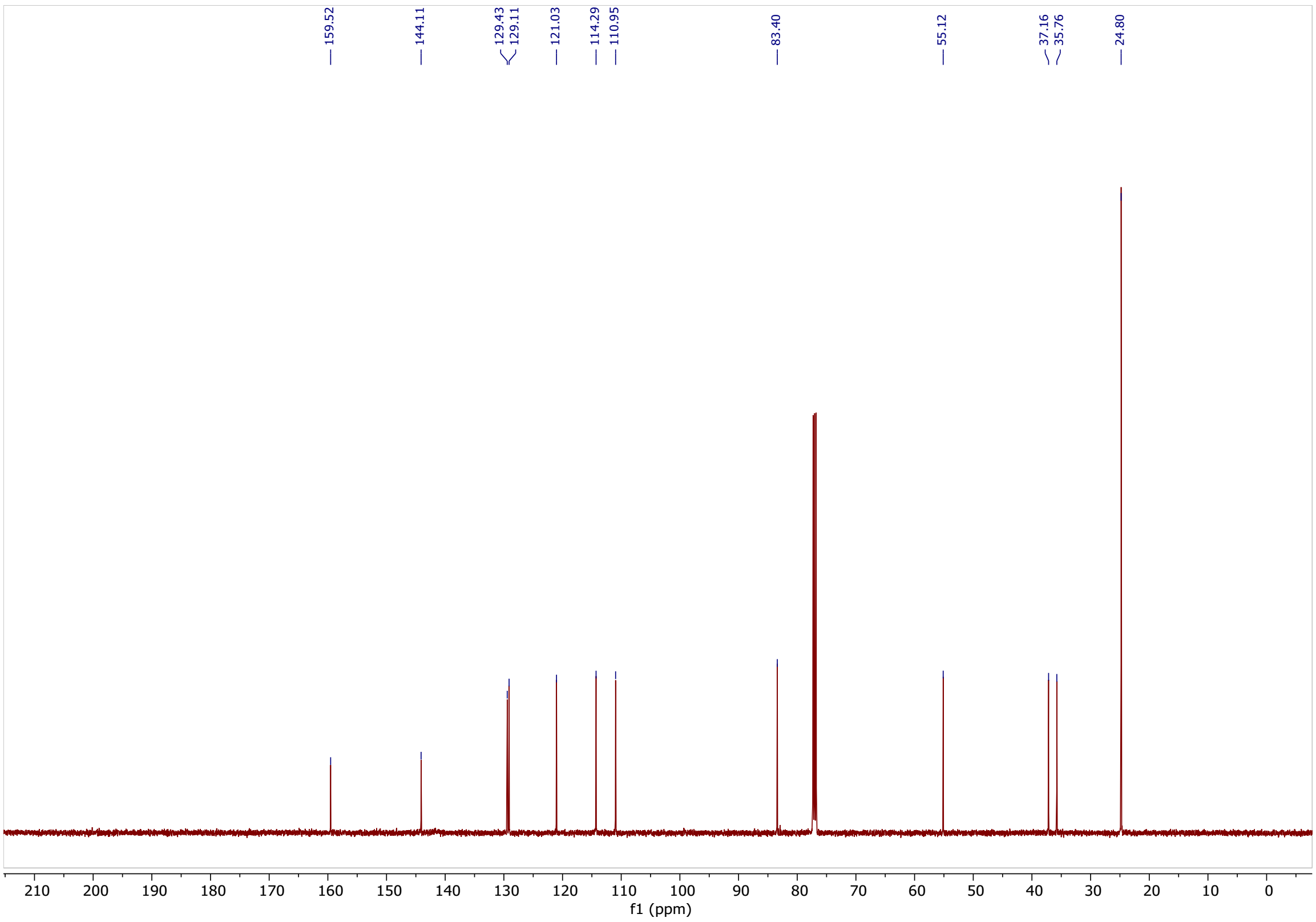

^{11}B NMR (160 MHz, Chloroform-*d*)

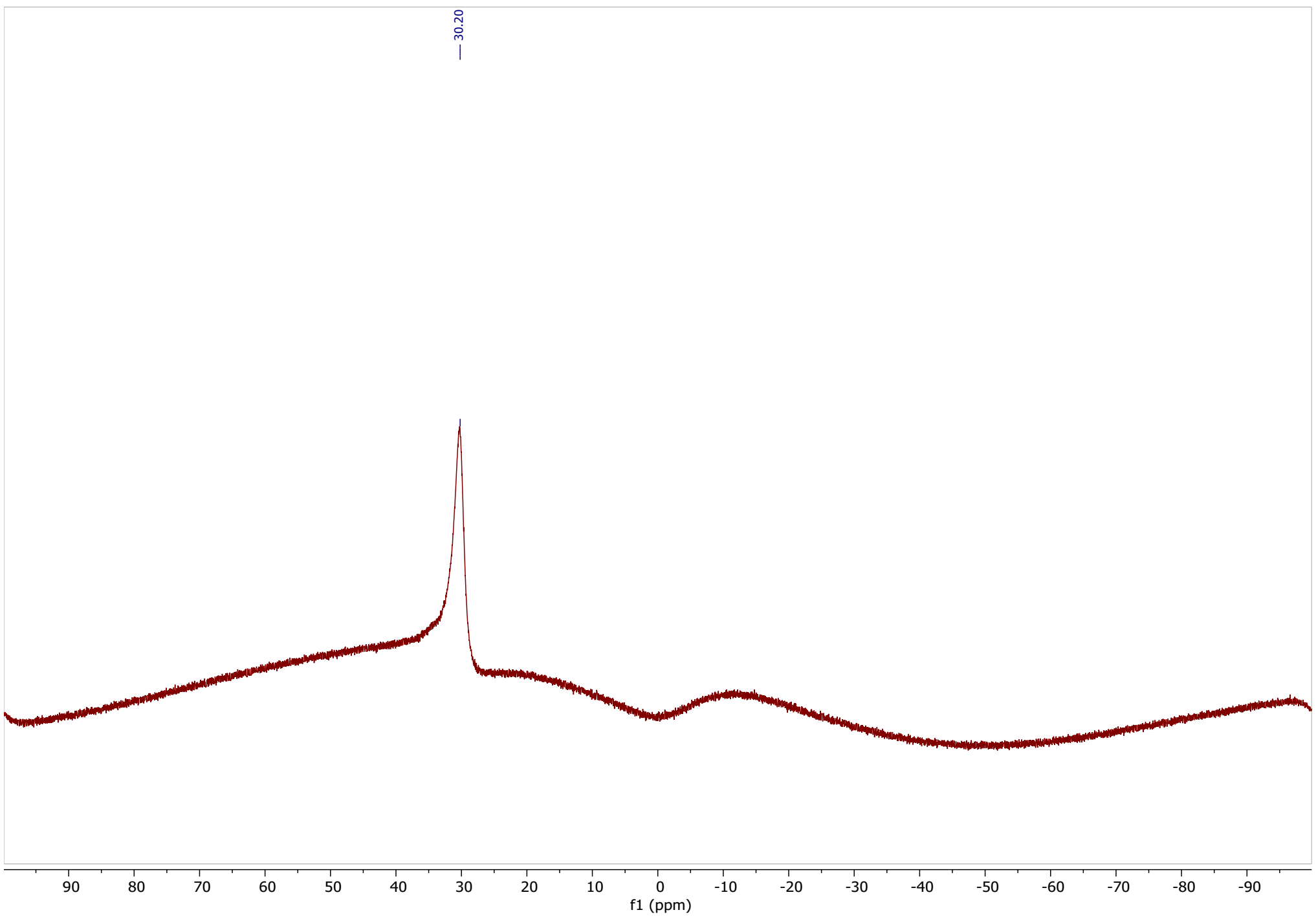

3-(4,4,5,5-tetramethyl-1,3,2-dioxaborolan-2-yl)but-3-en-1-yl 4-(N,N-dipropylsulfamoyl)benzoate (1p)

^{1}H NMR (400 MHz, Chloroform-*d*)

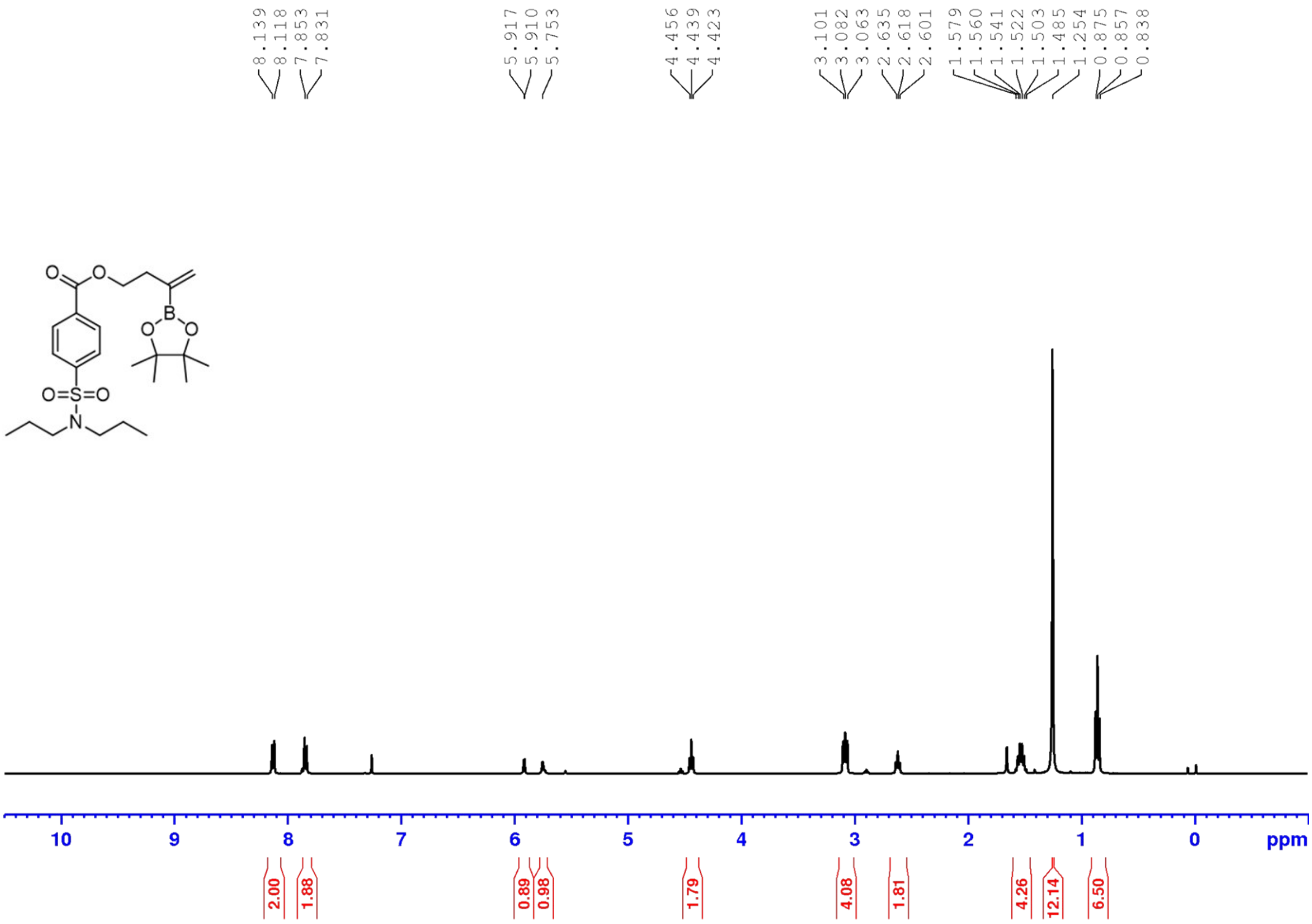

^{13}C NMR (100 MHz, Chloroform-*d*)

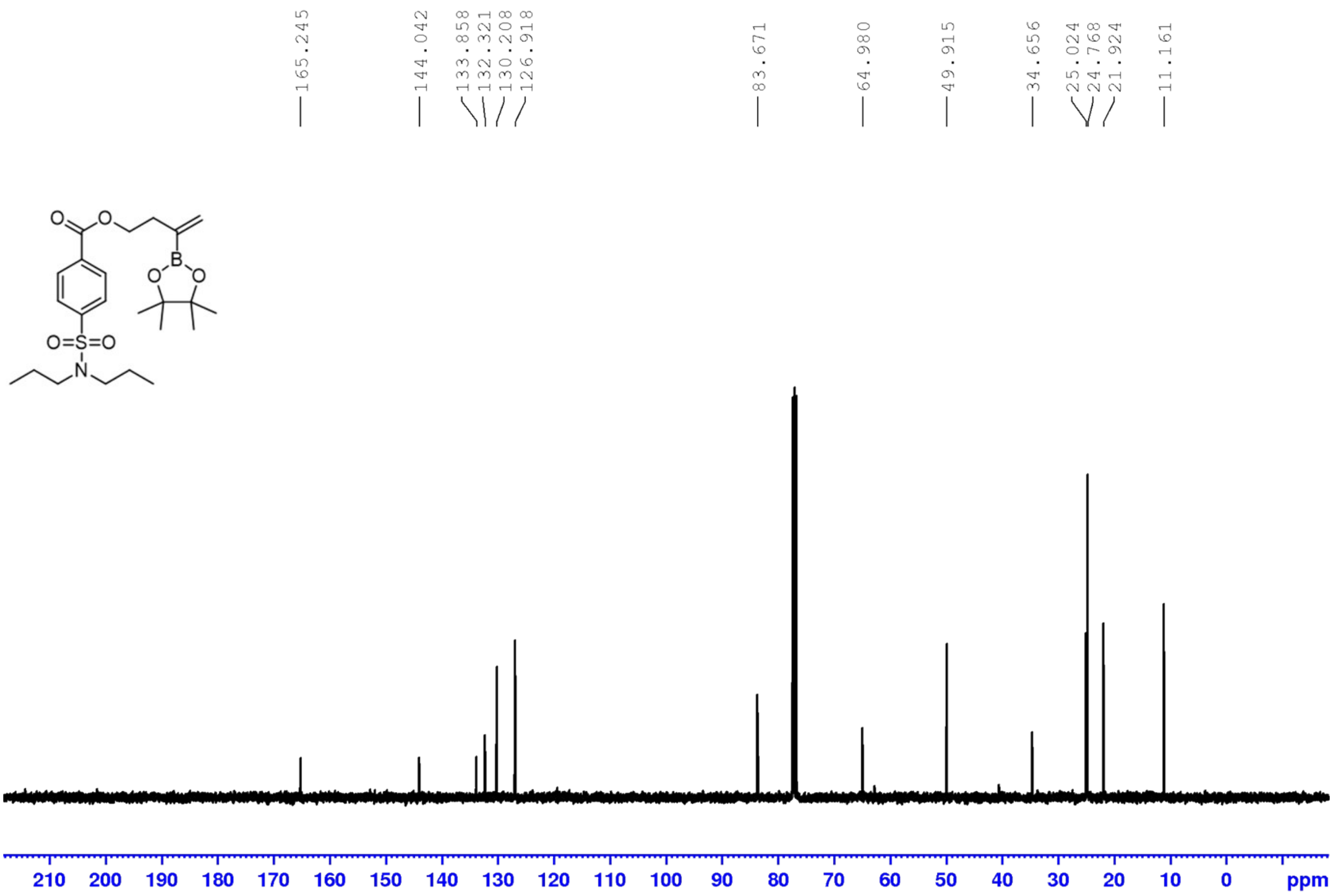

^{11}B NMR (128 MHz, Chloroform-*d*)

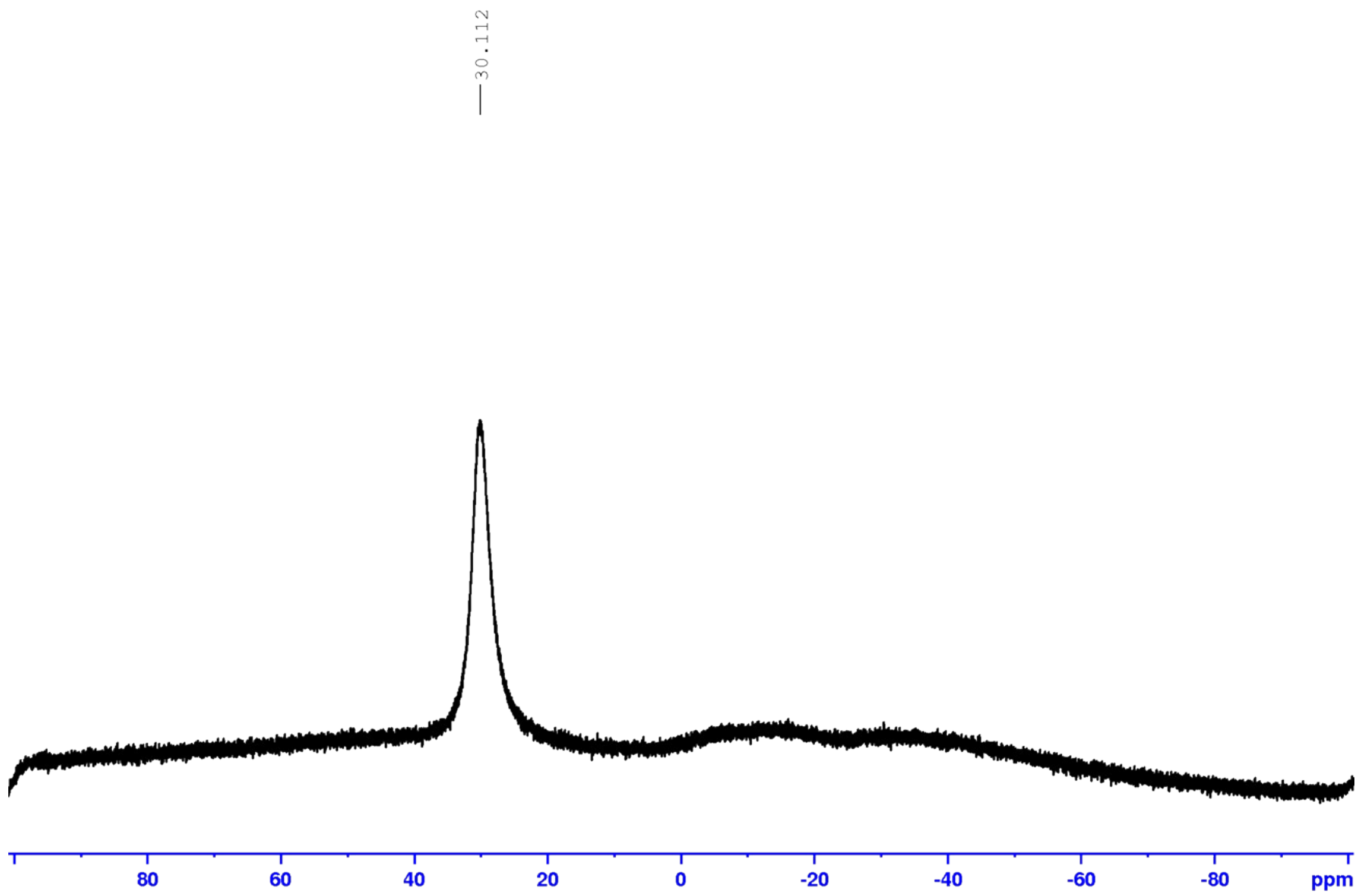

3-(4,4,5,5-tetramethyl-1,3,2-dioxaborolan-2-yl)but-3-en-1-yl 5-(2,5-dimethylphenoxy)-2,2-dimethylpentanoate (1q)

^{1}H NMR (400 MHz, Chloroform-*d*)

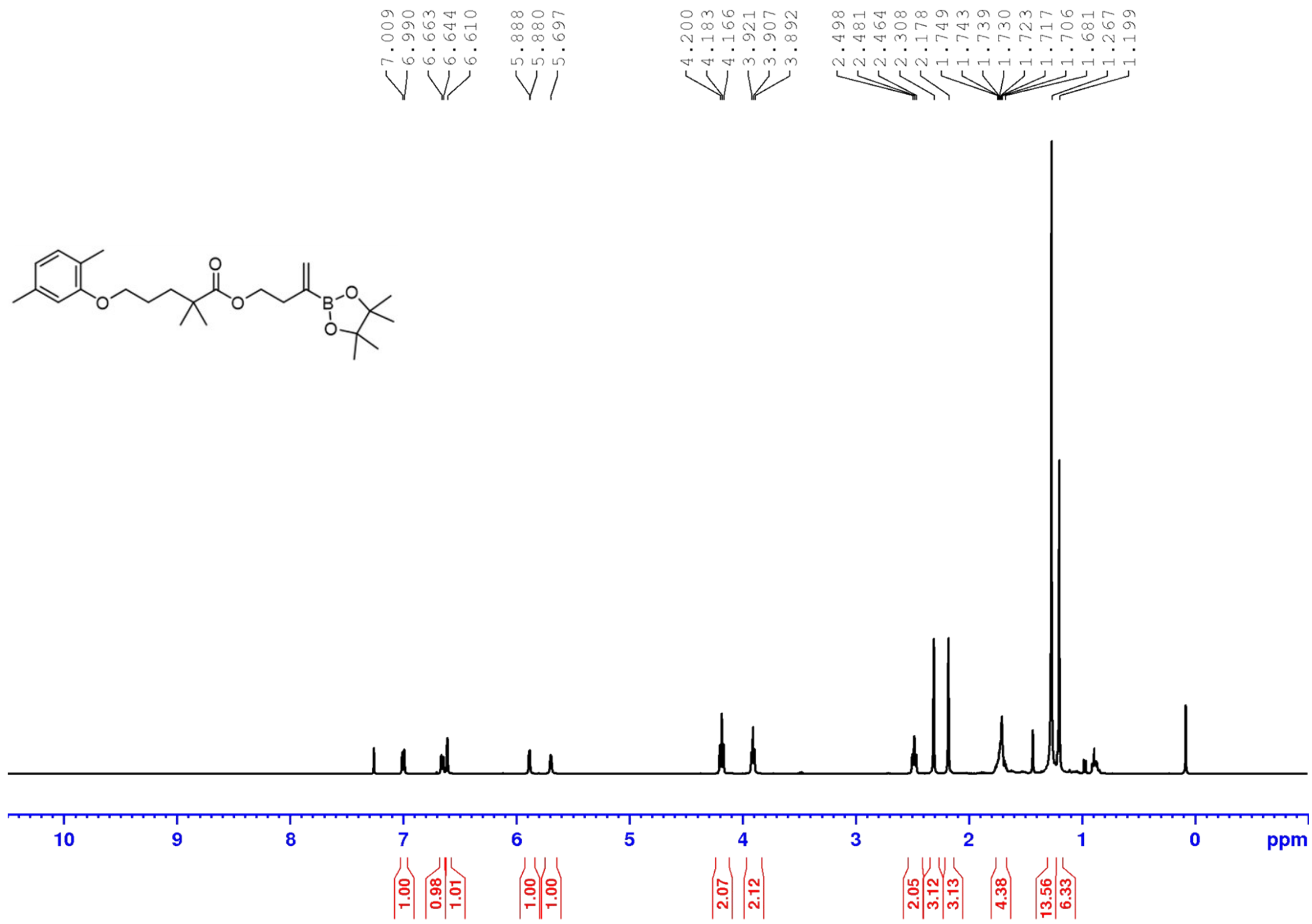

^{13}C NMR (100 MHz, Chloroform-*d*)

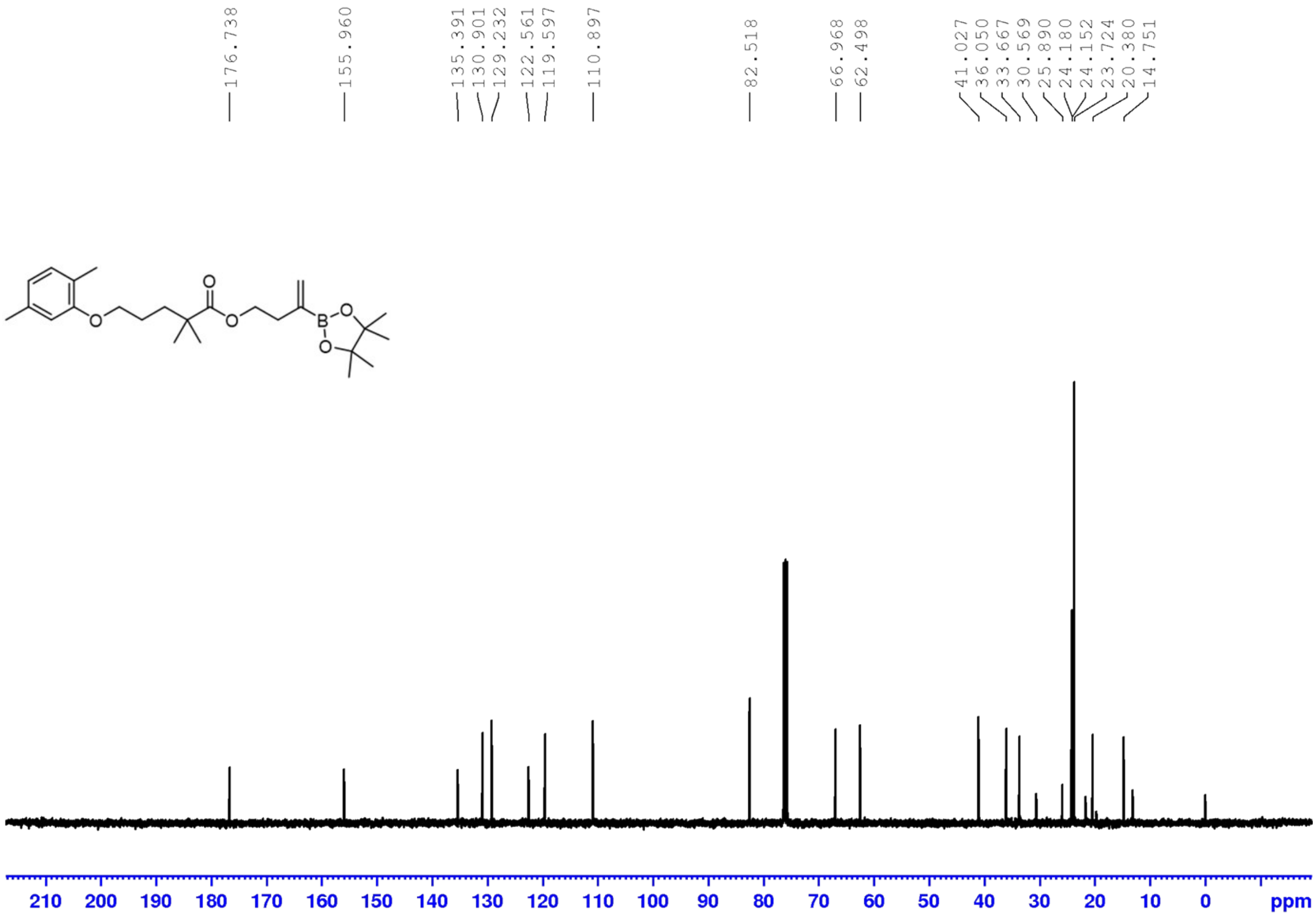

^{11}B NMR (128 MHz, Chloroform-*d*)

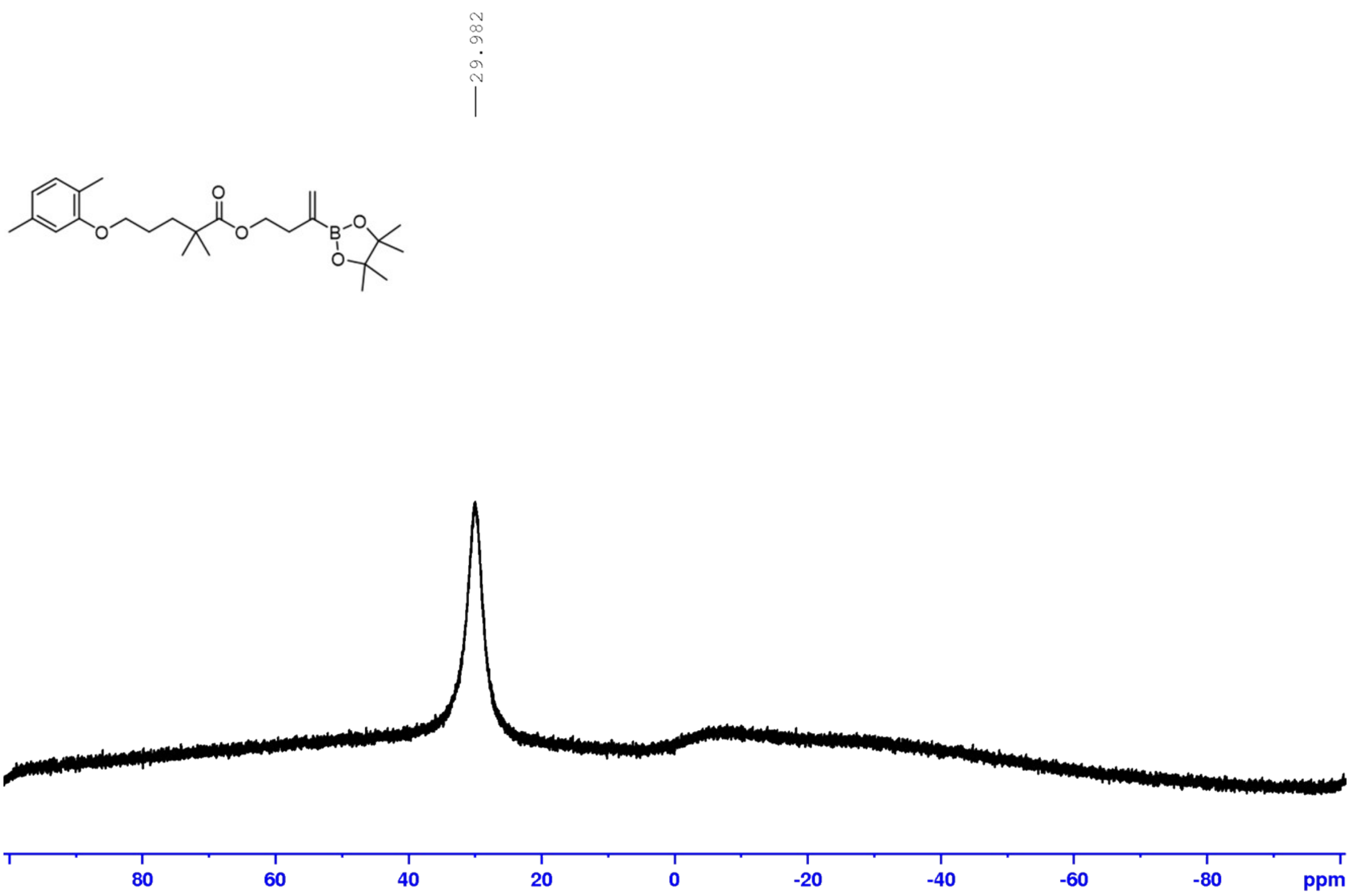

3-(4,4,5,5-tetramethyl-1,3,2-dioxaborolan-2-yl)but-3-en-1-yl 2-(4-isobutylphenyl)propanoate (1r)

^{1}H NMR (400 MHz, Chloroform-*d*)

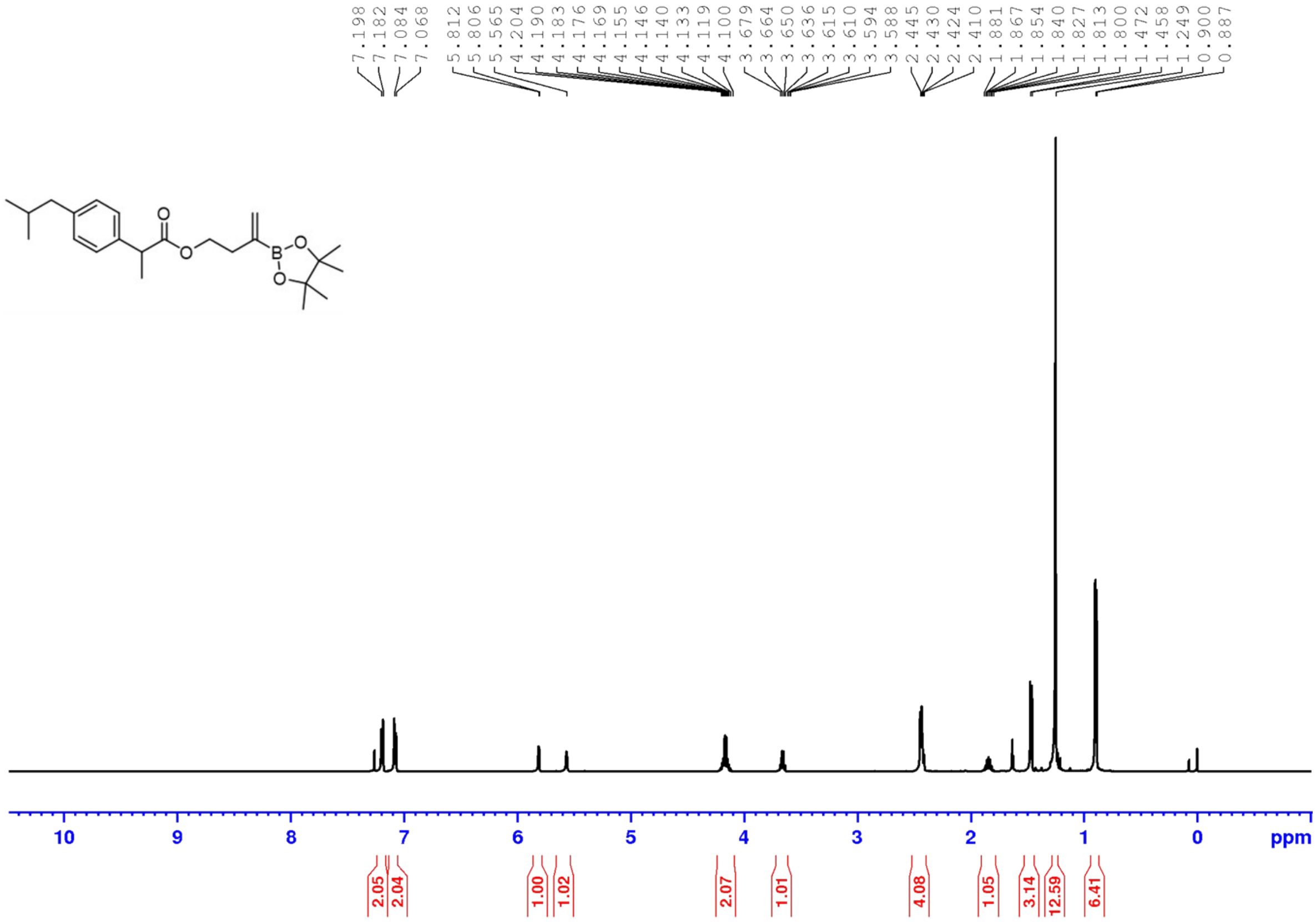

^{13}C NMR (100 MHz, Chloroform-*d*)

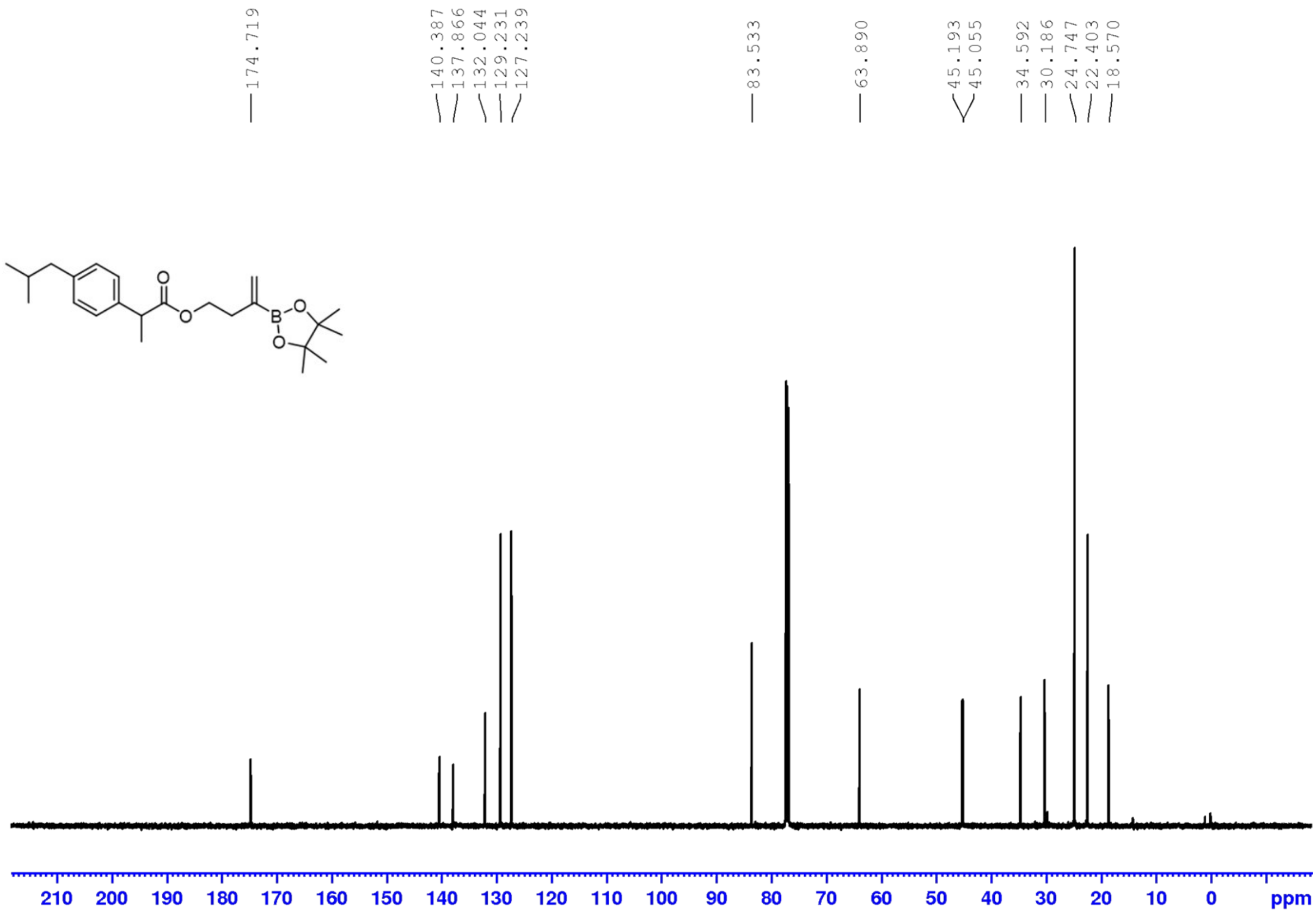

^{11}B NMR (128 MHz, Chloroform-*d*)

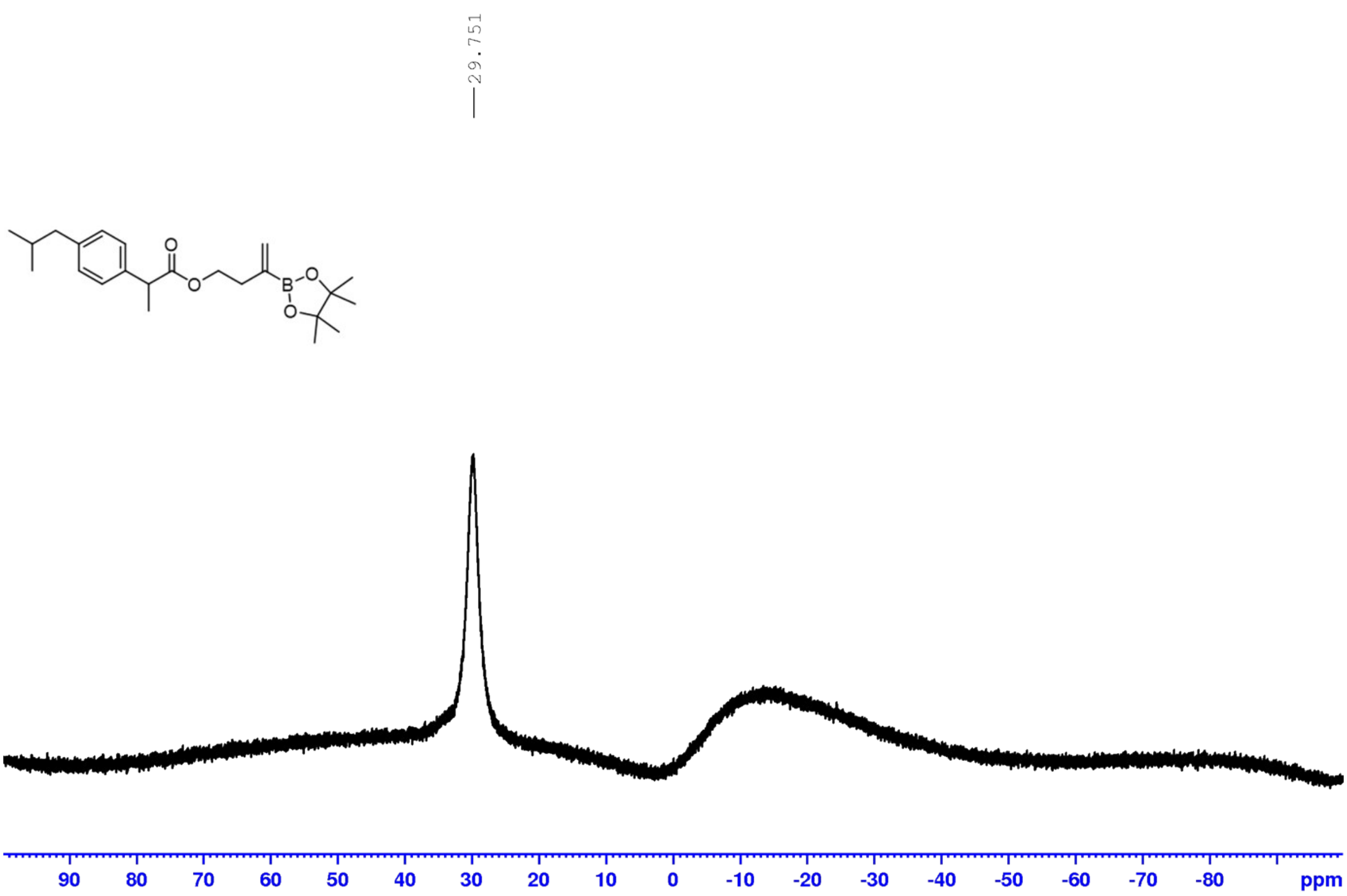

3-(4,4,5,5-tetramethyl-1,3,2-dioxaborolan-2-yl)but-3-en-1-yl (S)-2-(6-methoxynaphthalen-2-yl)propanoate (1s)

^{1}H NMR (400 MHz, Chloroform-*d*)

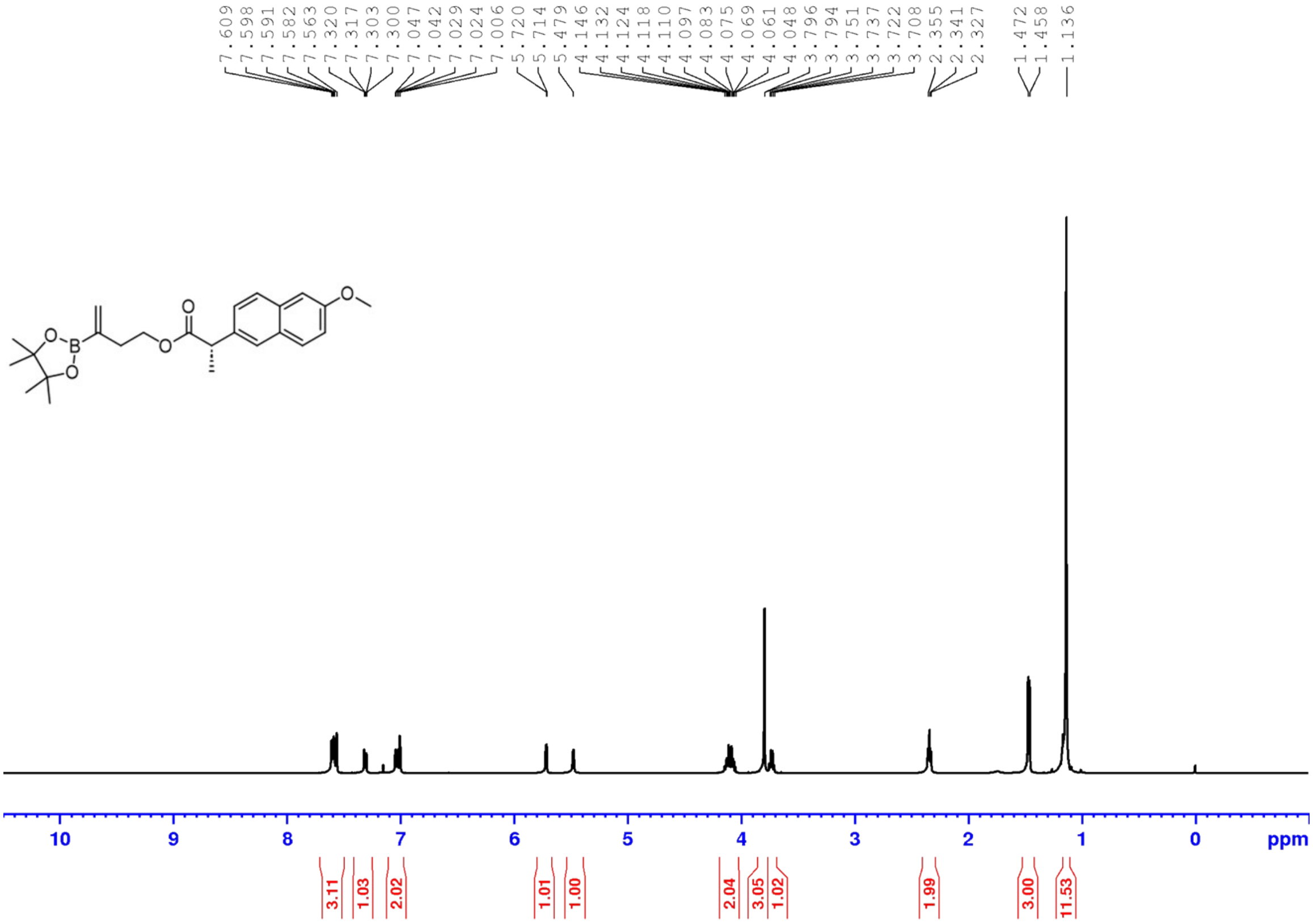

^{13}C NMR (100 MHz, Chloroform-*d*)

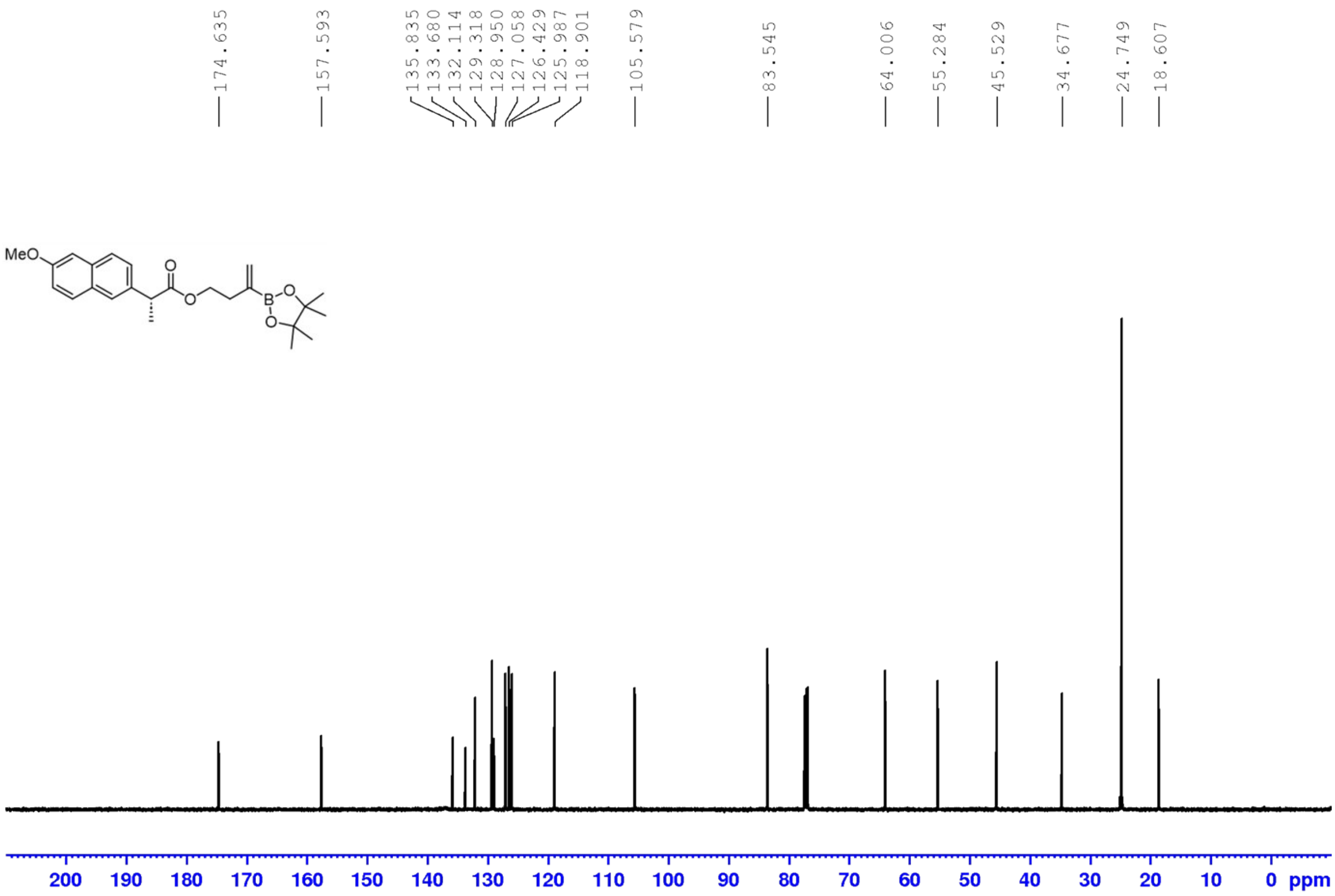

^{11}B NMR (128 MHz, Chloroform-*d*)

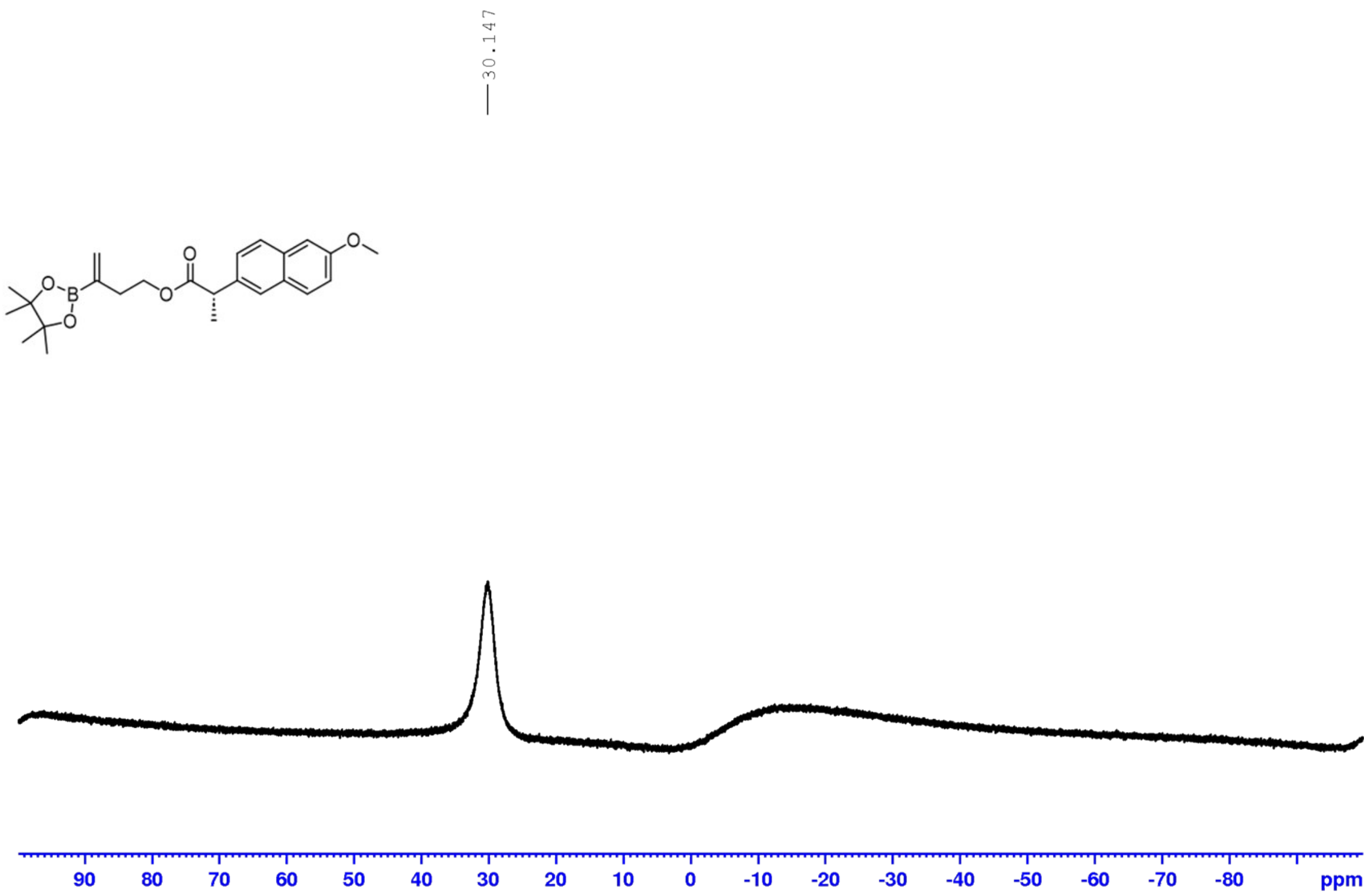

4,4,5,5-tetramethyl-2-(4,4,4-trifluoro-2-(phenylthio)butan-2-yl)-1,3,2-dioxaborolane (3aa)

^{1}H NMR (400 MHz, Chloroform-*d*)

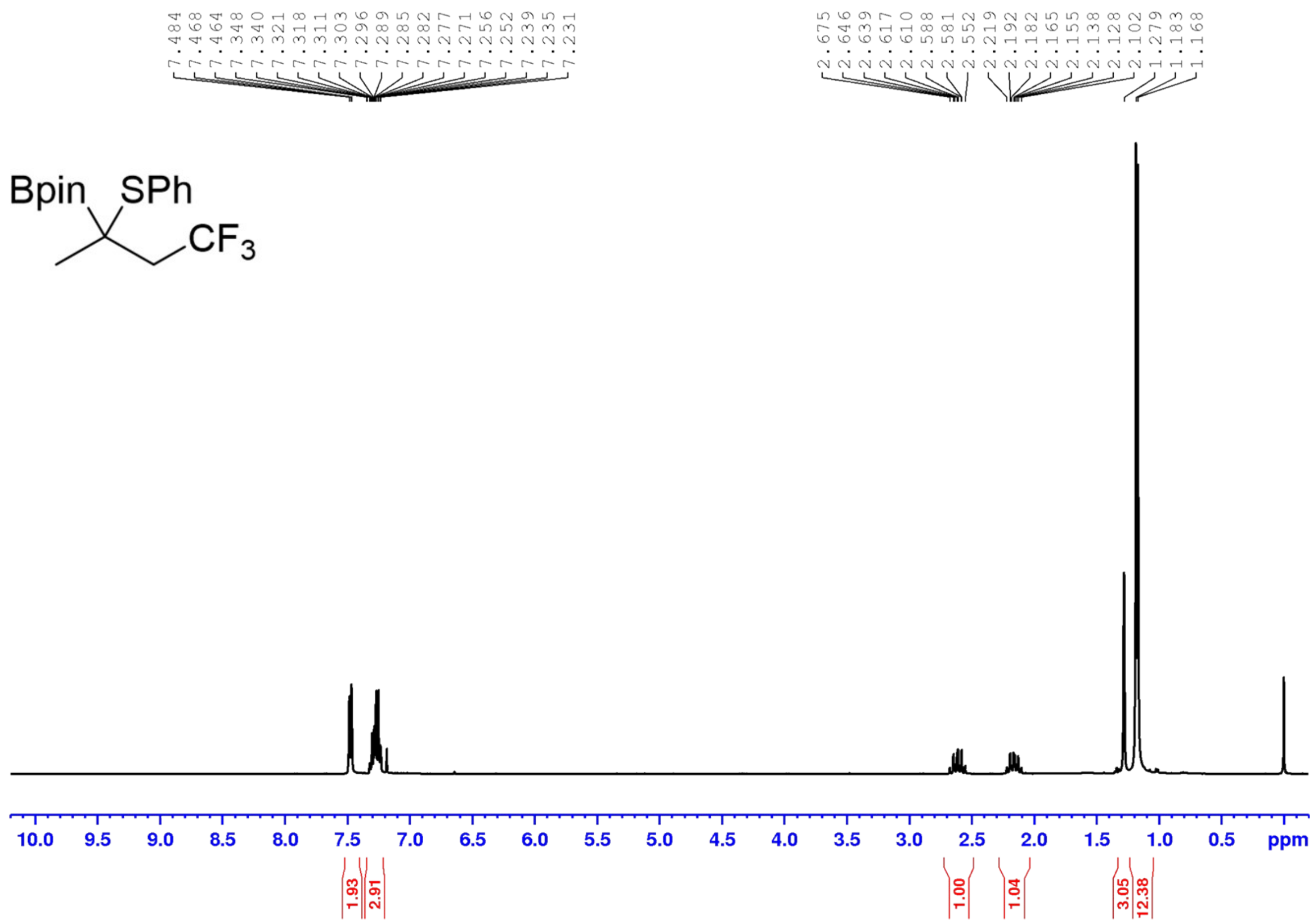

^{13}C NMR (126 MHz, Chloroform-*d*)

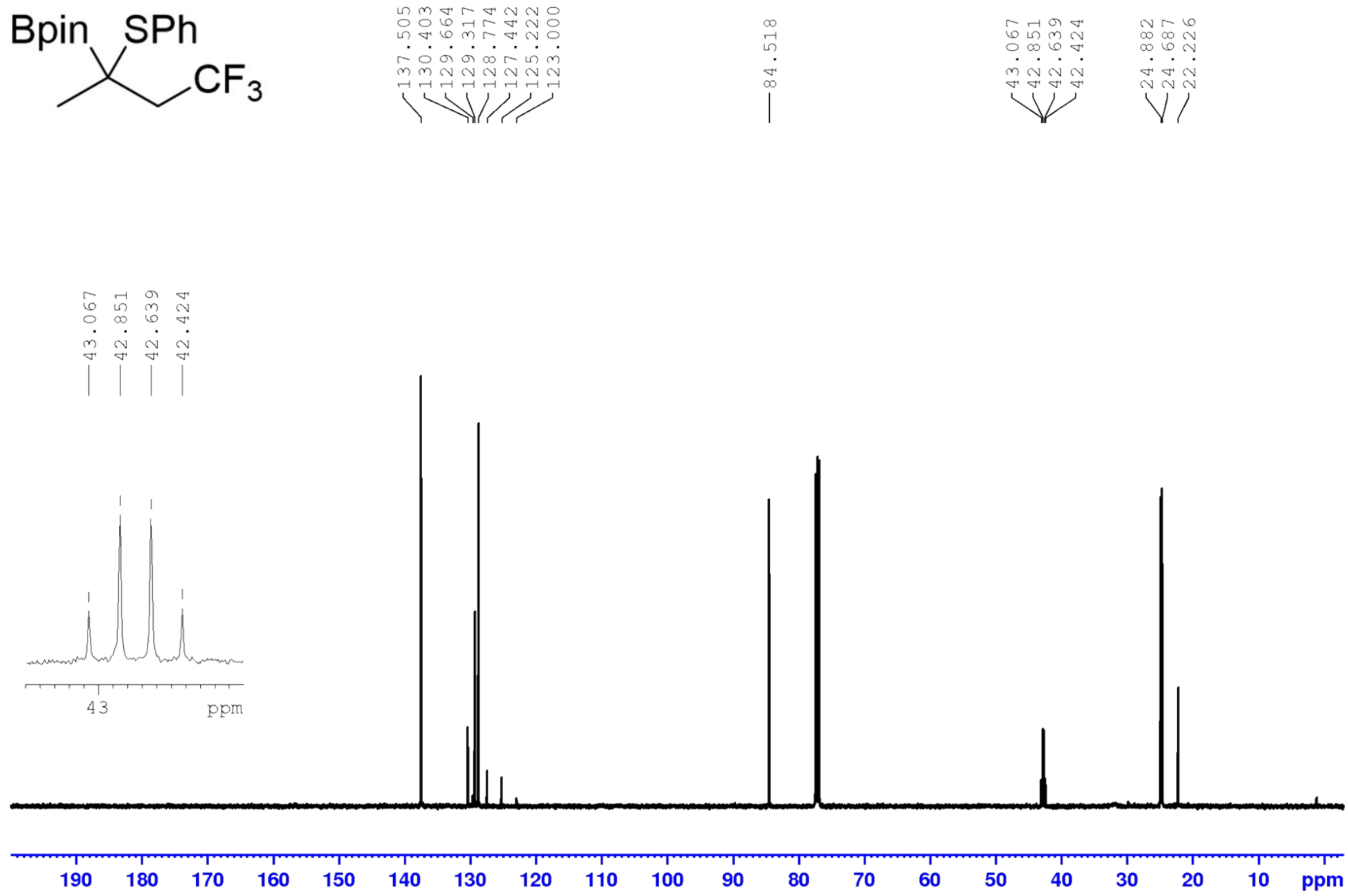

^{11}B NMR (128 MHz, Chloroform-*d*)

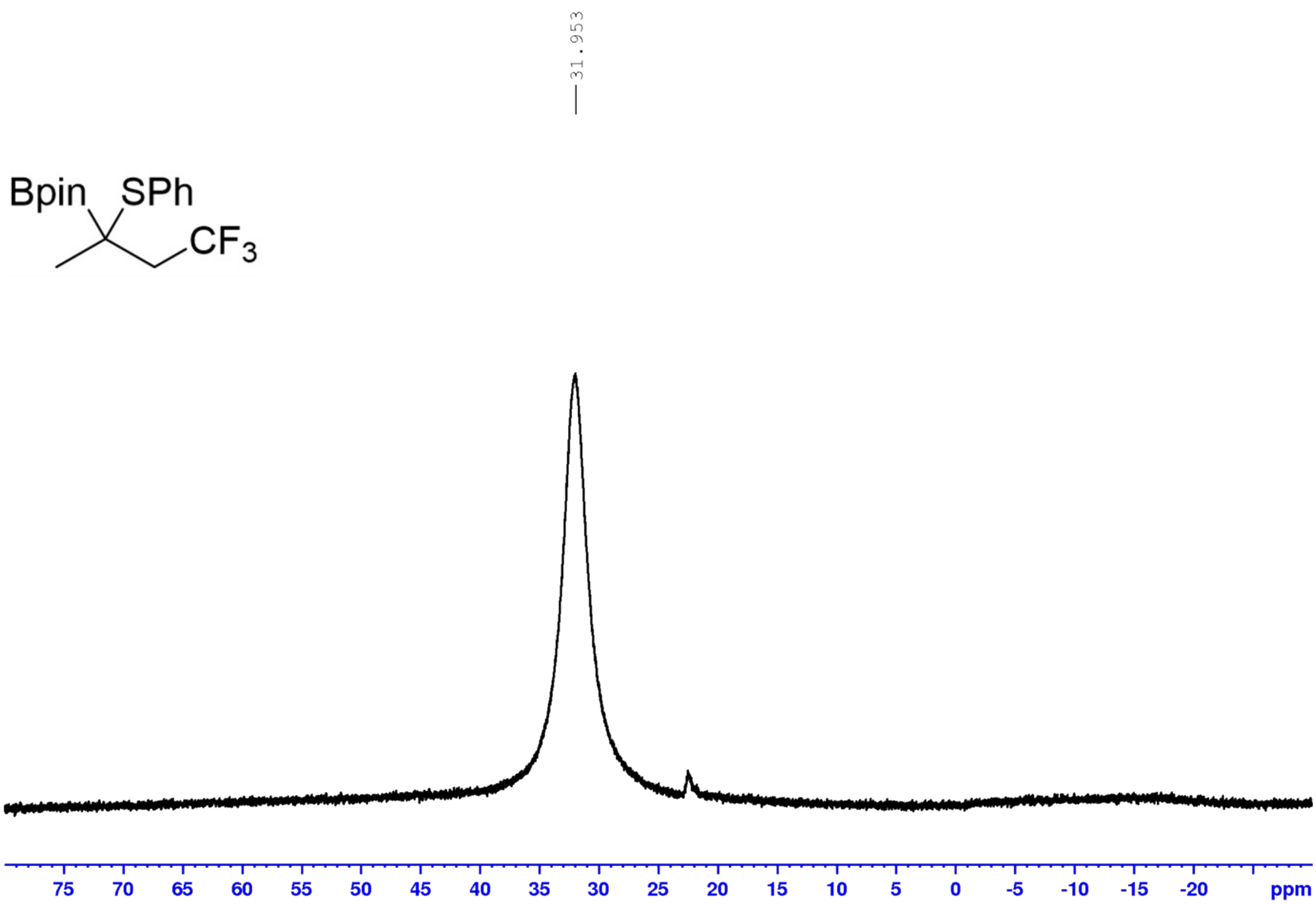

^{19}F NMR (376 MHz, Chloroform-*d*)

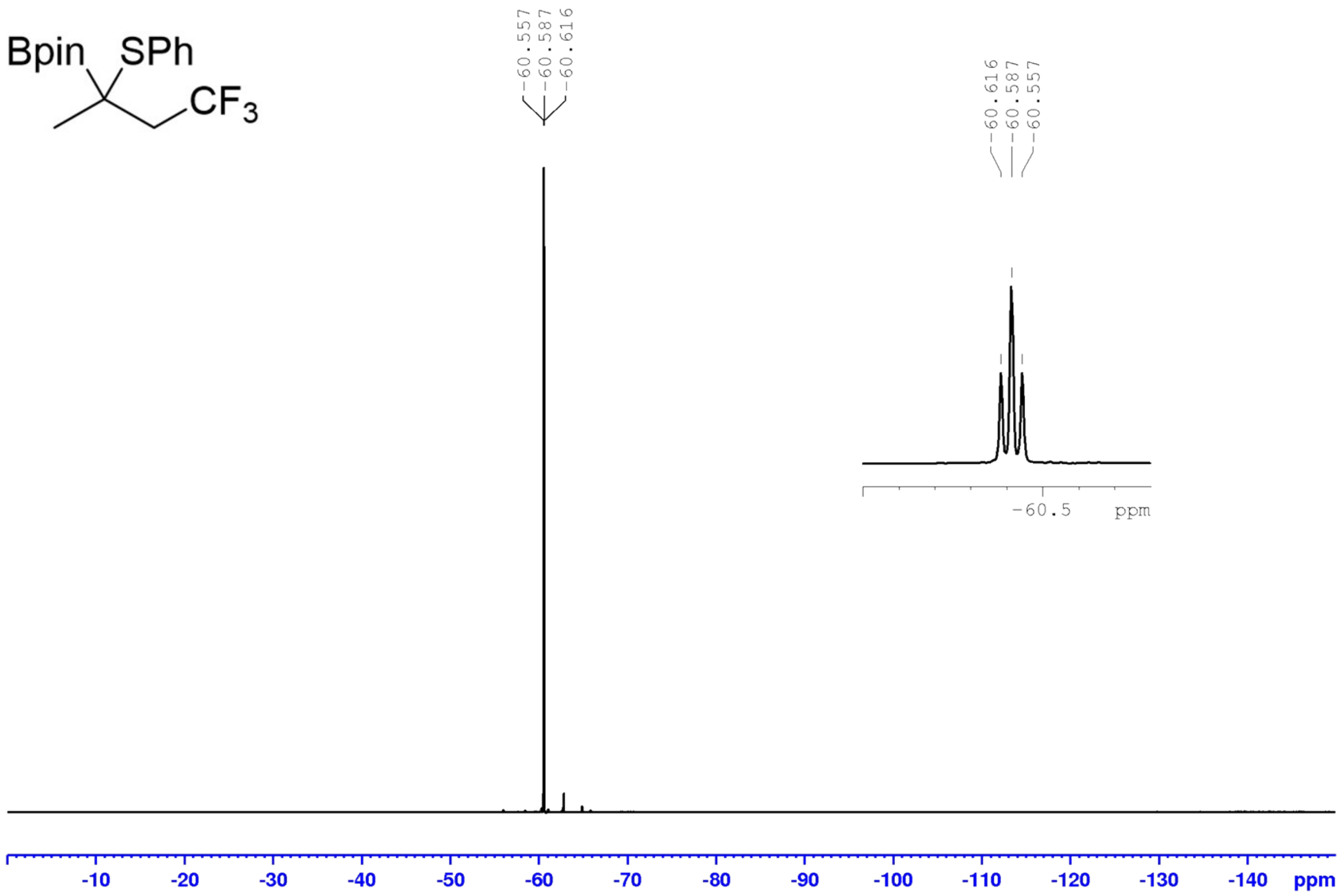

4,4,5,5-tetramethyl-2-(3,3,3-trifluoro-1-phenyl-1-(phenylthio)propyl)-1,3,2-dioxaborolane (3ba)

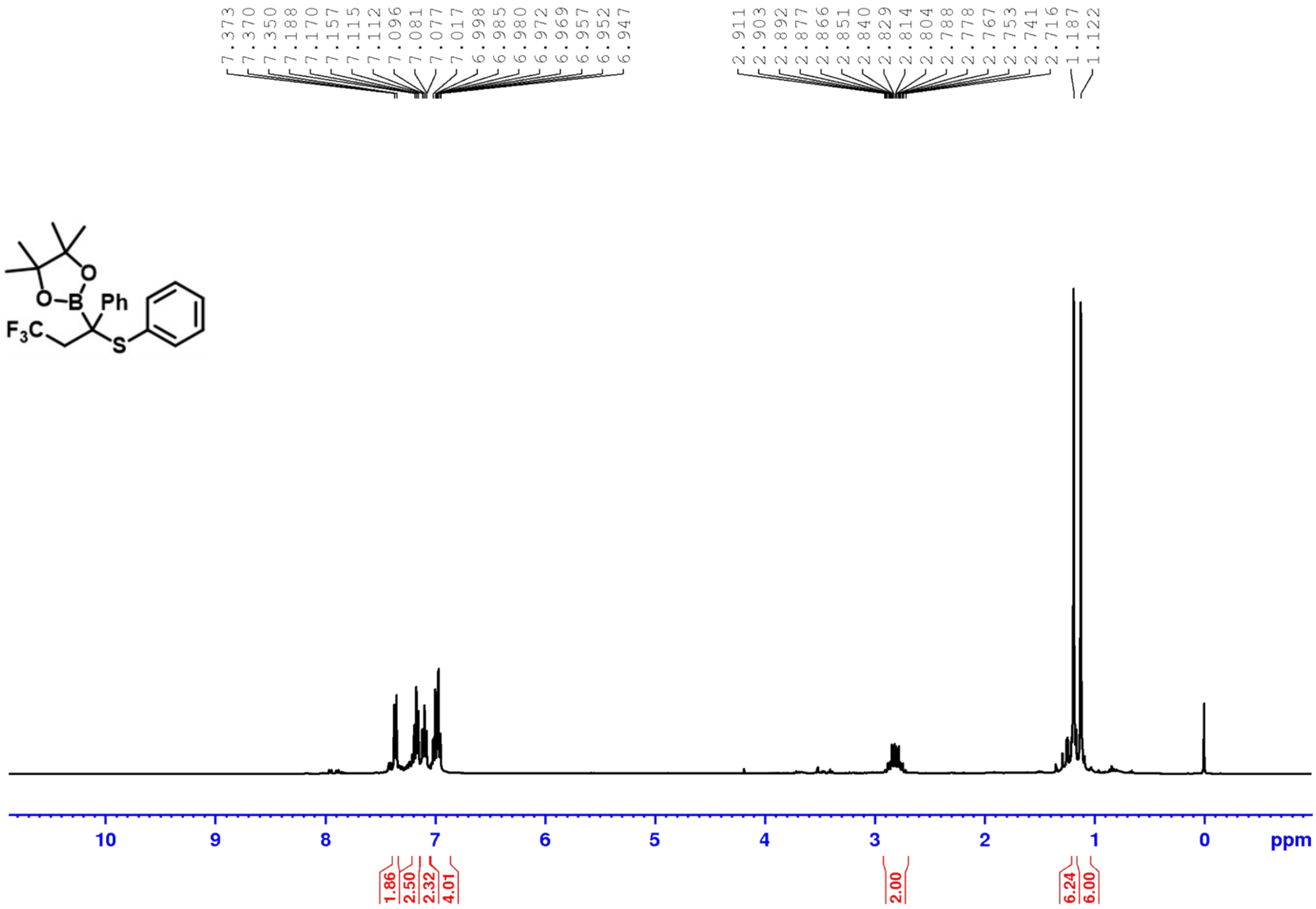

^{1}H NMR (400 MHz, Chloroform-*d*)

^{13}C NMR (100 MHz, Chloroform-*d*)

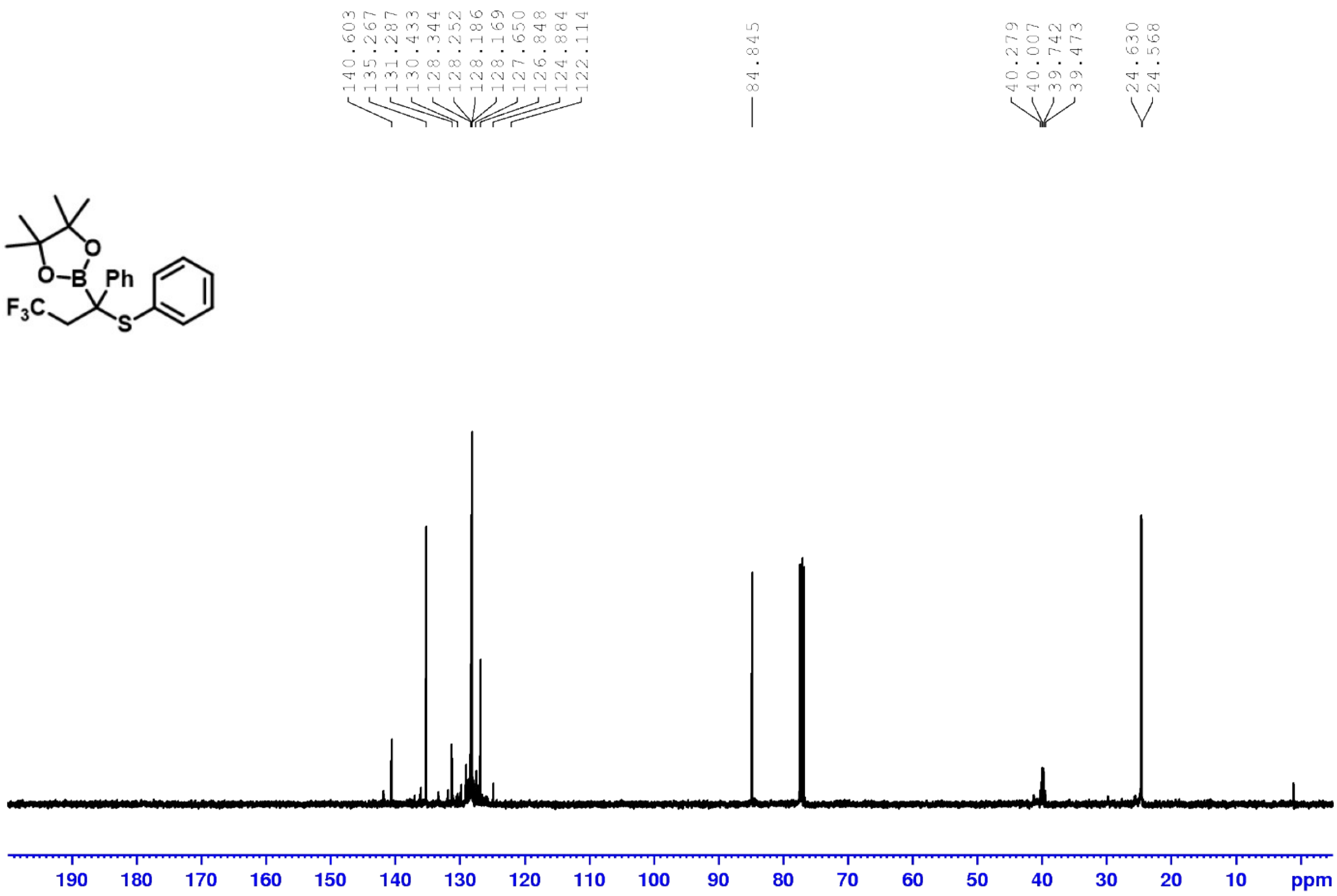

^{11}B NMR (128 MHz, Chloroform-*d*)

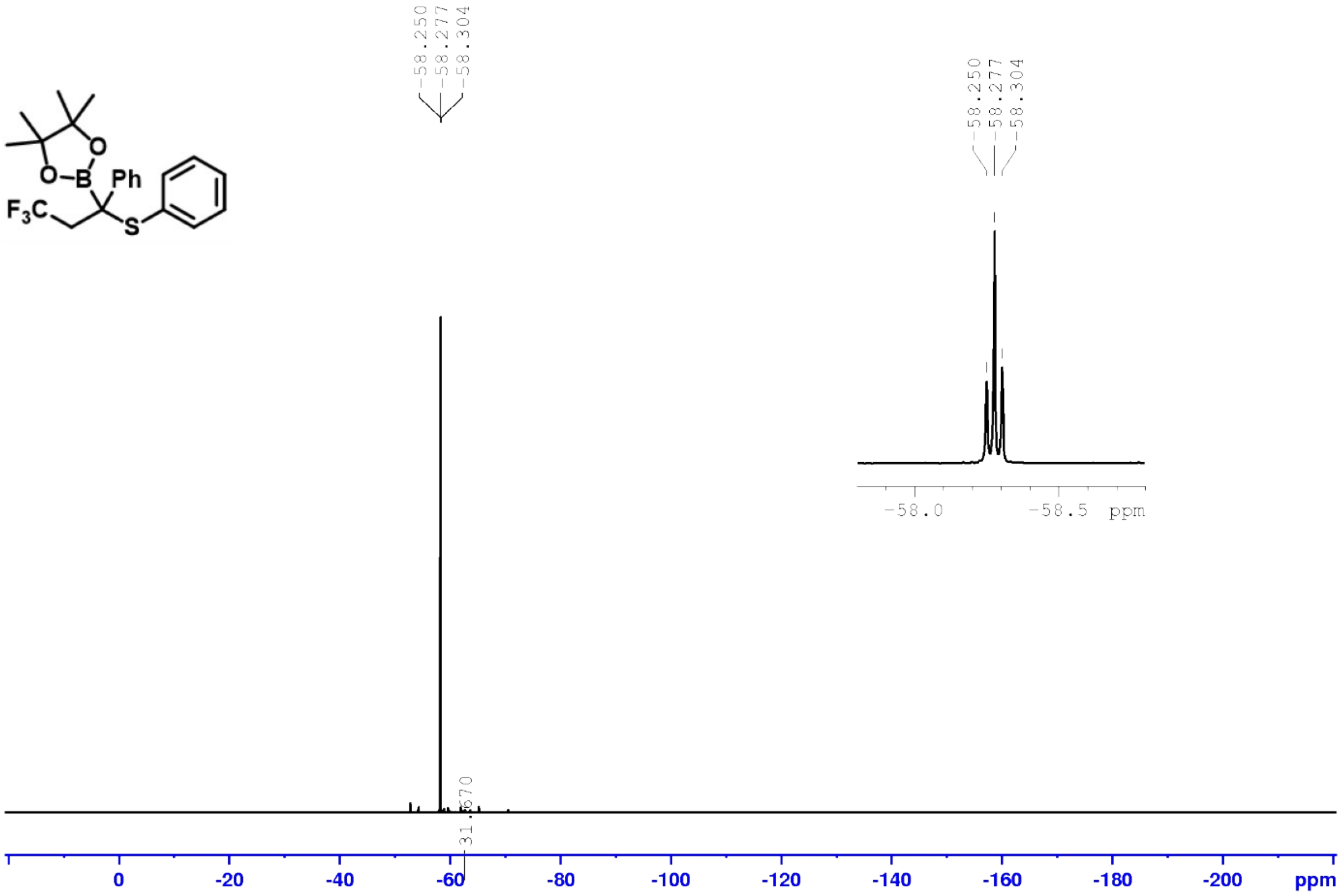

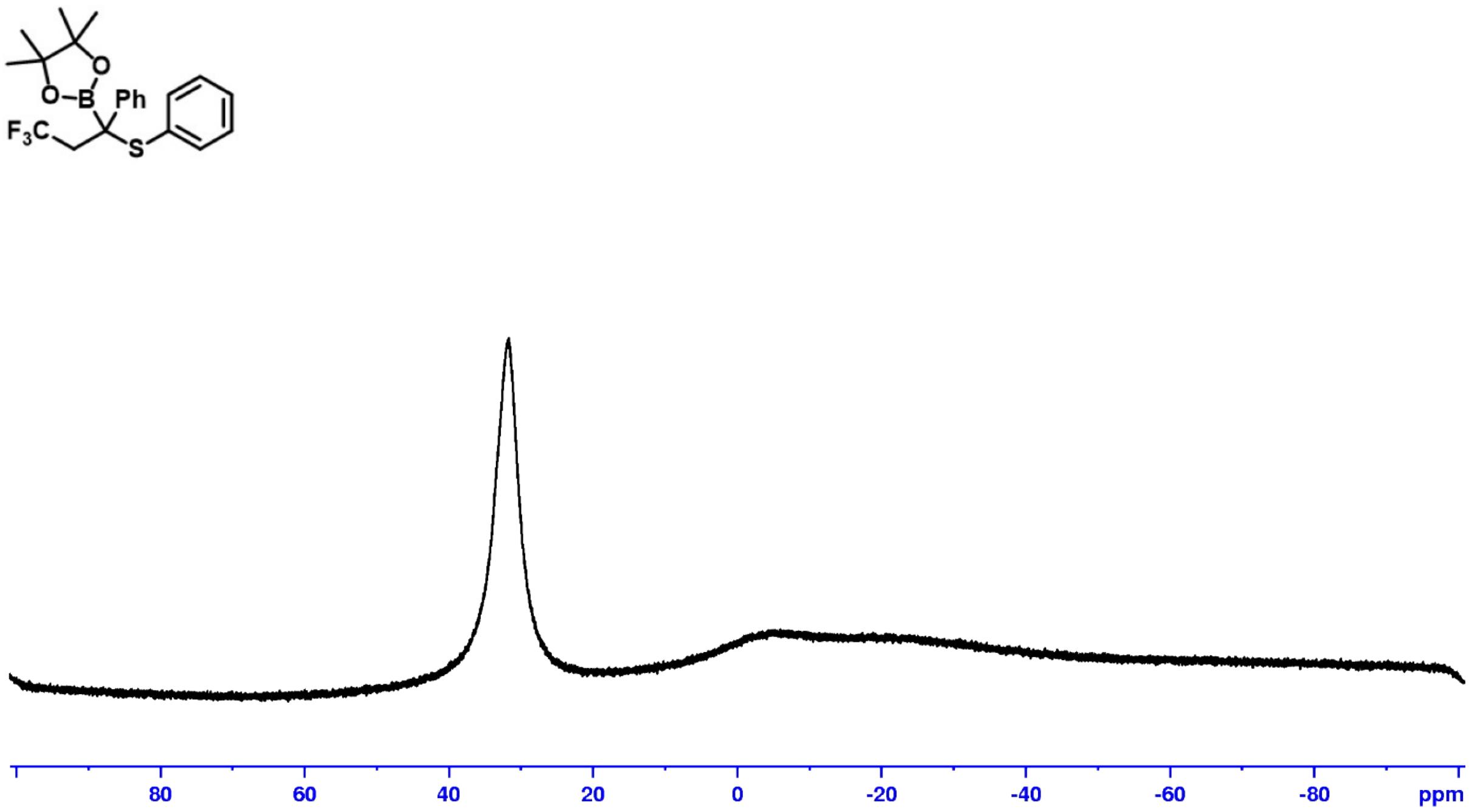

^{19}F NMR (376 MHz, Chloroform-*d*)

4,4,5,5-tetramethyl-2-(4,4,4-trifluoro-1-phenyl-2-(phenylthio)butan-2-yl)-1,3,2-dioxaborolane (3ca)

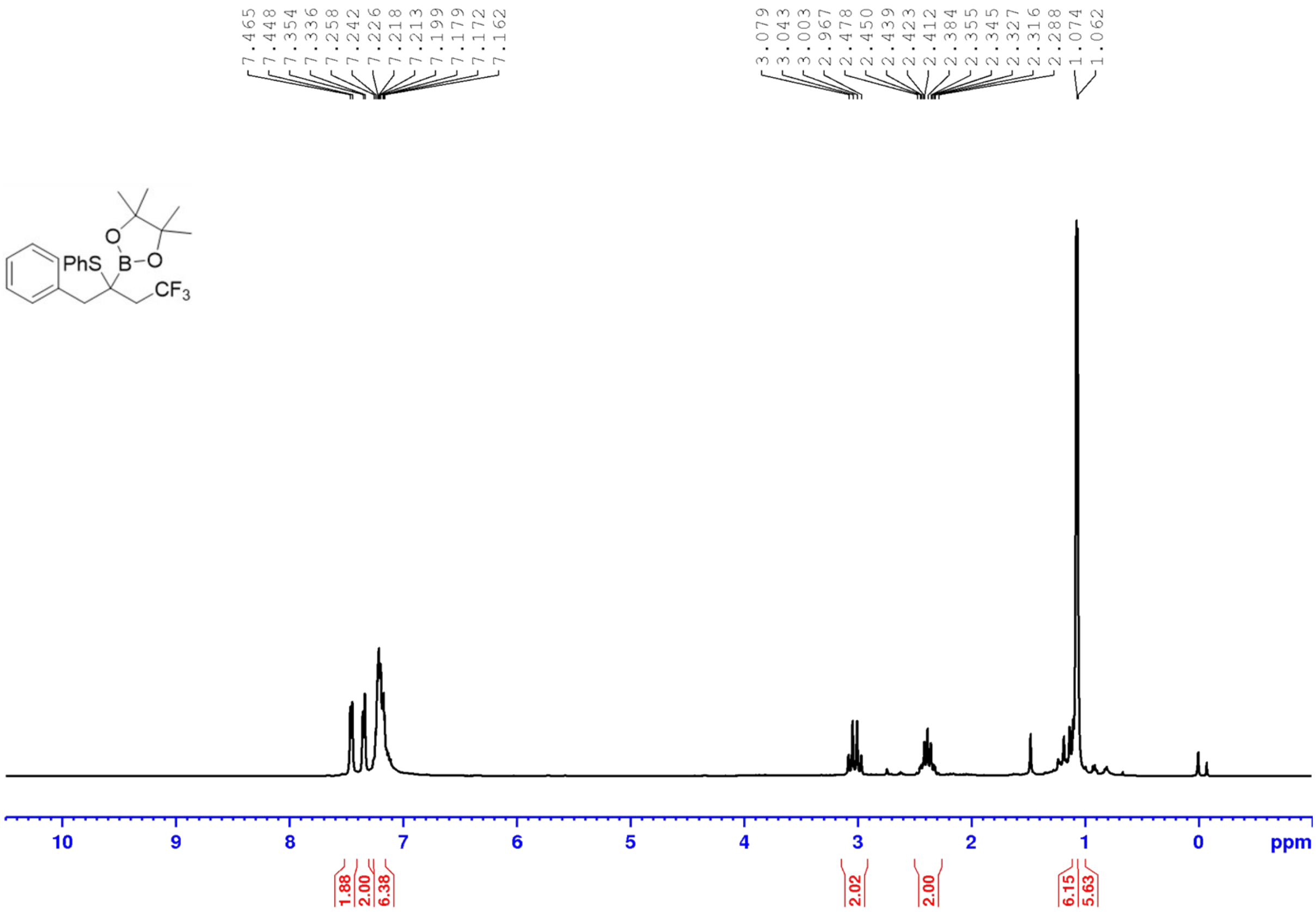

^{13}C NMR (126 MHz, Chloroform-*d*)

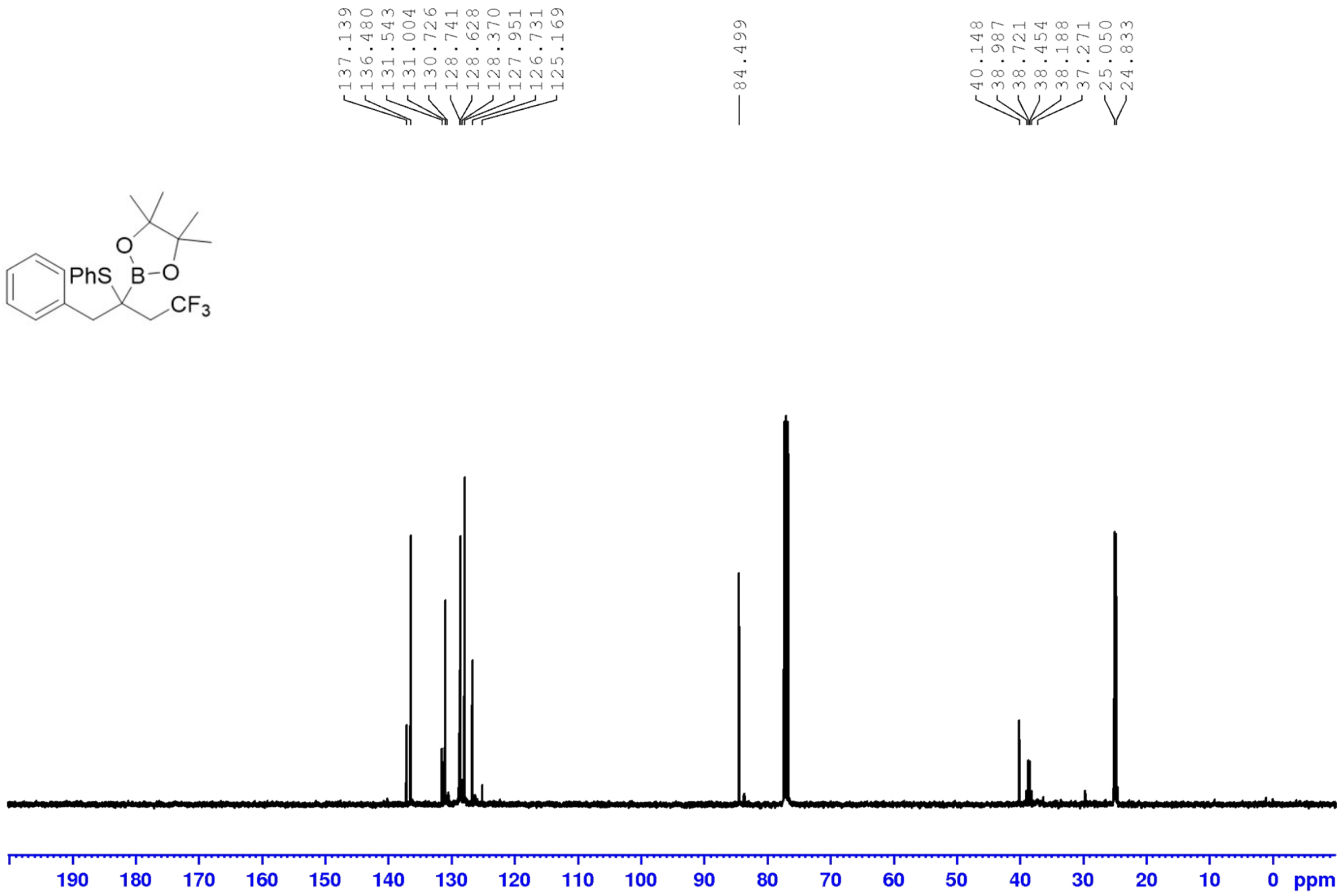

^{11}B NMR (128 MHz, Chloroform-*d*)

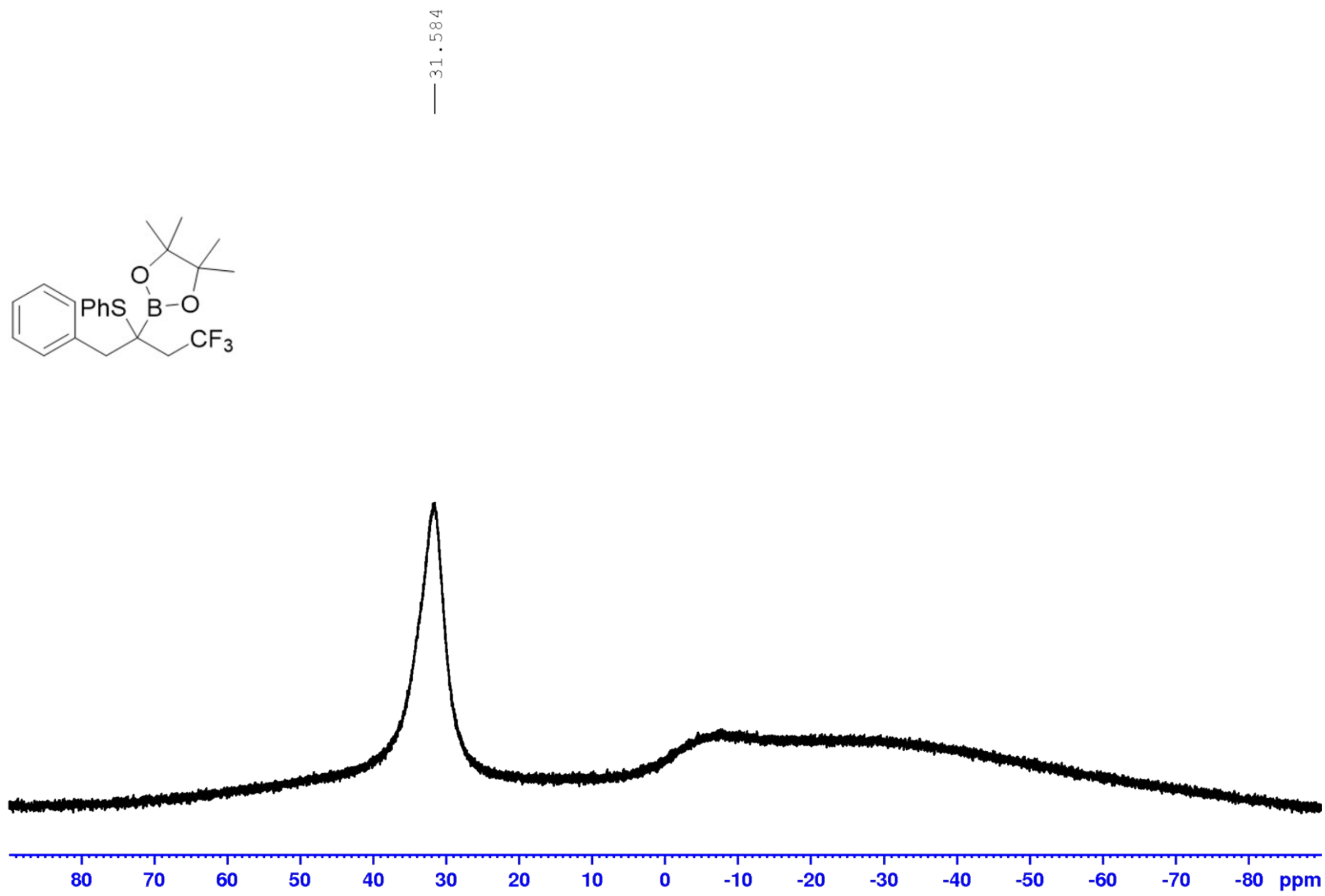

^{19}F NMR (376 MHz, Chloroform-*d*)

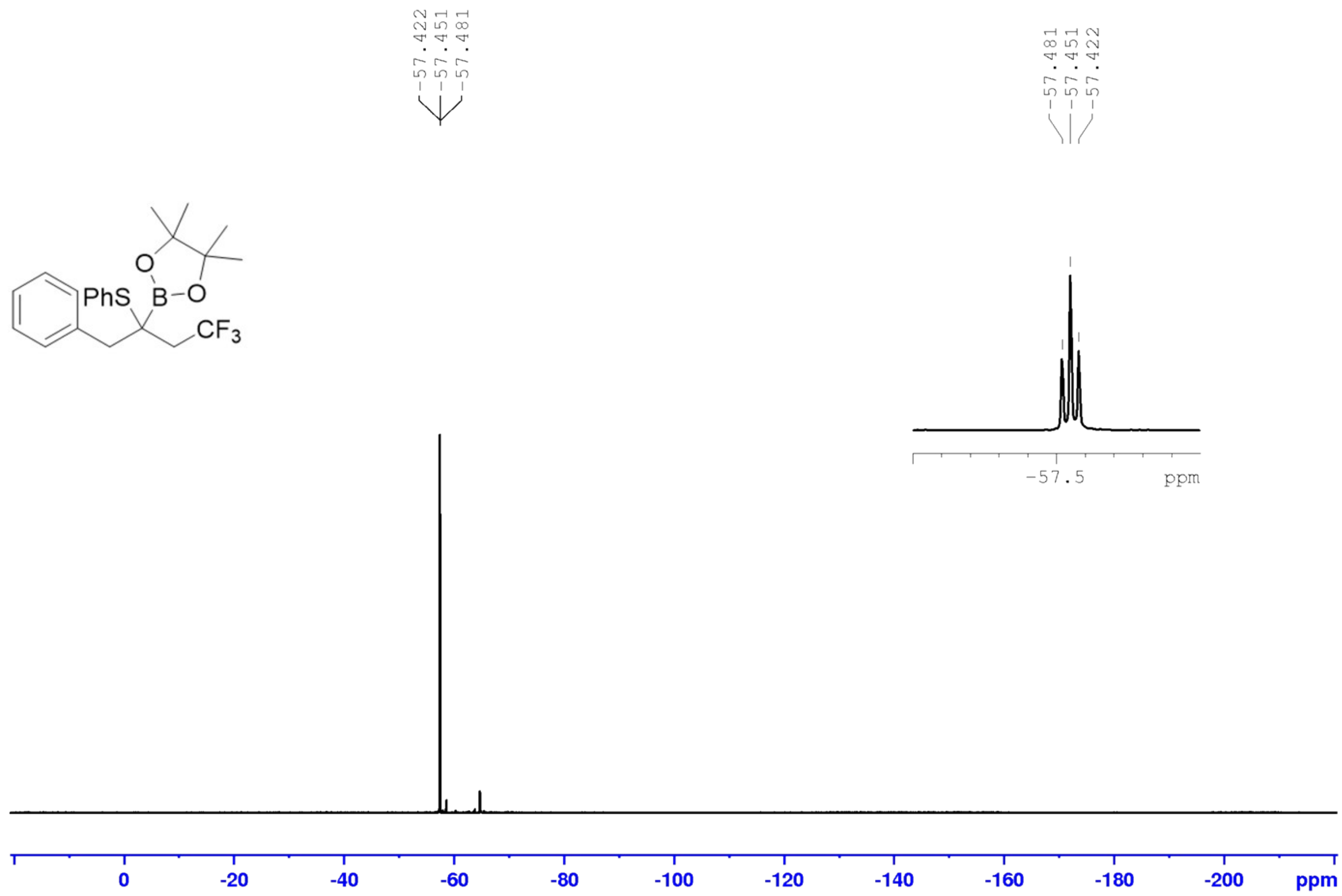

4,4,5,5-tetramethyl-2-(1,1,1-trifluoro-5-phenyl-3-(phenylthio)pentan-3-yl)-1,3,2-dioxaborolane (3da)

^{1}H NMR (400 MHz, Chloroform-*d*)

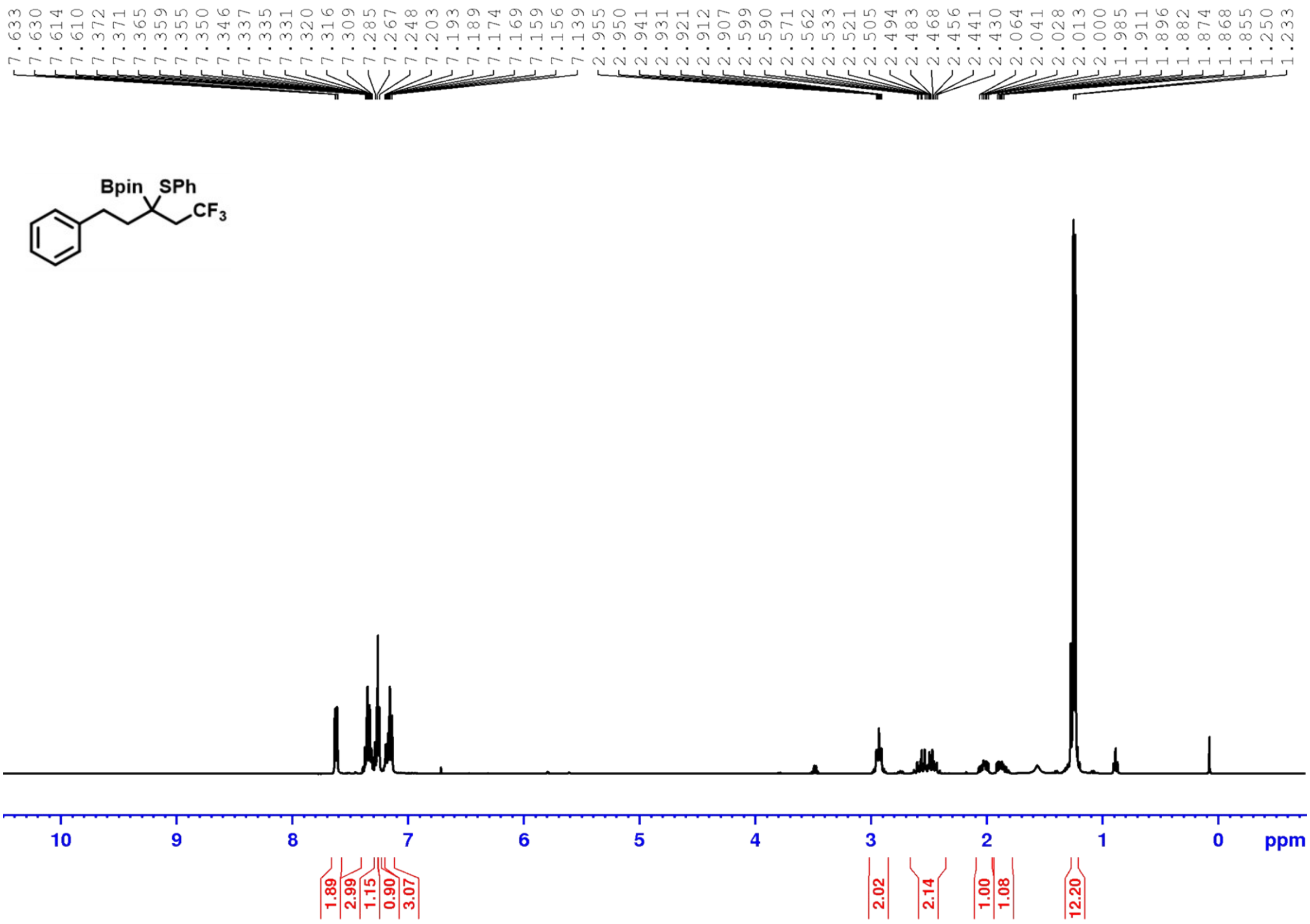

^{13}C NMR (101 MHz, Chloroform-*d*)

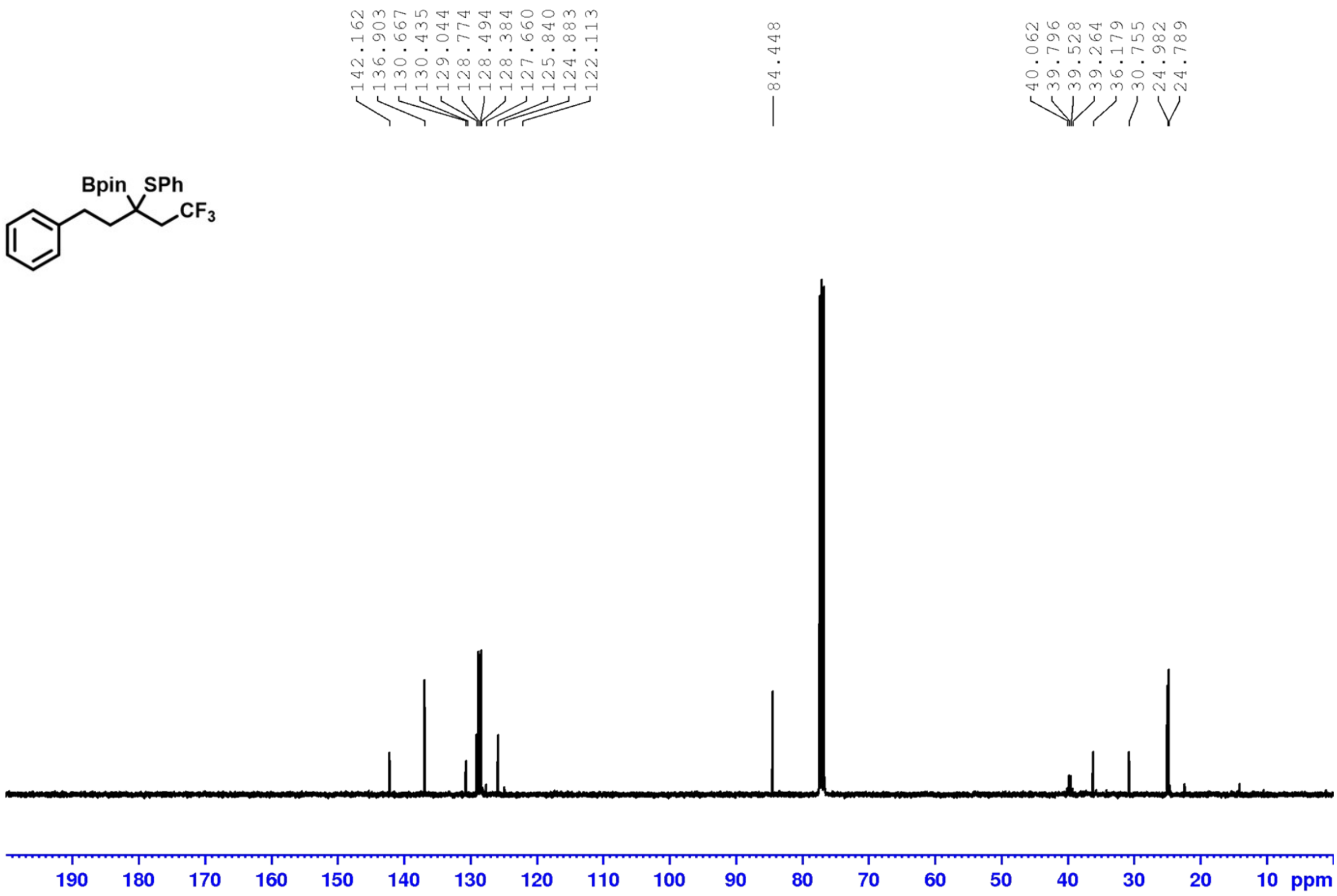

^{11}B NMR (128 MHz, Chloroform-*d*)

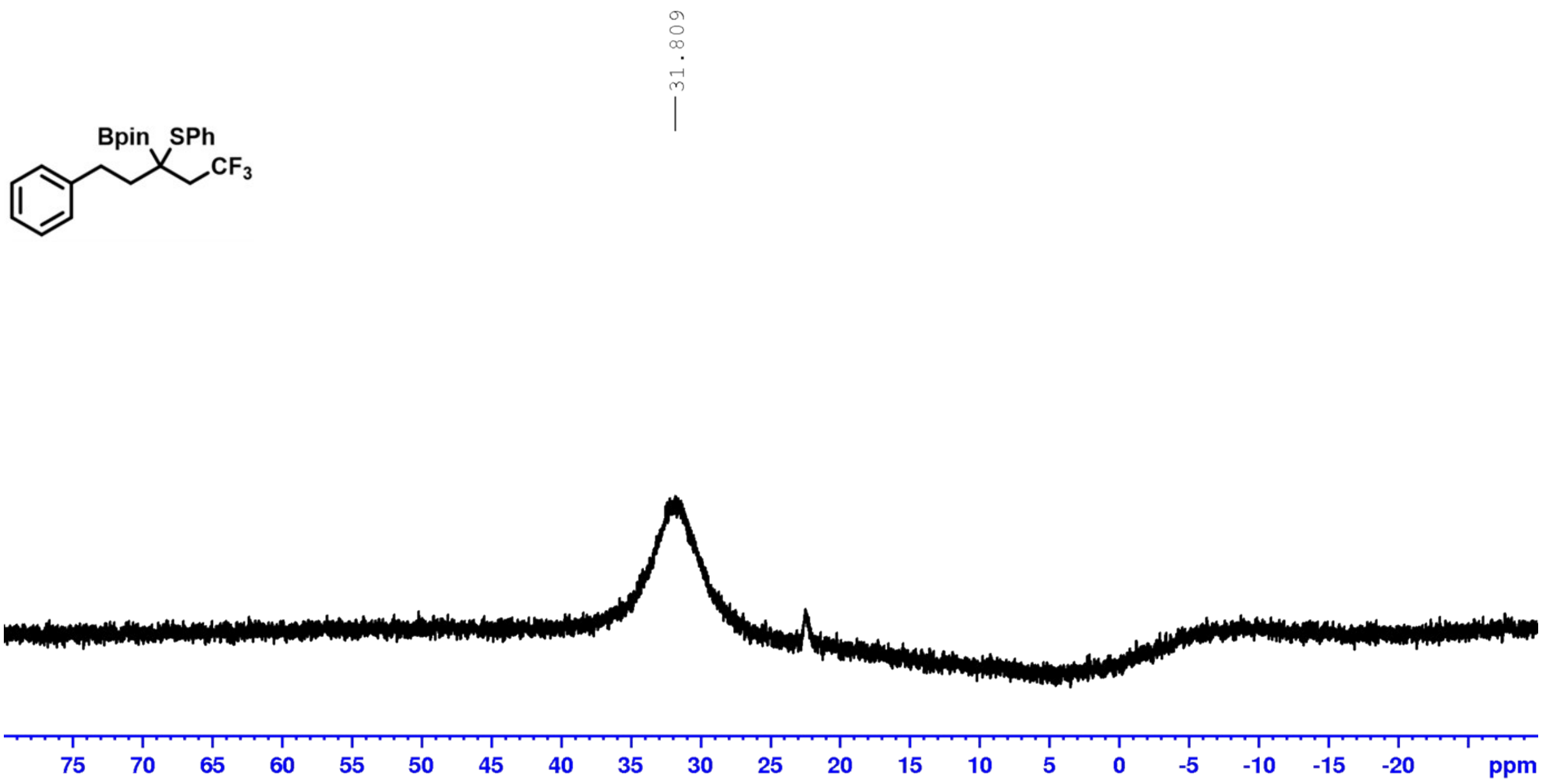

^{19}F NMR (376 MHz, Chloroform-*d*)

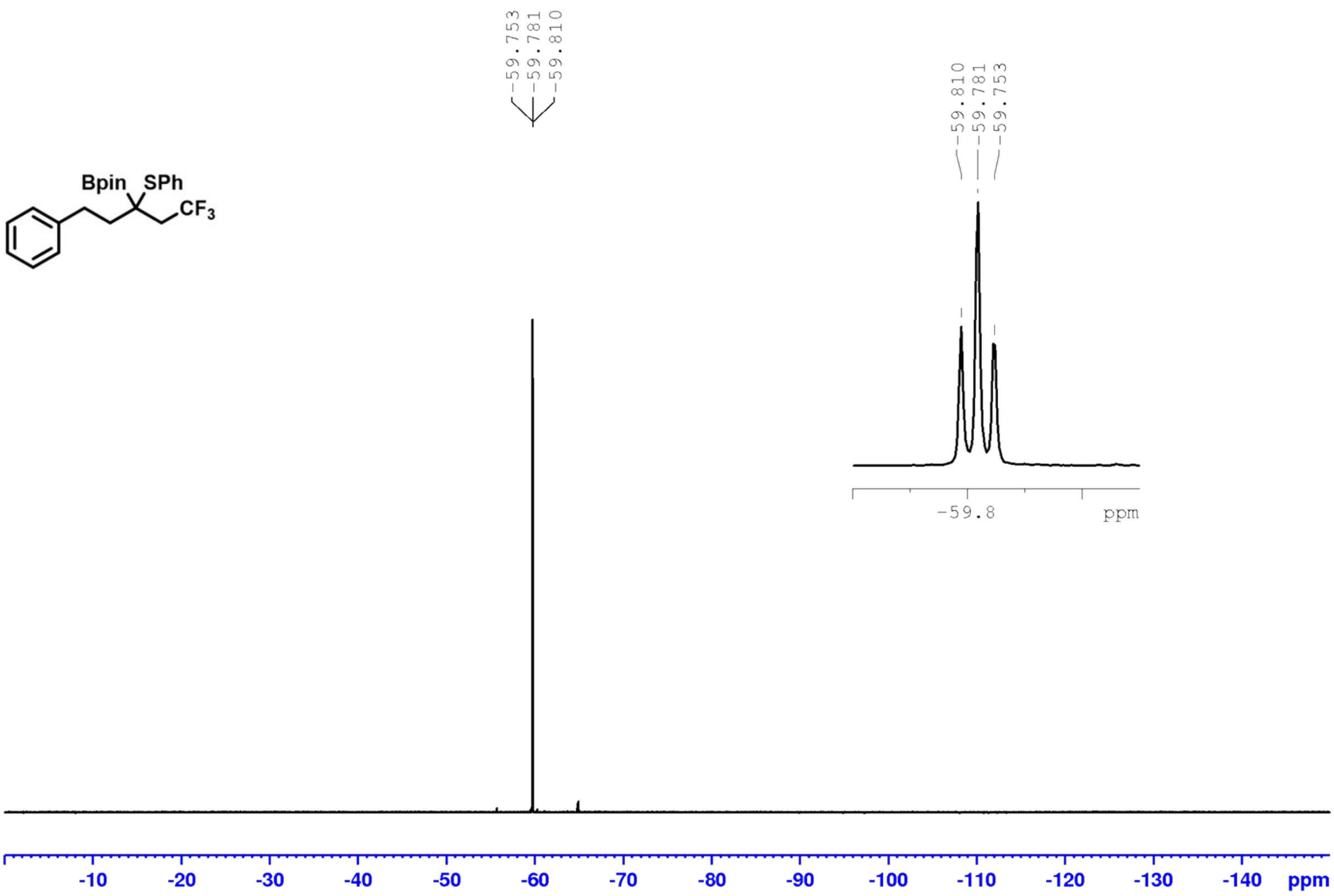

4,4,5,5-tetramethyl-2-(1,1,1-trifluoro-3-(phenylthio)-5-(p-tolyl)pentan-3-yl)-1,3,2-dioxaborolane (3ea)

^{1}H NMR (400 MHz, Chloroform-*d*)

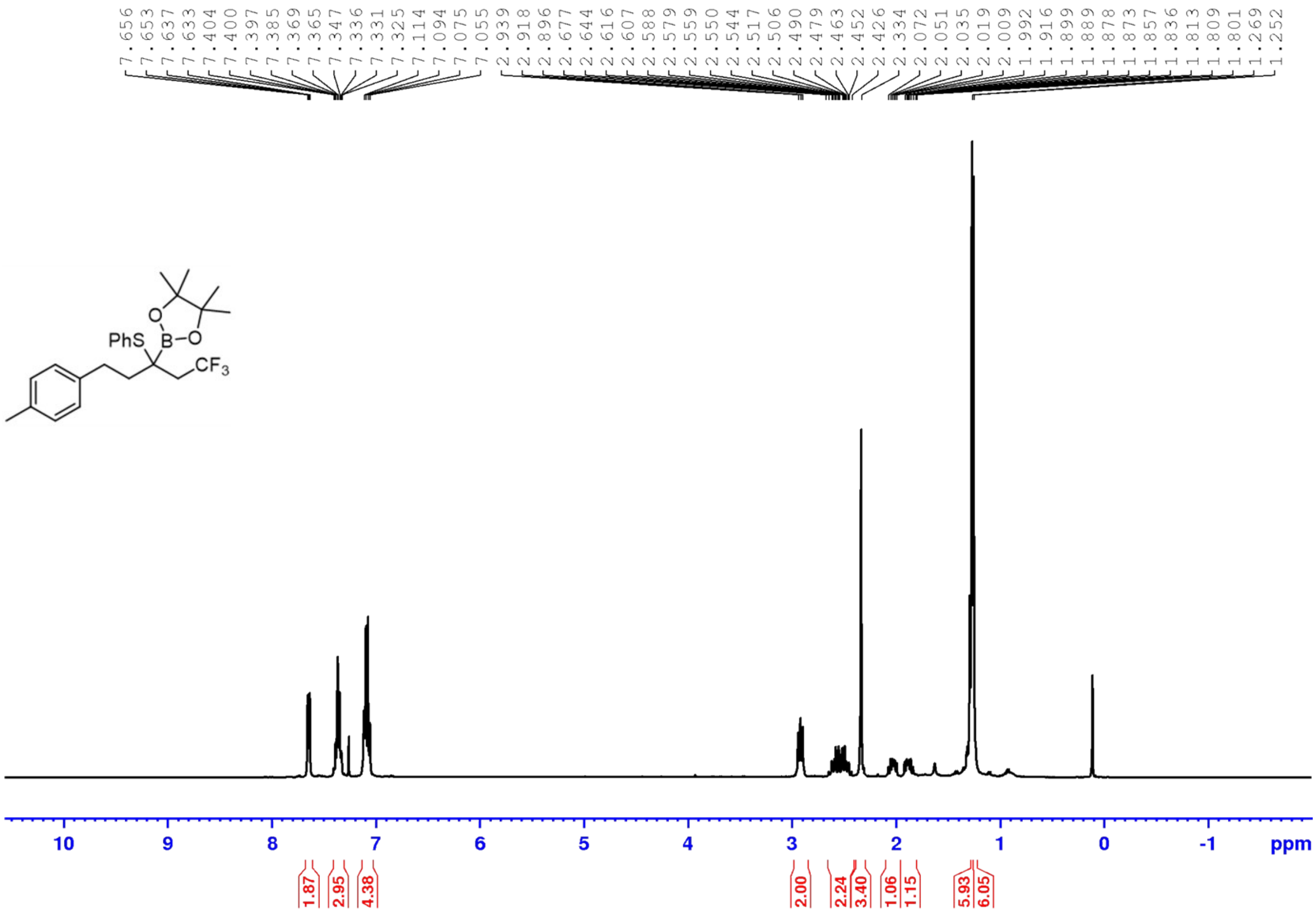

^{13}C NMR (125 MHz, Chloroform-*d*)

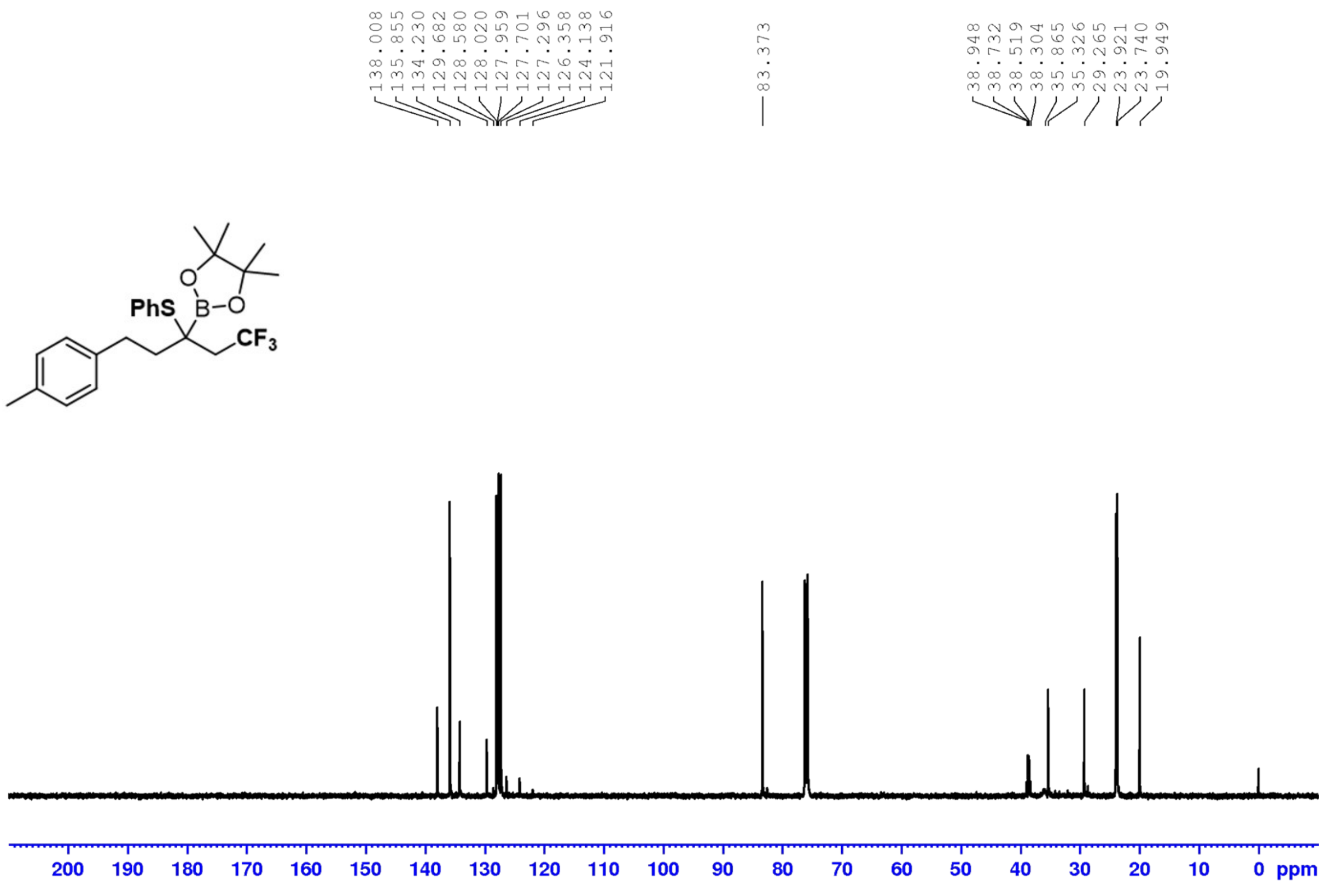

^{11}B NMR (160 MHz, Chloroform-*d*)

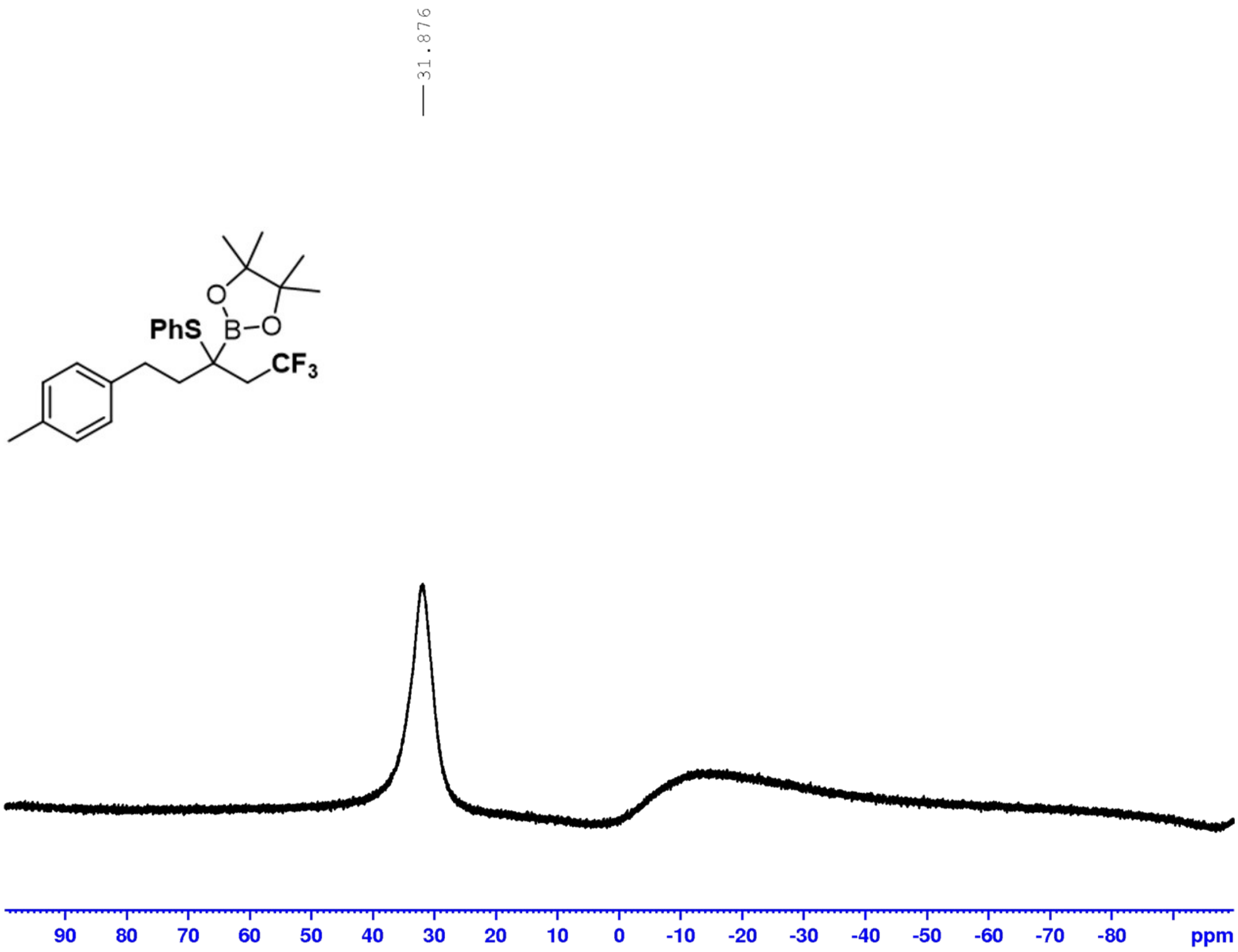

^{19}F NMR (376 MHz, Chloroform-*d*)

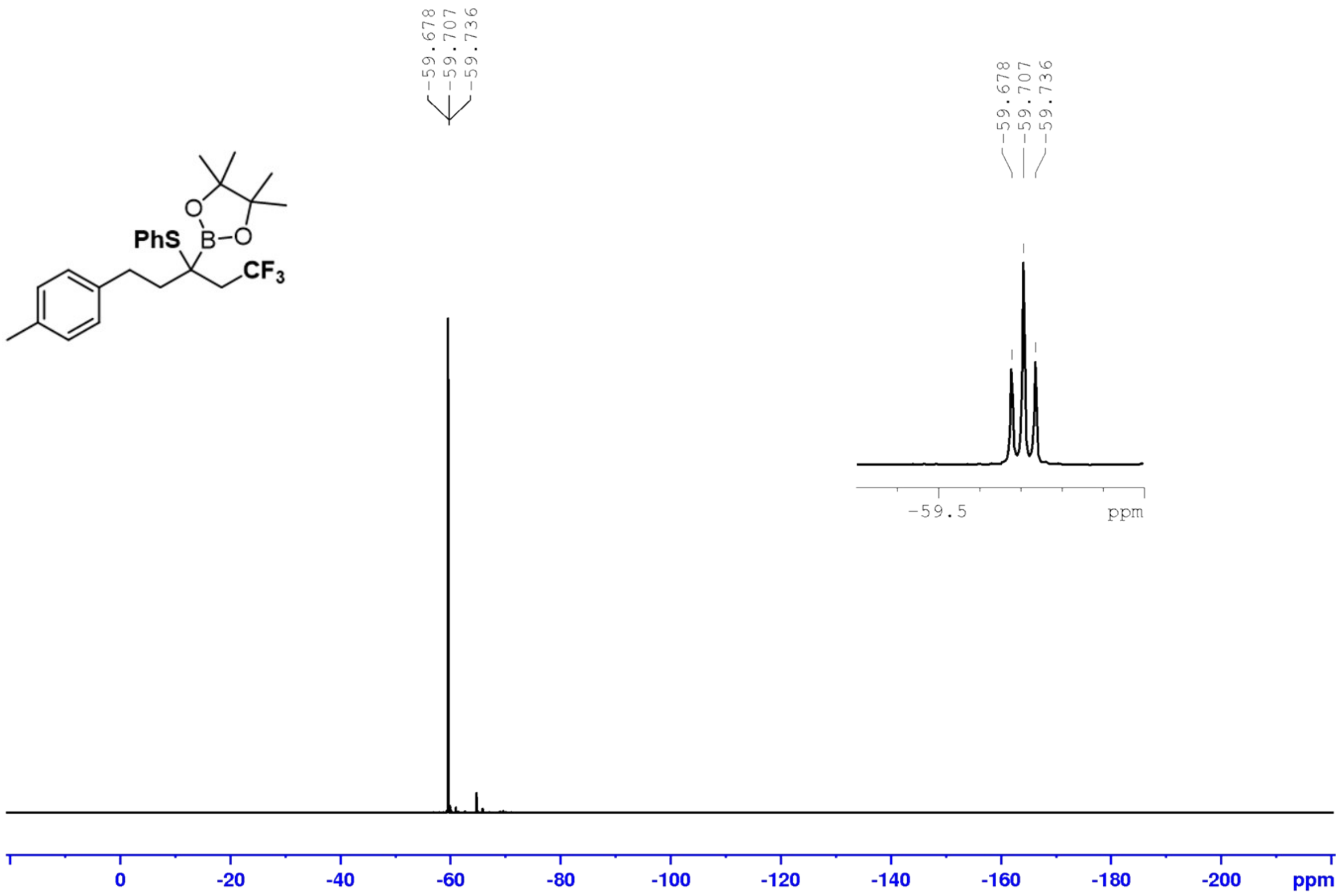

4,4,5,5-tetramethyl-2-(1,1,1-trifluoro-5-(4-fluorophenyl)-3-(phenylthio)pentan-3-yl)-1,3,2-dioxaborolane (3fa)

^{1}H NMR (400 MHz, Chloroform-*d*)

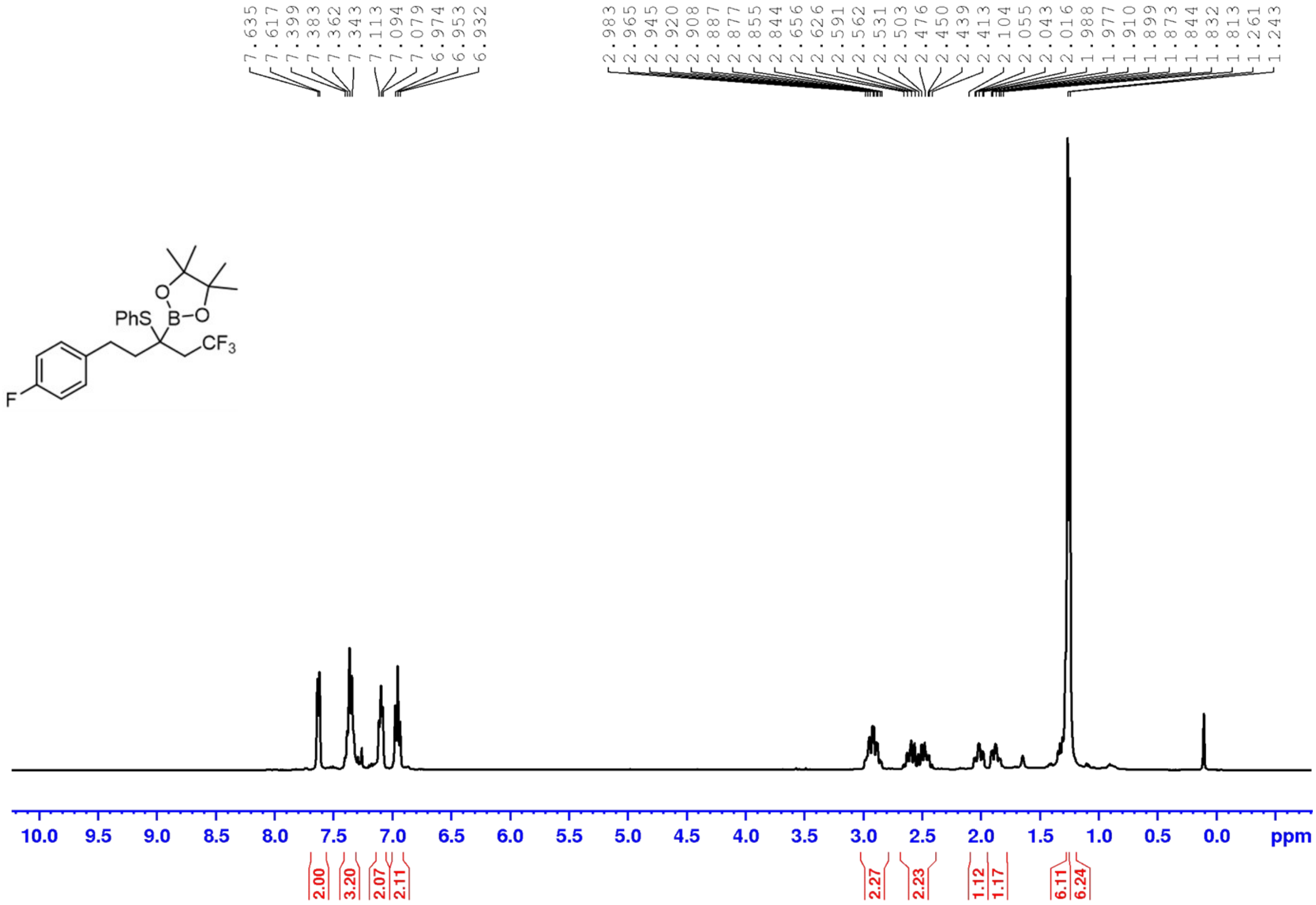

^{13}C NMR (100 MHz, Chloroform-*d*)

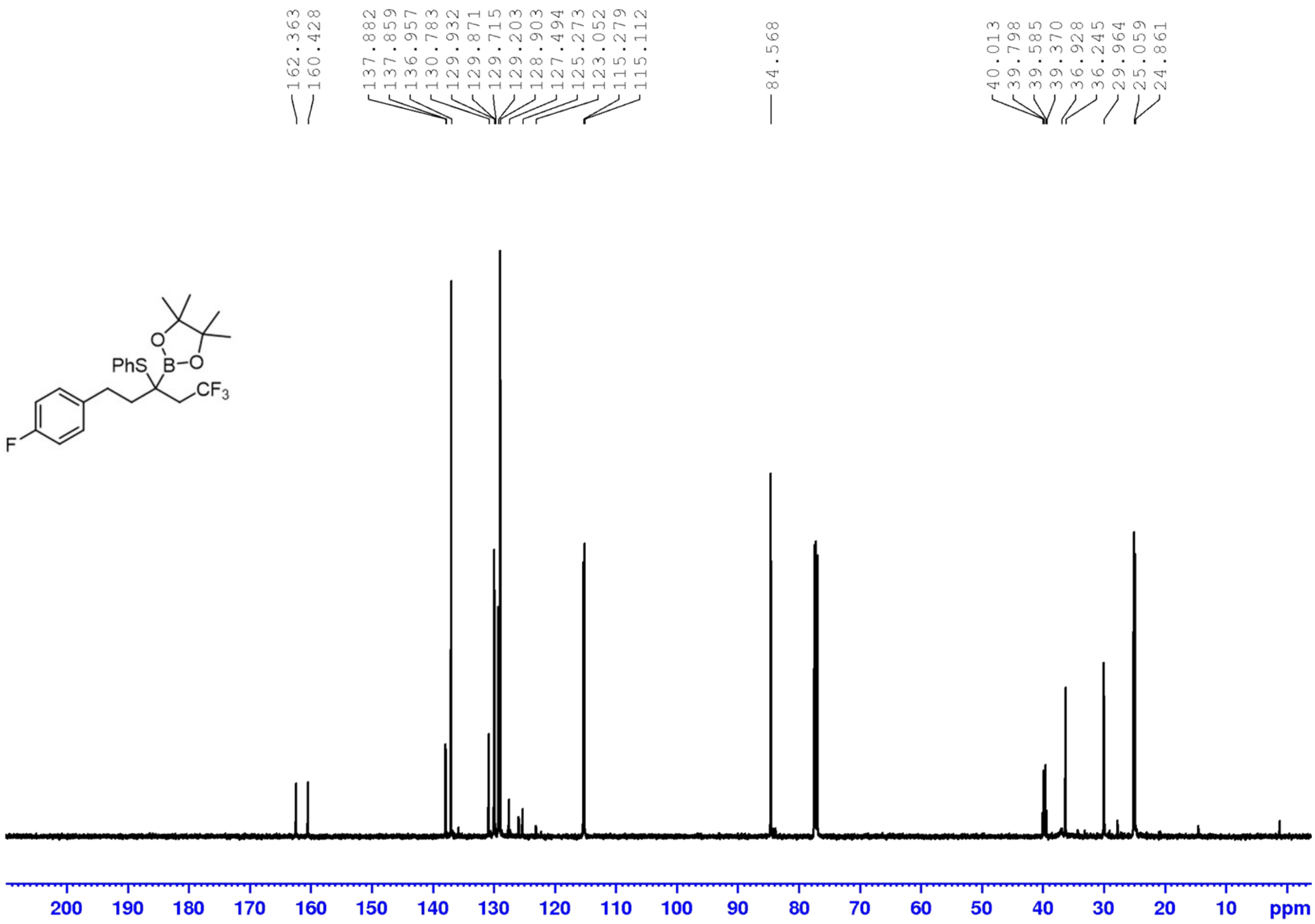

^{11}B NMR (128 MHz, Chloroform-*d*)

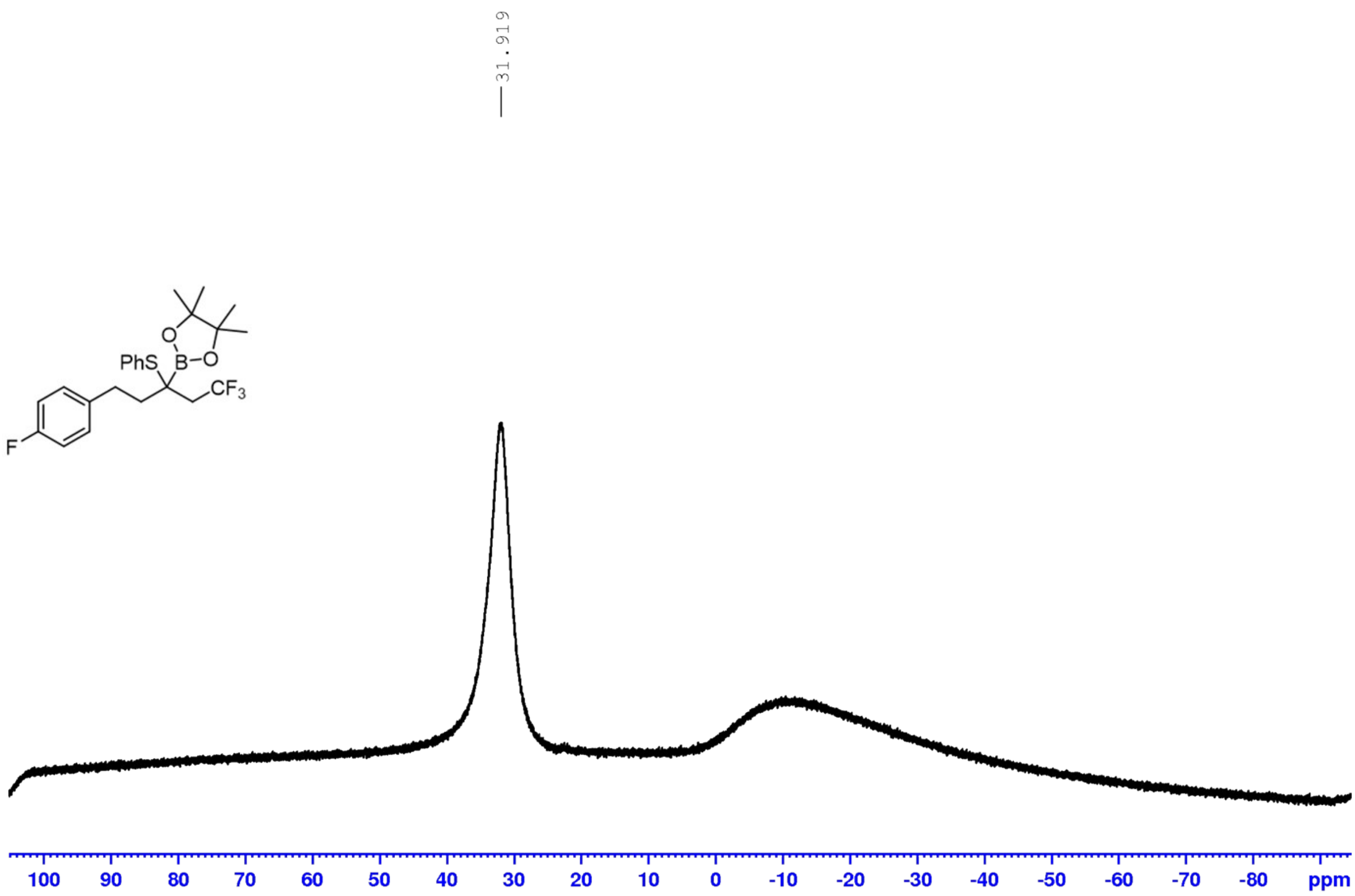

^{19}F NMR (376 MHz, Chloroform-*d*)

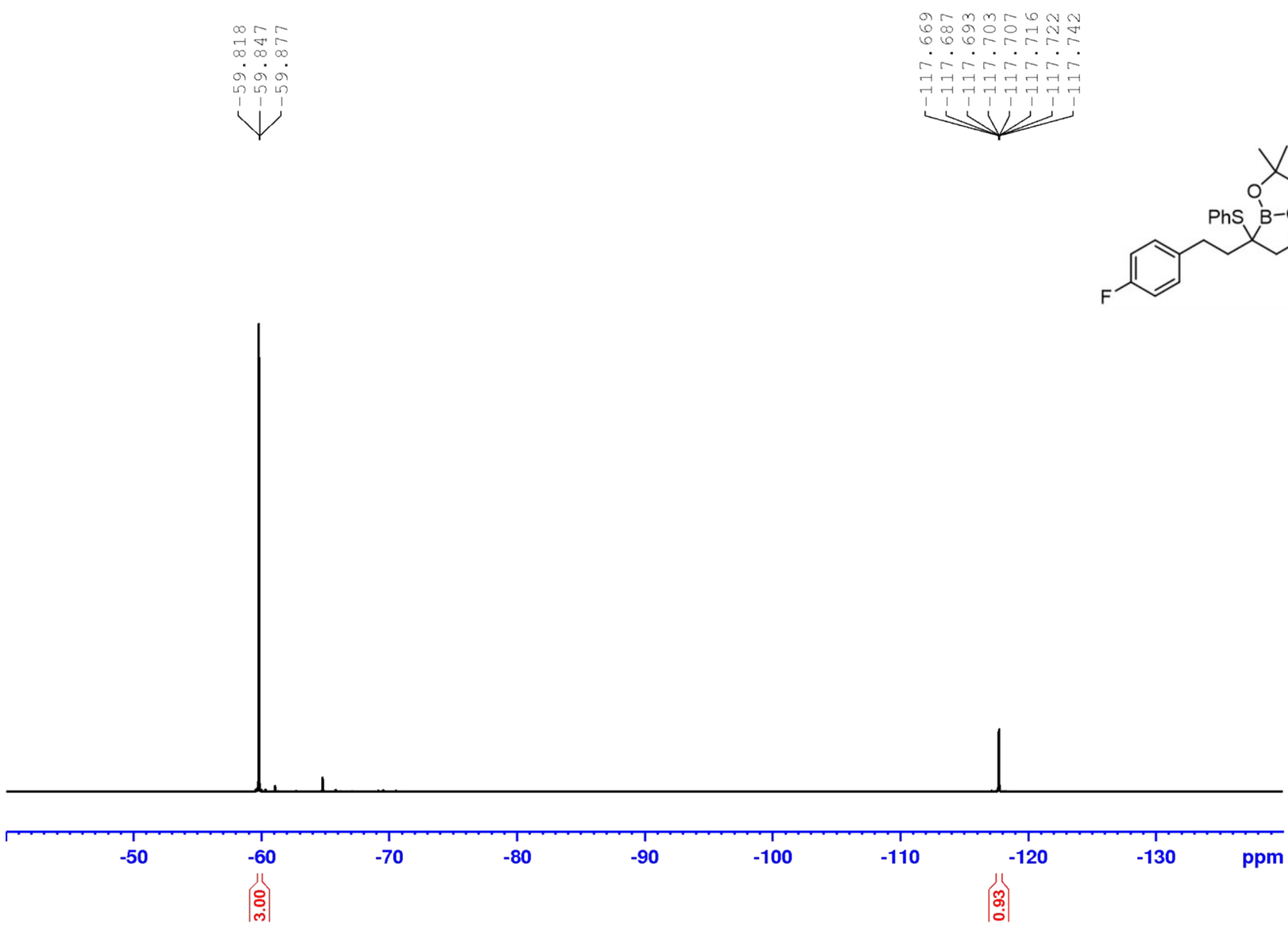

2-(5-(4-chlorophenyl)-1,1,1-trifluoro-3-(phenylthio)pentan-3-yl)-4,4,5,5-tetramethyl-1,3,2-dioxaborolane (3ga)

^{1}H NMR (400 MHz, Chloroform-*d*)

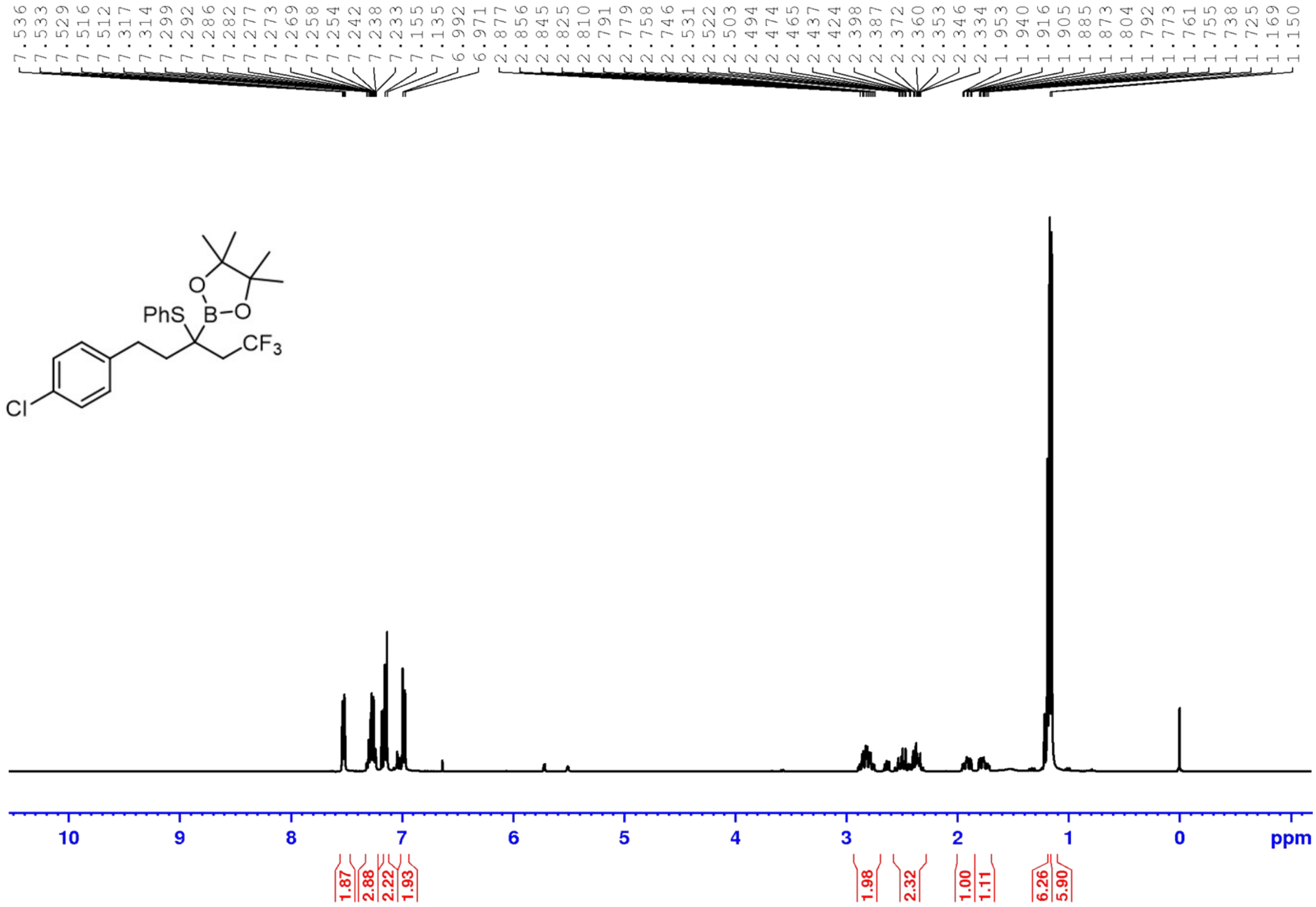

^{13}C NMR (125 MHz, Chloroform-*d*)

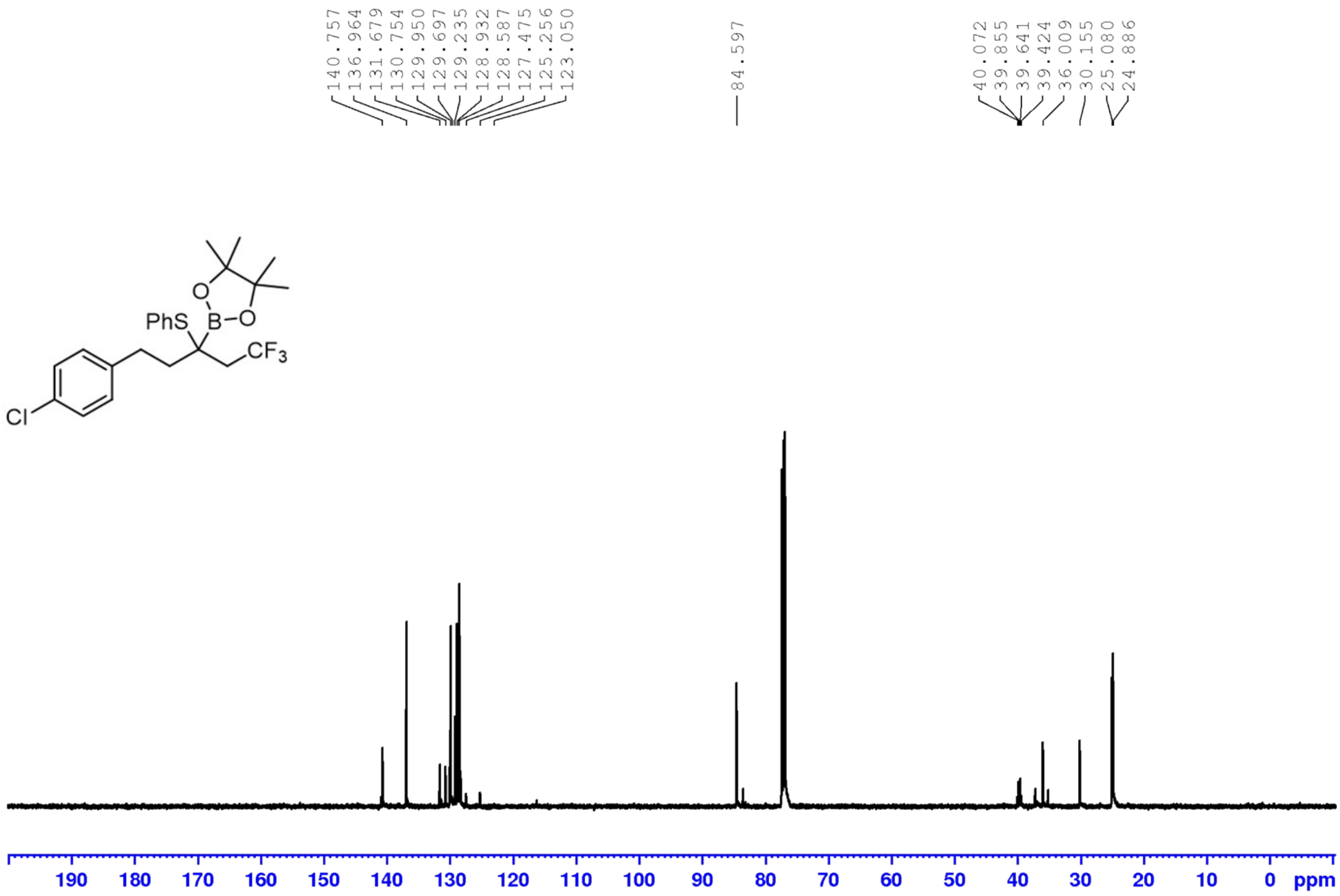

^{11}B NMR (128 MHz, Chloroform-*d*)

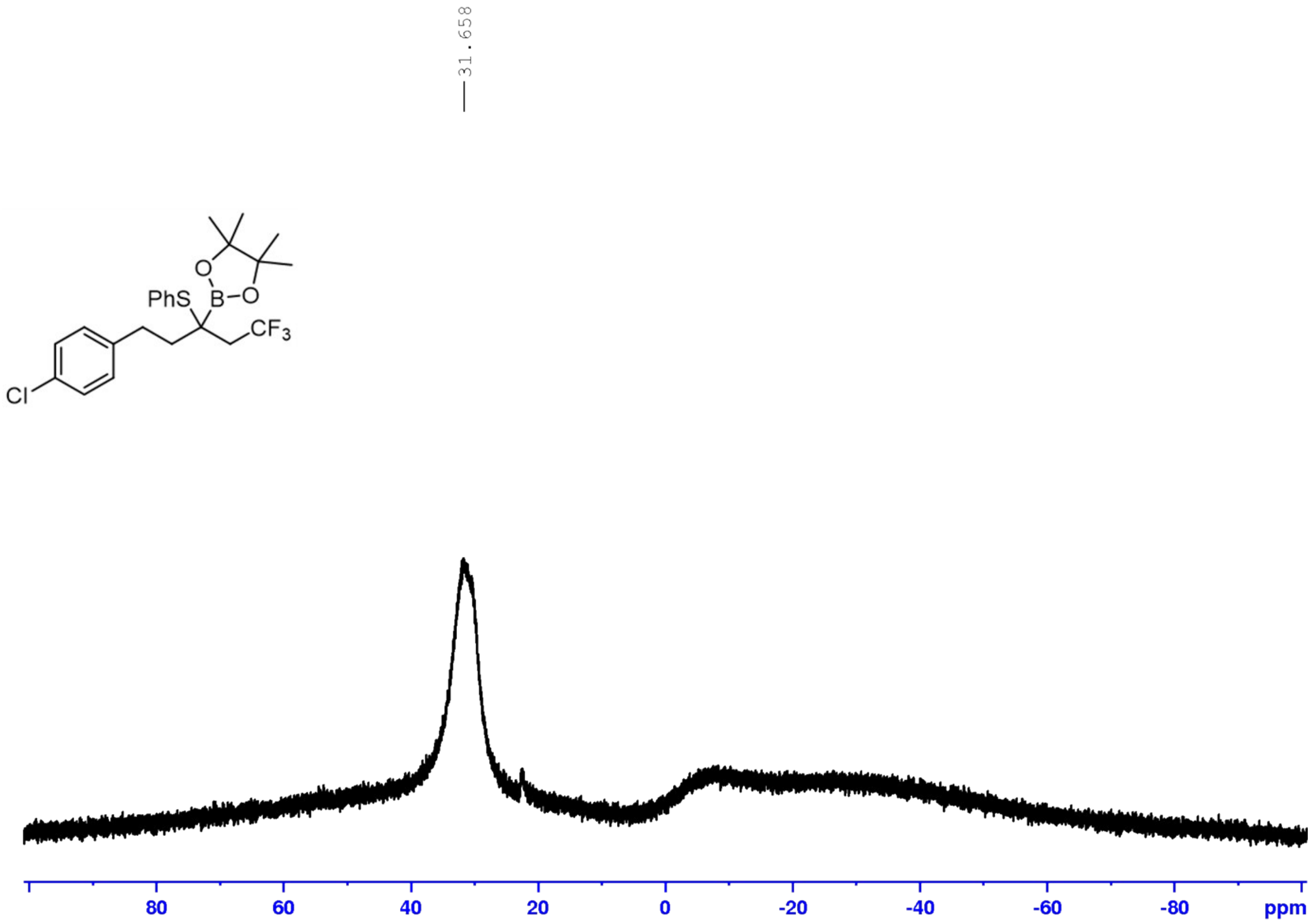

^{19}F NMR (376 MHz, Chloroform-*d*)

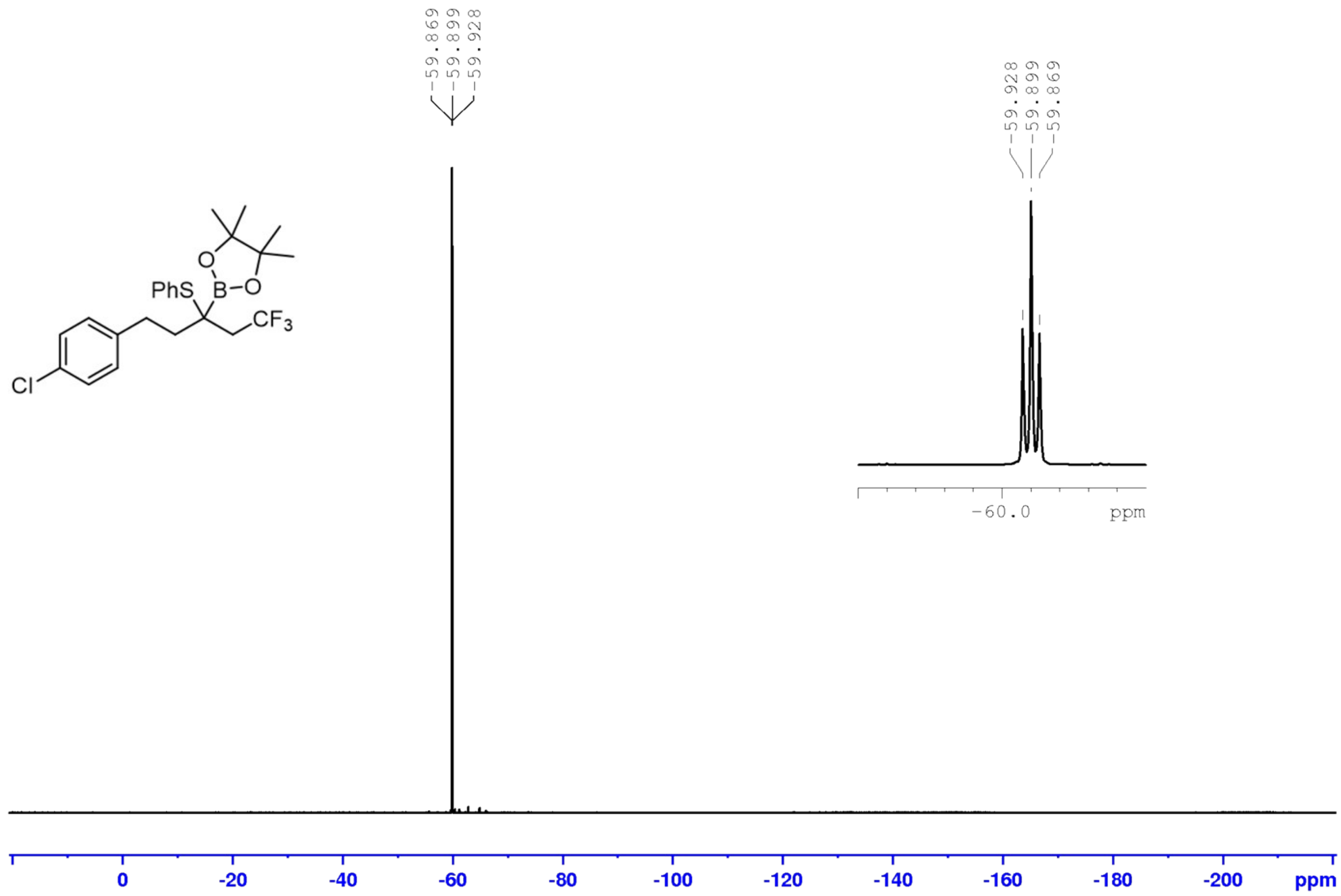

2-(5-(4-bromophenyl)-1,1,1-trifluoro-3-(phenylthio)pentan-3-yl)-4,4,5,5-tetramethyl-1,3,2-dioxaborolane (3ha)

^{1}H NMR (400 MHz, Chloroform-*d*)

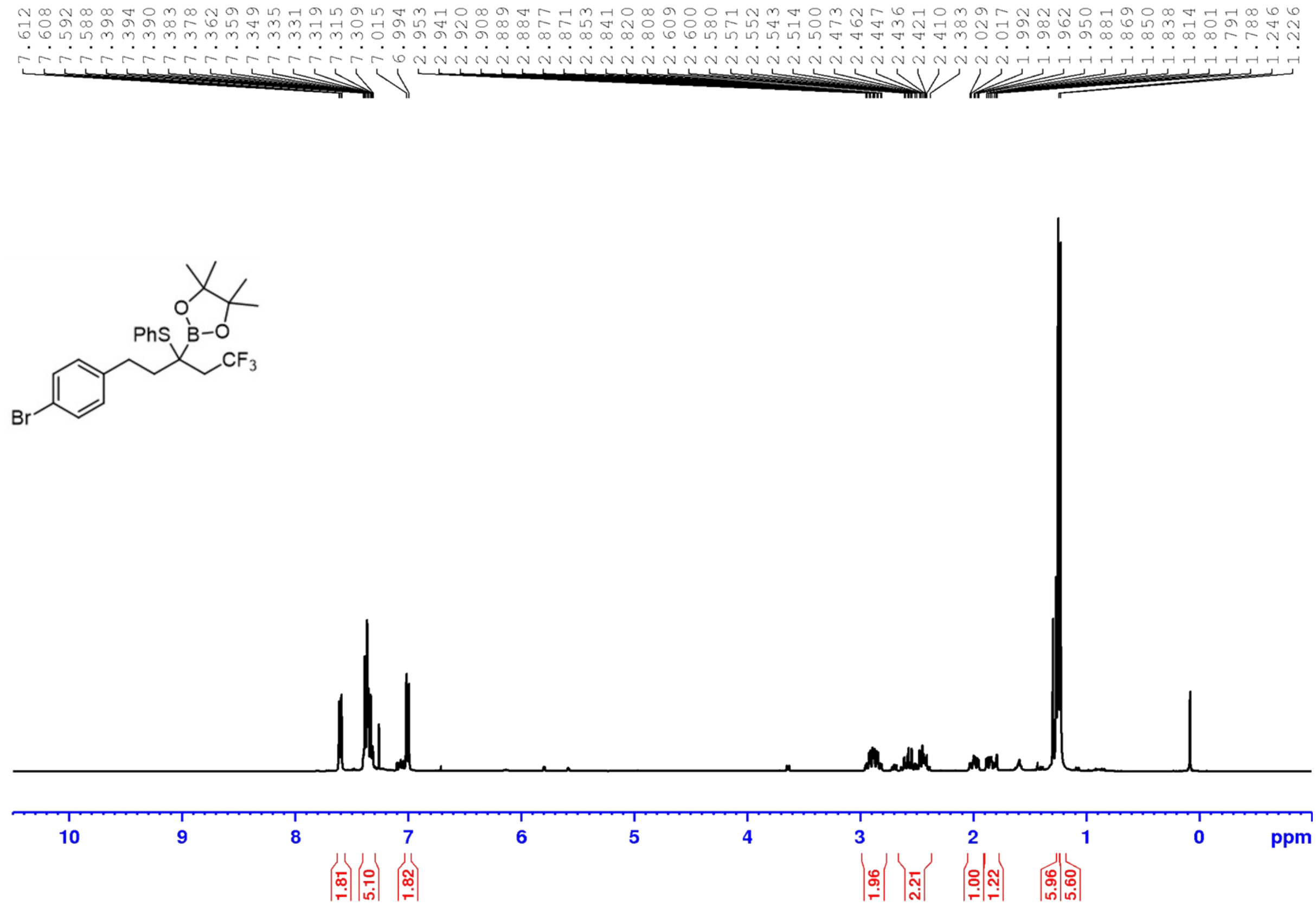

^{13}C NMR (100 MHz, Chloroform-*d*)

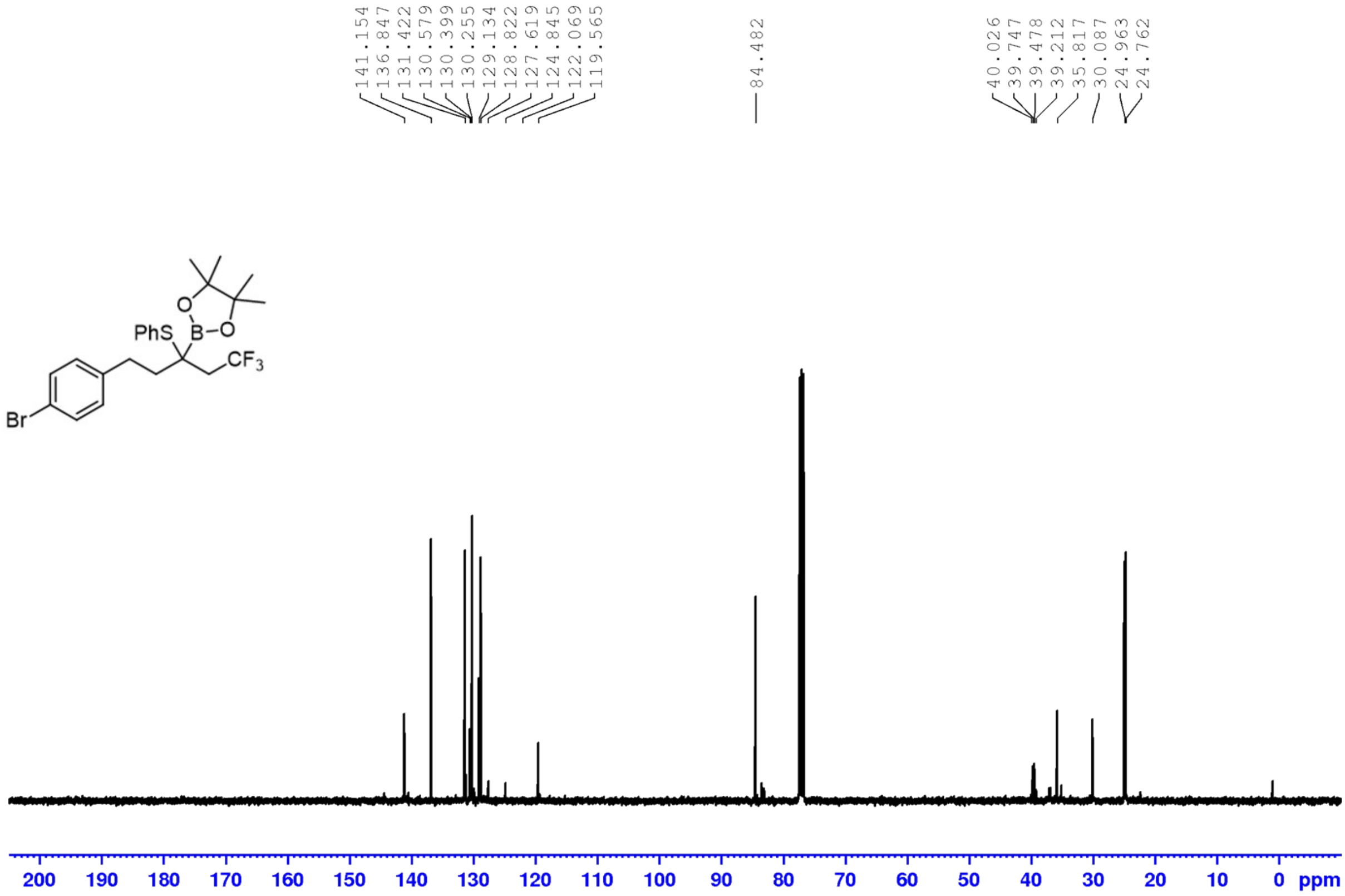

^{11}B NMR (128 MHz, Chloroform-*d*)

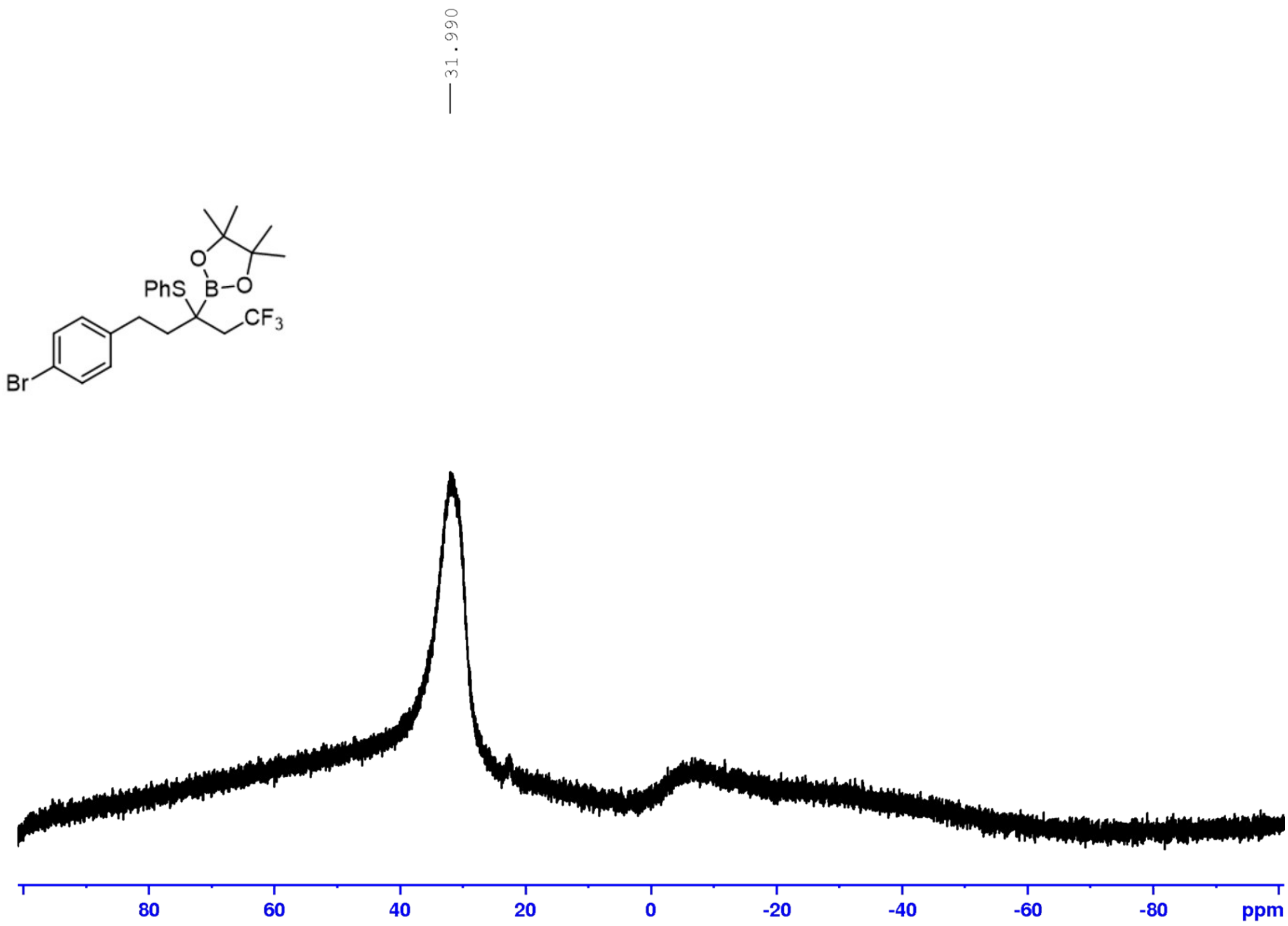

^{19}F NMR (376 MHz, Chloroform-*d*)

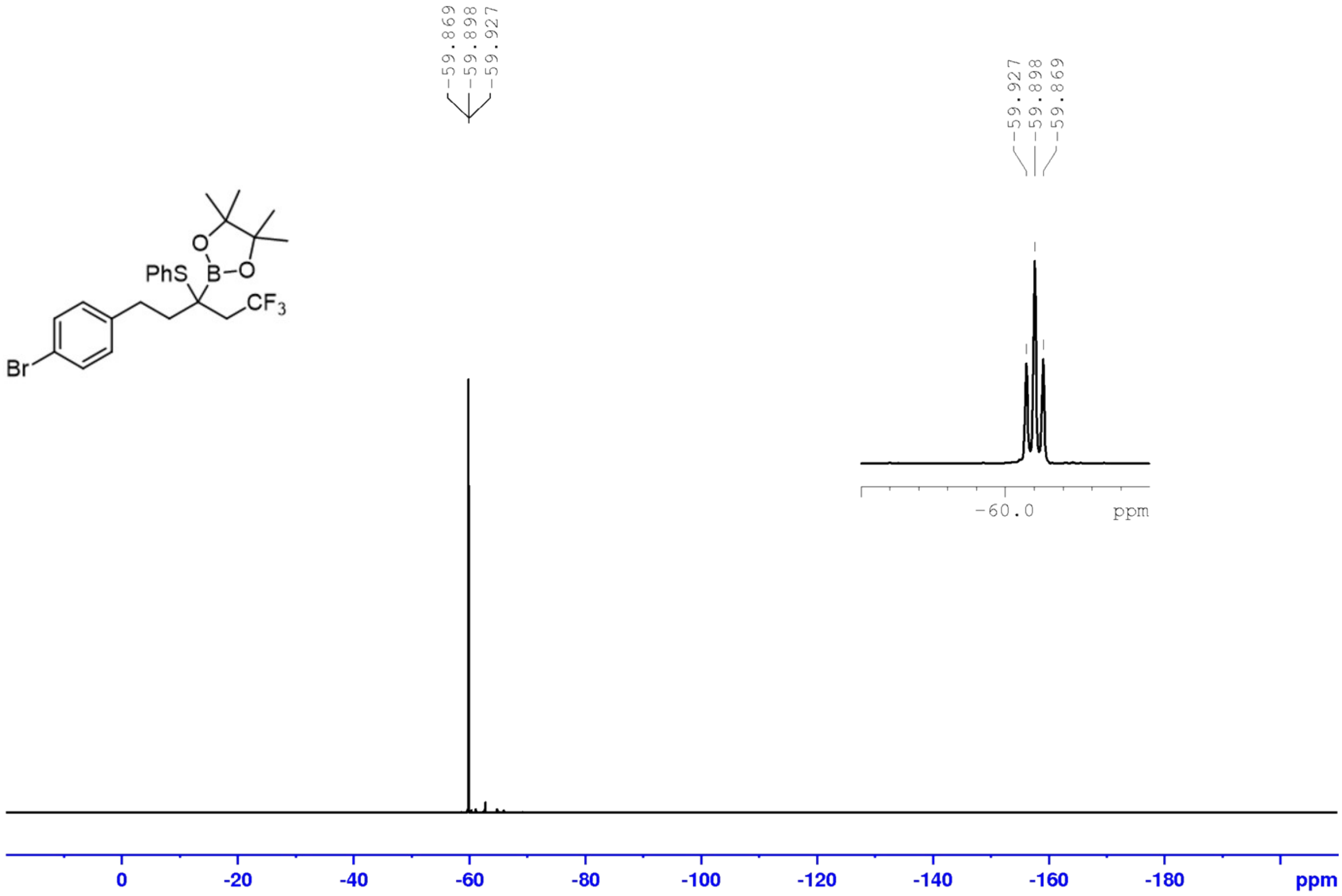

4,4,5,5-tetramethyl-2-(1,1,1-trifluoro-3-(phenylthio)-5-(4-(trifluoromethoxy)phenyl)pentan-3-yl)-1,3,2-dioxaborolane (3ia)

^{1}H NMR (400 MHz, Chloroform-*d*)

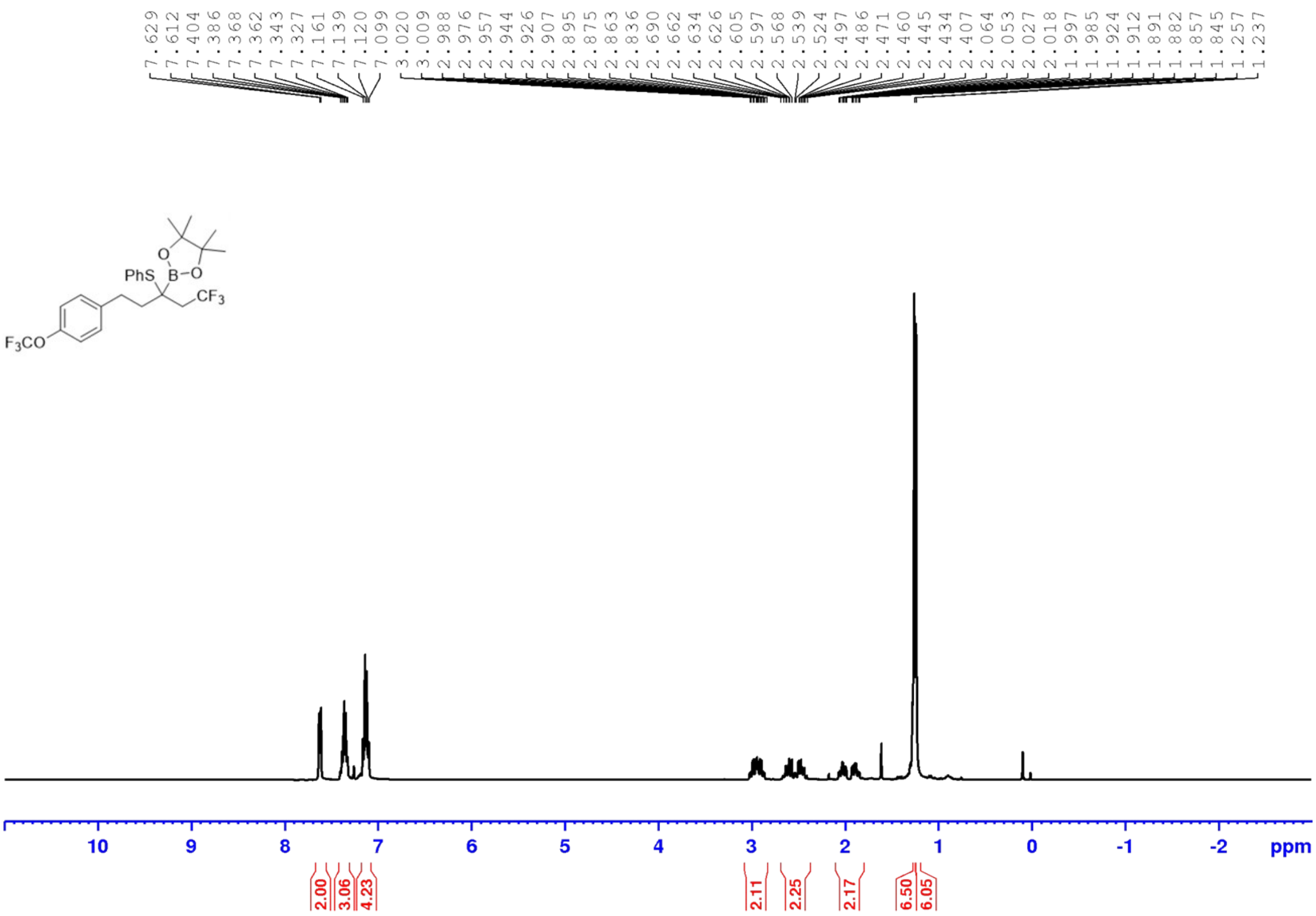

^{13}C NMR (100 MHz, Chloroform-*d*)

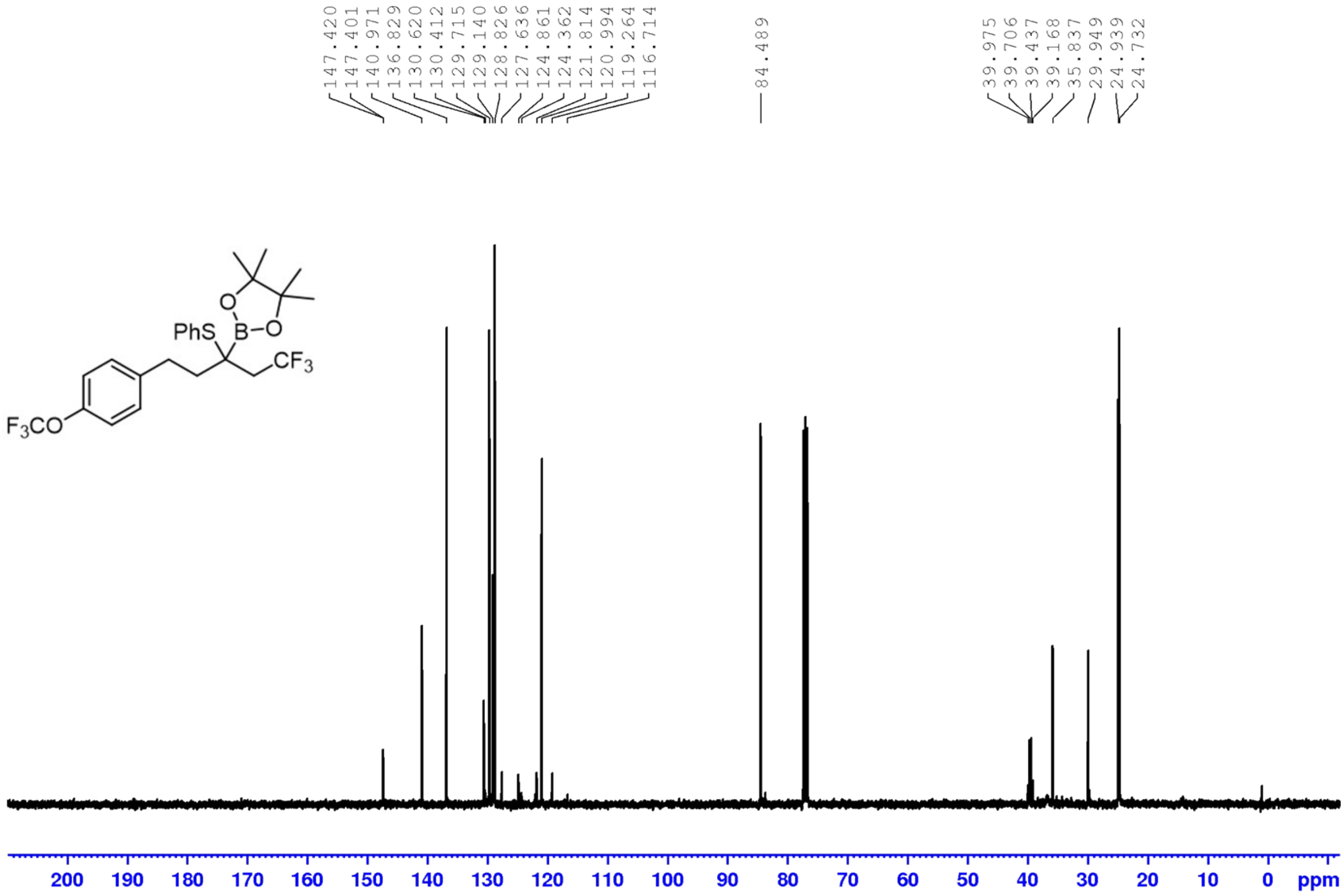

^{11}B NMR (128 MHz, Chloroform-*d*)

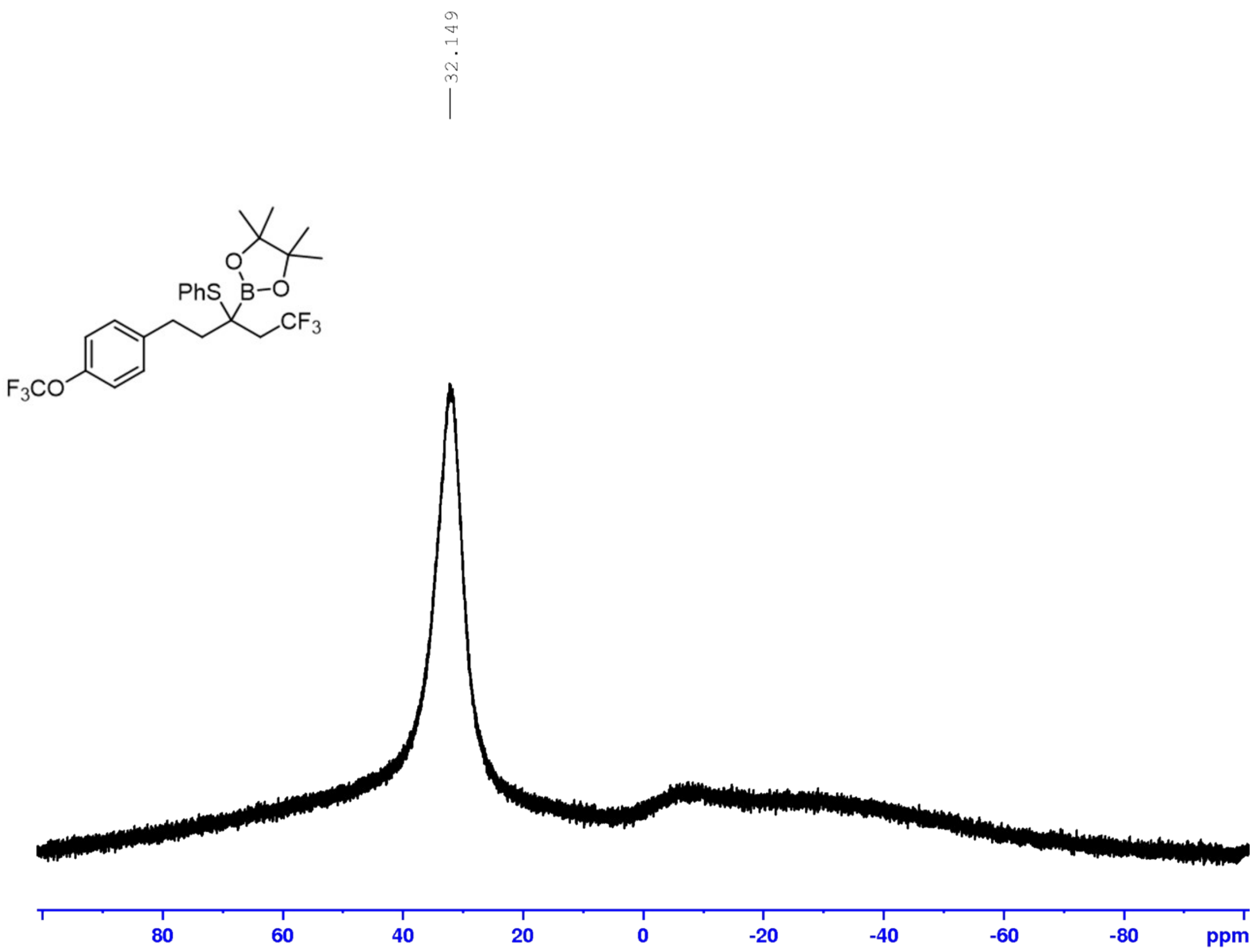

^{19}F NMR (376 MHz, Chloroform-*d*)

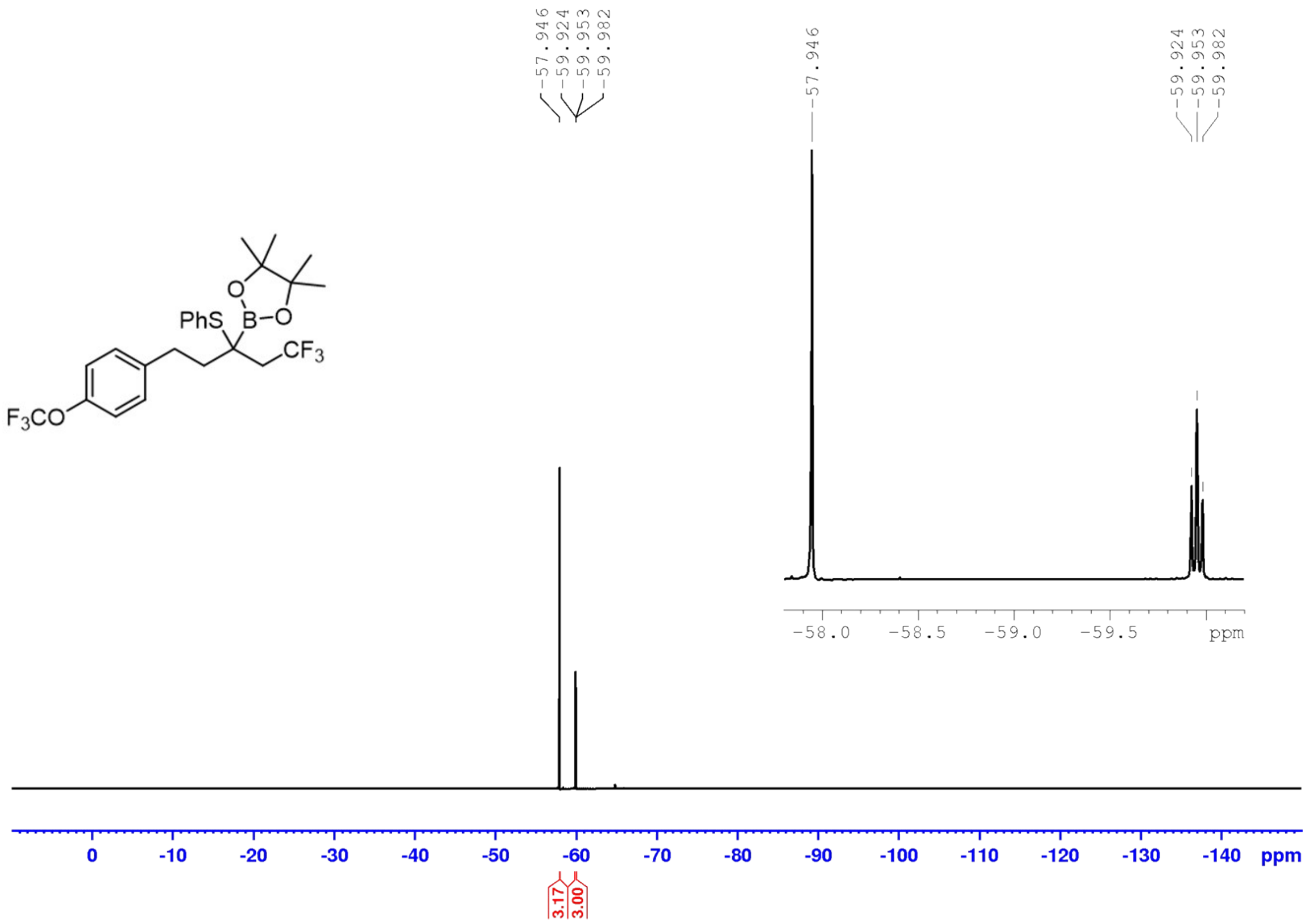

4,4,5,5-tetramethyl-2-(1,1,1-trifluoro-3-(phenylthio)-5-(3-(trifluoromethyl)phenyl)pentan-3-yl)-1,3,2-dioxaborolane (3ja)

^{1}H NMR (400 MHz, Chloroform-*d*)

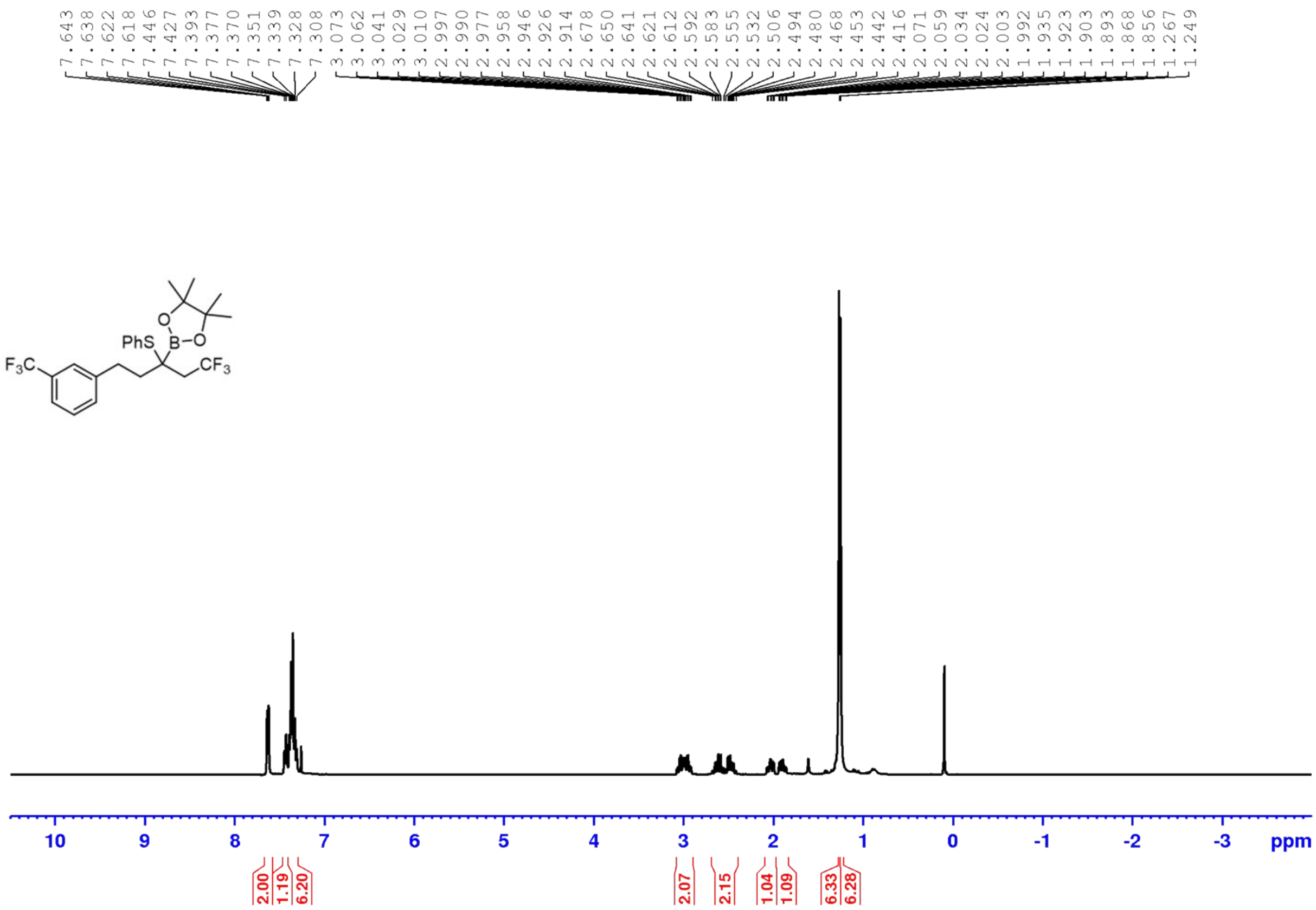

^{13}C NMR (125 MHz, Chloroform-*d*)

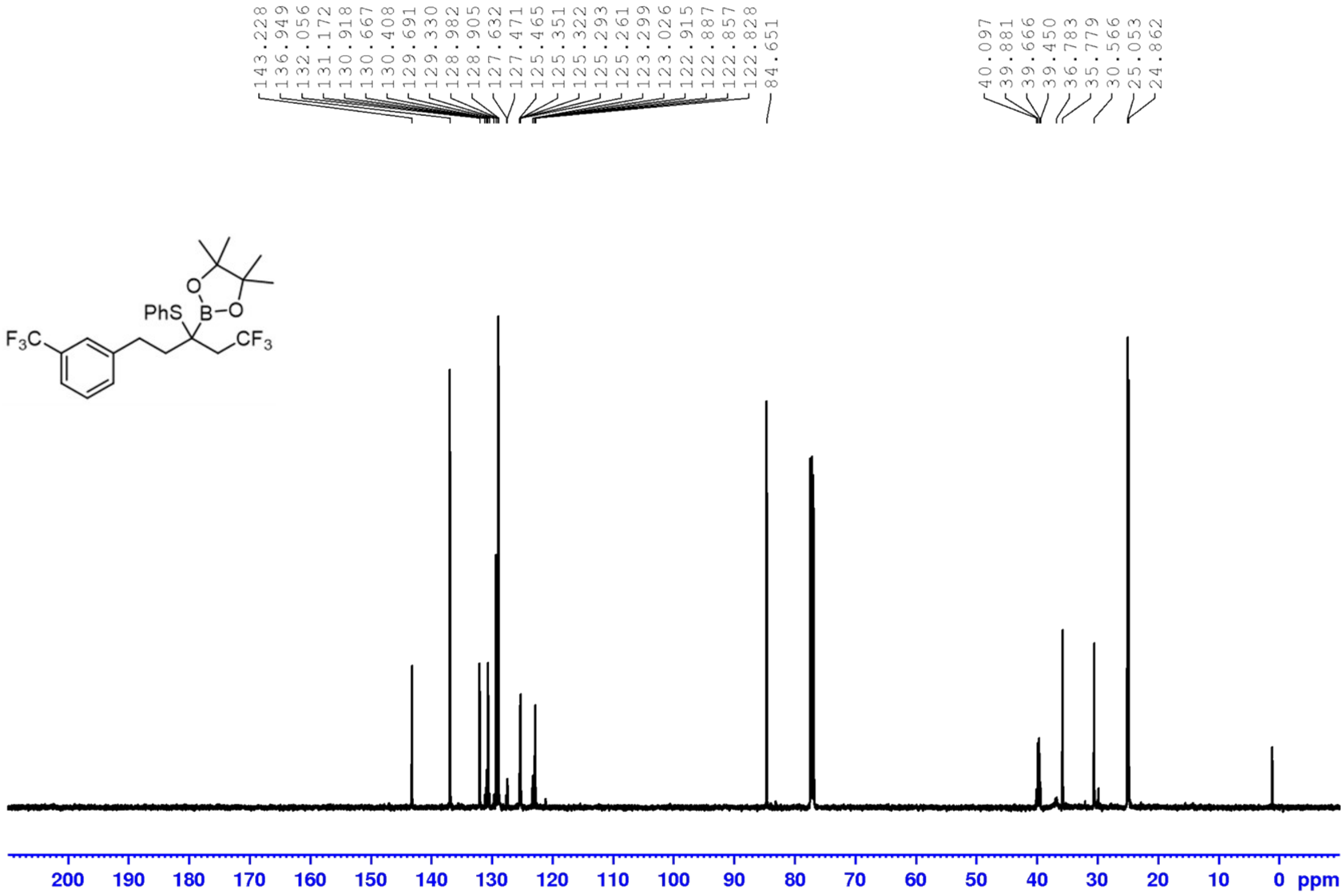

^{11}B NMR (160 MHz, Chloroform-*d*)

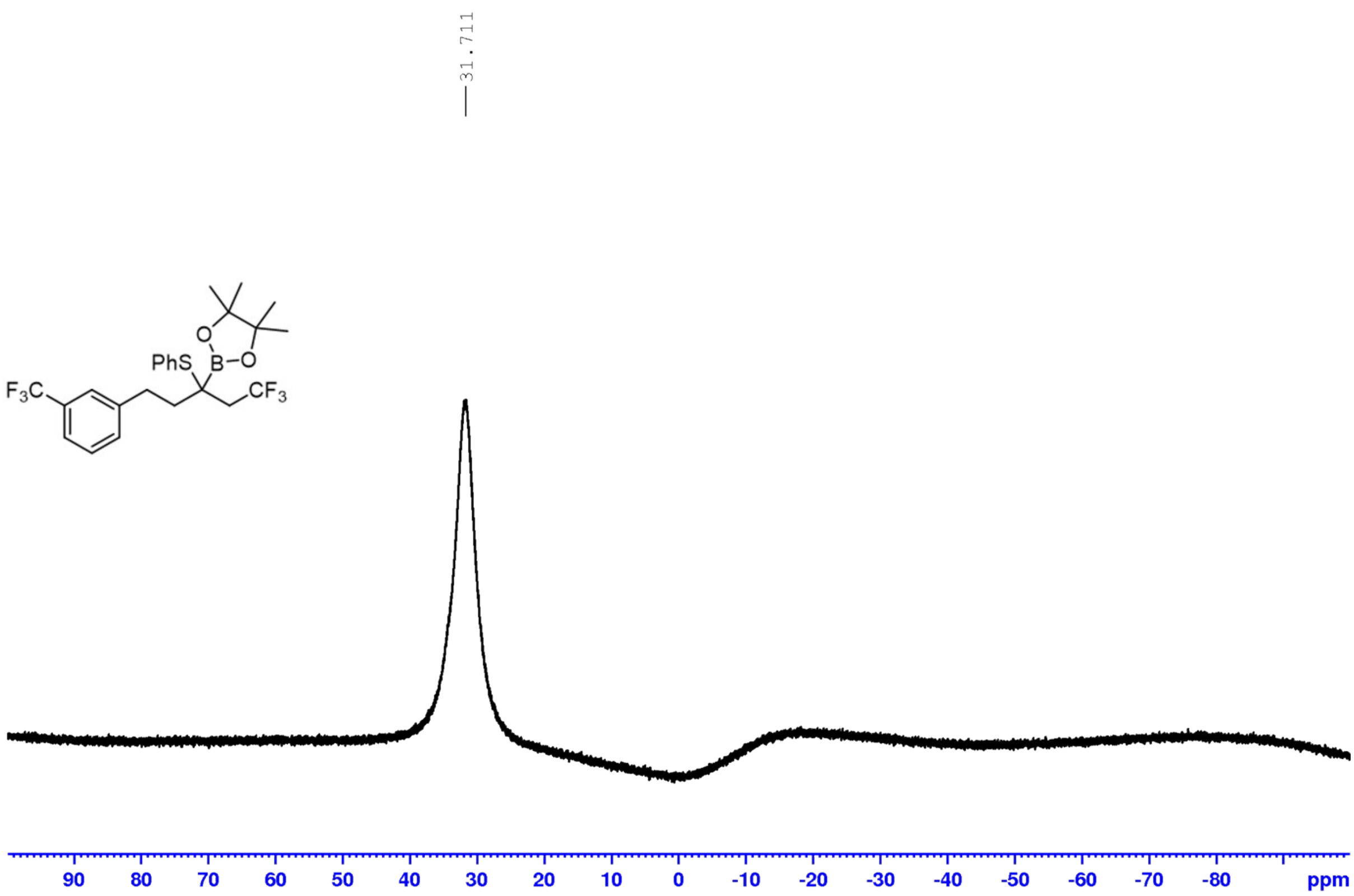

^{19}F NMR (376 MHz, Chloroform-*d*)

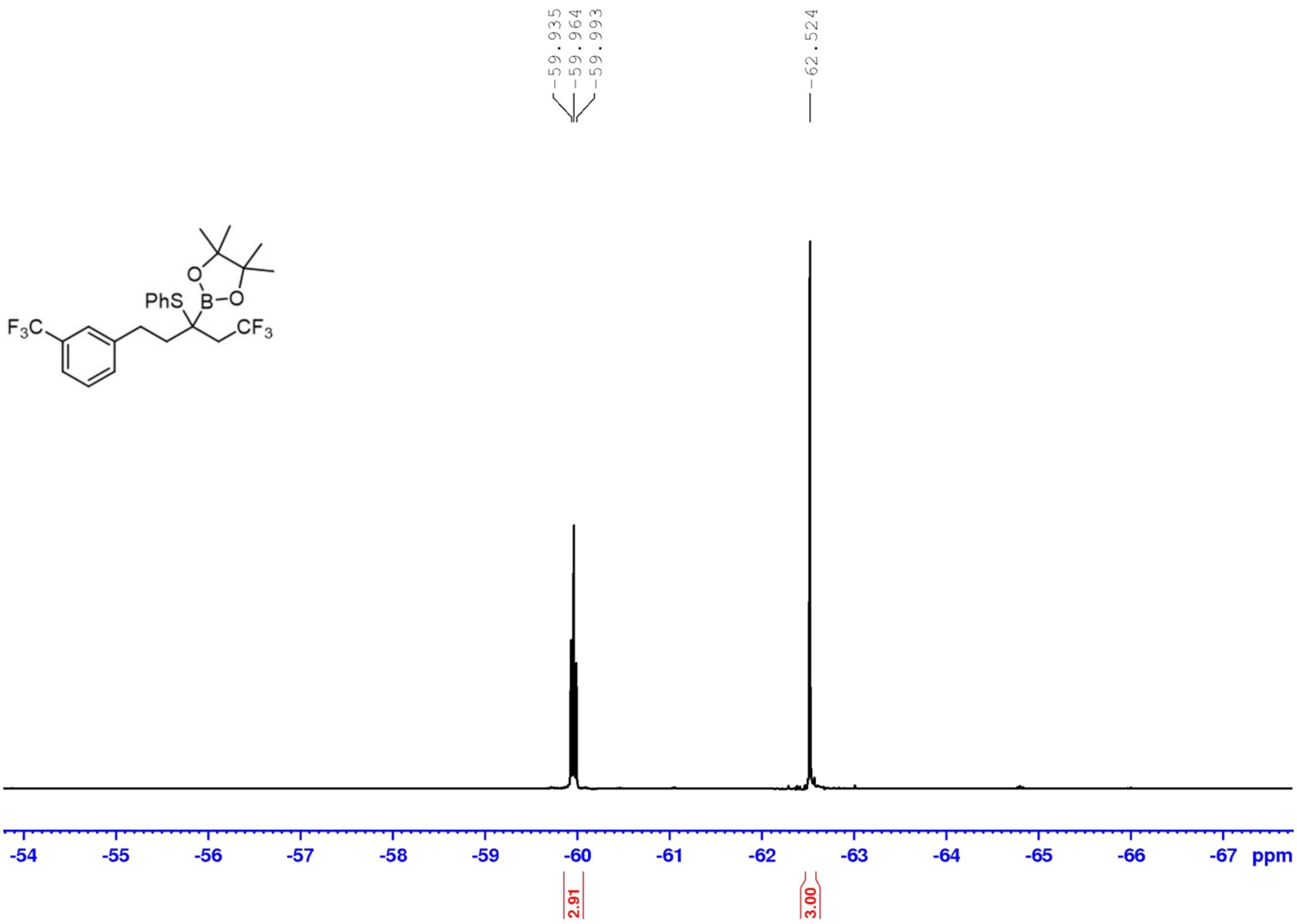

2-(5-(2,3-dimethylphenyl)-1,1,1-trifluoro-3-(phenylthio)pentan-3-yl)-4,4,5,5-tetramethyl-1,3,2-dioxaborolane (3ka)

^{1}H NMR (400 MHz, Chloroform-*d*)

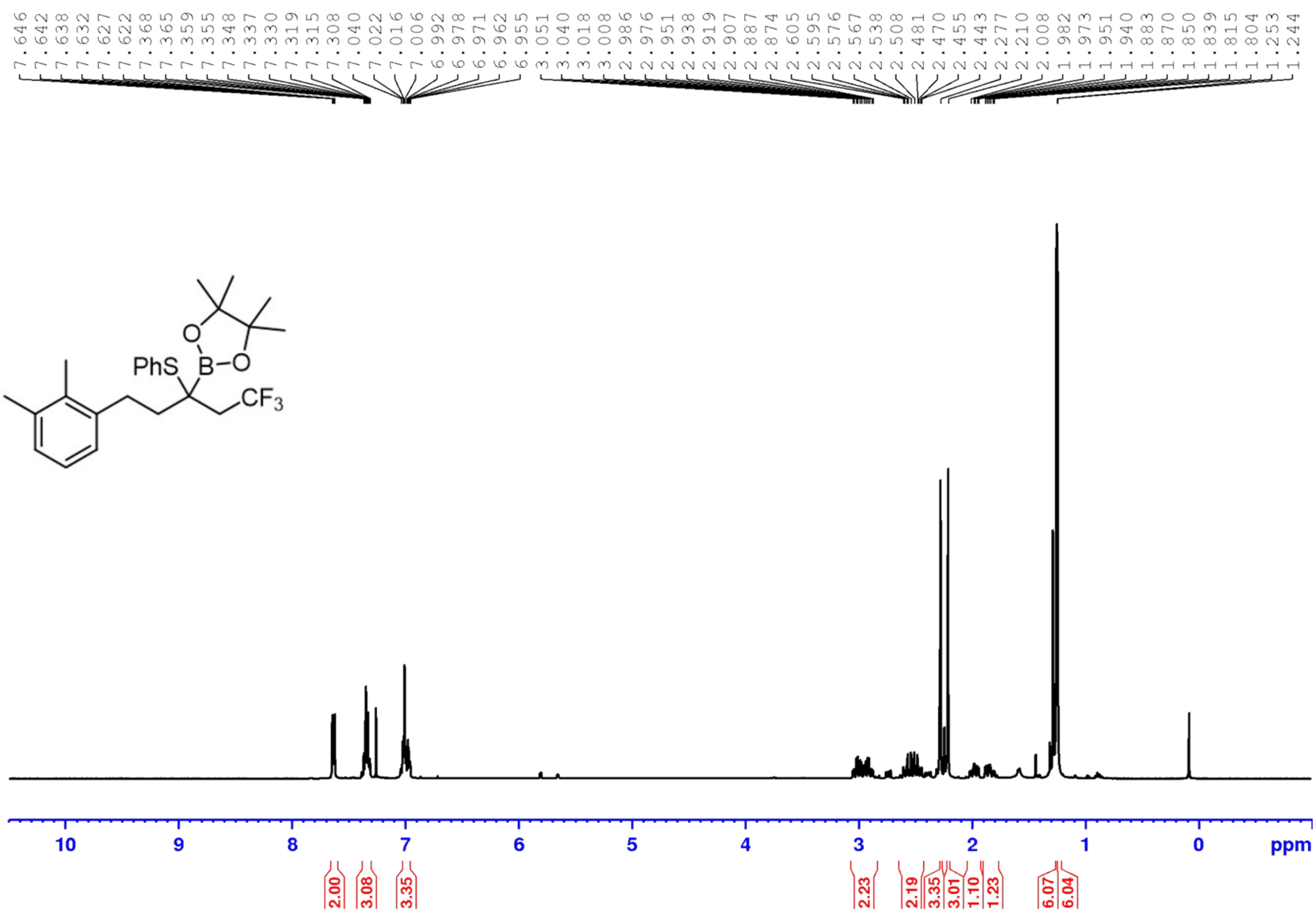

^{13}C NMR (125 MHz, Chloroform-*d*)

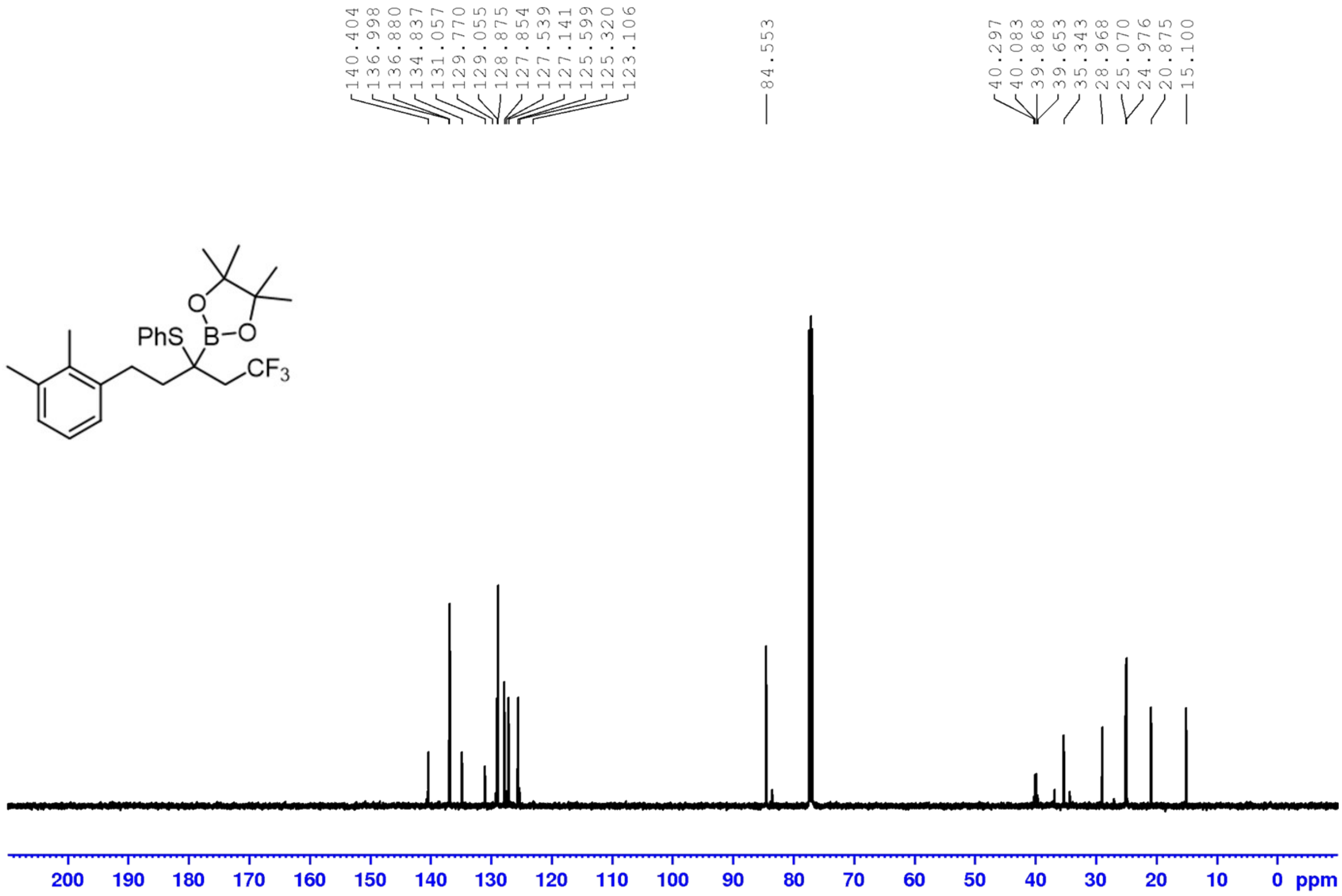

^{11}B NMR (128 MHz, Chloroform-*d*)

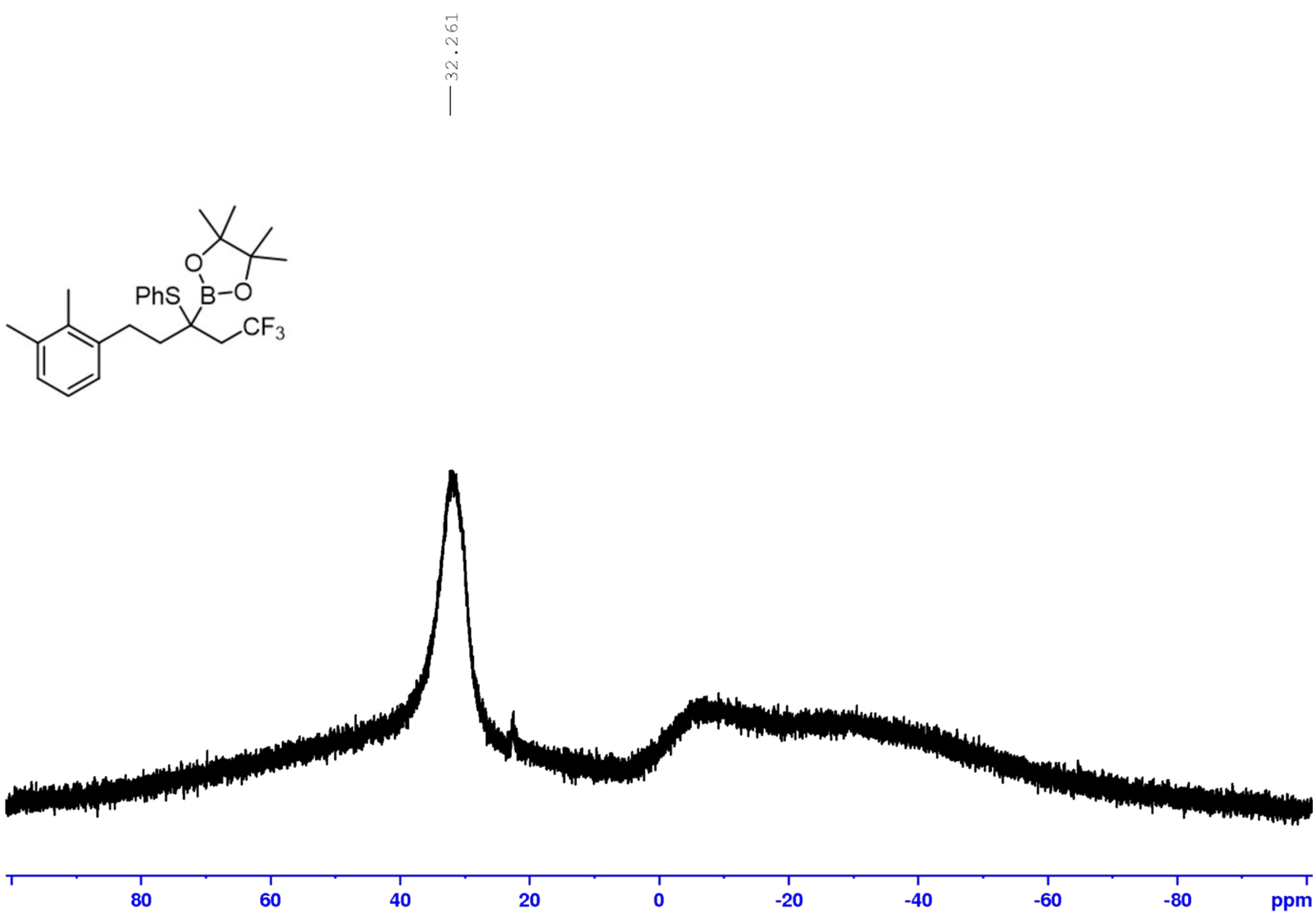

^{19}F NMR (376 MHz, Chloroform-*d*)

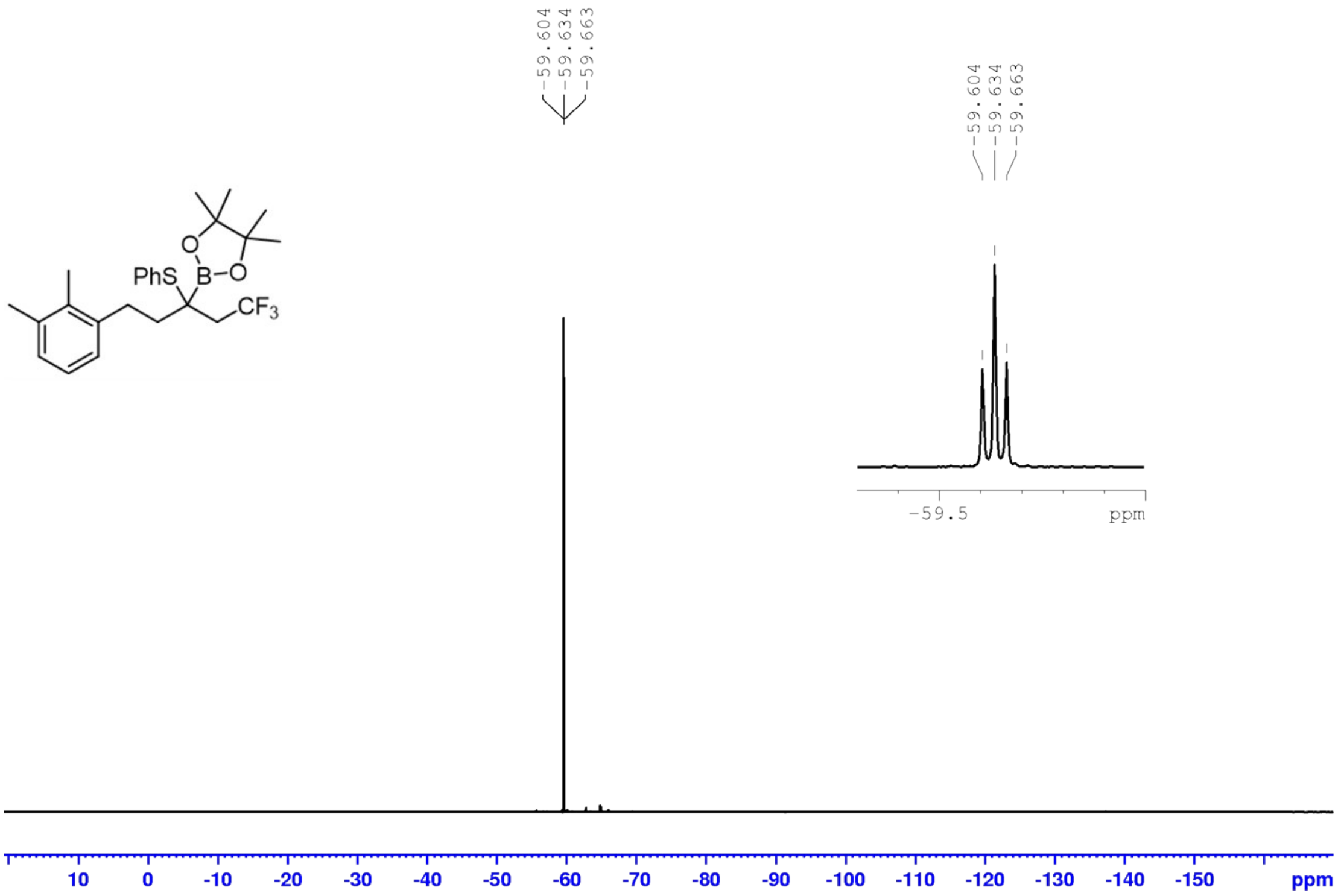

2-(5-(3,5-dimethylphenyl)-1,1,1-trifluoro-3-(phenylthio)pentan-3-yl)-4,4,5,5-tetramethyl-1,3,2-dioxaborolane (3la)

^{1}H NMR (400 MHz, Chloroform-*d*)

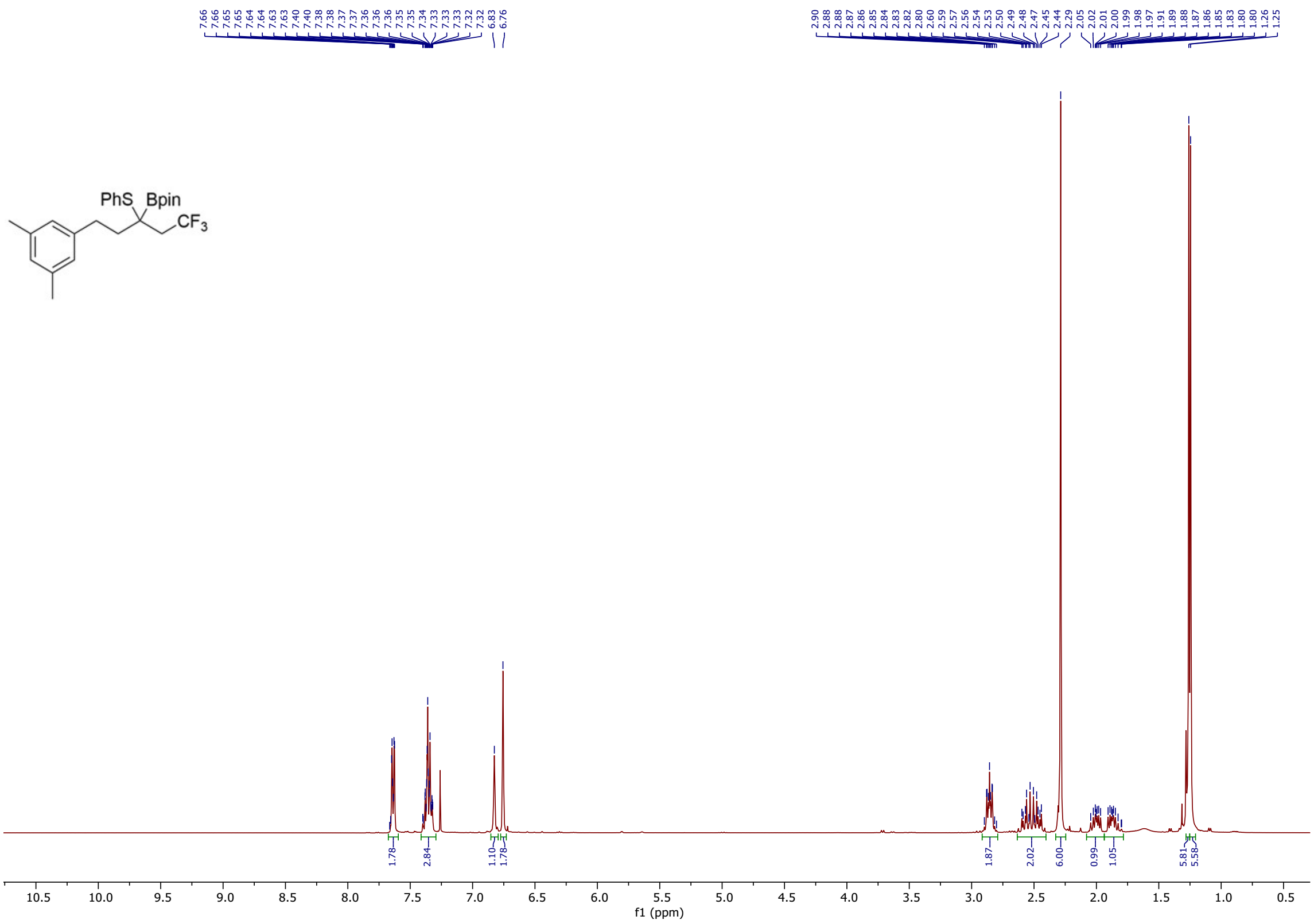

^{13}C NMR (125 MHz, Chloroform-*d*)

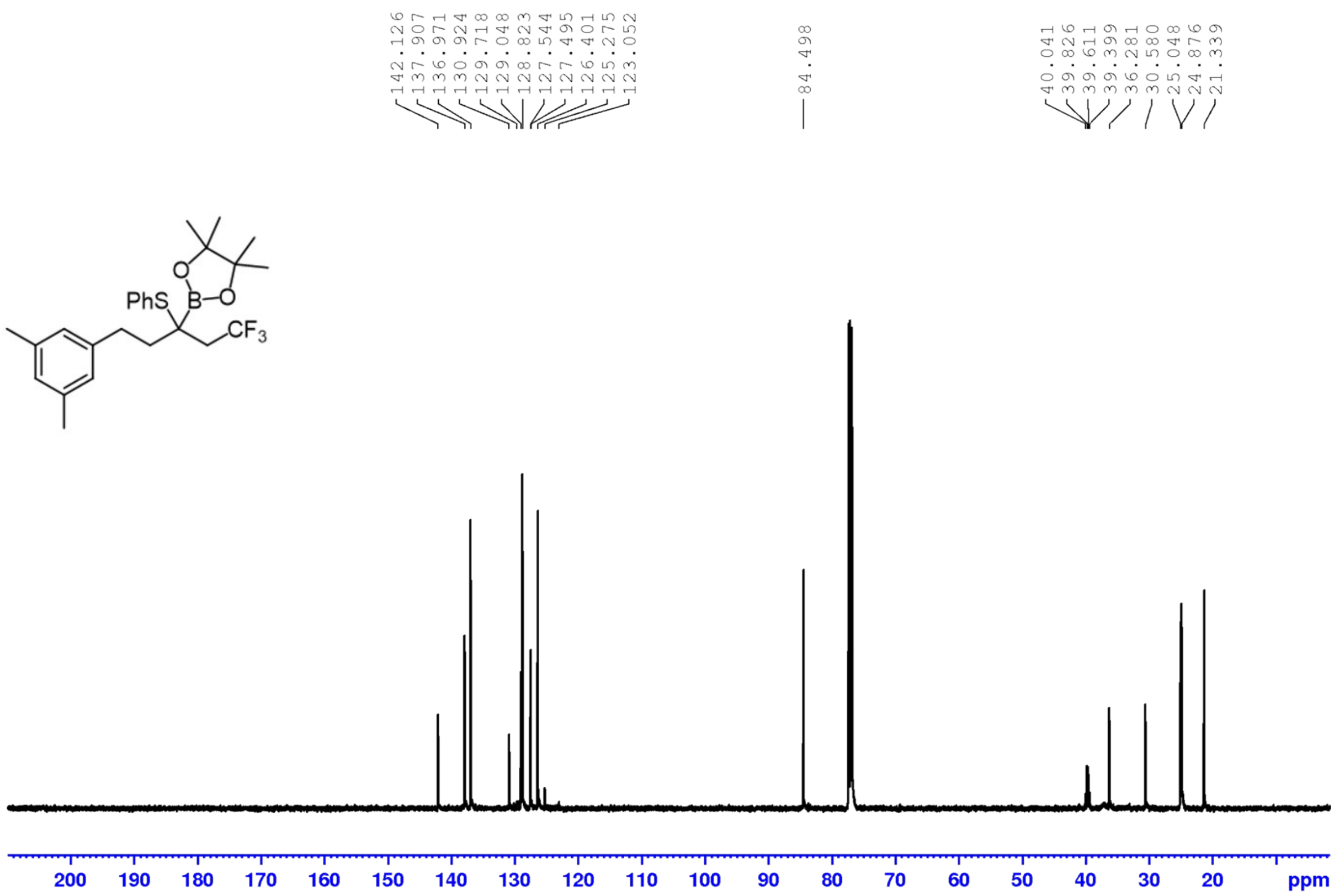

^{11}B NMR (128 MHz, Chloroform-*d*)

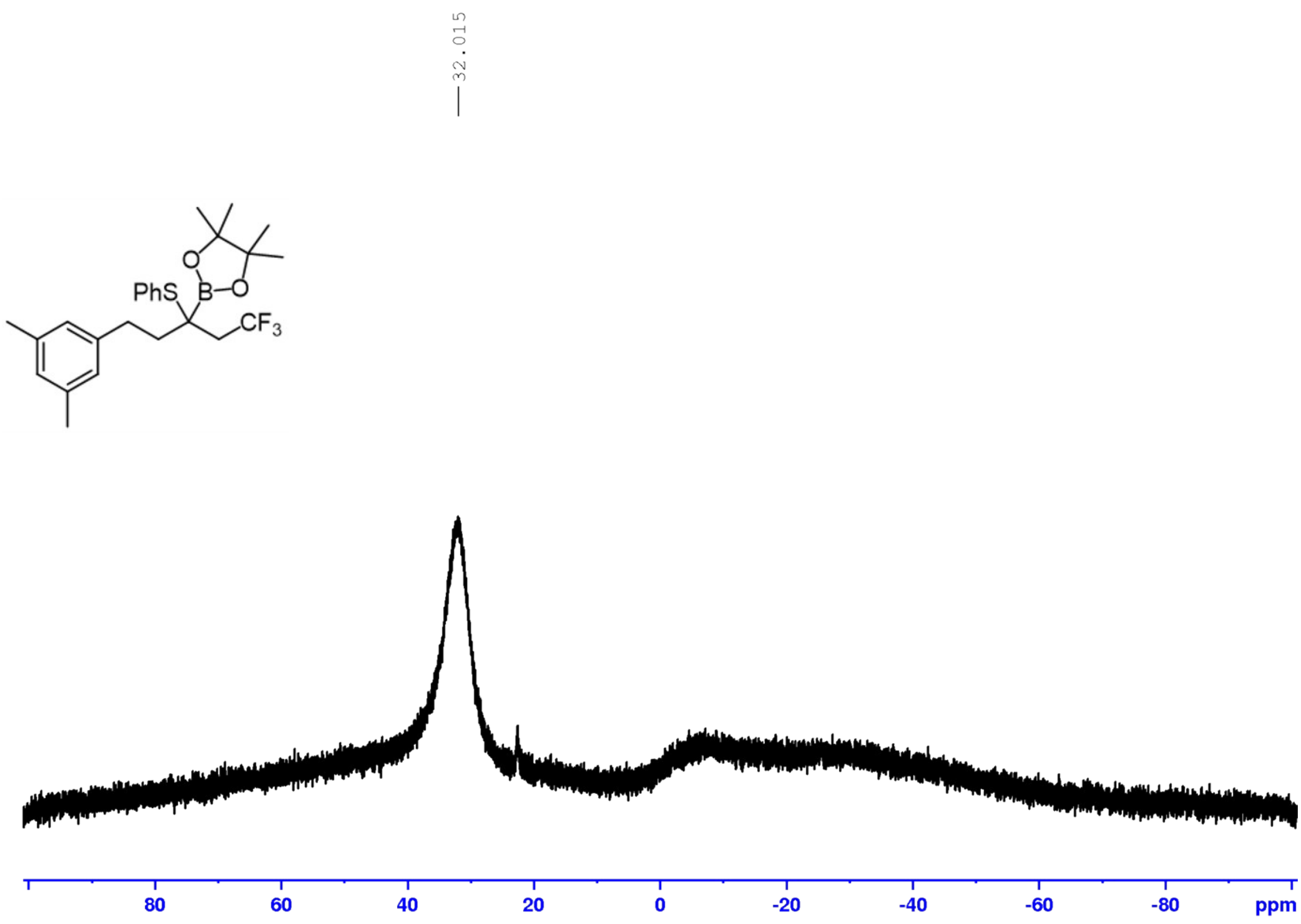

^{19}F NMR (470 MHz, Chloroform-*d*)

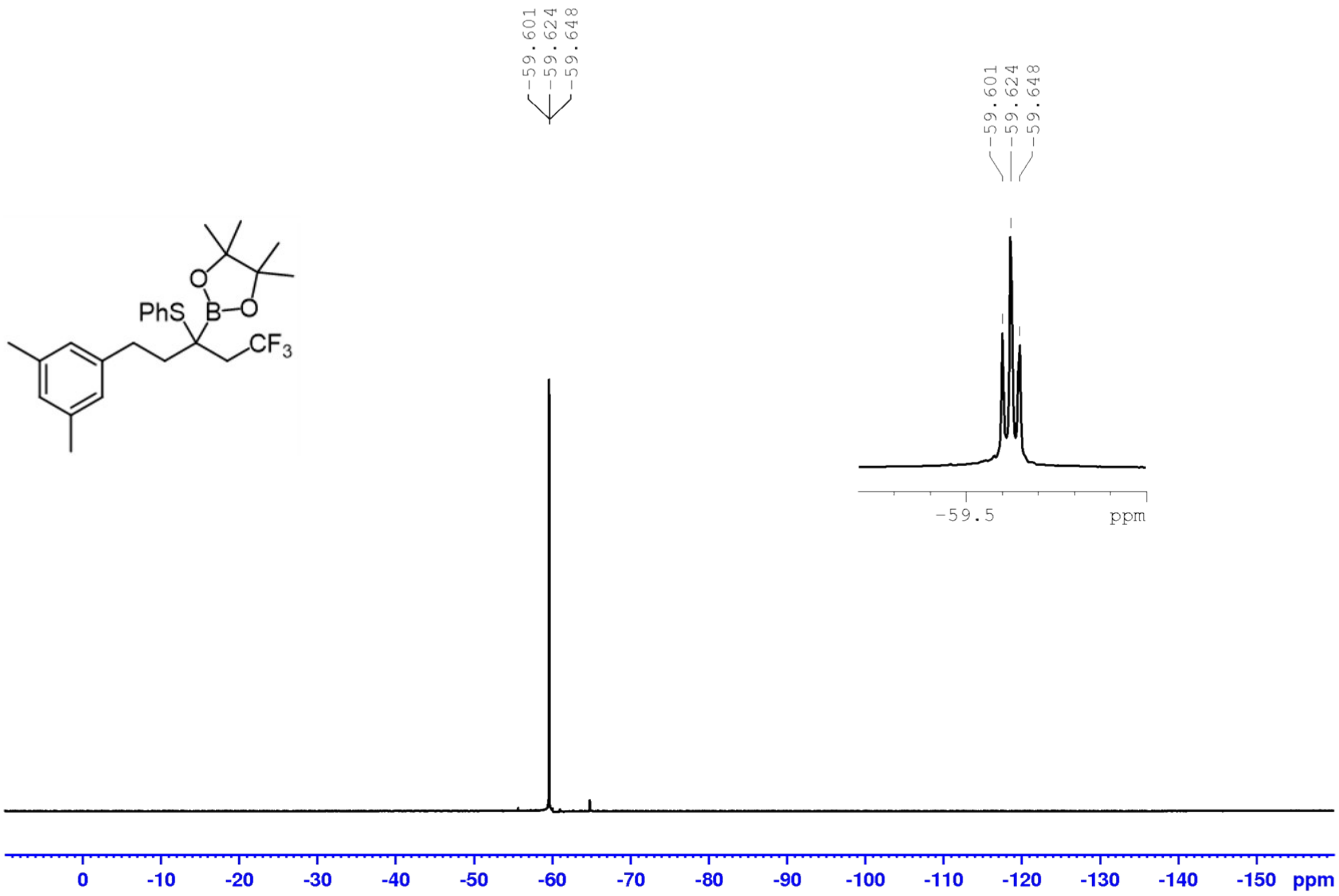

4,4,5,5-tetramethyl-2-(1,1,1-trifluoro-5-(3-methoxyphenyl)-3-(phenylthio)pentan-3-yl)-1,3,2-dioxaborolane (3ma)

^{1}H NMR (400 MHz, Chloroform-*d*)

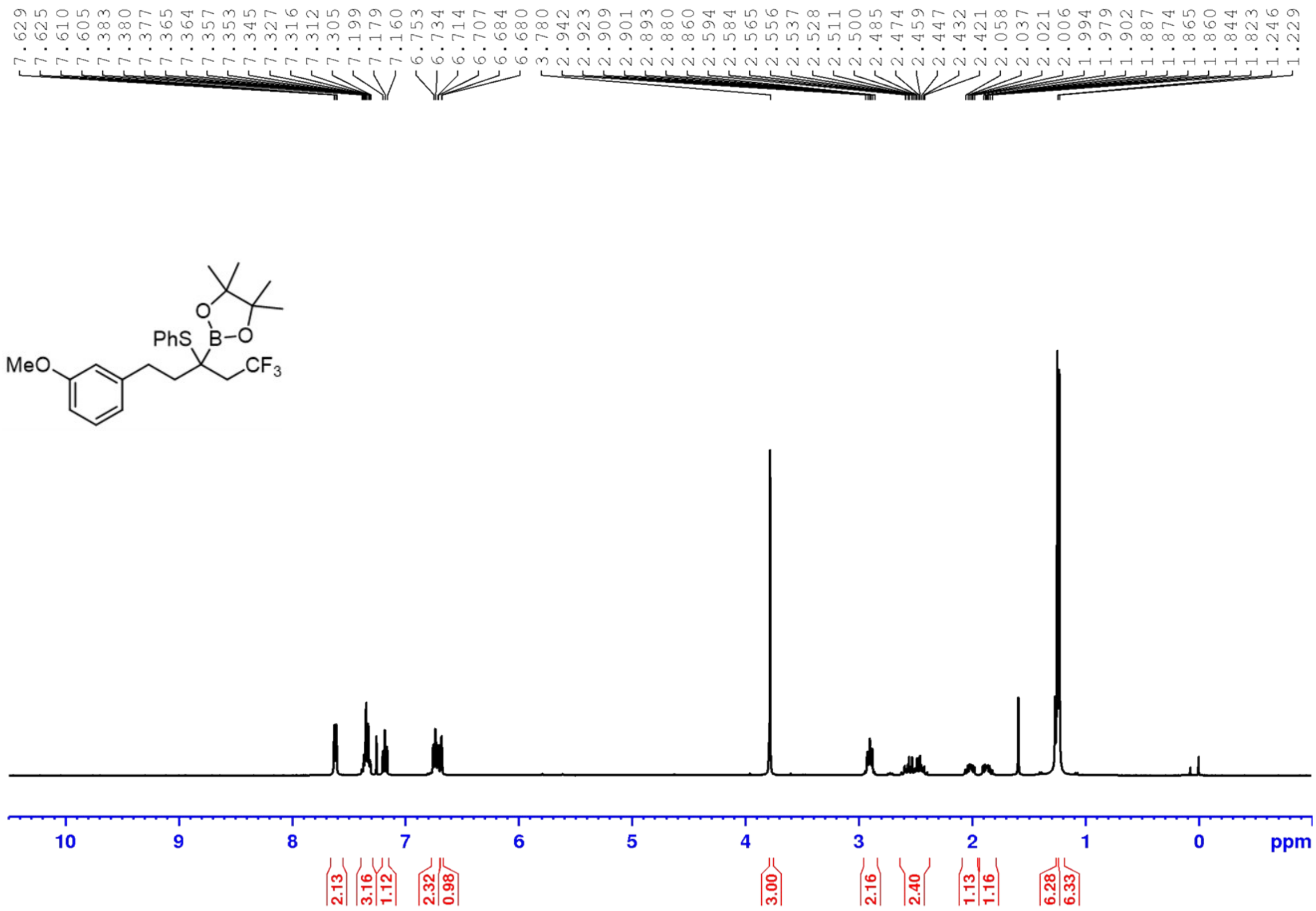

^{13}C NMR (126 MHz, Chloroform-*d*)

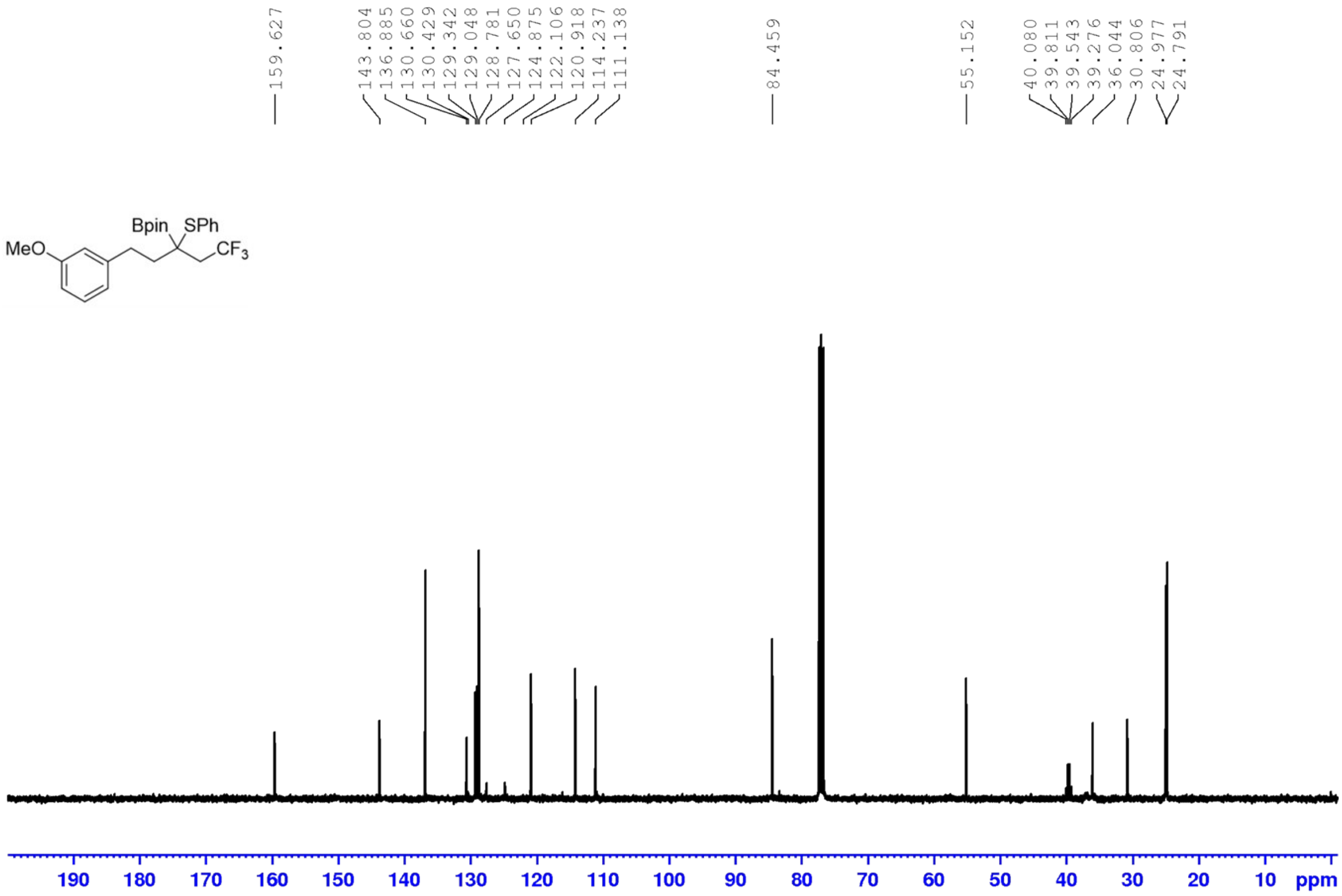

^{11}B NMR (128 MHz, Chloroform-*d*)

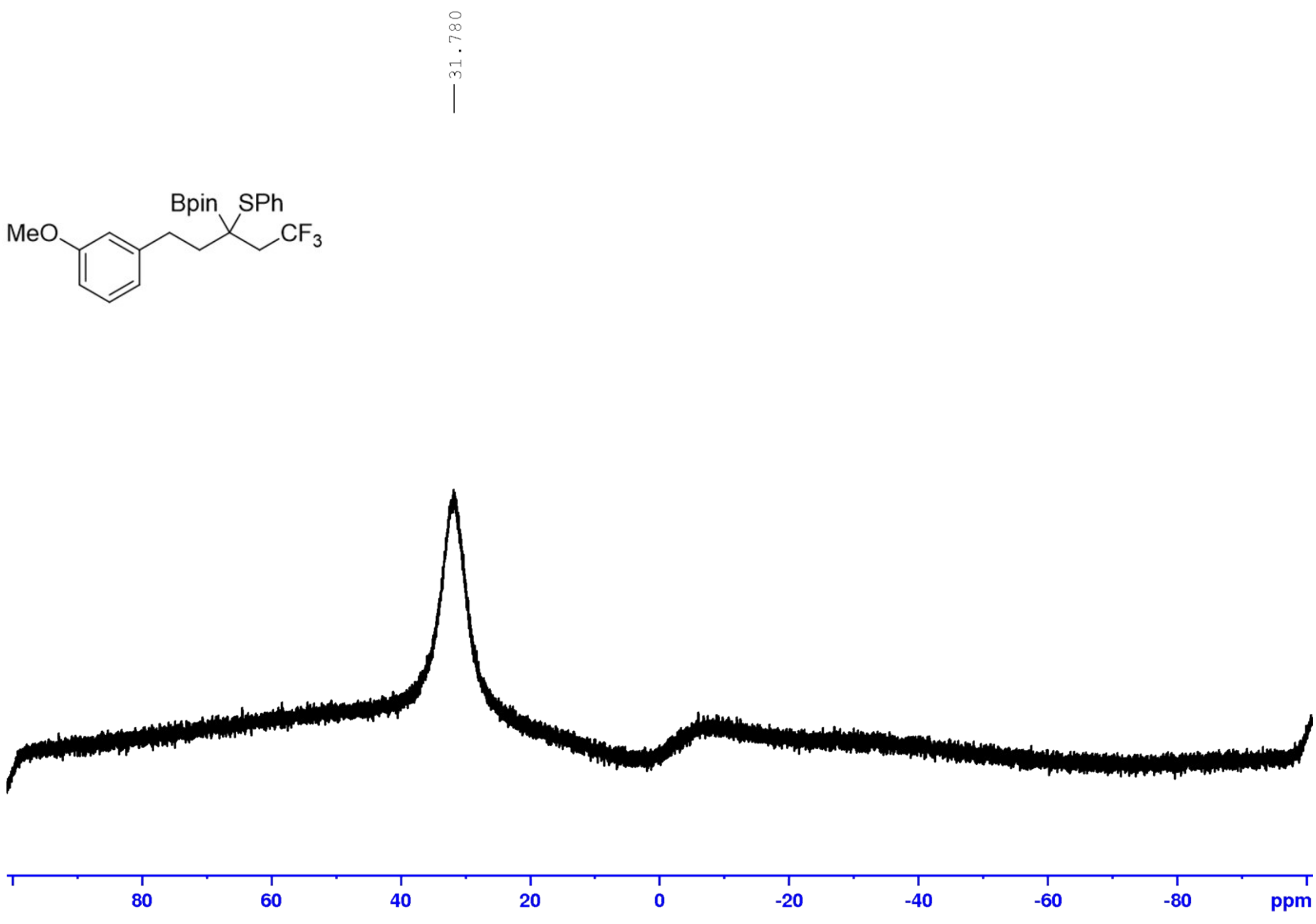

^{19}F NMR (376 MHz, Chloroform-*d*)

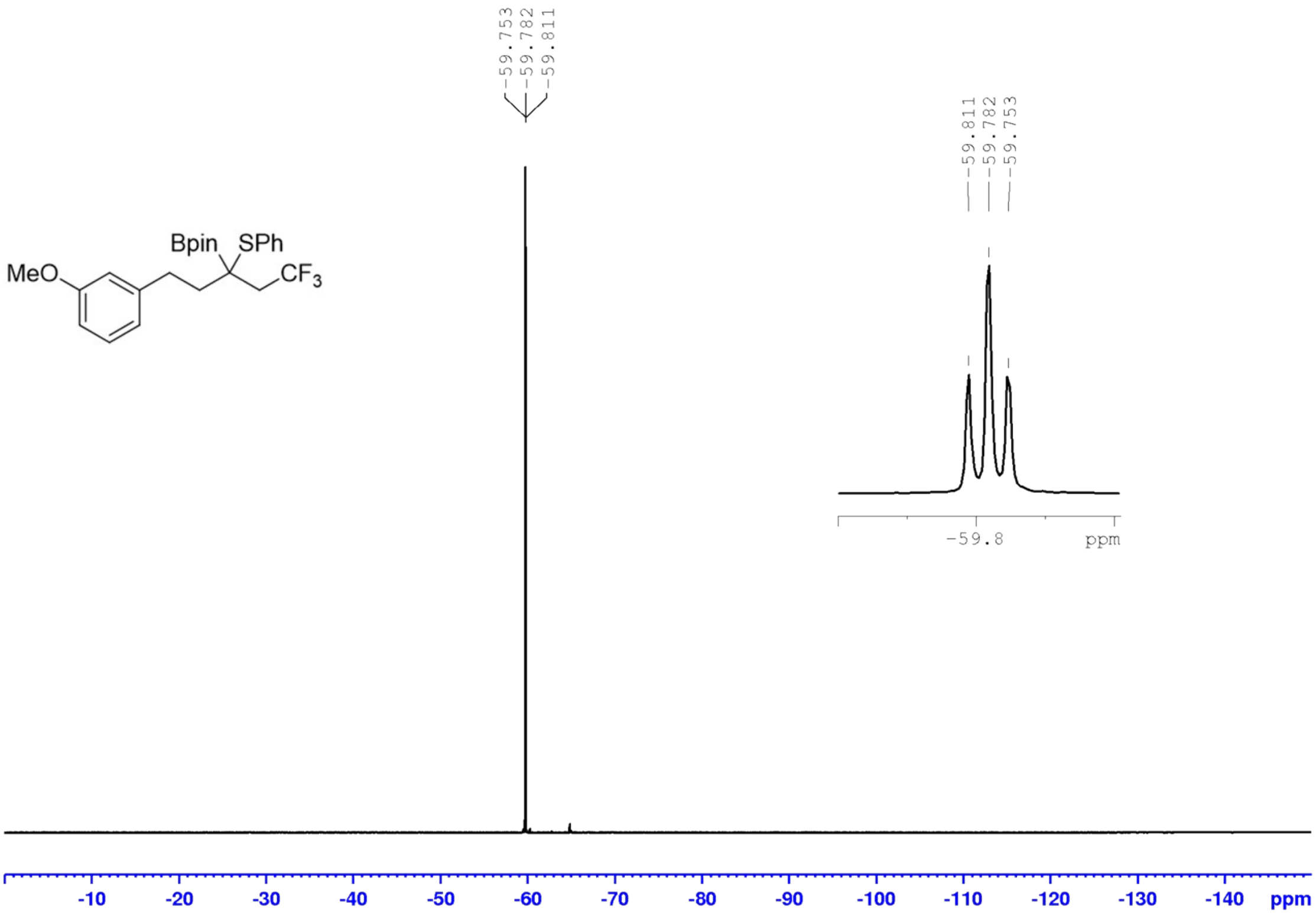

4,4,5,5-tetramethyl-2-(1,1,1-trifluoro-3-(phenylthio)decan-3-yl)-1,3,2-dioxaborolane (3na)

^{1}H NMR (500 MHz, Chloroform-*d*)

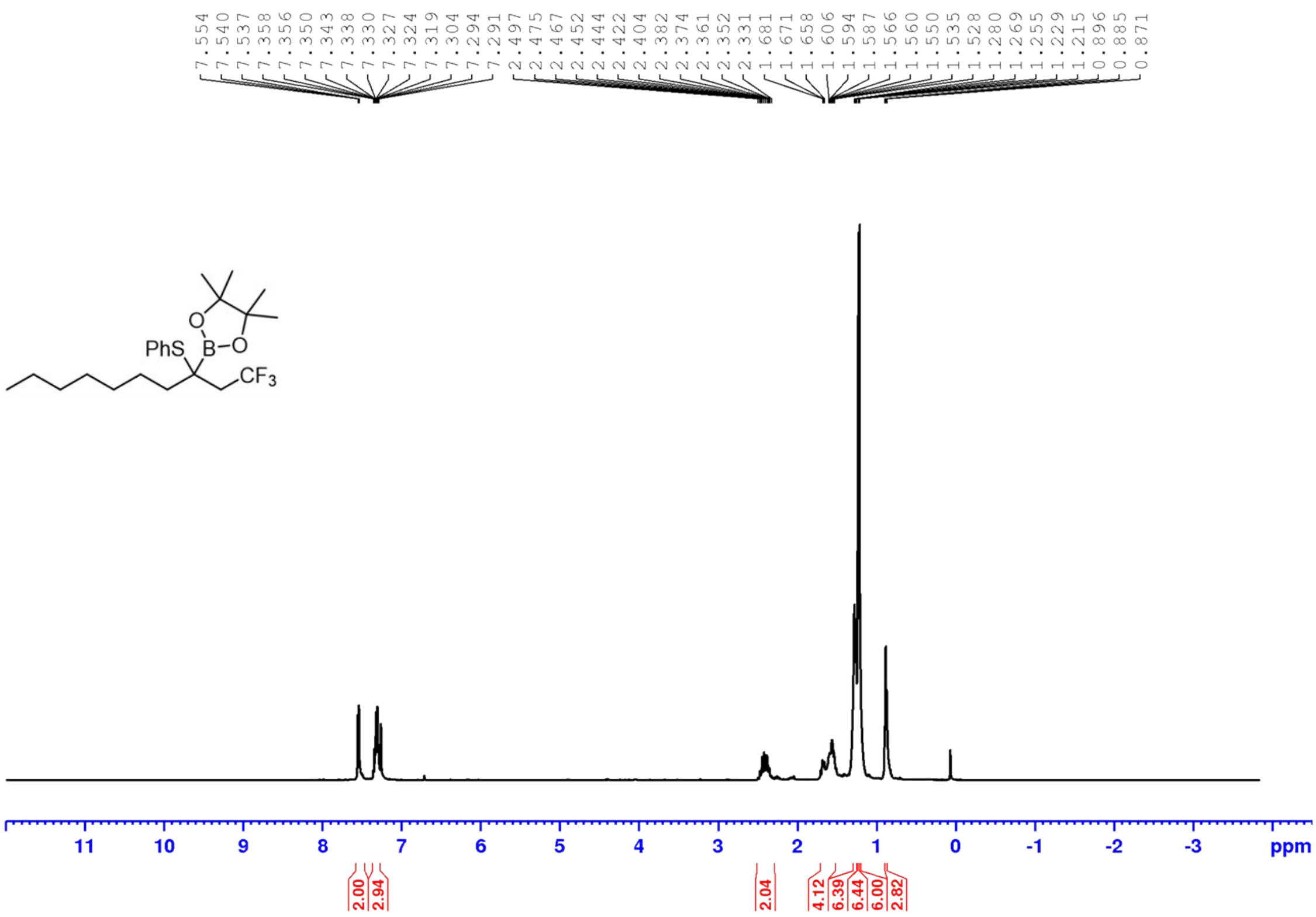

^{13}C NMR (125 MHz, Chloroform-*d*)

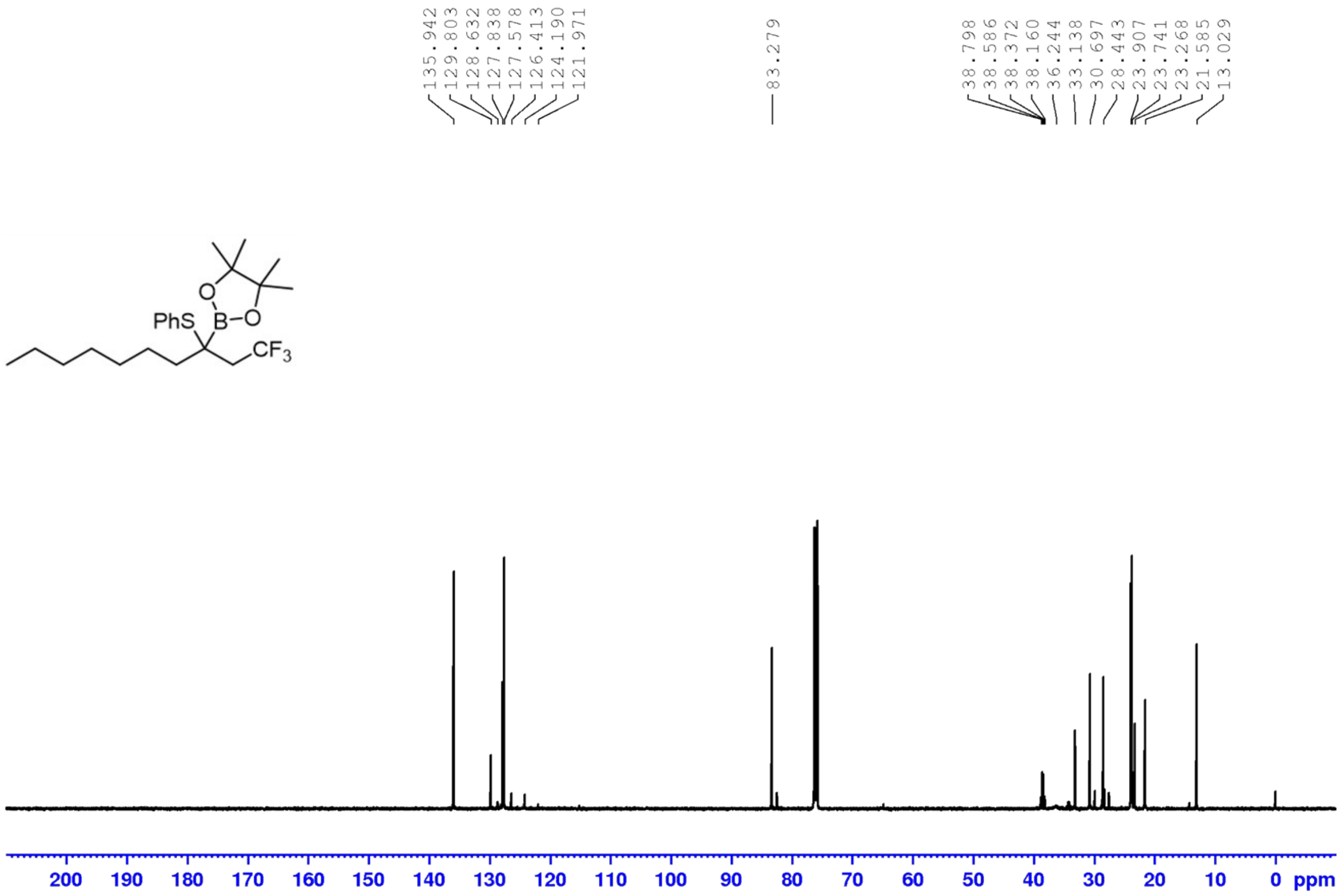

^{11}B NMR (160 MHz, Chloroform-*d*)

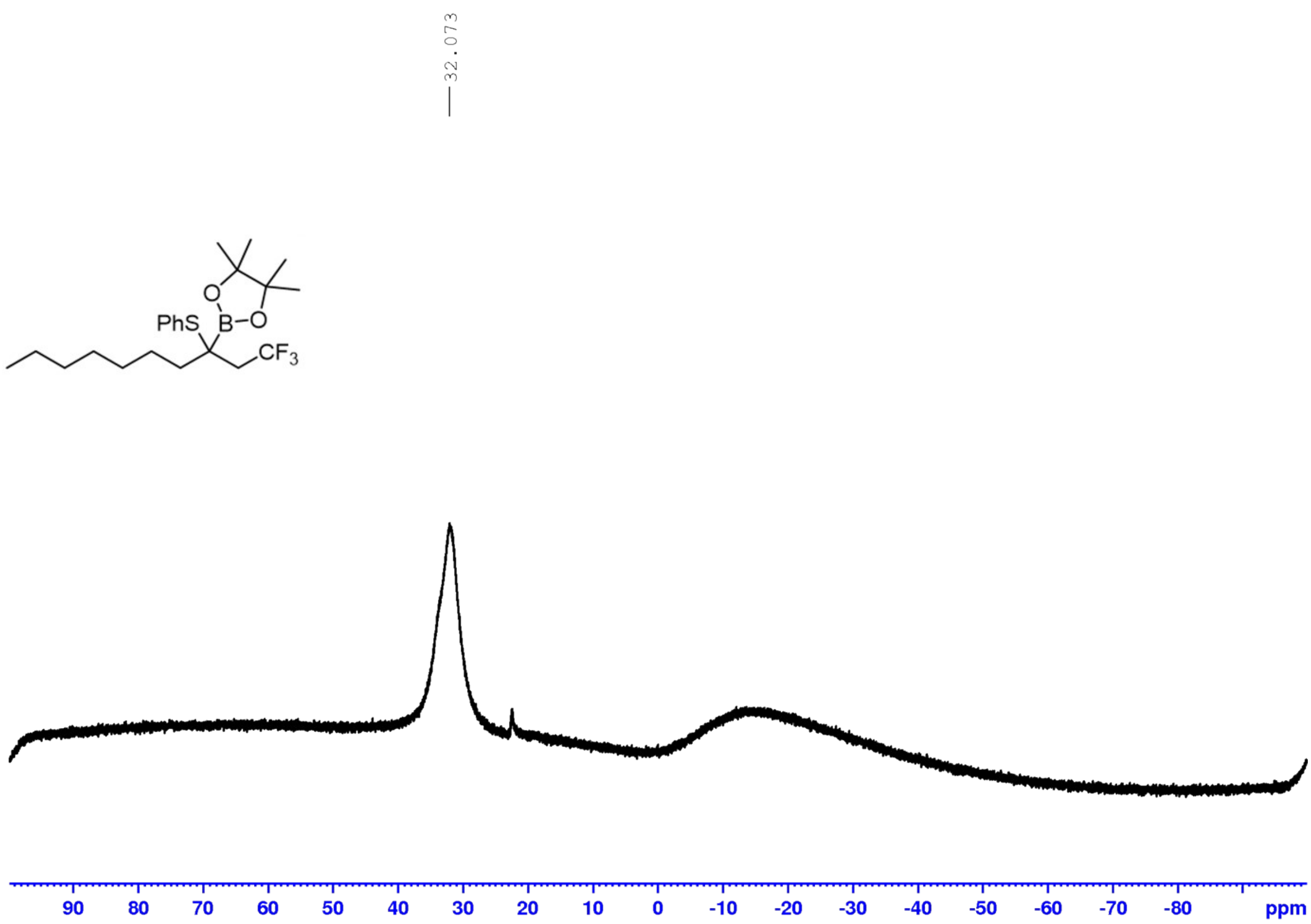

^{19}F NMR (470 MHz, Chloroform-*d*)

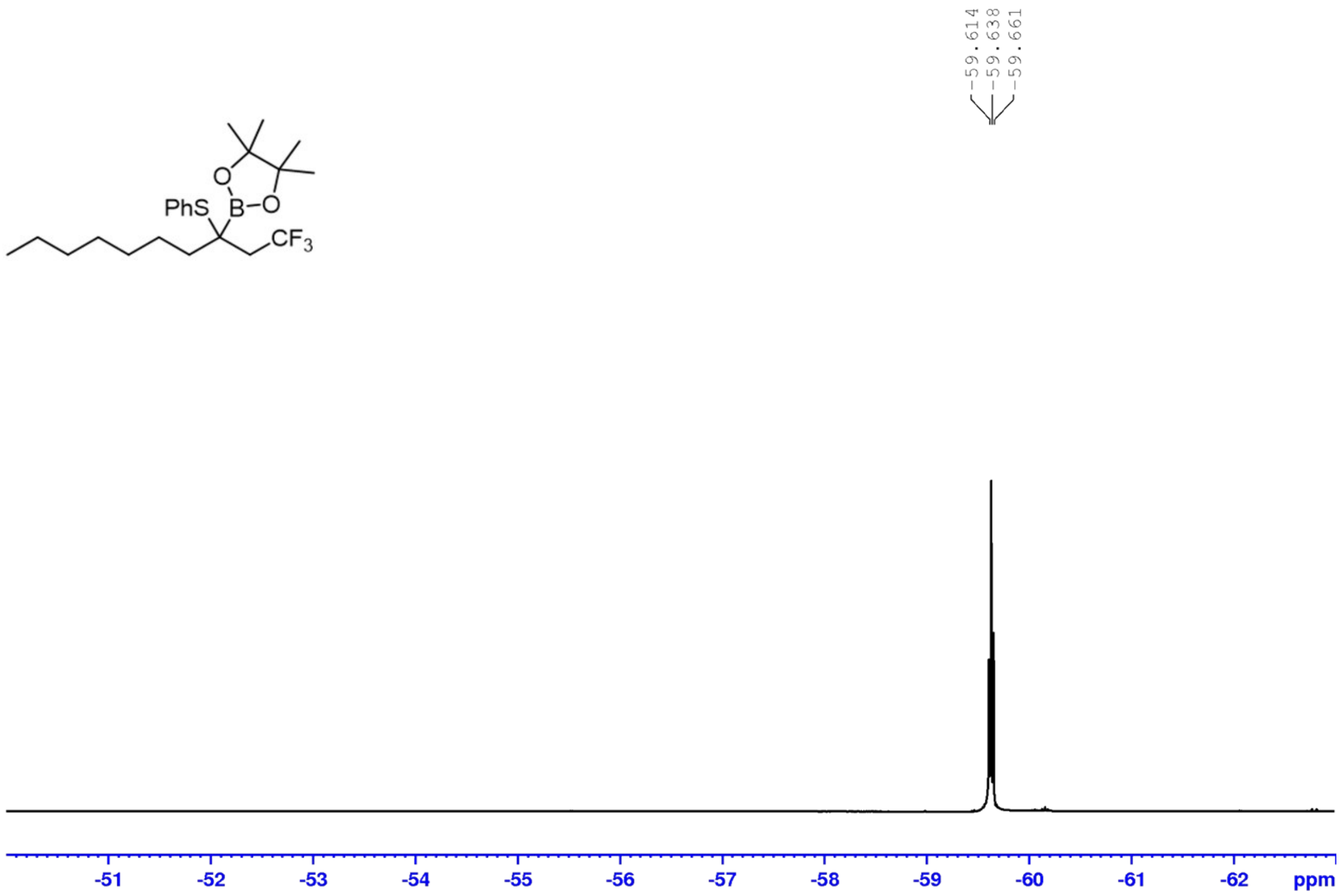

5,5,5-trifluoro-3-(phenylthio)-3-(4,4,5,5-tetramethyl-1,3,2-dioxaborolan-2-yl)pentyl pivalate (3oa)

^{1}H NMR (400 MHz, Chloroform-*d*)

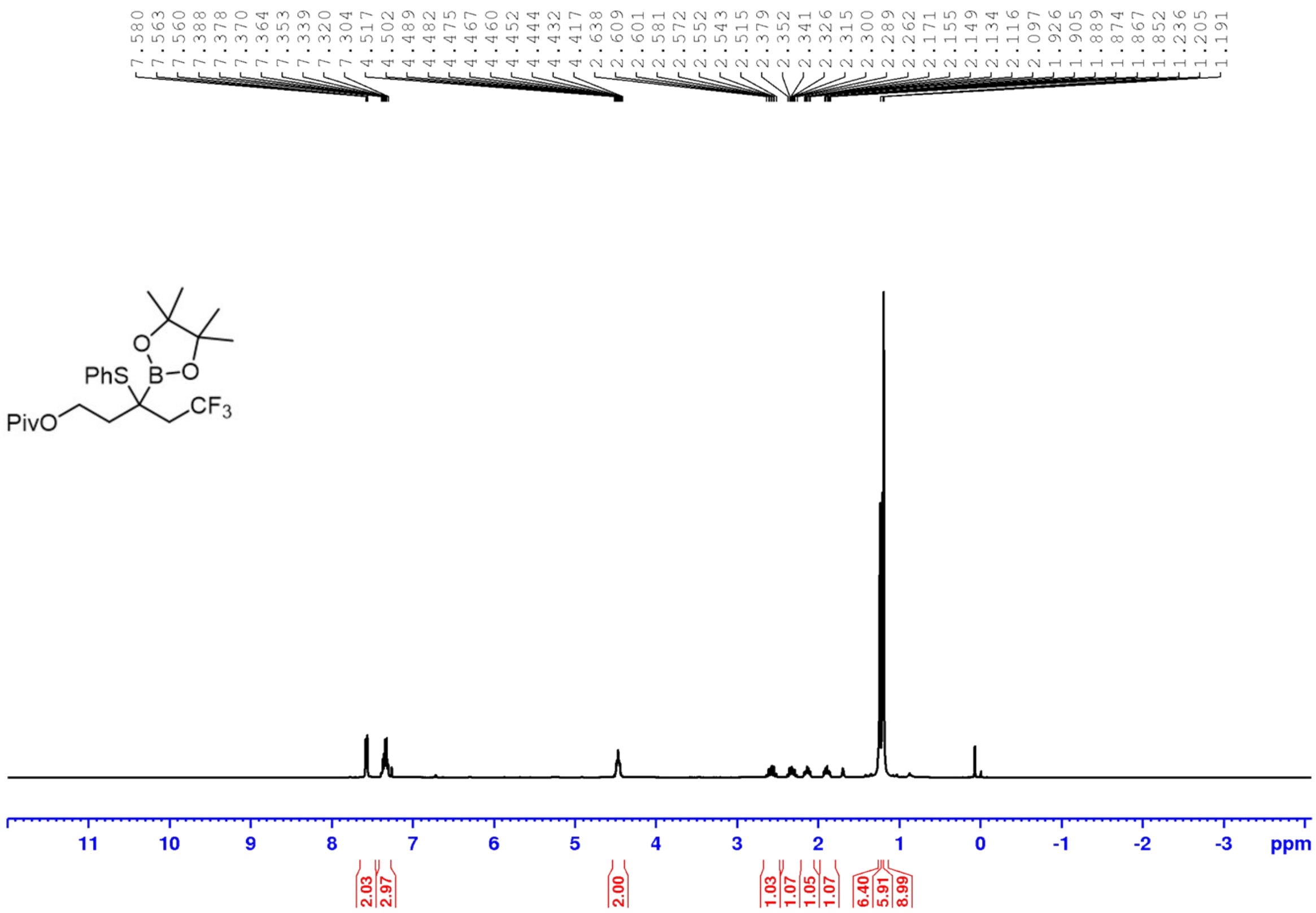

^{13}C NMR (100 MHz, Chloroform-*d*)

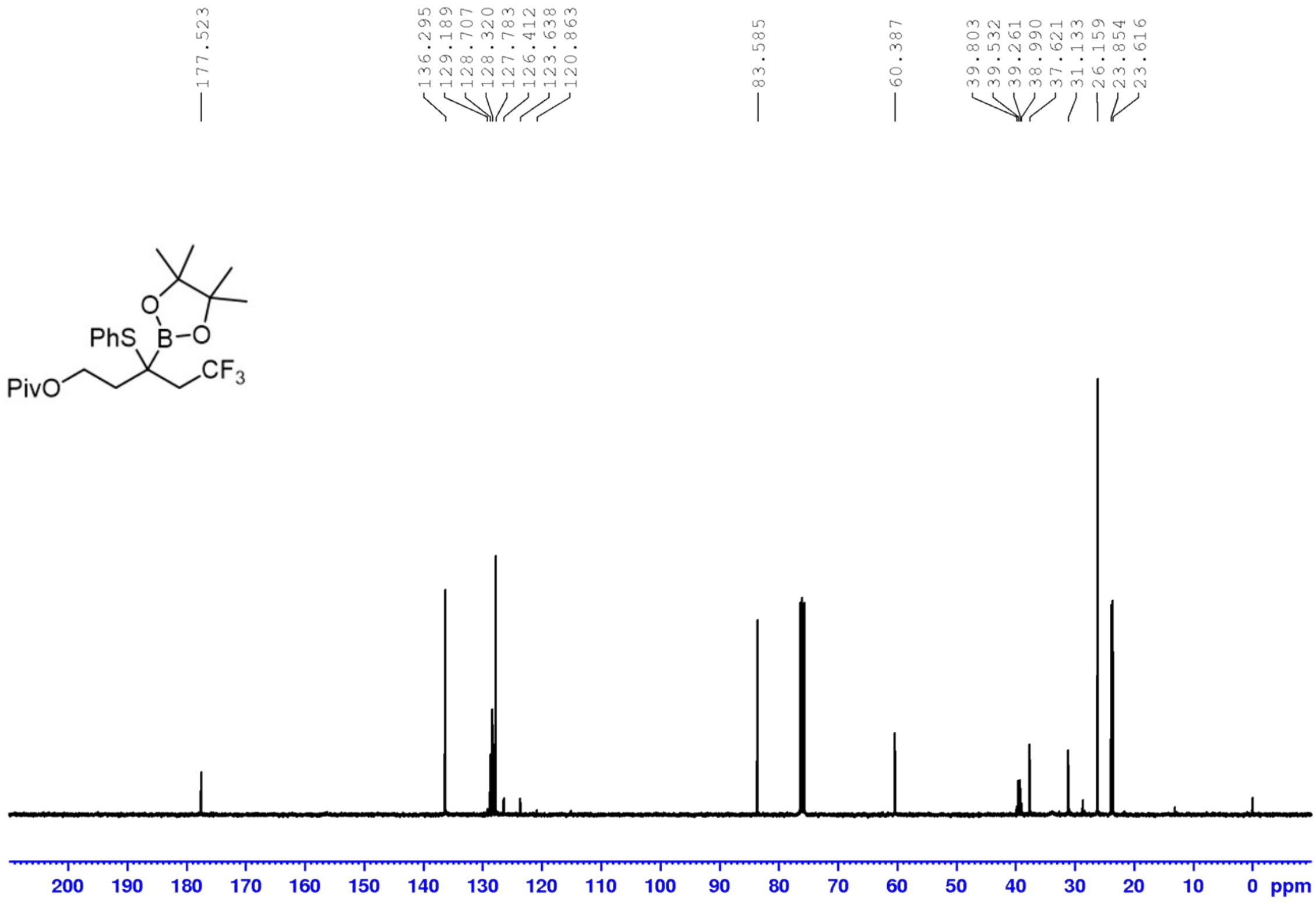

^{11}B NMR (128 MHz, Chloroform-*d*)

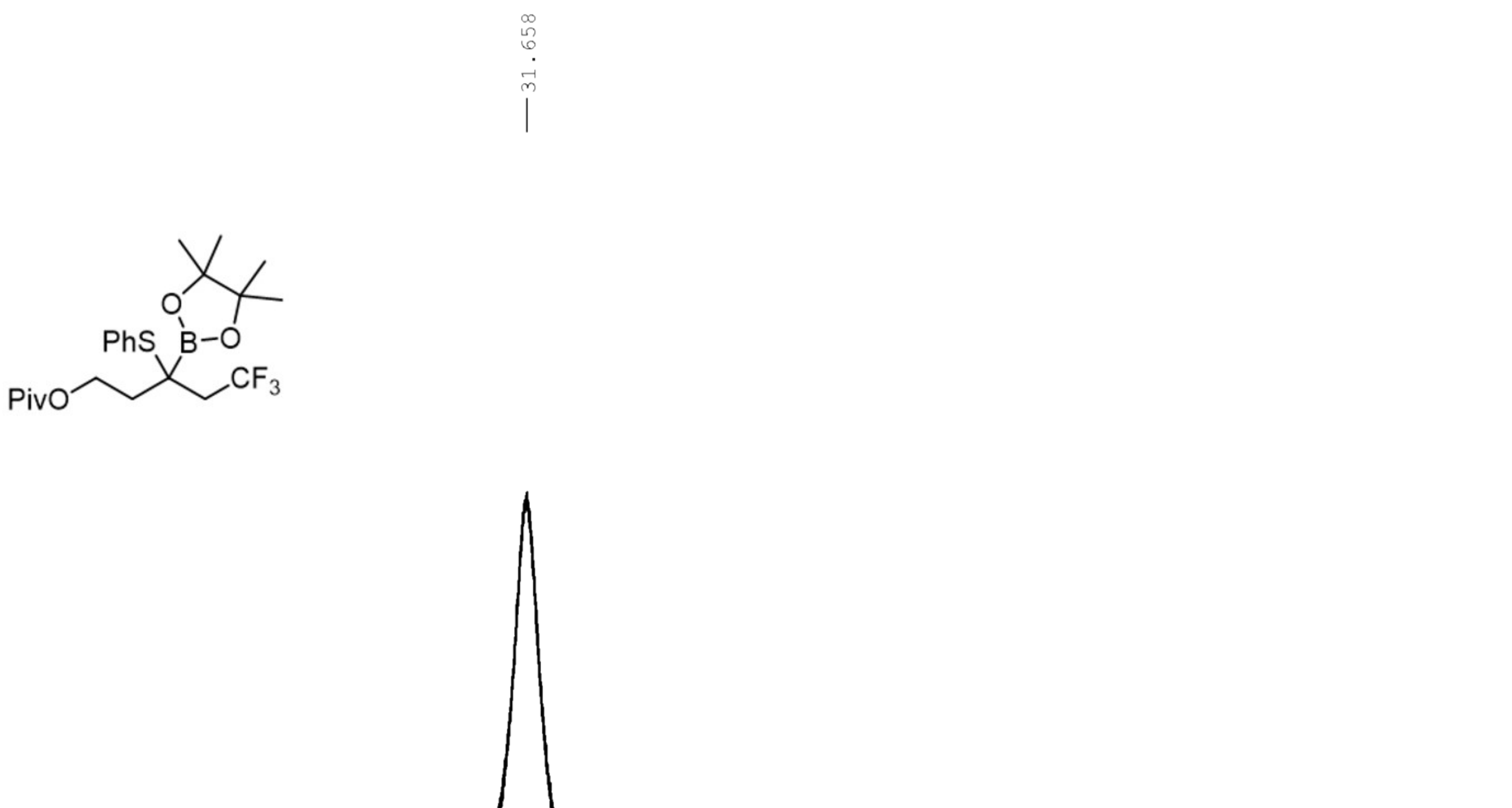

^{19}F NMR (376 MHz, Chloroform-*d*)

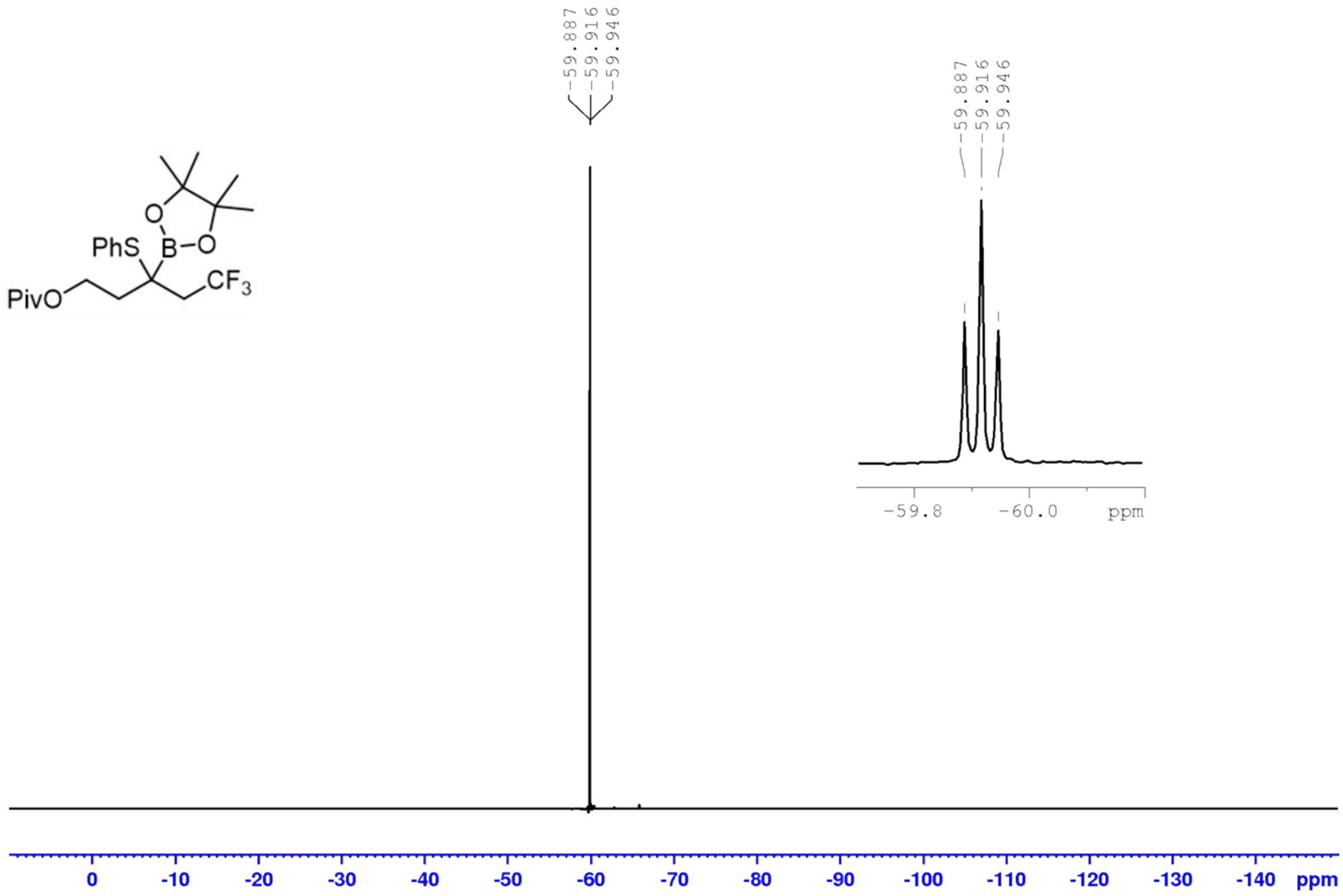

***tert*-butyldimethyl((5,5,5-trifluoro-3-(phenylthio)-3-(4,4,5,5-tetramethyl-1,3,2-dioxaborolan-2-yl)pentyl)oxy)silane (3pa)**

^{1}H NMR (500 MHz, Chloroform-*d*)

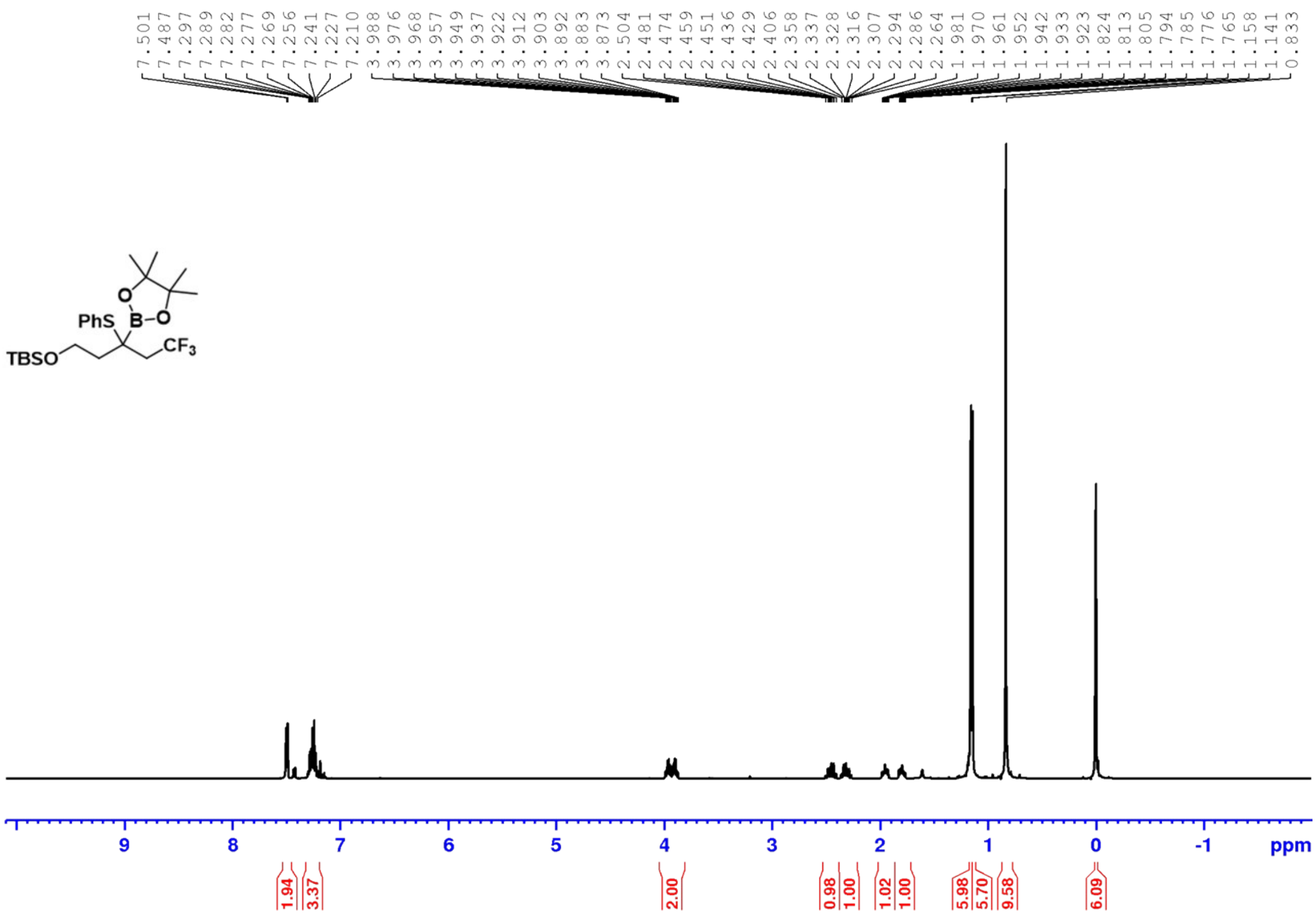

^{13}C NMR (125 MHz, Chloroform-*d*)

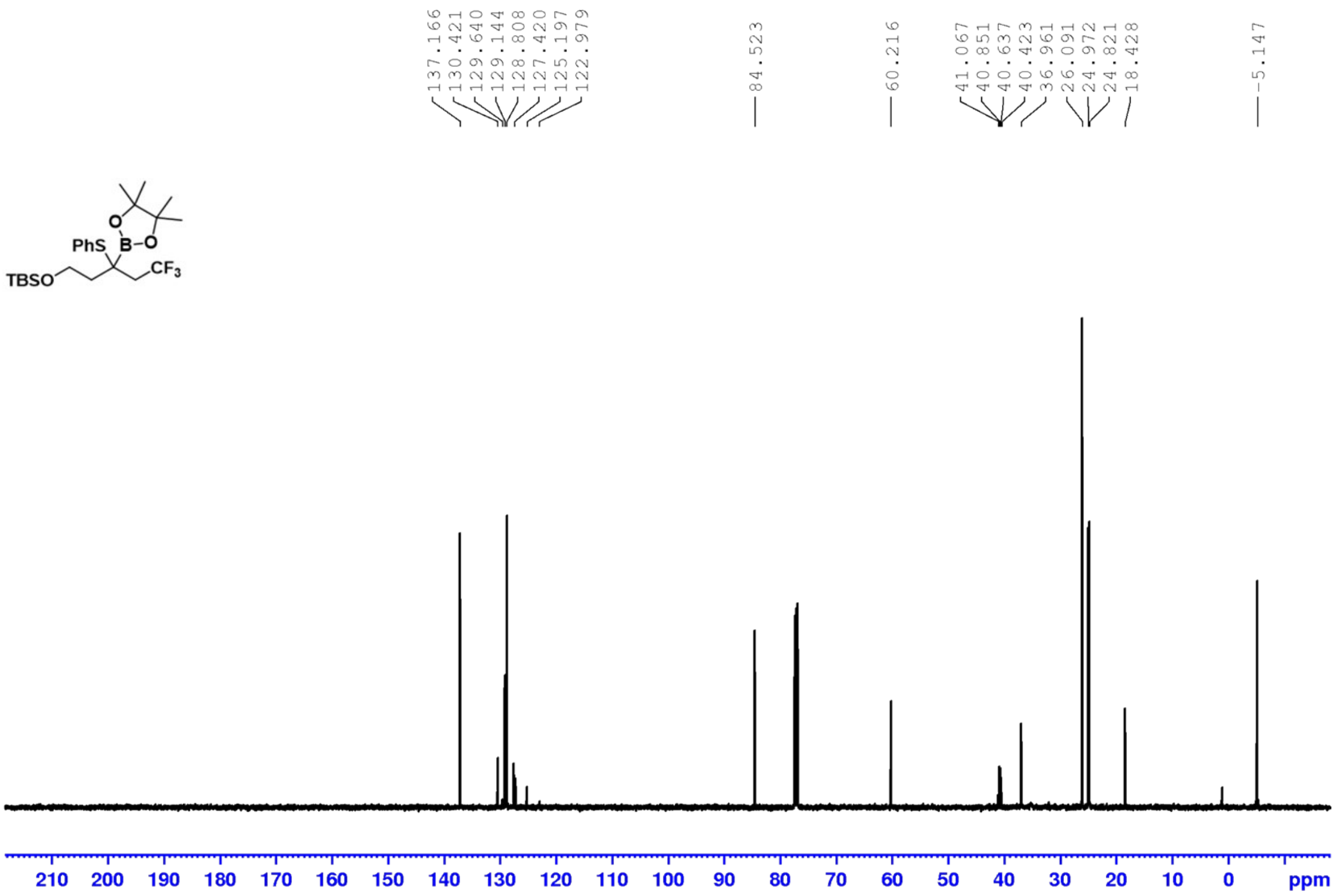

^{11}B NMR (160 MHz, Chloroform-*d*)

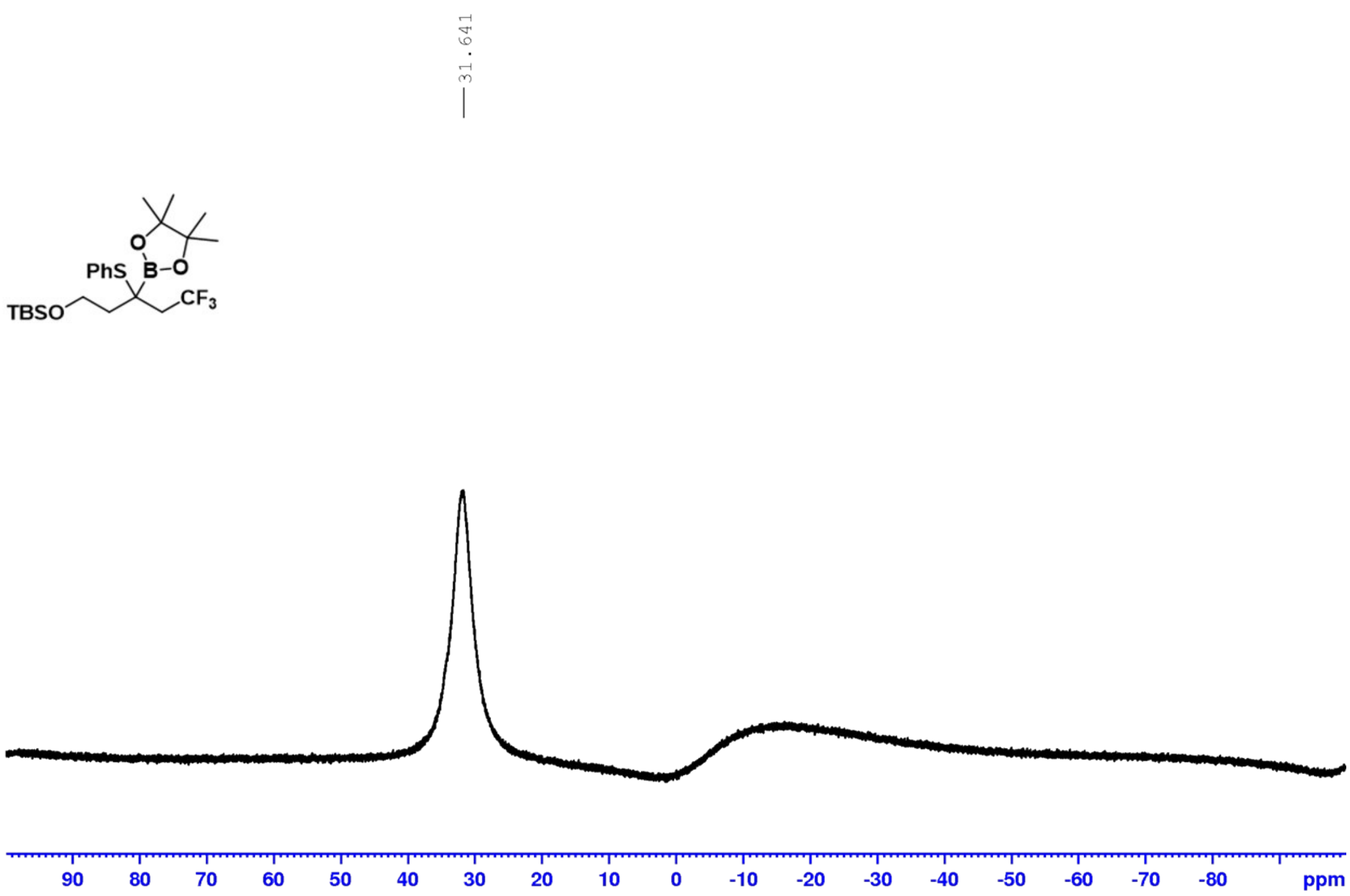

^{19}F NMR (470 MHz, Chloroform-*d*)

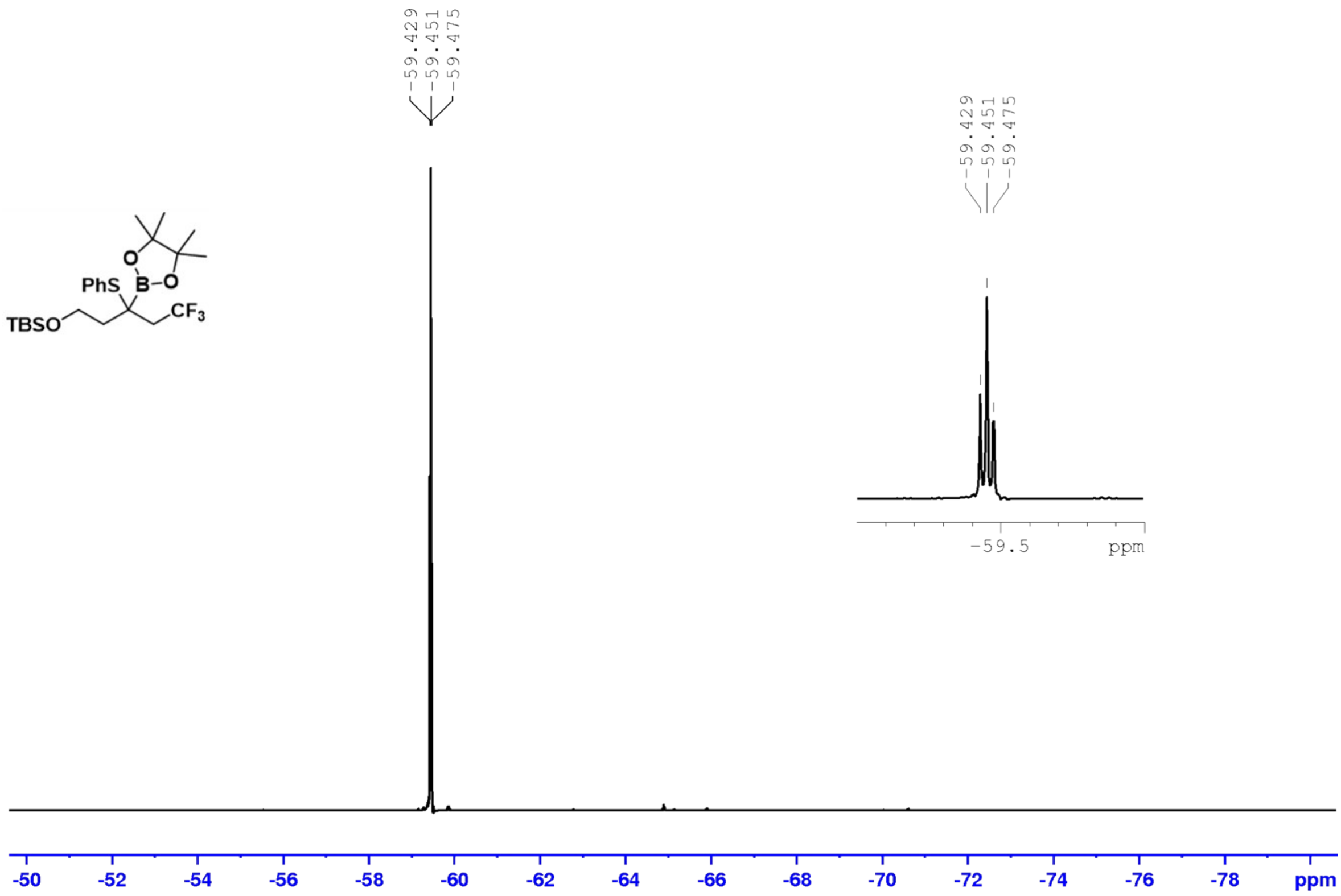

4,4,5,5-tetramethyl-2-(1,1,1-trifluoro-3-((p-tolylthio)methyl)-5-(4-(trifluoromethoxy)phenyl)pentan-3-yl)-1,3,2-dioxaborolane (5a)

^{1}H NMR (400 MHz, Chloroform-*d*)

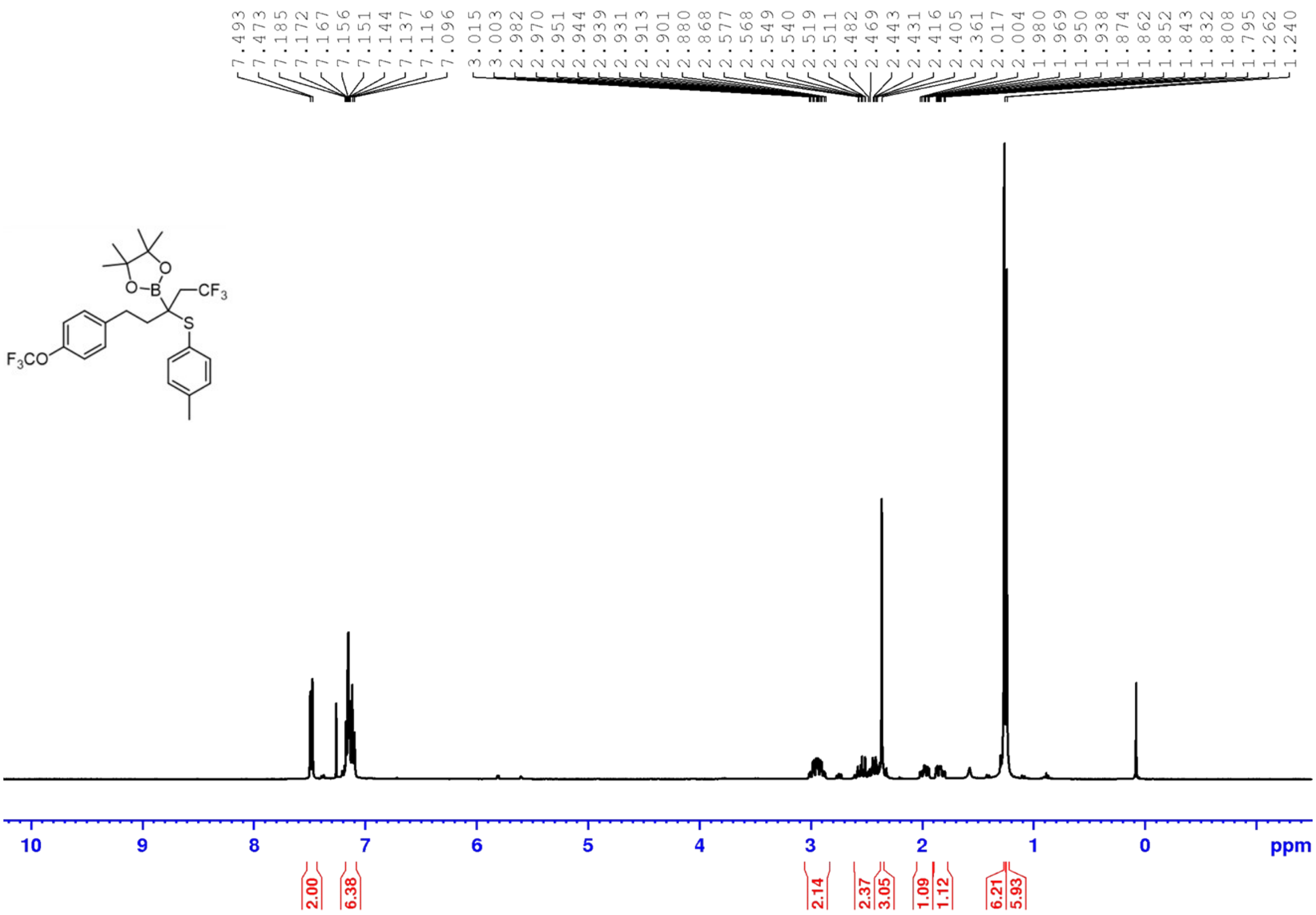

^{13}C NMR (125 MHz, Chloroform-*d*)

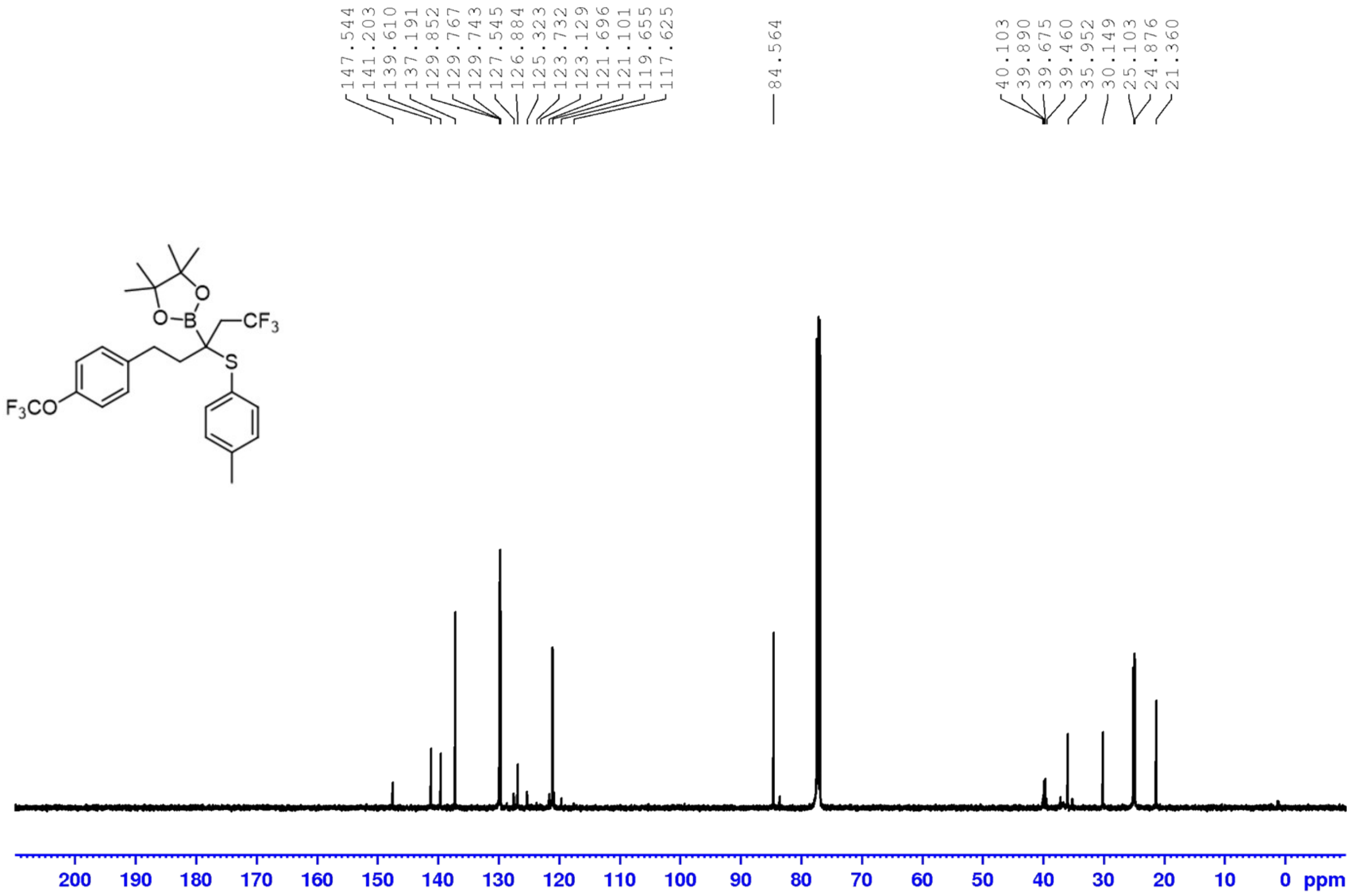

^{11}B NMR (128 MHz, Chloroform-*d*)

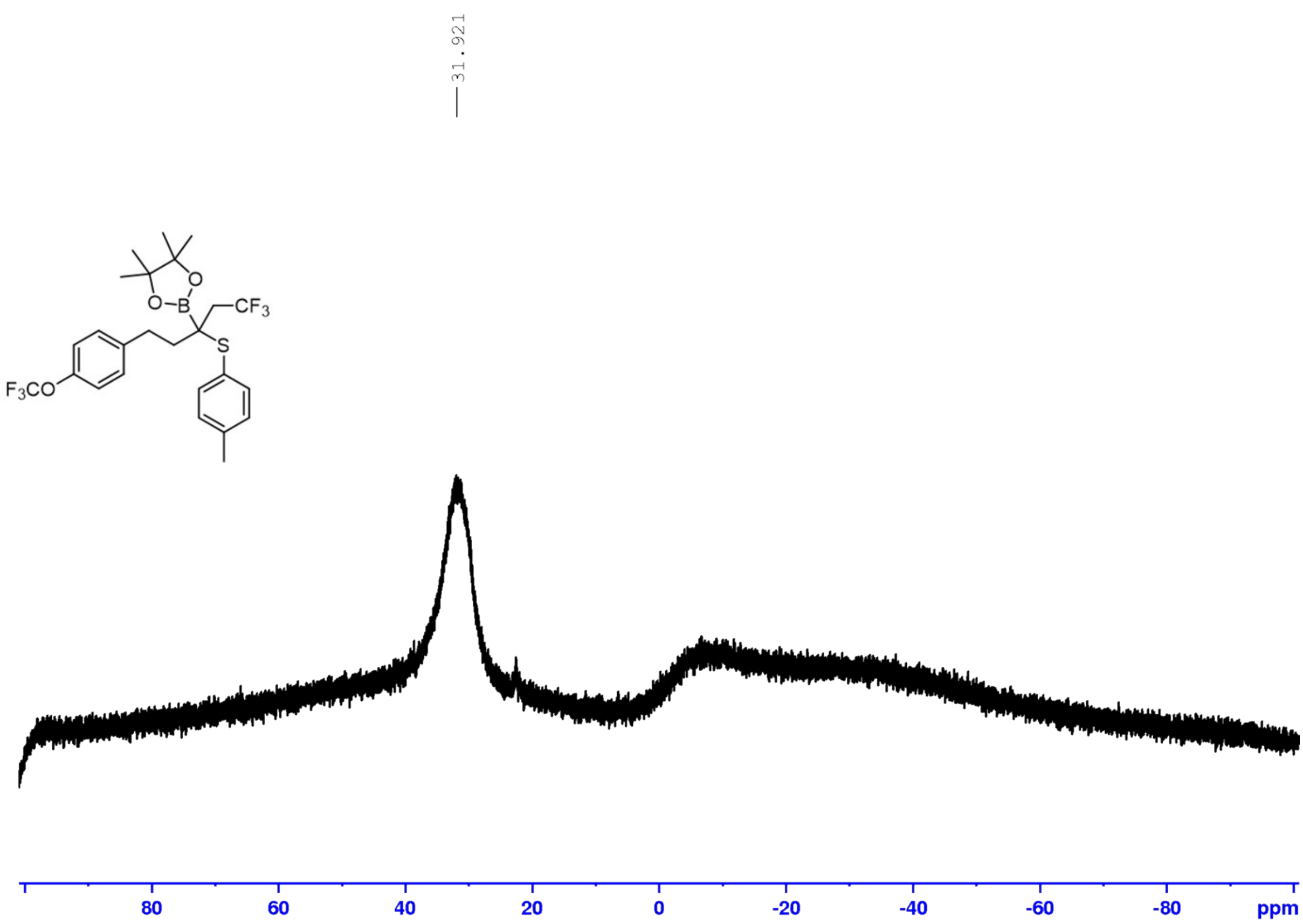

^{19}F NMR (376 MHz, Chloroform-*d*)

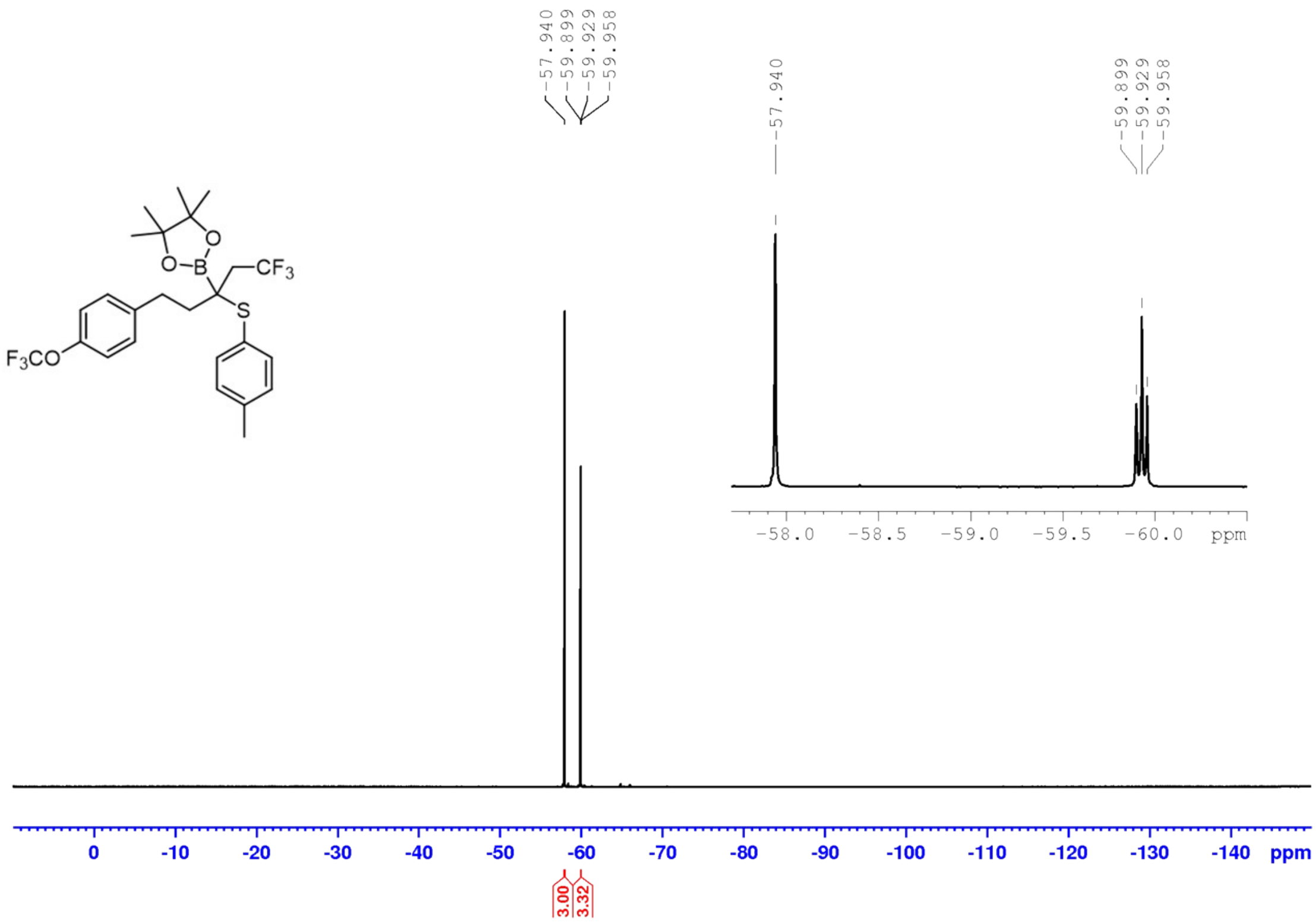

4,4,5,5-tetramethyl-2-(1,1,1-trifluoro-5-(p-tolyl)-3-((4-(trifluoromethyl)phenyl)thio)pentan-3-yl)-1,3,2-dioxaborolane (5b)

^{1}H NMR (400 MHz, Chloroform-*d*)

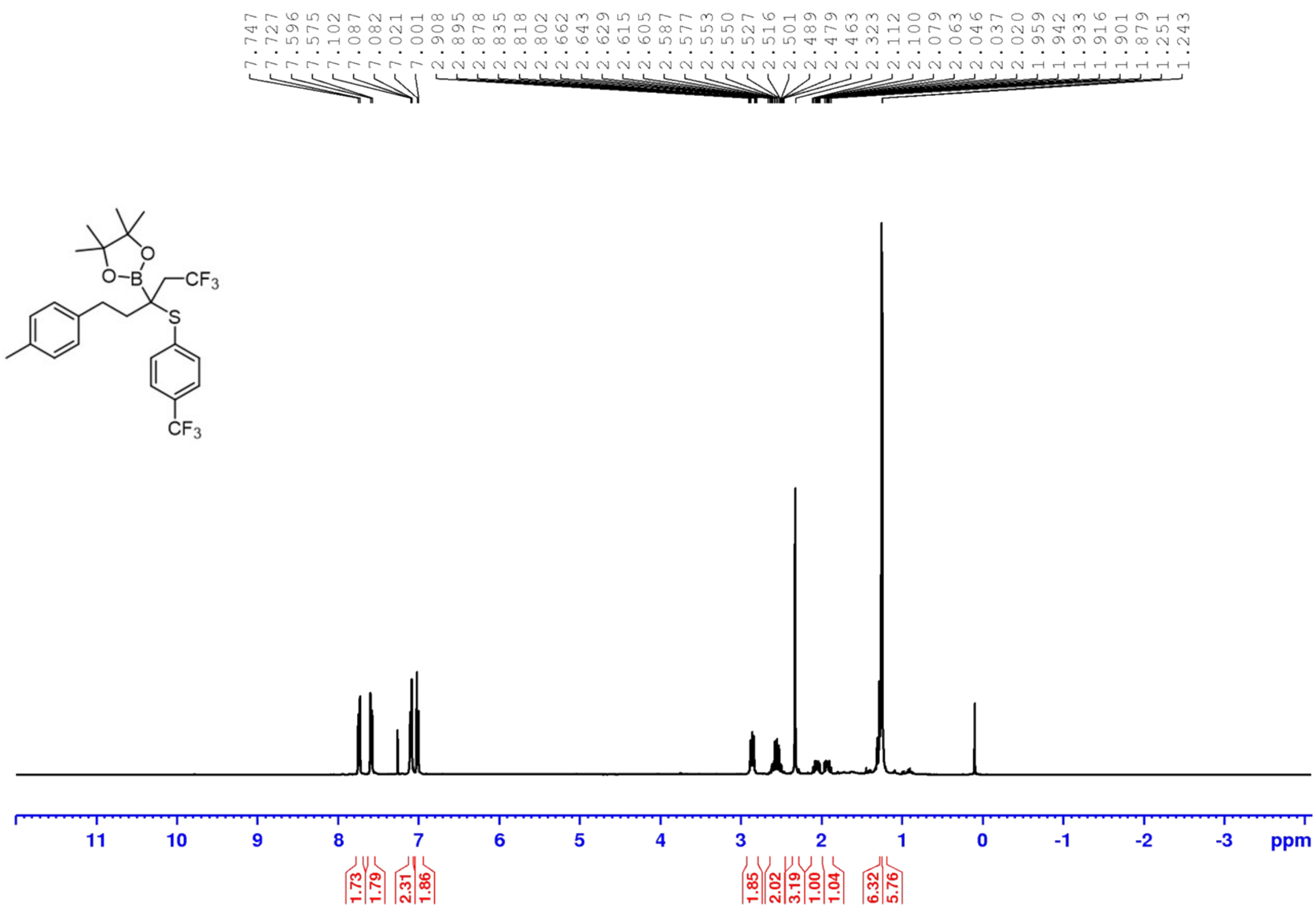

^{13}C NMR (100 MHz, Chloroform-*d*)

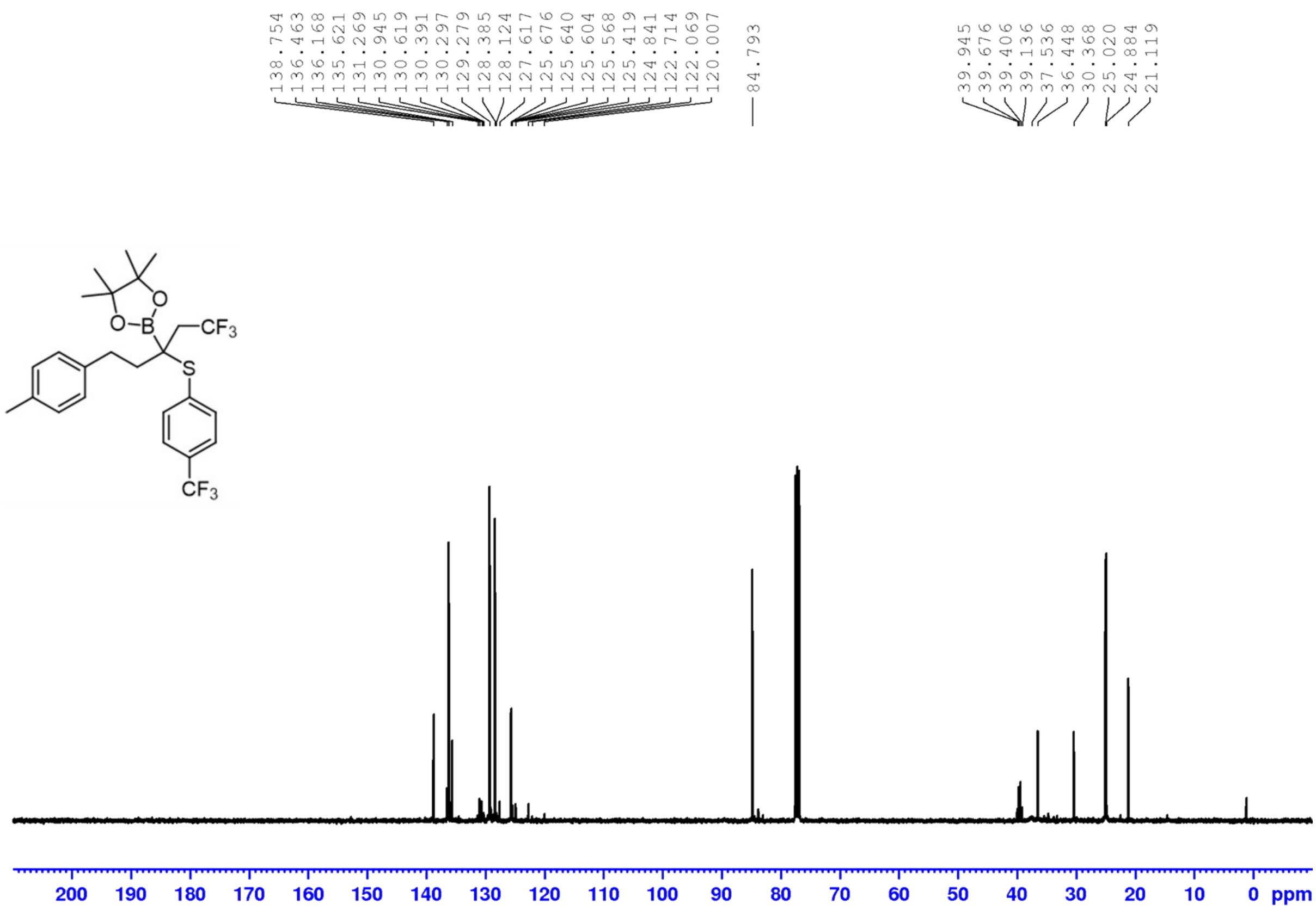

^{11}B NMR (128 MHz, Chloroform-*d*)

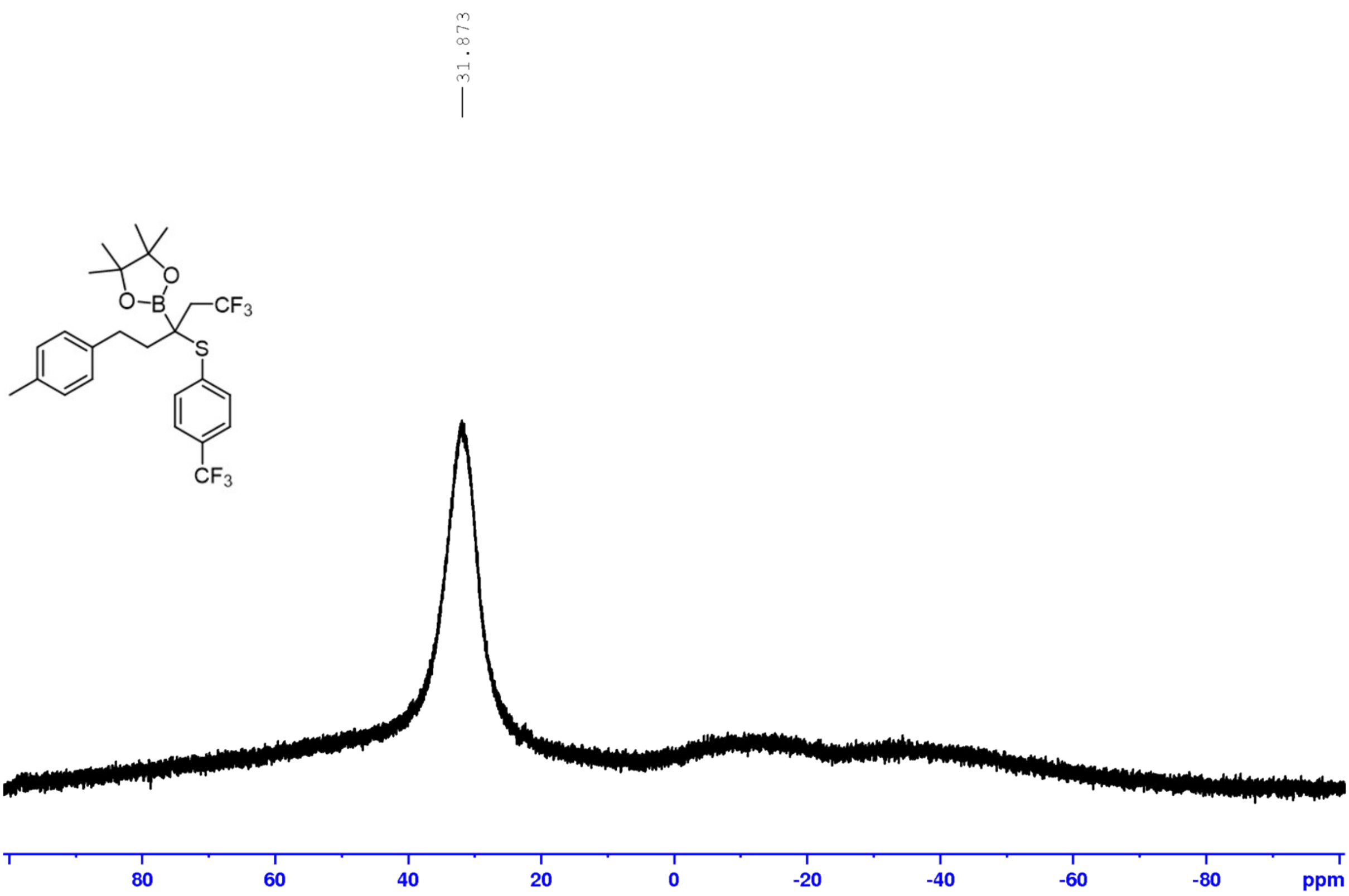

^{19}F NMR (376 MHz, Chloroform-*d*)

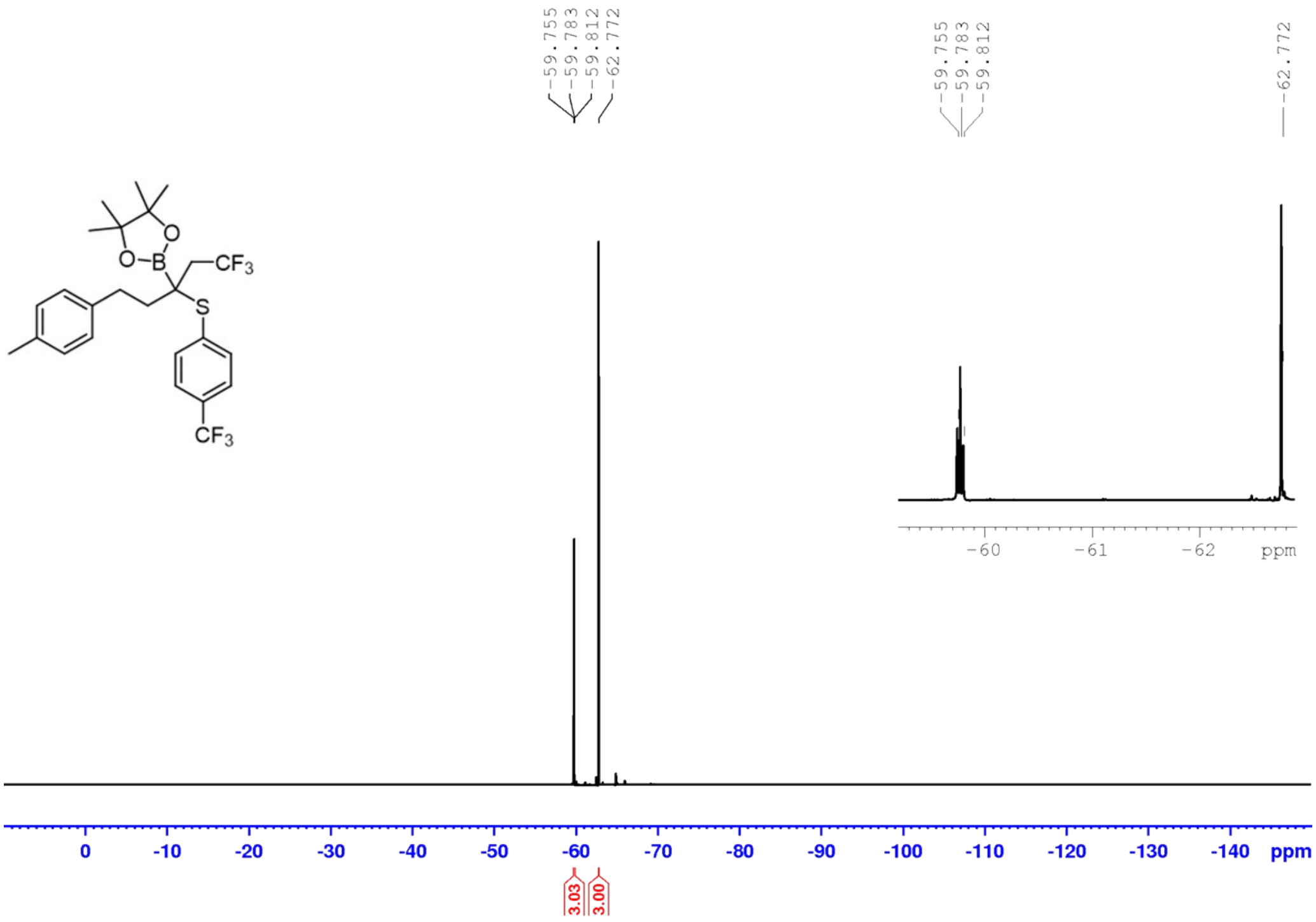

2-(3-((2,4-difluorophenyl)thio)-1,1,1-trifluoro-5-(4-fluorophenyl)pentan-3-yl)-4,4,5,5-tetramethyl-1,3,2-dioxaborolane (5c)

^{1}H NMR (400 MHz, Chloroform-*d*)

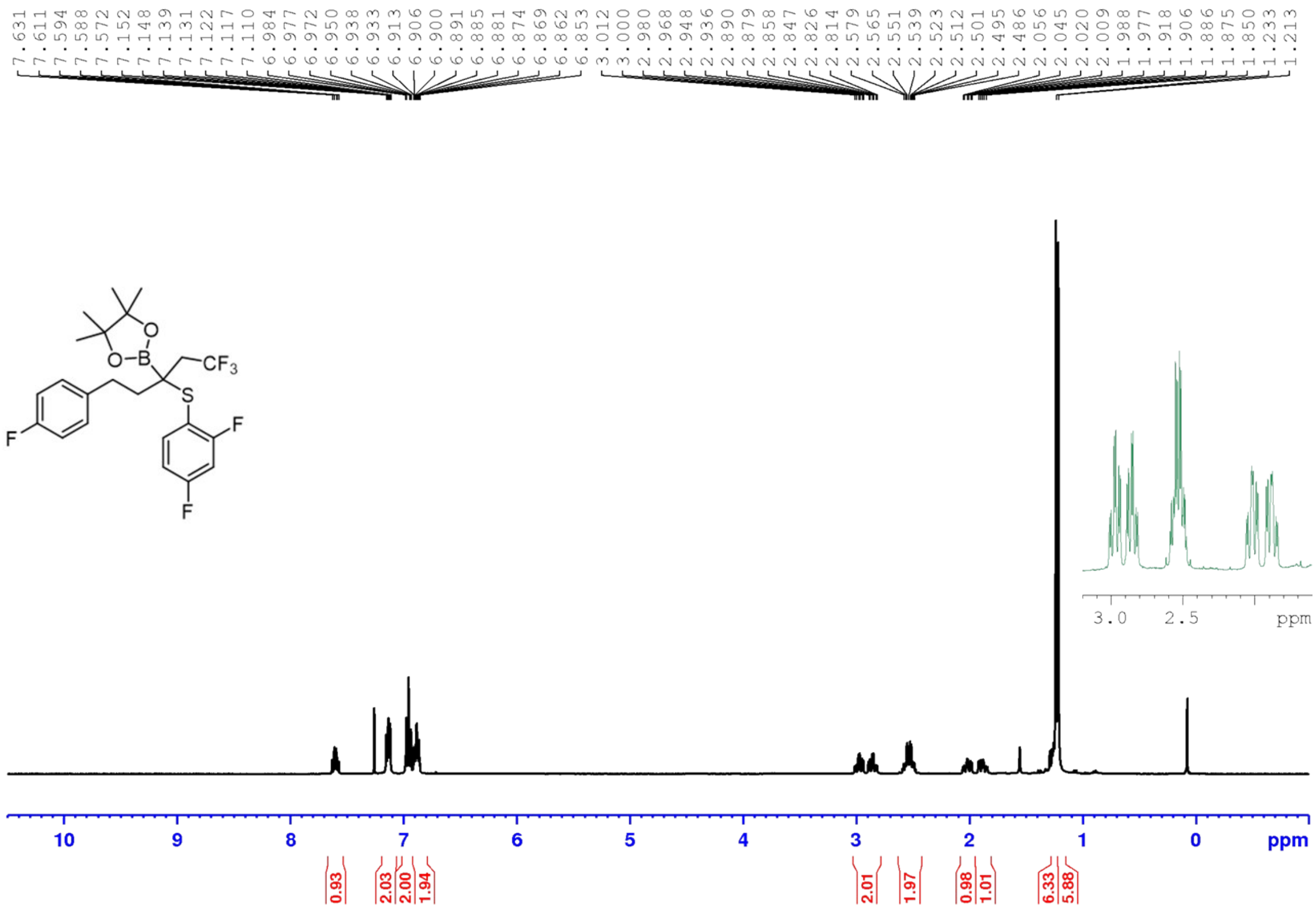

^{13}C NMR (100 MHz, Chloroform-*d*)

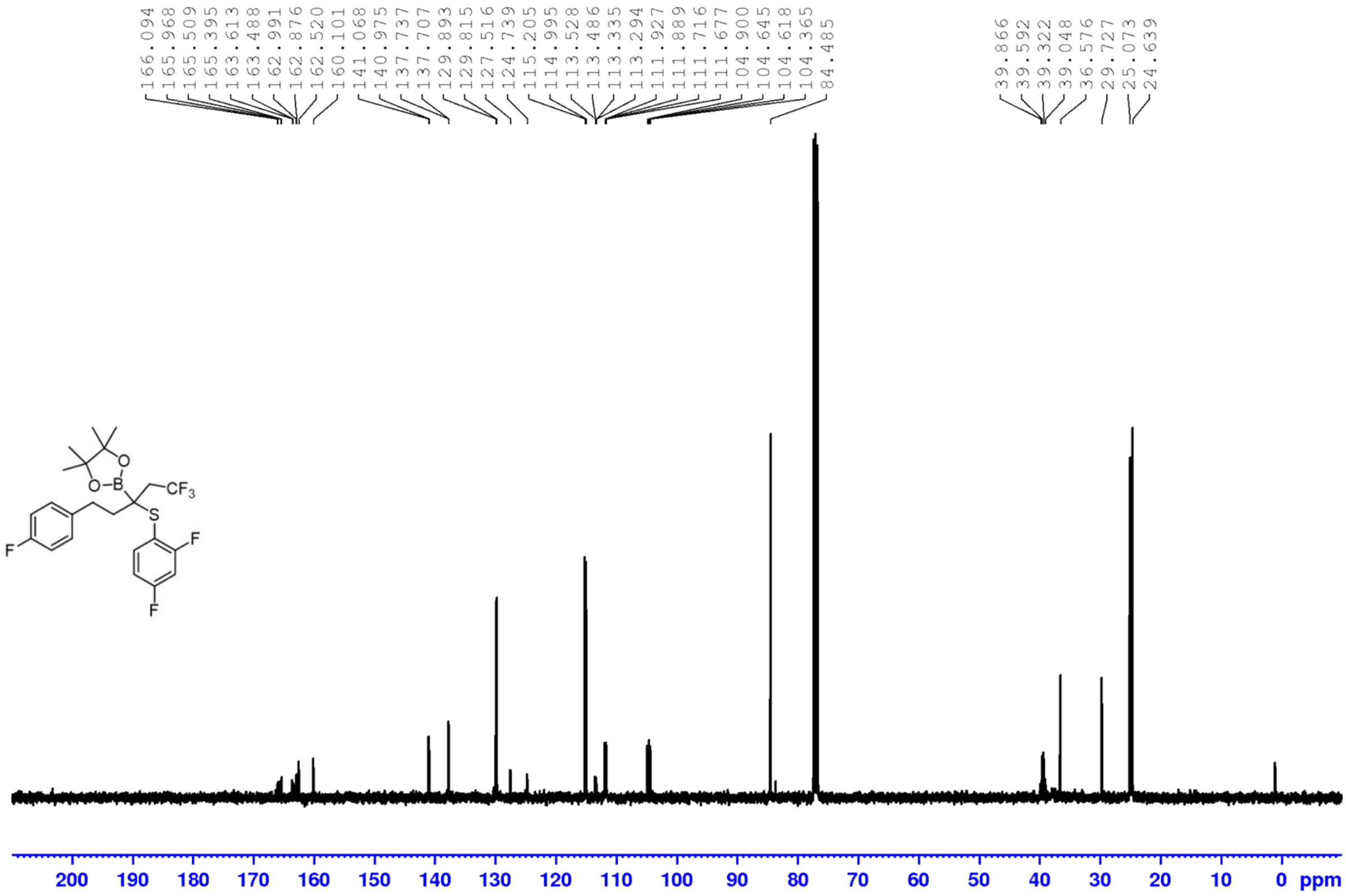

^{11}B NMR (128 MHz, Chloroform-*d*)

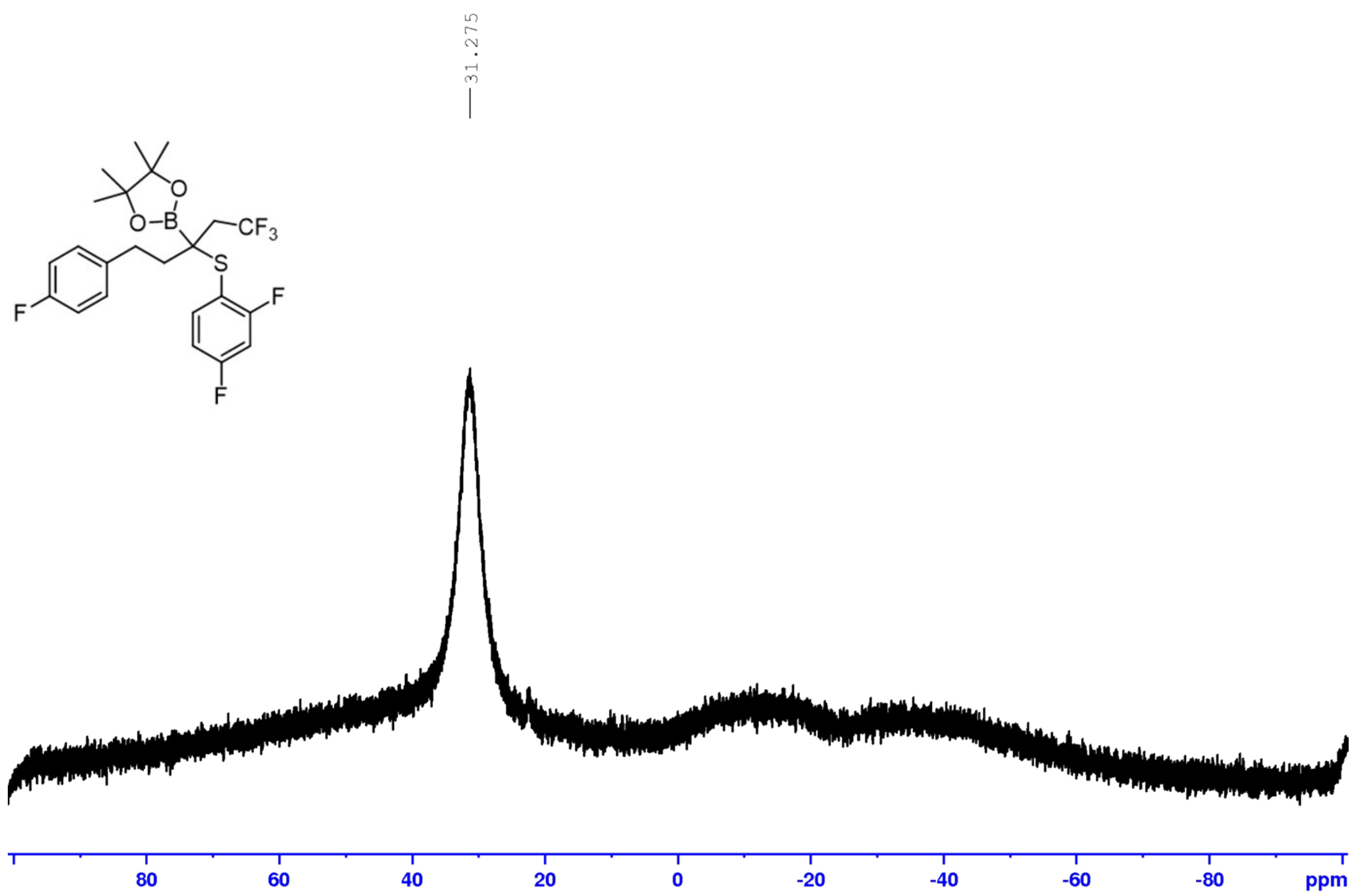

^{19}F NMR (376 MHz, Chloroform-*d*)

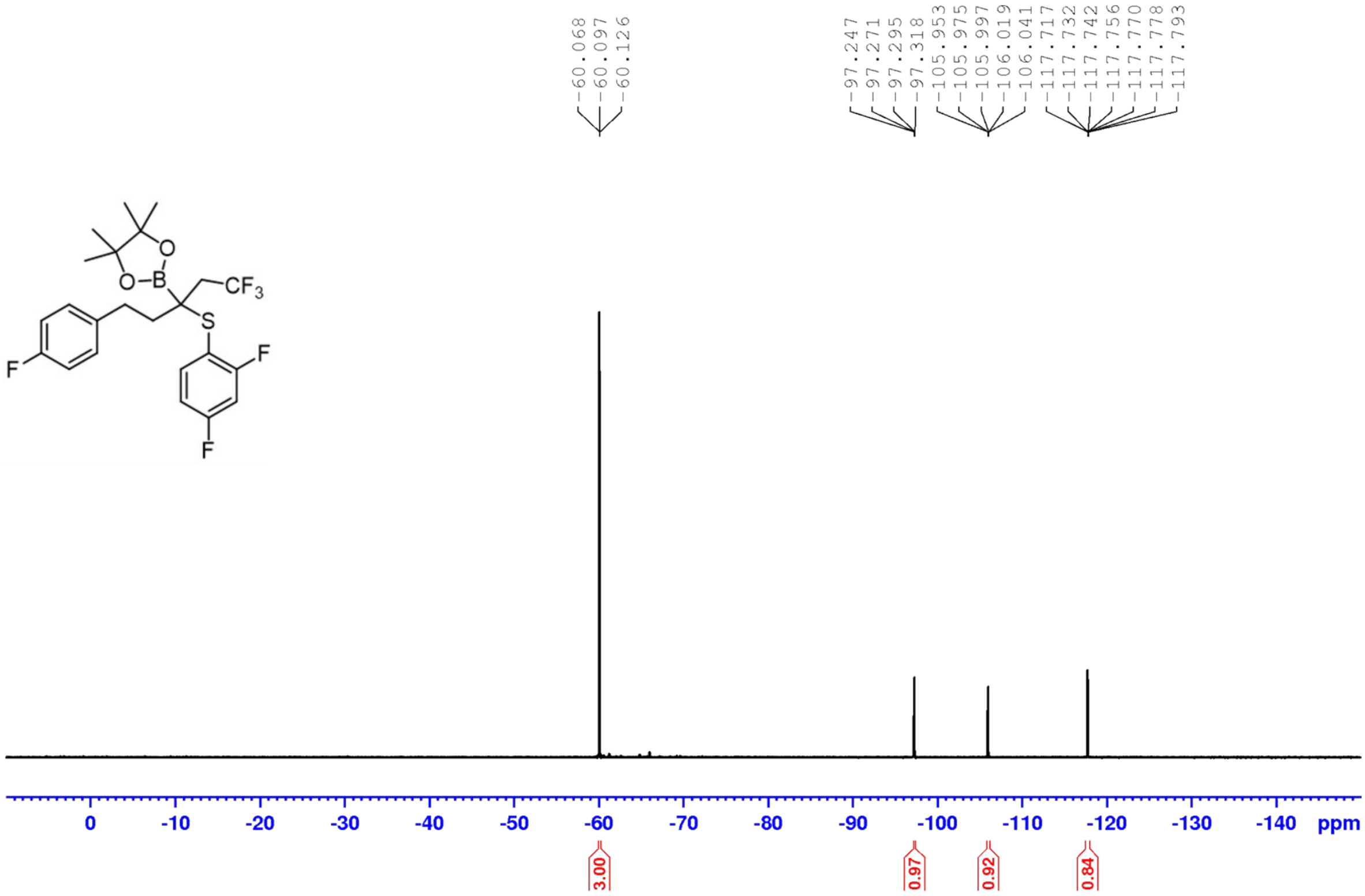

4,4,5,5-tetramethyl-2-(1,1,1-trifluoro-3-(thiophen-2-ylthio)-5-(p-tolyl)pentan-3-yl)-1,3,2-dioxaborolane (5d)

^{1}H NMR (400 MHz, Chloroform-*d*)

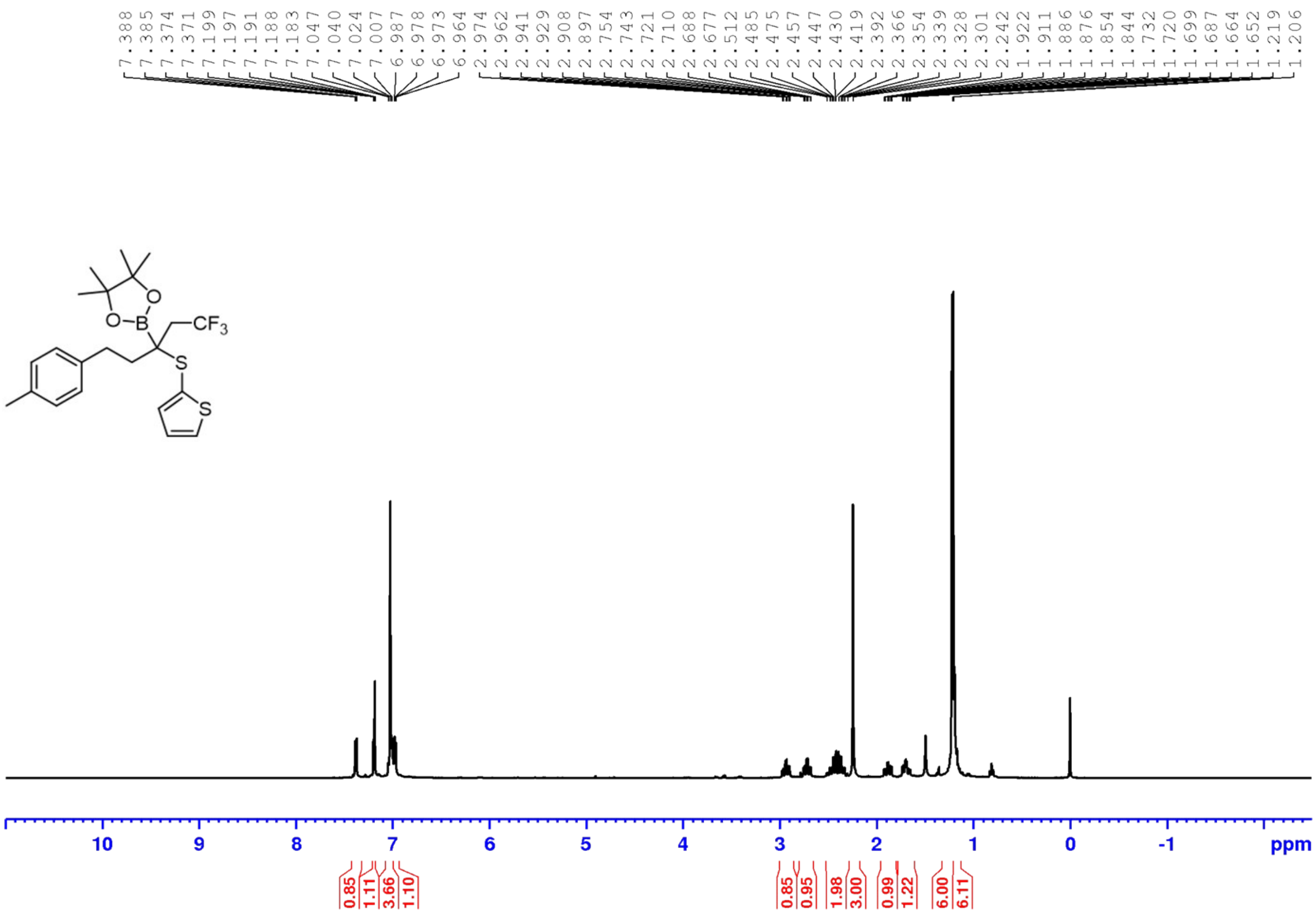

^{13}C NMR (125 MHz, Chloroform-*d*)

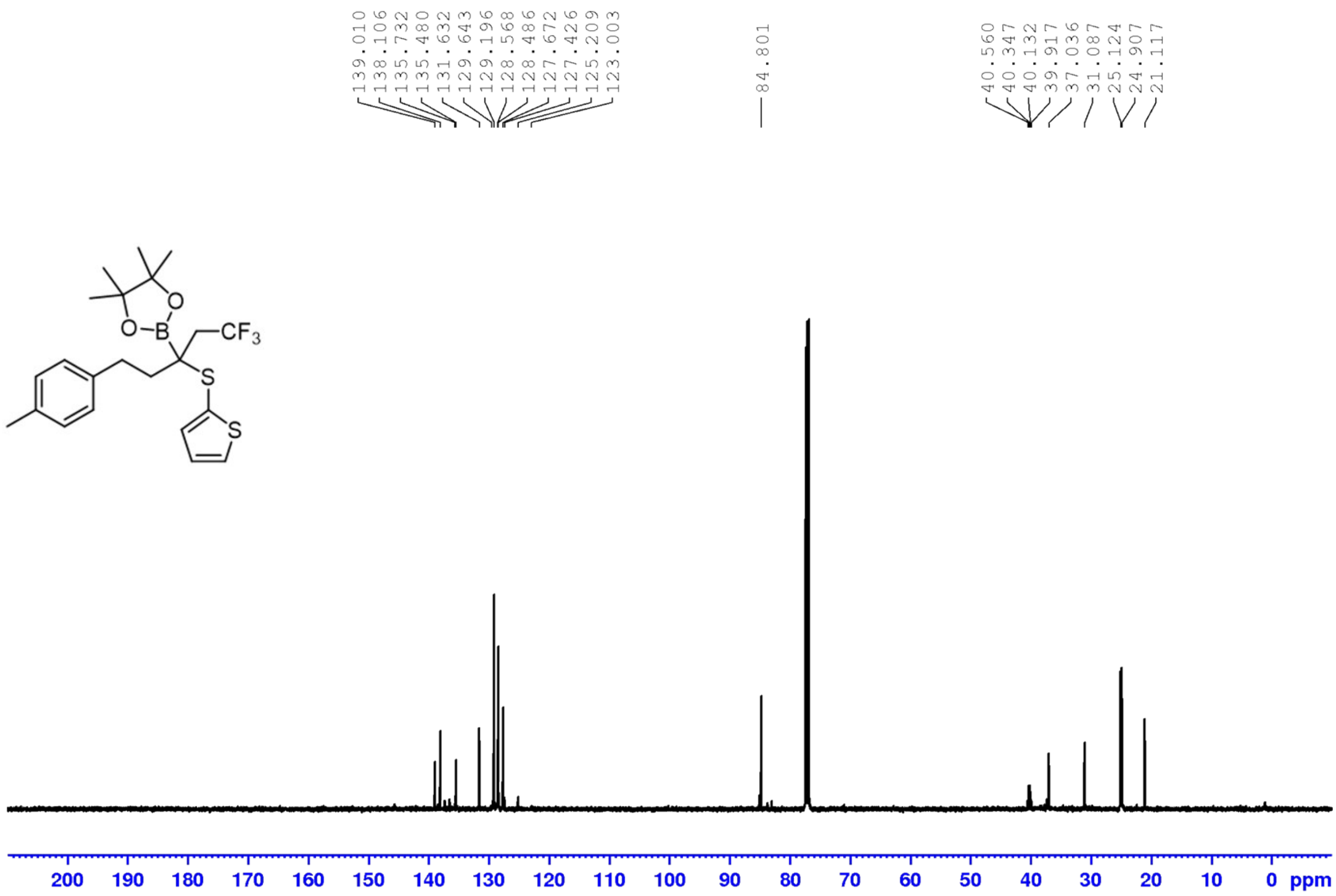

^{11}B NMR (128 MHz, Chloroform-*d*)

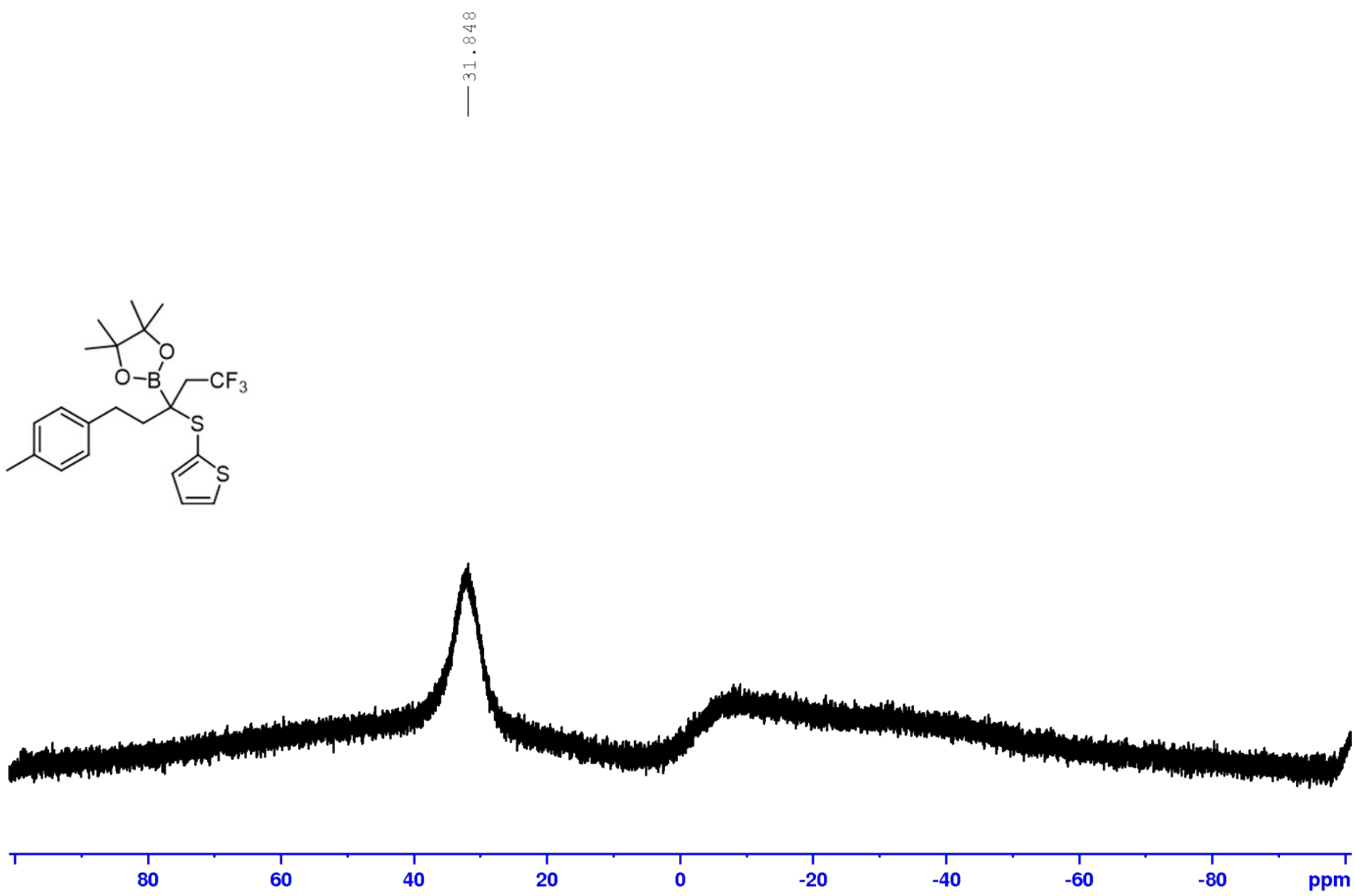

^{19}F NMR (376 MHz, Chloroform-*d*)

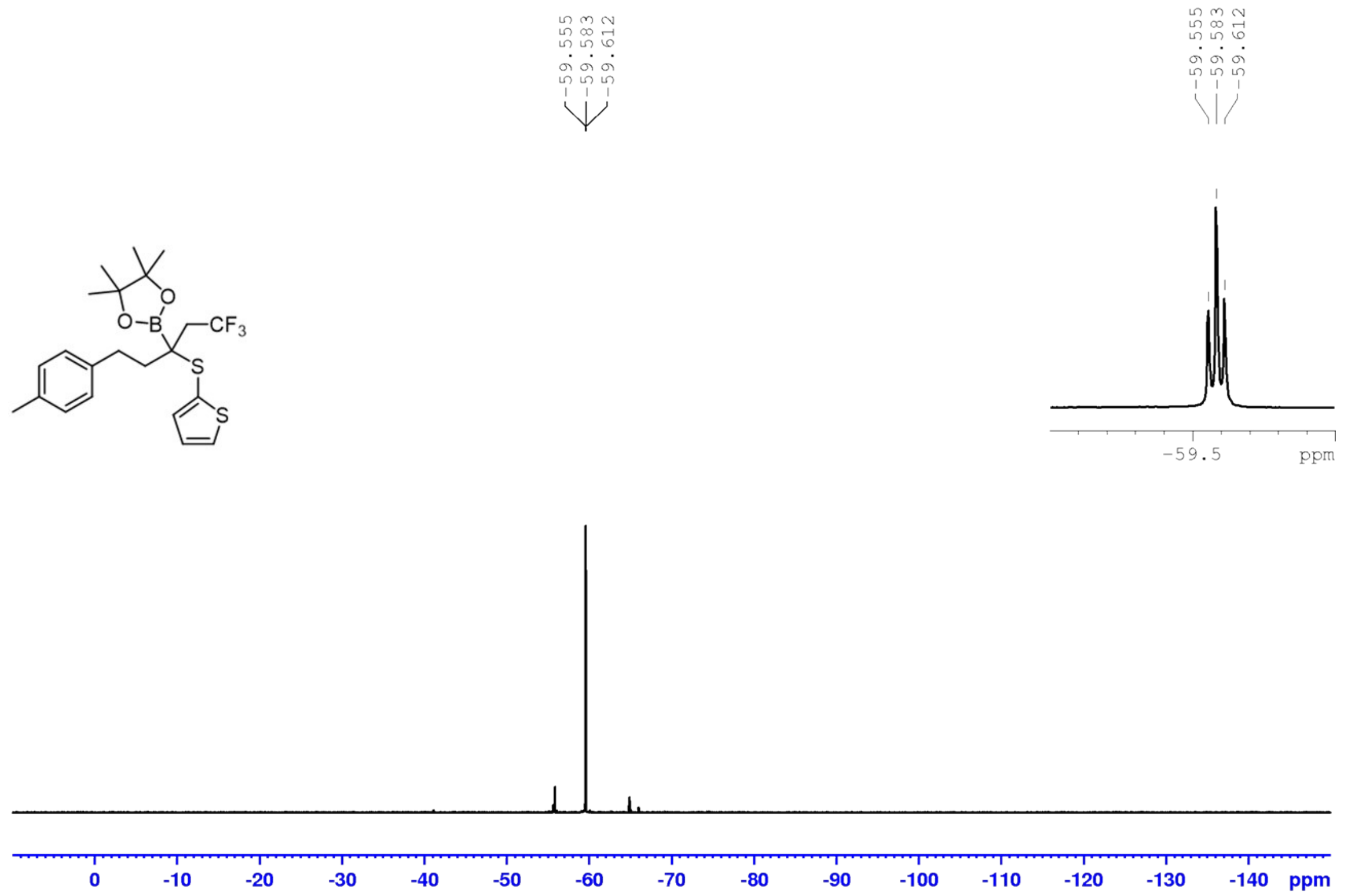

4,4,5,5-tetramethyl-2-(1,1,1-trifluoro-3-((2-methylfuran-3-yl)thio)-5-(p-tolyl)pentan-3-yl)-1,3,2-dioxaborolane (5e)

^{1}H NMR (400 MHz, Chloroform-*d*)

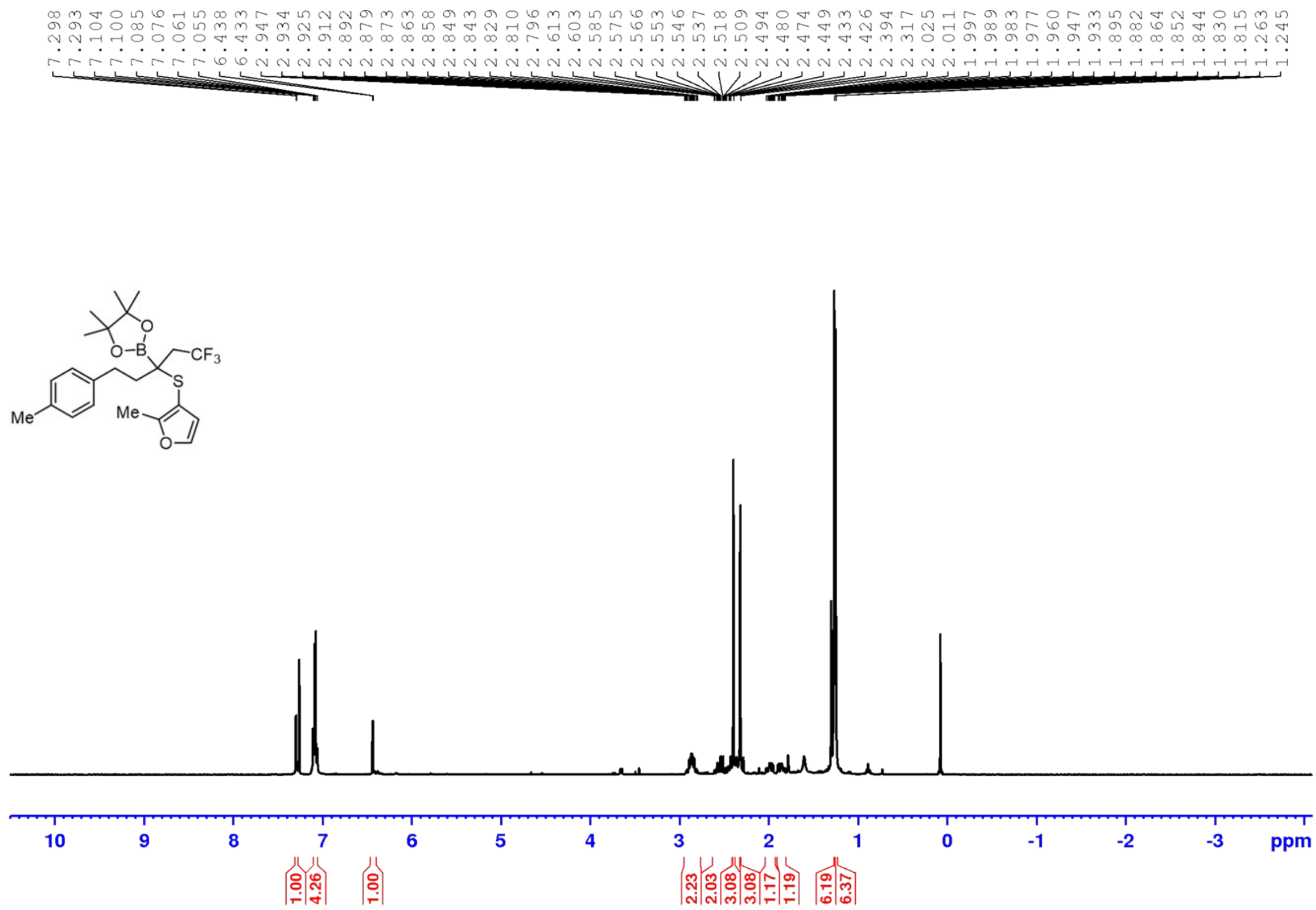

^{13}C NMR (100 MHz, Chloroform-*d*)

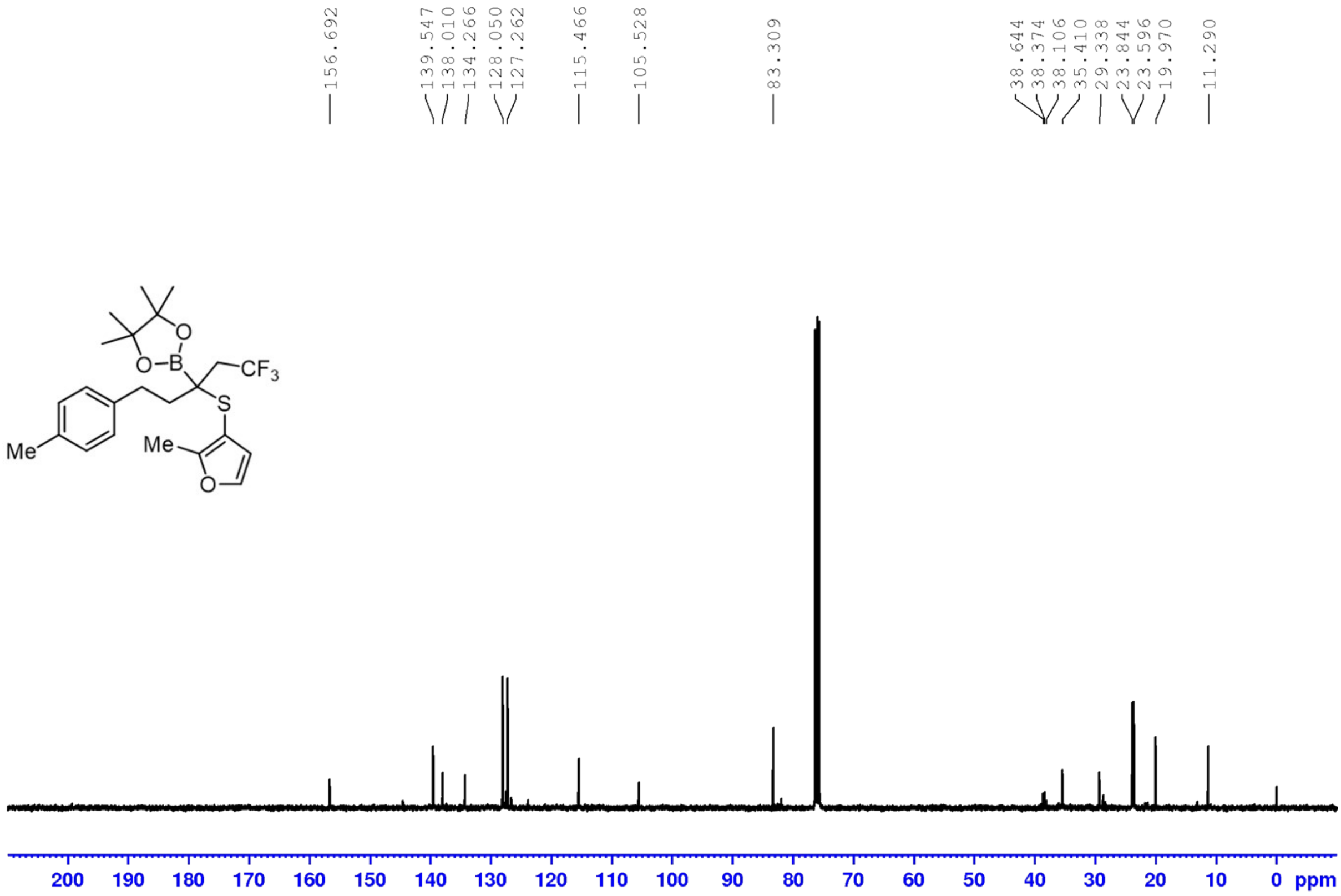

^{11}B NMR (128 MHz, Chloroform-*d*)

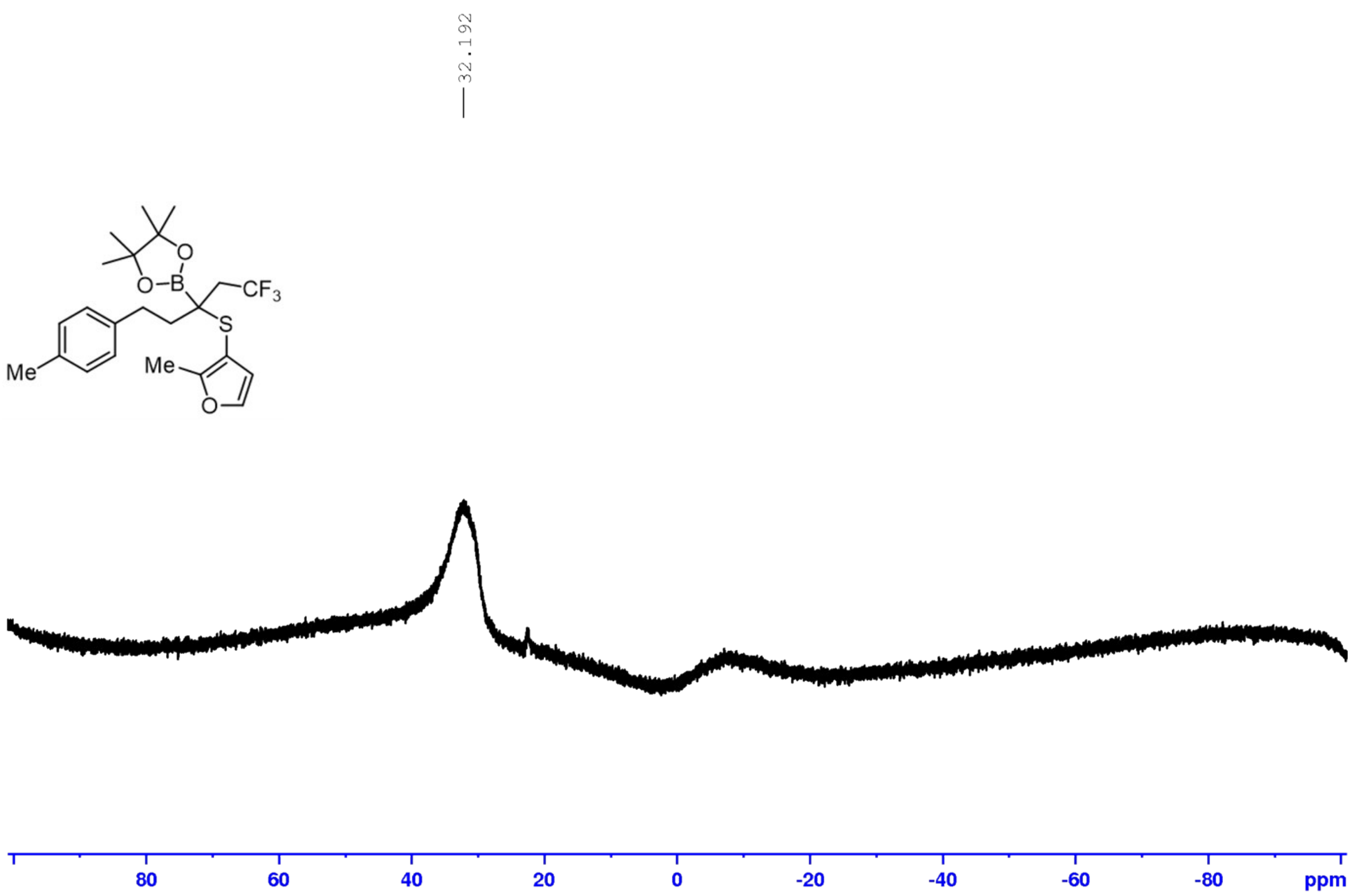

^{19}F NMR (376 MHz, Chloroform-*d*)

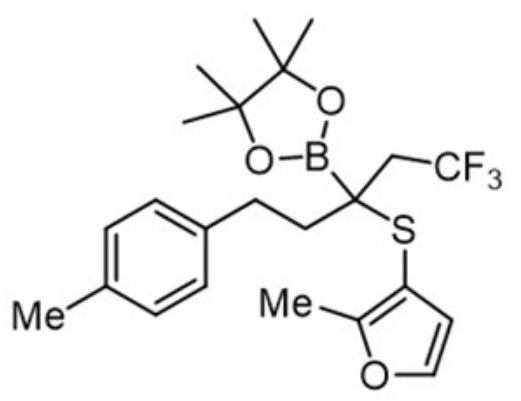

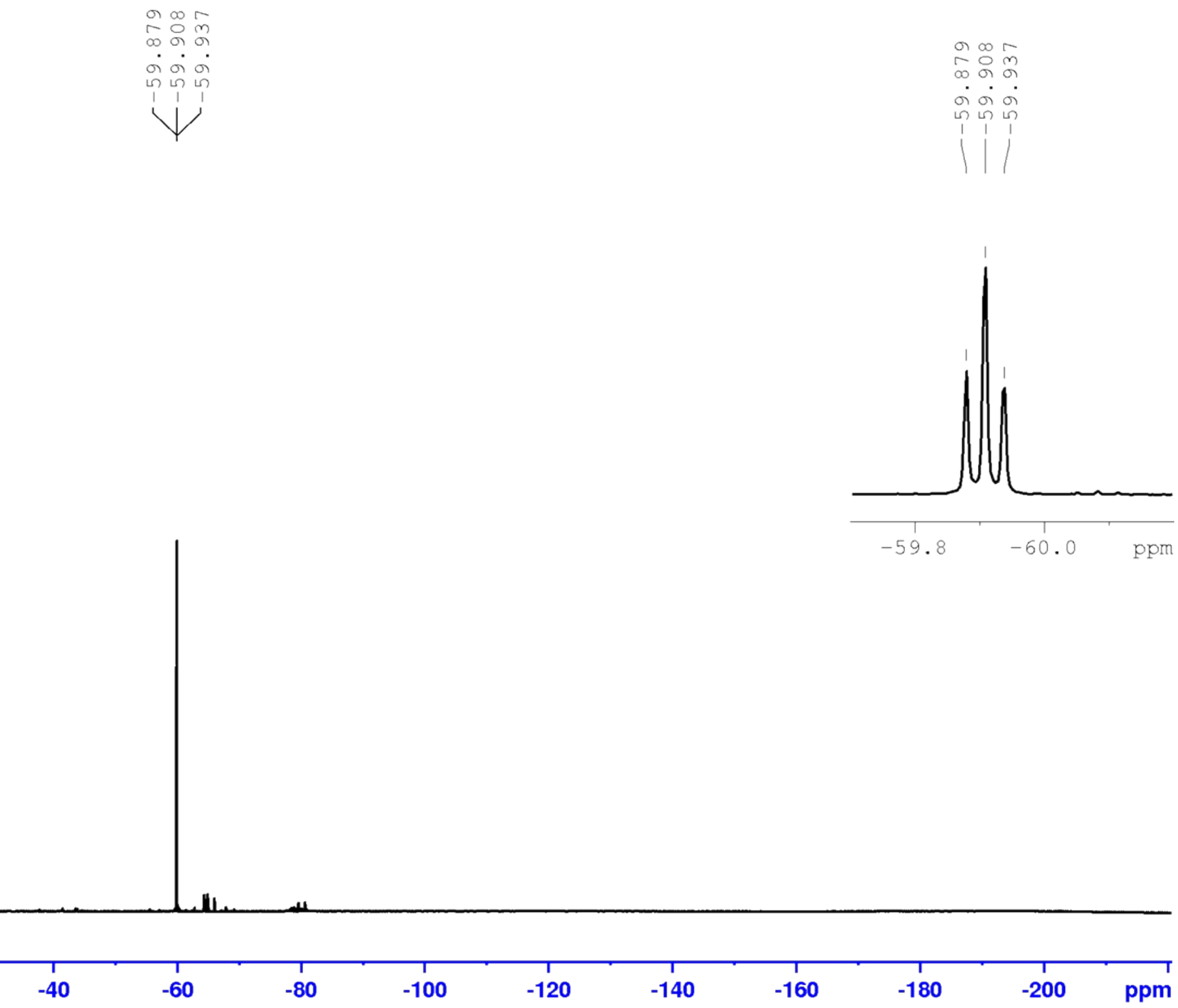

2-((4,4,4-trifluoro-2-(4,4,5,5-tetramethyl-1,3,2-dioxaborolan-2-yl)butan-2-yl)thio)-1,3,4-thiadiazole (5f)

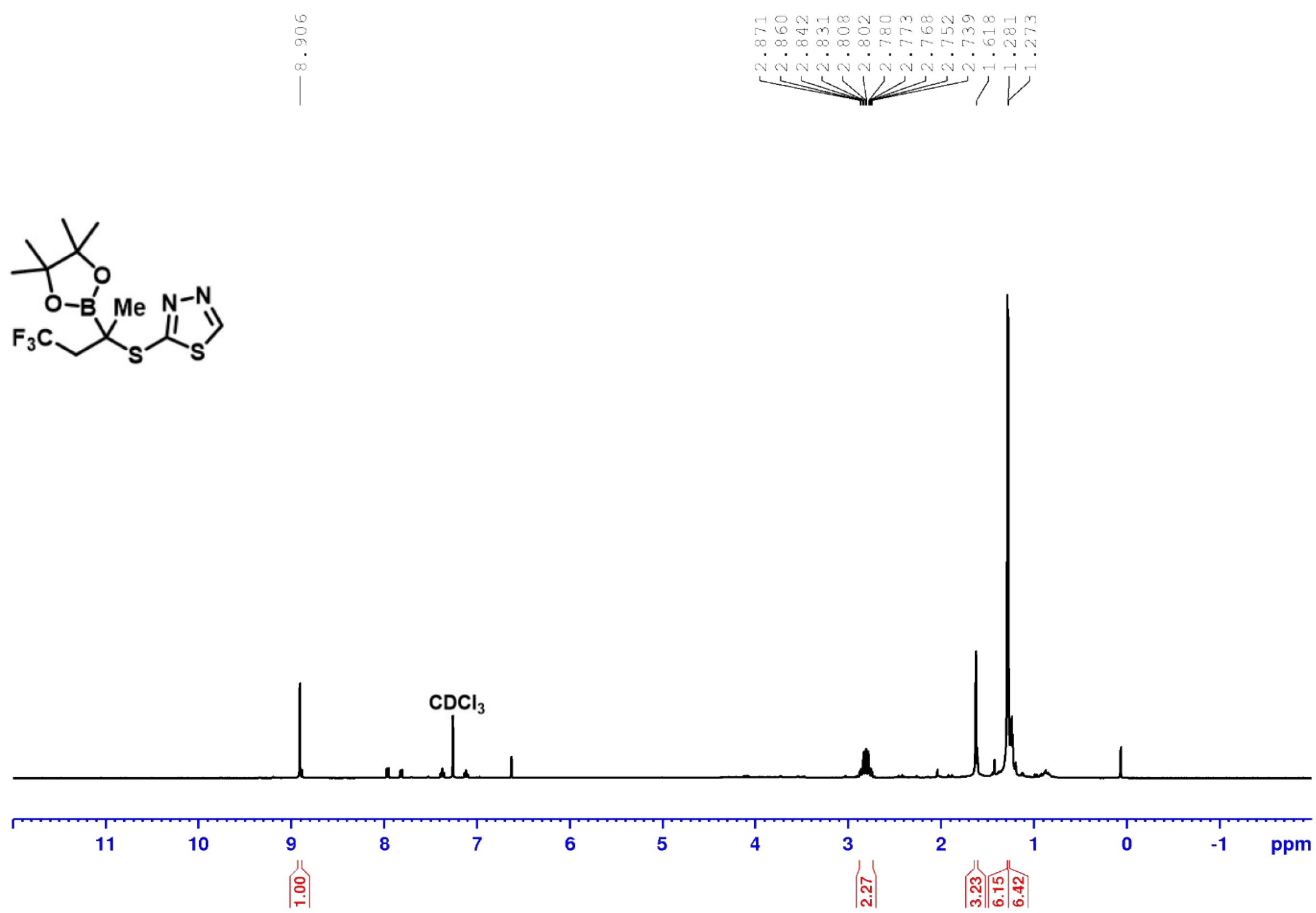

^{1}H NMR (400 MHz, Chloroform-*d*)

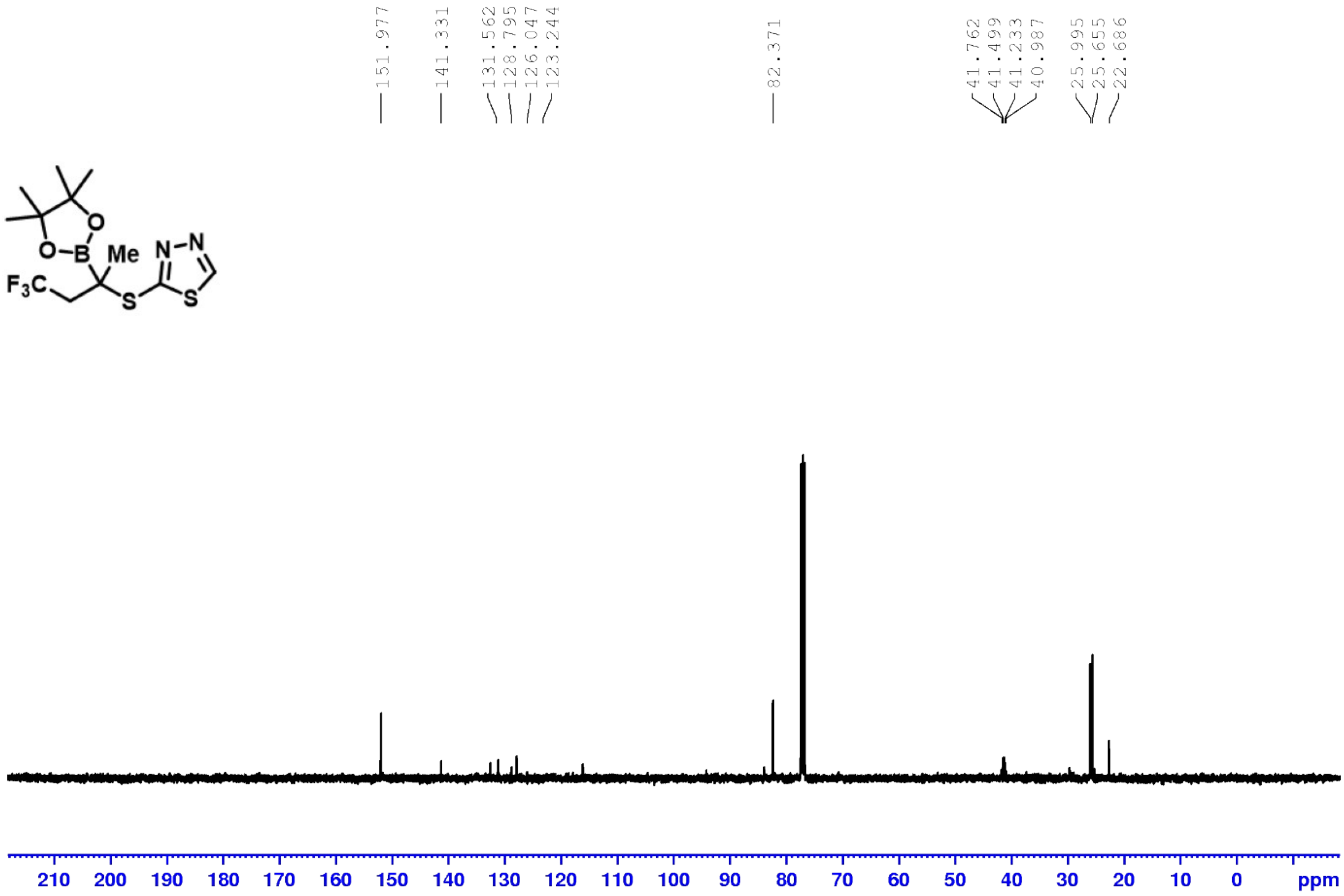

^{13}C NMR (100 MHz, Chloroform-*d*)

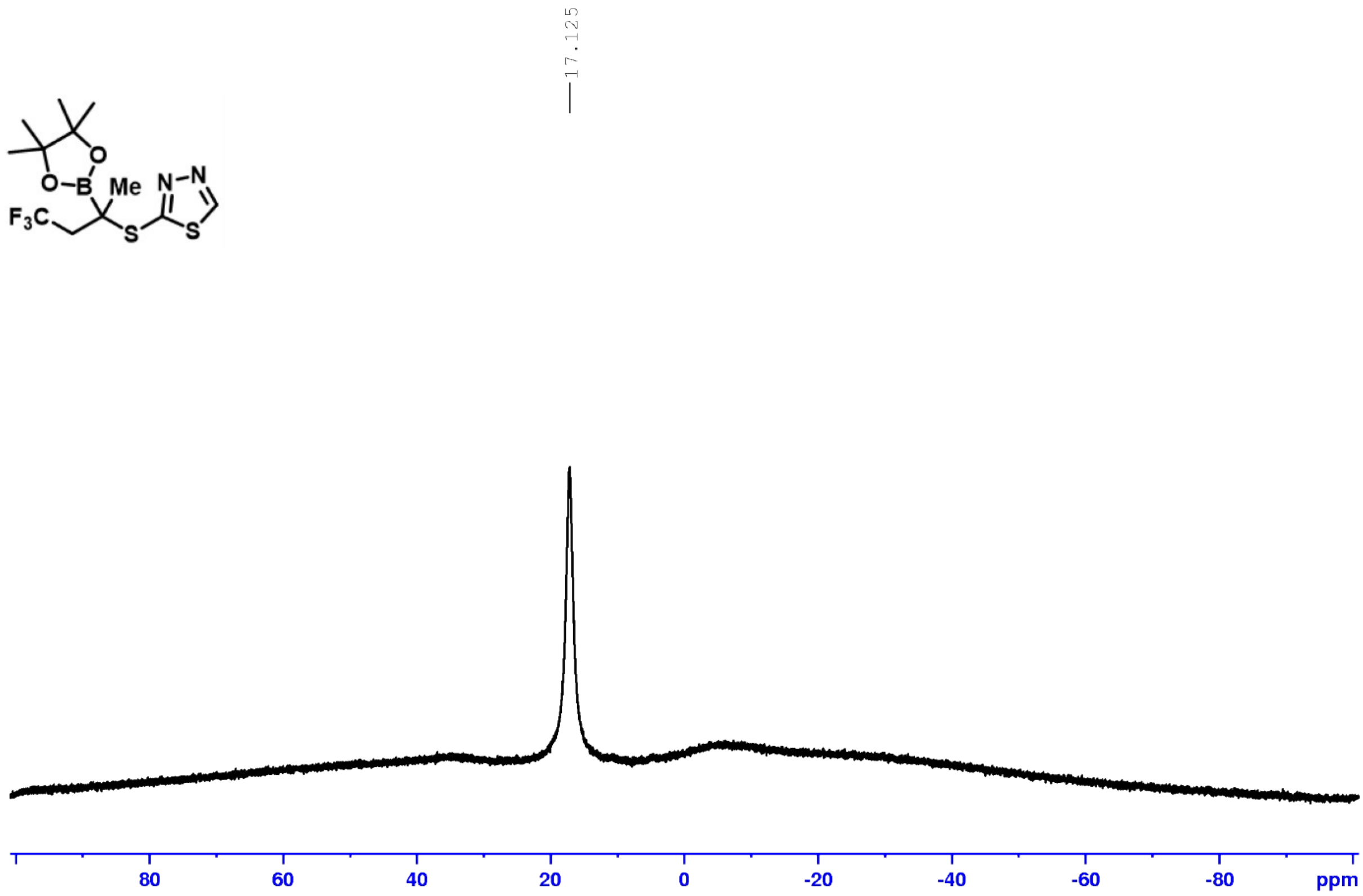

^{11}**B (128 MHz, Chloroform-*d*)**

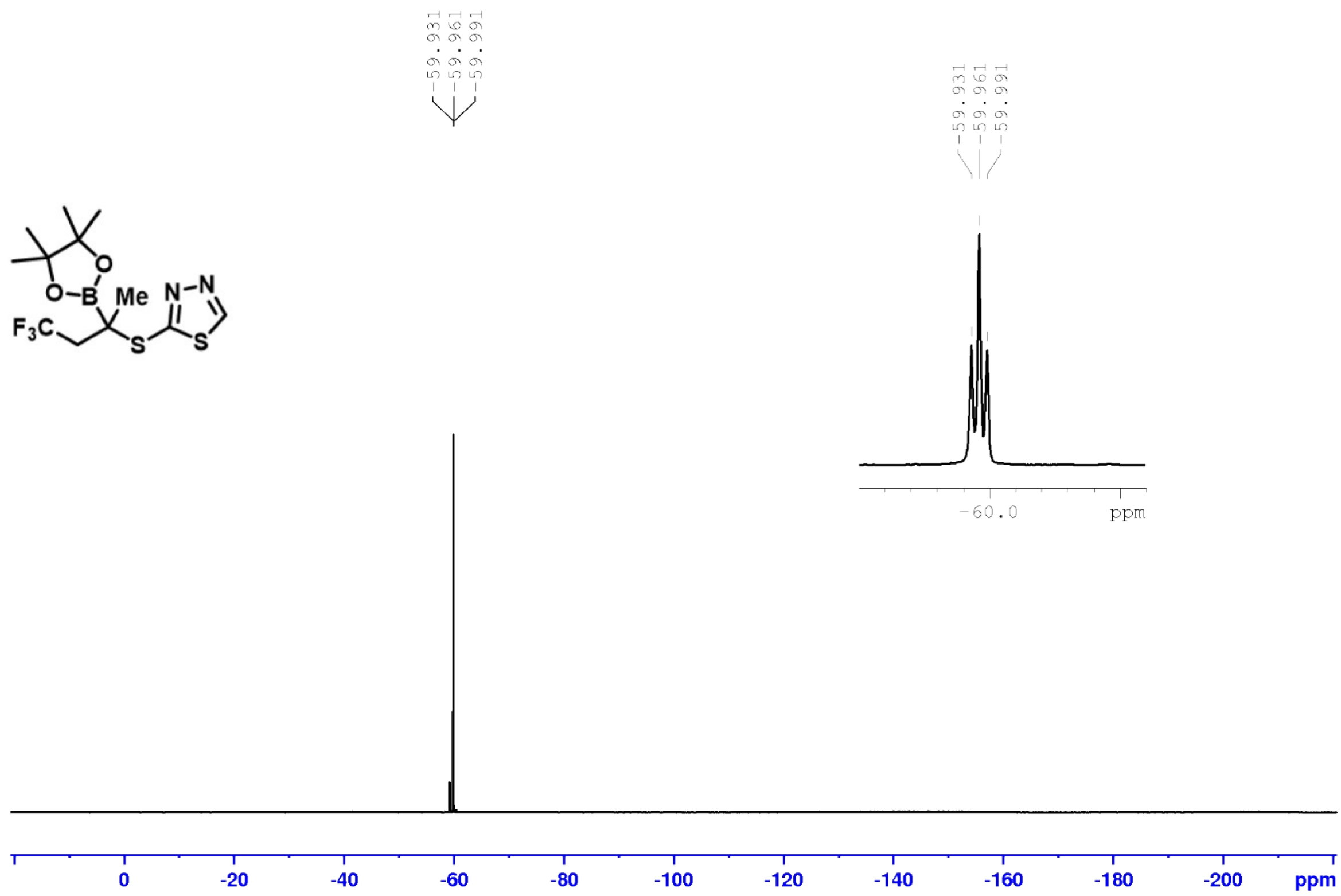

^{19}**F NMR (376 MHz, Chloroform-*d*)**

4-((4,4,4-trifluoro-2-(4,4,5,5-tetramethyl-1,3,2-dioxaborolan-2-yl)butan-2-yl)thio)pyridine (5g)

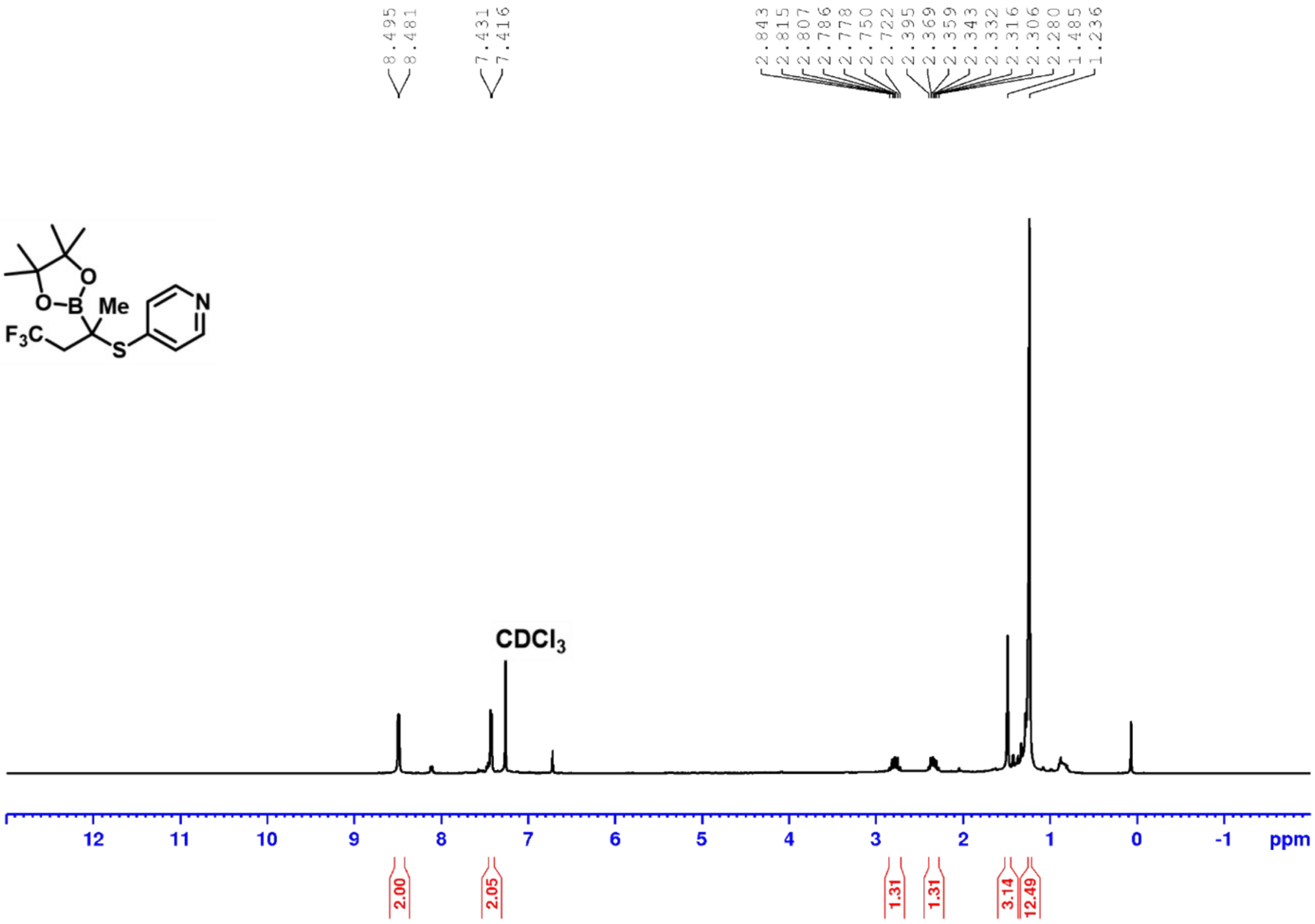

^{1}H NMR (400 MHz, Chloroform-*d*)

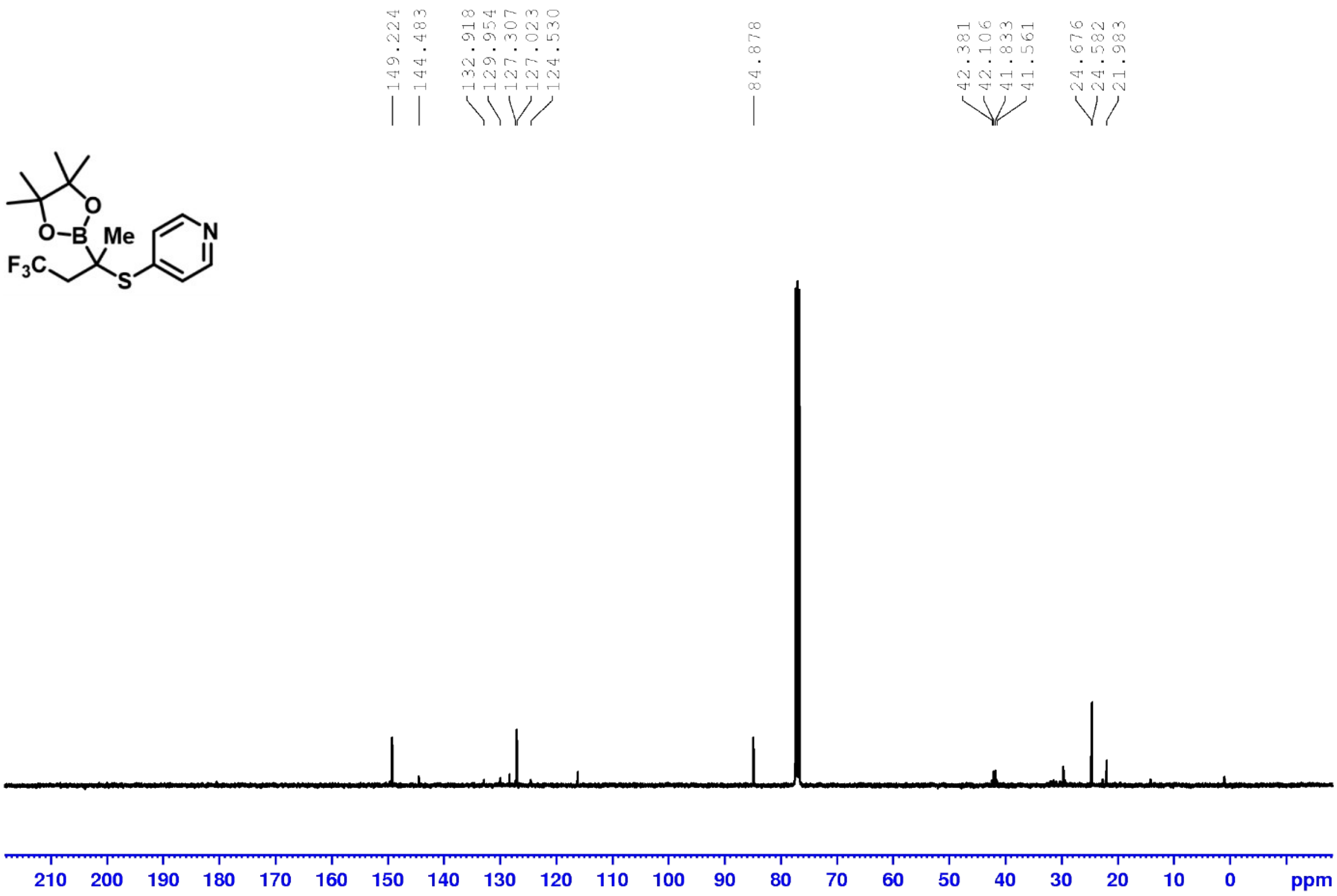

^{13}C NMR (100 MHz, Chloroform-*d*)

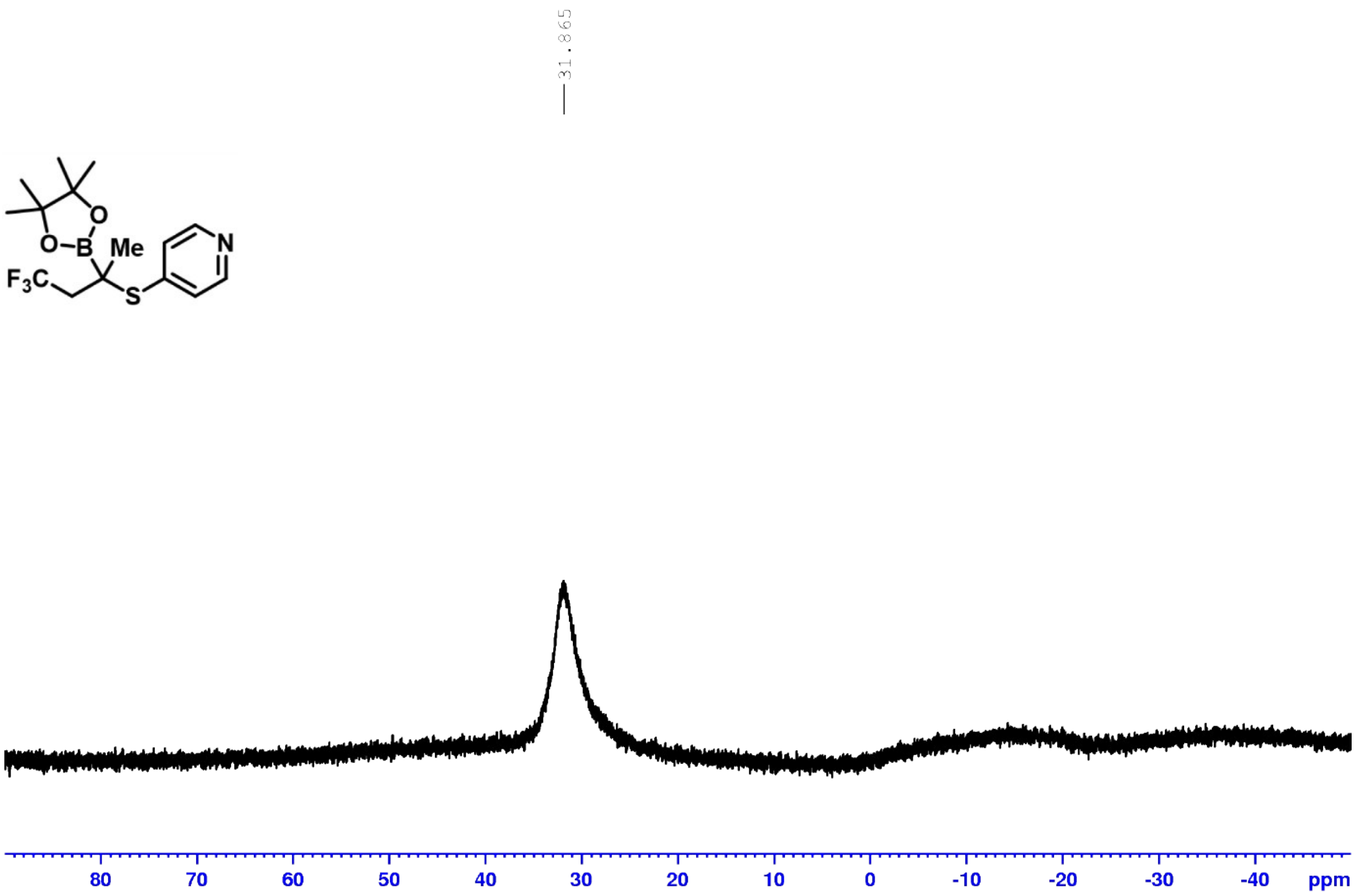

^{11}**B (128 MHz, Chloroform-*d*)**

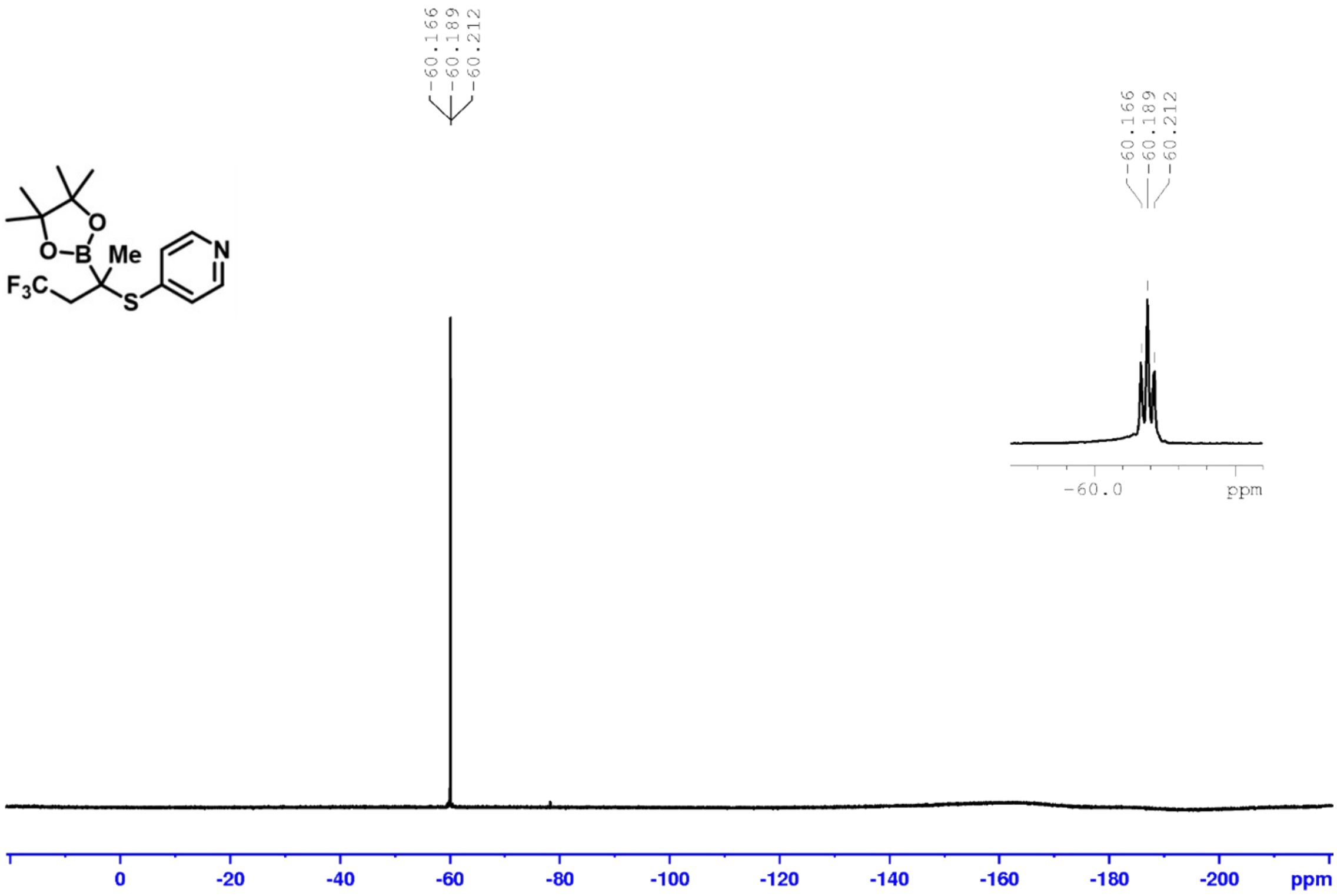

^{19}**F NMR (470 MHz, Chloroform-*d*)**

4,4,5,5-tetramethyl-2-(4,4,4-trifluoro-2-((4-methoxyphenyl)thio)butan-2-yl)-1,3,2-dioxaborolane (5h)

^{1}H NMR (400 MHz, Chloroform-*d*)

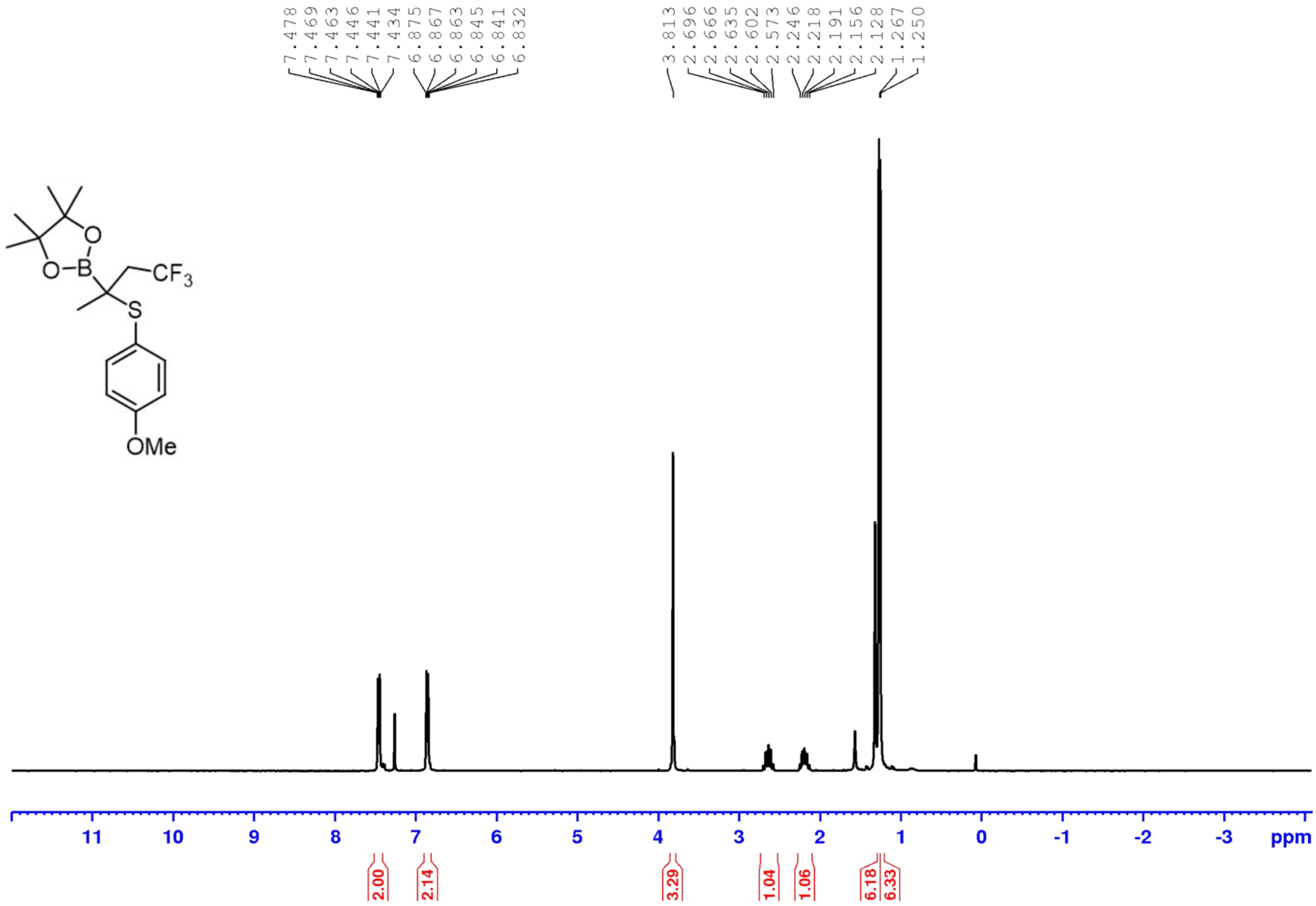

^{13}C NMR (125 MHz, Chloroform-*d*)

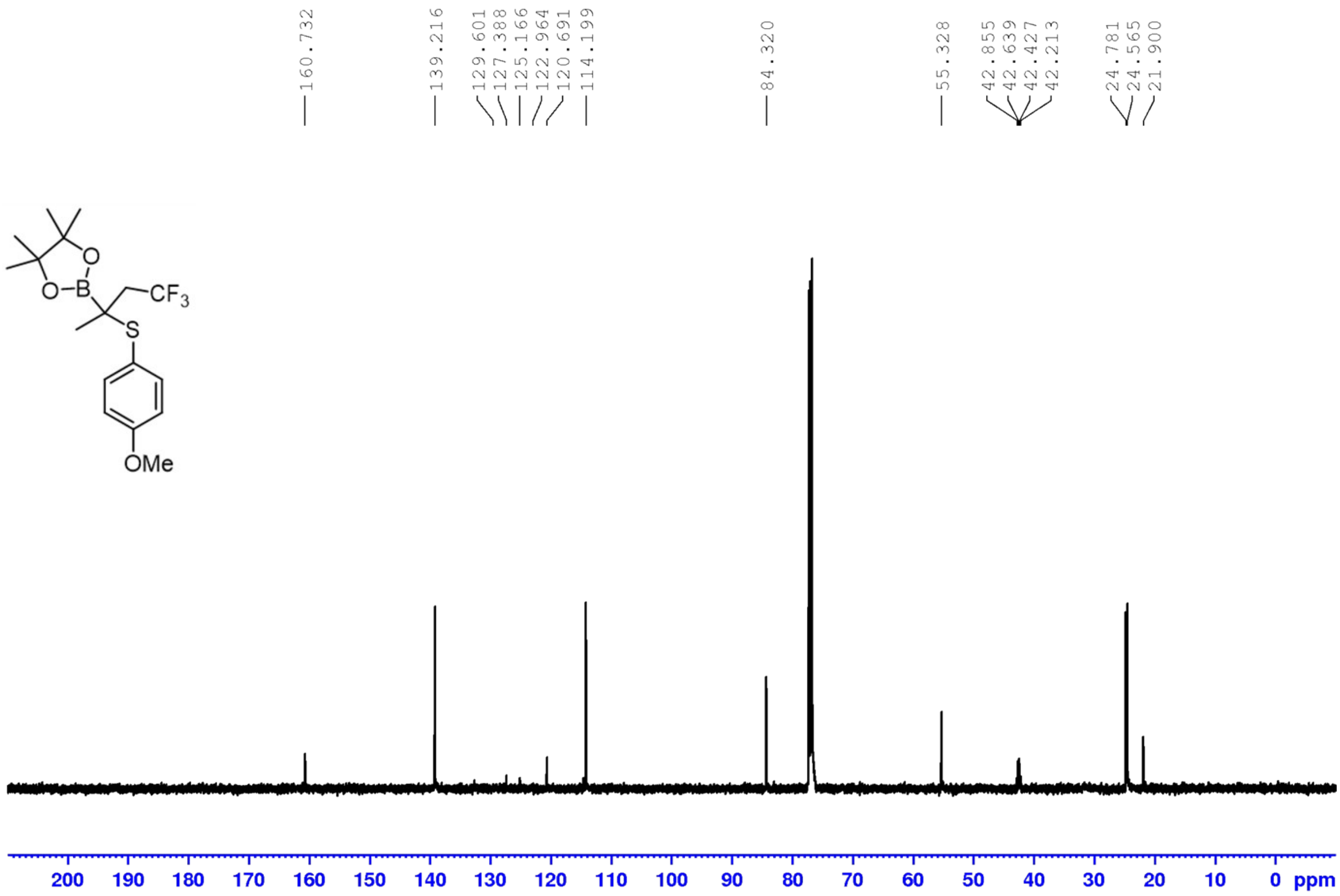

^{11}B NMR (128 MHz, Chloroform-*d*)

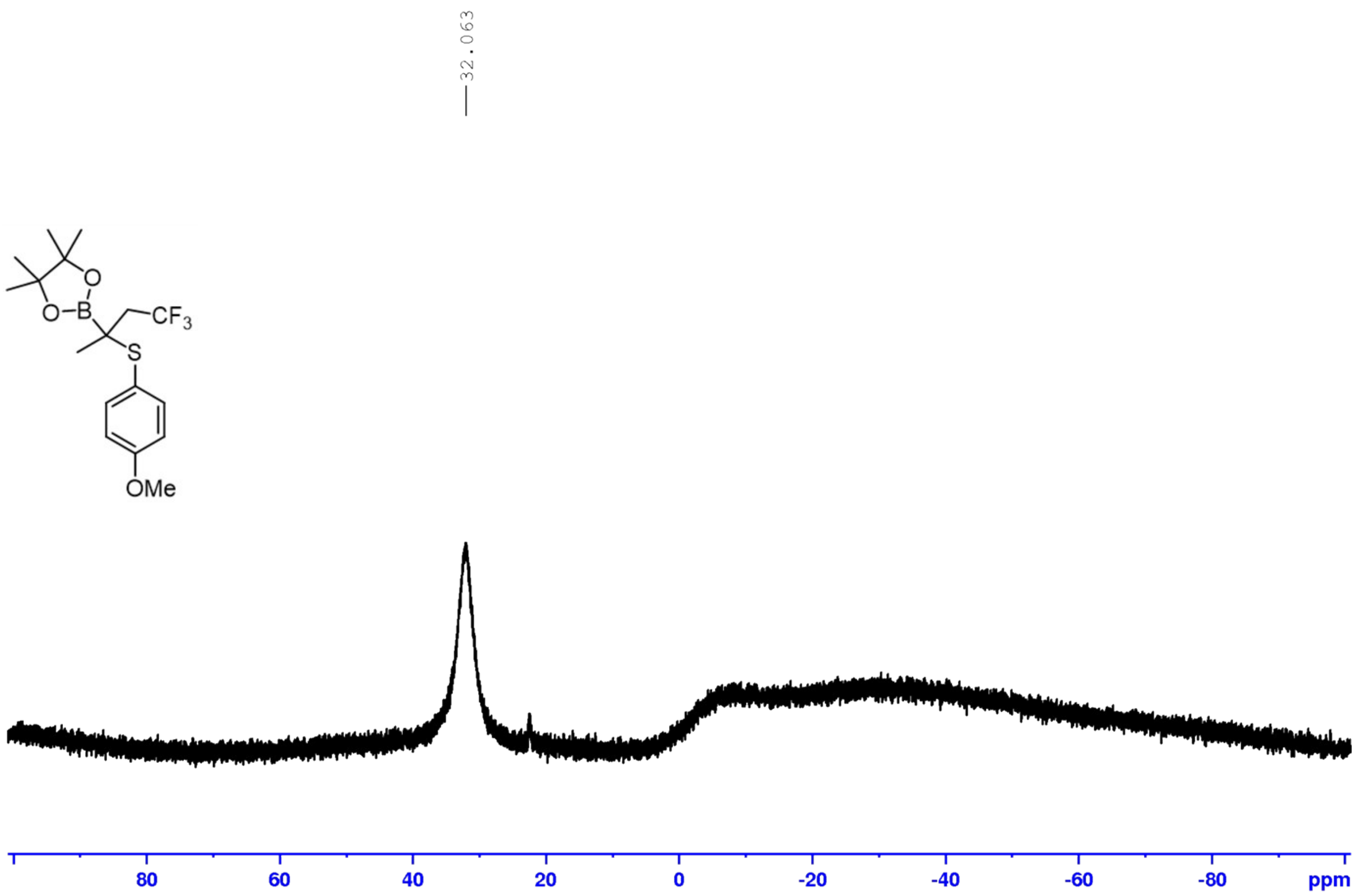

^{19}F NMR (376 MHz, Chloroform-*d*)

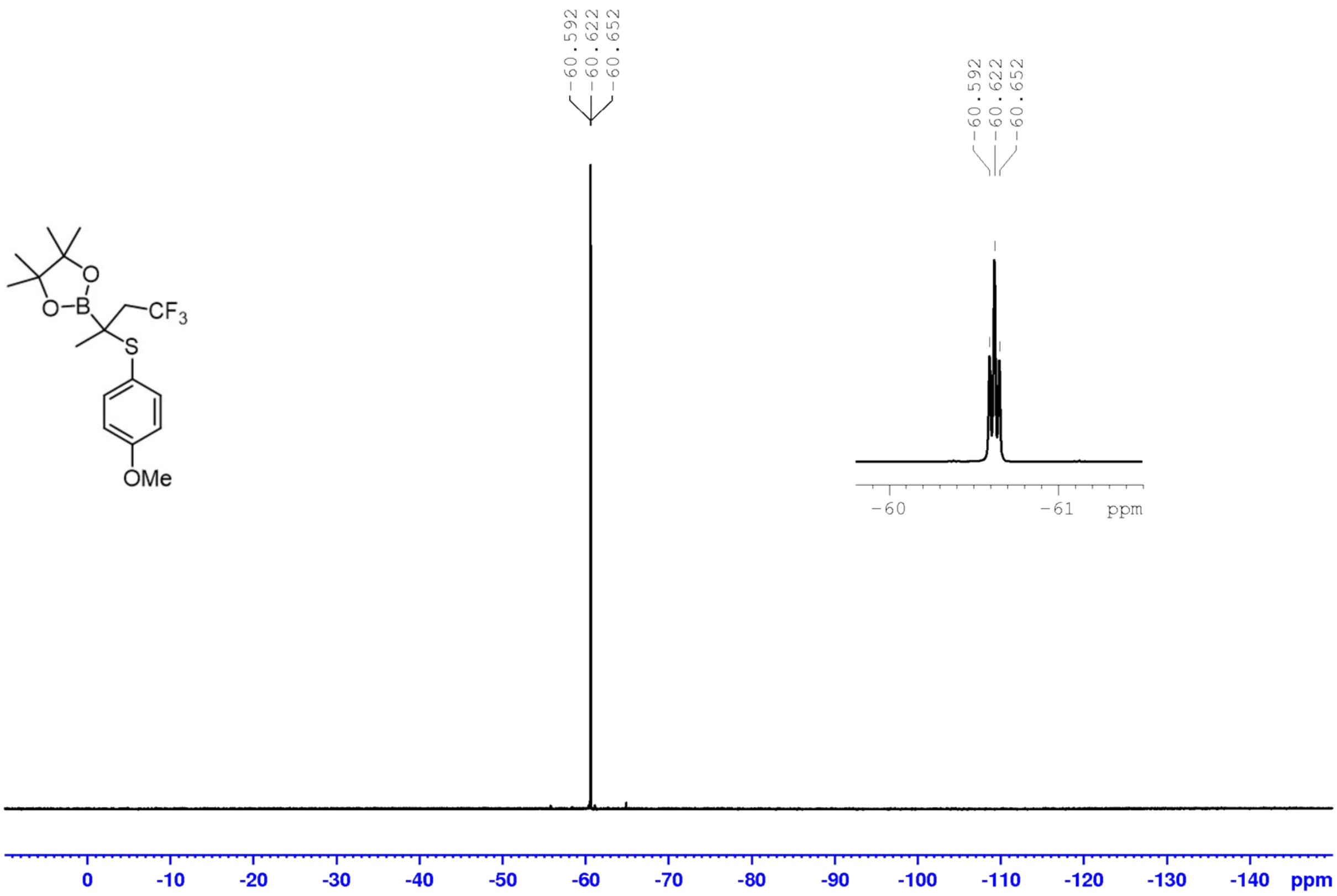

4,4,5,5-tetramethyl-2-(4,4,4-trifluoro-2-((2-methoxyphenyl)thio)butan-2-yl)-1,3,2-dioxaborolane (5i)

^{1}H NMR (400 MHz, Chloroform-*d*)

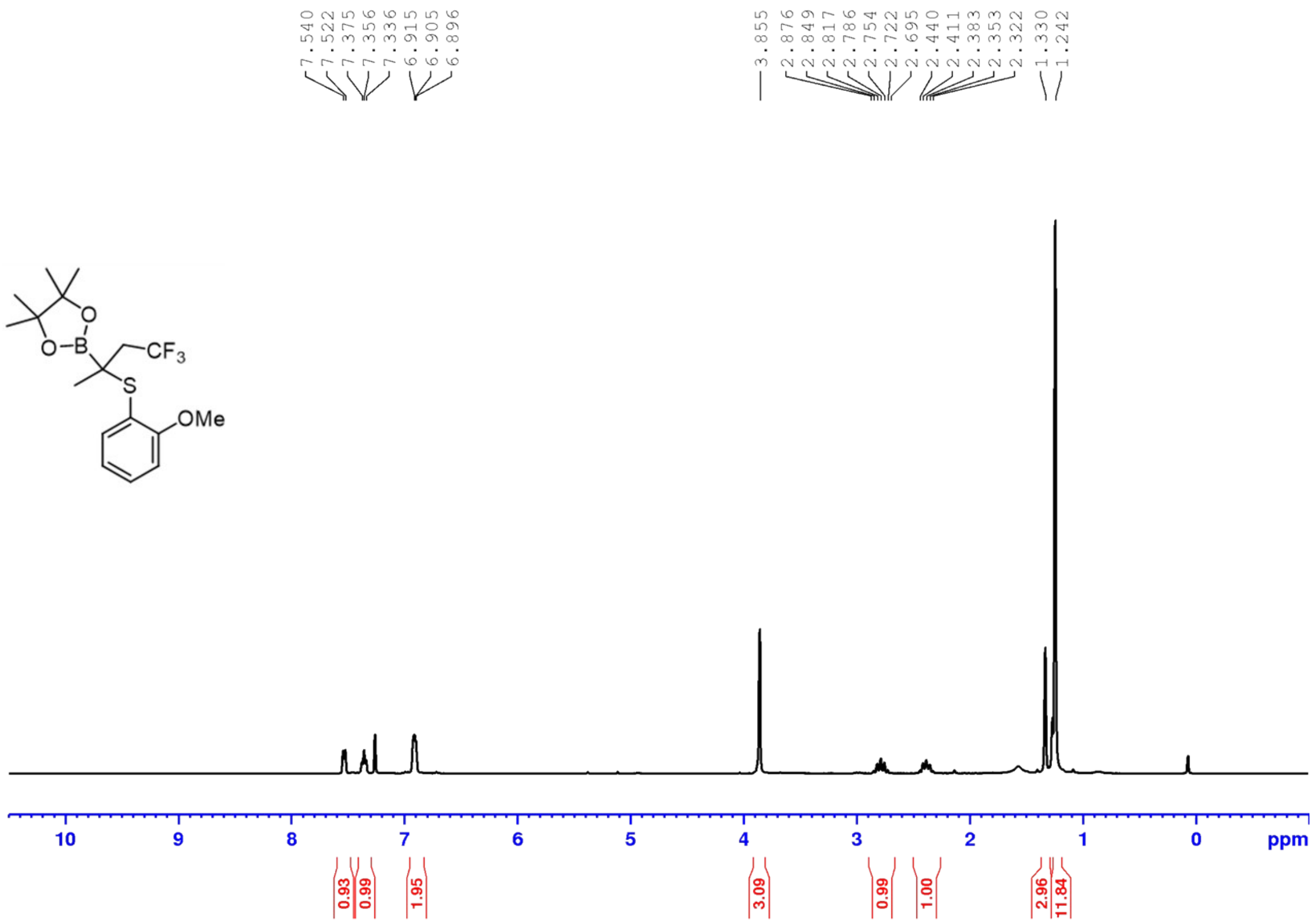

^{13}C NMR (100 MHz, Chloroform-*d*)

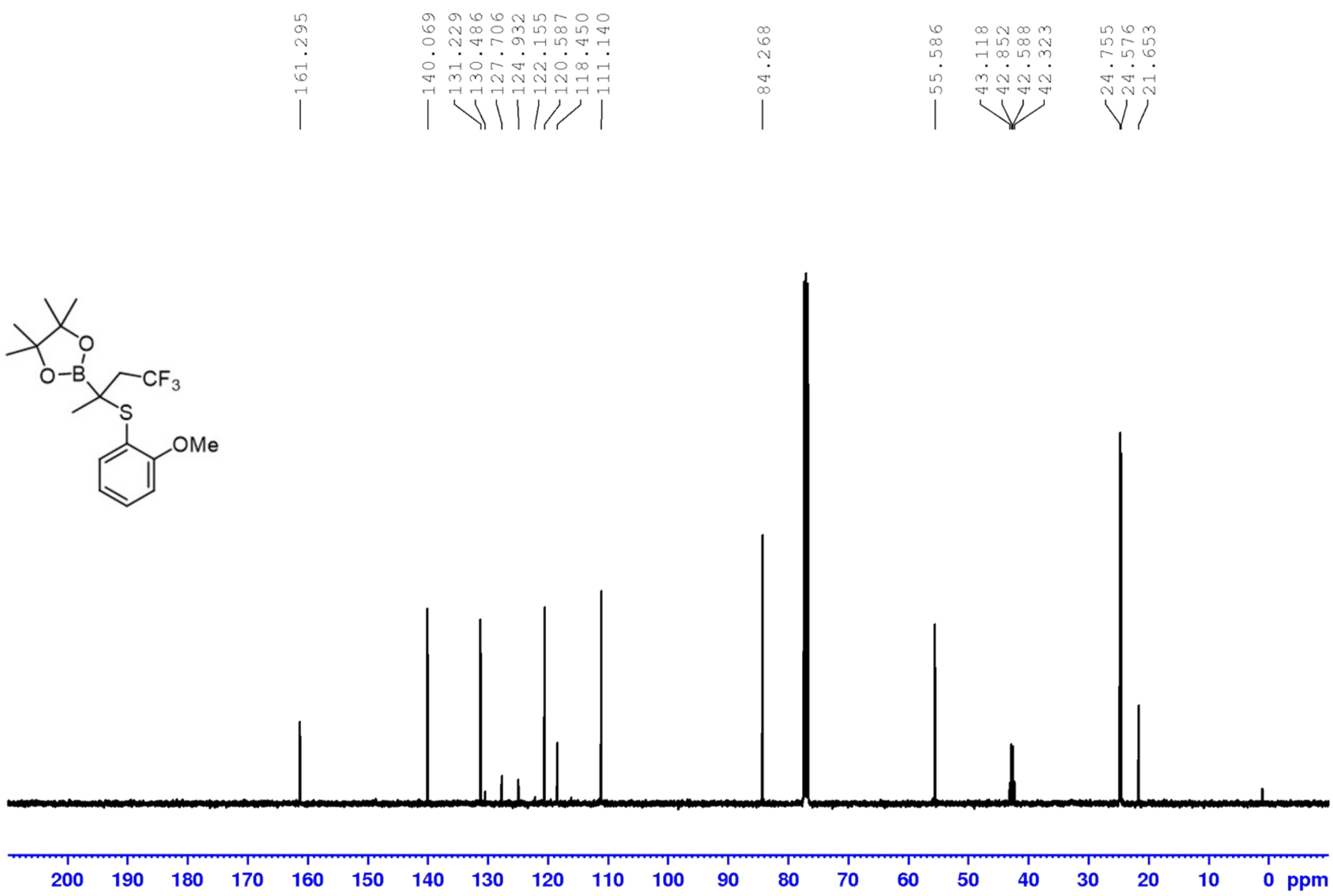

^{11}B NMR (128 MHz, Chloroform-*d*)

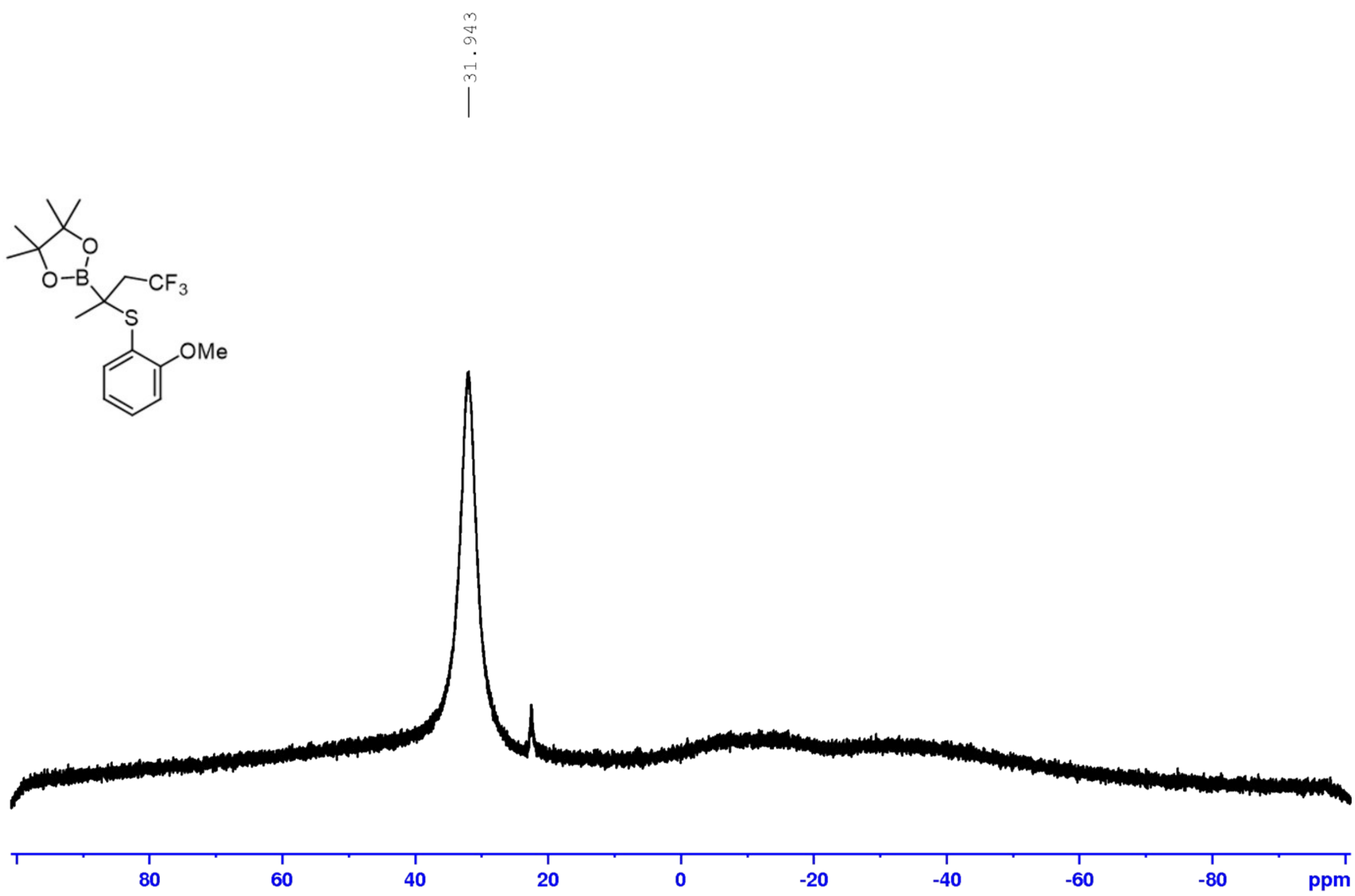

^{19}F NMR (376 MHz, Chloroform-*d*)

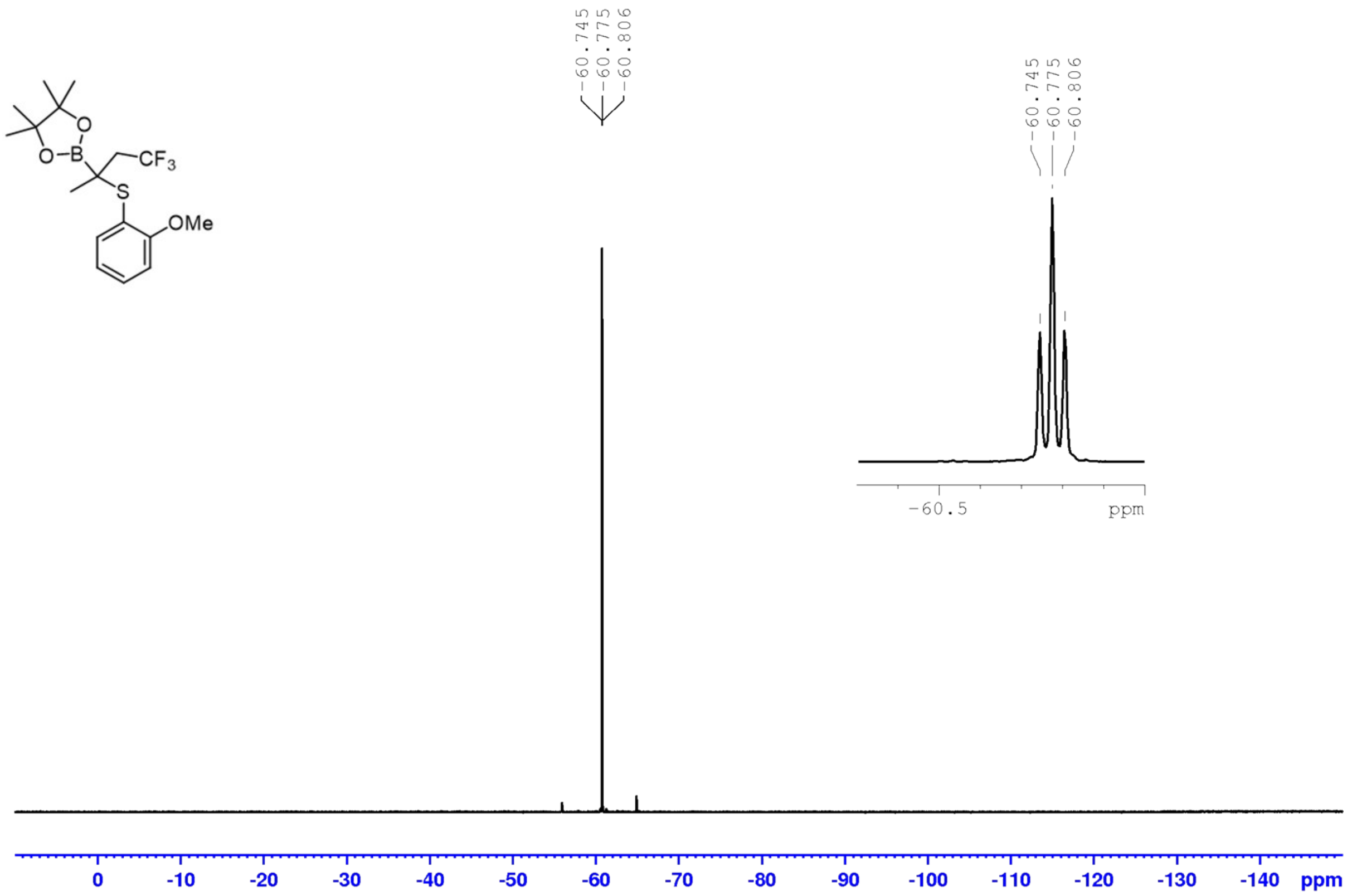

2-(2-((2,6-dichlorophenyl)thio)-4,4,4-trifluorobutan-2-yl)-4,4,5,5-tetramethyl-1,3,2-dioxaborolane (5j)

^{1}H NMR (400 MHz, Chloroform-*d*)

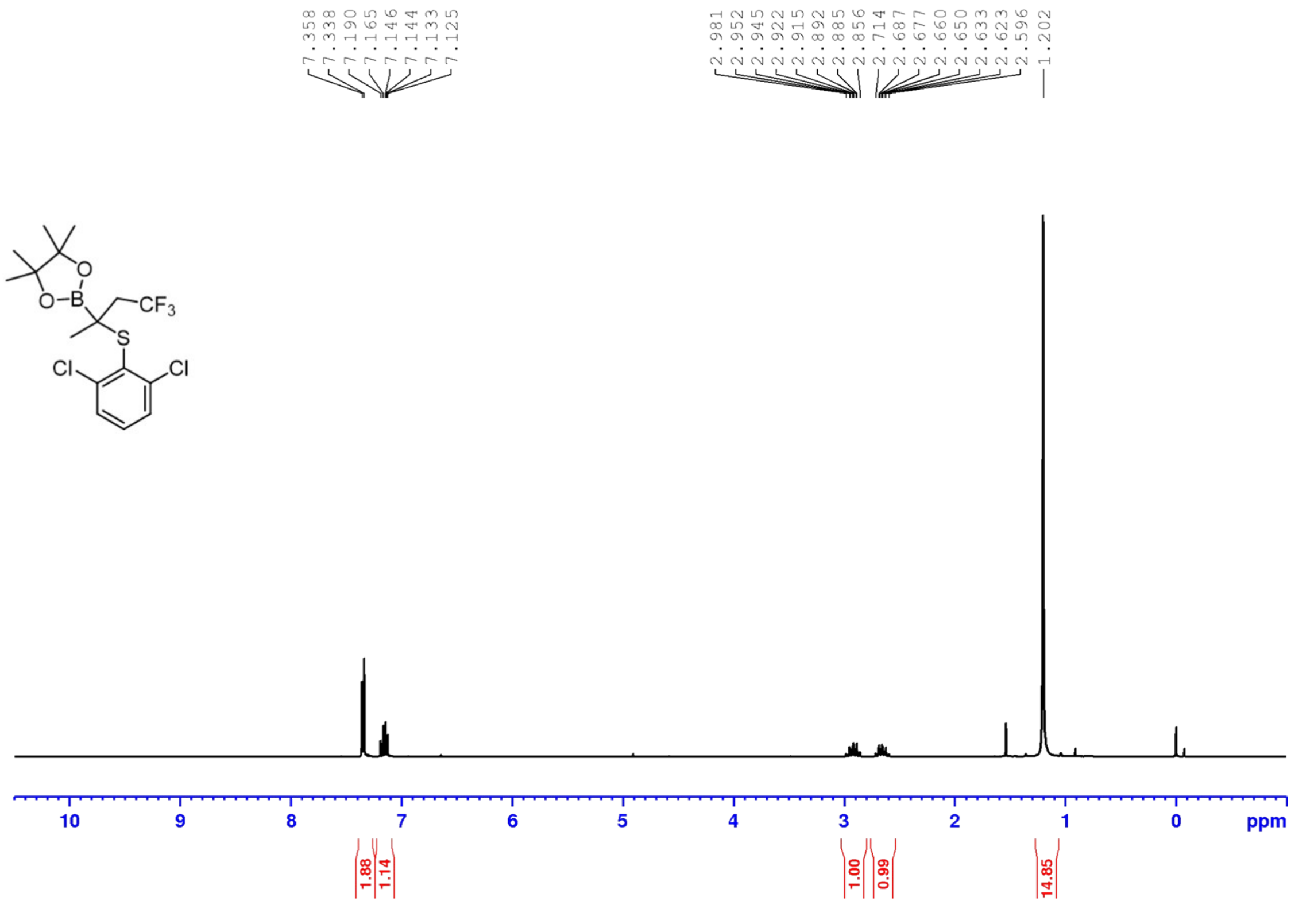

^{13}C NMR (125 MHz, Chloroform-*d*)

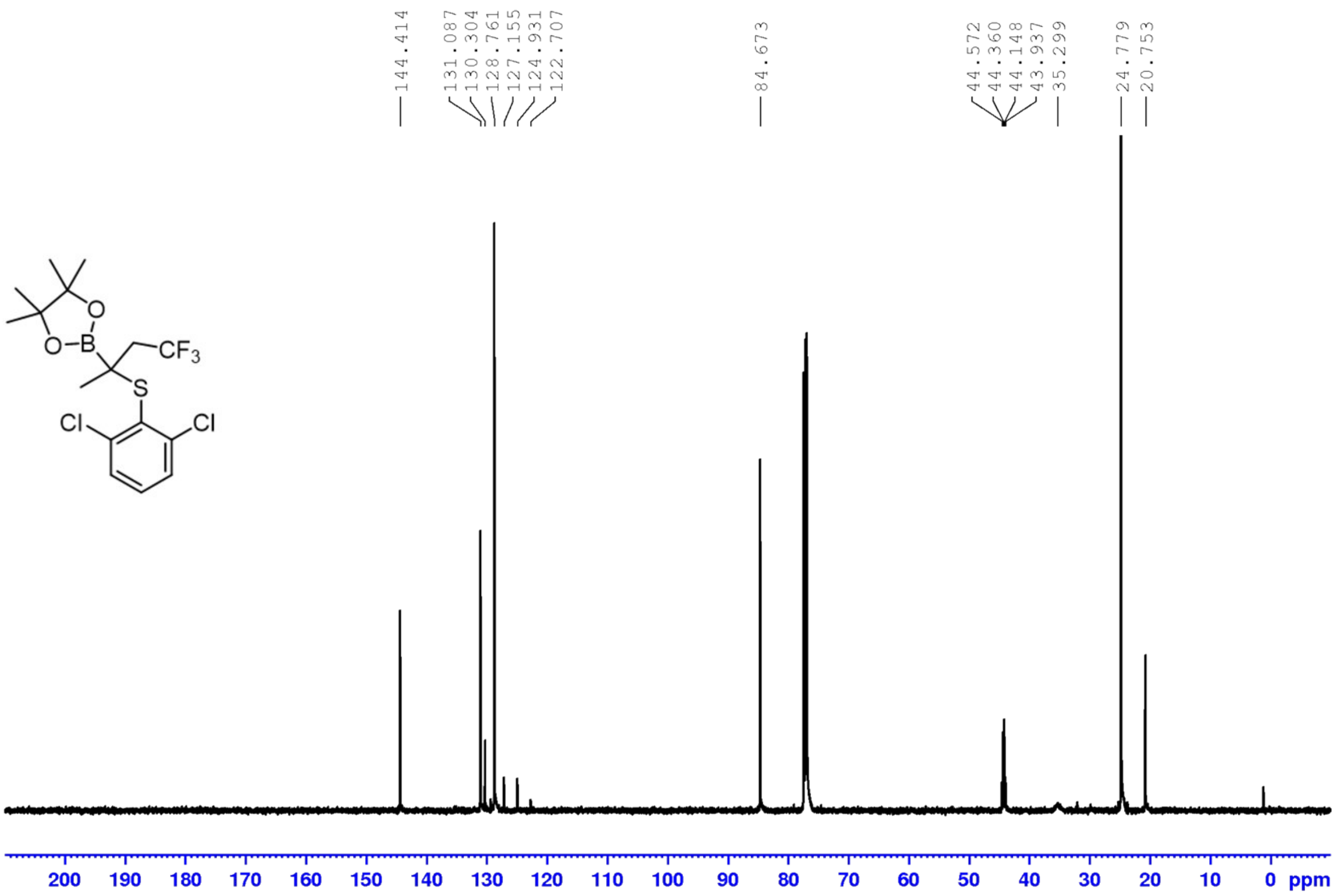

^{11}B NMR (160 MHz, Chloroform-*d*)

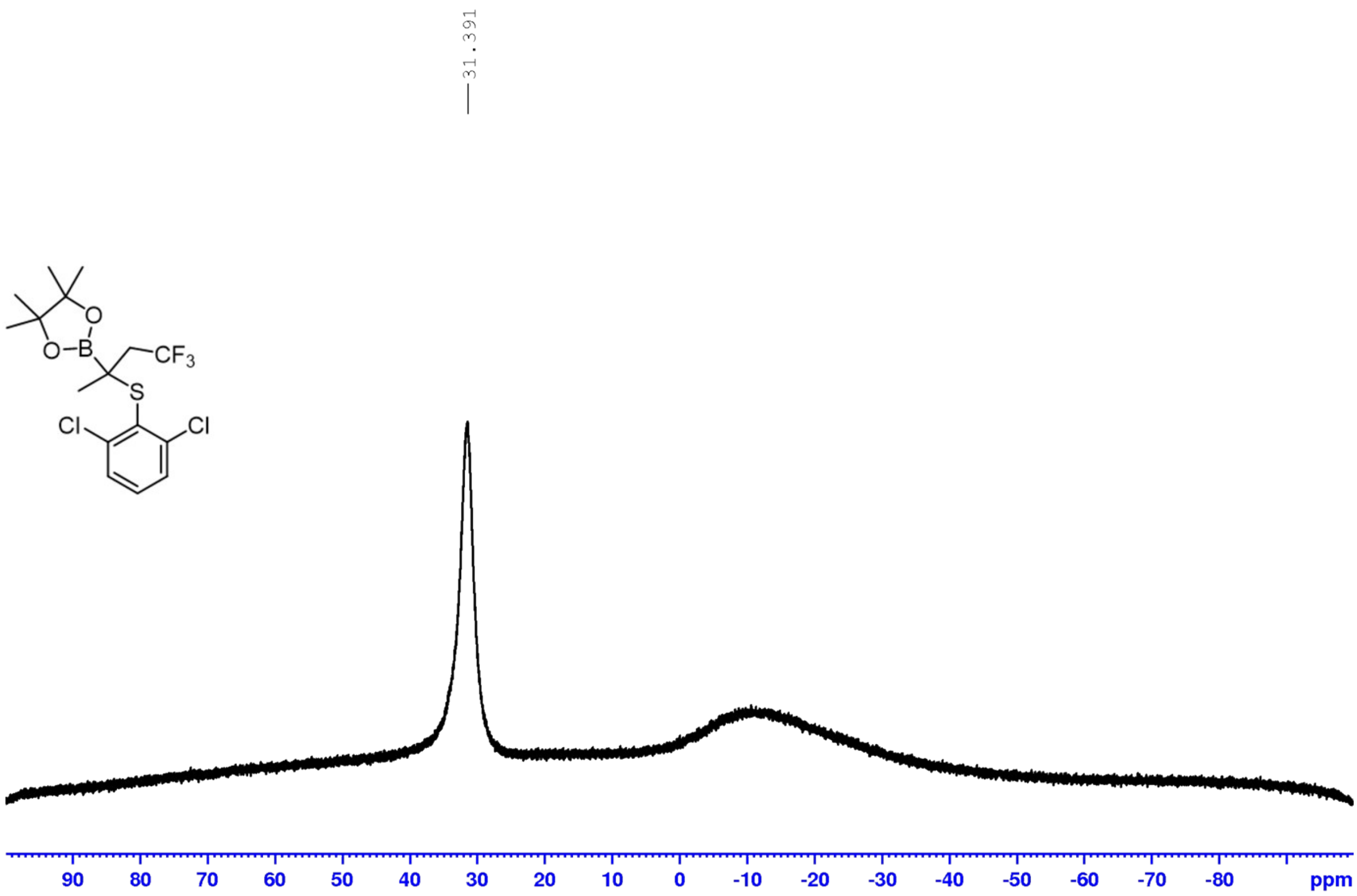

^{19}F NMR (376 MHz, Chloroform-*d*)

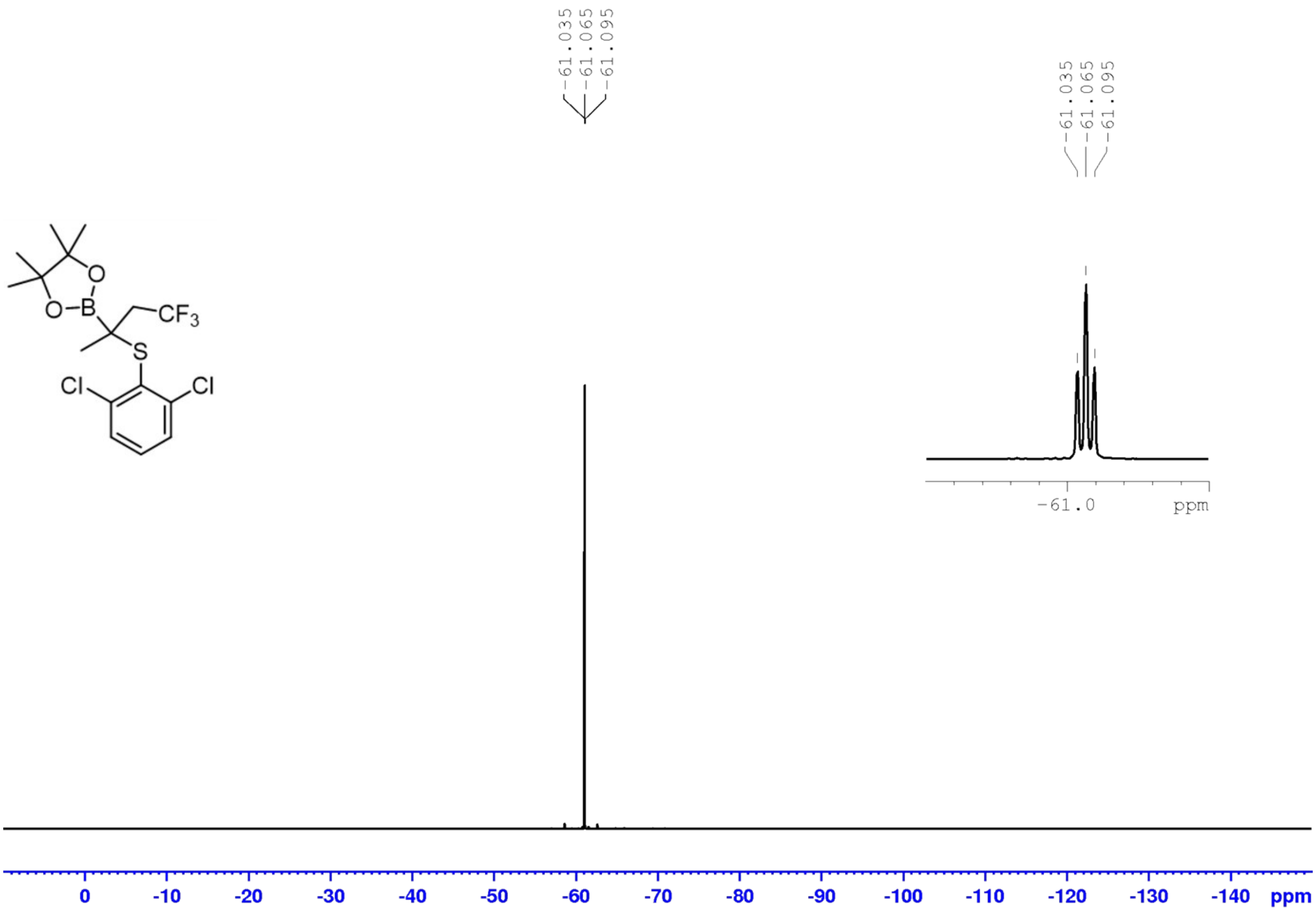

2-(2-((4-bromophenyl)thio)-4,4,4-trifluorobutan-2-yl)-4,4,5,5-tetramethyl-1,3,2-dioxaborolane (5k)

^{1}H NMR (400 MHz, Chloroform-*d*)

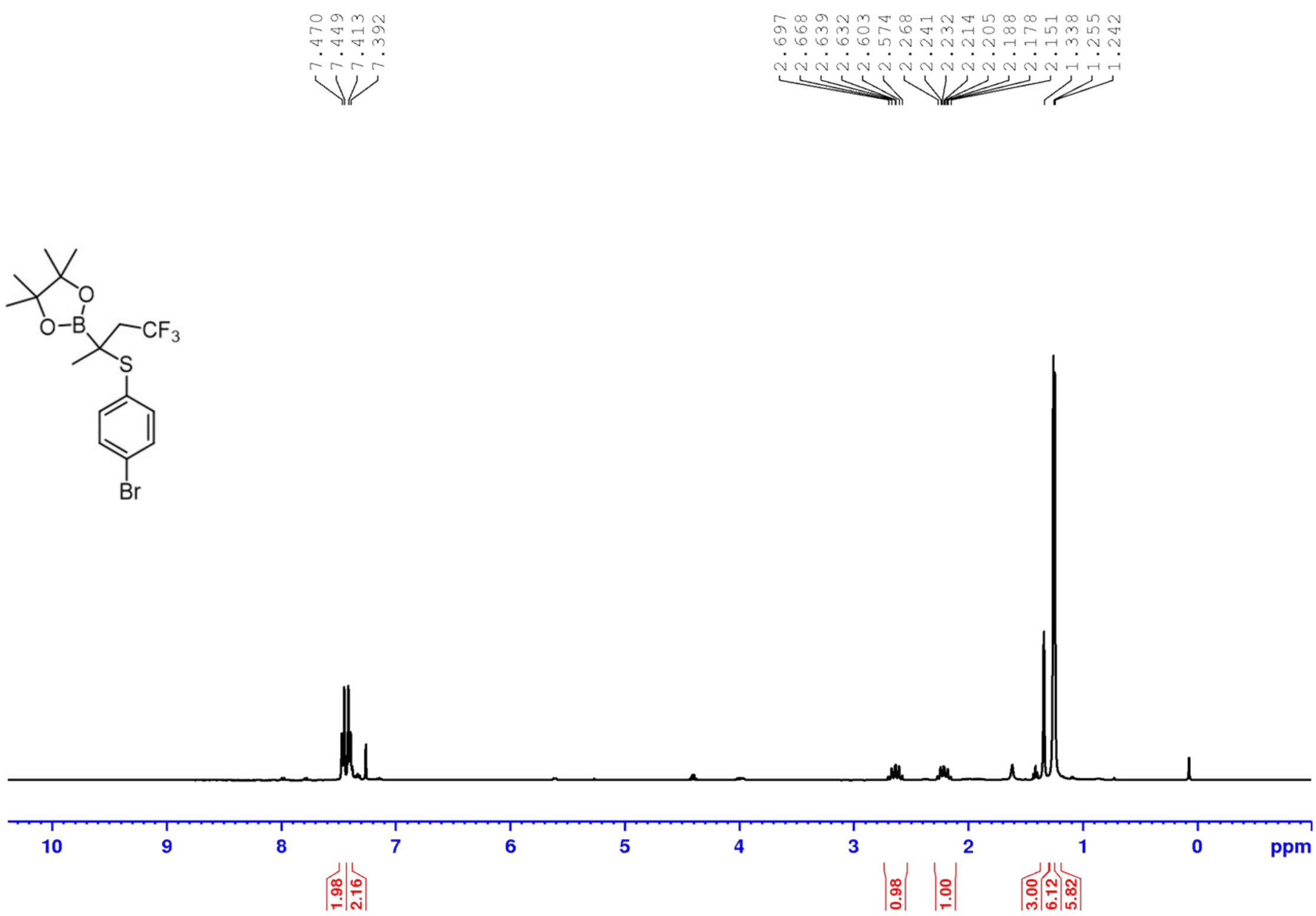

^{13}C NMR (100 MHz, Chloroform-*d*)

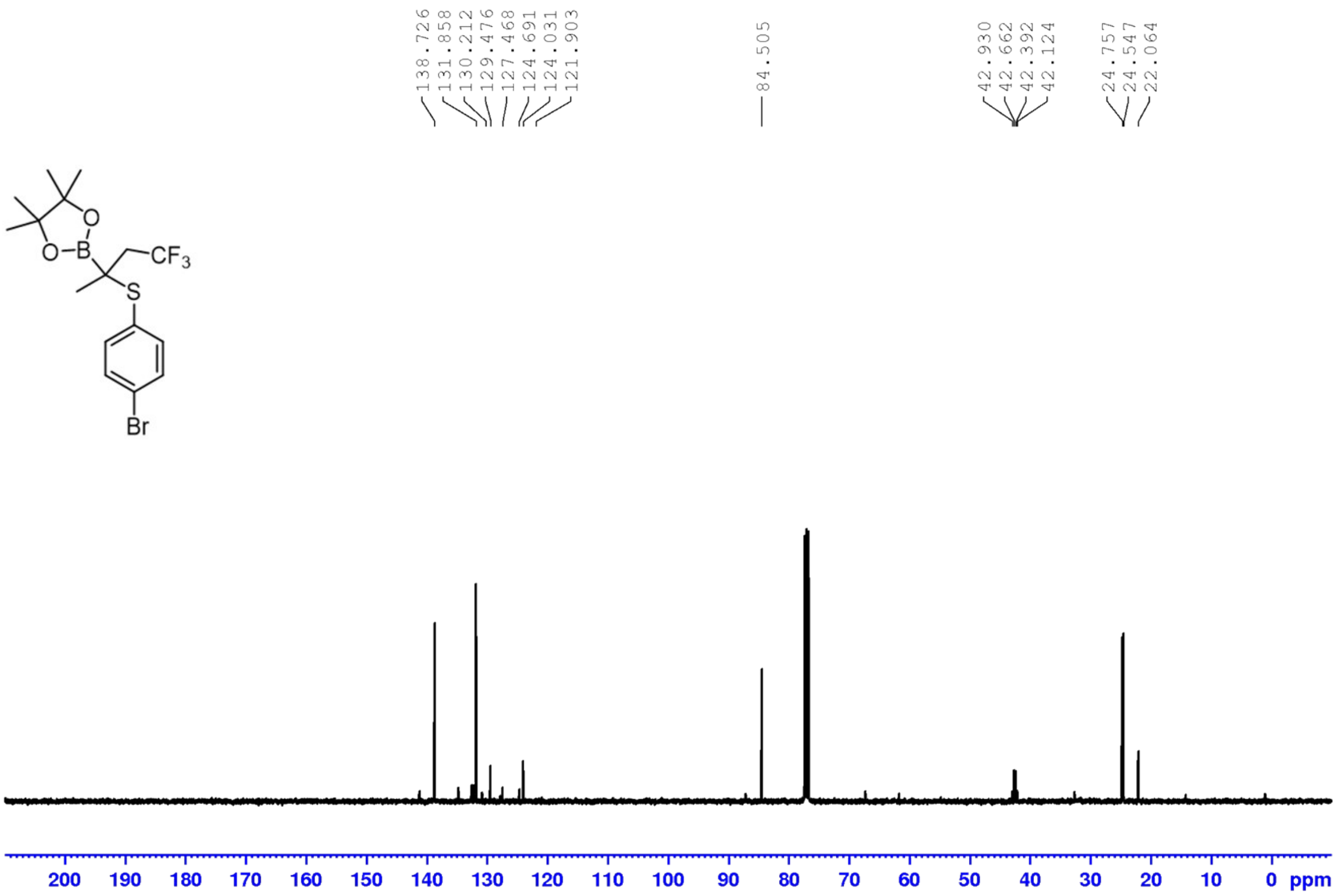

^{11}B NMR (128 MHz, Chloroform-*d*)

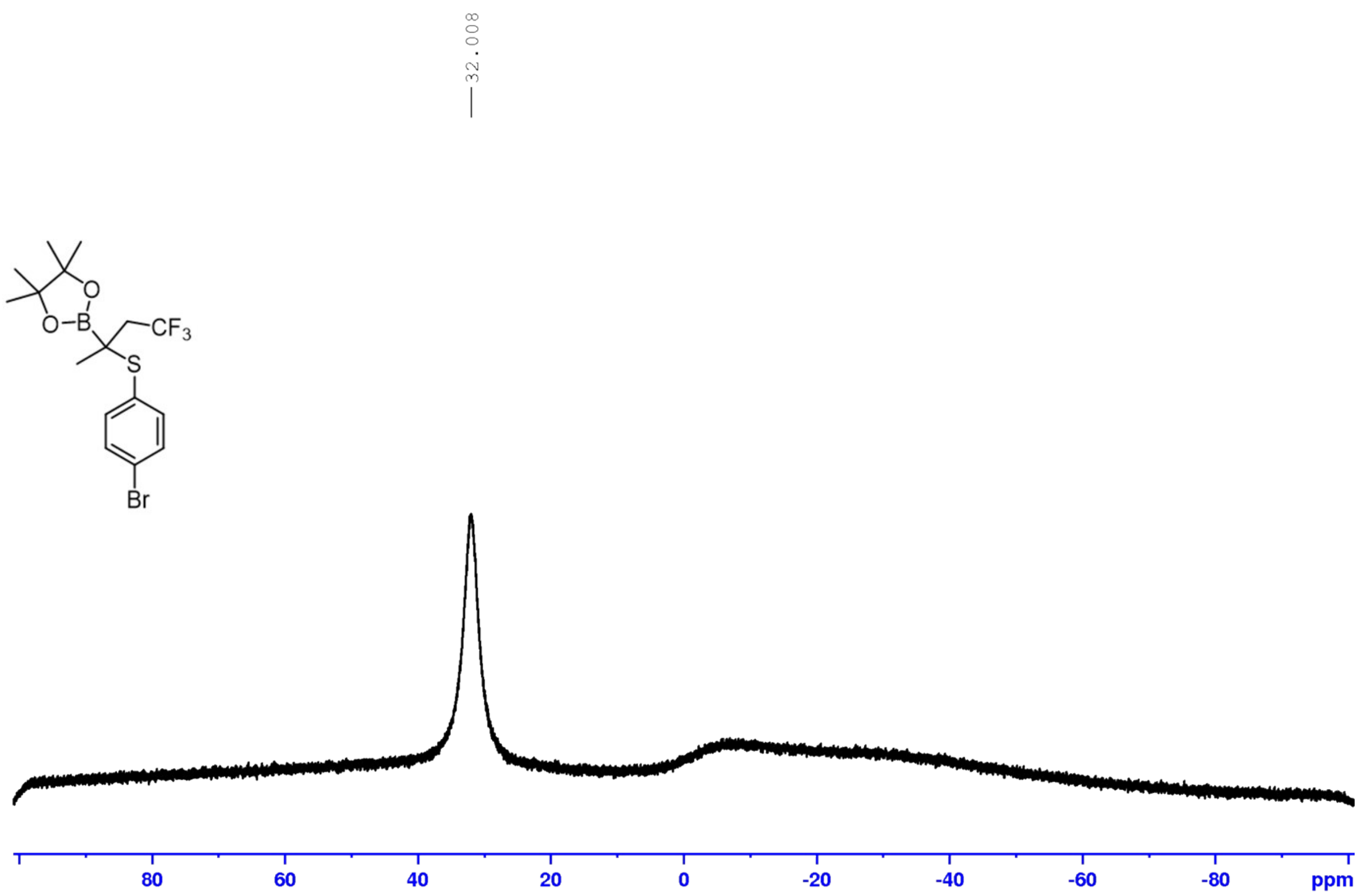

^{19}F NMR (376 MHz, Chloroform-*d*)

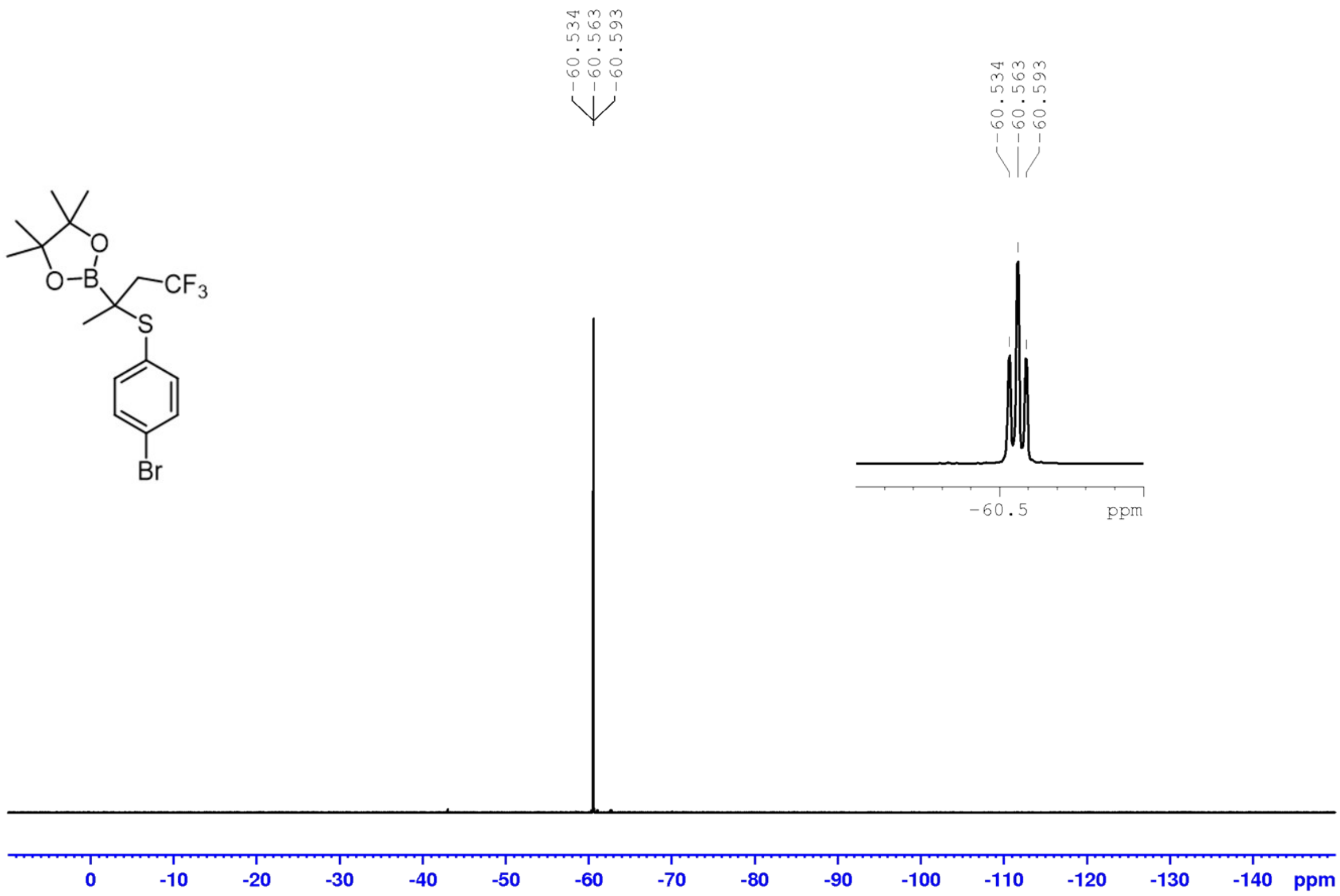

2-(3-(benzylthio)-1,1,1-trifluoro-5-(3-methoxyphenyl)pentan-3-yl)-4,4,5,5-tetramethyl-1,3,2-dioxaborolane (5l)

^{1}H NMR (400 MHz, Chloroform-*d*)

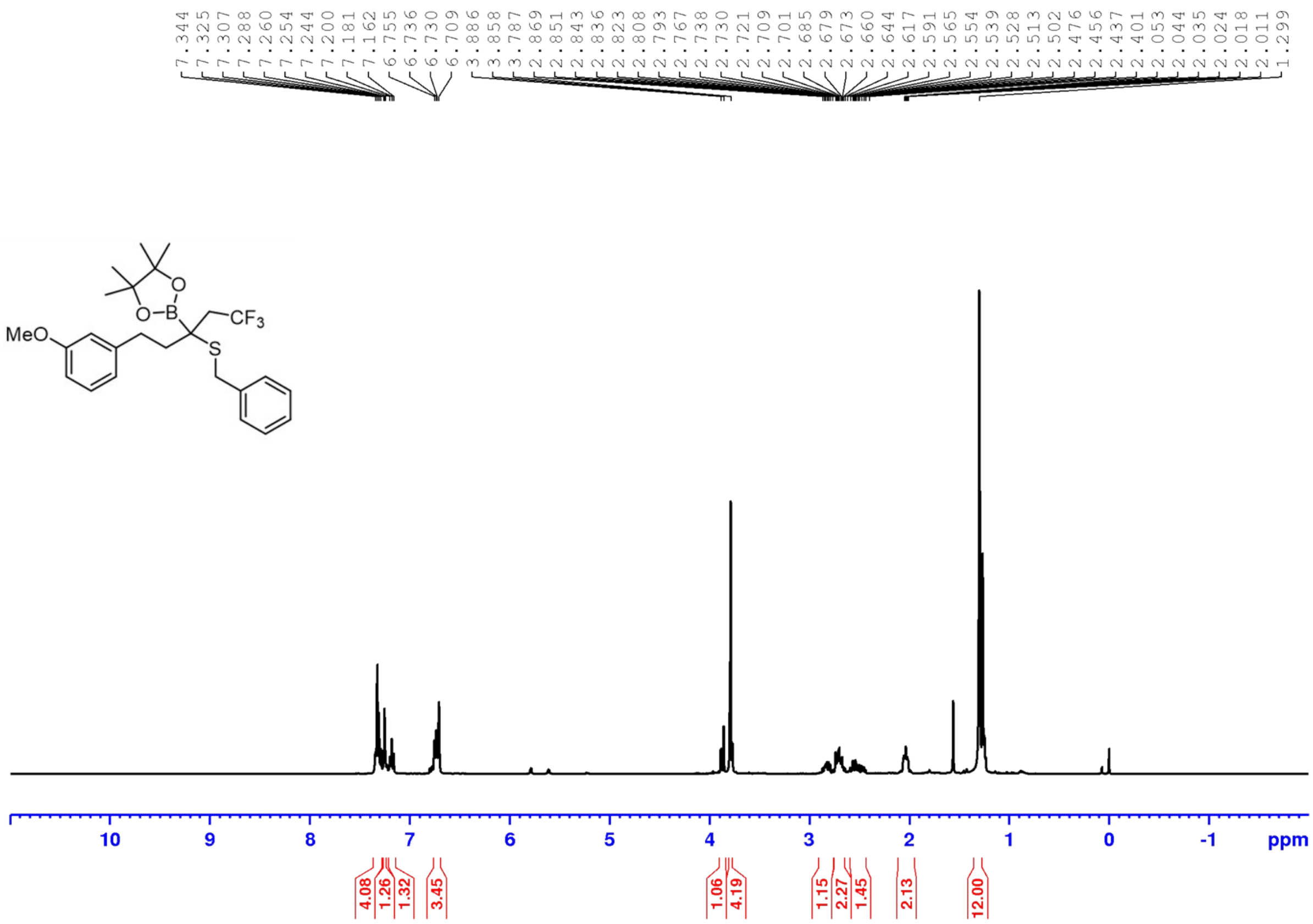

^{13}C NMR (125 MHz, Chloroform-*d*)

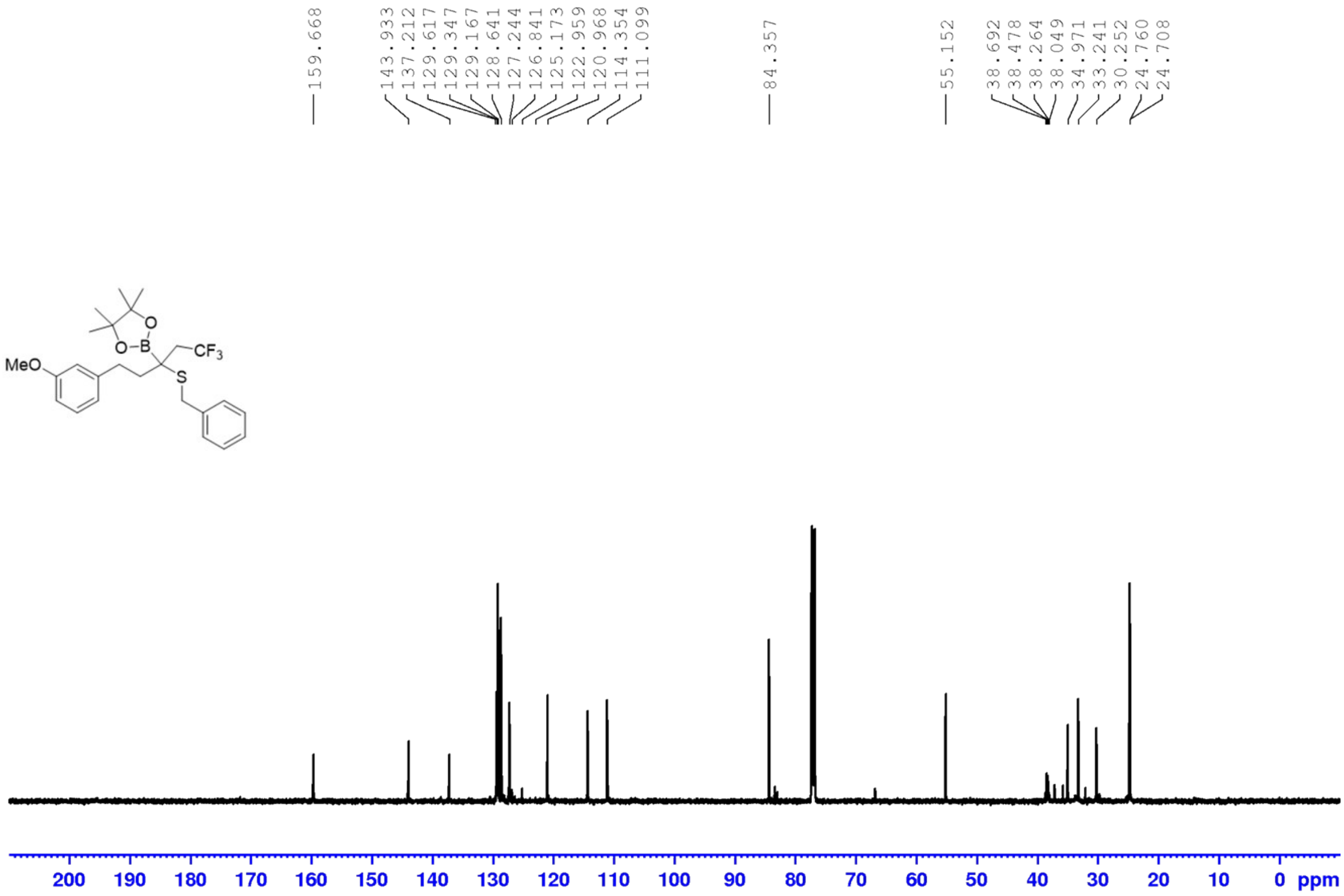

^{11}B NMR (128 MHz, Chloroform-*d*)

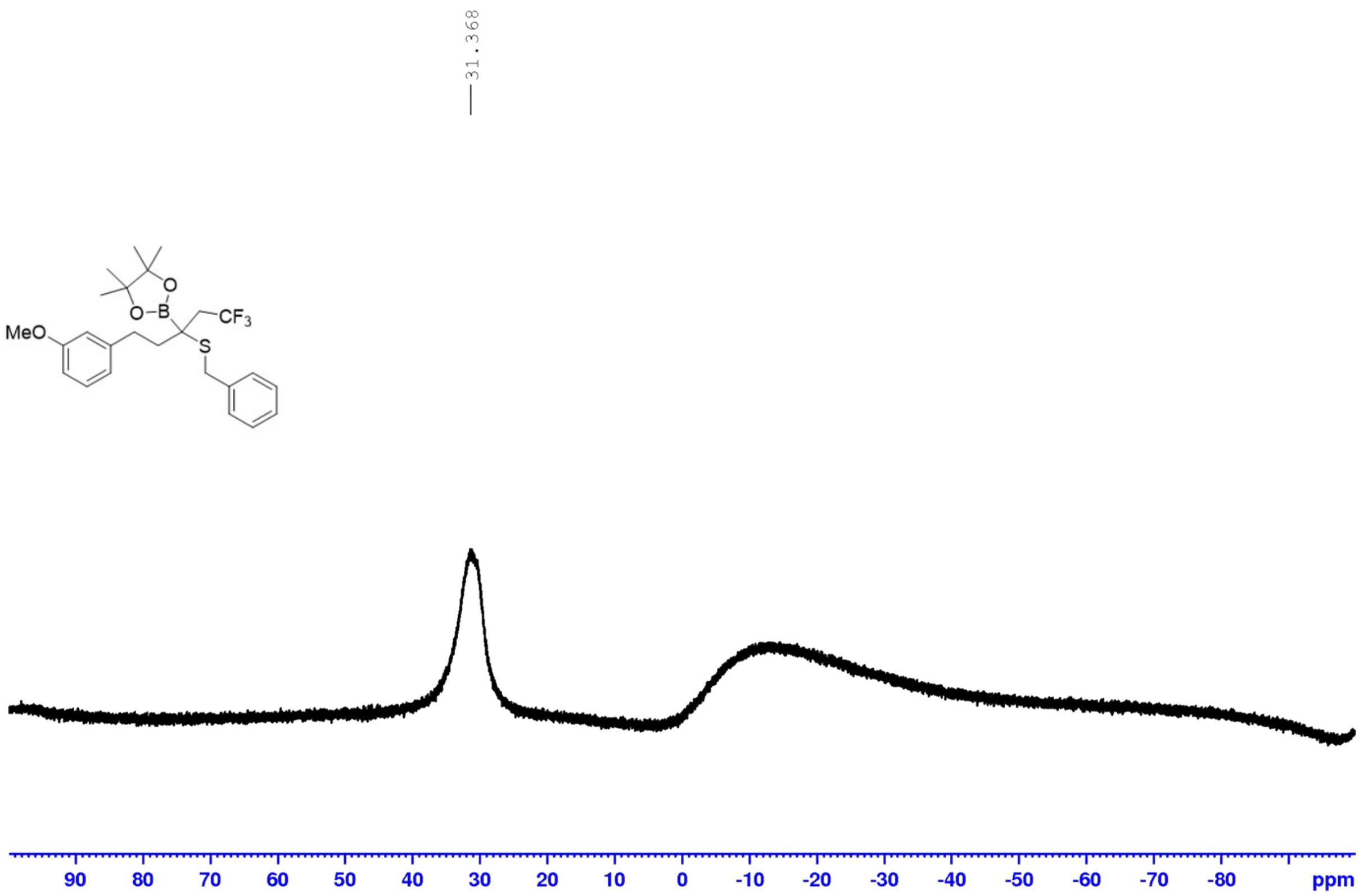

^{19}F NMR (376 MHz, Chloroform-*d*)

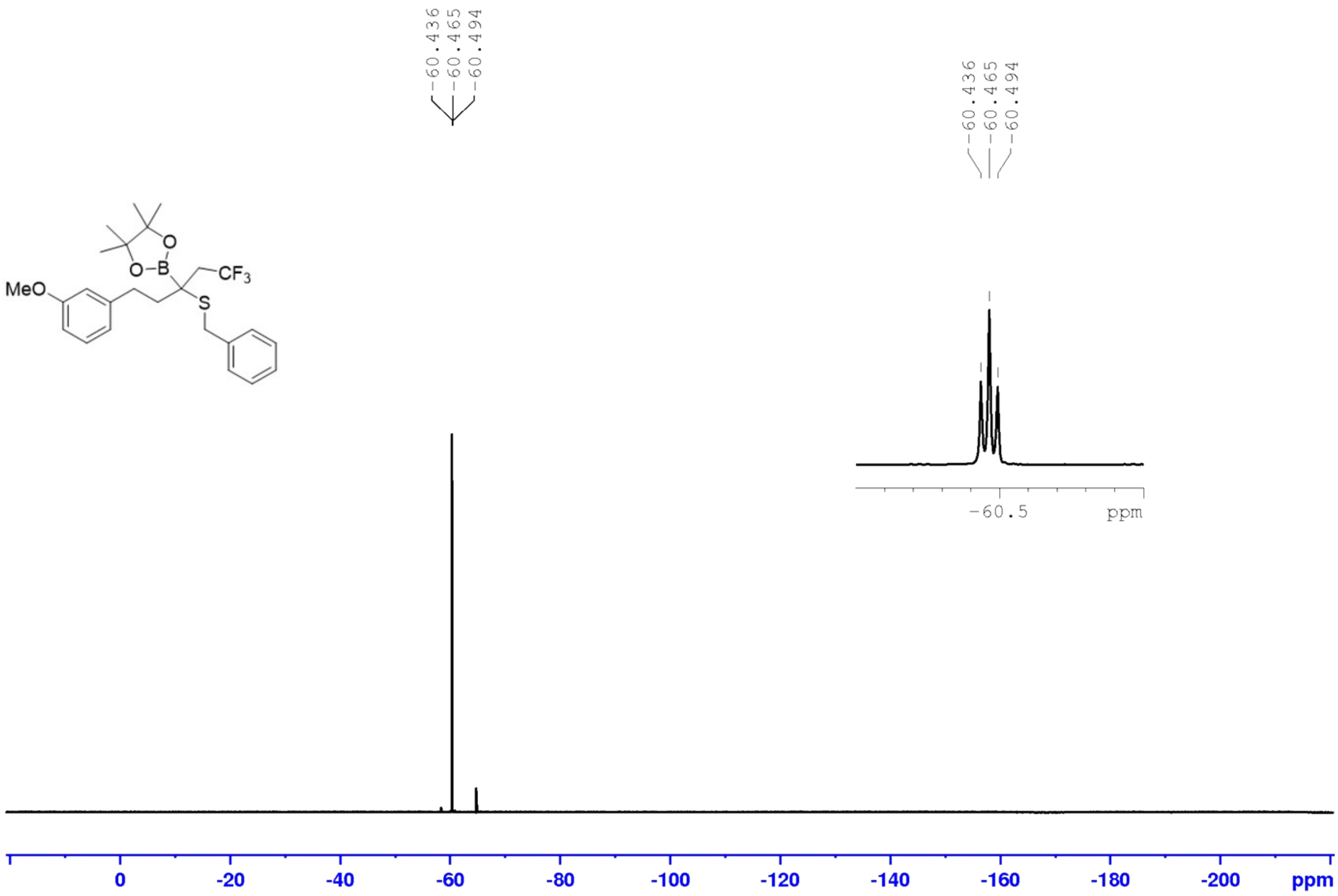

2-(3-(dodecylthio)-1,1,1-trifluoro-5-(3-methoxyphenyl)pentan-3-yl)-4,4,5,5-tetramethyl-1,3,2-dioxaborolane (5m)

^{1}H NMR (400 MHz, Chloroform-*d*)

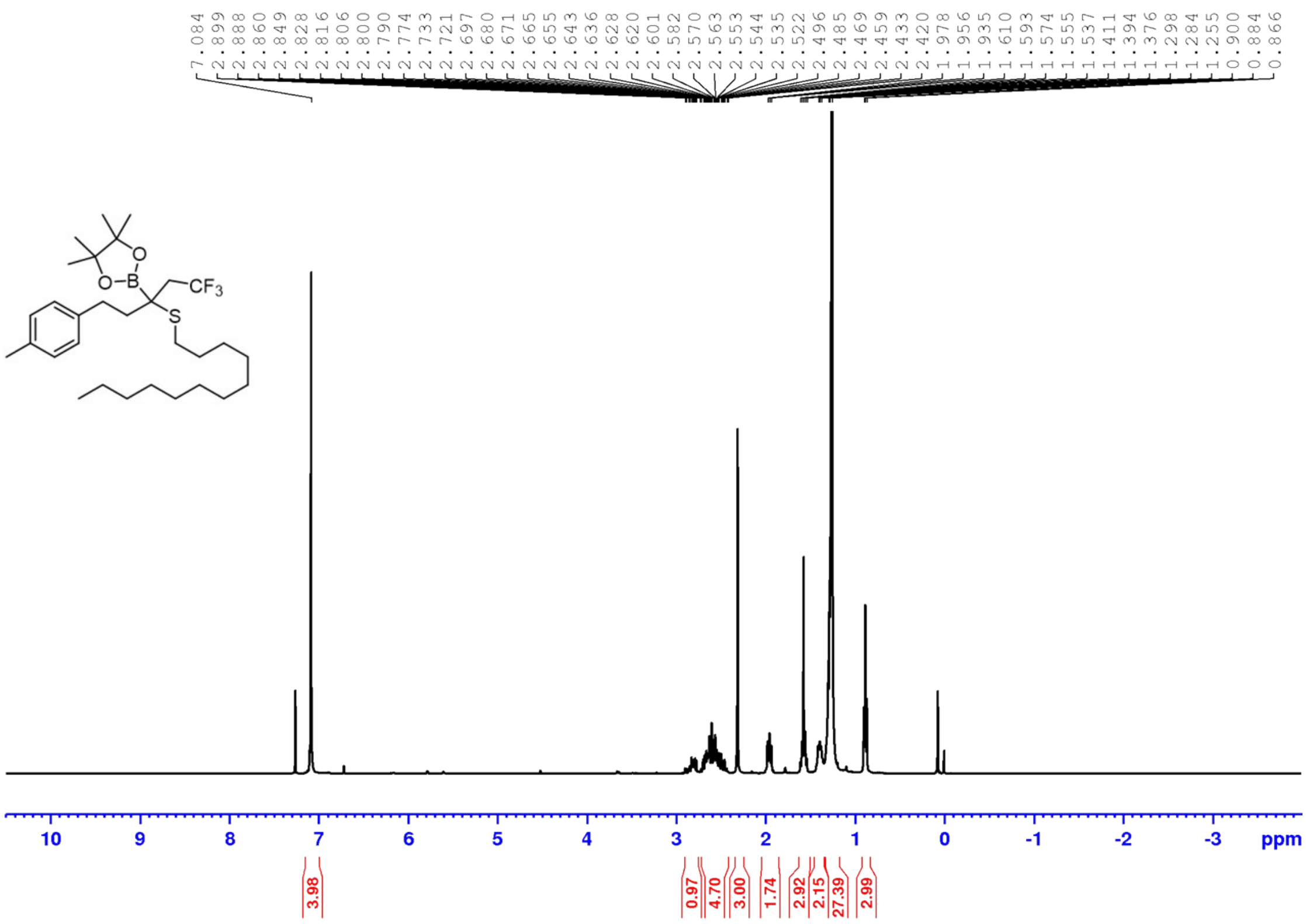

^{13}C NMR (125 MHz, Chloroform-*d*)

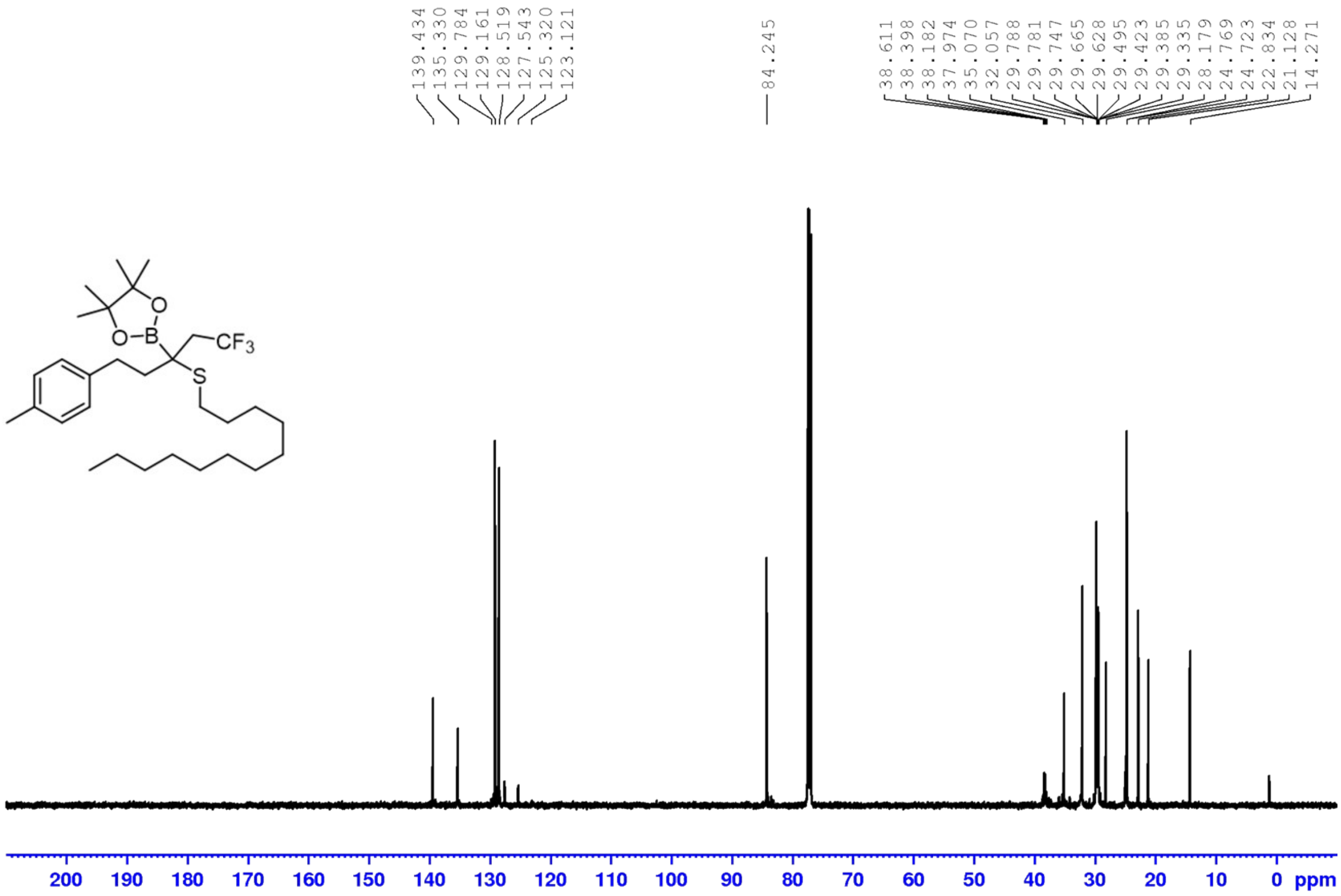

^{11}B NMR (128 MHz, Chloroform-*d*)

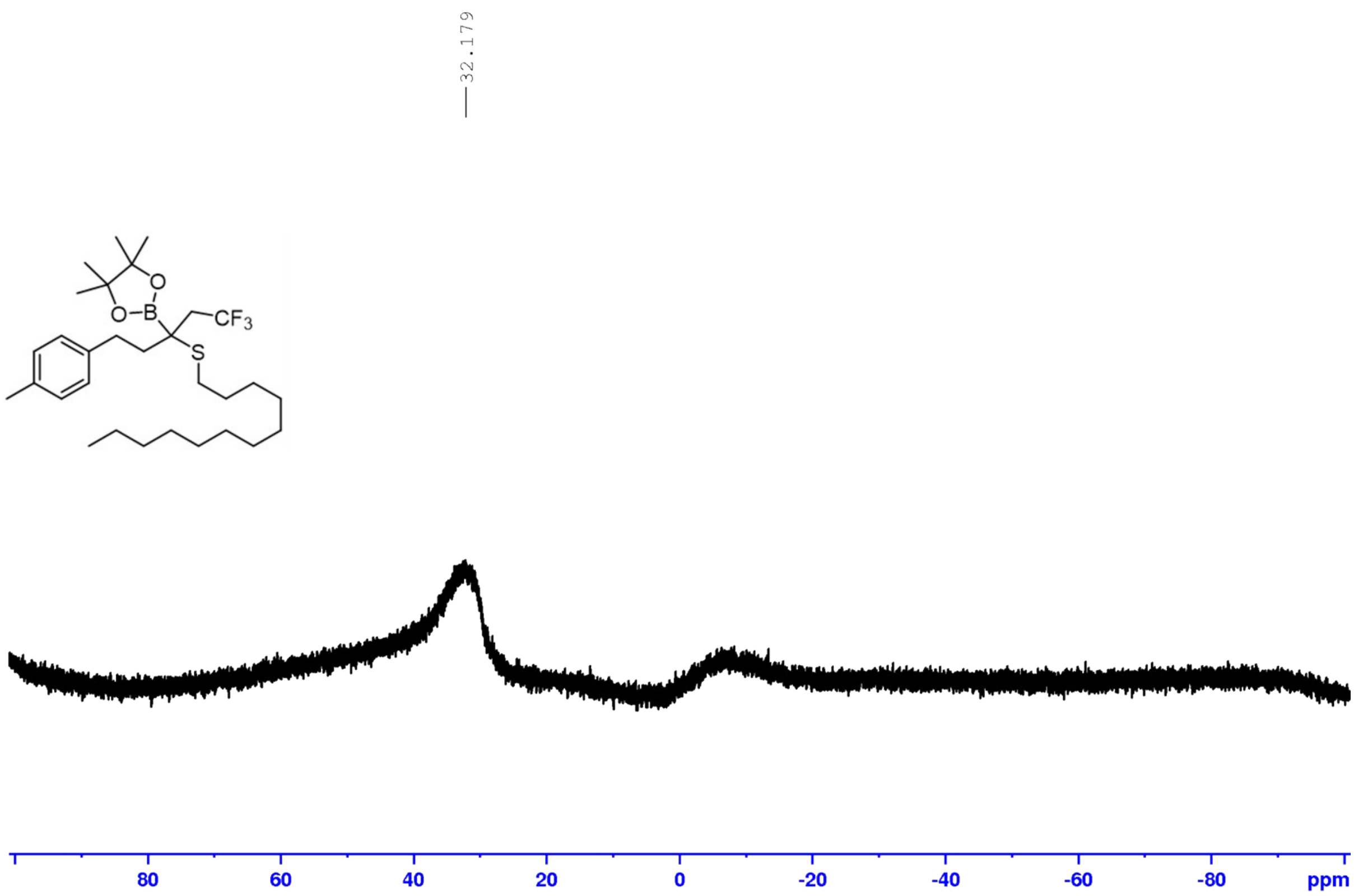

^{19}F NMR (376 MHz, Chloroform-*d*)

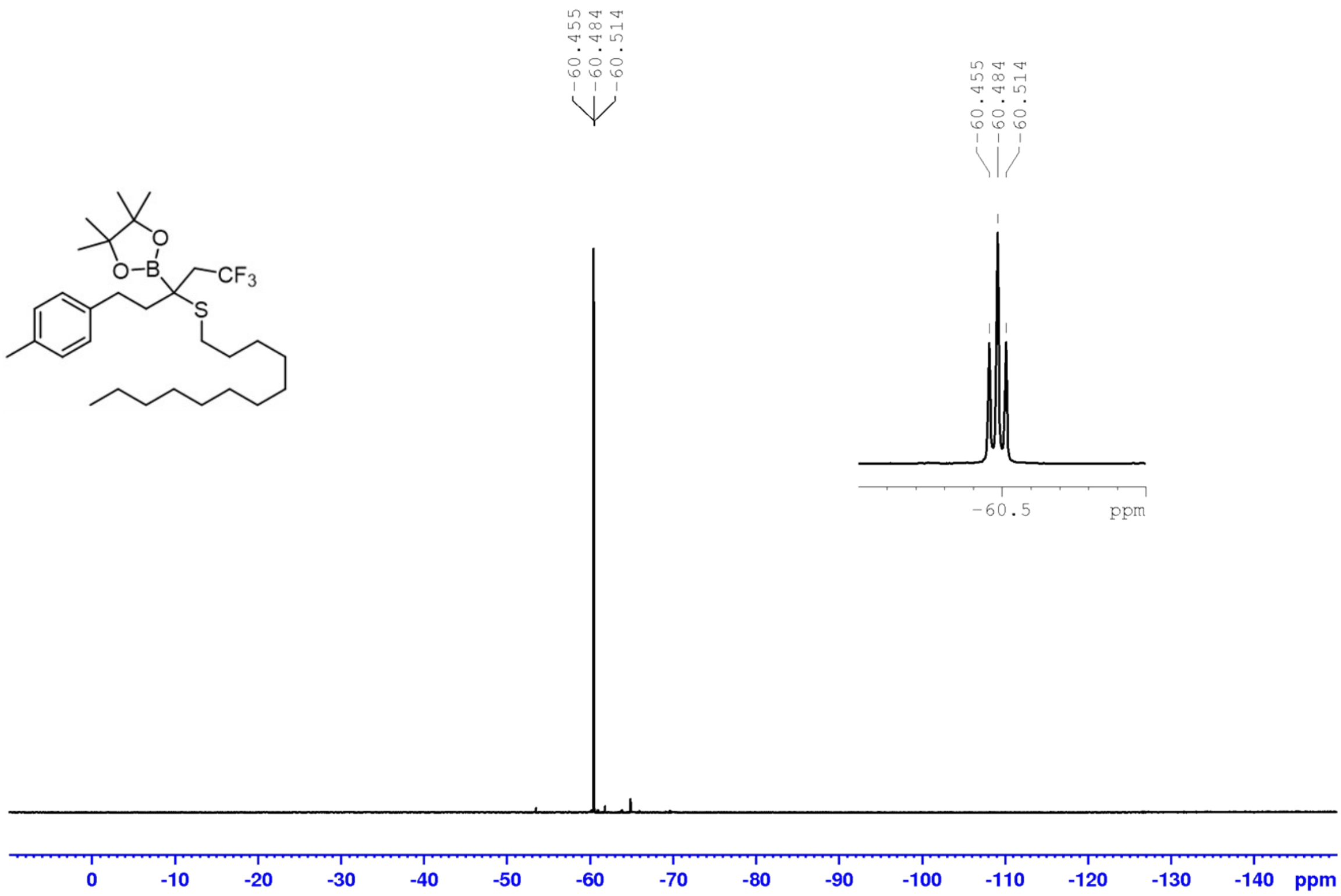

2-(3-(ethylthio)-1,1,1-trifluoro-5-(4-fluorophenyl)pentan-3-yl)-4,4,5,5-tetramethyl-1,3,2-dioxaborolane (5n)

^{1}H NMR (400 MHz, Chloroform-*d*)

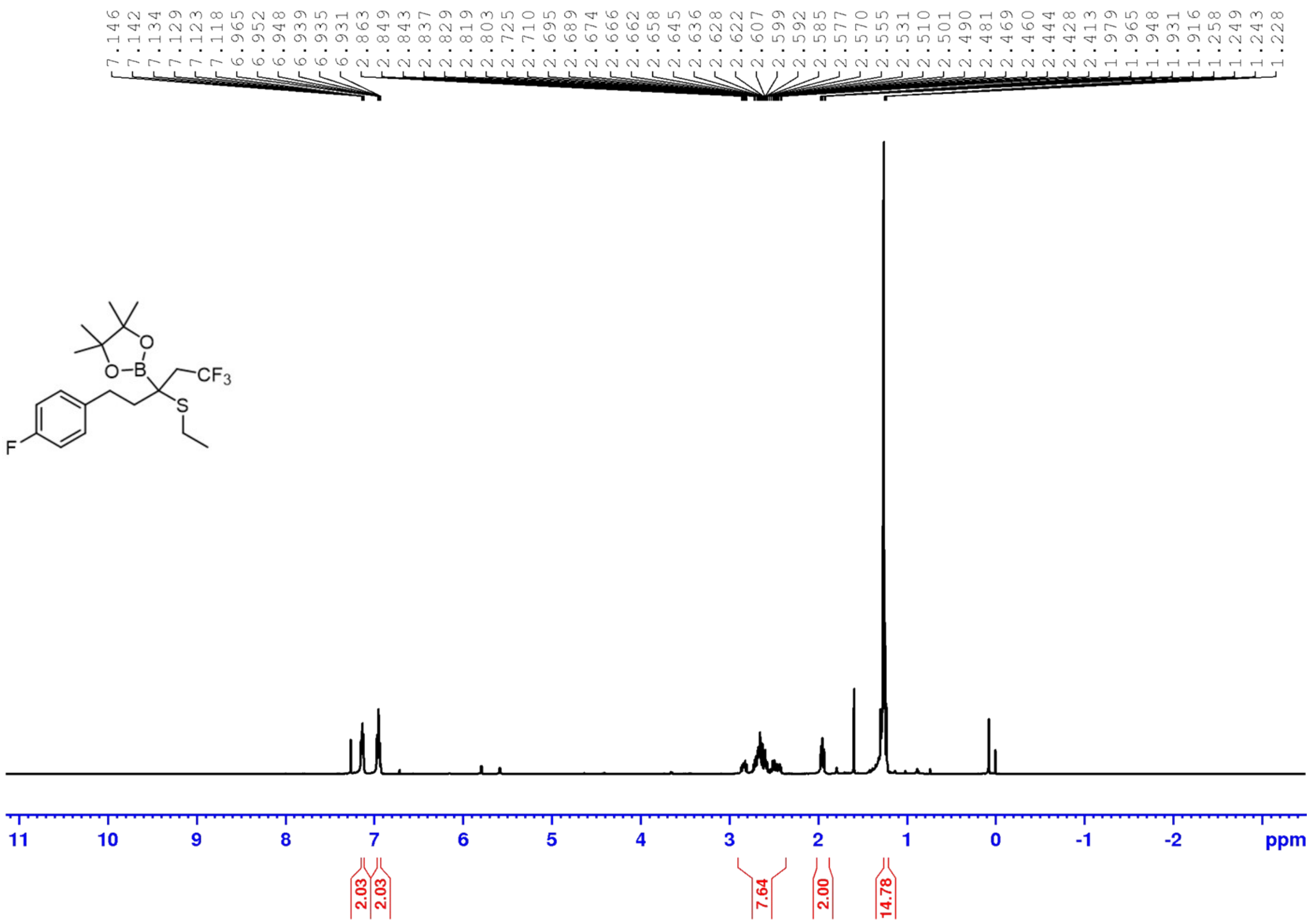

^{13}C NMR (100 MHz, Chloroform-*d*)

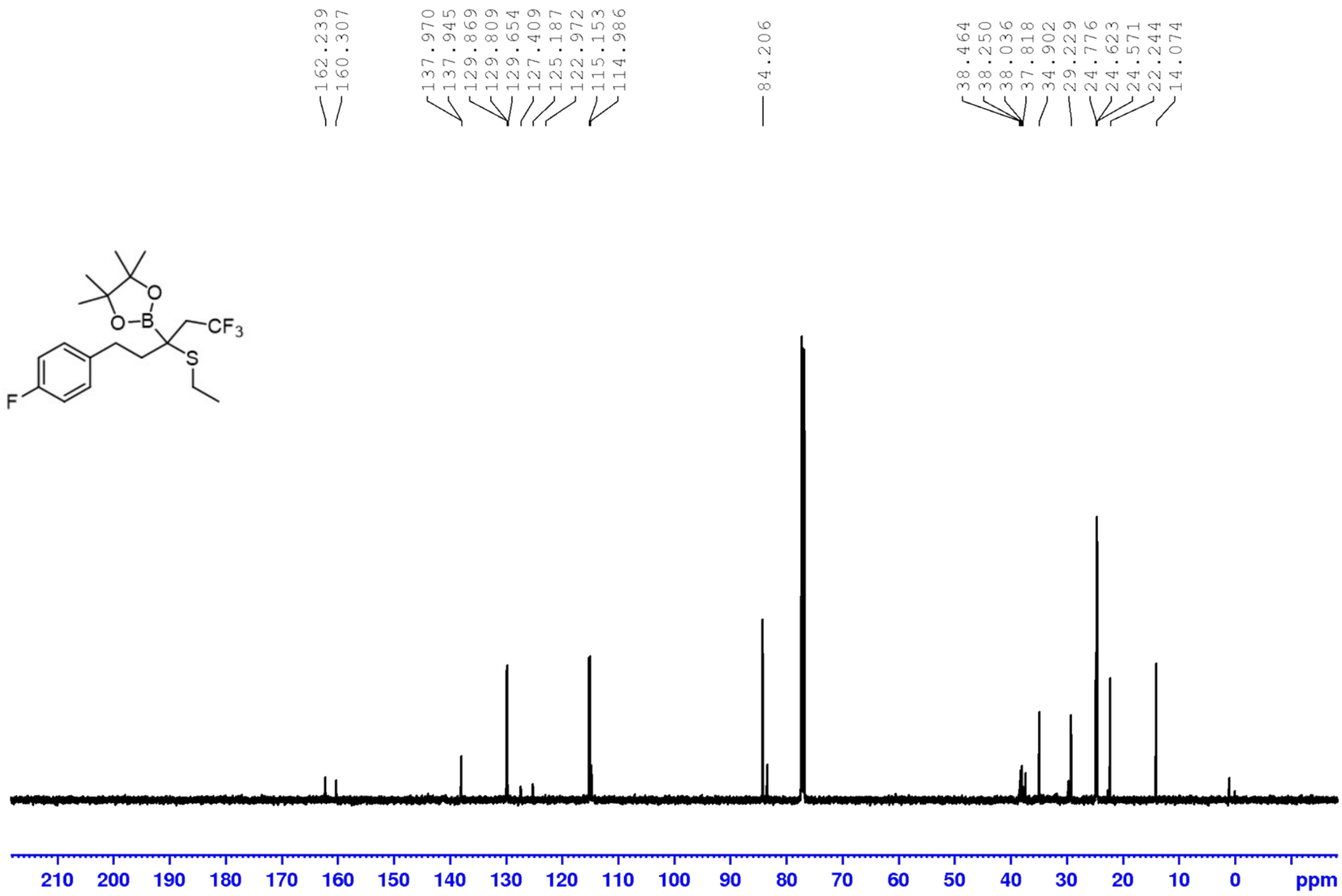

^{11}B NMR (128 MHz, Chloroform-*d*)

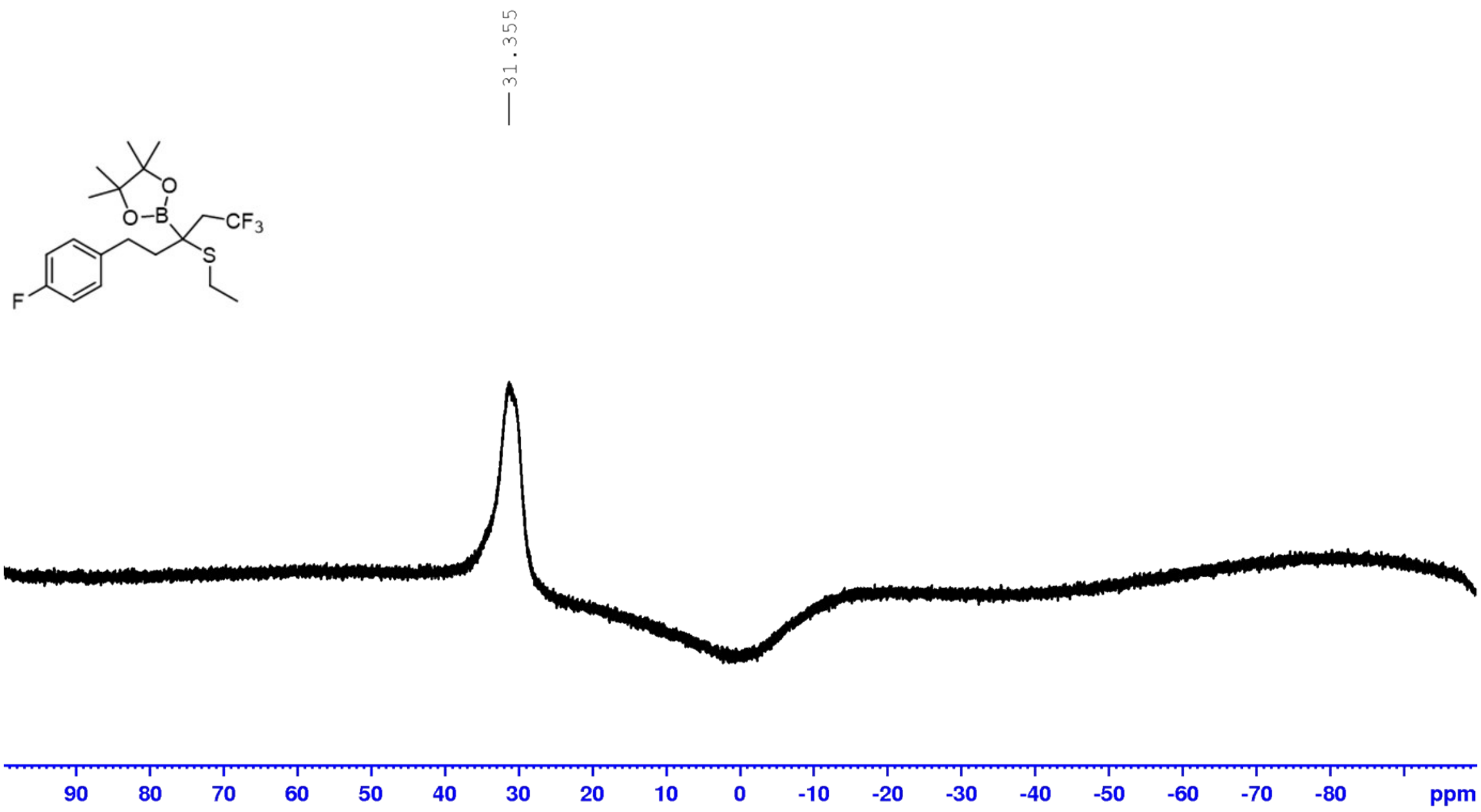

^{19}F NMR (376 MHz, Chloroform-*d*)

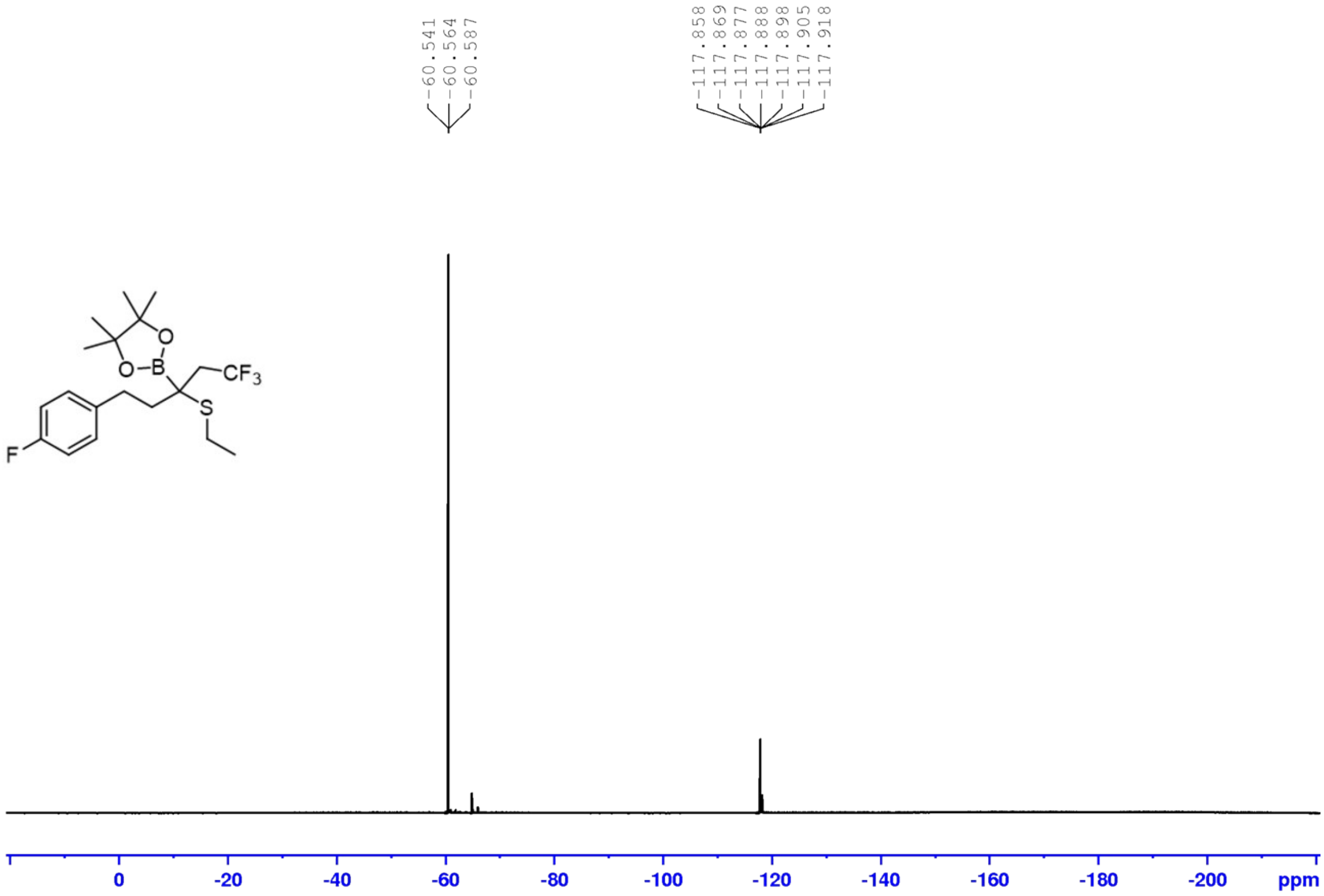

2-(2-(cyclohexylthio)-4,4,4-trifluorobutan-2-yl)-4,4,5,5-tetramethyl-1,3,2-dioxaborolane (5o)

^{1}H NMR (400 MHz, Chloroform-*d*)

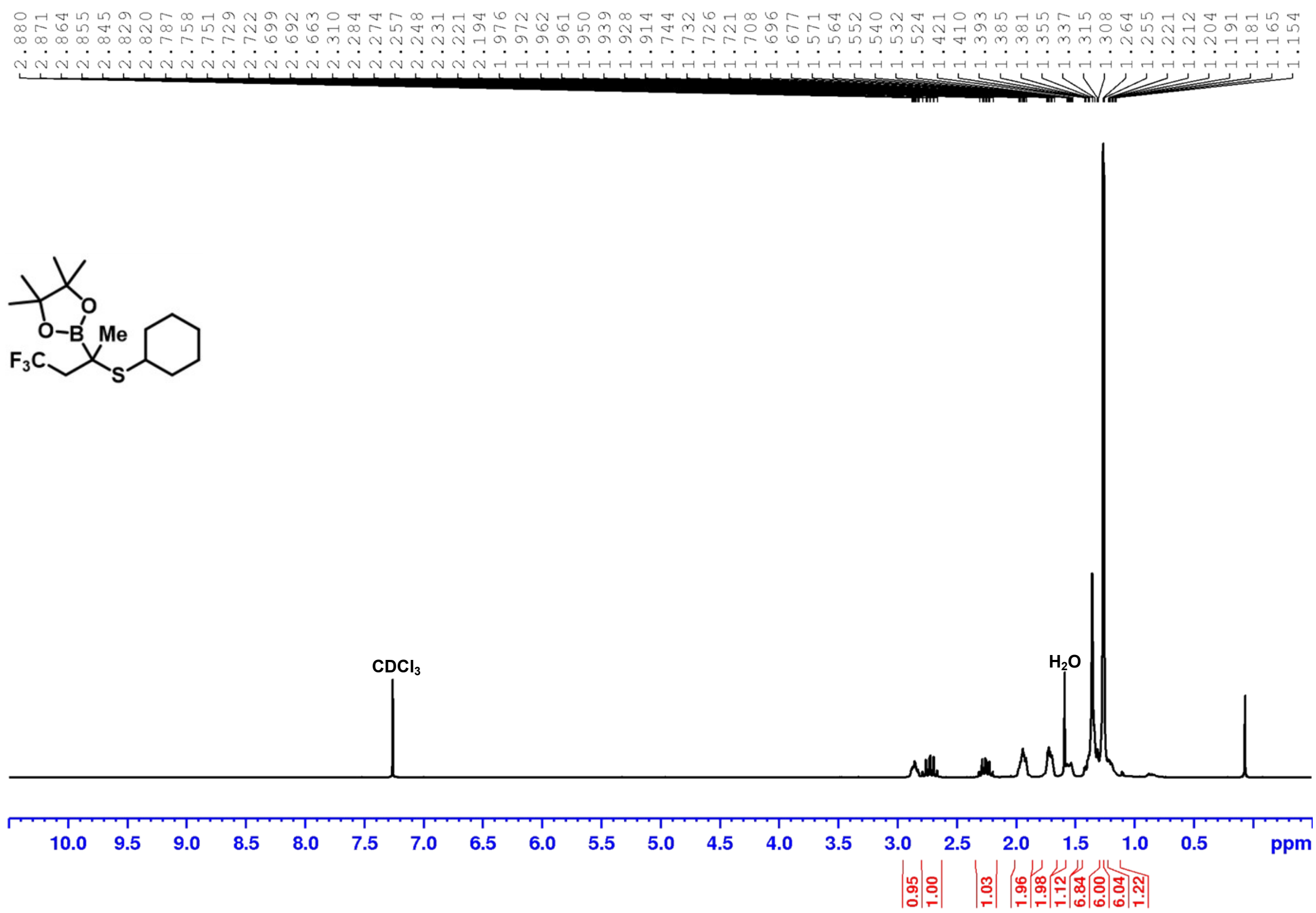

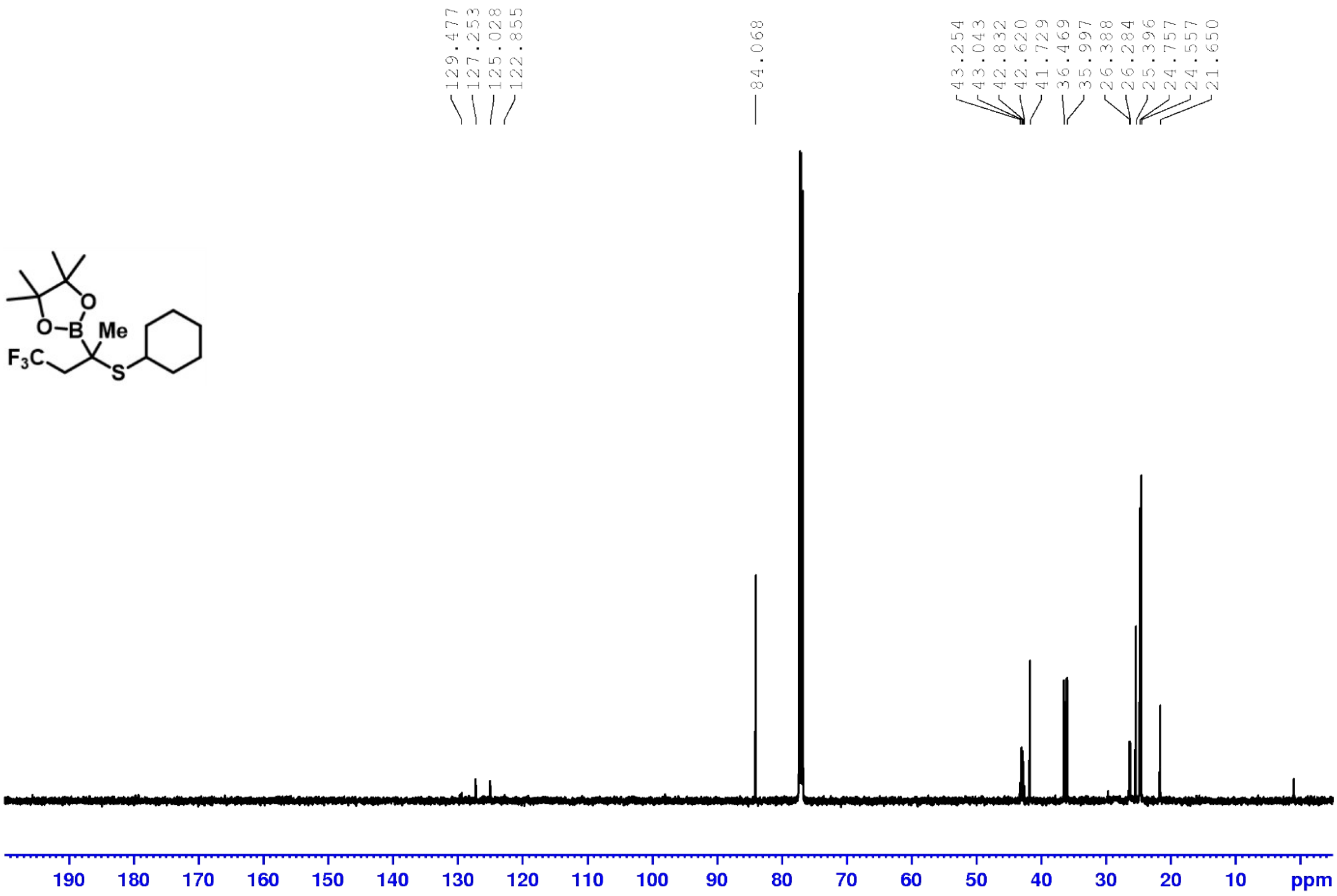

^{13}C NMR (126 MHz, Chloroform-*d*)

^{11}B NMR (160 MHz, Chloroform-*d*)

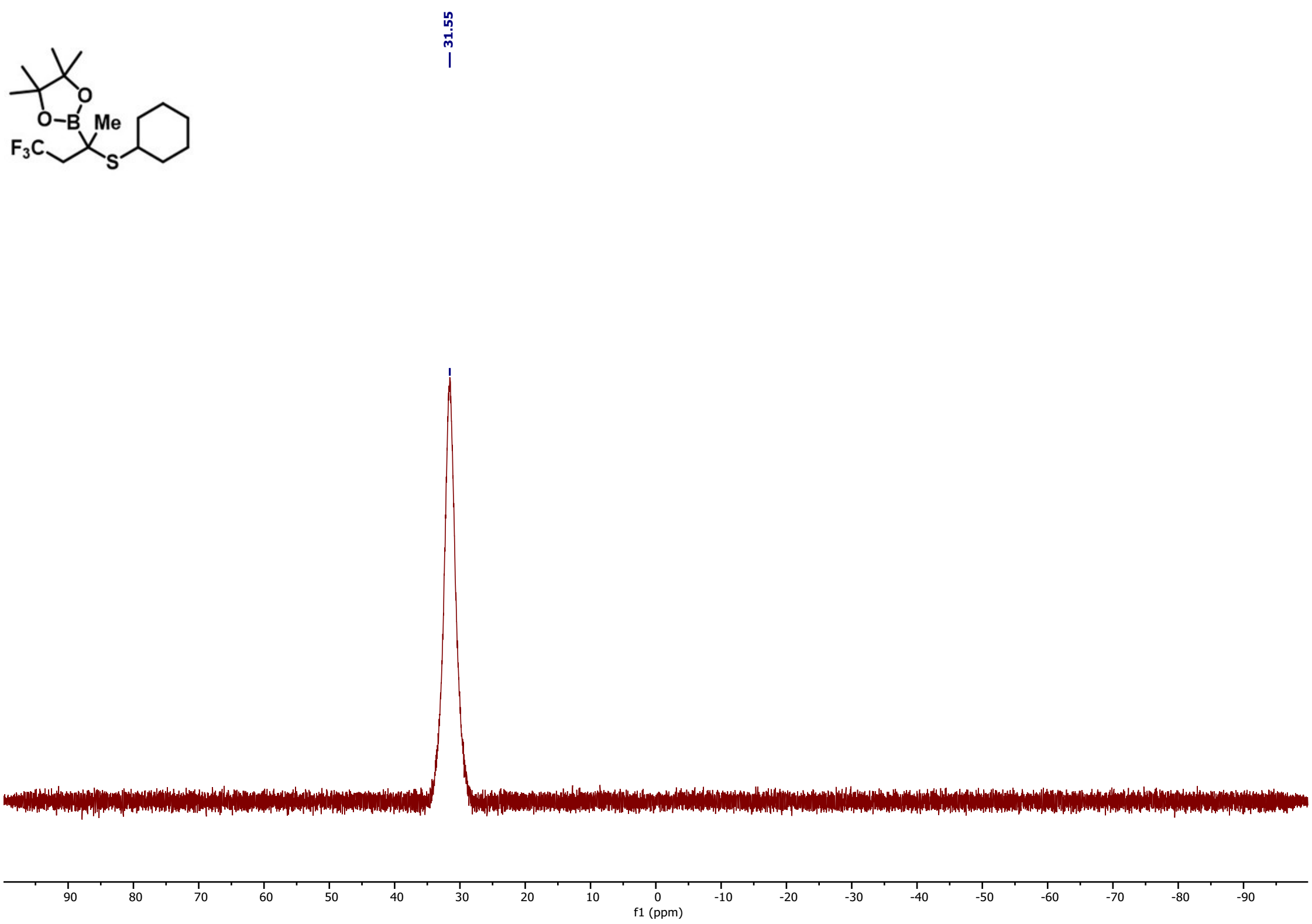

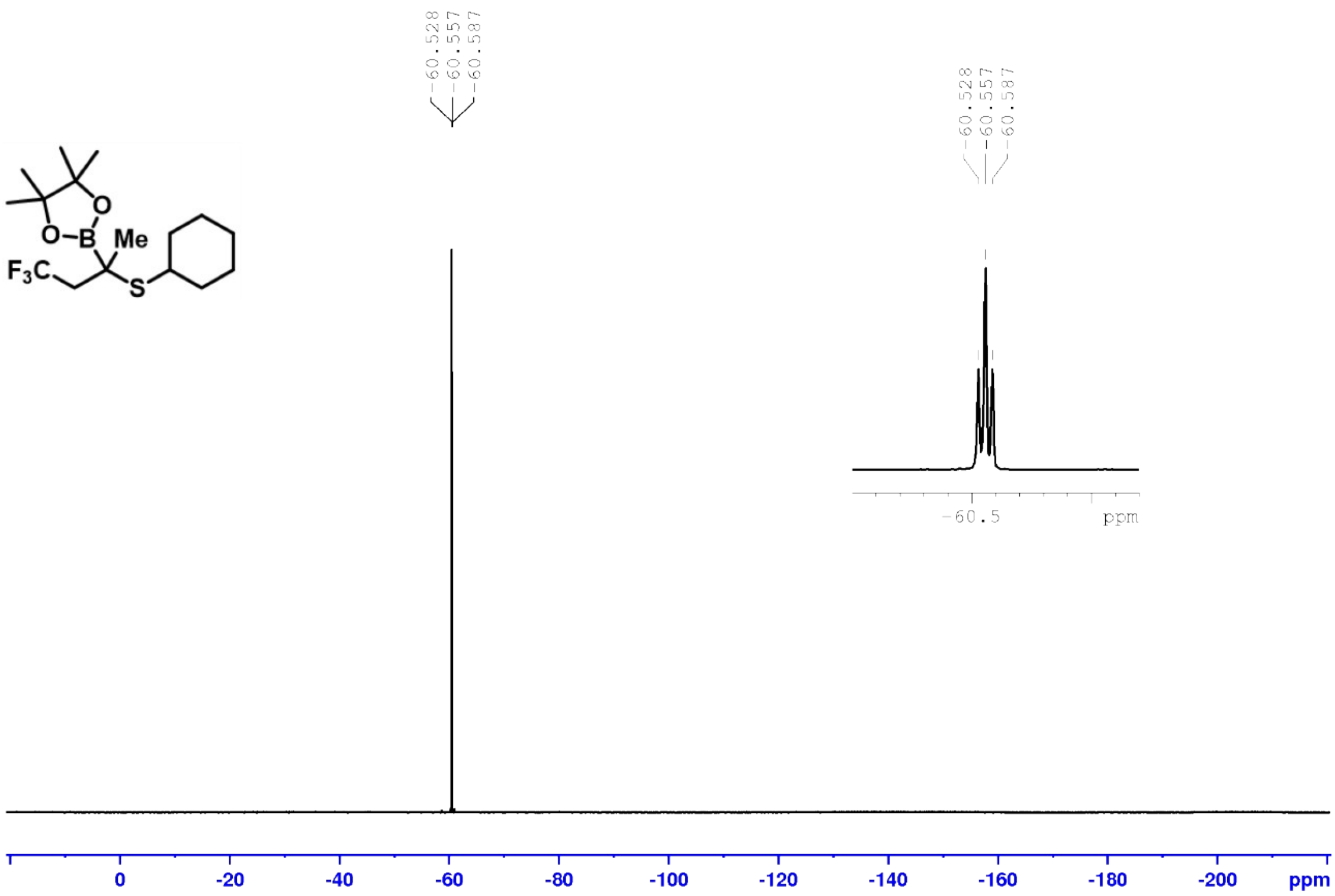

^{19}F NMR (376 MHz, Chloroform-*d*)

5,5,5-trifluoro-3-(phenylthio)-3-(4,4,5,5-tetramethyl-1,3,2-dioxaborolan-2-yl)pentyl 4-(N,N-dipropylsulfamoyl)benzoate (3pa)

^{1}H NMR (400 MHz, Chloroform-*d*)

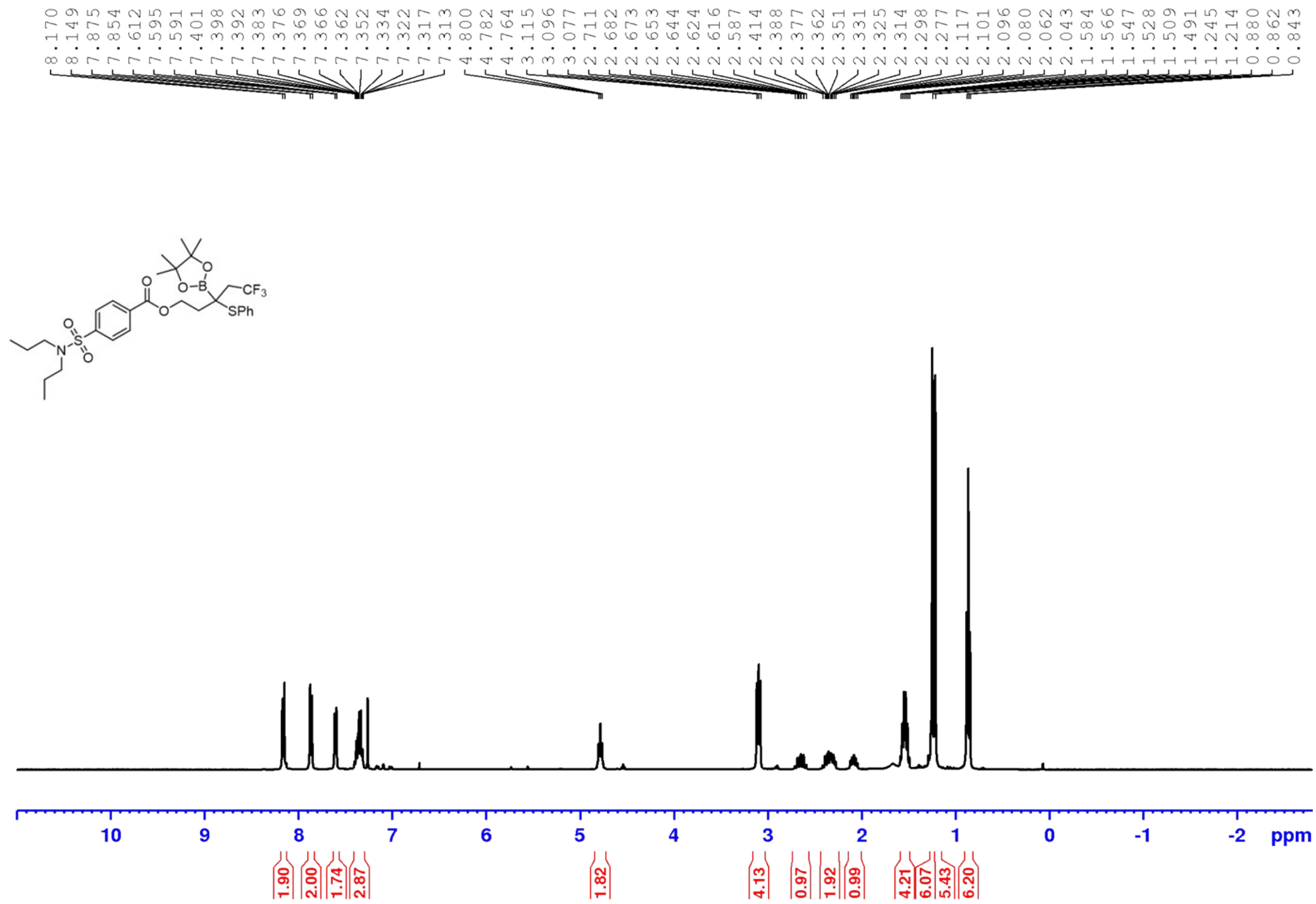

^{13}C NMR (100 MHz, Chloroform-*d*)

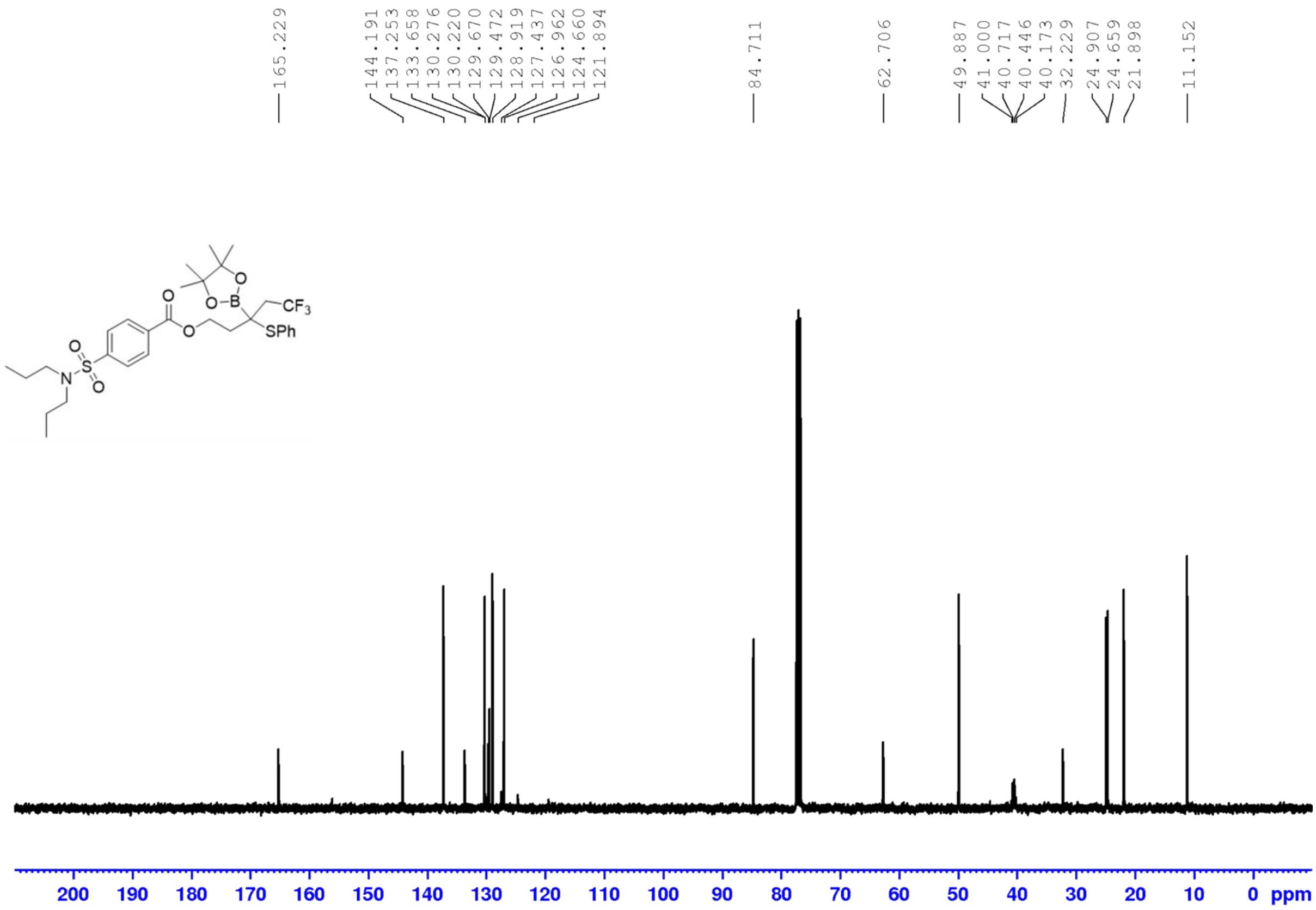

^{11}B NMR (128 MHz, Chloroform-*d*)

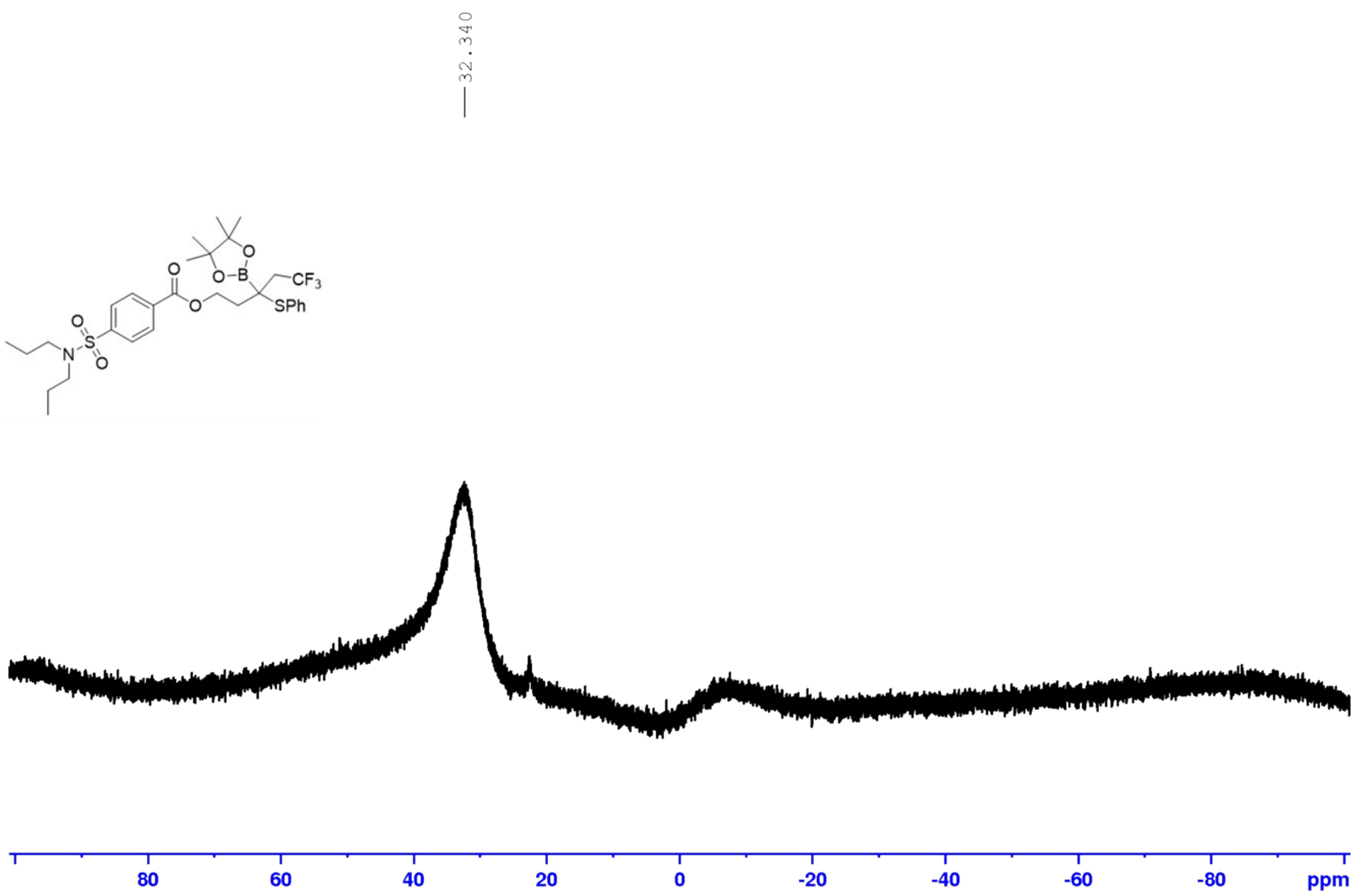

^{19}F NMR (376 MHz, Chloroform-*d*)

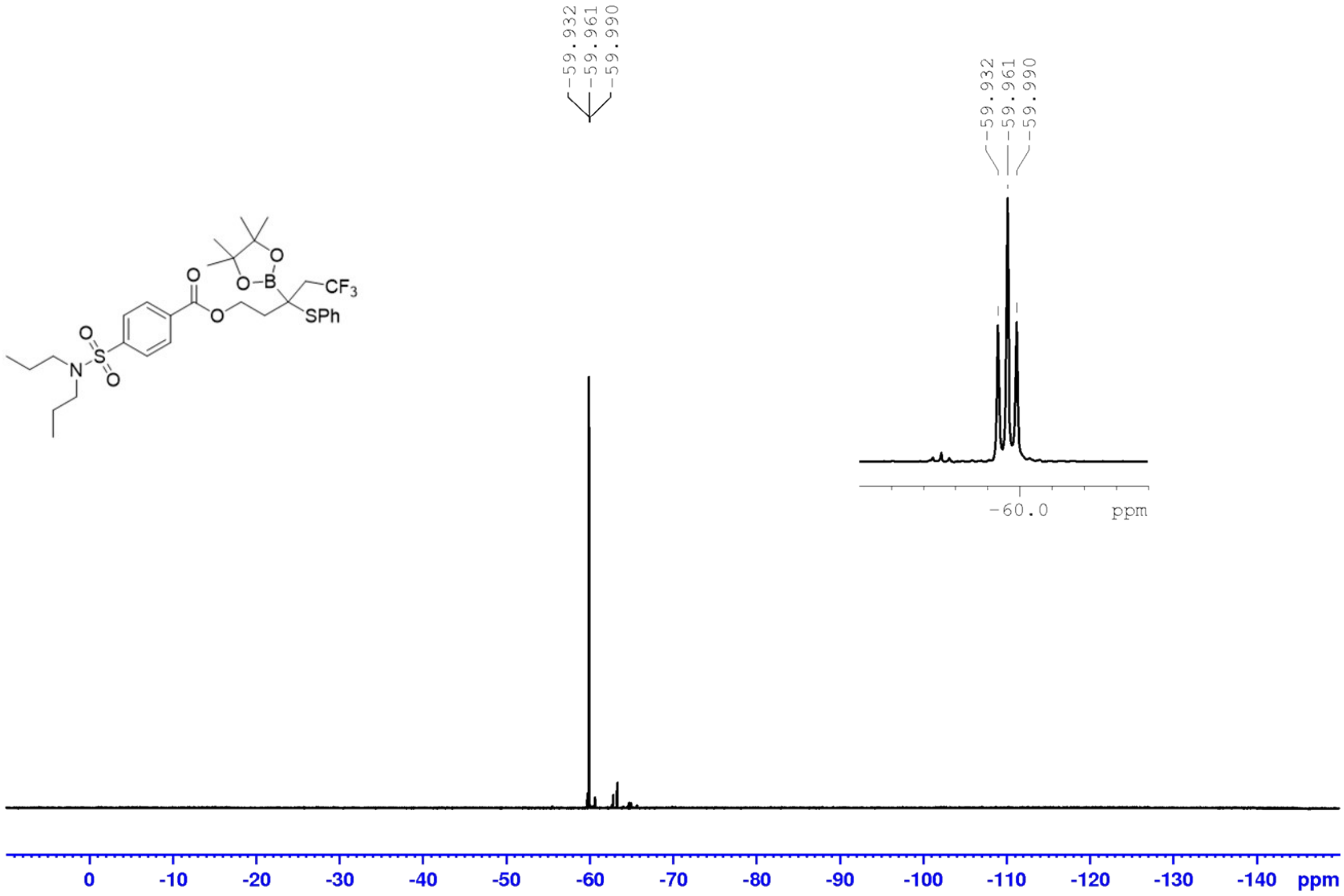

5,5,5-trifluoro-3-(phenylthio)-3-(4,4,5,5-tetramethyl-1,3,2-dioxaborolan-2-yl)pentyl 5-(2,5-dimethylphenoxy)-2,2-dimethylpentanoate (3qa)

^{1}H NMR (400 MHz, Chloroform-*d*)

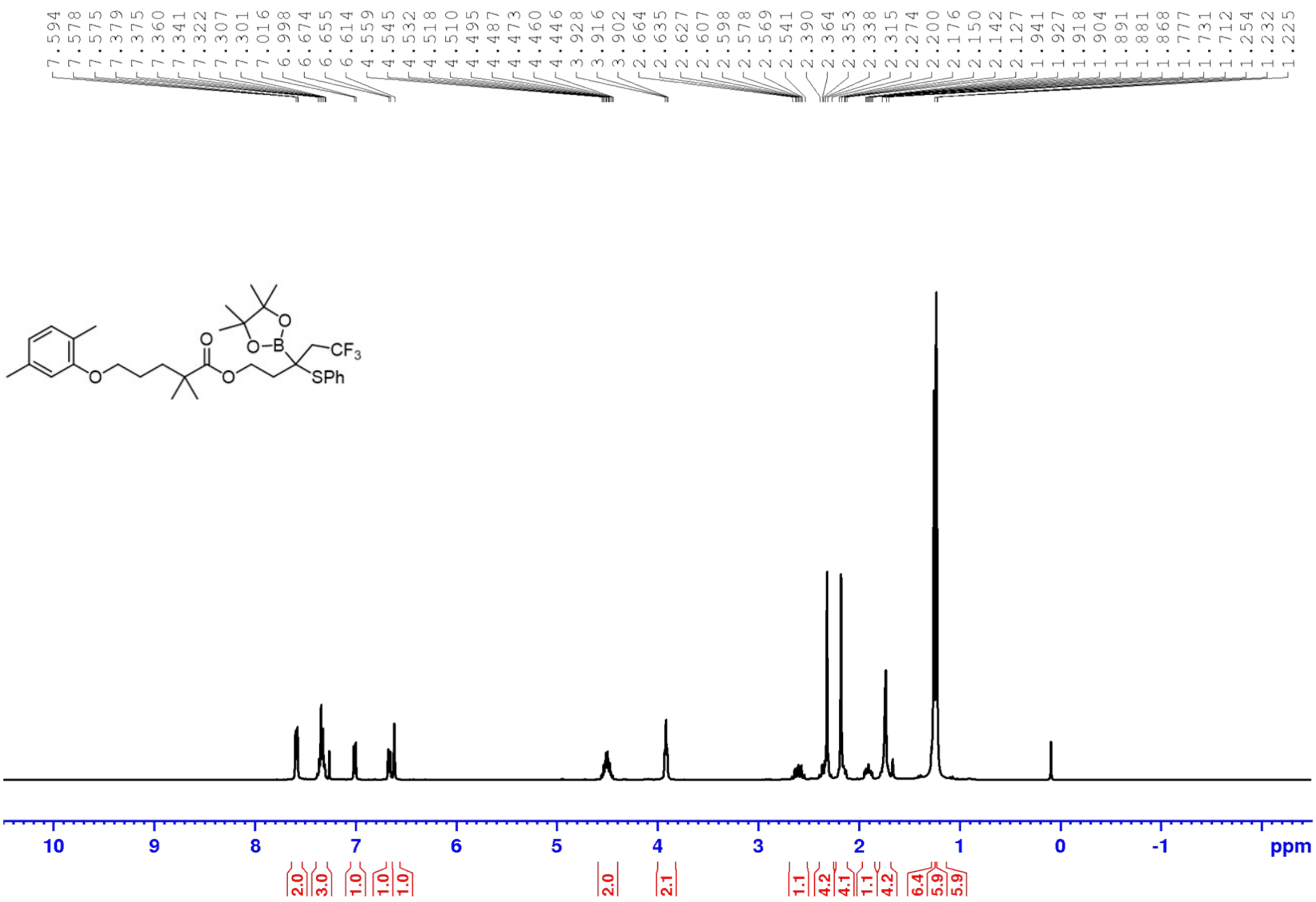

^{13}C NMR (125 MHz, Chloroform-*d*)

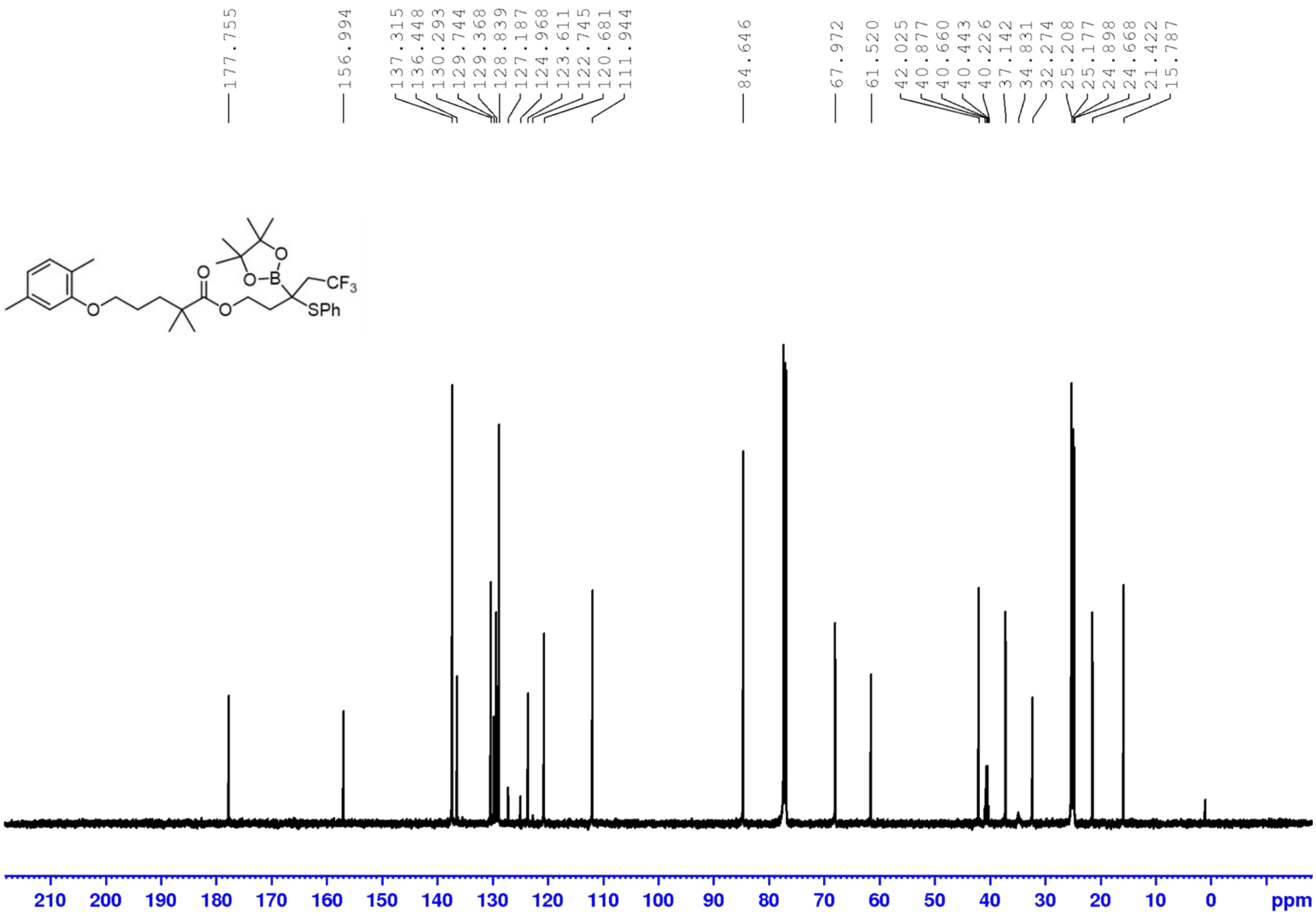

^{11}B NMR (160 MHz, Chloroform-*d*)

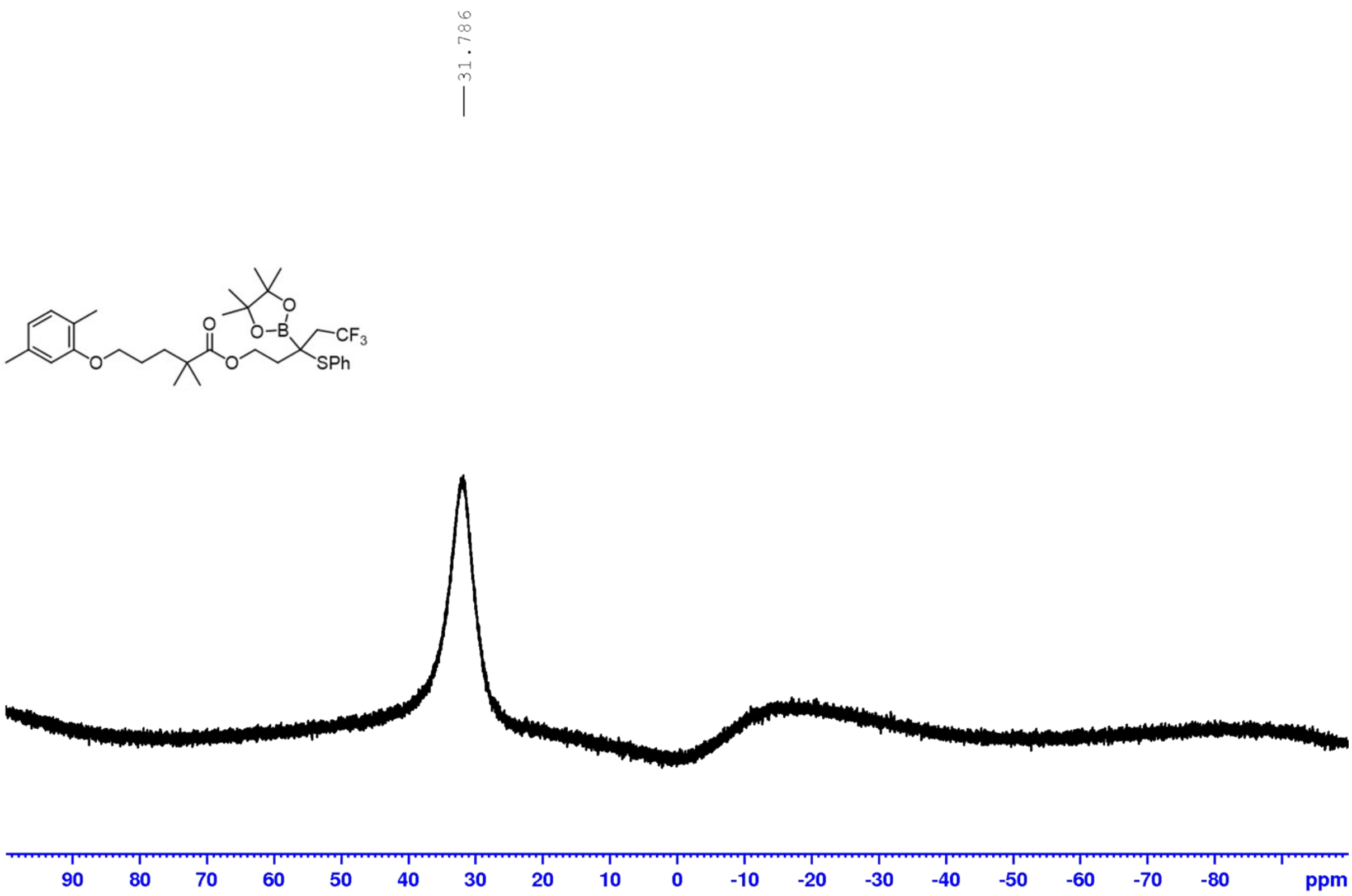

^{19}F NMR (376 MHz, Chloroform-*d*)

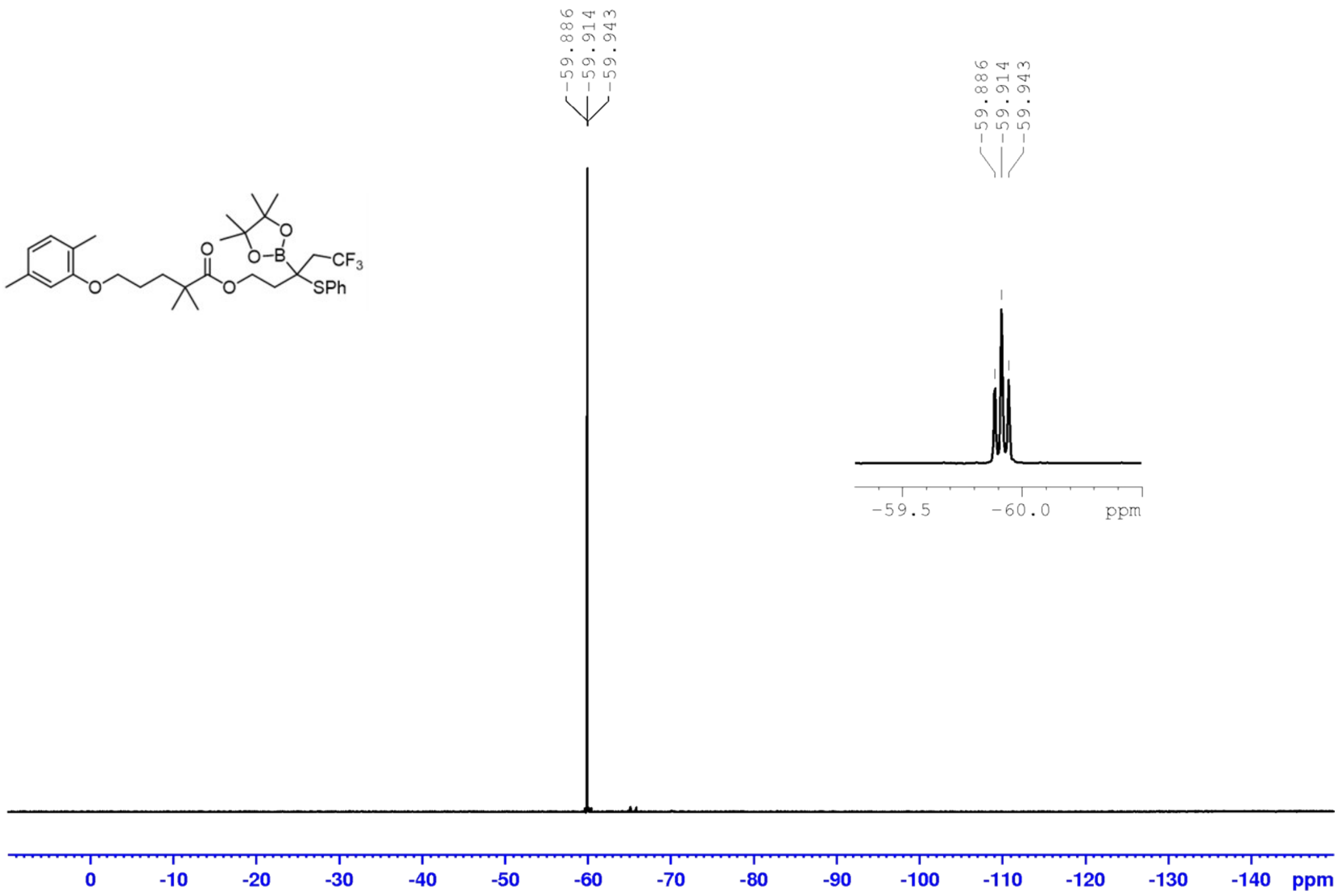

5,5,5-trifluoro-3-(phenylthio)-3-(4,4,5,5-tetramethyl-1,3,2-dioxaborolan-2-yl)pentyl 2-(4-isobutylphenyl)propanoate (3ra)

^{1}H NMR (500 MHz, Chloroform-*d*)

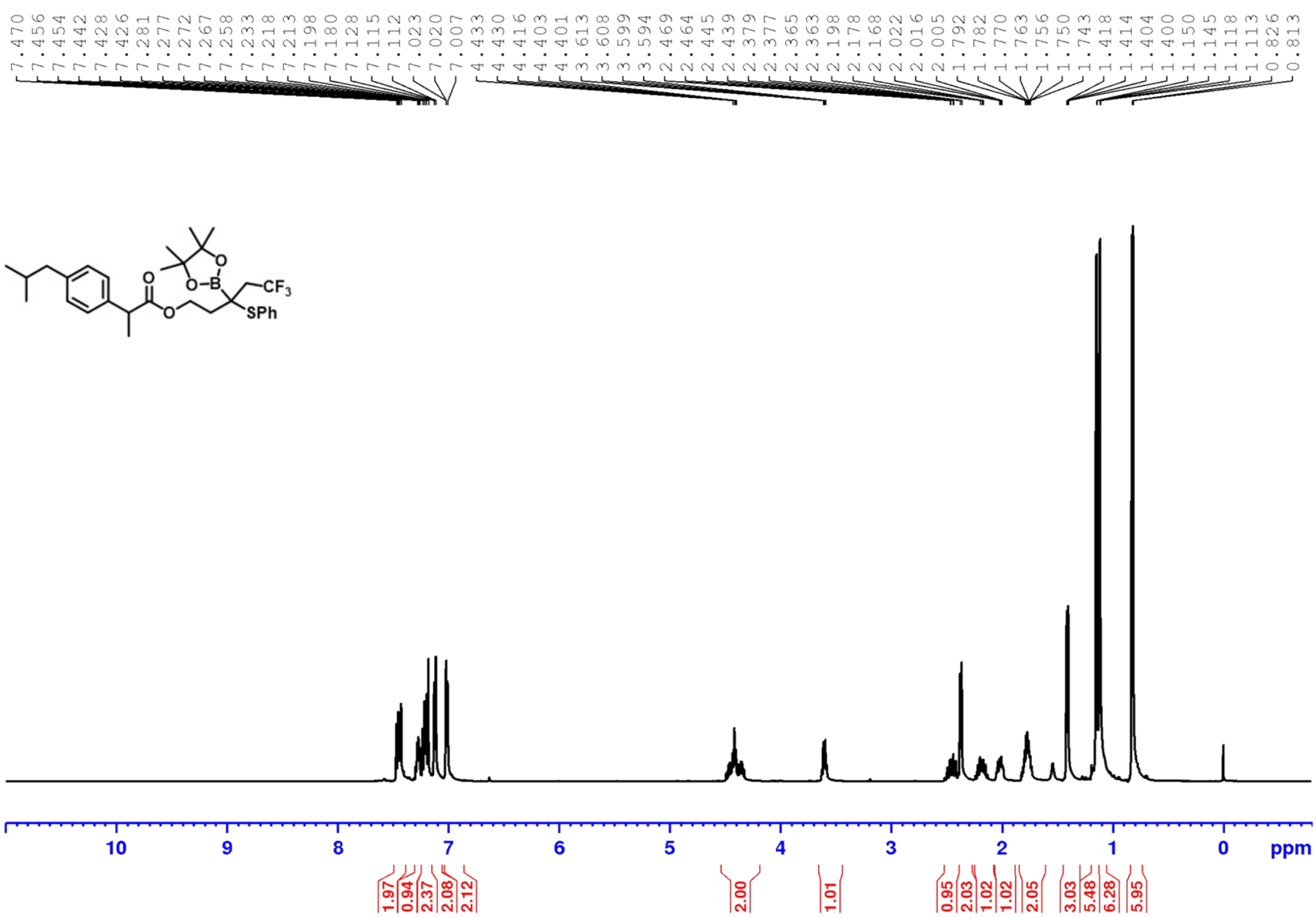

^{13}C NMR (125 MHz, Chloroform-*d*)

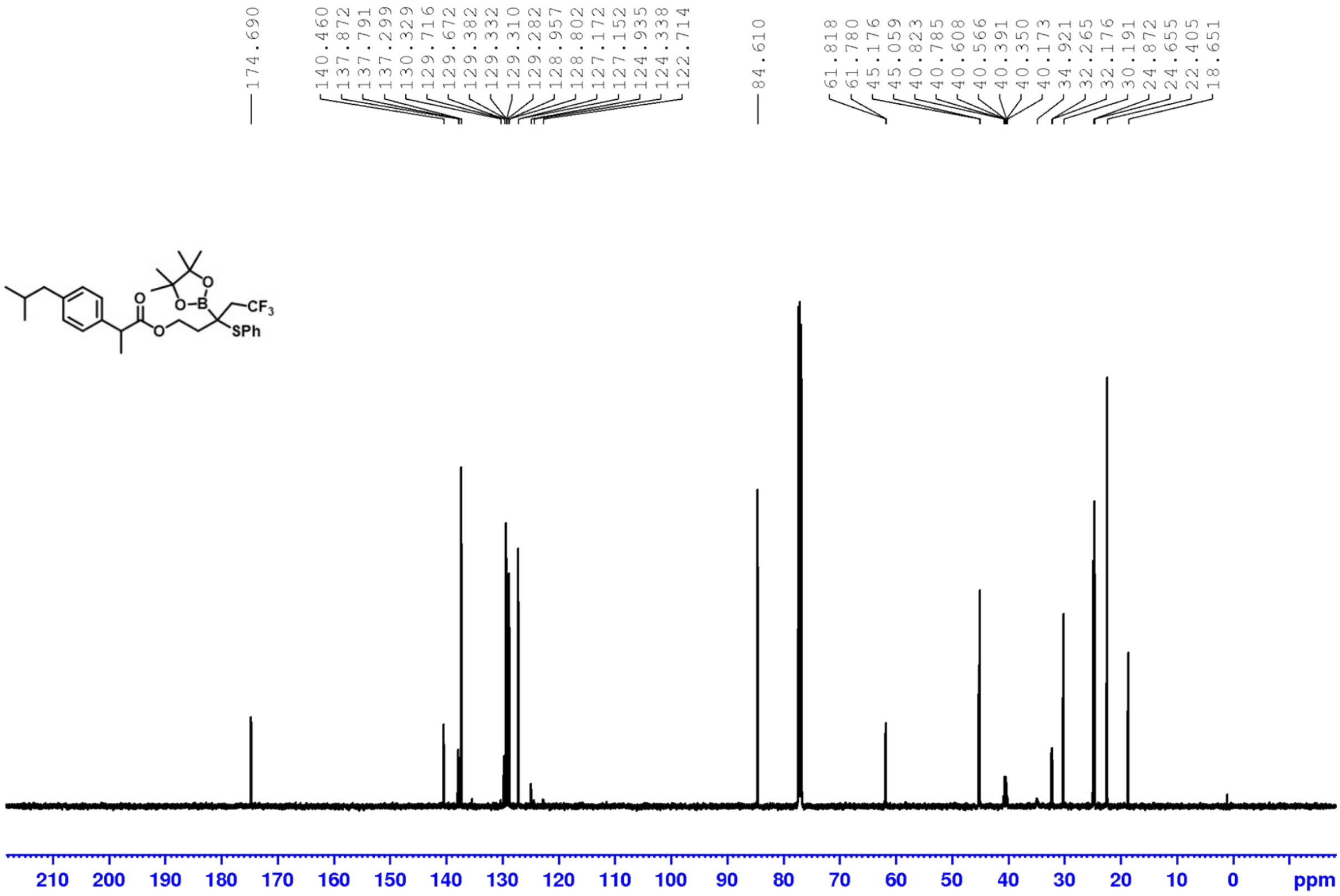

^{11}B NMR (160 MHz, Chloroform-*d*)

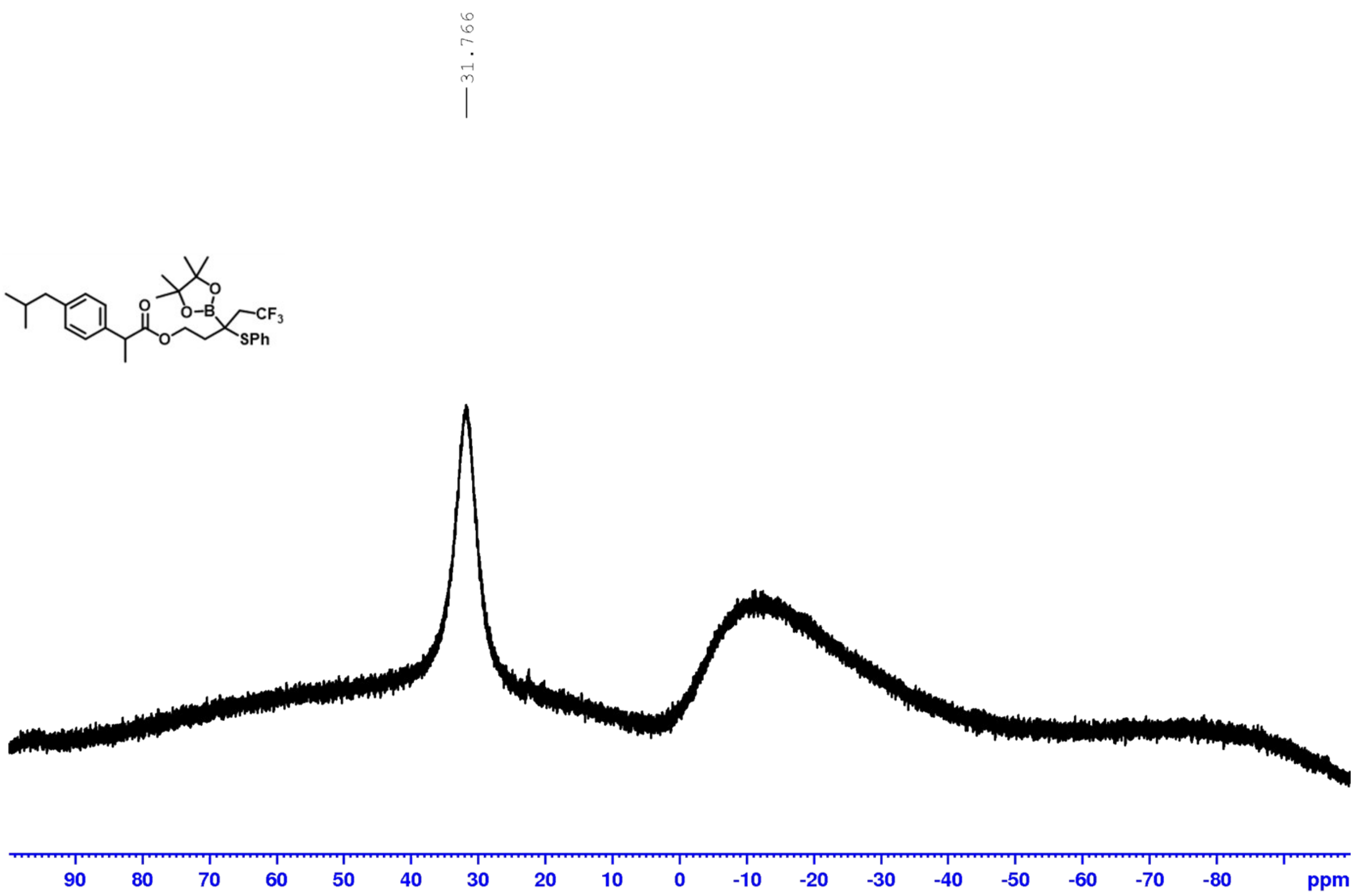

^{19}F NMR (470 MHz, Chloroform-*d*)

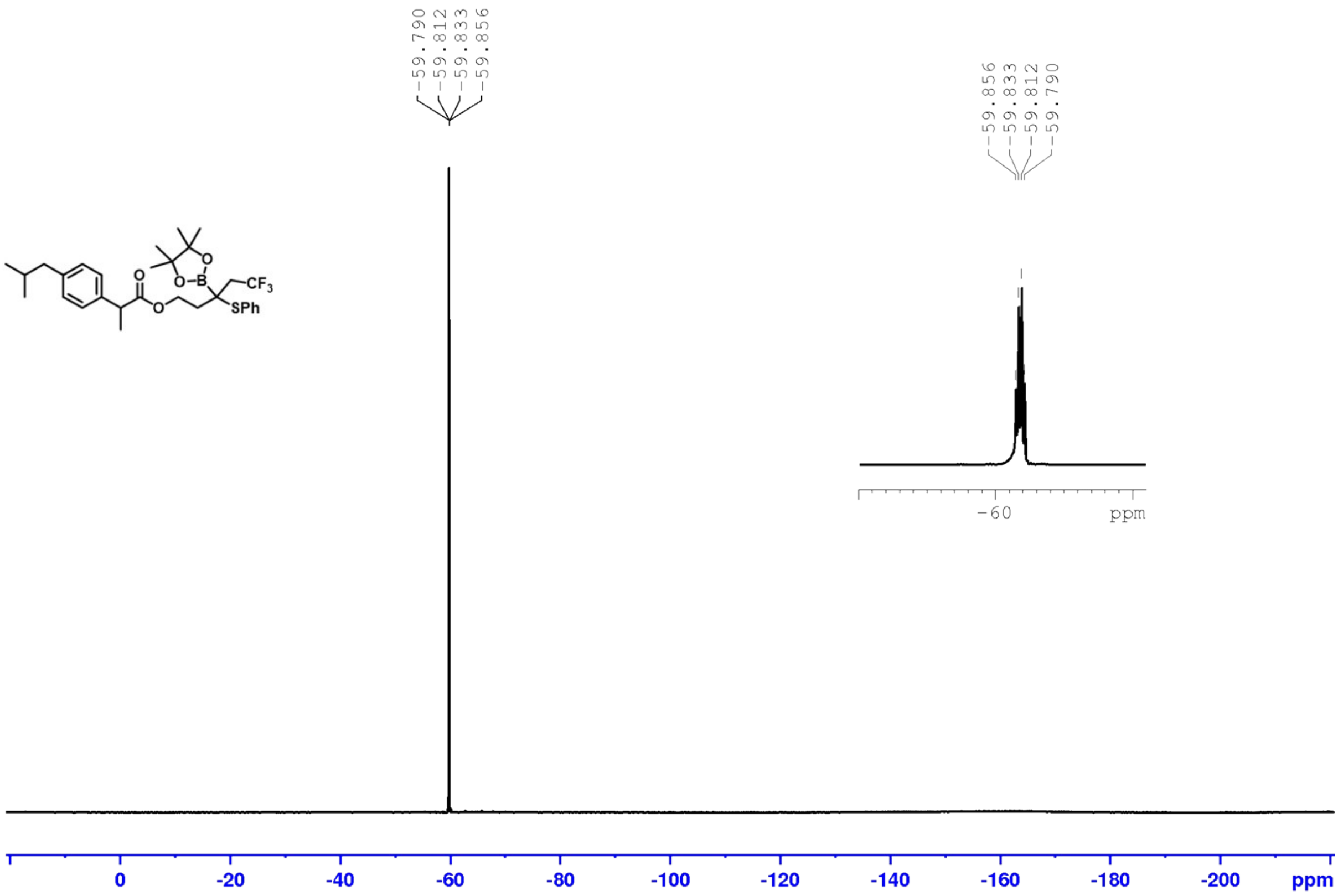

$^{19}F\{^{1}H\}$ NMR (470 MHz, Chloroform-*d*)

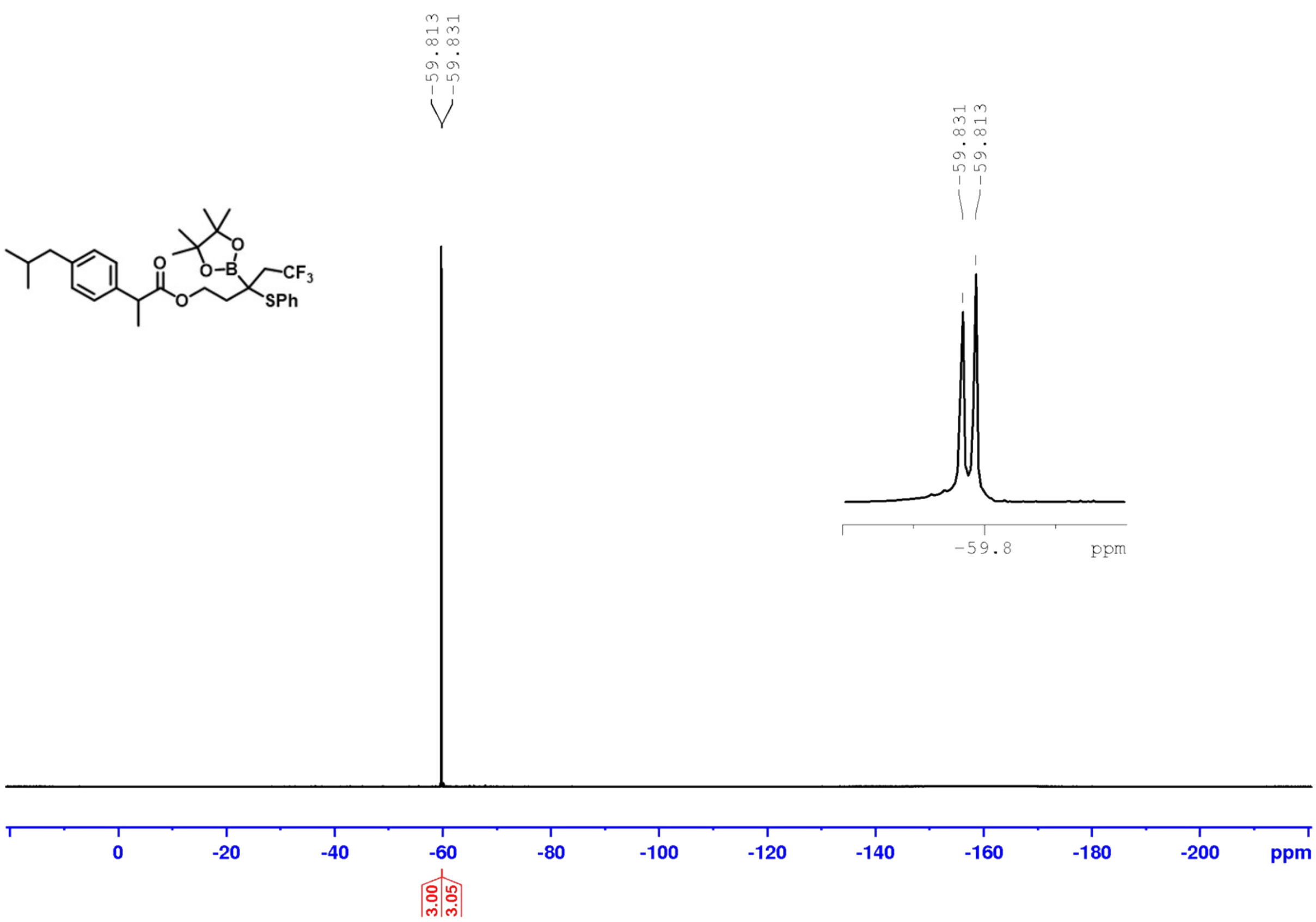

5,5,5-trifluoro-3-(phenylthio)-3-(4,4,5,5-tetramethyl-1,3,2-dioxaborolan-2-yl)pentyl (2R)-2-(6-methoxynaphthalen-2-yl)propanoate (3sa)

^{1}H NMR (500 MHz, Chloroform-*d*)

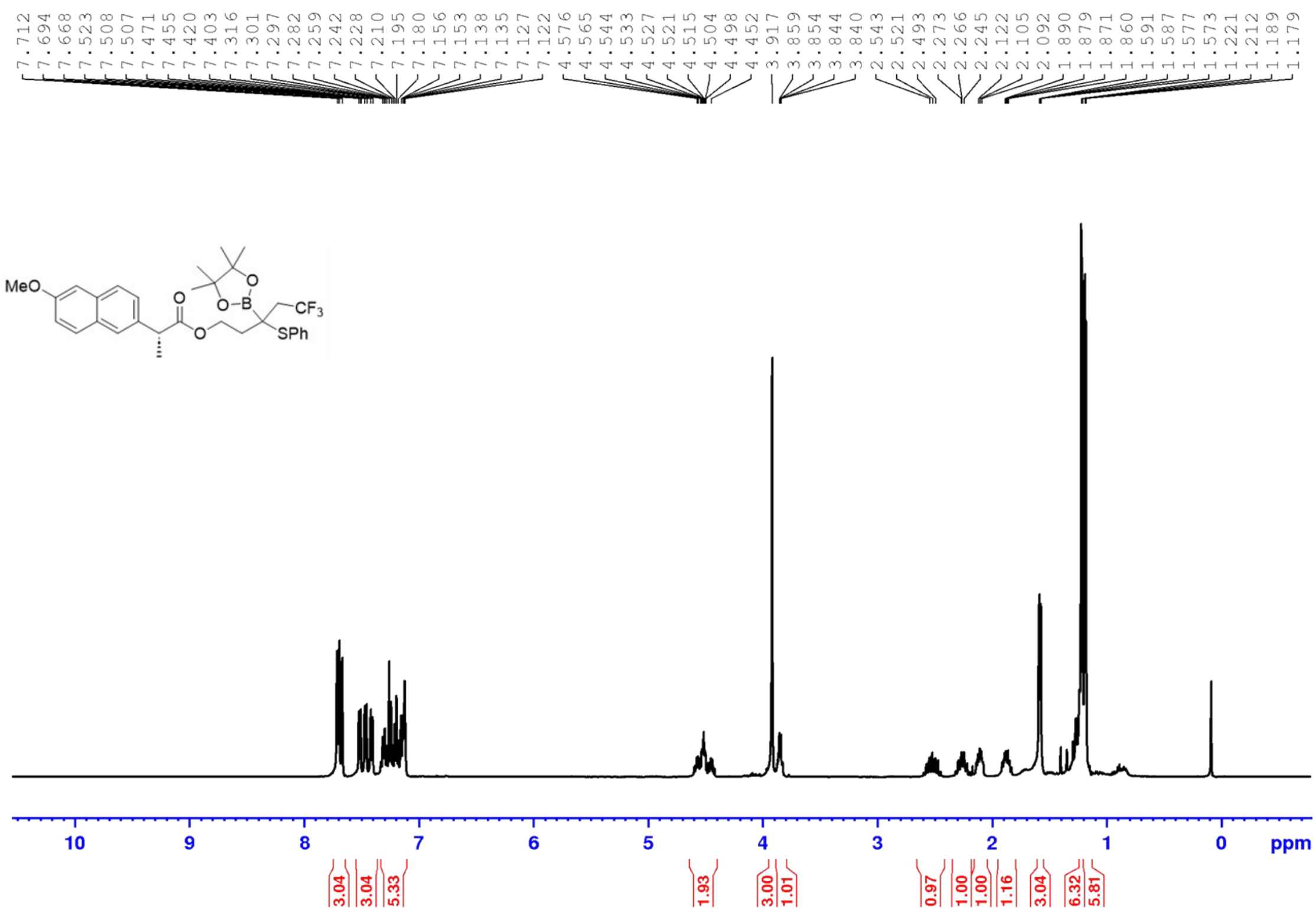

^{13}C NMR (125 MHz, Chloroform-*d*)

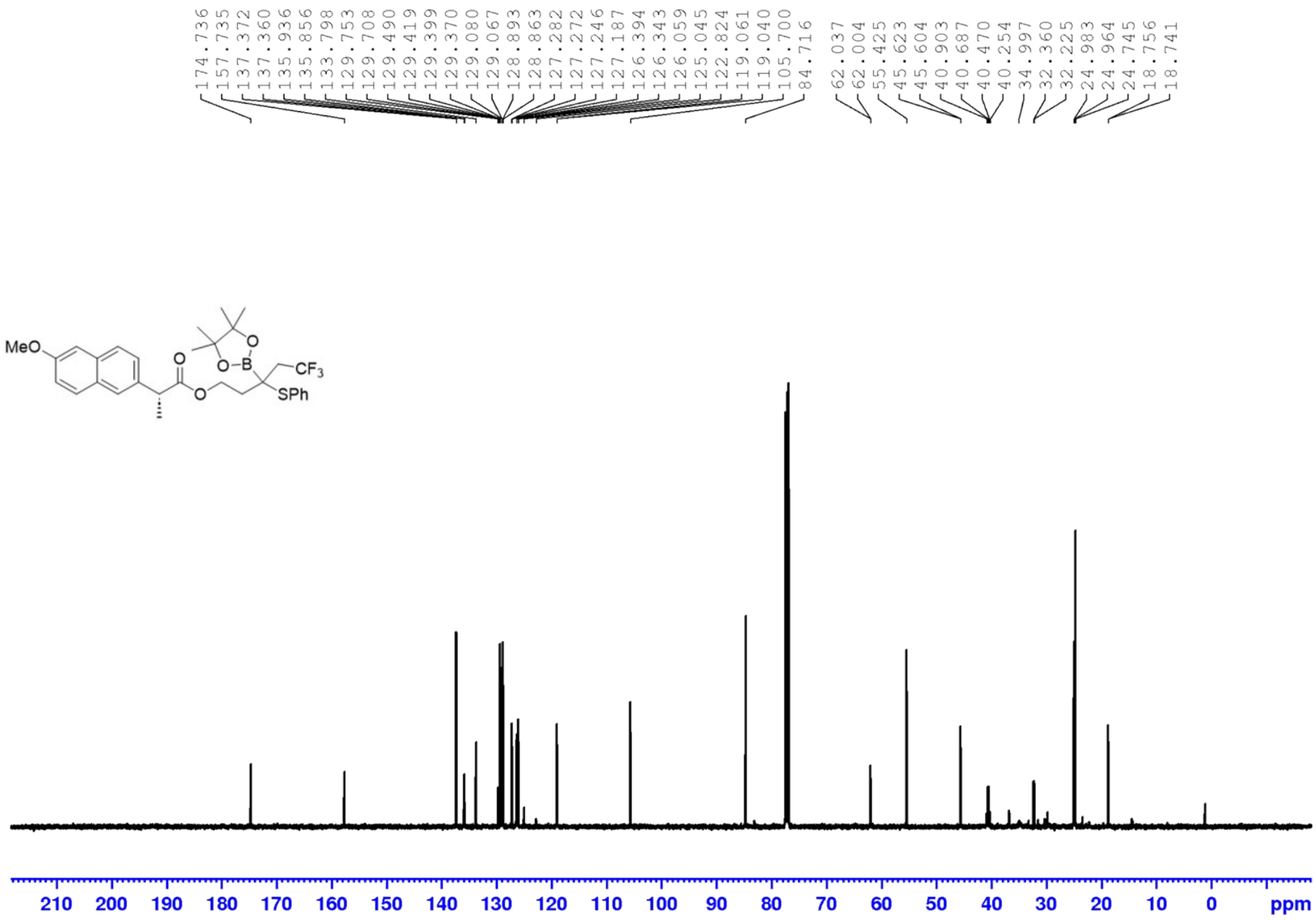

^{11}B NMR (160 MHz, Chloroform-*d*)

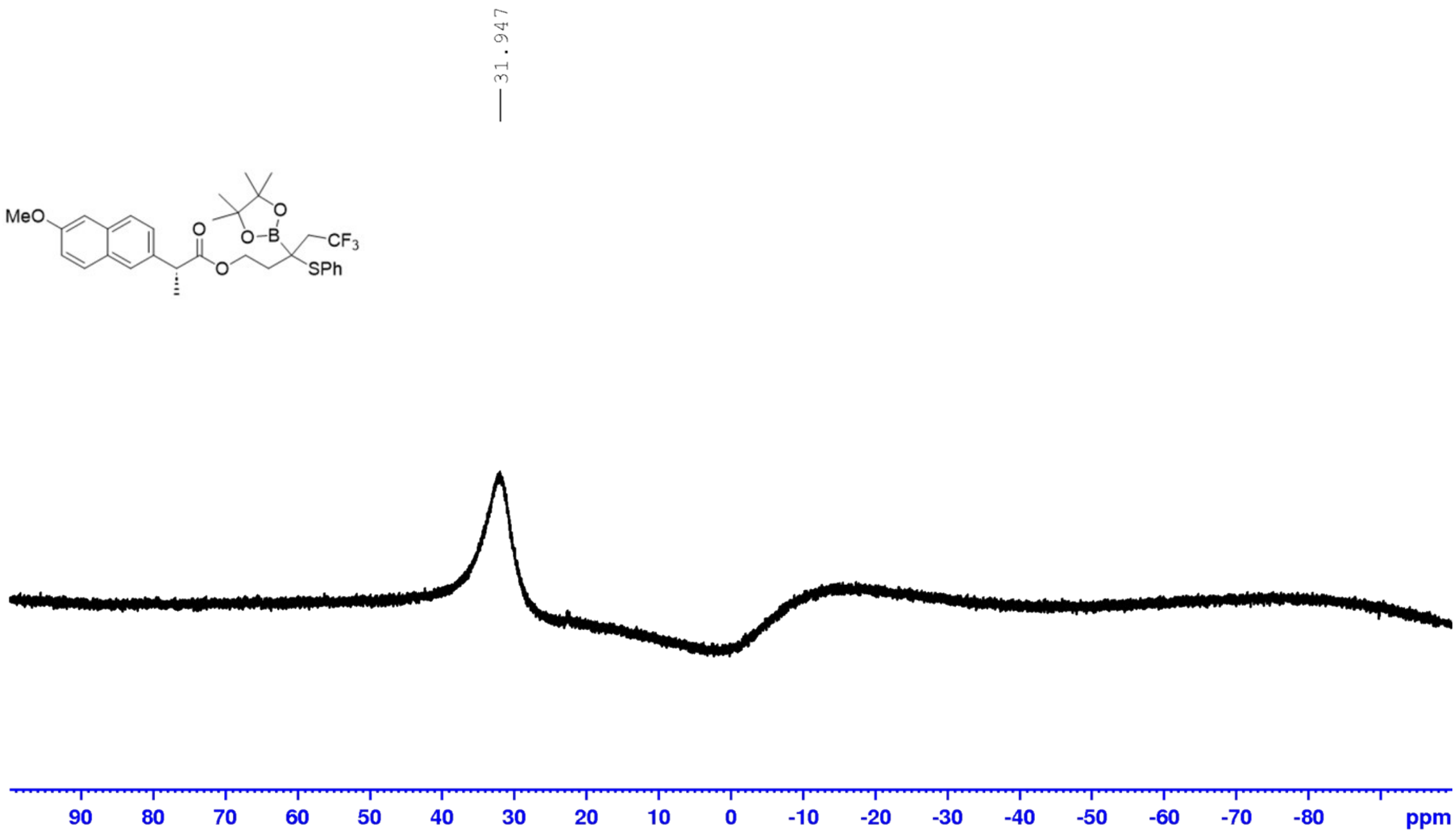

^{19}F NMR (376 MHz, Chloroform-*d*)

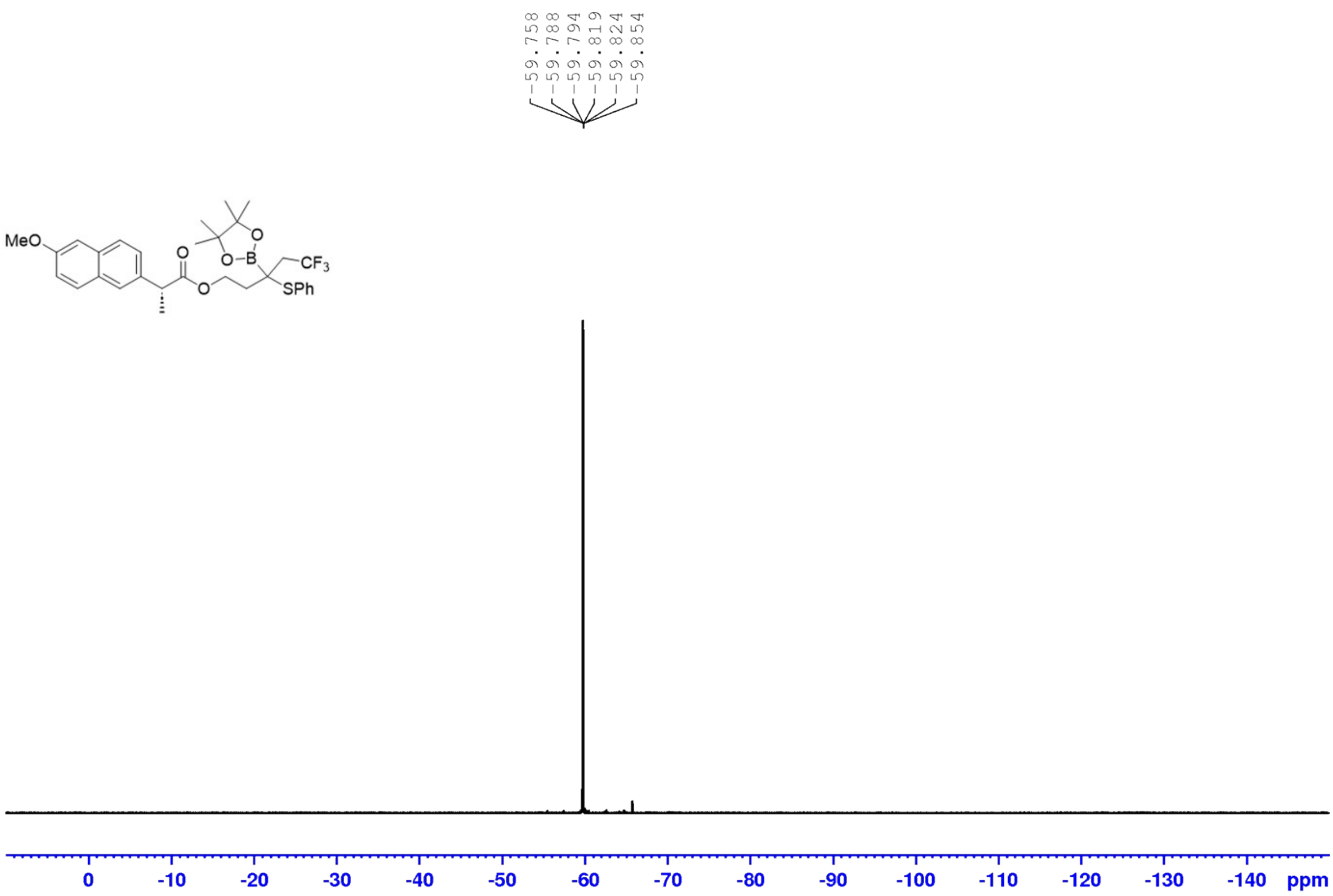

^{19}F{^{1}H} NMR (376 MHz, Chloroform-*d*)

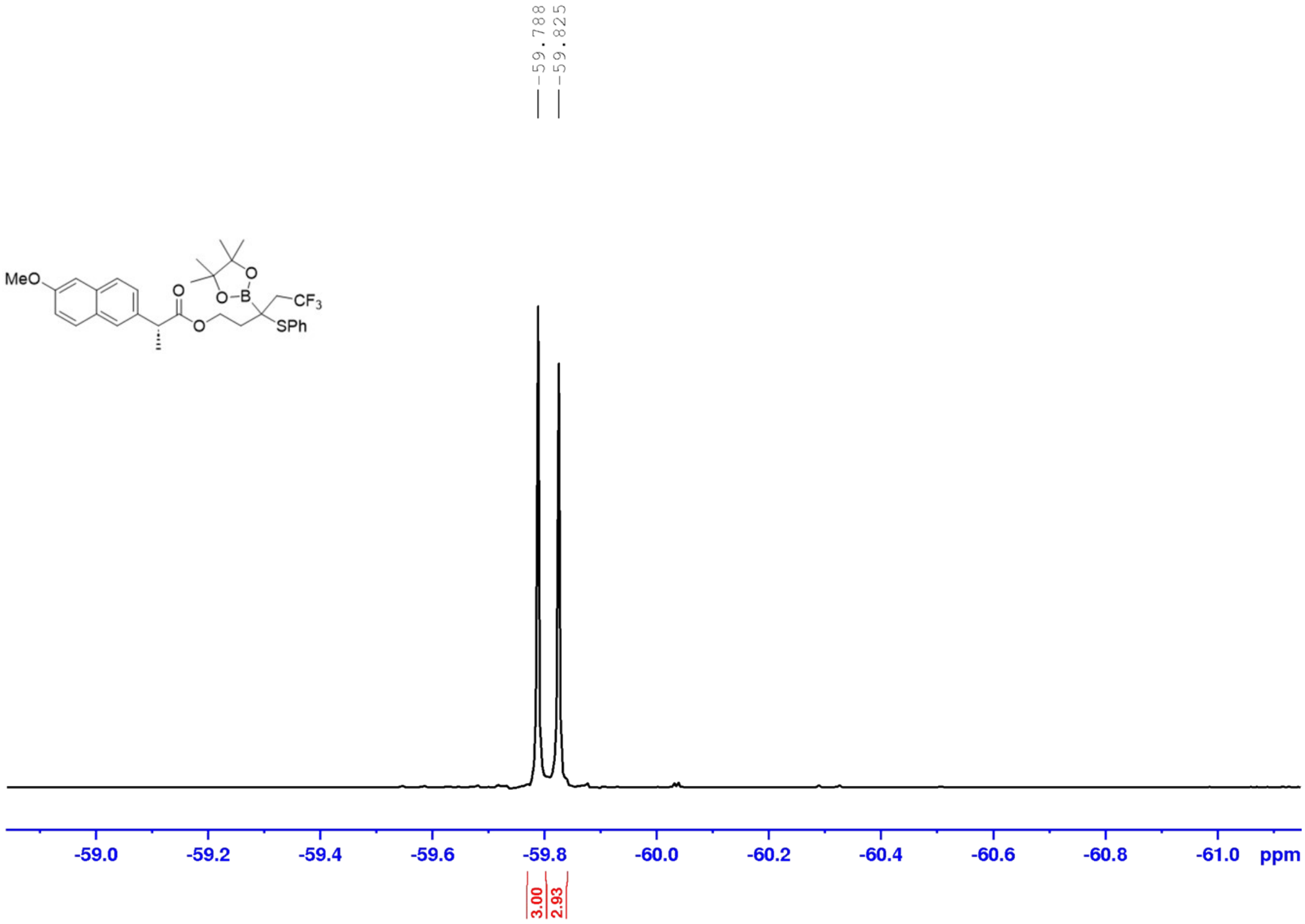

4,4,5,5-tetramethyl-2-(4,4,4-trifluoro-2-methyl-2-(phenylthio)butyl)-1,3,2-dioxaborolane (3aaa)

^{1}H NMR (400 MHz, Chloroform-*d*)

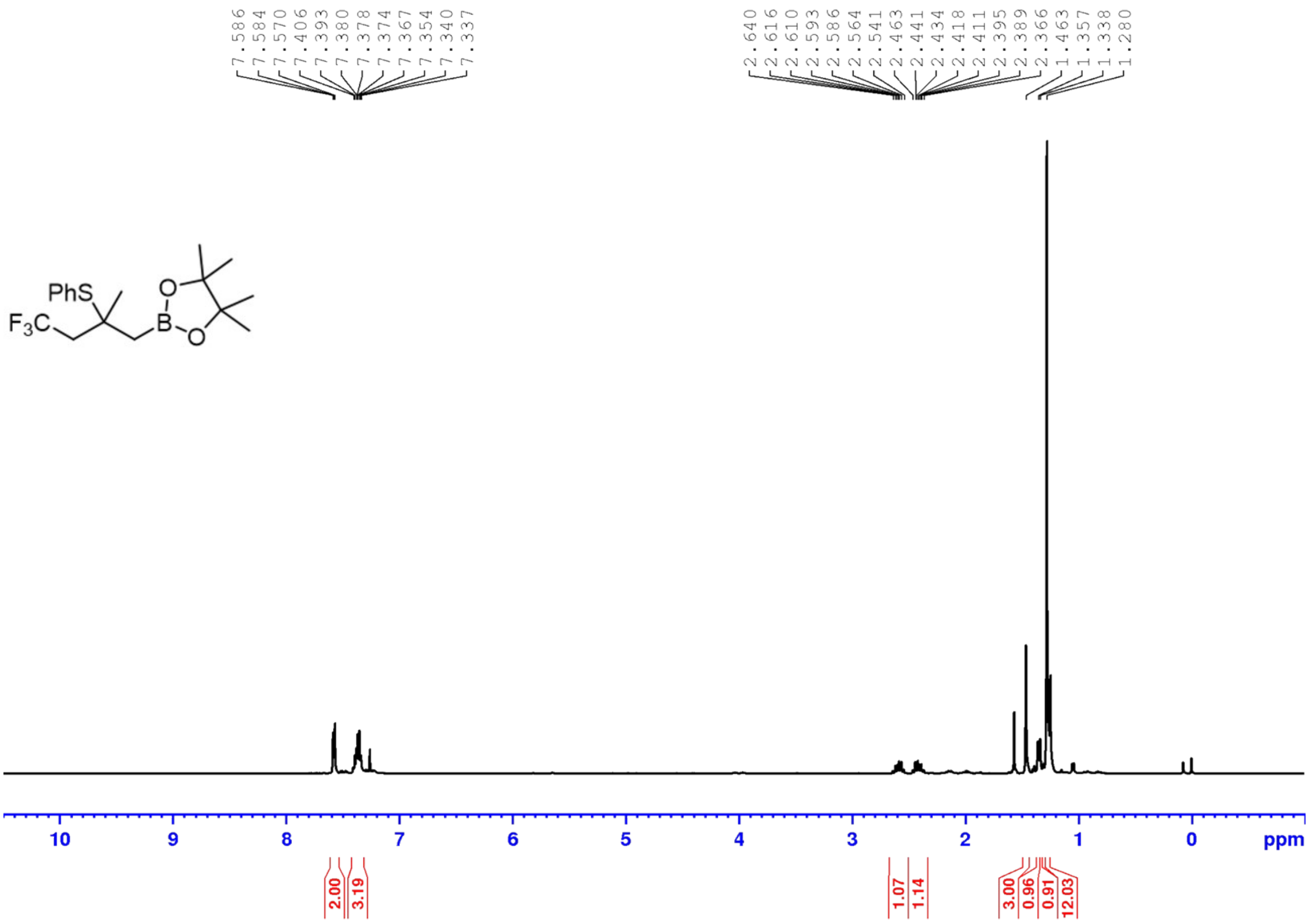

^{13}C NMR (125 MHz, Chloroform-*d*)

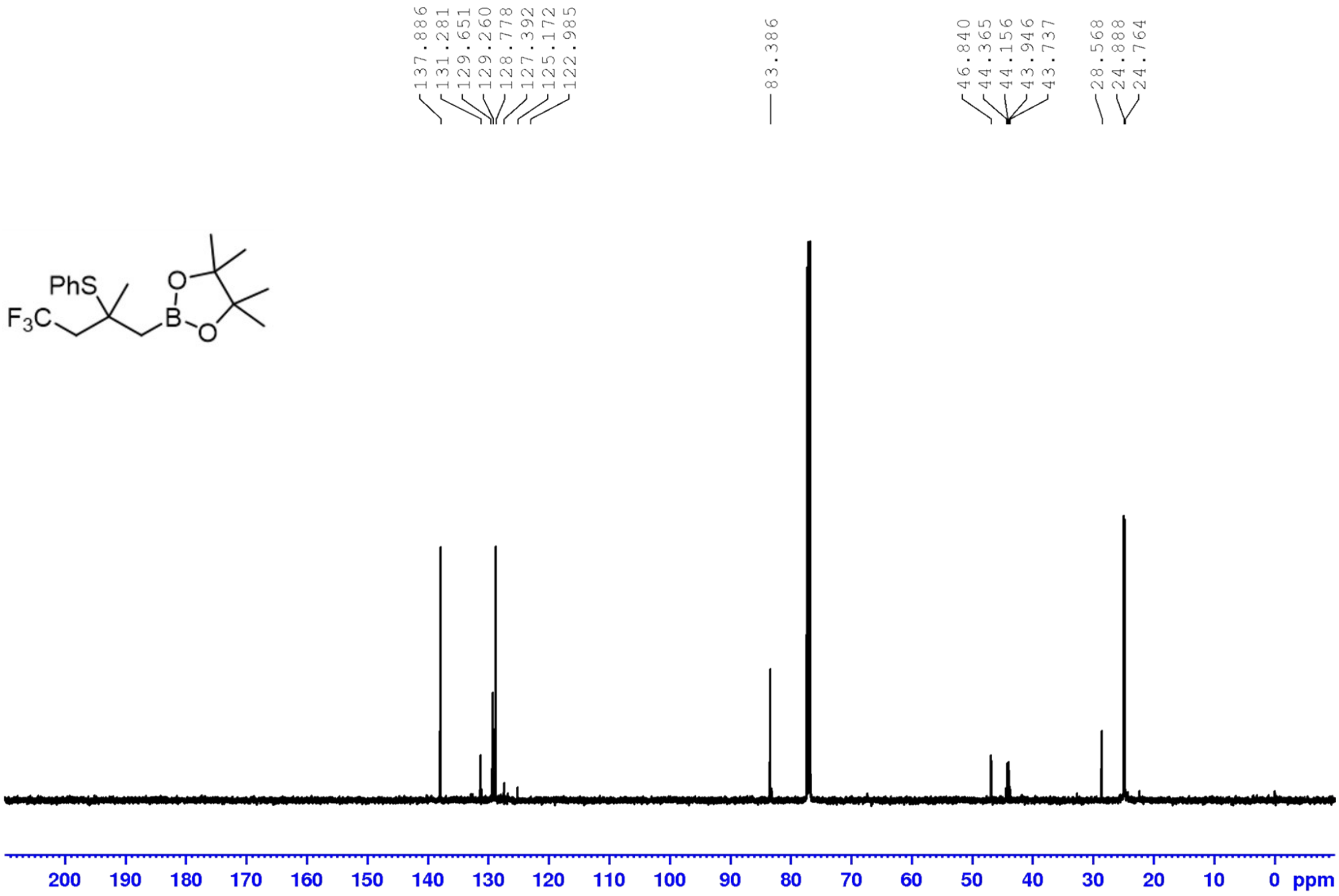

^{11}B NMR (128 MHz, Chloroform-*d*)

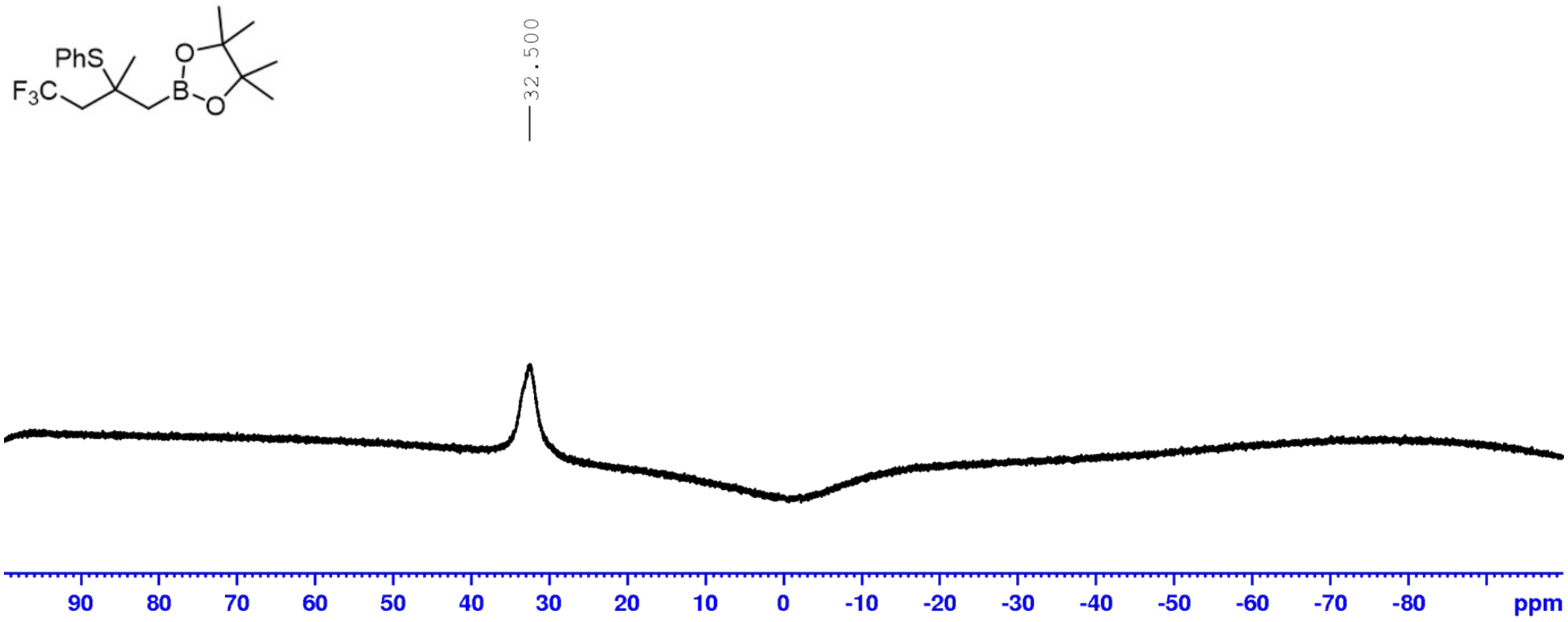

^{19}F NMR (376 MHz, Chloroform-*d*)

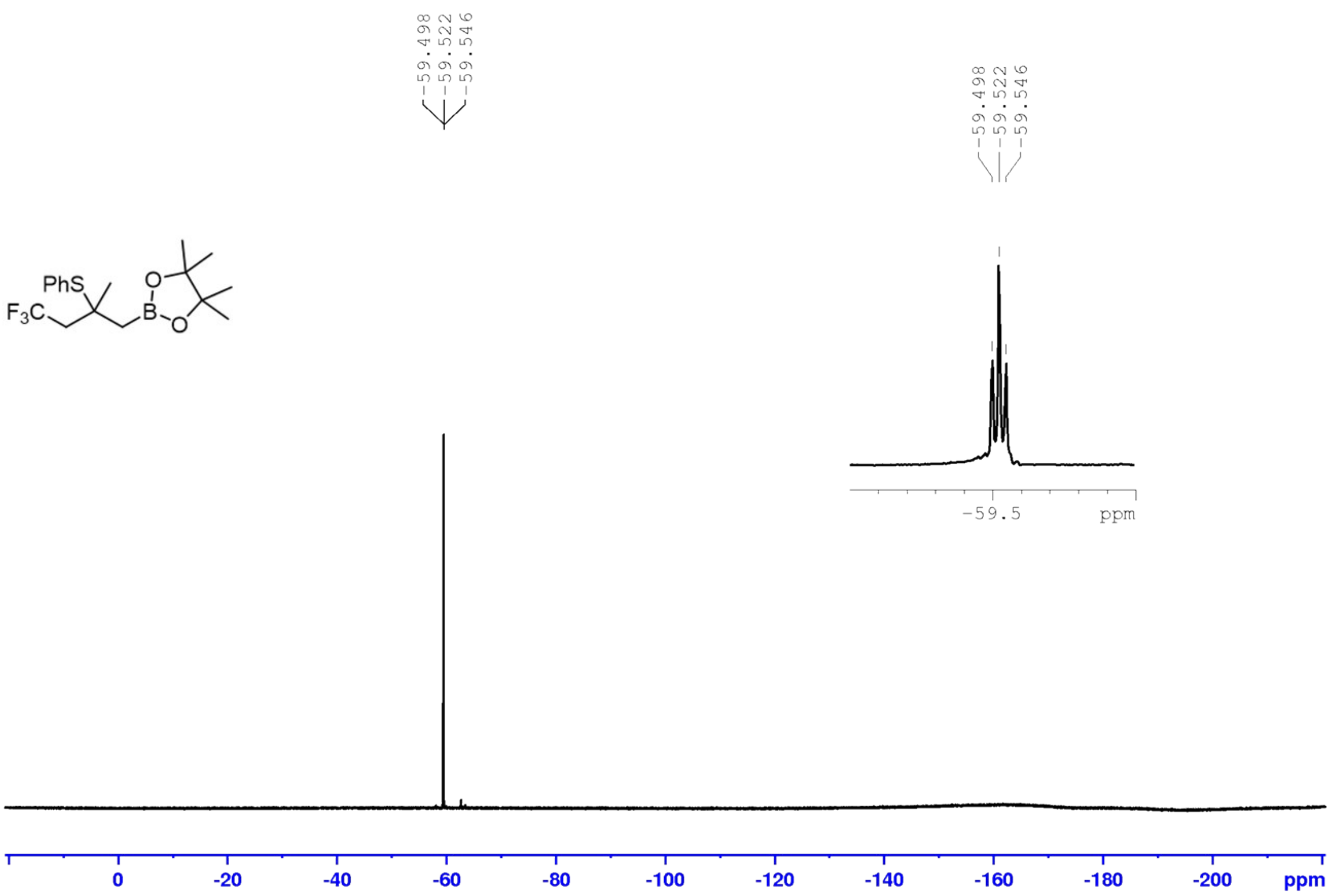

trifluoro(1,1,1-trifluoro-3-(phenylthio)-5-(4-(trifluoromethoxy)phenyl)pentan-3-yl)-l4-borane, potassium salt (3iaa)

^{1}H NMR (400 MHz, Acetone-d_6)

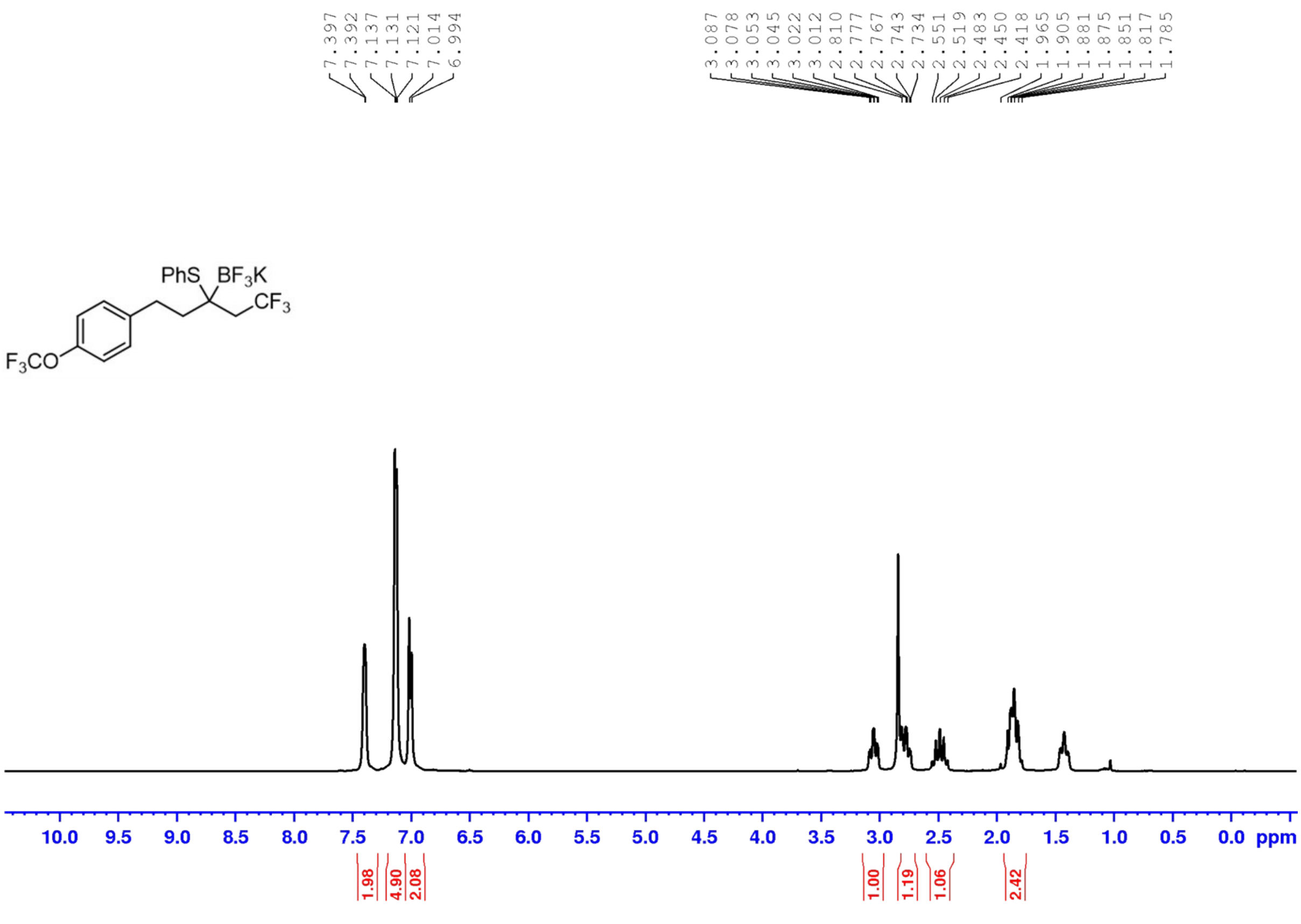

^{13}C NMR (125 MHz, Acetone-d_6)

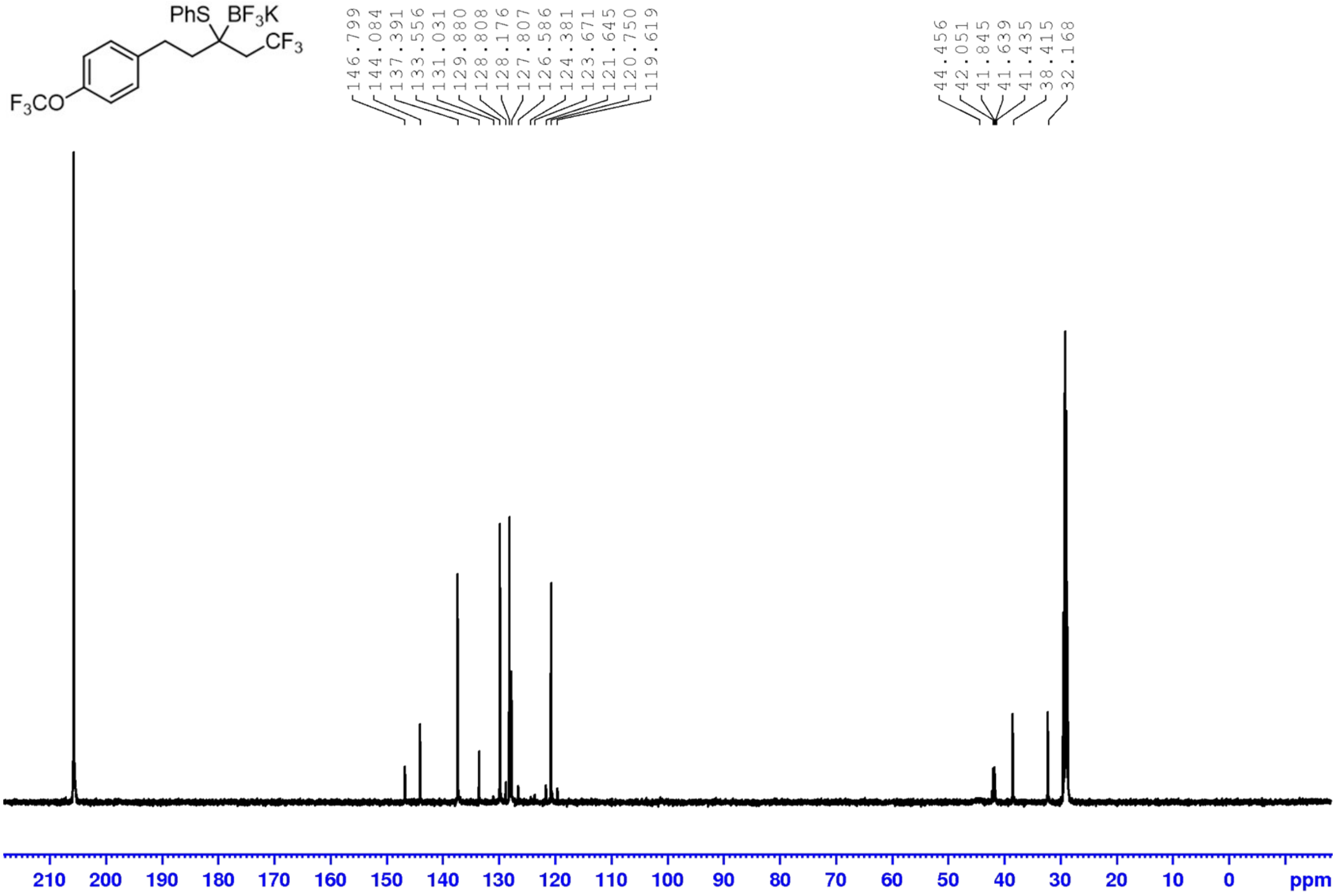

^{11}B NMR (128 MHz, Acetone-d_6)

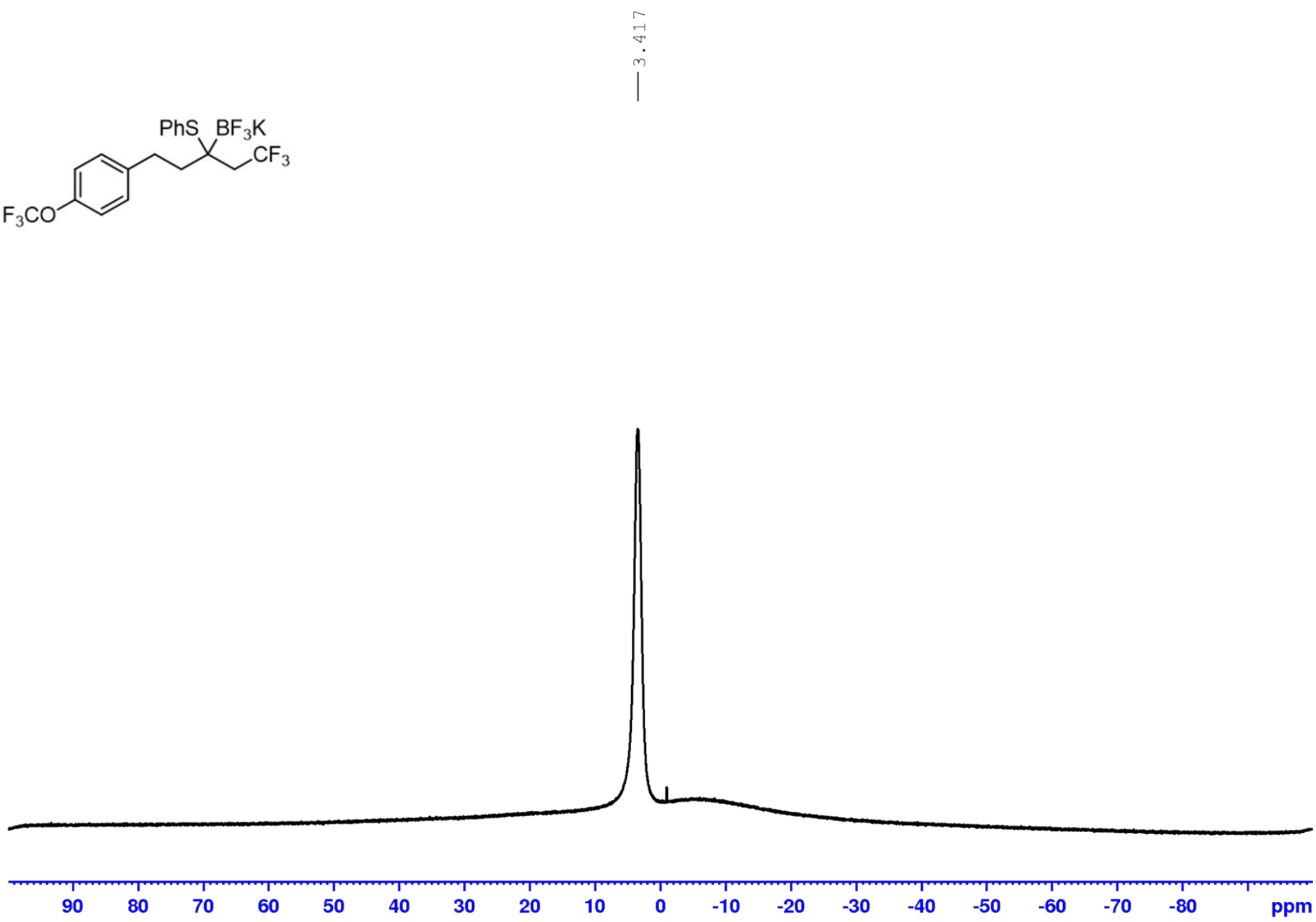

^{19}F NMR (376 MHz, Acetone-d_6)

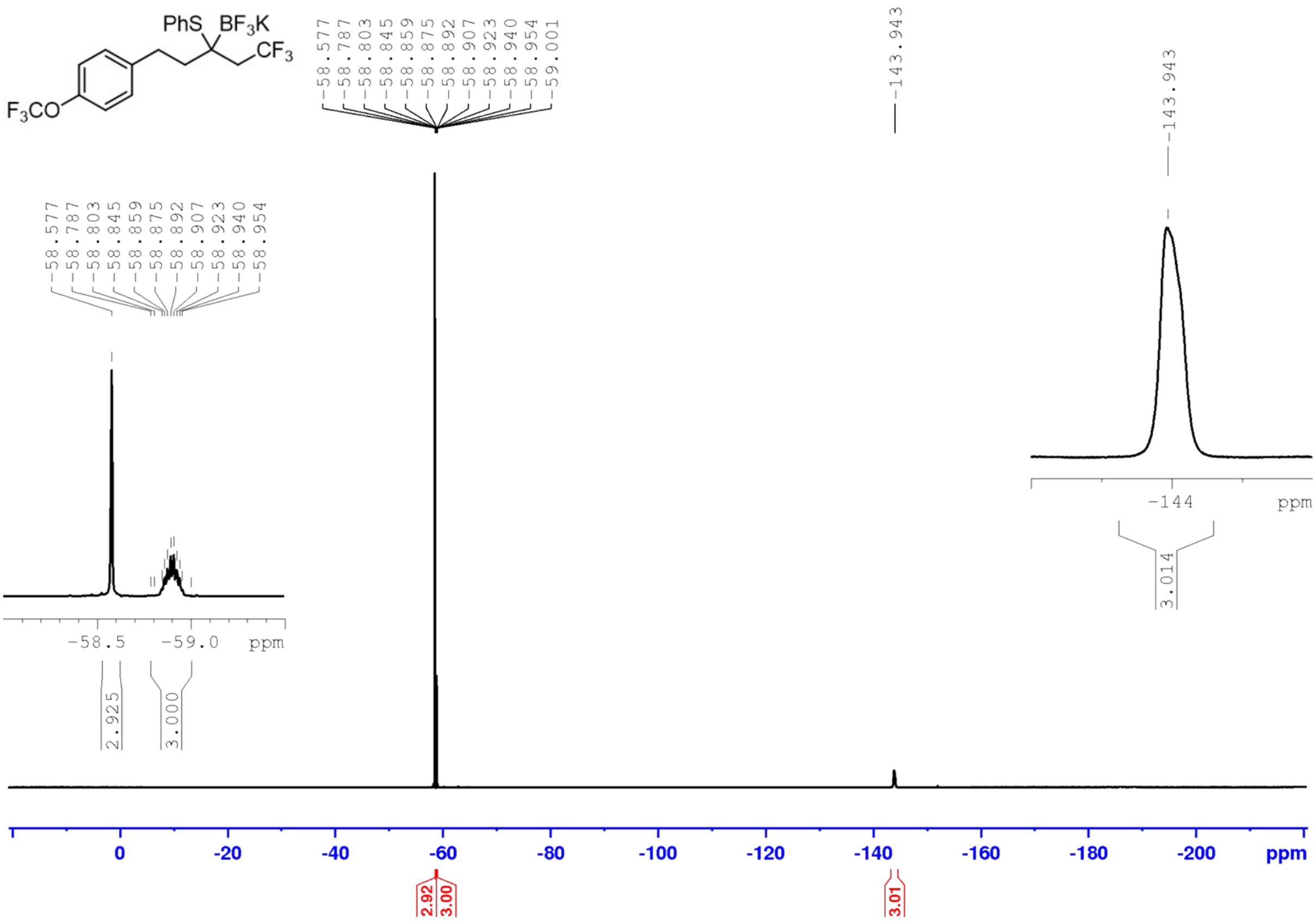

IX. References

[1] S. Ni, C. Zhu and J. Chen, *Org. Synth.*, 2013, **90**, 327–337. https://doi.org/10.1002/0471264229.os090.31

[2] W. Yuan and S. Ma, *Adv. Synth. Catal.*, 2012, **354**, 1867–1872. https://doi.org/10.1002/adsc.201100929

[3] W. J. Moran and J. P. Morken, *Org. Lett.*, 2006, **8**, 2413–2415. https://doi.org/10.1021/ol060735u

[4] J. Takagi, K. Takahashi, T. Ishiyama and N. Miyaura, *J. Am. Chem. Soc.*, 2002, **124**, 8001–8006. https://doi.org/10.1021/ja0202255.

[5] H. Sugiyama, F. Yokokawa and T. Shioiri, *Org. Lett.*, 2000, **2**, 2149–2152. https://doi.org/10.1021/ol0001281

[6] R. J. Thompson and J. C. Davis Jr., *Inorg. Chem.*, 1965, **4**, 1464–1467. https://doi.org/10.1021/ic50032a023

[7] A. D. Becke, *J. Chem. Phys.*, 1993, **98**, 5648–5652. https://doi.org/10.1063/1.464913

[8] C. Lee, W. Yang and R. G. Parr, *Phys. Rev. B*, 1988, **37**, 785. https://doi.org/10.1103/PhysRevB.37.785

[9] S. Grimme, J. Antony, S. Ehrlich and H. Krieg, *J. Chem. Phys.*, 2010, **132**, 154104. https://doi.org/10.1063/1.3382344

[10] F. Weigend and R. Ahlrichs, *Phys. Chem. Chem. Phys.*, 2005, **7**, 3297. https://doi.org/10.1039/B508541A

[11] F. Weigend, *Phys. Chem. Chem. Phys.*, 2006, **8**, 1057. https://doi.org/10.1039/B515623H

[12] A. Klamt and G. Schüürmann, *J. Chem. Soc., Perkin Trans. 2*, 1993, 799–805. https://doi.org/10.1039/P29930000799

[13] J. Andzelm, C. Kölmel and A. Klamt, *J. Chem. Phys.*, 1995, **103**, 9312–9320. https://doi.org/10.1063/1.469990

[14] V. Barone and M. Cossi, *J. Phys. Chem. A*, 1998, **102**, 1995–2001. https://doi.org/10.1021/jp9716997

[15] M. Cossi, N. Rega, G. Scalmani and V. Barone, *J. Comput. Chem.*, 2003, **24**, 669–681. https://doi.org/10.1002/jcc.10189

[16] R. Dennington, T. A. Keith and J. M. Millam, *GaussView, Version 6*, Semichem Inc., Shawnee Mission, KS, 2016. https://gaussian.com/gaussview6/

[17] *Chemcraft—Graphical Software for Visualization of Quantum Chemistry Computations, Version 1.8, build 682*, https://www.chemcraftprog.com

[18] *Avogadro—An Open-Source Molecular Builder and Visualization Tool, Version 1.2.0*, http://avogadro.cc.

[19] M. D. Hanwell, D. E. Curtis, D. C. Lonie, T. Vandermeersch, E. Zurek and G. R. Hutchison, *J. Cheminf.*, 2012, **4**, 17. https://doi.org/10.1186/1758-2946-4-17
